# Integrative DNA methylome and transcriptome analysis reveals DNA adenine methylation is involved in *Salmonella enterica* Typhimurium response to oxidative stress

**DOI:** 10.1128/spectrum.02479-23

**Published:** 2023-10-26

**Authors:** Wenting Zhang, Lin Lyu, Zhihiong Xu, Jinjing Ni, Danni Wang, Jie Lu, Yu-Feng Yao

**Affiliations:** 1 Laboratory of Bacterial Pathogenesis, Shanghai Institute of Immunology, Shanghai Jiao Tong University School of Medicine, Shanghai, China; 2 Department of Infectious Diseases, Ruijin Hospital, Shanghai Jiao Tong University School of Medicine, Shanghai, China; 3 State Key Laboratory of Microbial Metabolism, School of Life Sciences and Biotechnology, Shanghai Jiao Tong University, Shanghai, China; 4 Shanghai Key Laboratory of Emergency Prevention, Diagnosis and Treatment of Respiratory Infectious Diseases, Shanghai, China; South China Sea Institute of Oceanology Chinese Academy of Sciences, Guangzhou, Guangdong, China

**Keywords:** *Salmonella *Typhimurium, DNA methylation, oxidative stress

## Abstract

*Salmonella* could survive and replicate in macrophages, where it encounters multiple stresses. Deficiency of a DNA adenine methyltransferase impairs the survival of *Salmonella enterica* serovar Typhimurium (*S*. Typhimurium) in hydrogen peroxide. To investigate whether DNA methylation is involved in the expression of oxidative stress-responsive genes, we combined RNA-seq and single-molecule real-time sequencing to integrate transcriptome and methylome analysis. Here, we show that (i) the entire amount of m6A GATC remains stable during oxidative stress; (ii) no significant association is observed between DNA methylation and transcription level in most genes; (iii) coincidence of level change between transcription and m6A GATC in the regulatory regions is identified in 49 genes under oxidative stress. Some of them are known to contribute to bacterial defenses against oxidative stress through reducing H_2_O_2_ levels, directing aberrant protein product degradation and inducing outer membrane protein expression. Specifically, the transcription level of *ahpCF* is negatively correlated to the m6A GATC level in its regulatory region. The transcription of *smpB* is elevated along with the decrease in m6A level at position 2,879,954 on the minus strand. In contrast, mRNA levels of *STM14_2773* and *ycfR* are positively correlated with m6A GATC content in their regulatory regions. Highly stable DNA methylome and coupled change of m6A GATC with gene expression in specific positions suggest that DNA methylation homeostasis at genome-wide and plasticity in specific regions are crucial for bacterial response to oxidative stress. These findings provide new insights into the epigenetic regulatory mechanisms of gene expression in *S*. Typhimurium in the host microenvironment.

**IMPORTANCE:**

The intracellular pathogen *Salmonella enterica* serovar Typhimurium (*S*. Typhimurium) comes across a wide variety of stresses from entry to dissemination, such as reactive oxygen species. To adapt itself to oxidative stress, *Salmonella* must adopt various and complex strategies. In this study, we revealed that DNA adenine methyltransferase was essential for *S*. Typhimurium to survive in hydrogen peroxide. We then screened out oxidative stress-responsive genes that were potentially regulated by DNA methylation in *S*. Typhimurium. Our results show that the DNA methylome is highly stable throughout the genome, and the coupled change of m6A GATC with gene expression is identified in only a few positions, which suggests the complexity of the DNA methylation and gene expression regulation networks. The results may shed light on our understanding of m6A-mediated gene expression regulation in bacteria.

## INTRODUCTION

Bacteria harbor a diverse group of enzymes named DNA methyltransferases (MTases), which are able to insert epigenetic methylation modifications into DNA bases in a sequence-dependent manner (1). To date, three different forms of DNA methylation have been found in bacteria: N6-methyladenine (m6A), N4-methylcytosine (m4C), and 5-methylcytosine (m5C) (2). m6A is the most prevalent, abundant, and conserved DNA modification in bacteria, while m4C occurs less frequently than m6A in all bacteria. m4C has been observed more often in thermophilic bacteria than in non-thermophilic bacteria, possibly because it is substantially more resistant to heat-induced deamination than m5C (3).

The foodborne pathogenic bacteria *Salmonella* could cause gastroenteritis and typhoid fever in humans across the world (4). This intracellular pathogen comes across a wide variety of stresses from entry to dissemination, such as low pH in the gastrointestinal tract, antimicrobial peptides, and reactive oxygen species (ROS) (5). *Salmonella* is exposed to ROS such as H_2_O_2_ and O^2−^ from respiratory bursts in the phagocytic cells. ROS could cause damage to biomolecules like nucleic acids, carbohydrates, proteins, and lipids in *Salmonella* (6). To adapt itself to oxidative stress, *Salmonella* must adopt various and complex strategies, including the degradation of ROS before they act on target molecules, balancing periplasmic disulfide bond formation, and activating redox sensing regulatory systems (OxyR/SoxR regulons) (7).

*Salmonella enterica* serovar Typhimurium (*S*. Typhimurium) possesses several kinds of MTases. For example, DNA adenine methyltransferase (Dam) catalyzes the methylation of the adenine in the sequence 5′-GATC-3′ (m6A) (8). Mod and HsdM catalyze the methylation of the adenine in the sequence 5′-CGAAT-3′ (9) and 5′-AACGTGC-3′ (m6A) (10), respectively. Dcm is responsible for catalyzing the methylation of the 2nd cytosine in the sequence 5′-CCWGG-3′ (m5C) (11). STM14_1435 is a putative N4-methylcytosine DNA MTase.

Prokaryotic MTases function either as part of restriction modification systems or alone, participating in various cellular processes including antiviral defense, DNA replication and repair, cell cycle regulation, persister formation, and transcriptional modulation (12, 13). Moreover, an alteration in the level of DNA methylation can impact the virulence of bacteria such as *Salmonella enterica*, *Vibrio cholerae*, and *Clostridioides difficile* (14 – 16). It has been reported that deletion of the m4C MTase M2.HpyAII alters the expression of 102 genes, resulting in a decreased potential for *Helicobacter pylori* to induce inflammation and natural transformation (17).

Phase variation is a mechanism in which alternation between transcriptionally active (phase ON) and inactive (phase OFF) states occurs (18). Methylation of the promoter proximal GATC^prox^ site by Dam is required for transition to the phase ON state of pilin-related *pap* by specifically blocking PapI-dependent binding of leucine-responsive regulatory protein to promoter proximal sites in uropathogenic *Escherichia coli* (19). Phase variation controls the expression of *Salmonella* lipopolysaccharide modification genes by a DNA methylation-dependent mechanism (20). Modifications of the O-antigen that can affect the serotype include those carried out by the products of the glycosyltransferase (*gtr*) operon (21). The expression of the *gtr* genes is under the control of phase variation by epigenetic mechanisms requiring OxyR with Dam (20). A recent study demonstrated several undermethylated loci showing heterogeneous expression under different growth conditions, suggesting the relevance of the DNA adenine methylome as a source of phenotypic heterogeneity (22). Another study suggests that the GATC methylome provides chromosome structural support rather than regulatory support for *E. coli* survival during antibiotic stress and targeted bacterial DNA methylation as a viable approach to enhancing antibiotic activity (10).

The role and mechanism of DNA methylation in the oxidative stress response of bacteria remain elusive. Transcriptional profile of *E. coli* cells treated with hydrogen peroxide showed that SOS-response genes were induced (23), while *dam* mutants had elevated expression of SOS-related genes, such as *recA* and *uvrA* under both aerobic and low aerobic conditions (24). Yallaly and Eisenstark found that overexpression of Dam in *E. coli* renders bacteria more sensitive to hydrogen peroxide (25). Therefore, it seems that DNA methylation homeostasis is essential for bacterial oxidative stress resistance.

In this study, we first showed that Dam, but not the other four MTases tested, was essential for *S*. Typhimurium to survive after exposure to sublethal doses of hydrogen peroxide. We then used RNA-seq and single-molecule real-time (SMRT) sequencing to identify DNA methylation-regulated genes in response to oxidative stress in *S*. Typhimurium. We found that the overall level of m6A at GATC remained stable with H_2_O_2_ treatment; dynamic fluctuation of m6A at specific genomic sites is common. A set of 49 oxidative stress response genes showed a coupled change in m6A GATC and gene expression. The results may shed light on our understanding of bacterial GATC m6A-mediated gene expression regulation in environmental niches.

## RESULTS

### Dam is involved in *S*. Typhimurium response to hydrogen peroxide stress

**FIG 1:**
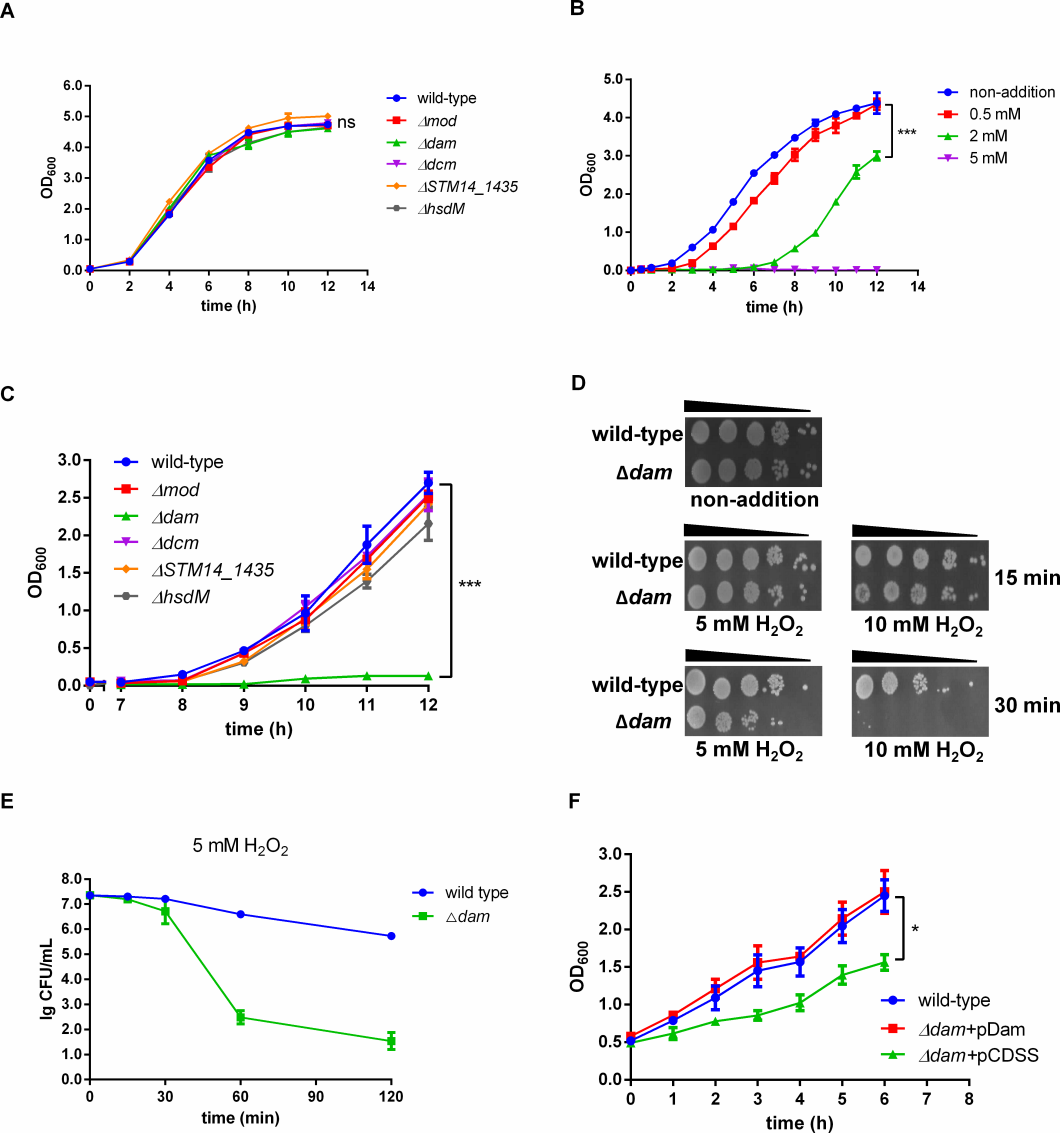
Dam is involved in *S.* Typhimurium resistance to hydrogen peroxide stress. (**A**) The growth curves of the DNA methylase mutants in LB medium. ns, not significant. (**B**) The growth curves of WT *S*. Typhimurium treated with series concentrations of H_2_O_2_ (0.5–5 mM). ***, *P* < 0.001. (**C**) The growth curves of the DNA methylase mutants with a 2-mM H_2_O_2_ treatment. ***, *P* < 0.001. (**D**) Spotted assays of the *dam* mutant at the indicated H_2_O_2_ concentrations and treatment duration. (**E**) Plated assays of the *dam* mutant at 5 mM H_2_O_2_ during 120 min of treatment. (**F**) The growth curve of the *dam* mutant under H_2_O_2_ stress was complemented by plasmid-expressing *dam*. *, *P* < 0.05.

Currently, at least five DNA methylases have been discovered in *S*. Typhimurium: Dam, Dcm, Mod, HsdM, and putative N4-methylcytosine methyltransferase STM14_1435 (26 – 29). To investigate the role of DNA methylation in *S*. Typhimurium experiencing hydrogen peroxide stress, we constructed these five DNA methylase gene knock-out strains individually. The growth of these deletion mutants was compared with that of the wild-type (WT) strain 14,028 s. These mutants exhibited similar growth rates in lysogeny broth (LB) medium as the WT strain (Fig. 1A). To determine the ideal concentration of hydrogen peroxide to induce stress, the WT strain was treated with a series of concentrations of H_2_O_2_ (from 0.5 to 5 mM), and growth curves were plotted. Compared with the mock-treatment control or 0.5 mM H_2_O_2_ treatment, the addition of 2 mM H_2_O_2_ caused an obvious delay in growth and a decline in maximum optical density during the stationary phase, while 5 mM H_2_O_2_ inhibited bacteria growth completely (Fig. 1B). Thus, strains were treated with 2 mM H_2_O_2_, and only Δ*dam* exhibited a growth defect (Fig. 1C). Similarly, the survival of Δ*dam* in different concentrations of H_2_O_2_ for the indicated treatment duration was determined by spot plating (Fig. 1D) and CFU counting assays (Fig. 1E). With the time increase, Δ*dam* showed impaired survival in both assays. The growth defect of Δ*dam* under H_2_O_2_ stress could be complemented by plasmid-expressing *dam* (Fig. 1F). Since Dam is responsible for catalyzing m6A modification at GATC motifs (8), we speculate that the essential role of Dam antagonism on oxidative stress in *S*. Typhimurium is exerted through manipulating the m6A level at the GATC motif to regulate the expression of stress response genes.

### Overview of transcriptome analysis under H_2_O_2_ stress

When bacteria encounter H_2_O_2_ stress, they must make a prompt response for survival and multiplication. To obtain an overall and comprehensive understanding of the transcriptome change in *S*. Typhimurium toward H_2_O_2_ stress, we used RNA-seq analysis to determine the effect of H_2_O_2_ at subinhibitory concentration on the gene transcriptional regulation of *S*. Typhimurium.

**FIG 2:**
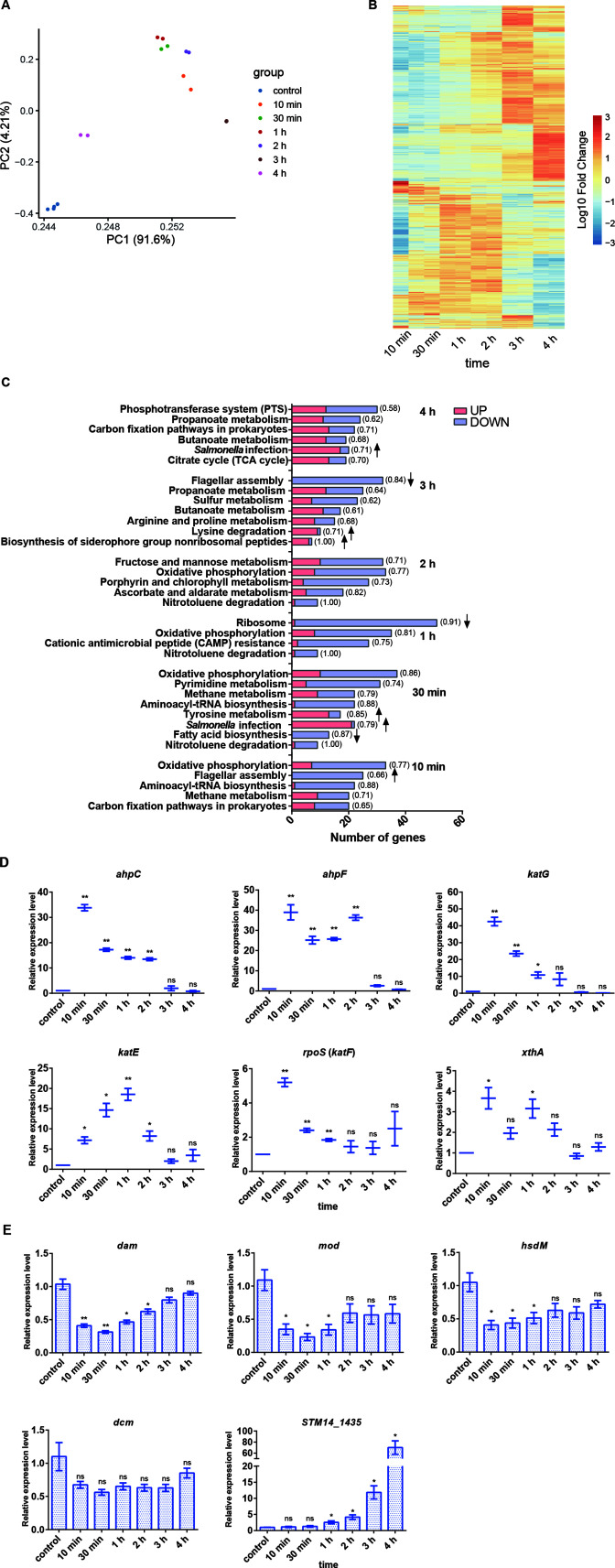
Overview of *S*. Typhimurium transcriptome under H_2_O_2_ stress. (**A**) The RNA-seq biological replicate samples were clustered by PCA, with circles with the same color indicating the same group. (**B**) Heat map cluster analysis of differentially expressed genes (DEGs) among the control and H_2_O_2_-treated groups. (**C**) Functional Kyoto Encyclopedia of Genes and Genomes enrichment analysis of the DEGs showed the most significantly enriched biological processes during H_2_O_2_ stress (10 min–4 h). (**D**) Transcriptional expression levels of *oxyR* regulon genes (including *ahpC*, *ahpF*, and *katG*), *rpoS* regulon genes (including *rpoS* and *katE*), and repair enzyme gene *xthA* during H_2_O_2_ stress adaption. *, *P* < 0.05; **, *P* < 0.01; ns, not significant. (**E**) Transcriptional expression levels of DNA methylase genes under H_2_O_2_ stress by quantitative real-time PCR. *, *P* < 0.05; **, *P* < 0.01; ns, not significant.

As an initial evaluation of the data set, we projected the transcriptomic data onto two dimensions using principal component analysis (PCA) (Fig. 2A). We found that the transcription phenotypes were clustered by treatment duration, indicating that there were considerable transcriptomic differences among these groups. Furthermore, data in the control group from two batches clustered together, illustrating that a minor batch effect exists between different groups. These indicated that the transcriptomic differences we observed were caused by H_2_O_2_ treatment, not batch effects.

As a result, 2,041, 2,748, 2,820, 2,597, 1,742, and 1,880 differentially expressed genes (DEGs) were identified in the H_2_O_2_-treated samples for 10 min, 30 min, 1 h, 2 h, 3 h, and 4 h, respectively (Fig. 2B; Data Set S1). Since the expression of about half of the genes in the *S*. Typhimurium genome is significantly altered, we conclude that oxidative challenge has a crucial impact on the *S*. Typhimurium transcriptome. To investigate the biological significance of DEGs, we performed Kyoto Encyclopedia of Genes and Genomes (KEGG) pathway analysis, and those with *P* < 0.05 were considered the most significantly enriched pathways. As shown in Fig. 2C, four most significantly upregulated biological processes were *Salmonella* infection, lysine degradation, biosynthesis of siderophore group non-ribosomal peptides, and tyrosine metabolism. The top three downregulated processes included flagellar assembly, fatty acid biosynthesis, and ribosome biogenesis.

We selected six oxidative stress genes, including *katG*, *katE*, *ahpC*, *ahpF*, *rpoS*, and *xthA* (30), for further verification. Our RNA-seq results showed that the fold change of *ahpC*, *ahpF*, *katG*, *katE*, *rpoS*, and *xthA* in the 10-min H_2_O_2_-treated group was 33.82, 38.59, 41.07, 5.24, 5.21, and 3.63, respectively (Data Set S1). Consistently, the expression levels of these genes were significantly upregulated about 3–40-fold in the 10-min H_2_O_2_-treated group and then gradually subsided to their initial levels by reverse transcription quantitative real-time PCR (RT-qPCR) (Fig. 2D), which confirmed the RNA-seq results.

Since *dam* or Dam-targeted genes are likely required for *Salmonella* survival under an oxidative stress response, we wonder whether the expression level of DNA methylase genes may change under oxidative stress. The transcriptional levels of *dam*, *dcm*, *mod*, *hsdM*, and *STM14_1435* under H_2_O_2_ stress were searched in RNA-seq data and validated by qPCR (Fig. 2E). The transcriptional levels of *dam*, *mod*, and *hsdM* were downregulated ≥2-fold after 10 min of H_2_O_2_ treatment and then gradually recovered to the basal level after 2–4 h. The transcriptional level of *dcm* did not change significantly with H_2_O_2_ treatment. In contrast, increased transcription of *STM14_1435* was much more significant after 1 h of treatment with H_2_O_2_ (Fig. 2E). Dam-modified GATC methylation was the focus of our subsequent studies based on the following considerations: (i) only *dam*, but not the other four MTase-encoding genes, is essential in anti-H_2_O_2_ defense; (ii) the function of STM14_1435, a putative N^4^-cytosine methyltransferase, is poorly characterized; (iii) Mod and HsdM (adenine methyltransferases) are more involved in restriction-modification systems, which mainly provide defense against phage DNA in bacteria.

### Overview of methylome analysis under H_2_O_2_ stress

To explore the DNA methylome of *S*. Typhimurium in the context of H_2_O_2_ stress, we extracted genomic DNA from *S*. Typhimurium grown in the absence or presence of H_2_O_2_ (2 mM) and analyzed for genome-wide methylation over time using SMRT sequencing. The reads were mapped to the *S*. Typhimurium genome reference sequence. Sequencing data with modification information, including position, modification quality value (QV), inter-pulse duration (IPD) value, fraction values, modification type, and modification context, i.e., the upstream and downstream sequences of a particular modification, were obtained for further analysis.

**FIG 3:**
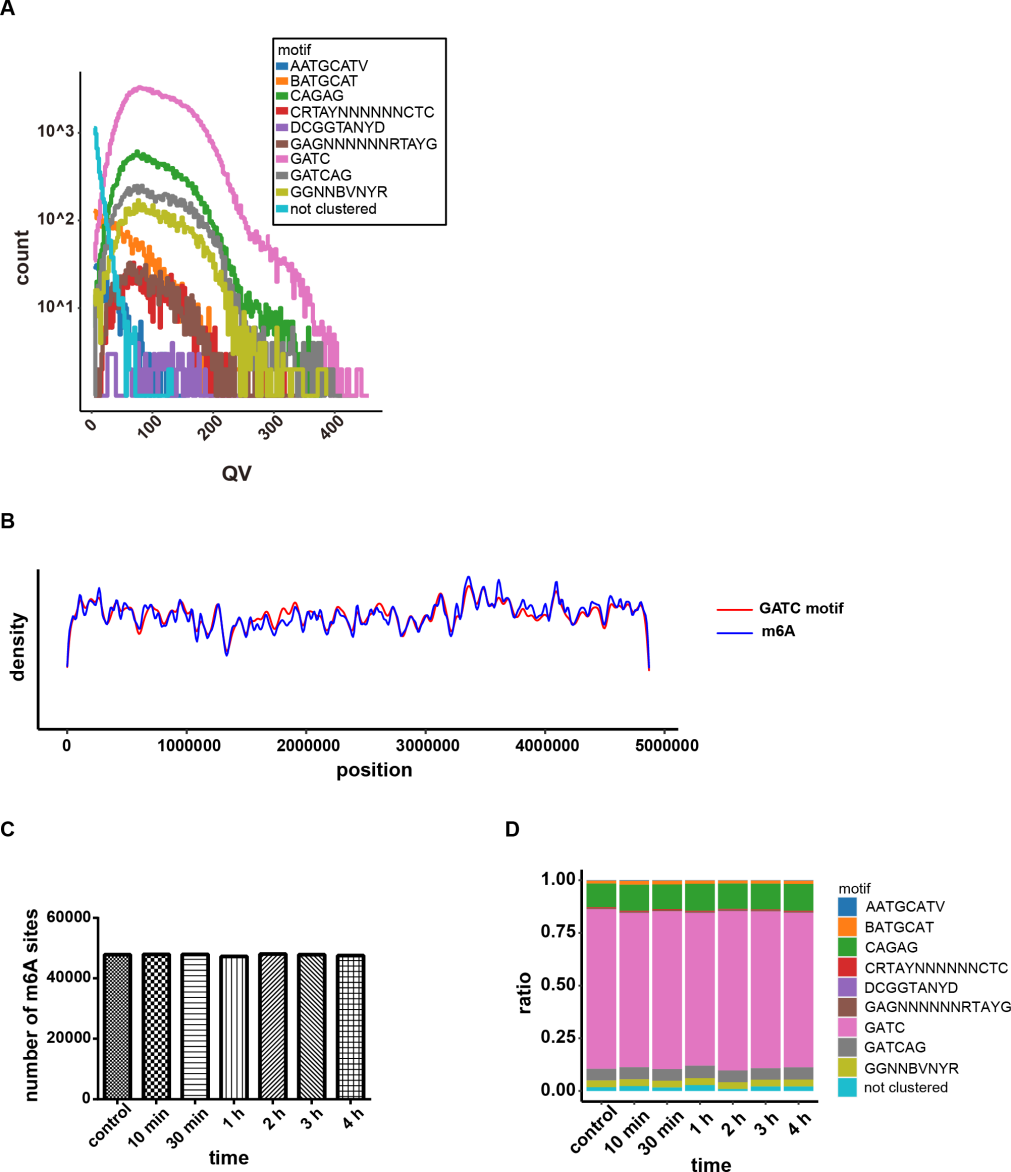
Overview of *S*. Typhimurium DNA methylome under H_2_O_2_ stress. (**A**) The methylation patterns and corresponding motifs detected in WT *S*. Typhimurium in the control and H_2_O_2_-treated groups. (**B**) Average density of GATC motifs and m6A sites along the chromosome of *S*. Typhimurium. (**C**) Calculation of the number of m6A sites among the control group and the indicated H_2_O_2_ treatment duration groups (10 min, 30 min, 1 h, 2 h, 3 h, 4 h) indicated that the m6A methylome was largely unaltered by H_2_O_2_ stress. (**D**) The ratio distribution of various methylated motifs among the control group and the indicated H_2_O_2_ treatment duration groups (10 min, 30 min, 1 h, 2 h, 3 h, 4 h).

The methylated motifs with QV >30 and corresponding methylation types in the control and H_2_O_2_-treated groups are shown, respectively, in Table 1. Compared with methylation of other sequences, m6A at GATC (catalyzed by Dam), CAGAG, and GATCAG motifs [catalyzed by unknown MTase(s)] had higher QVs (average 128.7, 120.9, and 130.9, respectively). m6A at GATC was the most prevalent across all methylated motifs (Fig. 3A). The average density distribution of the GATC motif was highly consistent with that of m6A along the chromosome of *S*. Typhimurium, though distribution divergence between m6A and the GATC motif exists in certain areas (Fig. 3B). The number of genome-wide m6A sites did not change much in all tested conditions (Fig. 3C). The proportion of motifs with m6A maintained stability under oxidative stress, although with small fluctuations (Fig. 3D). Oshima et al. found that GATC sequences are distributed unevenly in regulatory regions and the promoters of most of these Dam-controlled genes contain GATC sequences, suggesting that Dam-mediated methylation plays an important role in the global regulation of genes (24). It was also reported that many GATC sites were conserved in the two enteric bacteria, *S*. Typhimurium and *E. coli* (31). We calculated the numbers of GATC in the regulatory regions of *S*. Typhimurium and found that this motif was distributed unevenly. We found that about 50% of all *S*. Typhimurium genes contained more than two GATCs in the 500 bp upstream of the ORF start, with 763 genes containing three GATCs. The gene with the highest number of GATC sites was *STM14_4671* (*gidA*), which contains 17 GATCs, exhibiting a certain extent of evolutionary conservation with *E. coli* (24). Therefore, due to the abundance of methylation in regulatory regions, we propose that *S*. Typhimurium may dynamically alter specific m6A at GATC in regulatory regions to participate in oxidative stress adaptation.

**TABLE 1:** DNA base modifications in *S*. Typhimurium among the control and H_2_O_2_-treated groups^*a*^ ^,*b*^

Motifs	Modified position	Modification type	% of motifs detected	# of motifs detected	# of motifs in genome	Mean QV
**0** h						
GATC	2	m6A	99.8	38,413	38,458	151.9
CAGAG	4	m6A	99.6	5,645	5,663	145.1
GATCAG	5	m6A	99.9	2,899	2,900	159.0
BATGCAT	6	m6A	66.7	681	1,020	94.8
CRTAYNNNNNNCTC	4	m6A	100.0	235	235	146.1
CGGTAVYD	2	Unknown	34.7	1,519	4,376	47.0
GAGNNNNNNRTAYG	2	m6A	97.8	230	235	132.6
DVGGBVNHV	3	Unknown	4.4	4,415	100,086	42.4
GGNNBVNYR	1	Unknown	3.3	2,745	83,116	38.9
AATGCATV	6	m6A	37.1	98	264	64.6
**10** min						
GATC	2	m6A	99.8	38,418	38,458	143.6
CAGAG	4	m6A	99.8	5,654	5,663	135.4
GATCAG	5	m6A	99.9	2,899	2,900	146.6
BATGCAT	6	m6A	67.1	685	1,020	91.2
CRTAYNNNNNNCTC	4	m6A	100.0	235	235	132.7
GAGNNNNNNRTAYG	2	m6A	98.2	231	235	125.1
KMGGTAVYD	3	Unknown	32.1	840	2,612	47.9
GGNNBVNYR	1	Unknown	4.9	4,133	83,116	39.7
AATGCATV	6	m6A	40.1	106	264	62.9
CCAGGAAHD	2	m4C	23.0	122	529	43.8
**30** min						
GATC	2	m6A	99.8	38,419	38,458	135.6
CAGAG	4	m6A	99.6	5,646	5,663	129.2
GATCAG	5	m6A	99.9	2,899	2,900	139.9
BATGCAT	6	m6A	66.8	682	1,020	86.1
CRTAYNNNNNNCTC	4	m6A	100.0	235	235	128.6
GAGNNNNNNRTAYG	2	m6A	98.2	231	235	119.8
KMGGTAVYD	3	Unknown	32.0	838	2,612	47.2
AATGCATV	6	m6A	38.2	101	264	62.7
GVAGTAMCR	3	Unknown	25.8	112	433	43.1
CCAGGAAHD	2	m4C	20.0	106	529	41.3
**1** h						
GATC	2	m6A	99.9	38,421	38,458	129.1
CAGAG	4	m6A	99.7	5,649	5,663	117.1
GATCAG	5	m6A	99.8	2,895	2,900	126.8
BATGCATV	6	m6A	68.2	516	756	79.2
CRTAYNNNNNNCTC	4	m6A	100.0	235	235	116.3
GAGNNNNNNRTAYG	2	m6A	98.2	231	235	107.8
DSGGTAVYR	3	Unknown	28.1	922	3,281	45.0
GG	1	Unknown	1.3	7,932	601,021	39.3
**2** h						
GATC	2	m6A	99.6	38,315	38,458	97.6
CAGAG	4	m6A	98.9	5,604	5,663	92.7
GATCAG	5	m6A	99.5	2,889	2,900	100.2
BATGCATV	6	m6A	63.7	482	756	69.4
CRTAYNNNNNNCTC	4	m6A	100.0	235	235	90.5
GAGNNNNNNRTAYG	2	m6A	94.8	223	235	87.5
KMGGTAVYR	3	Unknown	26.3	546	2,076	43.2
BATGCATTH	6	m6A	34.5	75	217	53.9
**3** h						
GATC	2	m6A	99.4	38,232	38,458	92.6
CAGAG	4	m6A	99.3	5,624	5,663	88.0
GATCAG	5	m6A	99.6	2,891	2,900	92.9
CRTAYNNNNNNCTC	4	m6A	99.5	234	235	88.1
GAGNNNNNNRTAYG	2	m6A	95.7	225	235	83.3
BATGCATV	6	m6A	53.5	405	756	60.1
GMGGTAVYR	3	Unknown	23.4	304	1,296	41.6
BATGCATTH	6	m6A	23.9	52	217	50.3
**4** h						
GATC	2	m6A	99.9	38,424	38,458	150.3
CAGAG	4	m6A	99.7	5,650	5,663	138.9
GATCAG	5	m6A	99.9	2,899	2,900	151.2
BATGCATV	6	m6A	68.1	515	756	92.7
CRTAYNNNNNNCTC	4	m6A	100.0	235	235	137.1
GAGNNNNNNRTAYG	2	m6A	97.8	230	235	129.3
DVGGTAVYD	3	Unknown	24.2	1,231	5,074	47.5
GG	1	Unknown	1.1	7,117	601,021	40.3
BATGCATT	6	m6A	33.7	89	264	65.0
AATGCATVH	6	m6A	32.4	64	197	62.9
DCCAGGAMY	3	m4C	20.5	101	491	43.4

^*a*^ The proportion of motifs detected (% of motifs detected) was represented as the detected motifs number (# of motifs detected) divided by the motifs number in the genome (# of motifs in the genome).

^*b*^ N, A/T/C/G; R, A/G; Y, C/T; V, G/A/C; H, A/T/C; D, G/A/T; W, A/T; B, C/G/T; K, G/T.

### Combined analysis of the DNA methylome and transcriptome

**FIG 4:**
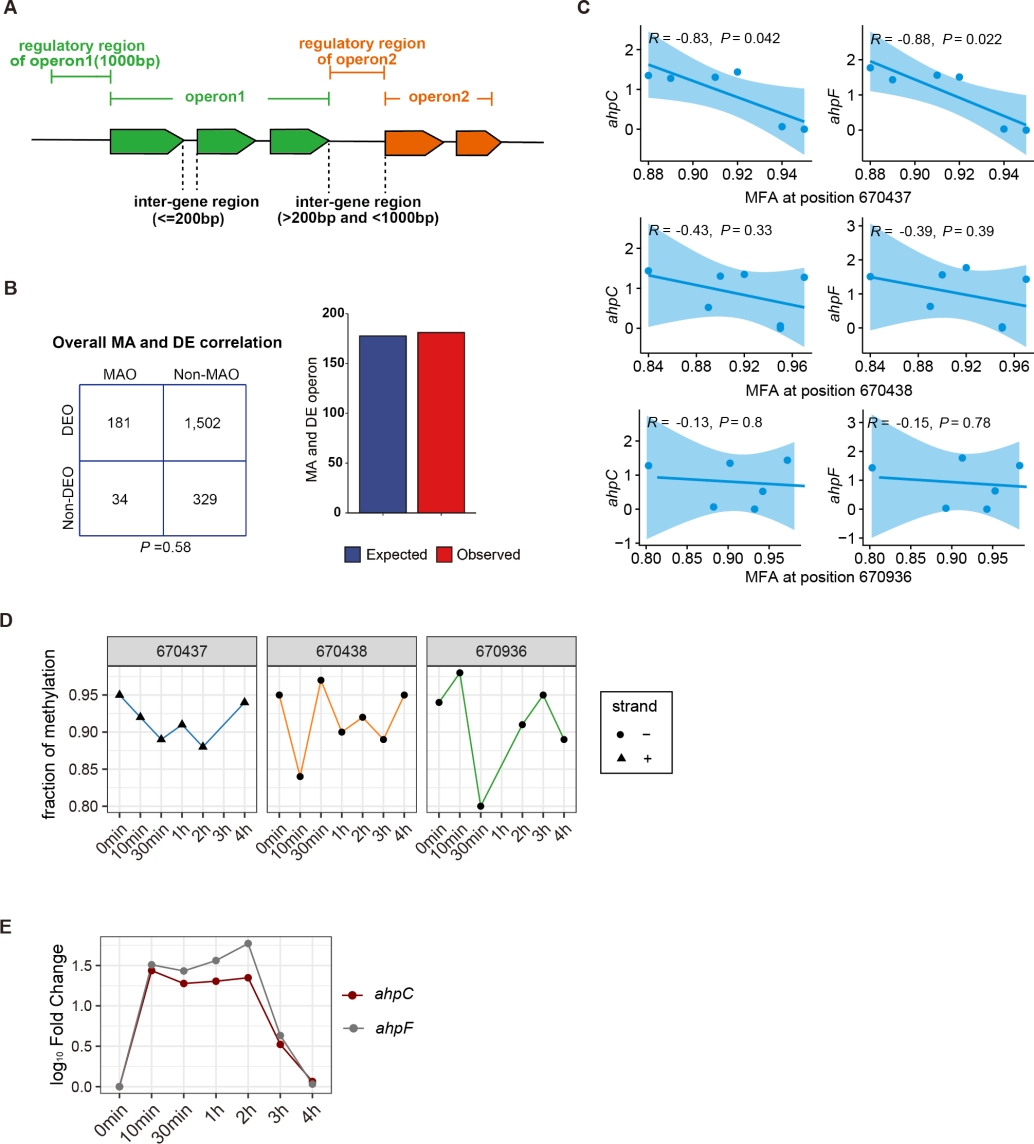
Combined analysis of the DNA methylome and transcriptome. (**A**) Definition of the inter-gene region and regulatory region. (**B**) Overall correlation between methylation-altered and differentially expressed operons by *χ* ^2^ test. Operons are stratified into methylation-altered operon (MAO), non-methylation-altered operon, differentially expressed operon (DEO), and non-differentially expressed operon. Expected values are calculated by multiplying the frequency of MAO by the frequency of DEO by the total number of operons in the analysis. (**C**) Pearson correlation coefficient *R* between *ahpC*, *ahpF* transcriptional expression change, and m6A fraction alteration in the *ahpCF* regulatory region. (**D**) Fraction of m6A methylation at GATC in the *ahpCF* regulatory region among the H_2_O_2_-treated groups and the control group. (**E**) Log_10_ fold change of *ahpCF* under H_2_O_2_ stress over time compared to the control group.

To identify the loci where transcription could be affected by m6A GATC in its regulatory region during oxidative stress, a combined analysis of the transcriptome and methylome was performed. We first linked m6A GATC sites in regulatory regions with operons, in the form of which bacterial genes were co-transcribed. A schematic diagram showing an operon and its upstream regulatory region is presented in Fig. 4A. Operons were defined by concatenate genes on the same strand with inter-gene regions less than 200 nt. The nearest region covering 1,000 nt upstream of the start codon of the first gene in a particular operon was considered the regulatory region. In cases where the inter-gene distances between two nearby operons were less than 1,000 nt, the entire region between the nearby genes was regarded as the regulatory region for the downstream gene. In this way, ~2,000 inter-gene regions were defined as regulatory regions.

Then, positions with qualified m6A at GATC in these regulatory regions were extracted to query modification fraction alterations (MFAs). Positions that had a proportional change of methylation greater than three times of the standard deviation were screened out. Simultaneously, gene expression levels in the respective operon were obtained by querying RNA-seq data. Results showed that m6A at GATC in 215 operons significantly changed during oxidative stress, which were defined as methylation-altered operons (MAOs). The remaining operons (1,831/2,046) are defined as non-MAO. Meanwhile, operons were stratified into differentially expressed operons (DEOs) and non-DEO. We did not observe a significant correlation between overall methylation pattern and transcriptional level (*χ* ^2^ test, *P* = 0.58) (Fig. 4B, left panel). Expected values are calculated by multiplying the frequency of MAO and the frequency of DEO by the total number of operons (Fig. 4B, right panel, blue bar), indicating the expected number of operons that methylation and transcription simultaneously altered; the red bar indicates the actually observed number of operons in this case in our data and analysis. The information about these 215 operons on m6A GATC profiles, fraction of methylation, log_10_ fold change over time, and Pearson correlation *R* of the respective operon are summarized in the Supplementary Figure.

Finally, we investigated the relationship between m6A at GATC and gene transcription alterations by Pearson correlation. Forty-nine genes located in 33 operons with high a correlation *R* coefficient (cutoff: |*R*| >0.6 and *P* < 0.05) are listed in Table 2 (including 19 genes with negative correlation in 18 operons and 30 genes with positive correlation in 15 operons). The methylation and expression patterns of these loci were as follows.

*ahpCF* was a representative operon showing a negative correlation between m6A abundance in its regulatory region and *ahpCF* transcriptional alterations under H_2_O_2_ stress. Pearson correlation *R* between *ahpC*, *ahpF* transcriptional change, and m6A MFA at position 670,437 (−706 bp upstream of the start codon) was −0.83 and −0.88, respectively, while neither position 670,438 (−705 bp upstream of the start codon) nor position 670,936 (−207 bp upstream of the start codon) showed a significant correlation with *ahpCF* transcriptional alterations (Fig. 4C). Specifically, the m6A modification pattern in the regulatory region and the transcriptional fold change of *ahpCF* during H_2_O_2_ stress are shown in Fig. 4D and E, respectively. The negative correlation value between m6A content and *ahpCF* transcriptional level changes suggested that the expression of *ahpCF* under oxidative stress could be inversely regulated by DNA methylation in its regulatory region.

**TABLE 2:** Genes filtered from the combined analysis of the transcriptome and DNA methylome with a high correlation *R* value

ID	Name	*R*	*P*	Description
*STM14_0567*	*ybaN*	0.90	0.016	Hypothetical protein STM14_0567
*STM14_0707*	***ahpC***	0.83	0.042	Alkyl hydroperoxide reductase subunit C
*STM14_0708*	***ahpF***	0.88	0.022	Alkyl hydroperoxide reductase F52a subunit
*STM14_0817*	*potE*	0.84	0.038	Putrescine transporter
*STM14_0961*		0.82	0.043	Hypothetical protein STM14_0961
*STM14_1135*		0.82	0.045	Hypothetical protein STM14_1135
*STM14_1267*	*scsB*	0.99	0.0063	Suppression of copper-sensitivity protein
*STM14_1725*	*ydhC*	0.96	0.002	Inner membrane transport protein YdhC
*STM14_1798*	*ynfL*	0.90	0.04	Putative transcriptional regulator
*STM14_1898*	*nmpC*	0.99	0.014	Putative outer membrane porin
*STM14_1900*	*smvA*	0.99	0.01	Methyl viologen resistance
*STM14_1904*		0.96	0.0095	Hypothetical protein STM14_1904
*STM14_2065*		0.83	0.04	Hypothetical protein STM14_2065
*STM14_2272*		0.98	0.00066	Phage-tail assembly-like protein
*STM14_2807*		0.77	0.044	Putative dehydratase
*STM14_3294*	***smpB***	0.89	0.007	SsrA-binding protein
*STM14_3338*	*fljB*	0.78	0.038	Flagellin
*STM14_4269*		0.83	0.022	Hypothetical protein STM14_4269
*STM14_5330*	*cybC*	0.94	0.0049	Cytochrome b562
*STM14_0076*		0.88	0.024	Hypothetical protein STM14_0076
*STM14_0177*		0.76	0.046	Putative cytoplasmic protein
*STM14_0178*		0.84	0.017	Na^+^/galactoside symporter
*STM14_0180*	*aroP*	0.84	0.017	Aromatic amino acid transporter
*STM14_1053*		0.90	0.014	Hypothetical protein STM14_1053
*STM14_1388*	***ycfR***	0.68	0.05	Putative outer membrane protein
*STM14_2212*	*manX*	0.82	0.024	Mannose-specific enzyme IIAB
*STM14_2213*	*manY*	0.83	0.022	Mannose-specific enzyme IIC
*STM14_2492*		0.93	0.0021	Hypothetical protein STM14_2492
***STM14_2773***		0.79	0.033	Putative outer membrane protein
*STM14_3625*		0.77	0.045	Hypothetical protein STM14_3625
*STM14_3723*		0.86	0.014	Putative mannitol dehydrogenase
*STM14_3843*	*ygiK*	0.76	0.047	Putative transporter
*STM14_4271*	*yhhY*	0.78	0.039	Putative acetyltransferase
*STM14_4426*	*yiaL*	0.87	0.0012	Putative cytoplasmic protein
*STM14_4427*		0.93	0.0023	Putative chemotaxis protein
*STM14_4428*	*yiaM*	0.80	0.029	2,3-Diketo-L-gulonate TRAP transporter small permease
*STM14_4429*	*yiaN*	0.89	0.0078	Hypothetical protein STM14_4429
*STM14_4430*	*yiaO*	0.76	0.048	Putative periplasmic dicarboxylate-binding protein
*STM14_4431*	*lyxK*	0.88	0.0084	L-xylulose kinase
*STM14_4432*	*sgbH*	0.80	0.032	3-Keto-L-gulonate-6-phosphate decarboxylase
*STM14_4433*	*sgbU*	0.91	0.0046	Putative L-xylulose 5-phosphate 3-epimerase
*STM14_4434*	*sgbE*	0.83	0.02	L-ribulose-5-phosphate 4-epimerase
*STM14_4442*	*yibF*	0.76	0.049	Glutathione S-transferase
*STM14_5162*		0.86	0.03	Hypothetical protein STM14_5162
*STM14_5320*		0.79	0.035	Hypothetical protein STM14_5320
*STM14_5324*		0.79	0.034	Myo-inositol 2-dehydrogenase
*STM14_5325*		0.85	0.015	Putative permease
*STM14_5326*		0.80	0.03	Putative cytoplasmic protein
*STM14_5327*		0.78	0.038	Putative endonuclease

^*a*^ The bold are representative of our results.

**FIG 5:**
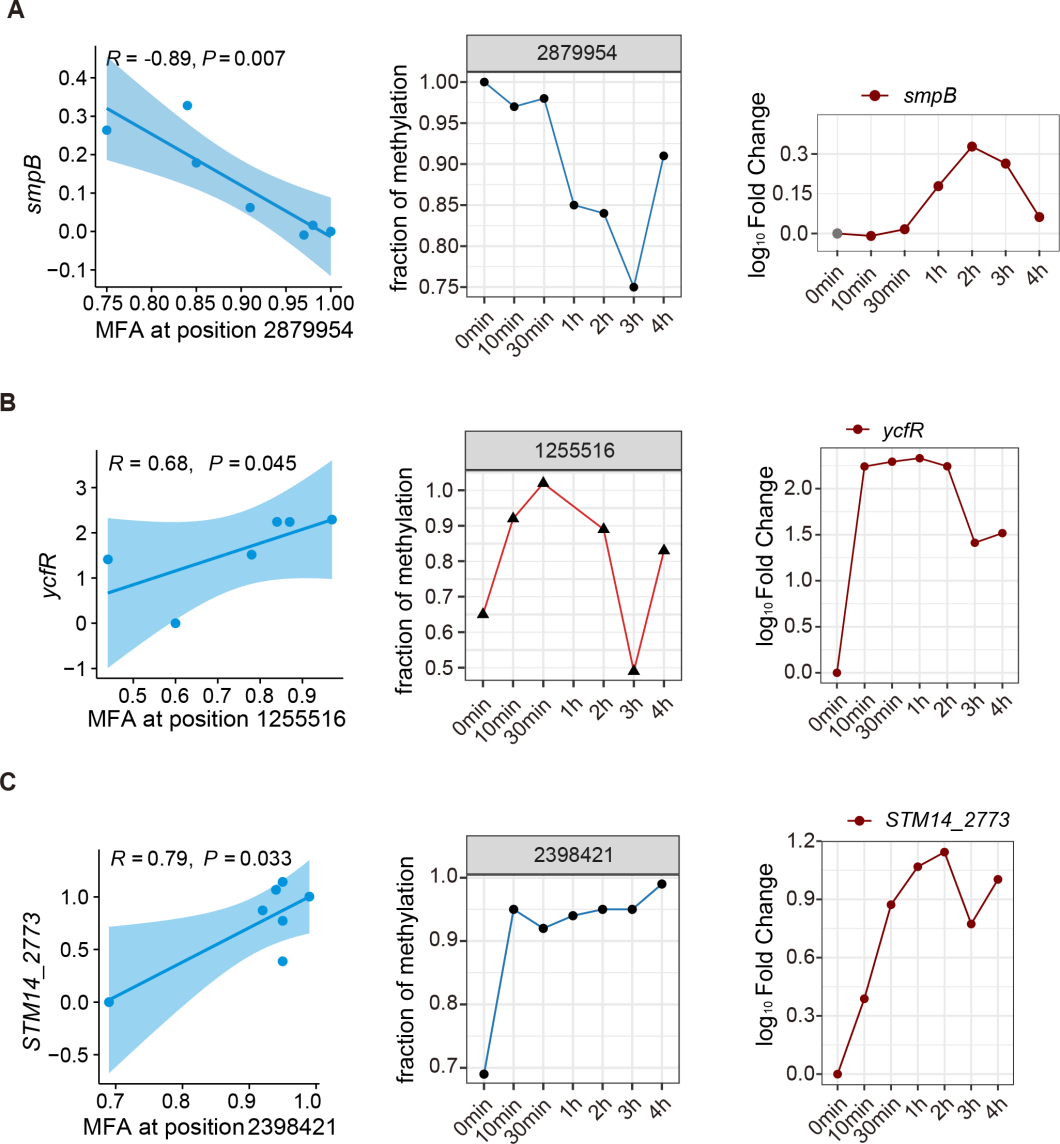
The correlation analysis of DNA methylation and transcription in the translational quality control system and outer membrane genes. Pearson correlation coefficient *R* of *smpB* (A, left), *ycfR* (B, left), and *STM14_2773* (C, left) transcriptional expression change and m6A fraction alteration in *smpB*, *ycfR*, and *STM14_2773* regulatory regions, respectively; fraction of m6A methylation at GATC in *smpB* (A, middle), *ycfR* (B, middle), and *STM14_2773* (C, middle) regulatory regions among the H_2_O_2_-treated groups and the control group; log_10_ fold change of *smpB* (A, right), *ycfR* (B, right), and *STM14_2773* (C, right) under H_2_O_2_ stress over time compared to the control group. 54. Supplementary Figure. m6A at GATC in 215 operons significantly changed during oxidative stress. The information about these 215 operons, regarding m6A GATC profiles, fraction of methylation, log_10_ fold change over time, and Pearson correlation *R*, was presented, respectively.

Under oxidative stress, another operon showing a negative correlation between gene transcriptional level and m6A content was *smpB* (Small protein B) (Fig. 5A). Compared with the control group, the m6A level at position 2,879,954 in the *smpB* regulatory region decreased dynamically until 3 h after H_2_O_2_ treatment, which was in contrast to the *smpB* transcriptional level. SmpB is a component of the translational quality control system. The SmpB-SsrA quality control surveillance system rescues ribosomes stalled on incomplete or damaged mRNAs and directs aberrant protein product degradation (trans-translation) (32). Our RNA-seq data showed that oxidative stress had a strong impact on ribosome functions. Transcription of about 91% of ribosome-related genes changed under oxidative stress, and nearly all were downregulated. Thus, the induction of *smpB* facilitates the trans-translation process.

The outer membrane of Gram-negative bacteria provides protection against toxic molecules, including ROS. Several operons showed a positive correlation between regulatory region methylation and gene transcription. For example, the correlation *R* between *ycfR* (putative outer membrane protein) transcription and MFA at position 1,255,516 was 0.68 (*P* = 0.045) (Fig. 5B). YcfR is involved in biofilm formation (33), and when treated with 6% electrolyzed oxidizing water, expression of *ycfR* was upregulated (34). Outer membrane protein STM14_2773 was also involved (Fig. 5C), indicating that *S*. Typhimurium may induce transcription of outer membrane protein-coding genes by regulating m6A at GATC to maintain membrane integrity under oxidative stress.

## DISCUSSION

In this study, we constructed a series of DNA methylase gene knock-out strains and treated these mutants with subinhibitory concentrations of H_2_O_2_ *in vitro*, mimicking the oxidative stress bacteria confronted in host cells. We identified *dam* as an essential oxidative stress gene. In fact, *dam* plays pleiotropic roles in the pathogenesis of enteric bacteria (35) and the methyl-directed mismatch repair system under oxidative stress (36). It was reported that the minimal inhibitory concentration of H_2_O_2_ toward *dam* mutant was lower than that toward WT *S*. Typhimurium (37). A recent study identified 80 genes, including *dam* involved in oxidative stress resistance, through a combined analysis of transposon-seq and targeted proteomics (38). Using pCDSS-*dam* (lower copies of the plasmid providing chromosomal level complementation), growth defects of the *dam* mutant in oxidative stress were recovered, implying that *dam* is important for *S*. Typhimurium oxidative stress adaptation. We asked whether more *dam* transcripts were beneficial for *S*. Typhimurium to cope with oxidative stress. WT *S*. Typhimurium cells were exposed to H_2_O_2_ in a 4-h adaptive period (39). After qPCR validation of RNA-seq data, we found that *dam* showed a trend of decreasing and then recovering, and there was no induced transcription of *dam* after hydrogen peroxide treatment. The result indicated that overexpression of Dam did not confer a survival advantage for *S*. Typhimurium in oxidative stress. Consistent with this, it is supposed that moderate amounts of Dam may confer bacteria with a certain extent of damage repair capacity (25). Dam levels must be tightly controlled since both DNA replication and mismatch repair need to be precisely timed, both of which are dependent on Dam. The delicate balance between the amount of Dam and the level of DNA per cell is critical for cell survival.

Our DNA methylation profiles by SMRT showed that the total number of m6A did not change significantly at different time points after H_2_O_2_ treatment, and the amount of m6A at GATC was stable under oxidative stress. Cohen et al. found the remarkable stability of the GATC methylome during antibiotic exposure and supposed that m6A at GATC may provide an important backbone structure for *E. coli* under antibiotic stress (10). They reasoned that the GATC methylome provided structural rather than regulatory support for bacterial survival during antibiotic stress, although they cannot exclude the additional involvement of transcriptional dysregulation, enabling the DNA repair process to function in the context of antibiotic stress. Bourgeois et al. found that the methylome is remarkably static; >95% of adenosine bases retain their methylation status across conditions (40), suggesting that DNA methylation is an essential factor for bacterial response to environmental stresses. Furthermore, ∆*dam* bacteria developed significantly more DNA damage than WT cells during H_2_O_2_ treatment (10), and overexpressing Dam in *E. coli* increased bacterial sensitivity to H_2_O_2_. These findings, combined with our data, support the idea that homeostasis of m6A at GATC is essential for bacterial resistance to oxidative stress.

Also, we found that there are newly discovered motifs for which Mtases are still unknown (Table 1). Several DNA methyltransferases have compatibility with substrate specificity and thus recognize multiple motifs. On the other hand, identification of new DNA methyltransferases faces challenges such as DNA methylation sequence motif confirmation by liquid chromatography coupled to mass spectrometry analysis and verification of putative Mtase activity and specificity (41).

There is some controversy regarding the role of *dam* in regulating gene expression, either due to the technique/approaches used or the sensitivity of the experiments (22, 24). Differences in expression were detected in 10 loci harboring undermethylated GATC sites in *Salmonella* with different methylation backgrounds (WT/Δ*dam*/Dam ++*+Salmonella*) by single cell analysis (22). Among them, a larger ON subpopulation was detected at loci *holA* in Δ*dam*, suggesting Dam-dependent repression; Dam methylase overproduction abolished the formation of the Nan^ON^ subpopulation (22). However, most of the 10 loci did not show significant transcriptional differences in Δ*dam* through DNA microarray analysis, except that *holA* was upregulated and *nanA* was downregulated in the *dam* mutant compared to WT (24). It seems that a WT genetic background would be more authentic and suitable to investigate the role of Dam-catalyzed m6A during oxidative stress. Thus, we treated WT *Salmonella* with hydrogen peroxide to investigate gene expression regulation by m6A at GATC. Due to the potential role of m6A at GATC in regulatory regions, we propose that transcription of antioxidative genes could be regulated by m6A at GATC in regulatory regions. Through a combined analysis of the transcriptome and DNA methylome, we found that *ahpCF*, encoding an alkyl hydroperoxide reductase to decompose hydrogen peroxide, was likely to be regulated at m6A GATC in its regulatory region. Flagellin-encoding gene *STM14_3338* (*flgB*) was among the genes filtered from the combined analysis with significantly strong correlation (*R* = −0.78, *P* = 0.038, Table 2), indicating that the motility of bacteria may be m6A-regulated under oxidative stress.

In eukaryotes, stress could elicit epigenetic modifications of histones and DNA that support long-lasting downstream responses (42, 43). Several studies have demonstrated that DNA methylation plays an important role in regulating gene expression, especially in stress-mediated signaling pathways (44, 45). A study found that the expression of *BMP4*, a member of the bone morphogenetic protein family, and its methylation at CpG sites −199 (*BMP4^−199^*) and −123 (*BMP4^−123^*), both lying in CpG islands, were inversely correlated in eight sensitive and resistant cell lines. *BMP4^−199^* and *BMP4^−123^* methylation were significantly correlated to each other, while *BMP4^−199^* methylation exhibited a stronger correlation with *BMP4* gene expression than the *BMP4^−123^* site. *BMP4^−199^* and *BMP4* ^−123^ methylation were inversely correlated with cisplatin resistance in the gastric cancer lines (*P* < 0.05 for both sites). *BMP4* expression levels were also positively correlated with cisplatin resistance.

In conclusion, we found that DNA methylation homeostasis at the genome-wide level and variability in specific regions related to gene transcription were essential for *Salmonella* to respond to oxidative stress. These findings will provide new insights into bacterial epigenetic regulatory mechanisms of gene expression in the host microenvironment.

## MATERIALS AND METHODS

### Bacterial strain, plasmid, oligodeoxynucleotides, and growth conditions

The bacterial strain and plasmid used in this study are listed in Table S1. The strains of *Salmonella* used in this study belong to serovar Typhimurium and are derived from strain ATCC 14,028S (a link to the genome sequence, https://www.ncbi.nlm.nih.gov/nuccore/CP001363.1). For simplicity, *Salmonella enterica* serovar Typhimurium is named *S*. Typhimurium throughout the text. The DNA oligonucleotides used in this study are listed in Table S2. The knock-out strains were constructed by integration into the chromosome of *S*. Typhimurium using the lambda Red recombination system (46). Bacteria were grown at 37°C in liquid LB on nutrient agar plates containing 1.5% (wt/vol) agar and supplemented with antibiotics as required. The final concentrations of antibiotics used were 100 µg/mL of ampicillin, 17 µg/mL of chloramphenicol, and 50 µg/mL of spectinomycin. *Escherichia coli* DH5α was used as the host for plasmid DNA preparation. To induce complementary plasmid expression, arabinose was added at a final concentration of 10 mM.

### Bacteria resistance to H_2_O_2_

To depict the growth curves of WT *S*. Typhimurium and its derived mutants, overnight cultures of bacteria were inoculated into fresh LB broth media to give a seeding concentration corresponding to OD_600_ = 0.01 and then cultured at 37°C with 250 rpm shaking. To determine the appropriate H_2_O_2_ concentration toward WT *S*. Typhimurium growth curves for the following phenotypic screening, WT *S*. Typhimurium cells were cultured in LB medium overnight and then 1:100 inoculated into fresh LB medium, which contained freshly prepared H_2_O_2_, to give final concentrations ranging from 0.5 to 5 mM, and OD_600_ was measured at regular intervals as indicated. For determining bacteria survival after H_2_O_2_ exposure, log-phase cultures were diluted to OD_600_ = 0.2 with phosphate buffered saline, exposed to 5 or 10 mM H_2_O_2_, and incubated by rolling at 37°C. Then, aliquots were removed from each group at the indicated time points, serially 10-fold diluted, spotted (2 µL of dilutions), or plated (100 µL of dilutions) on LB agar plates as previously described (47). To determine the subinhibitory concentration of H_2_O_2_, 100 µL of bacteria cultures (OD_600_ = 0.01) was mixed with different concentrations of H_2_O_2_ in a 96-well plate, and the OD was measured after 14 h of incubation at 37°C. For plasmid complementation, WT and mutants with empty vector (pCDSS) or plasmid-expressing *dam* (pCDSS-*dam*) were grown to early-log phase supplemented with arabinose. H_2_O_2_ at a final concentration of 2 mM was then added into the cultures for further OD measurements.

### RNA isolation for RNA-seq and cDNA synthesis for quantitative RT-PCR

Stationary-phase WT *S*. Typhimurium cultures were inoculated by 1:1,000 in 25 mL LB medium in 250 mL baffled flasks and grown at 37°C with 200 rpm shaking until OD reached 0.5. WT cultures were then treated with subinhibitory concentrations of H_2_O_2_ for different periods (10 min, 30 min, 1 h, 2 h, 3 h, 4 h). The control group received no H_2_O_2_ treatment and was collected at 0 min. RNA was isolated with the RiboPure-Bacteria kit (Ambion), and genomic DNA was digested with DNase I as previously described (48). For RNA-seq, RNA samples were fragmented by heating at 95°C for 10 min and annealed to biotinylated random primers. A 5′ adaptor, which contained an Illumina primer, was added to the sequence. First-strand cDNA was synthesized by RT. Then, Illumina primers were used to obtain double-stranded cDNA by the PCR method. cDNA fragments of 300 to 500 bp were harvested by gel extraction and then directly amplified with a TruSeq PE cluster kit (Illumina, USA). Sequencing reactions were performed on an Illumina HiSeq 2000 sequencer.

For qRT-PCR, cDNA was synthesized using the SuperScript III First Strand kit (Invitrogen) with random hexamers. Target gene transcript levels were measured by quantitative real-time PCR with SYBR Green as the label and normalized to *16S rRNA* transcript levels by the 7500 Fast Real-Time PCR System. The relative transcriptional level was determined by the method of 2^−ΔΔCt^ (49). Primers for qPCR are listed in Table S2. Samples were run in triplicate.

### Genomic DNA extraction for PacBio sequencing

Bacteria were collected at the same time as RNA-seq samples were prepared and from the same WT cultures grown in the presence or absence of H_2_O_2_. Genomic DNA was extracted using the GenElute Bacterial Genomic DNA Extraction Kit (Sigma). The concentrations of genomic DNA were measured using Nanodrop 2000 (Thermo Scientific). Samples were prepared in three repetitions according to a guide for SMRT (single molecule real-time, PacBio) bell template preparation and then sent for SMRT bell library preparation, followed by sequencing using a PacBio RS II instrument as previously described (50).

### Raw data processing

For RNA-seq raw data, to obtain high-quality reads that could be used for later analysis, raw reads need to be filtered. Trimmomatic software was first used for quality control and linker removal (51). Rockhopper2 (52) was used to align clean reads to the reference genome of *S*. Typhimurium. Rockhopper2 (53) was also used to obtain the number of reads aligned to the gene in each sample and to calculate RPKM (reads per kilobase per million mapped reads) to compare transcript expression between different samples. Using DESeq to standardize counts, with an adjusted *P*-value <0.05 and a difference of multiples greater than two as filtering criteria, genes were considered to have undergone transcriptional changes during H_2_O_2_ stress. KEGG enrichment analysis of the DEGs was performed to determine biological functions or pathways that are mainly affected by DEGs. For SMRT raw data, h5 files from SMRT sequencing were processed with SMRT link v7.0.1 provided by Pacific Biosciences to build data sets by sample. Genome-wide detection of epigenomic modifications was performed using the standard pipeline embedded in the SMRT link toolkit with default parameters. After SMRT link analysis, a CSV file with modification information including position, modification QV, IPD value, modification type, and modification context, i.e., upstream and downstream sequence of a particular modification, could be obtained for further analysis.

### Combined analysis of DNA methylome and transcriptome

DNA methylation modification data were imported into R, and further analysis was performed with in-house R scripts. Due to Dam-methylated GATC motifs, modifications annotated with m6A on GATC motifs that met the QV threshold (30) were preserved for downstream analysis. Here, a default QV (defined as an estimate for the accuracy of basecalls during sequencing) of 30 was used, corresponding to 99.9% accuracy. As gene expression in bacteria is often regulated in the form of an operon, certain modifications may regulate the expression of several successive downstream genes. Operons are defined by concatenate genes on the same strand with inter-gene regions less than 200 nt. In addition, regulatory regions, i.e., inter-gene regions upstream of the start codon of the first gene of a particular operon, were defined. As a modification far away from certain operons is not likely to regulate expression directly, inter-gene regions longer than 1,000 nt were trimmed, and only the nearest 1,000 nt were preserved to define regulatory regions. In this way, ~2,000 inter-gene regions were defined as regulatory regions, and positions with qualified modifications in these regions were extracted to query MFAs. To focus on positions that had undergone dramatic alteration in methylation, we filtered the results to obtain positions that had a proportional change in methylation greater than three times the standard deviation. Genes in the respective operon were obtained for querying RNA-seq data simultaneously. Pearson correlation analysis was performed to find a possible relationship between m6A GATC and gene expression alterations.

### Statistical analysis

Descriptive and statistical comparisons were performed using GraphPad Prism version 5.0 (GraphPad Software, San Diego, CA, USA). A statistical comparison of mean values between two specific groups was carried out using the Student’s *t*-test. ANOVA analysis was performed to compare the mean values of more than two groups. *, *P* < 0.05; **, *P* < 0.01; ***, *P* < 0.001; ns, not significant.

## Data Availability

All of the PacBio sequencing and RNA-seq data sets generated in this study have been submitted to the NCBI BioProject database under accession number PRJNA1007249.

## SUPPLEMENTAL MATERIAL

Data Set S1 spectrum.02479-23-s0001.xlsx Differentially expressed genes (DEGs) were identified in the H_2_O_2_-treated samples for 10 min, 30 min, 1 h, 2 h, 3 h, and 4 h.

Fig. S1 spectrum.02479-23-s0002.pdf m6A at GATC in 215 operons significantly changed during oxidative stress.

Table S1 spectrum.02479-23-s0003.pdf Bacterial strains and plasmids used in this study

Table S2 spectrum.02479-23-s0004.pdf DNA oligonucleotides used in this study

ASM does not own the copyrights to Supplemental Material that may be linked to, or accessed through, an article. The authors have granted ASM a non-exclusive, world-wide license to publish the Supplemental Material files. Please contact the corresponding author directly for reuse.
