# Supplementary material for: Integrative DNA methylome and transcriptome analysis reveals DNA adenine methylation is involved in Salmonella enterica Typhimurium response to oxidative stress
Source: Microbiol Spectr. 2023 Oct 26;11(6):e02479-23. doi: 10.1128/spectrum.02479-23 (PMC10715015; doi:10.1128/spectrum.02479-23)
Supplement: Fig. S1 — m6A at GATC in 215 operons significantly changed during oxidative stress. [file spectrum.02479-23-s0002.pdf]

Supplementary Figure. m6A at GATC in 215 operons significantly changed during oxidative stress. The information of these 215 operons, regarding m6A GATC profiles, fraction of methylation, $\log_{10}$ fold change over time and Pearson correlation *R* was presented respectively.

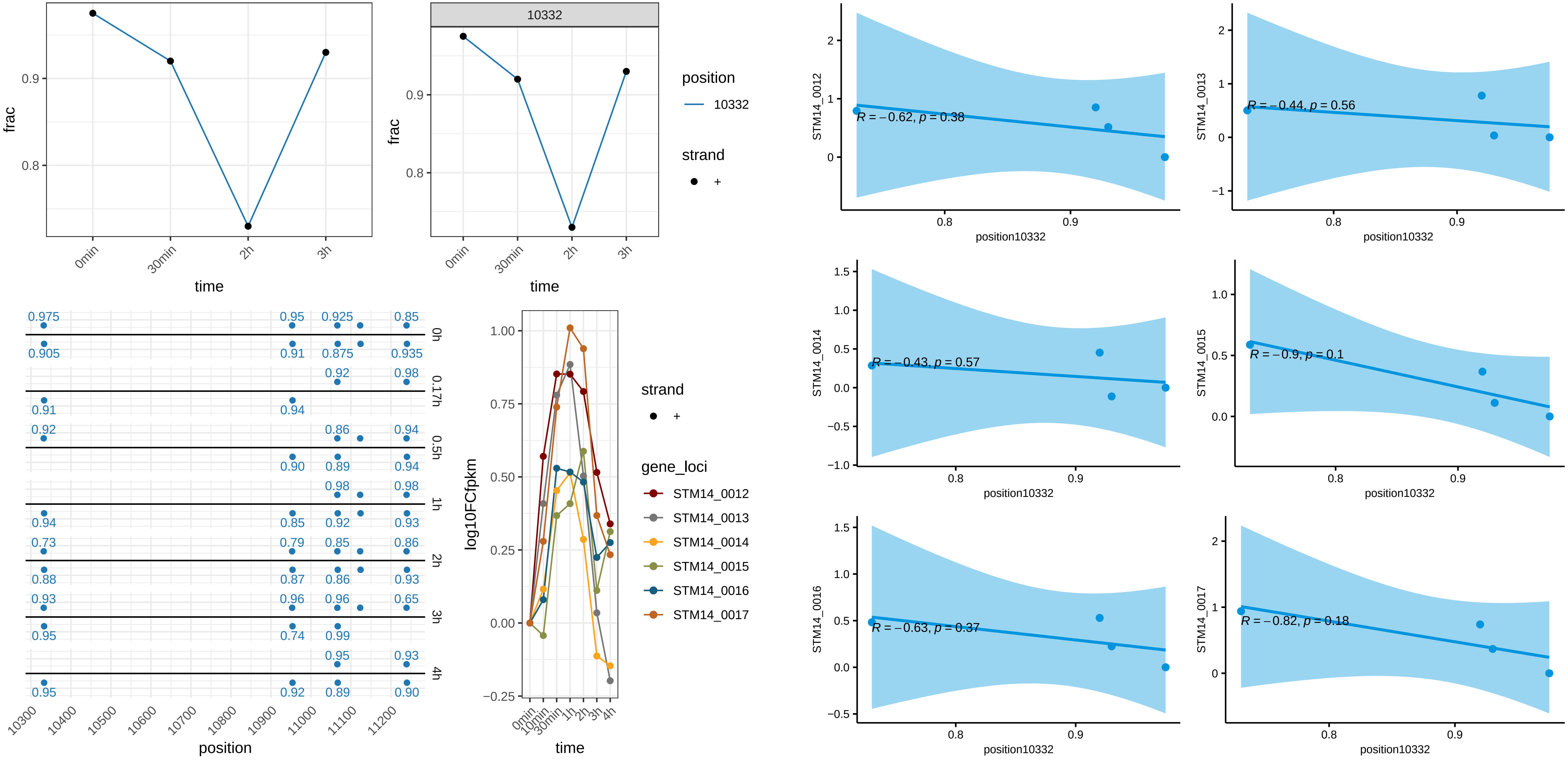

frac
time
0min
30min
2h
3h
10332
position
strand
+
STM14_0012
STM14_0013
STM14_0014
STM14_0015
STM14_0016
STM14_0017
R = −0.62, p = 0.38
R = −0.44, p = 0.56
R = −0.43, p = 0.57
R = −0.9, p = 0.1
R = −0.63, p = 0.37
R = −0.82, p = 0.18
position10332
log10FCfpkm
gene_loci
0h
0.17h
0.5h
1h
2h
3h
4h
10min
1h

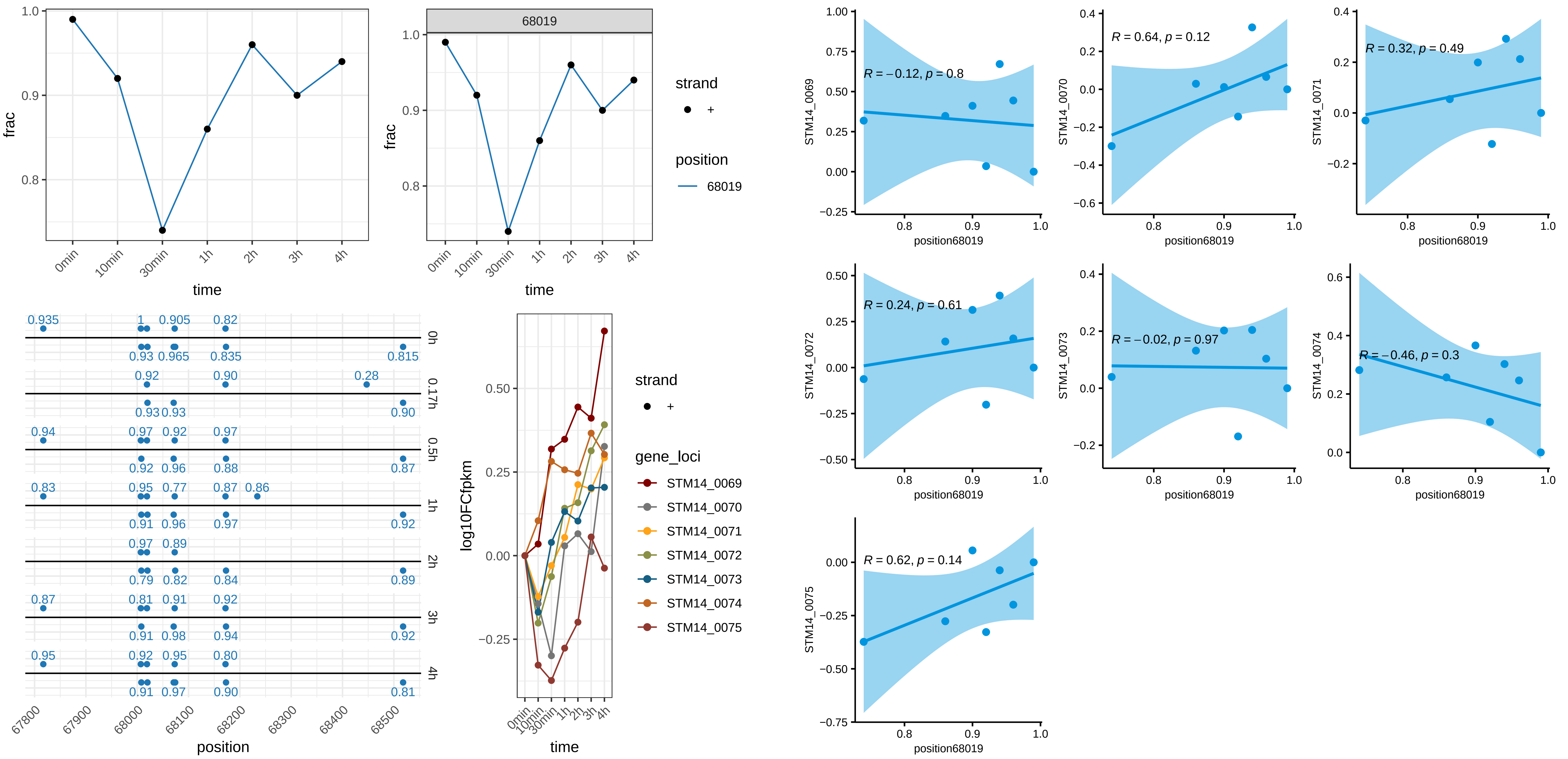

frac
time
0min
10min
30min
1h
2h
3h
4h
68019
strand
+
position
68019
position
67800
67900
68000
68100
68200
68300
68400
68500
0h
0.17h
0.5h
1h
2h
3h
4h
log10FCfpkm
gene_loci
STM14_0069
STM14_0070
STM14_0071
STM14_0072
STM14_0073
STM14_0074
STM14_0075
position68019
R = −0.12, p = 0.8
R = 0.64, p = 0.12
R = 0.32, p = 0.49
R = 0.24, p = 0.61
R = −0.02, p = 0.97
R = −0.46, p = 0.3
R = 0.62, p = 0.14

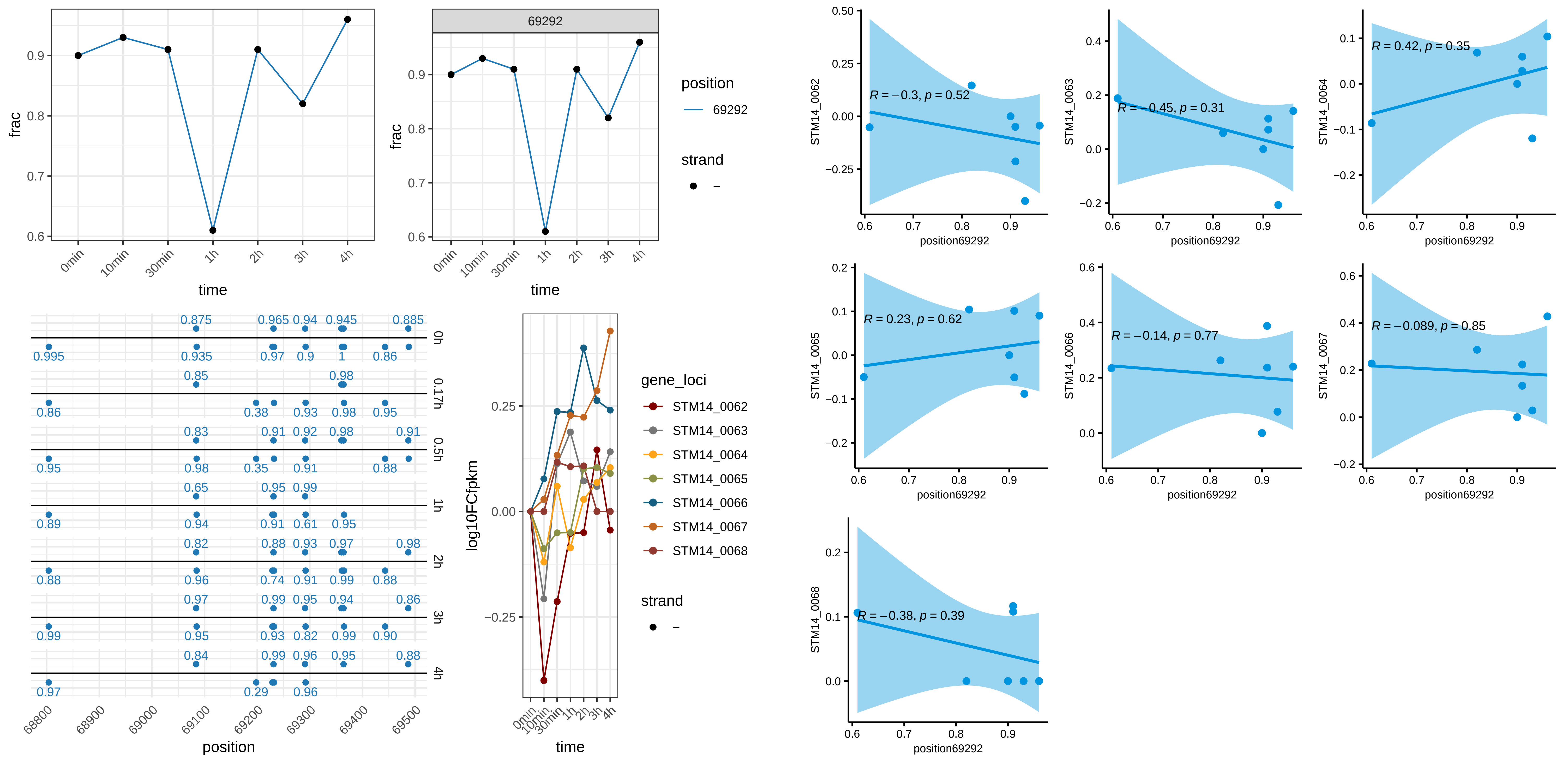

frac
time
69292
position
strand
gene_loci
STM14_0062
STM14_0063
STM14_0064
STM14_0065
STM14_0066
STM14_0067
STM14_0068
log10FCfpkm
R = −0.3, p = 0.52
R = −0.45, p = 0.31
R = 0.42, p = 0.35
R = 0.23, p = 0.62
R = −0.14, p = 0.77
R = −0.089, p = 0.85
R = −0.38, p = 0.39
position69292

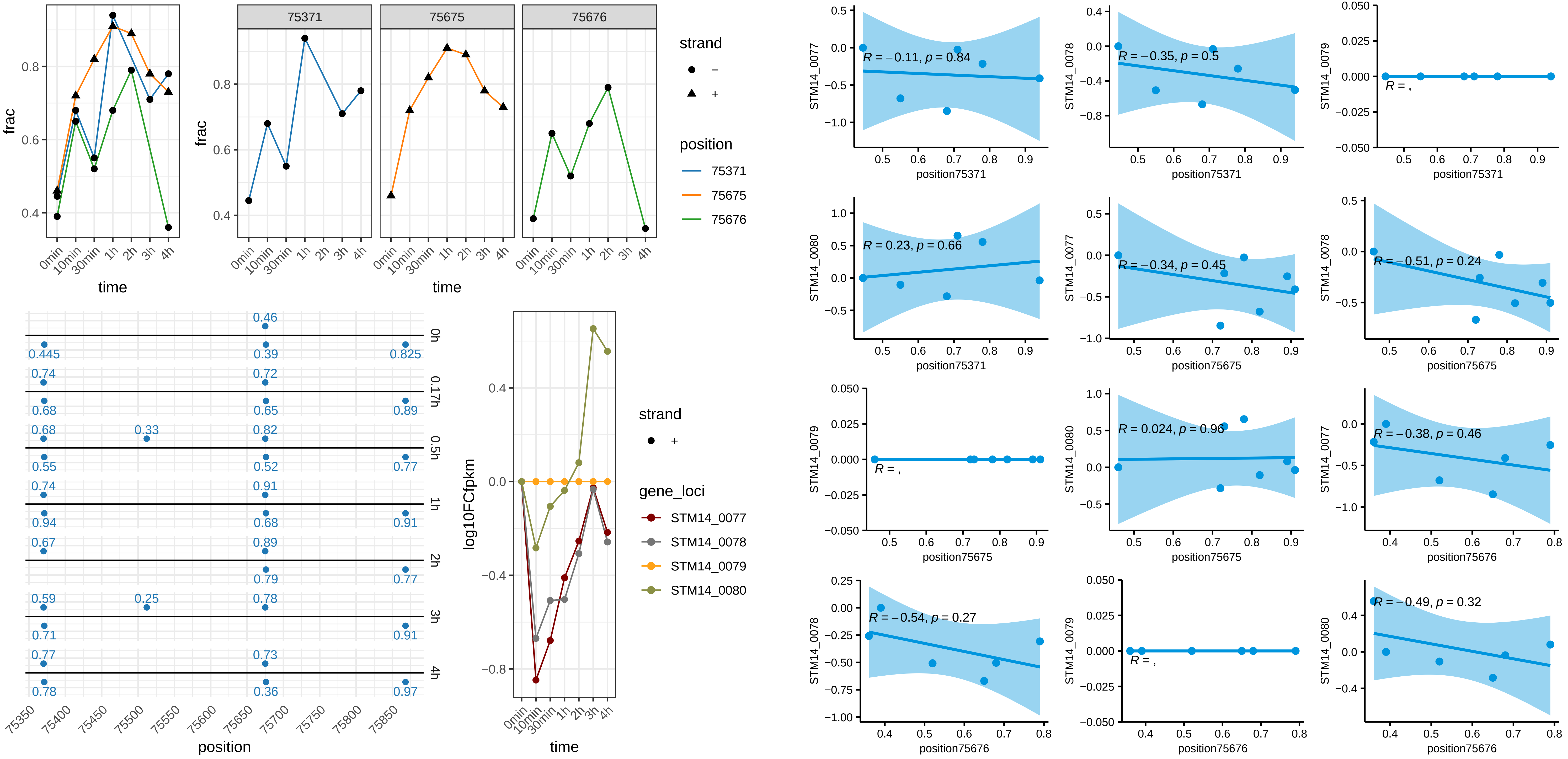
frac
time
0min
10min
30min
1h
2h
3h
4h
75371
75675
75676
strand
–
+
position
75371
75675
75676
0.445
0.39
0.825
0.46
0.74
0.72
0.68
0.65
0.89
0.68
0.33
0.82
0.55
0.52
0.77
0.74
0.91
0.94
0.68
0.91
0.67
0.89
0.79
0.77
0.59
0.25
0.78
0.71
0.91
0.77
0.73
0.78
0.36
0.97
0h
0.17h
0.5h
1h
2h
3h
4h
position
log10FCfpkm
gene_loci
STM14_0077
STM14_0078
STM14_0079
STM14_0080
R = −0.11, p = 0.84
R = −0.35, p = 0.5
R = ,
R = 0.23, p = 0.66
R = −0.34, p = 0.45
R = −0.51, p = 0.24
R = ,
R = 0.024, p = 0.96
R = −0.38, p = 0.46
R = −0.54, p = 0.27
R = ,
R = −0.49, p = 0.32
position75371
position75675
position75676

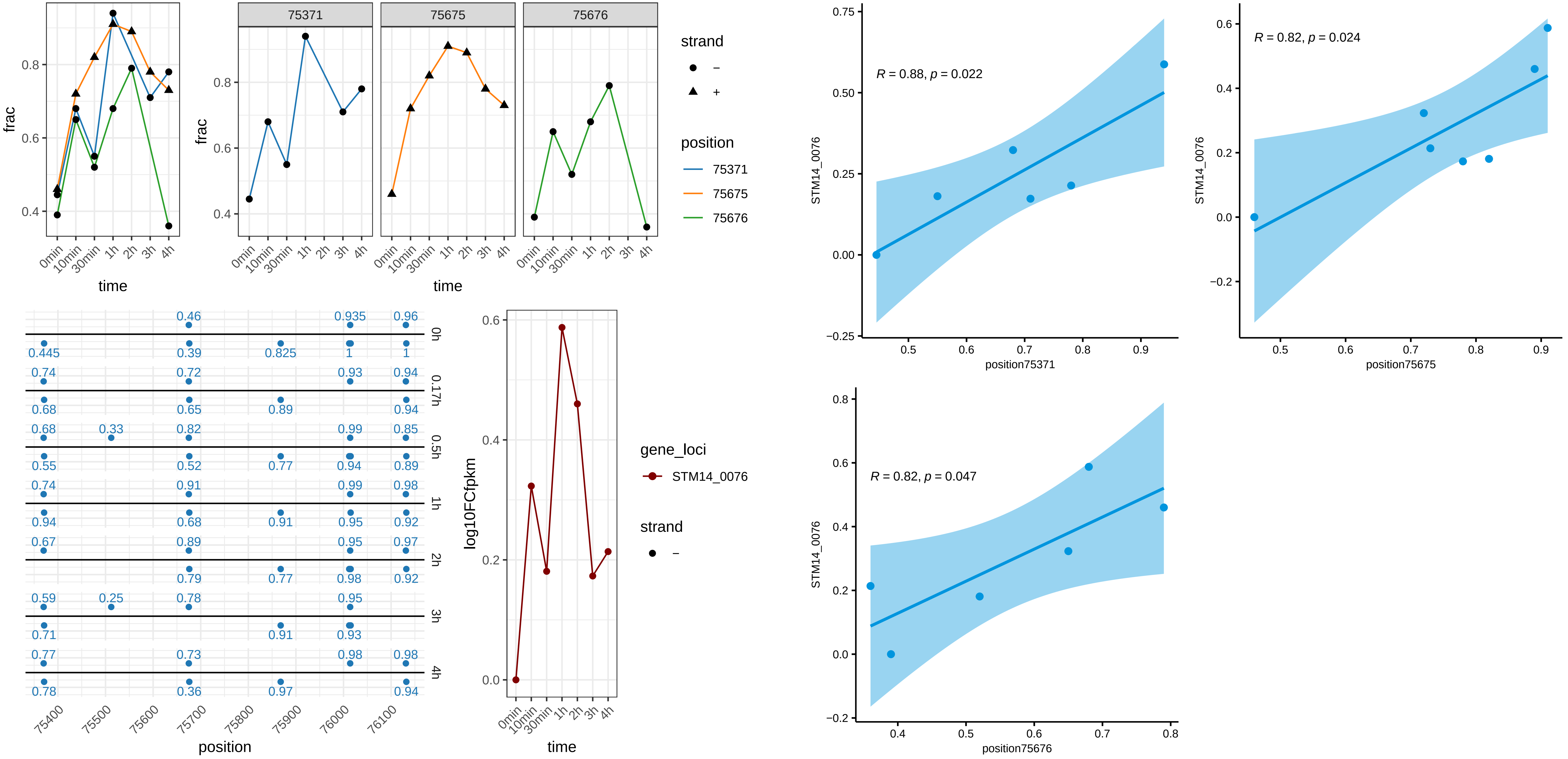

frac
time
0min
10min
30min
1h
2h
3h
4h
75371
75675
75676
strand
–
+
position
75371
75675
75676
R = 0.88, p = 0.022
STM14_0076
position75371
R = 0.82, p = 0.024
position75675
R = 0.82, p = 0.047
position75676
0h
0.17h
0.5h
1h
2h
3h
4h
position
log10FCfpkm
gene_loci
STM14_0076
strand
–

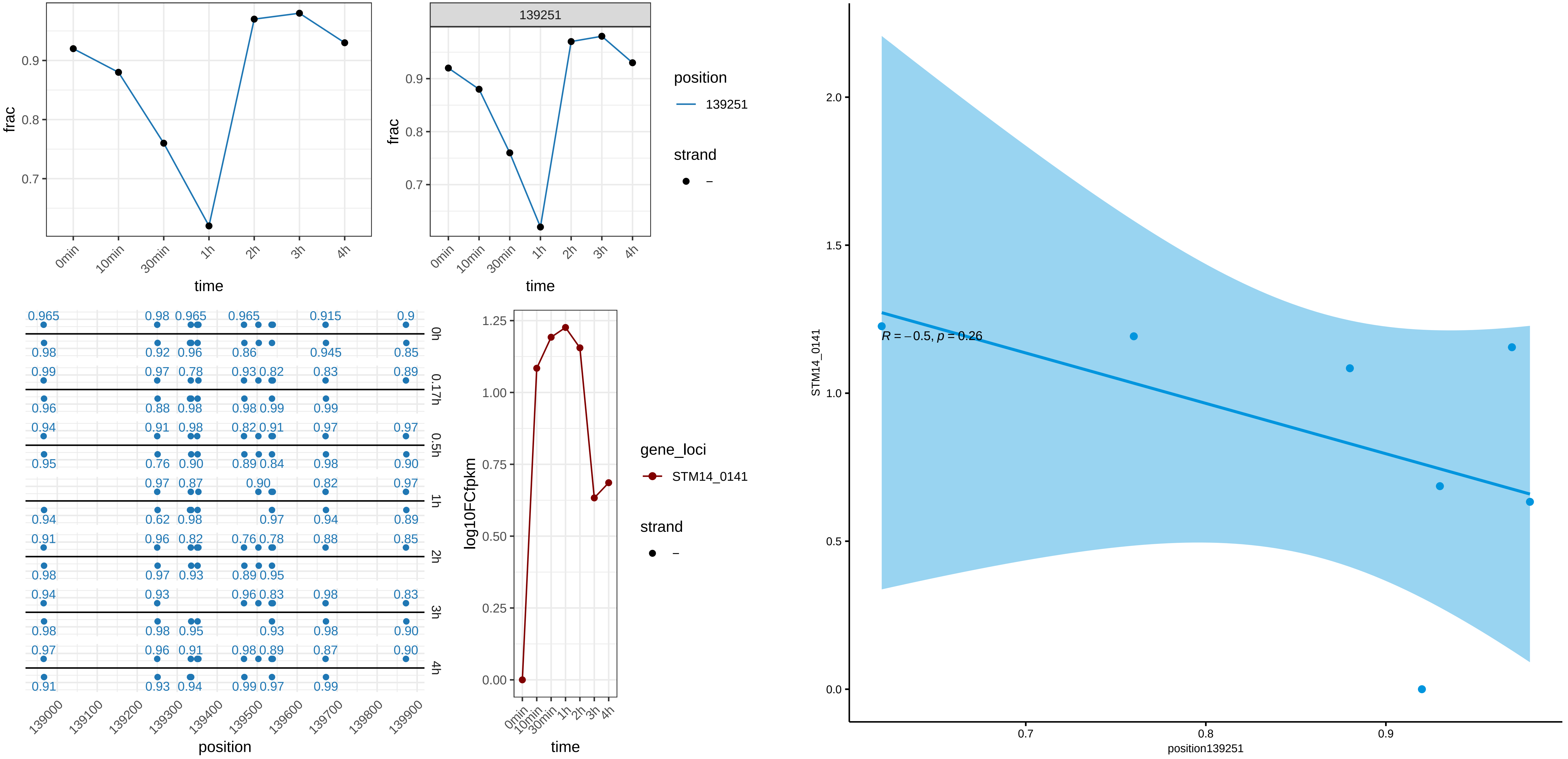
139251
frac
time
0min
10min
30min
1h
2h
3h
4h
0.7
0.8
0.9
position
139251
strand
–
position
139000
139100
139200
139300
139400
139500
139600
139700
139800
139900
0h
0.17h
0.5h
1h
2h
3h
4h
log10FCfpkm
0.00
0.25
0.50
0.75
1.00
1.25
gene_loci
STM14_0141
STM14_0141
position139251
$R = -0.5, p = 0.26$
0.0
0.5
1.0
1.5
2.0

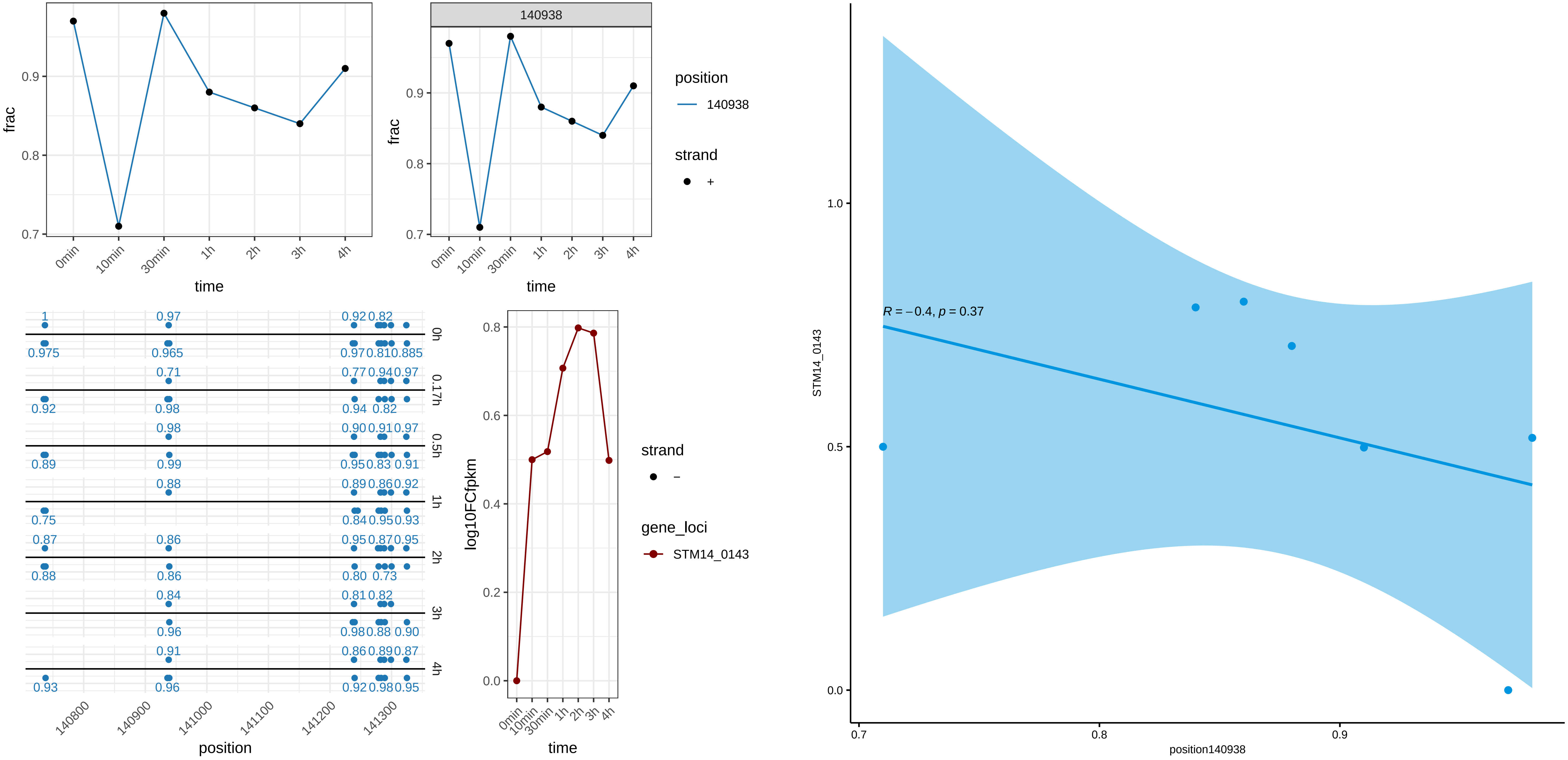

frac
time
140938
position
140938
strand
+
0min
10min
30min
1h
2h
3h
4h
position
0h
0.17h
0.5h
1h
2h
3h
4h
log10FCfpkm
strand
–
gene_loci
STM14_0143
R = −0.4, p = 0.37
STM14_0143
position140938

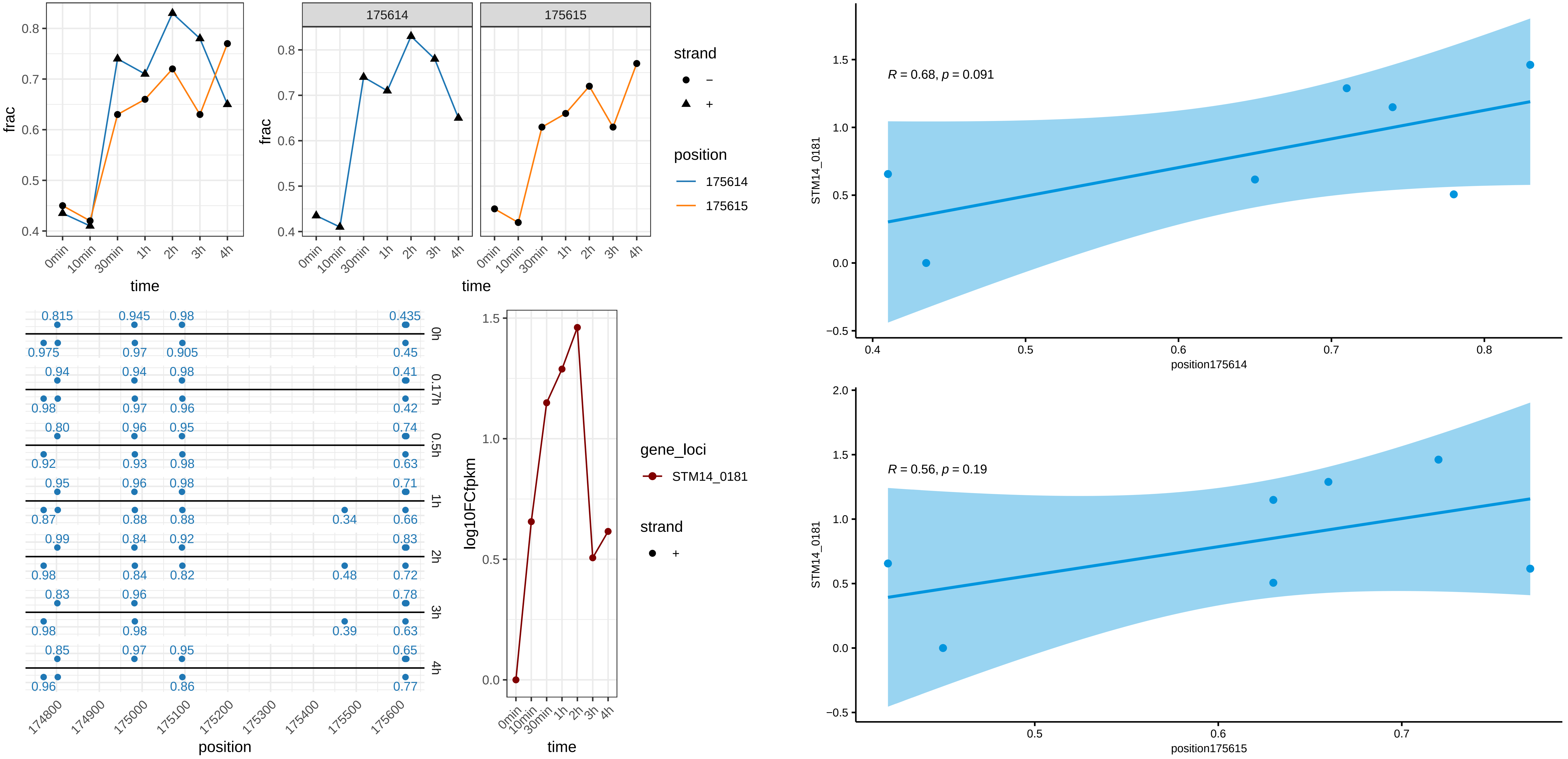

frac
time
0min
10min
30min
1h
2h
3h
4h
175614
175615
strand
–
+
position
175614
175615
0.815
0.945
0.98
0.435
0.975
0.97
0.905
0.45
0.94
0.94
0.98
0.41
0.98
0.97
0.96
0.42
0.80
0.96
0.95
0.74
0.92
0.93
0.98
0.63
0.95
0.96
0.98
0.71
0.87
0.88
0.88
0.34
0.66
0.99
0.84
0.92
0.83
0.98
0.84
0.82
0.48
0.72
0.83
0.96
0.78
0.98
0.98
0.39
0.63
0.85
0.97
0.95
0.65
0.96
0.86
0.77
0h
0.17h
0.5h
1h
2h
3h
4h
174800
174900
175000
175100
175200
175300
175400
175500
175600
position
log10FCfpkm
time
gene_loci
STM14_0181
strand
+
R = 0.68, p = 0.091
STM14_0181
position175614
R = 0.56, p = 0.19
STM14_0181
position175615

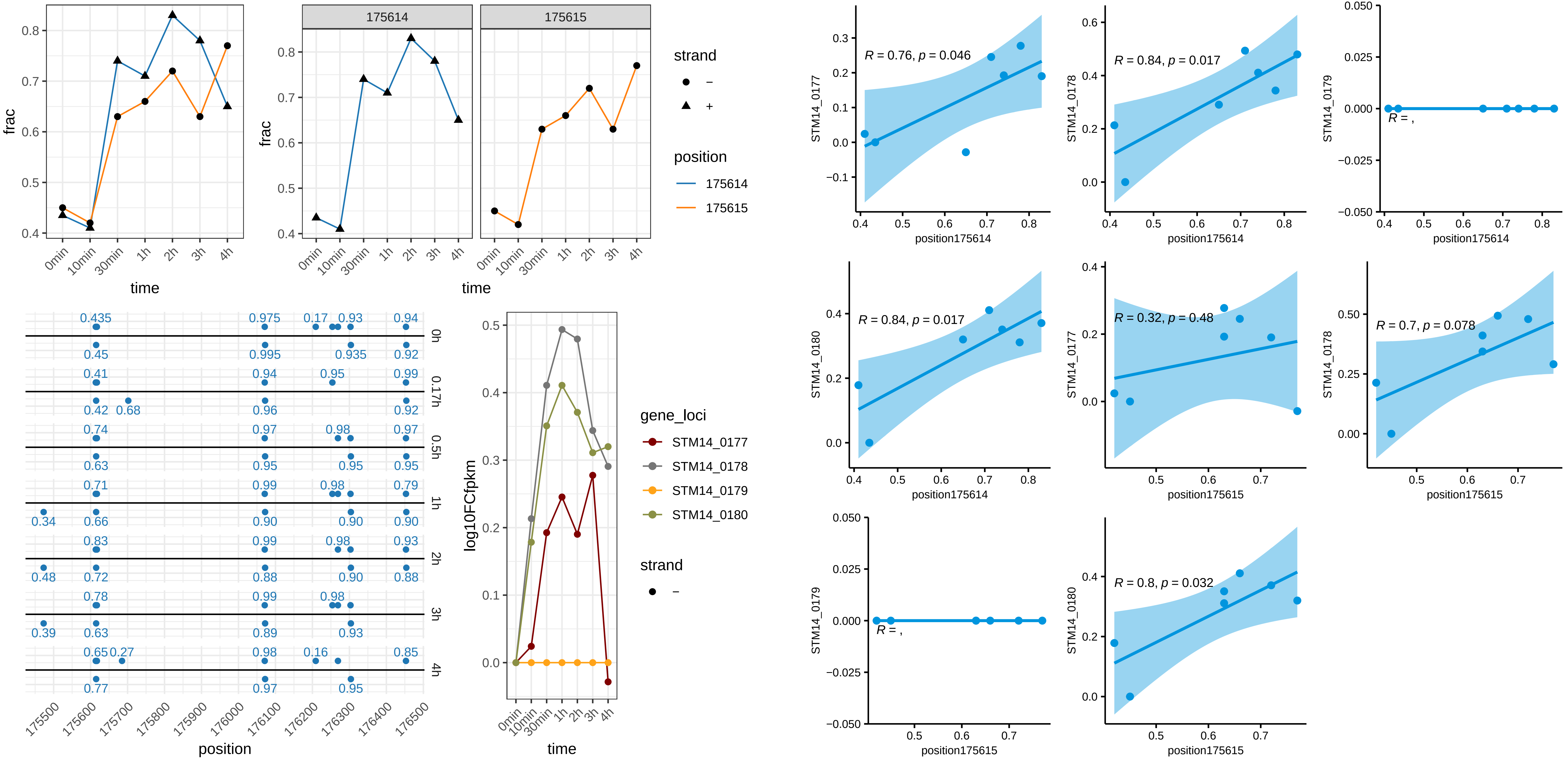

frac
time
0min
10min
30min
1h
2h
3h
4h
175614
175615
strand
–
+
position
175614
175615
position
0h
0.17h
0.5h
1h
2h
3h
4h
log10FCfpkm
gene_loci
STM14_0177
STM14_0178
STM14_0179
STM14_0180
strand
–
R = 0.76, p = 0.046
R = 0.84, p = 0.017
R = ,
R = 0.84, p = 0.017
R = 0.32, p = 0.48
R = 0.7, p = 0.078
R = ,
R = 0.8, p = 0.032
STM14_0177
STM14_0178
STM14_0179
STM14_0180
position175614
position175615

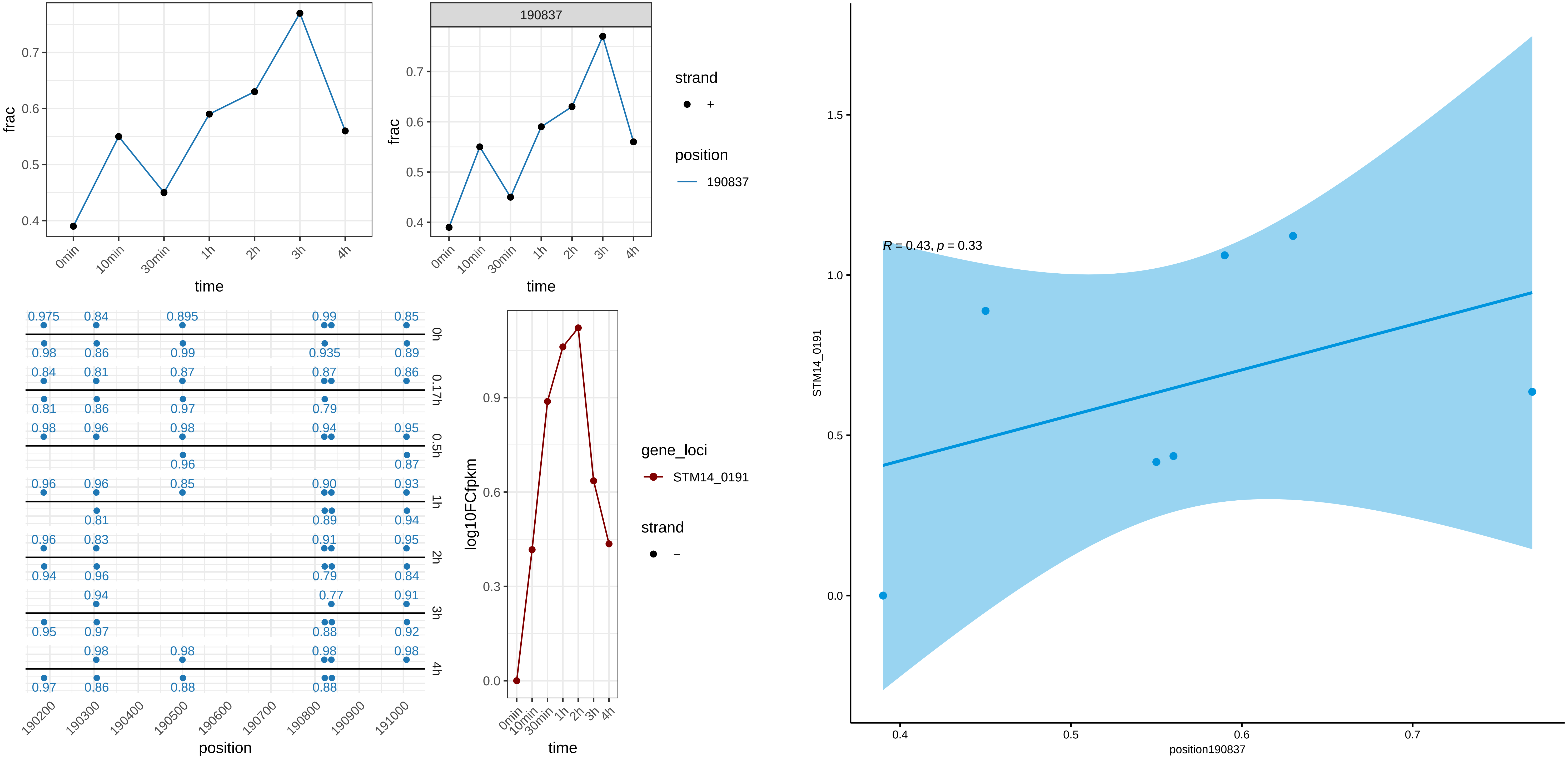

frac
time
190837
strand
+
position
190837
log10FCfpkm
gene_loci
STM14_0191
strand
–
R = 0.43, p = 0.33
STM14_0191
position190837

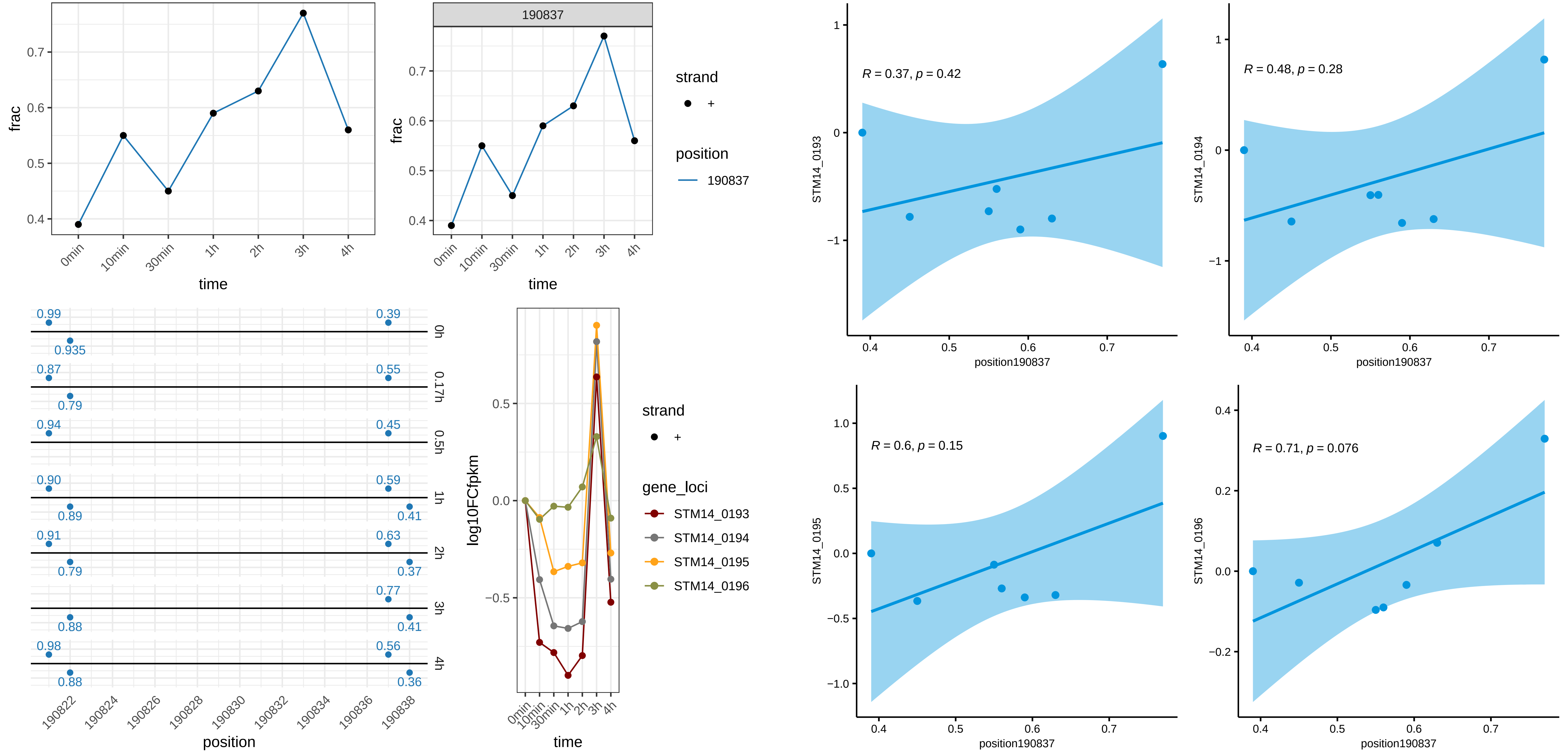

frac
0.4
0.5
0.6
0.7
0min
10min
30min
1h
2h
3h
4h
time
190837
strand
+
position
190837
0.99
0.39
0.935
0.87
0.55
0.79
0.94
0.45
0.90
0.59
0.89
0.41
0.91
0.63
0.79
0.37
0.77
0.88
0.41
0.98
0.56
0.88
0.36
0h
0.17h
0.5h
1h
2h
3h
4h
190822
190824
190826
190828
190830
190832
190834
190836
190838
position
log10FCfpkm
0.5
0.0
−0.5
gene_loci
STM14_0193
STM14_0194
STM14_0195
STM14_0196
R = 0.37, p = 0.42
STM14_0193
position190837
R = 0.48, p = 0.28
STM14_0194
R = 0.6, p = 0.15
STM14_0195
R = 0.71, p = 0.076
STM14_0196

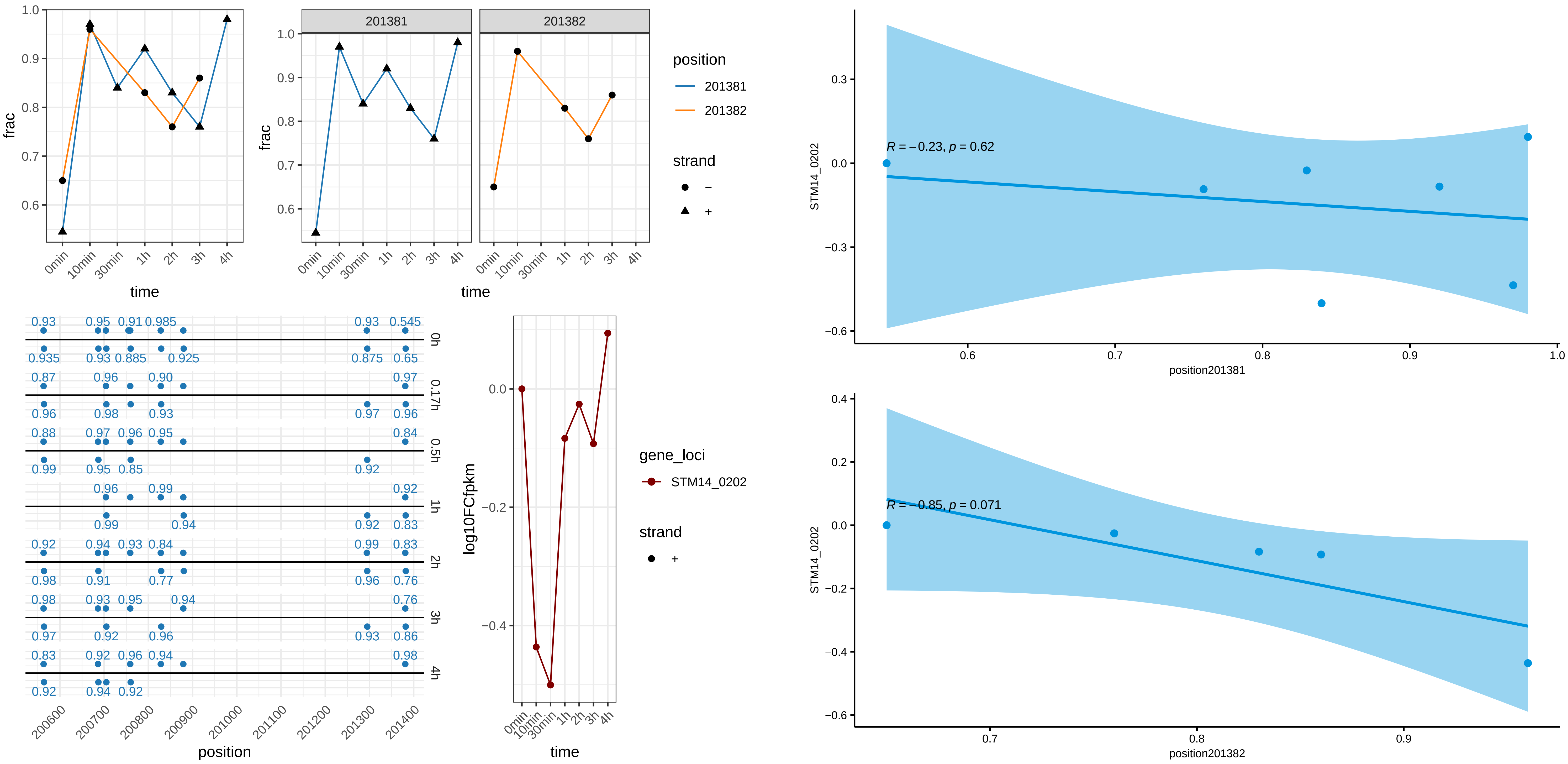
frac
time
0min
10min
30min
1h
2h
3h
4h
201381
201382
position
strand
–
+
0h
0.17h
0.5h
1h
2h
3h
4h
log10FCfpkm
gene_loci
STM14_0202
STM14_0202
position201381
R = −0.23, p = 0.62
position201382
R = −0.85, p = 0.071

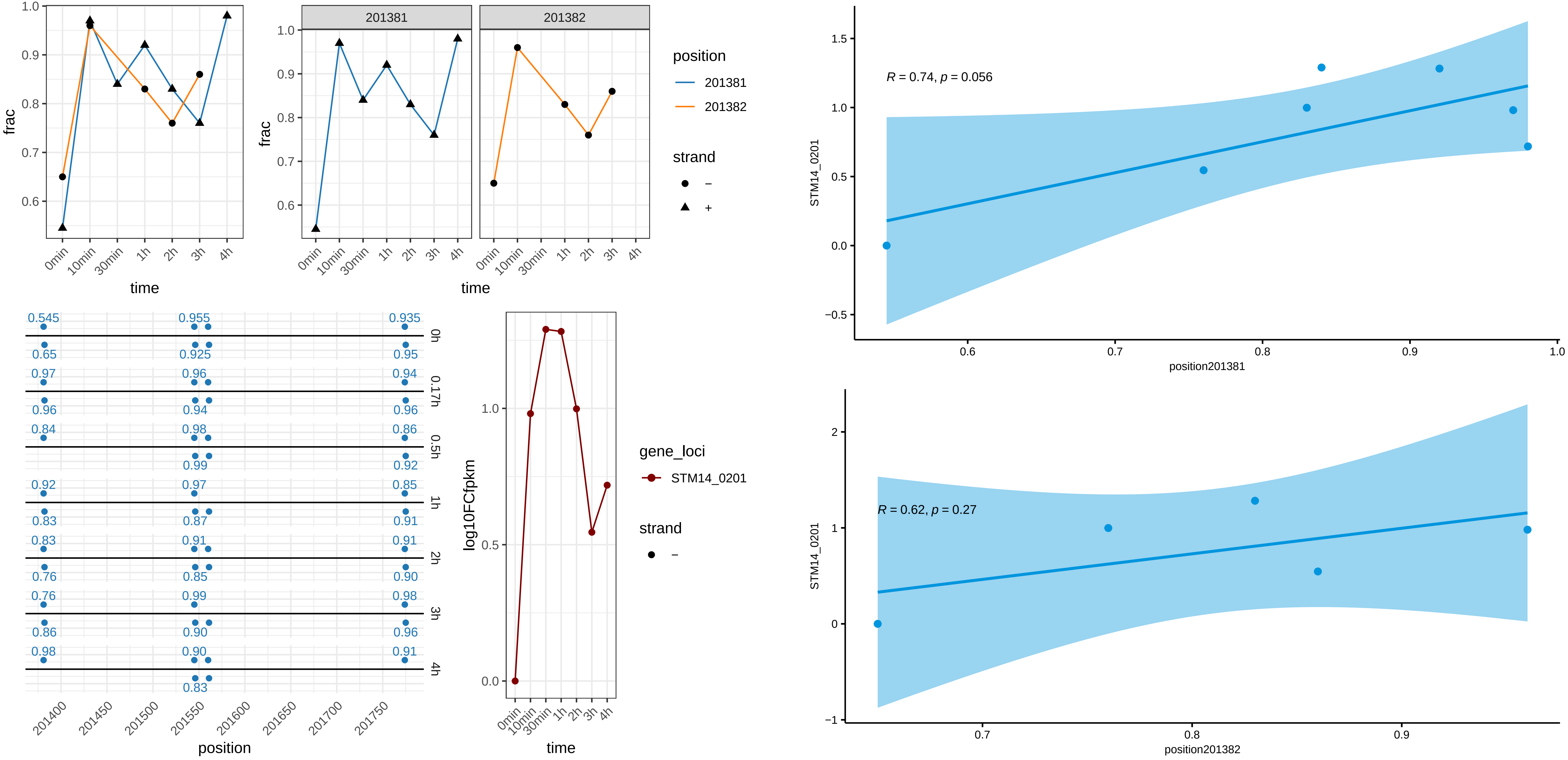
frac
time
0min
10min
30min
1h
2h
3h
4h
201381
201382
position
201381
201382
strand
–
+
R = 0.74, p = 0.056
STM14_0201
position201381
R = 0.62, p = 0.27
position201382
0.545
0.955
0.935
0.65
0.925
0.95
0.97
0.96
0.94
0.96
0.94
0.96
0.84
0.98
0.86
0.99
0.92
0.92
0.97
0.85
0.83
0.87
0.91
0.83
0.91
0.91
0.76
0.85
0.90
0.76
0.99
0.98
0.86
0.90
0.96
0.98
0.90
0.91
0.83
0h
0.17h
0.5h
1h
2h
3h
4h
position
log10FCfpkm
gene_loci
STM14_0201
strand

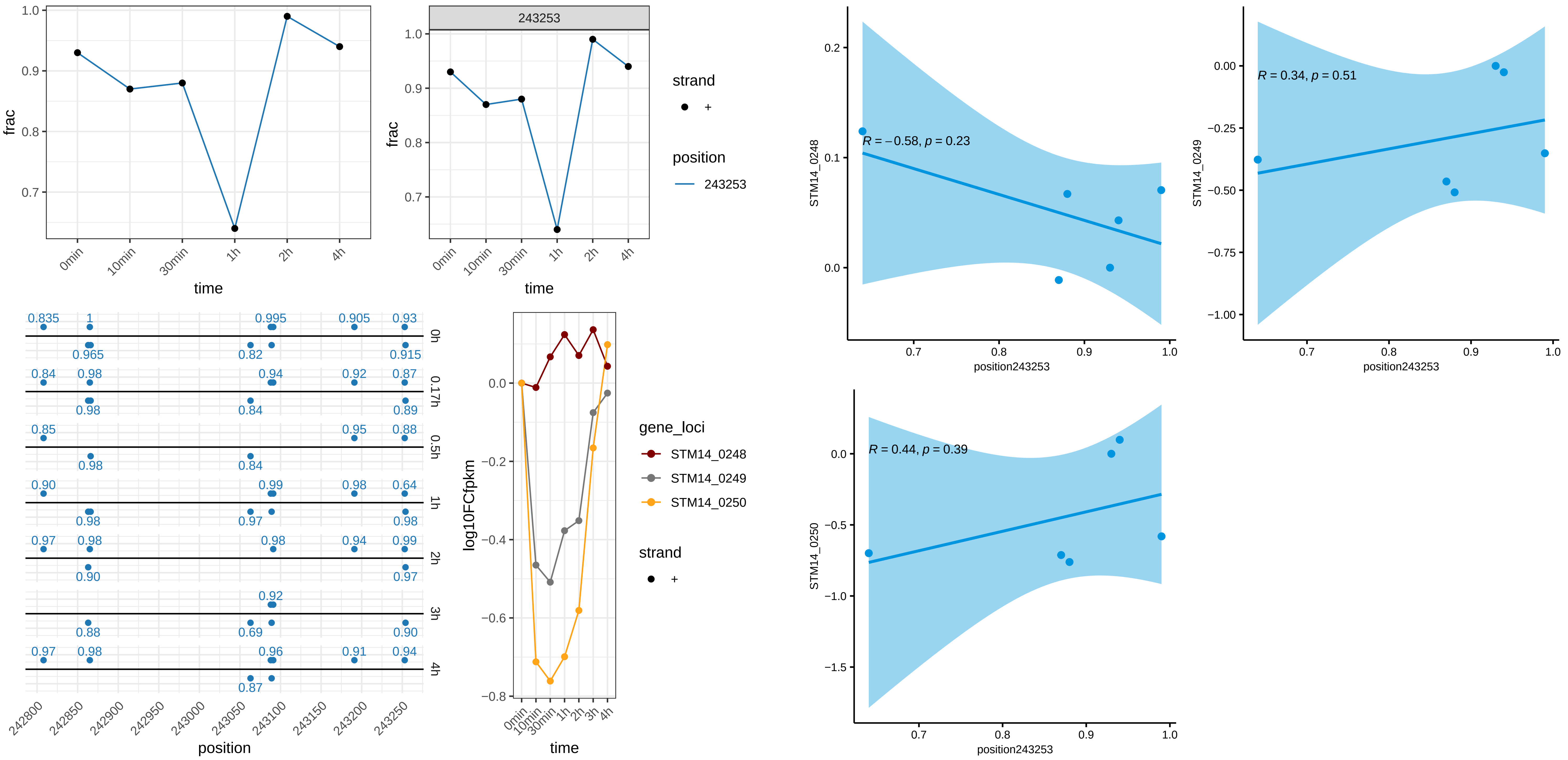

frac
time
0min
10min
30min
1h
2h
4h
243253
strand
+
position
243253
R = −0.58, p = 0.23
STM14_0248
position243253
R = 0.34, p = 0.51
STM14_0249
position243253
R = 0.44, p = 0.39
STM14_0250
position243253
0h
0.17h
0.5h
1h
2h
3h
4h
position
log10FCfpkm
time
3h
gene_loci
STM14_0248
STM14_0249
STM14_0250
strand
+

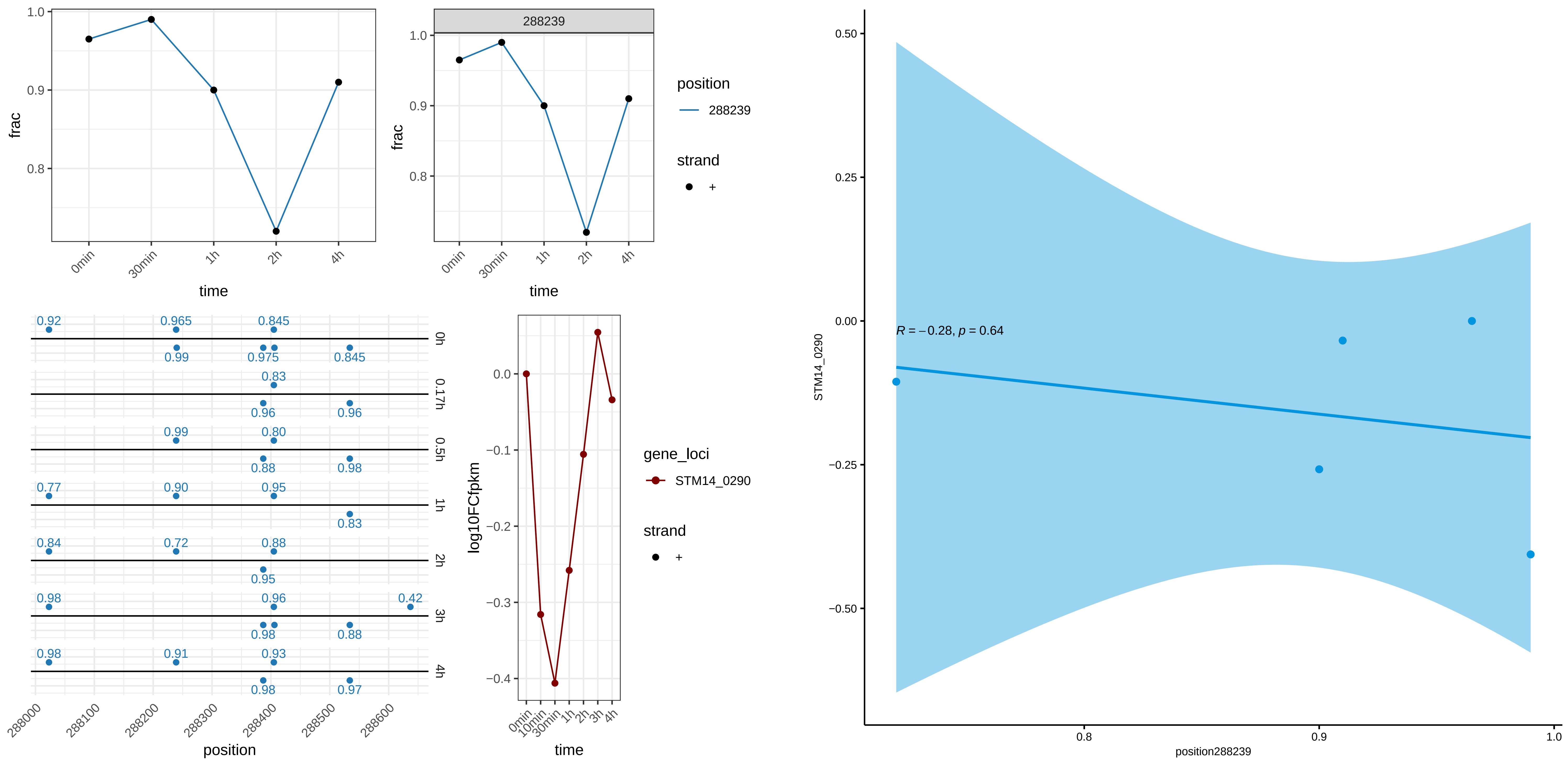

288239
frac
time
position
strand
gene_loci
STM14_0290
log10FCfpkm
R = −0.28, p = 0.64
position288239

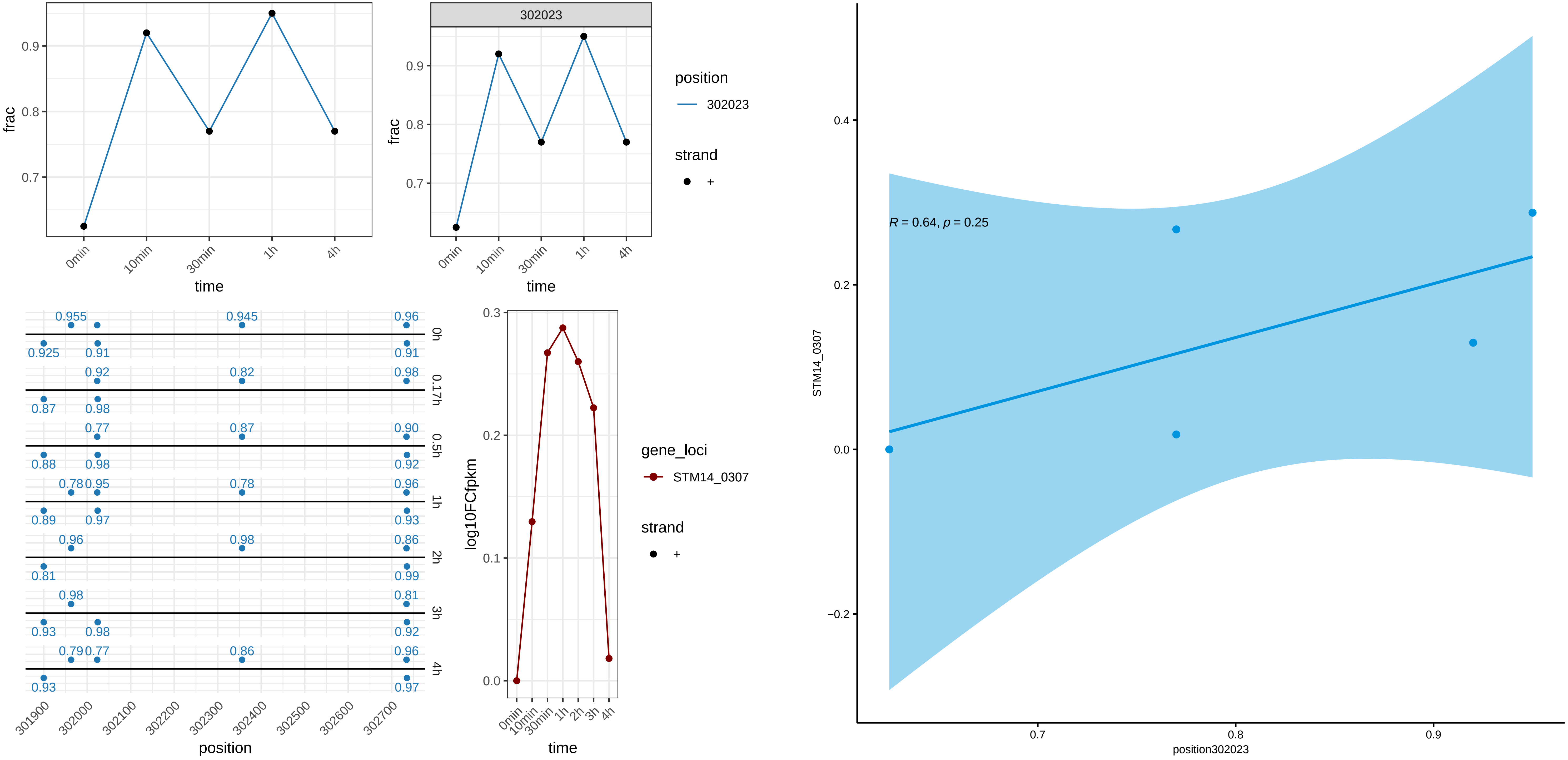

frac
time
0min
10min
30min
1h
4h
302023
position
302023
strand
+
0.955
0.945
0.96
0.925
0.91
0.91
0.92
0.82
0.98
0.87
0.98
0.77
0.87
0.90
0.88
0.98
0.92
0.78
0.95
0.78
0.96
0.89
0.97
0.93
0.96
0.98
0.86
0.81
0.99
0.98
0.81
0.93
0.98
0.92
0.79
0.77
0.86
0.96
0.93
0.97
0h
0.17h
0.5h
1h
2h
3h
4h
position
log10FCfpkm
2h
3h
gene_loci
STM14_0307
strand
+
R = 0.64, p = 0.25
STM14_0307
position302023

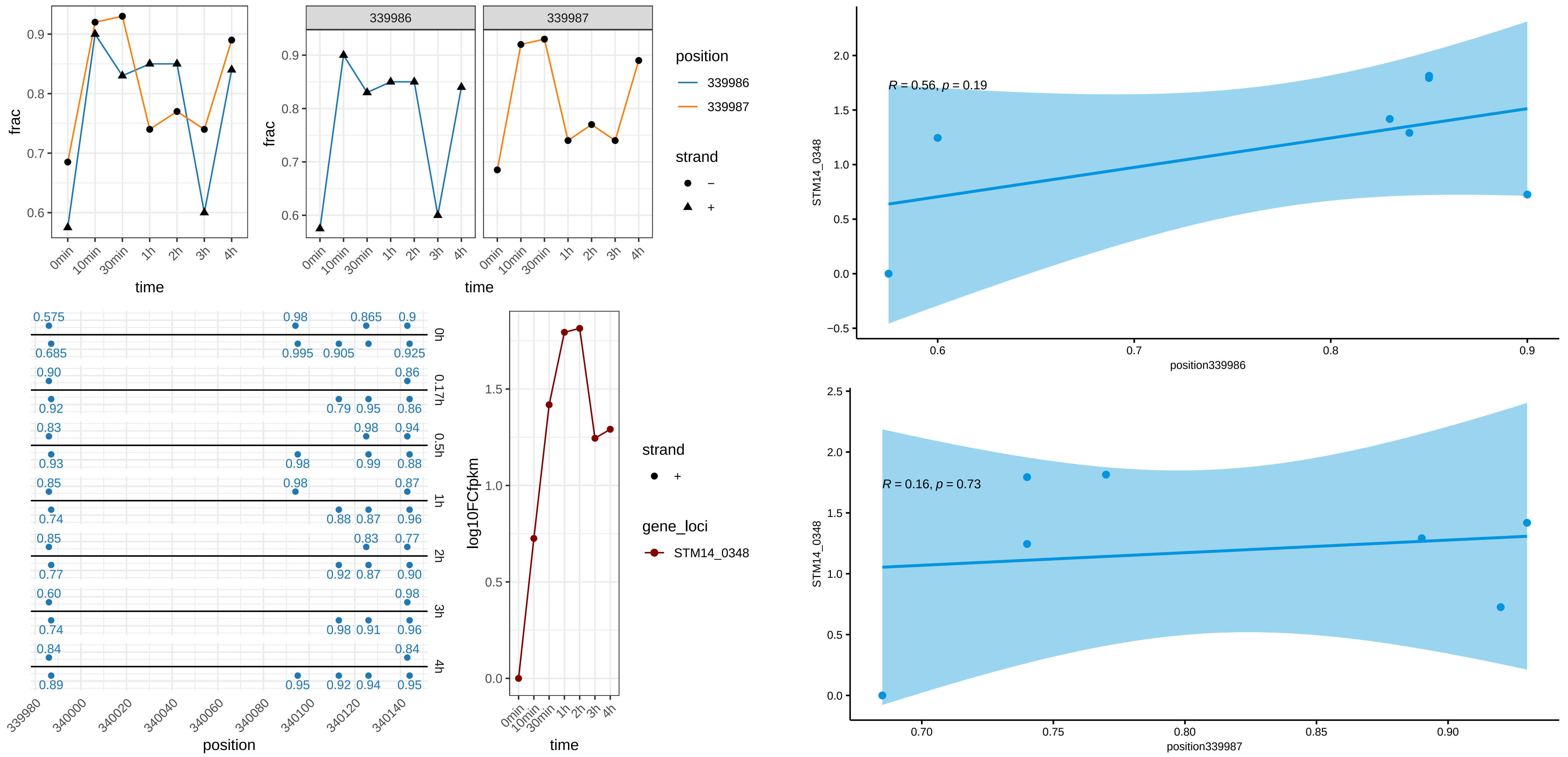

frac
time
0min
10min
30min
1h
2h
3h
4h
339986
339987
position
strand
–
+
R = 0.56, p = 0.19
STM14_0348
position339986
R = 0.16, p = 0.73
position339987
0.575
0.98
0.865
0.9
0.685
0.995
0.905
0.925
0h
0.17h
0.5h
1h
2h
3h
4h
log10FCfpkm
gene_loci
STM14_0348

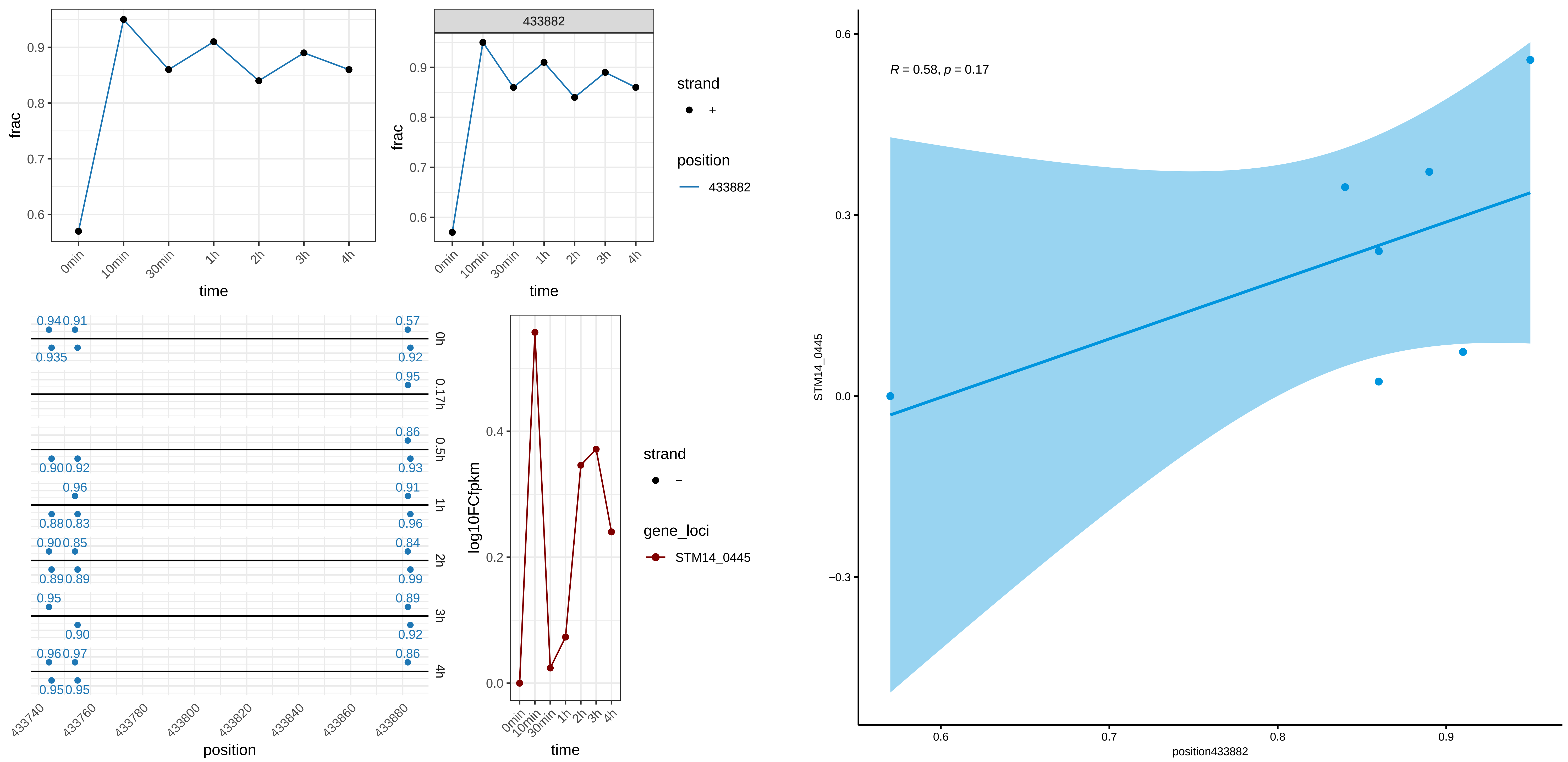

frac
time
433882
strand
+
position
433882
0.94
0.91
0.57
0.935
0.92
0.95
0.86
0.90
0.92
0.93
0.96
0.91
0.88
0.83
0.96
0.90
0.85
0.84
0.89
0.89
0.99
0.95
0.89
0.90
0.92
0.96
0.97
0.86
0.95
0.95
0h
0.17h
0.5h
1h
2h
3h
4h
position
log10FCfpkm
gene_loci
STM14_0445
R = 0.58, p = 0.17
STM14_0445
position433882

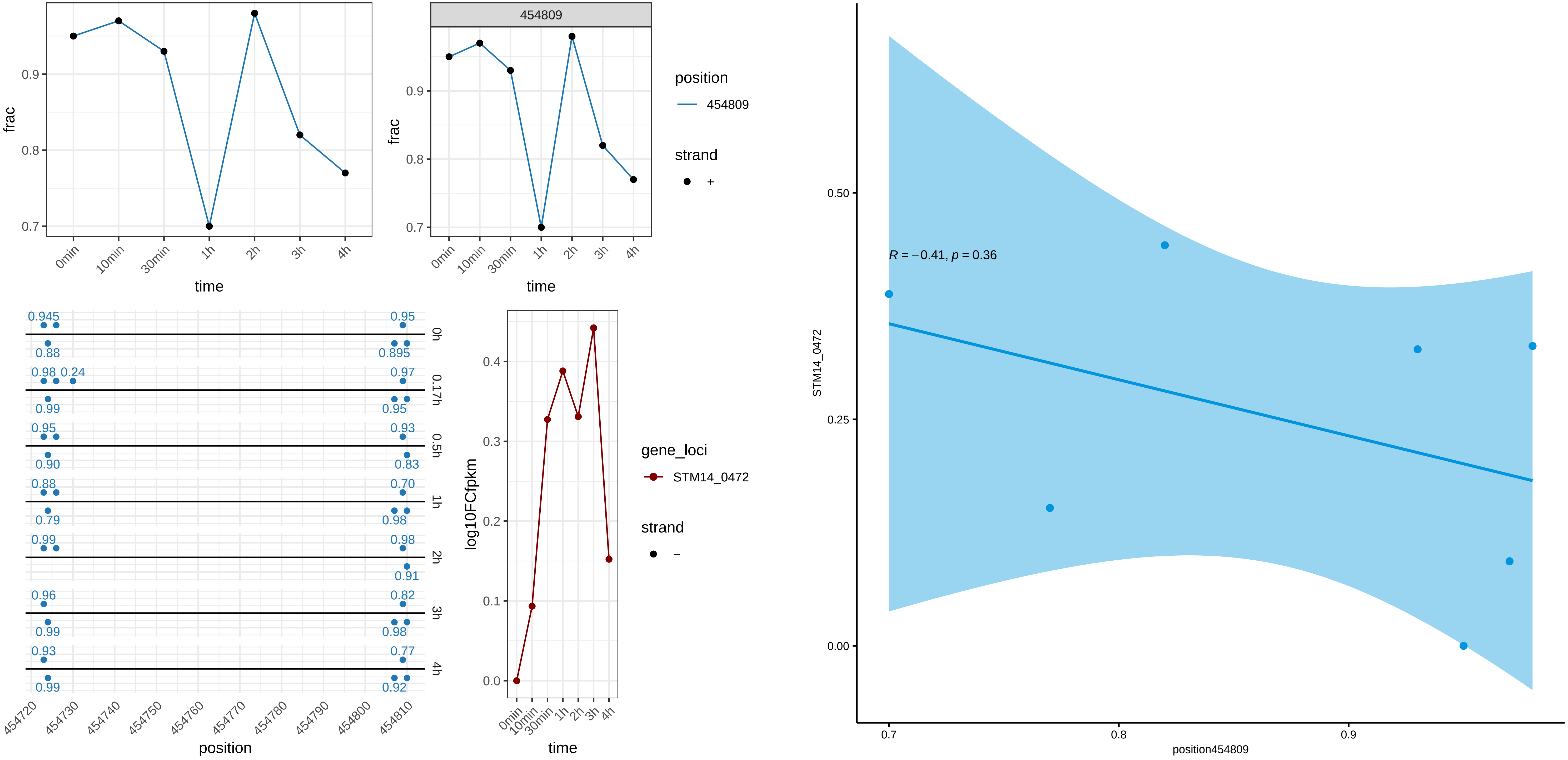

frac
time
0min
10min
30min
1h
2h
3h
4h
454809
position
strand
+
position
0h
0.17h
0.5h
1h
2h
3h
4h
log10FCfpkm
gene_loci
STM14_0472
−
R = −0.41, p = 0.36
STM14_0472
position454809

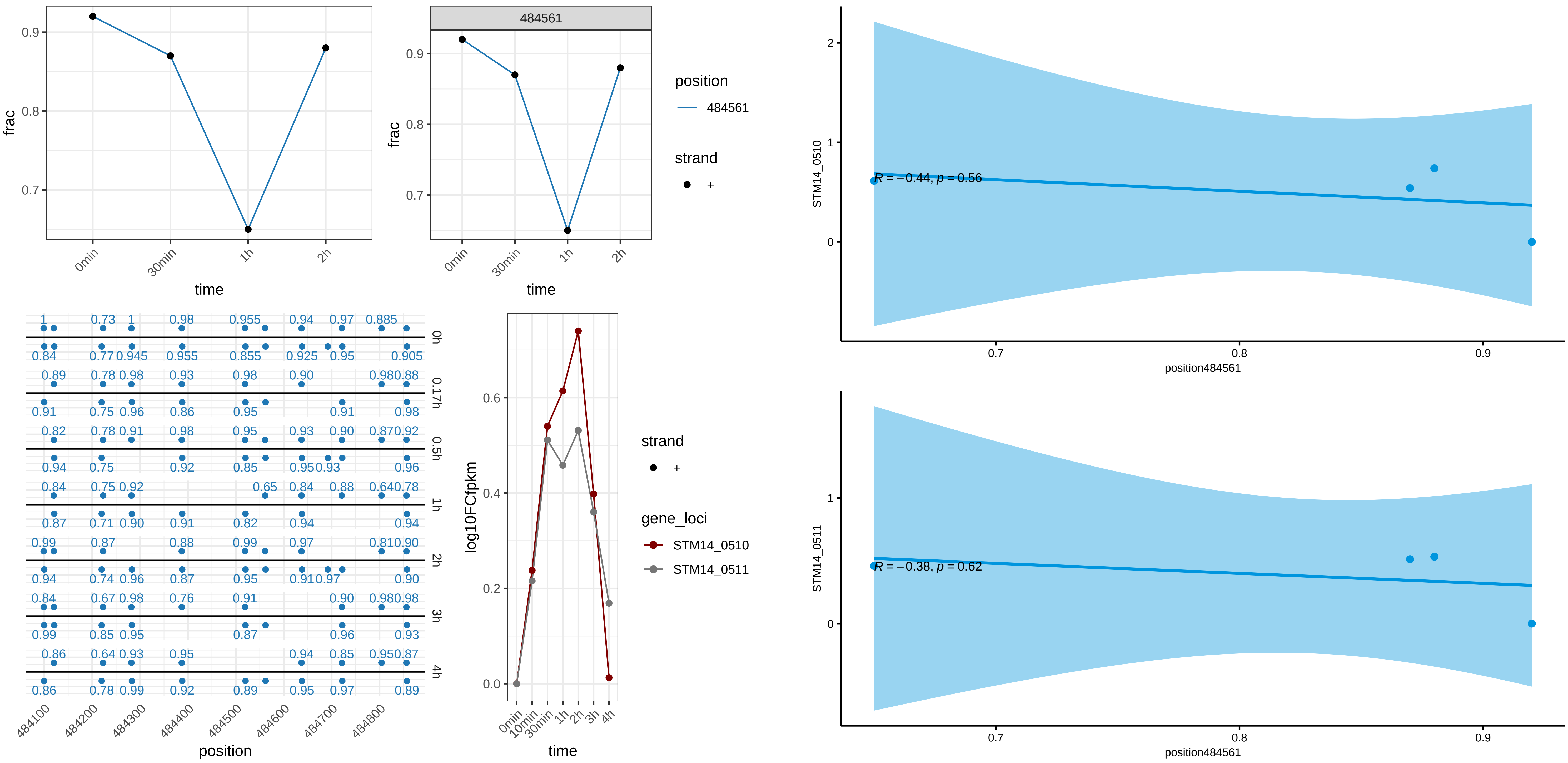

frac
time
0min
30min
1h
2h
484561
position
484561
strand
+
log10FCfpkm
0min
10min
30min
1h
2h
3h
4h
gene_loci
STM14_0510
STM14_0511
0h
0.17h
0.5h
1h
2h
3h
4h
484100
484200
484300
484400
484500
484600
484700
484800
STM14_0510
R = −0.44, p = 0.56
position484561
STM14_0511
R = −0.38, p = 0.62
position484561

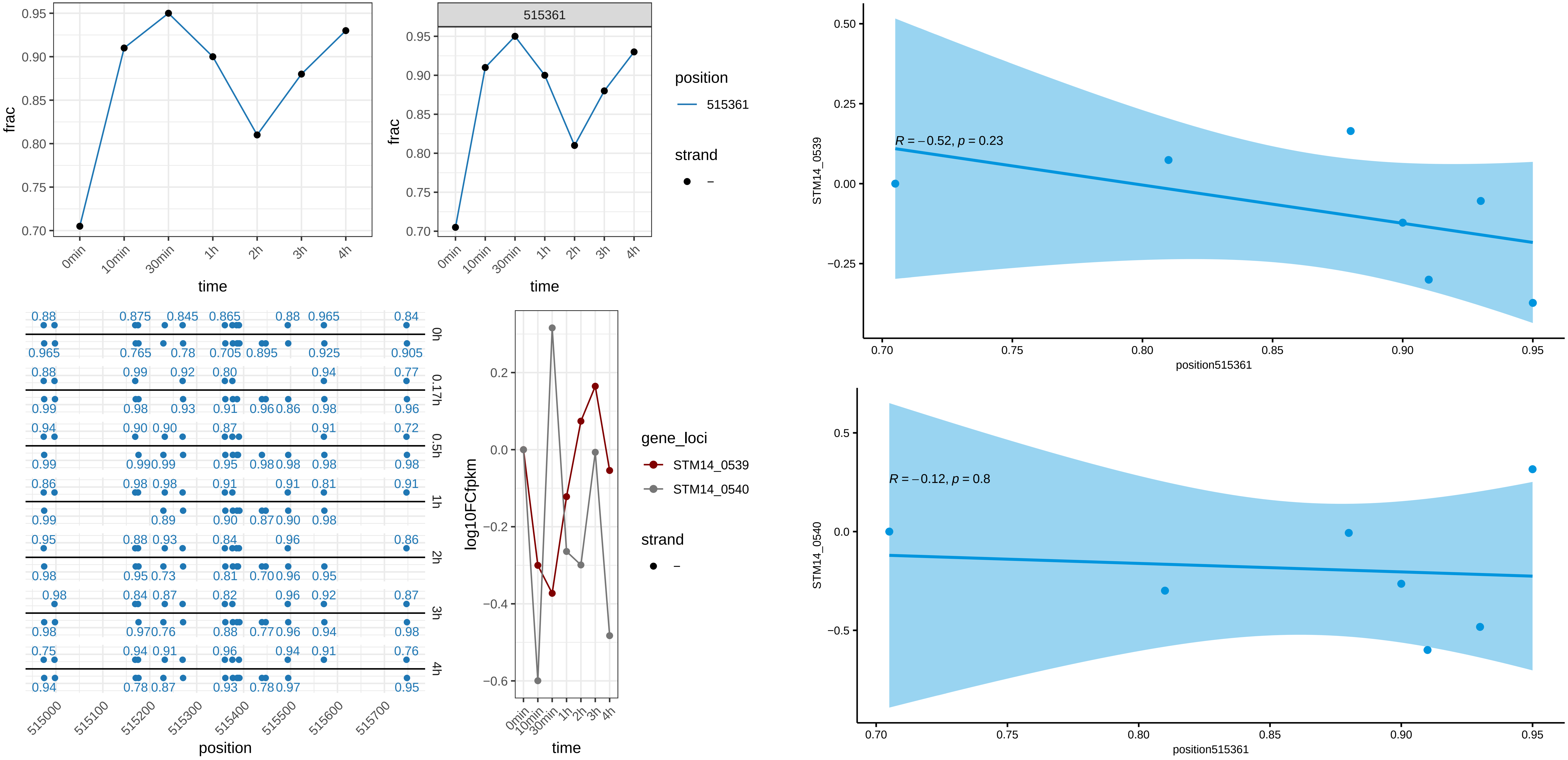
frac
time
515361
position
515361
strand
log10FCfpkm
gene_loci
STM14_0539
STM14_0540
R = −0.52, p = 0.23
STM14_0539
position515361
R = −0.12, p = 0.8
STM14_0540
position515361

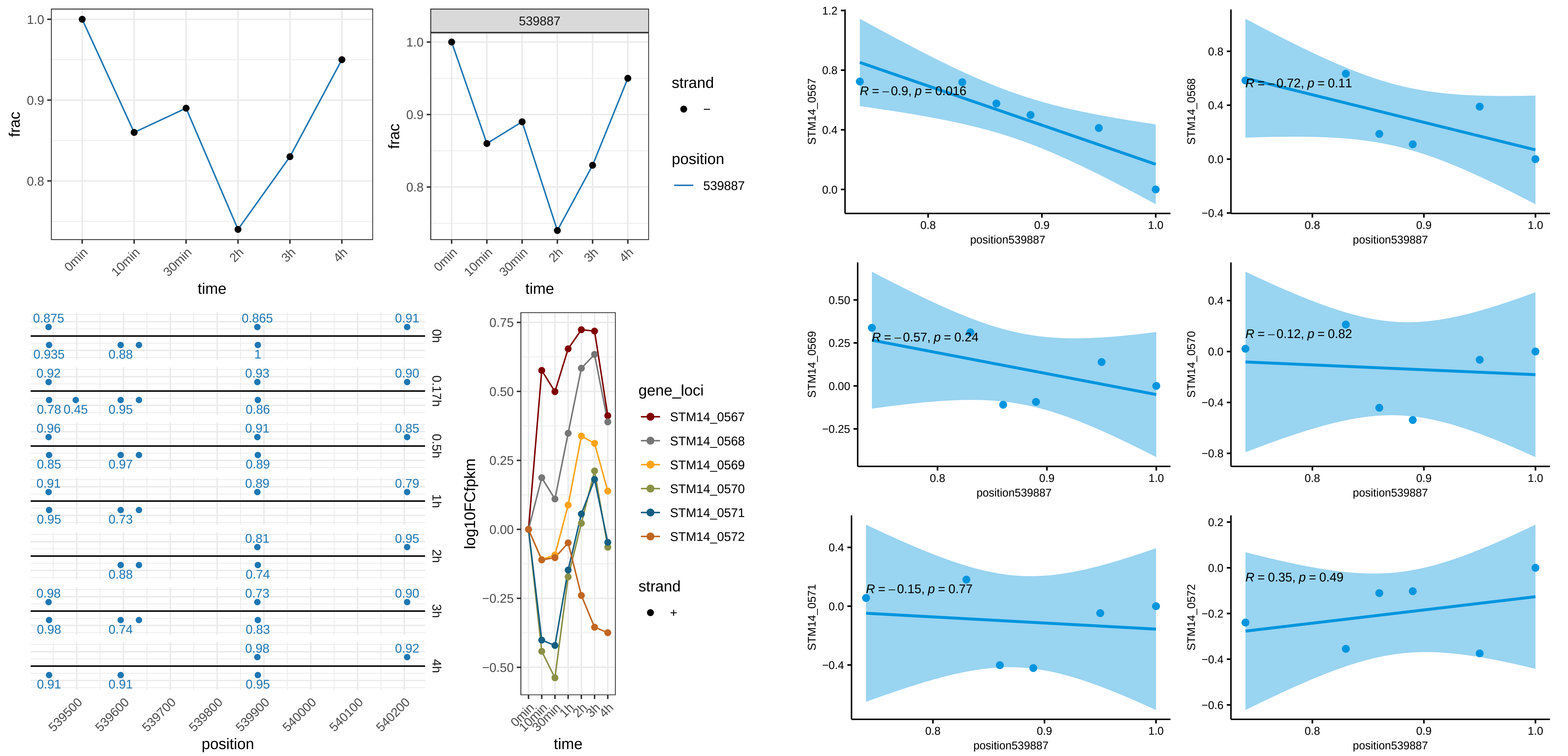

frac
time
539887
strand
position
539887
0h
0.17h
0.5h
1h
2h
3h
4h
position
log10FCfpkm
gene_loci
STM14_0567
STM14_0568
STM14_0569
STM14_0570
STM14_0571
STM14_0572
R = −0.9, p = 0.016
R = −0.72, p = 0.11
R = −0.57, p = 0.24
R = −0.12, p = 0.82
R = −0.15, p = 0.77
R = 0.35, p = 0.49
position539887

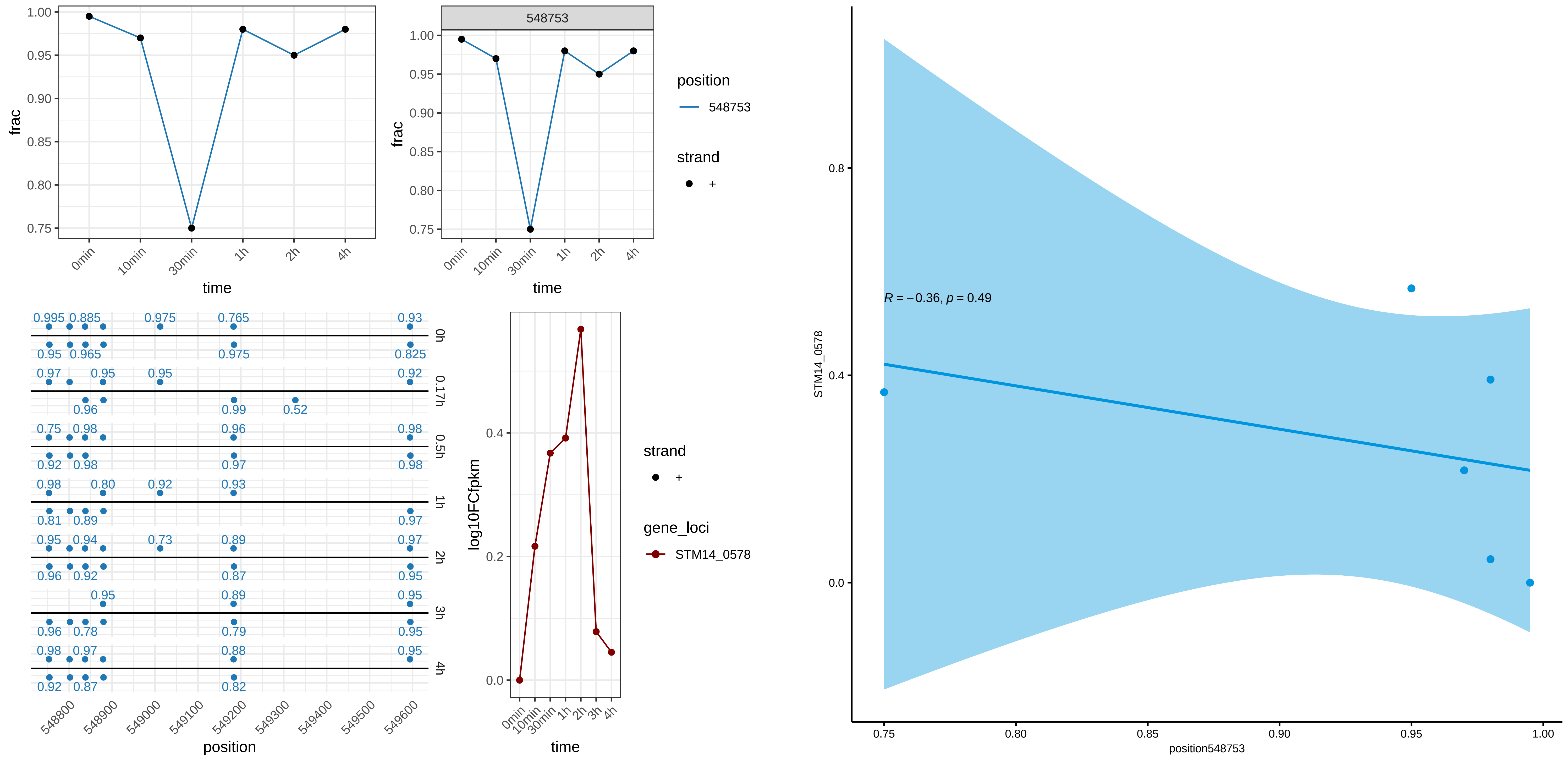

frac
time
548753
position
548753
strand
+
position
log10FCfpkm
time
strand
+
gene_loci
STM14_0578
0h
0.17h
0.5h
1h
2h
3h
4h
R = −0.36, p = 0.49
STM14_0578
position548753

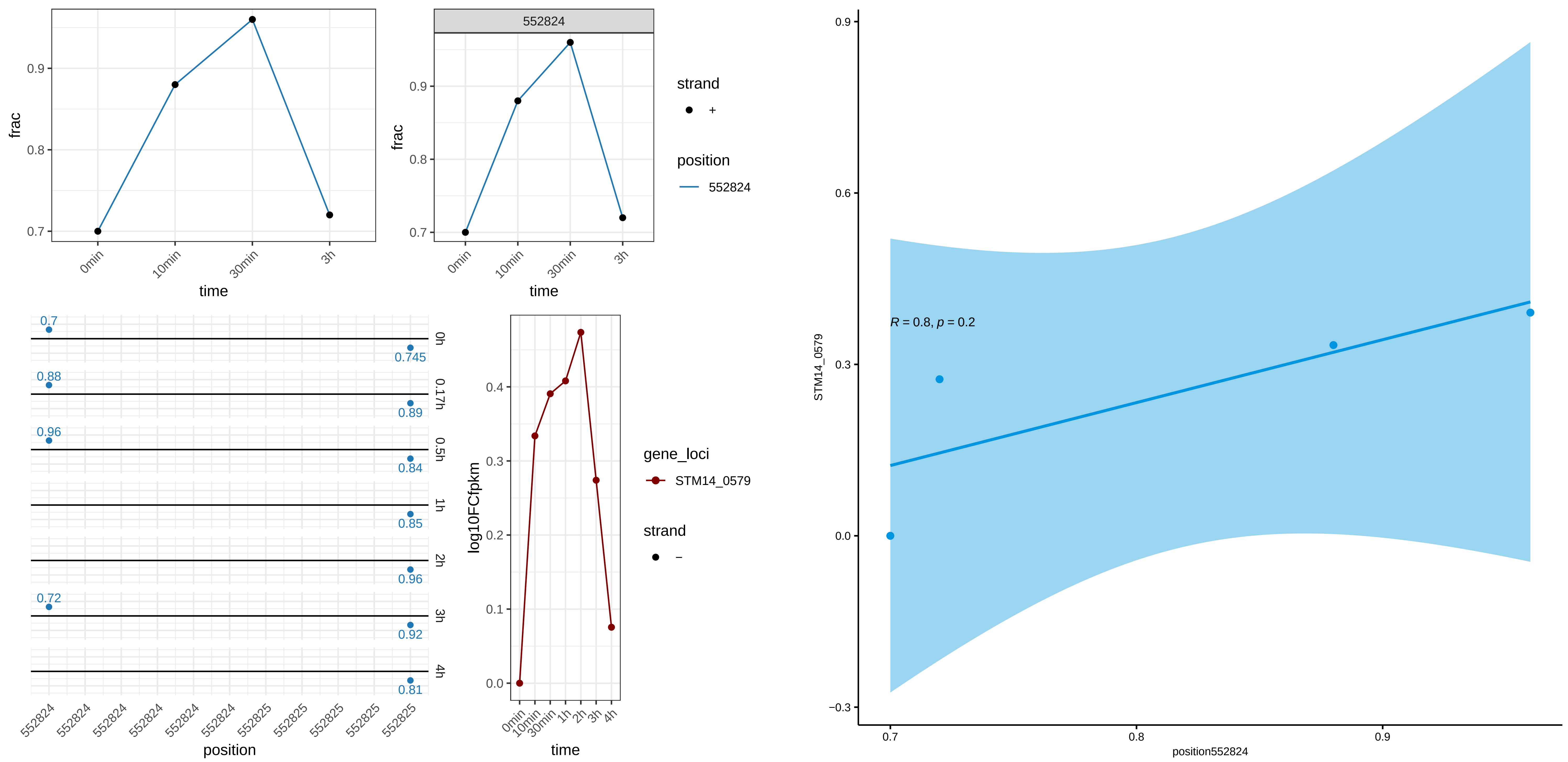

frac
time
0min
10min
30min
3h
552824
strand
+
position
552824
gene_loci
STM14_0579
–
log10FCfpkm
0h
0.17h
0.5h
1h
2h
3h
4h
position
552825
R = 0.8, p = 0.2
STM14_0579
position552824

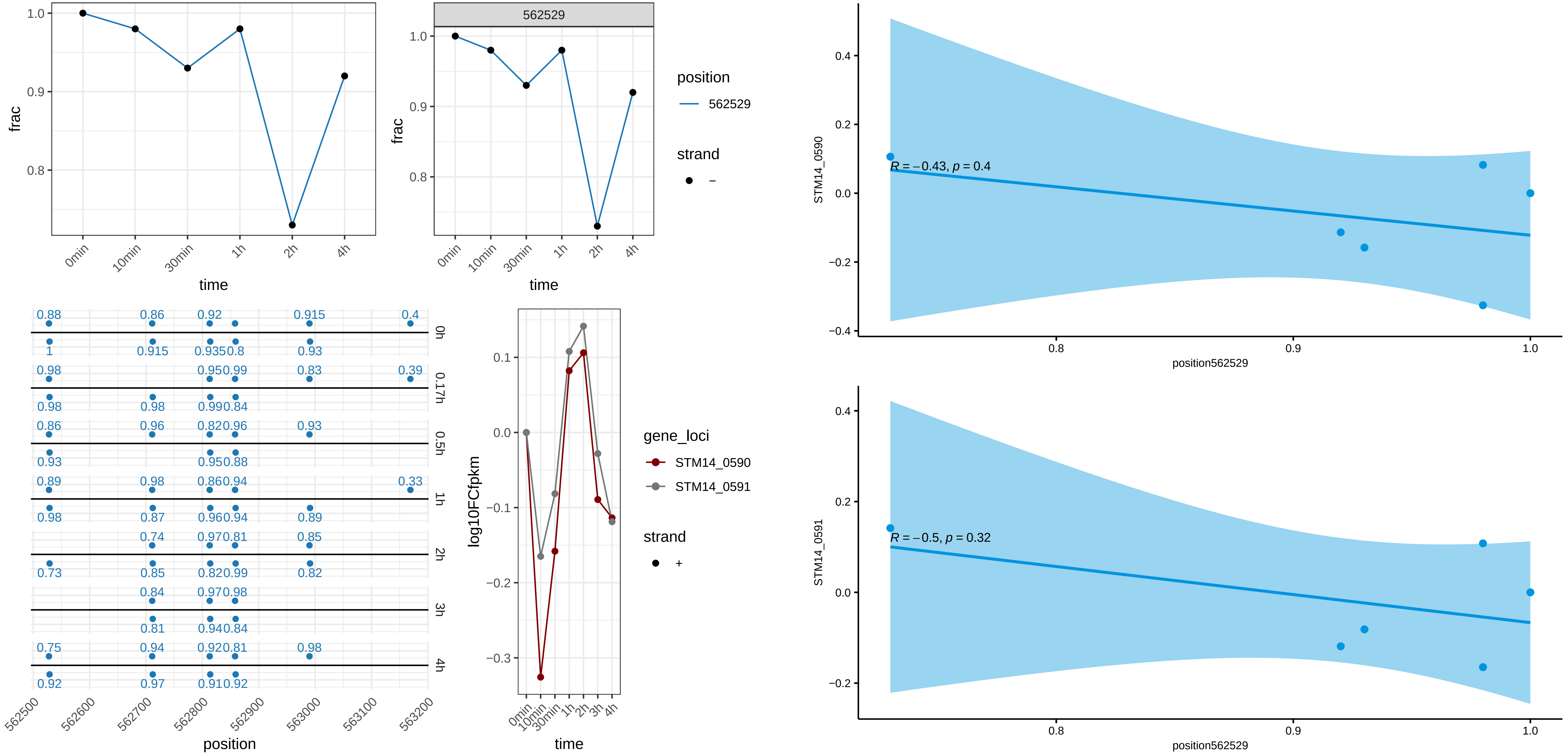

frac
time
0min
10min
30min
1h
2h
4h
562529
position
562529
strand
–
log10FCfpkm
gene_loci
STM14_0590
STM14_0591
strand
+
position
0h
0.17h
0.5h
1h
2h
3h
4h
STM14_0590
R = −0.43, p = 0.4
position562529
STM14_0591
R = −0.5, p = 0.32
position562529

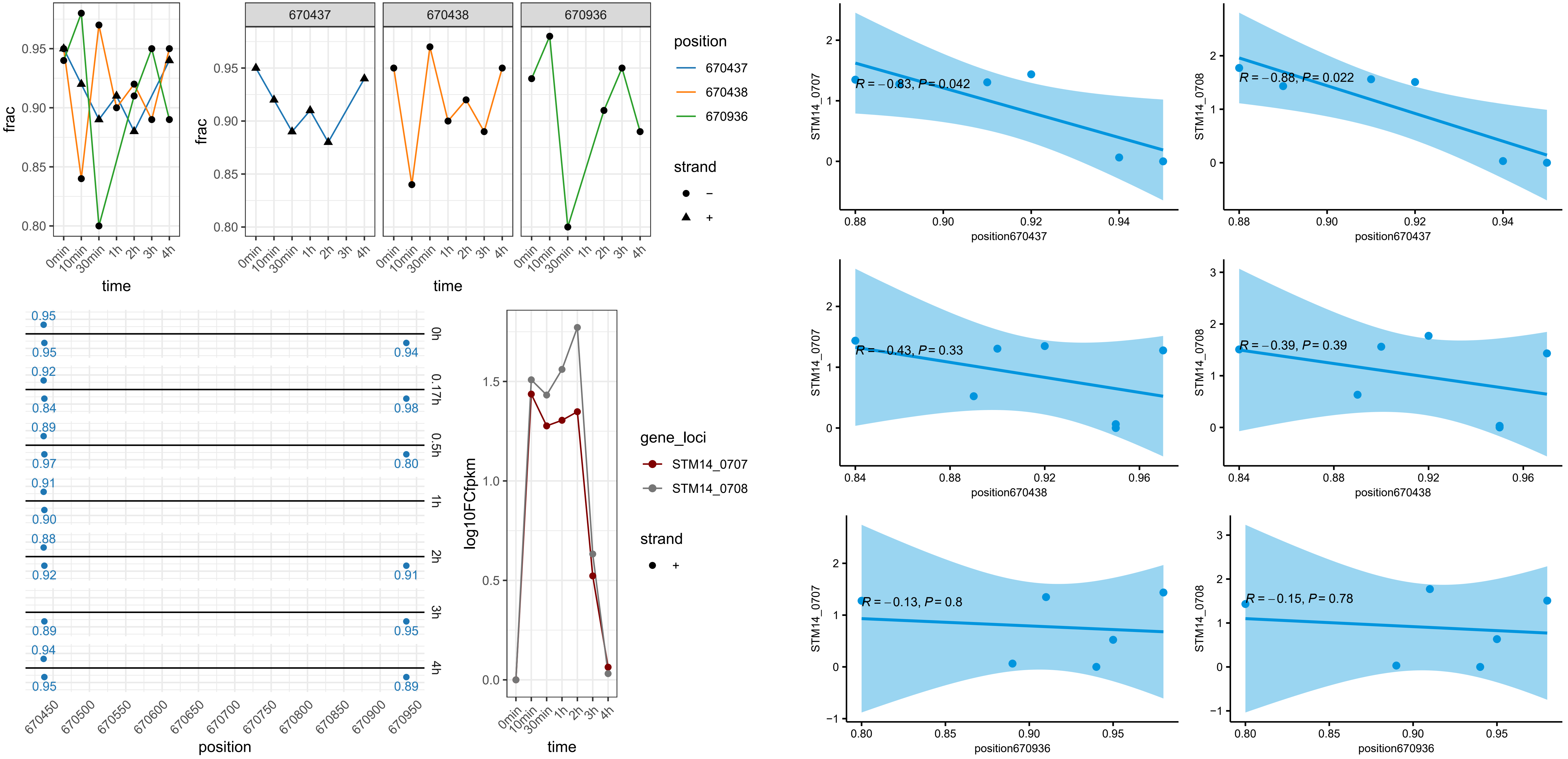

frac
time
0min
10min
30min
1h
2h
3h
4h
670437
670438
670936
position
strand
gene_loci
STM14_0707
STM14_0708
log10FCfpkm
0h
0.17h
0.5h
1h
2h
3h
4h
position
R = −0.83, P = 0.042
R = −0.88, P = 0.022
R = −0.43, P = 0.33
R = −0.39, P = 0.39
R = −0.13, P = 0.8
R = −0.15, P = 0.78
position670437
position670438
position670936
STM14_0707
STM14_0708

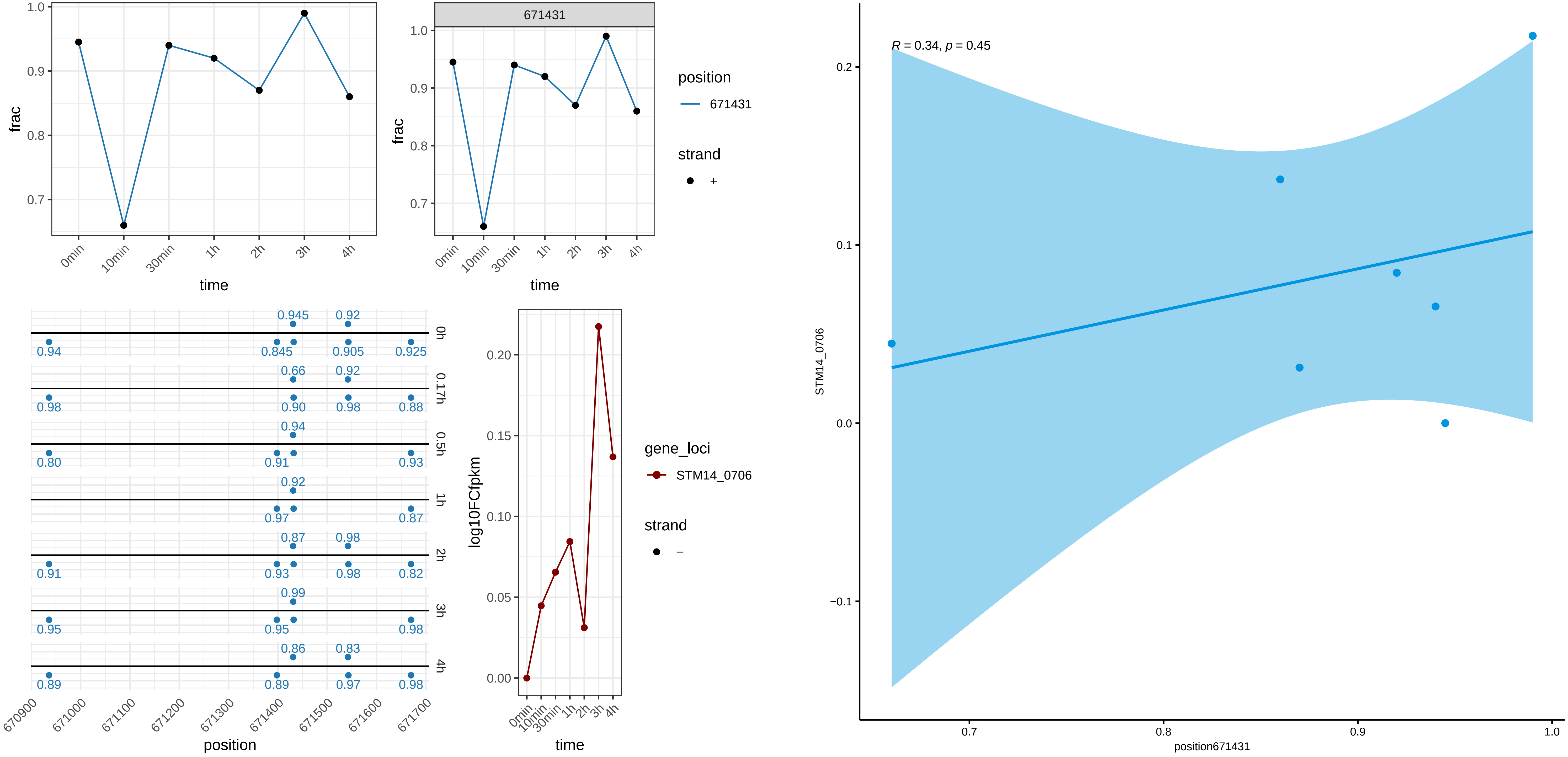

frac
time
0min
10min
30min
1h
2h
3h
4h
671431
position
671431
strand
+
0.945
0.92
0.94
0.845
0.905
0.925
0.66
0.98
0.90
0.88
0.80
0.91
0.93
0.97
0.87
0.82
0.99
0.95
0.86
0.83
0.89
0h
0.17h
0.5h
1h
2h
3h
4h
670900
671000
671100
671200
671300
671400
671500
671600
671700
log10FCfpkm
gene_loci
STM14_0706
strand
–
R = 0.34, p = 0.45
STM14_0706
position671431

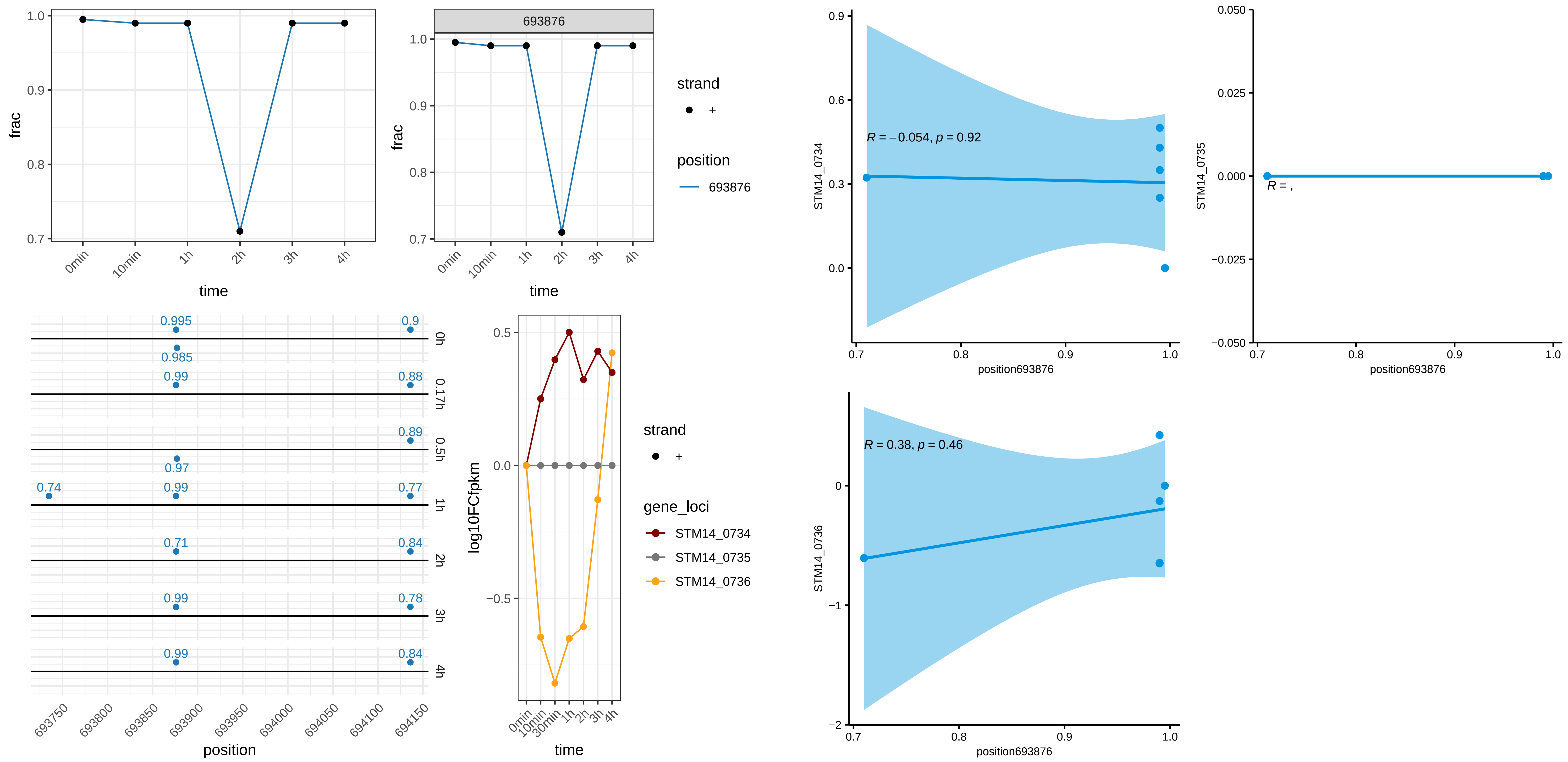

frac
time
0min
10min
1h
2h
3h
4h
693876
strand
+
position
693876
0.995
0.9
0.985
0.99
0.88
0.89
0.97
0.74
0.77
0.71
0.84
0.78
0h
0.17h
0.5h
position
log10FCfpkm
30min
gene_loci
STM14_0734
STM14_0735
STM14_0736
R = −0.054, p = 0.92
position693876
R = ,
R = 0.38, p = 0.46

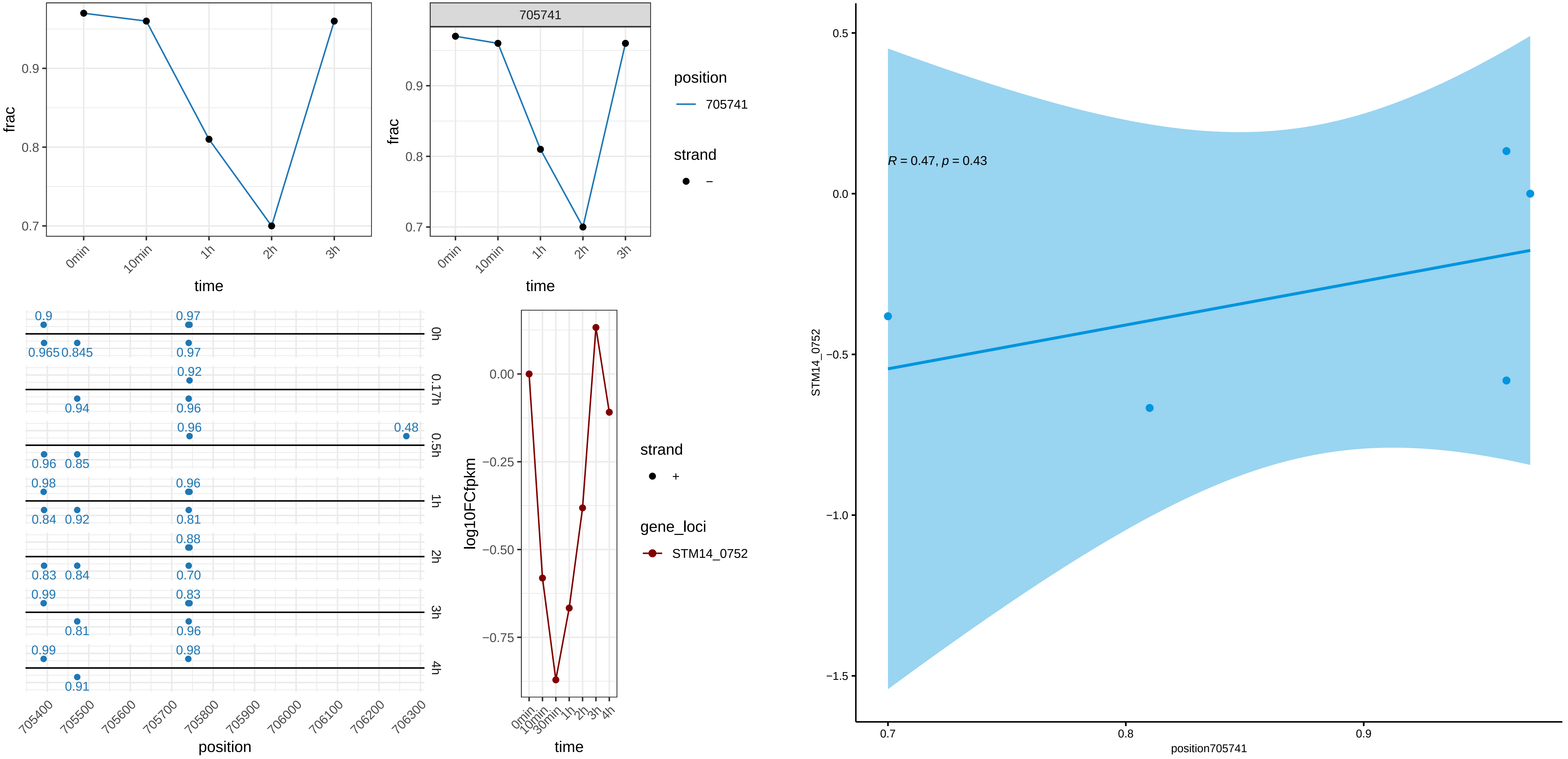

frac
time
0min
10min
1h
2h
3h
705741
position
strand
−
position
0.9
0.965
0.845
0.97
0.92
0.94
0.96
0.48
0.85
0.98
0.84
0.81
0.88
0.83
0.70
0.99
0.91
0h
0.17h
0.5h
4h
705400
705500
705600
705700
705800
705900
706000
706100
706200
706300
log10FCfpkm
30min
+
gene_loci
STM14_0752
R = 0.47, p = 0.43
position705741

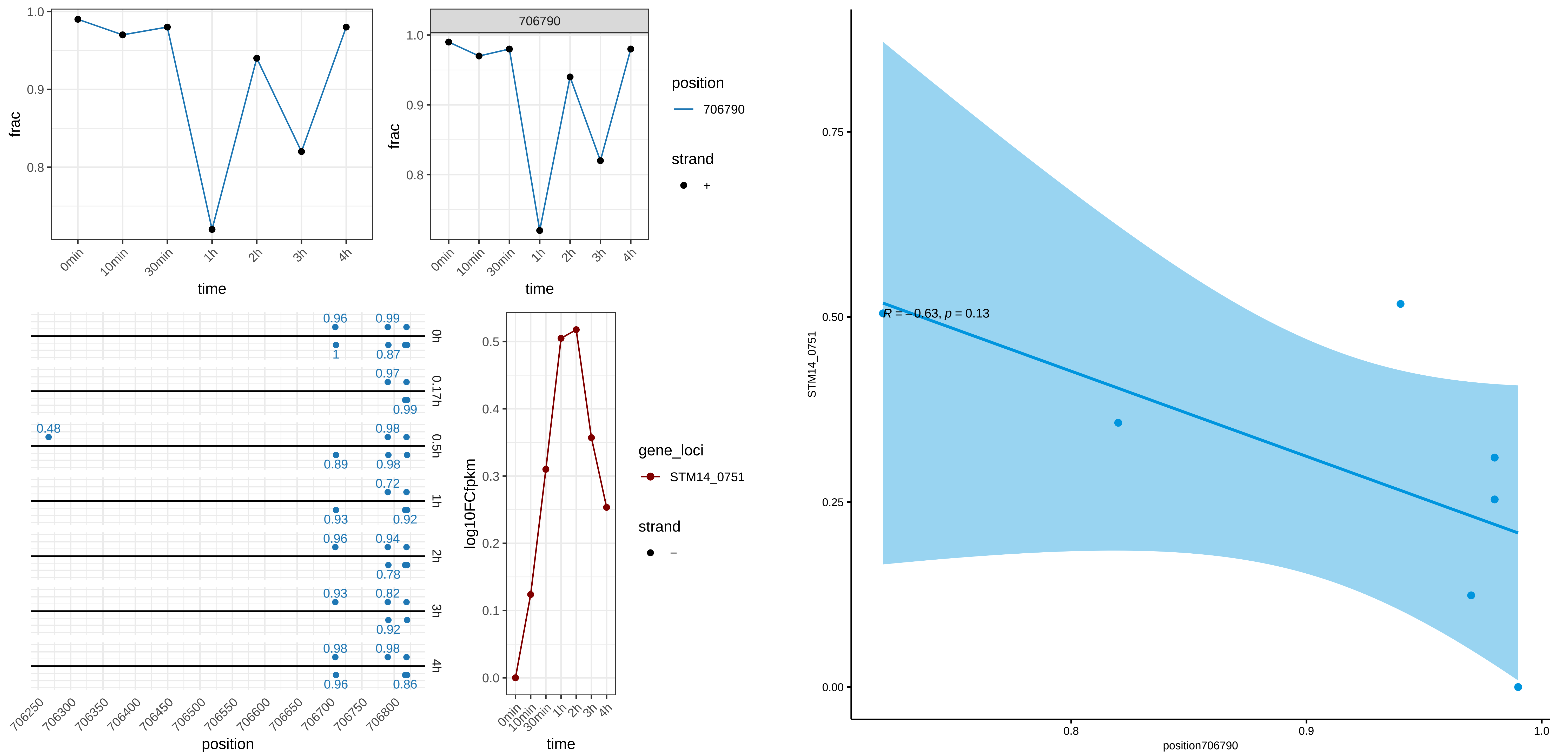

frac
time
706790
position
strand
+
log10FCfpkm
gene_loci
STM14_0751
–
R = −0.63, p = 0.13
STM14_0751
position706790

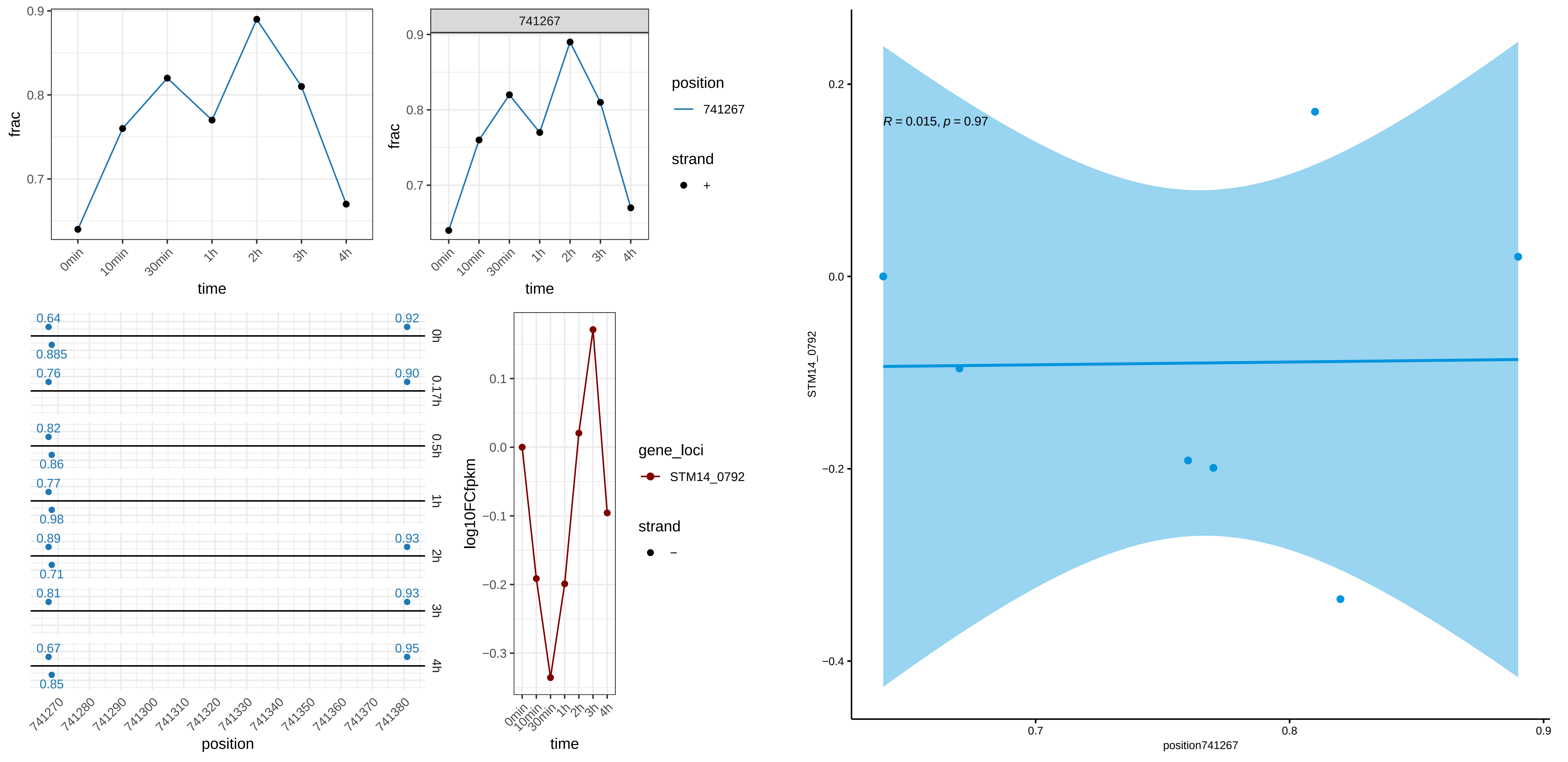

frac
time
0min
10min
30min
1h
2h
3h
4h
741267
position
741267
strand
+
position
0h
0.17h
0.5h
1h
2h
3h
4h
log10FCfpkm
gene_loci
STM14_0792
strand
–
R = 0.015, p = 0.97
STM14_0792
position741267

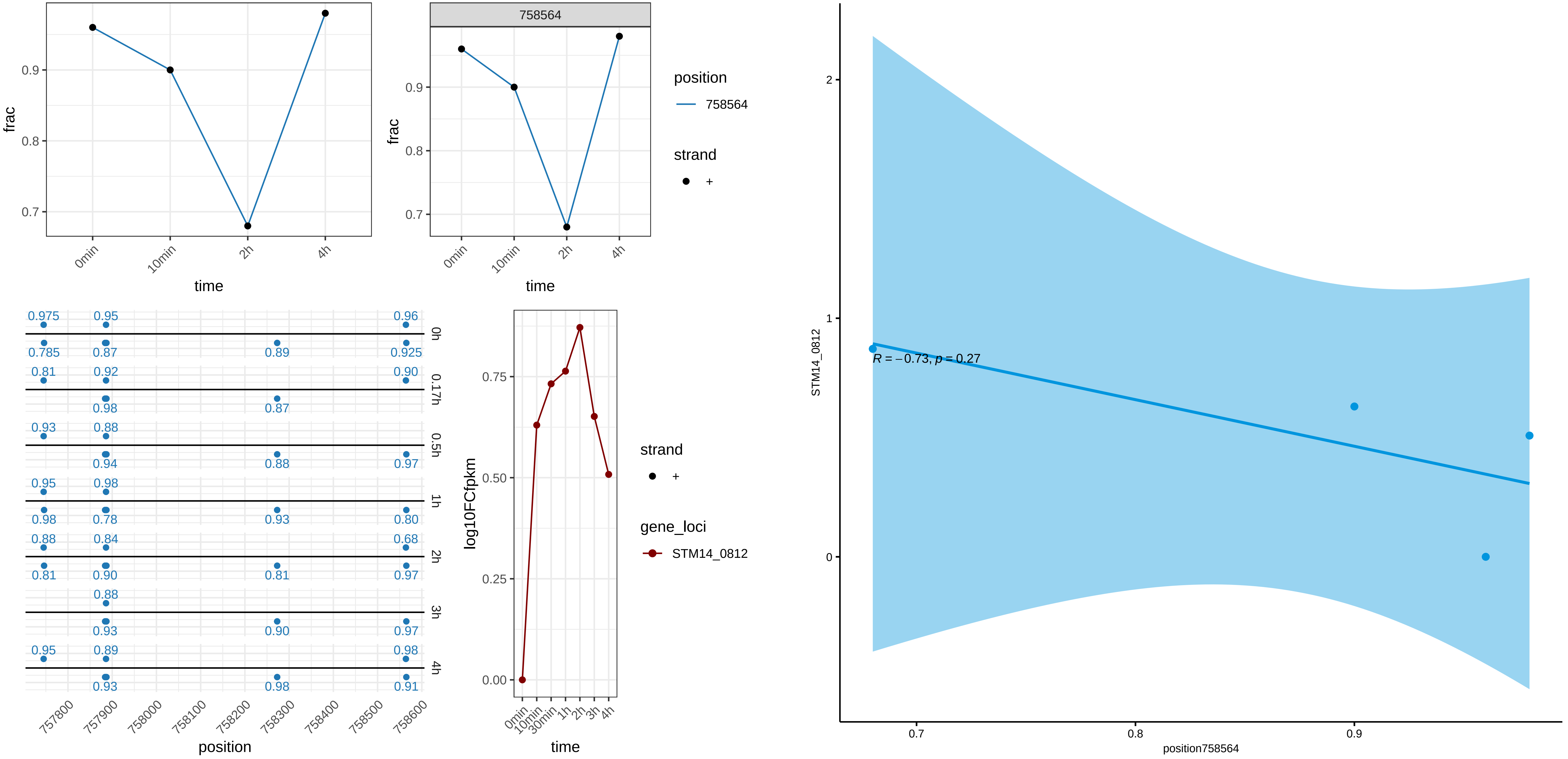

frac
time
0min
10min
2h
4h
758564
position
strand
+
log10FCfpkm
gene_loci
STM14_0812
0h
0.17h
0.5h
1h
3h
R = −0.73, p = 0.27
position758564

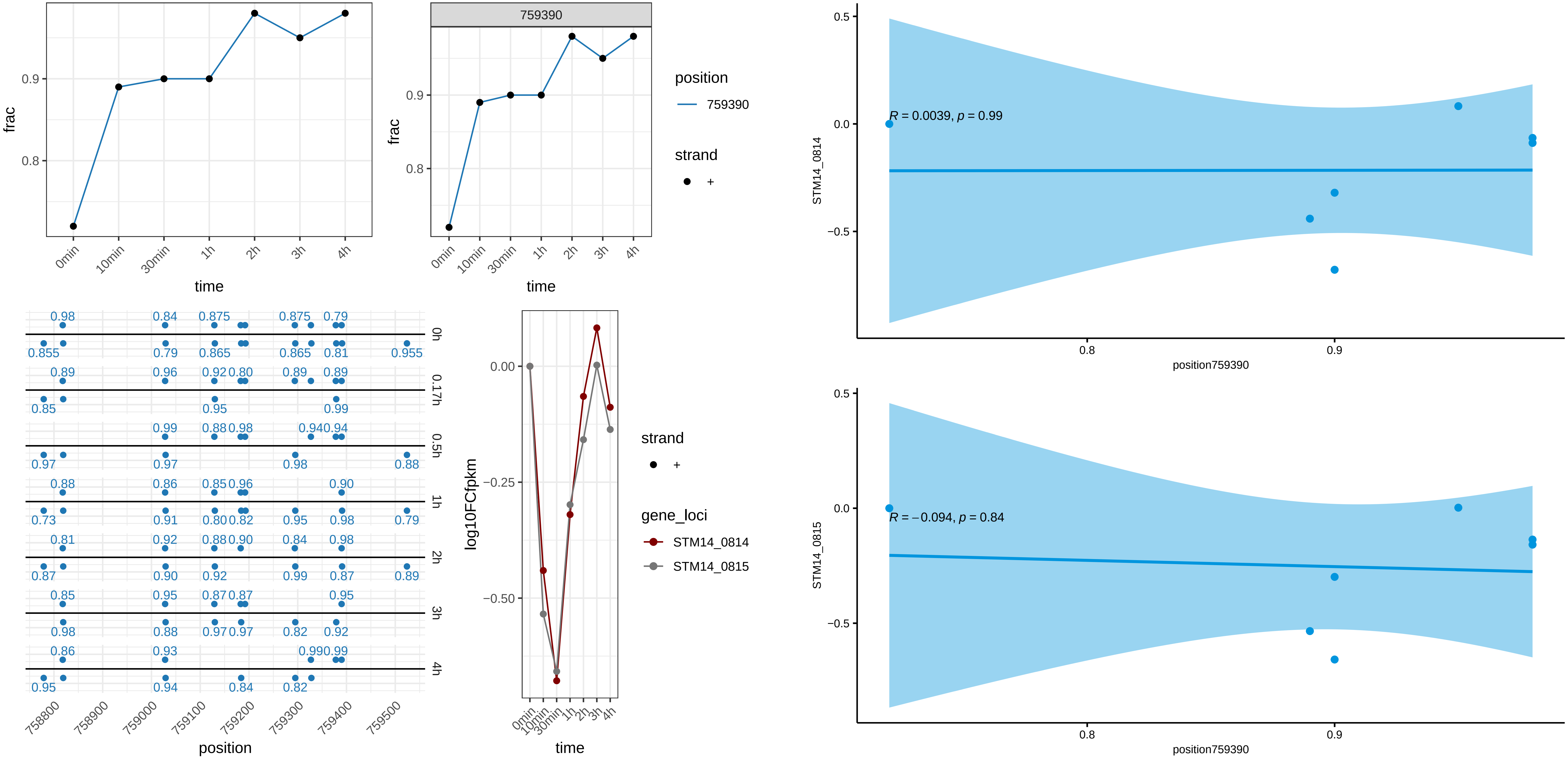

frac
time
0min
10min
30min
1h
2h
3h
4h
759390
position
759390
strand
+
position
log10FCfpkm
gene_loci
STM14_0814
STM14_0815
R = 0.0039, p = 0.99
STM14_0814
position759390
R = −0.094, p = 0.84
STM14_0815
position759390

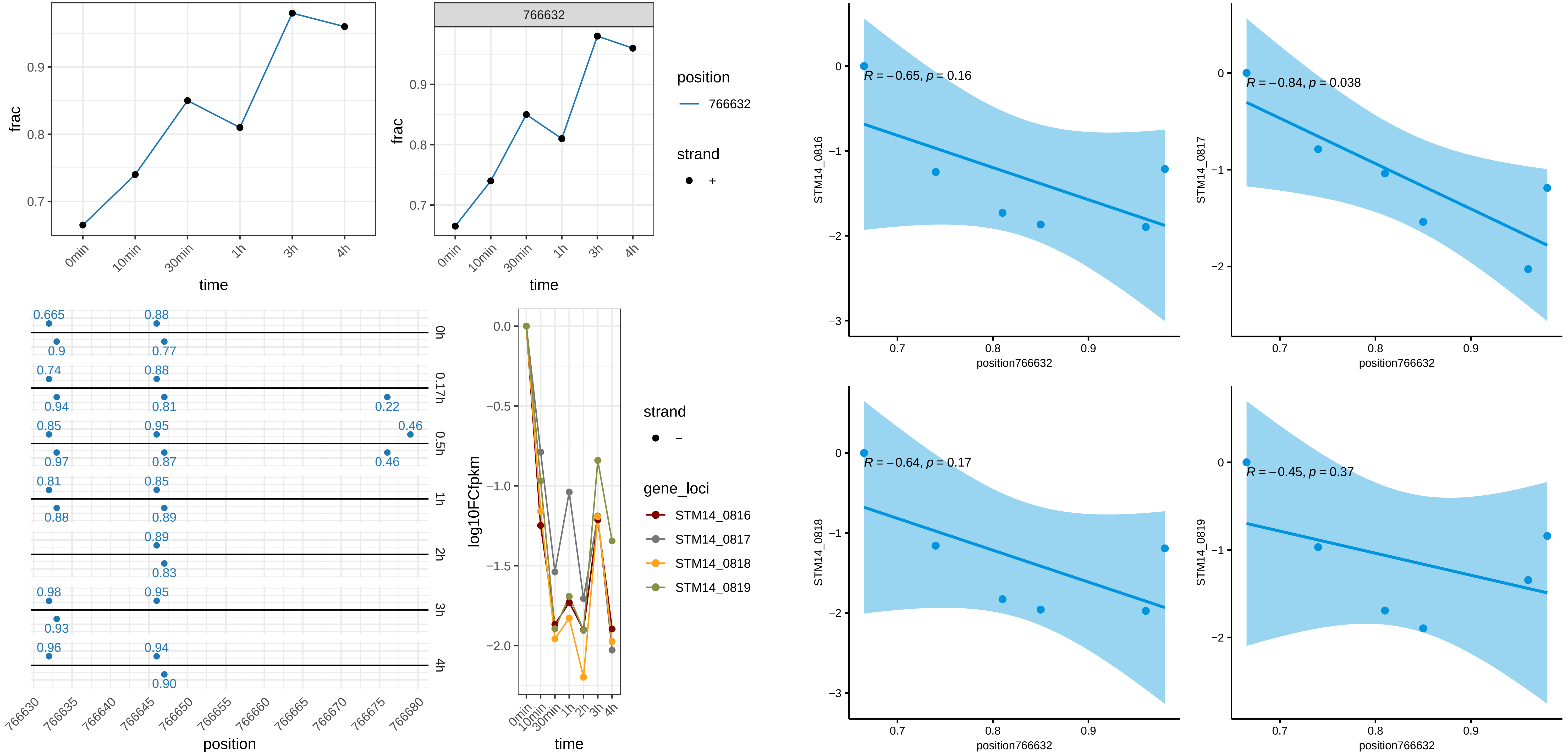

frac
time
766632
position
strand
log10FCfpkm
gene_loci
STM14_0816
STM14_0817
STM14_0818
STM14_0819
R = −0.65, p = 0.16
R = −0.84, p = 0.038
R = −0.64, p = 0.17
R = −0.45, p = 0.37
position766632

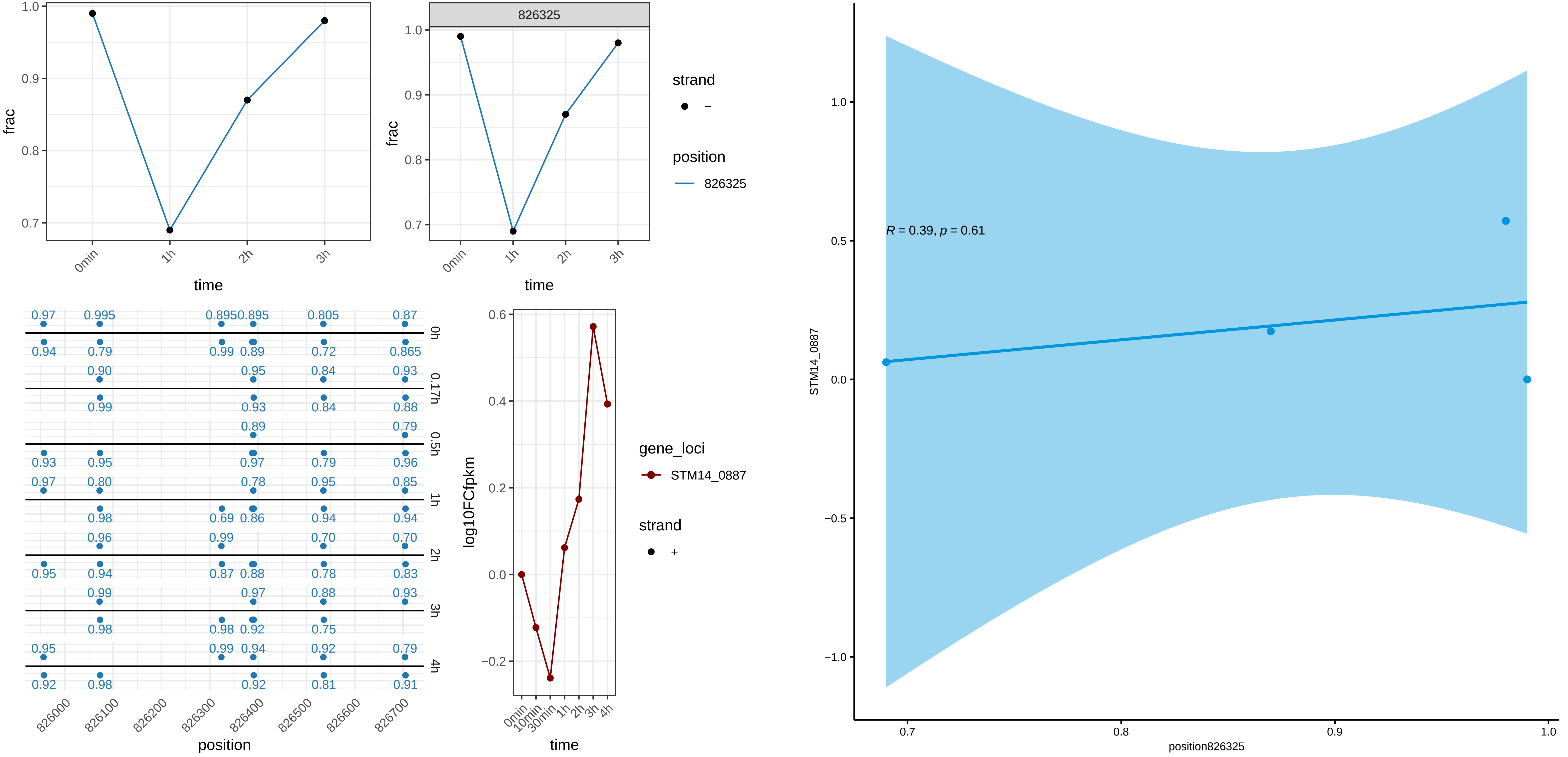

frac
time
0min
1h
2h
3h
826325
strand
–
position
826325
0.97 0.995 0.8950.895 0.805 0.87
0.94 0.79 0.99 0.89 0.72 0.865
0.90 0.95 0.84 0.93
0.99 0.93 0.84 0.88
0.89 0.79
0.93 0.95 0.97 0.79 0.96
0.97 0.80 0.78 0.95 0.85
0.98 0.69 0.86 0.94 0.94
0.96 0.99 0.70 0.70
0.95 0.94 0.87 0.88 0.78 0.83
0.99 0.97 0.88 0.93
0.98 0.98 0.92 0.75
0.95 0.99 0.94 0.92 0.79
0.92 0.98 0.92 0.81 0.91
0h
0.17h
0.5h
1h
2h
3h
4h
826000
826100
826200
826300
826400
826500
826600
826700
position
log10FCfpkm
0min
10min
30min
1h
2h
3h
4h
time
gene_loci
STM14_0887
strand
+
R = 0.39, p = 0.61
STM14_0887
position826325

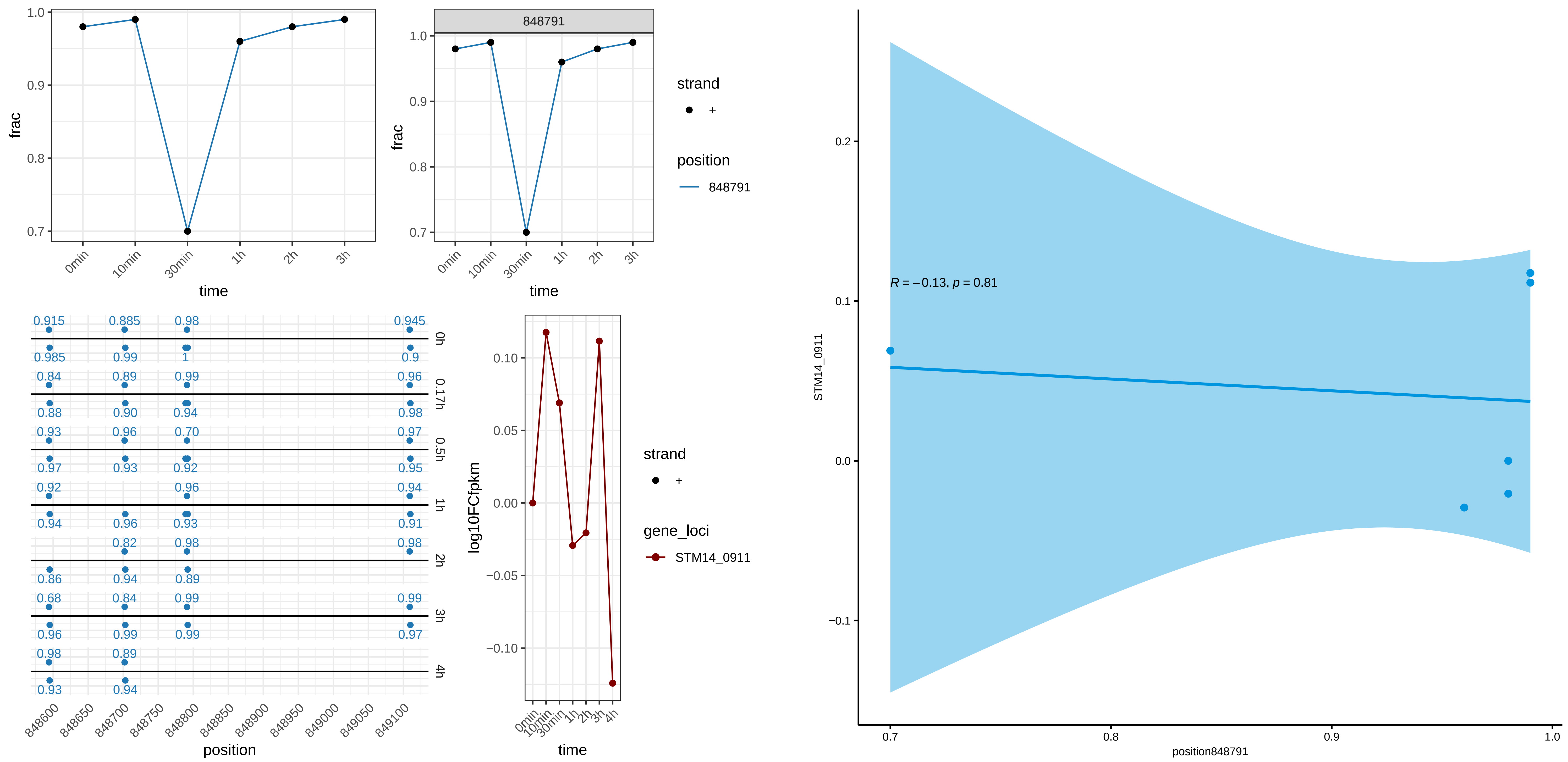

frac
time
848791
strand
+
position
848791
R = −0.13, p = 0.81
STM14_0911
position848791
log10FCfpkm
gene_loci
STM14_0911
0h
0.17h
0.5h
1h
2h
3h
4h
position

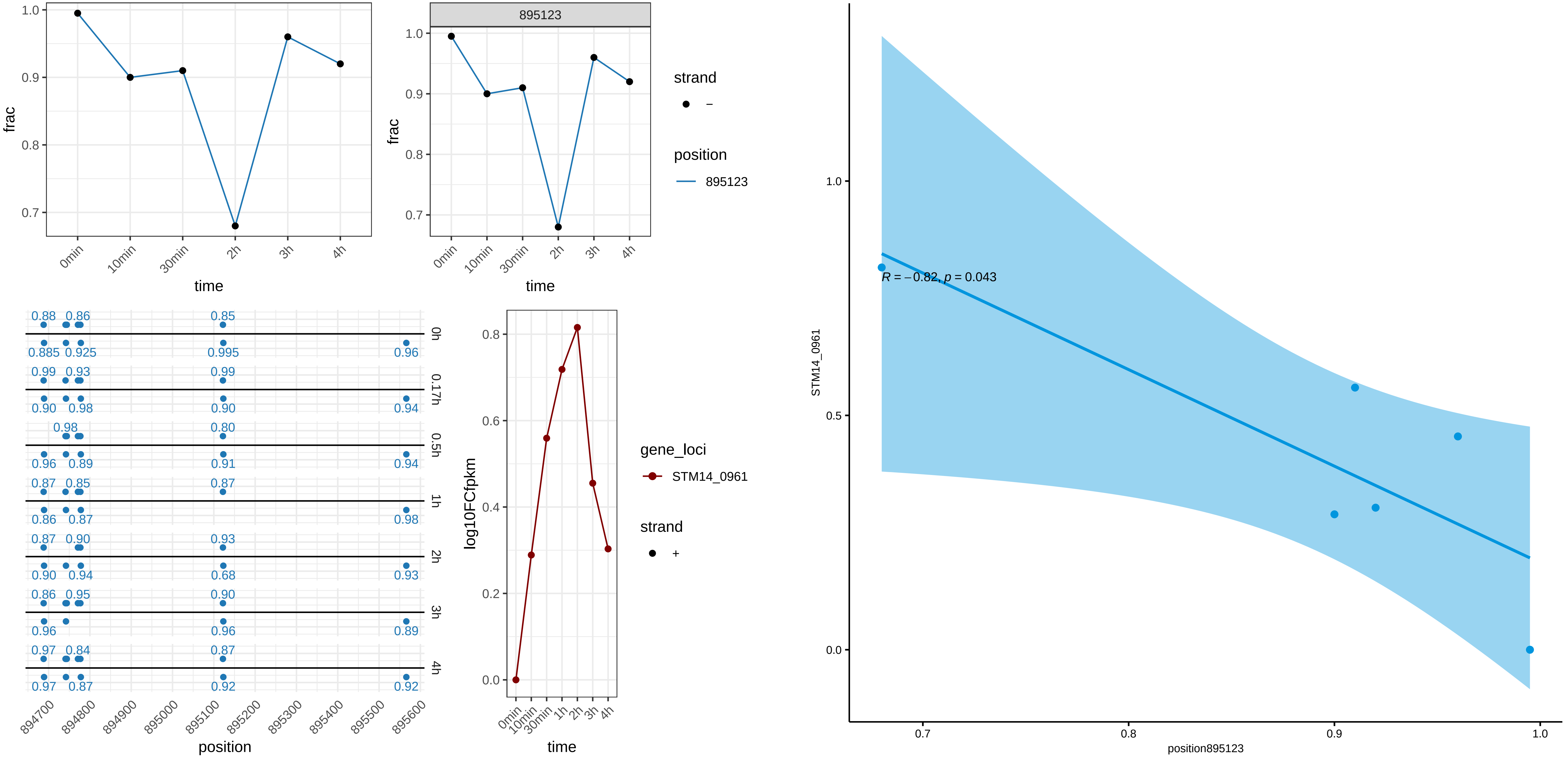
frac
time
0min
10min
30min
2h
3h
4h
895123
strand
–
position
895123
0.88 0.86
0.85
0.885 0.925
0.995
0.96
0h
0.99 0.93
0.99
0.90 0.98
0.90
0.94
0.17h
0.98
0.80
0.96 0.89
0.91
0.94
0.5h
0.87 0.85
0.87
0.86 0.87
0.98
1h
0.87 0.90
0.93
0.90 0.94
0.68
0.93
2h
0.86 0.95
0.90
0.96
0.96
0.89
3h
0.97 0.84
0.87
0.97 0.87
0.92
0.92
4h
894700
894800
894900
895000
895100
895200
895300
895400
895500
895600
position
log10FCfpkm
1h
gene_loci
STM14_0961
strand
+
R = –0.82, p = 0.043
STM14_0961
position895123

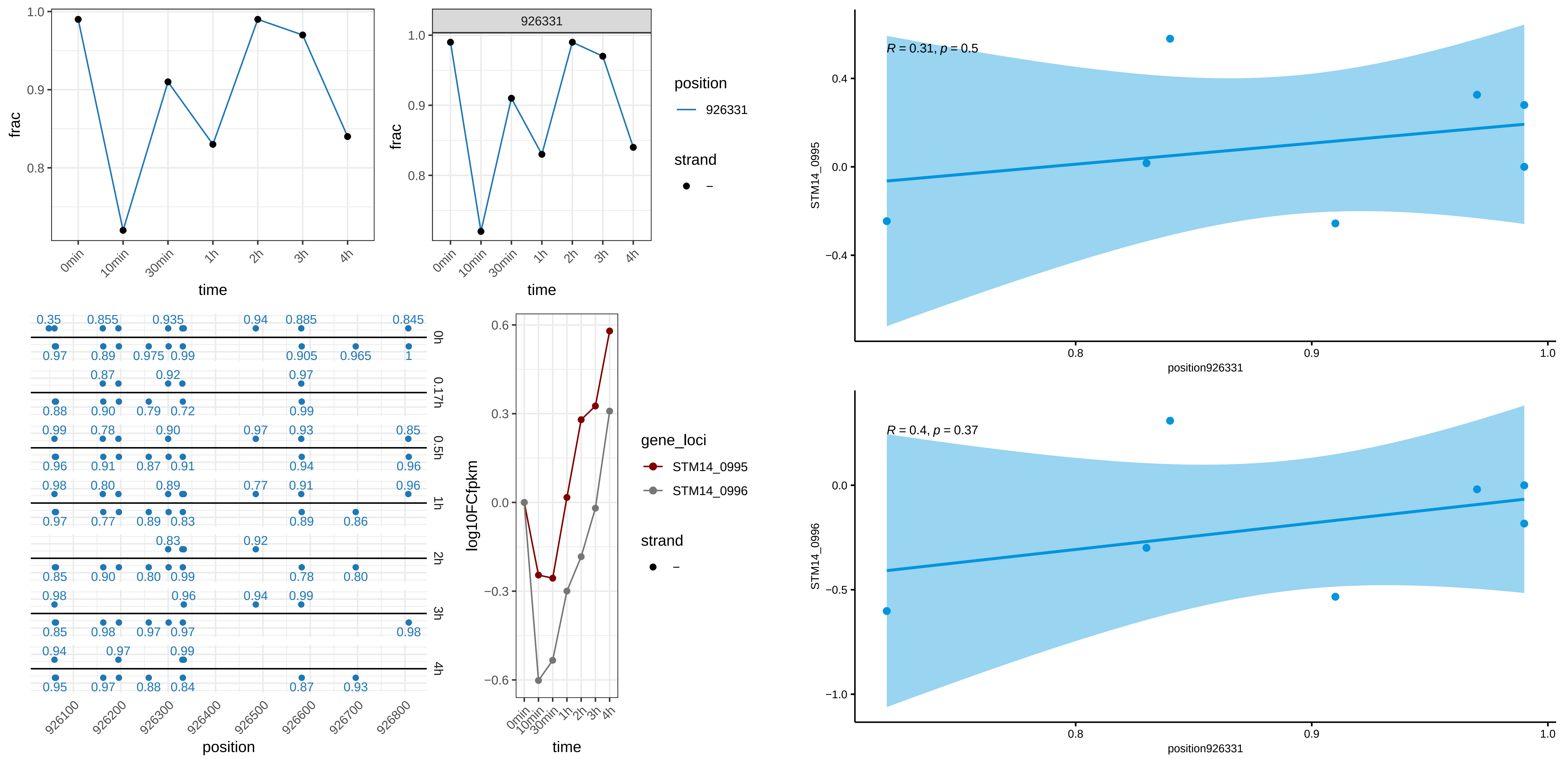

frac
time
0min
10min
30min
1h
2h
3h
4h
926331
position
926331
strand
–
R = 0.31, p = 0.5
STM14_0995
position926331
R = 0.4, p = 0.37
STM14_0996
position926331
0h
0.17h
0.5h
1h
2h
3h
4h
position
log10FCfpkm
gene_loci
STM14_0995
STM14_0996
strand
–
time

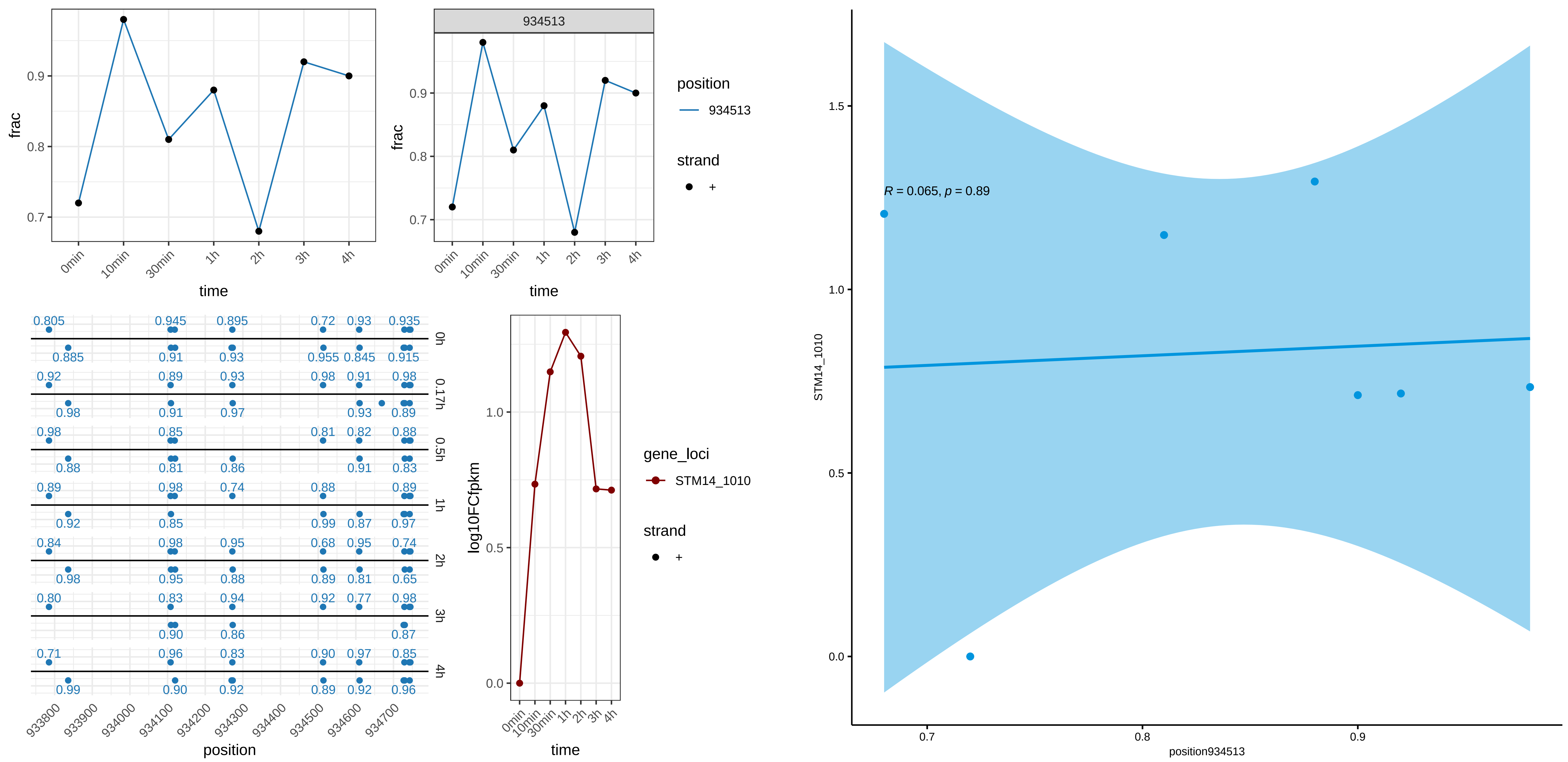

934513
frac
time
position
934513
strand
+
log10FCfpkm
gene_loci
STM14_1010
position934513
R = 0.065, p = 0.89

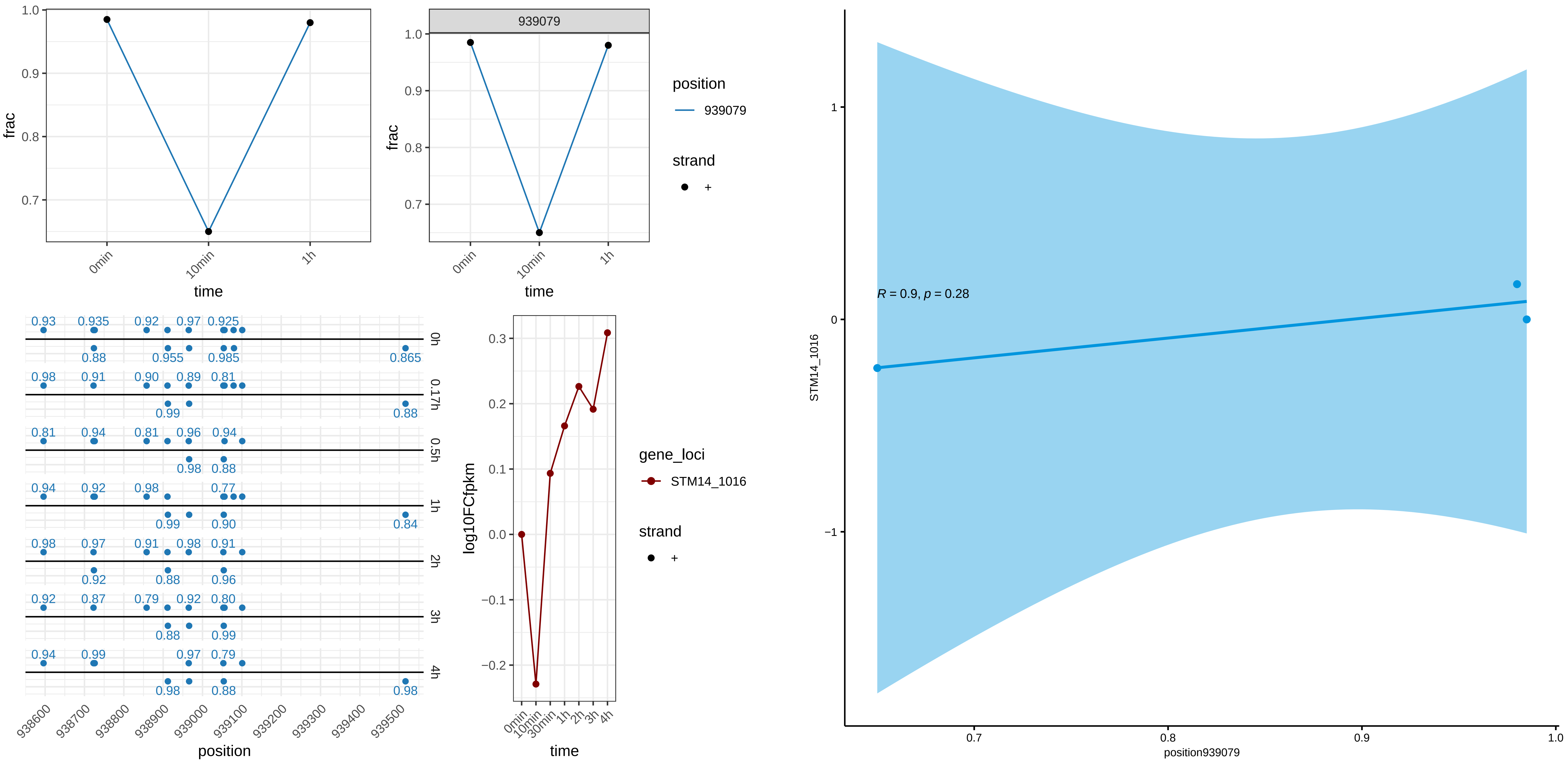

frac
time
0min
10min
1h
939079
position
strand
+
0h
0.17h
0.5h
2h
3h
4h
position
log10FCfpkm
30min
gene_loci
STM14_1016
R = 0.9, p = 0.28
position939079

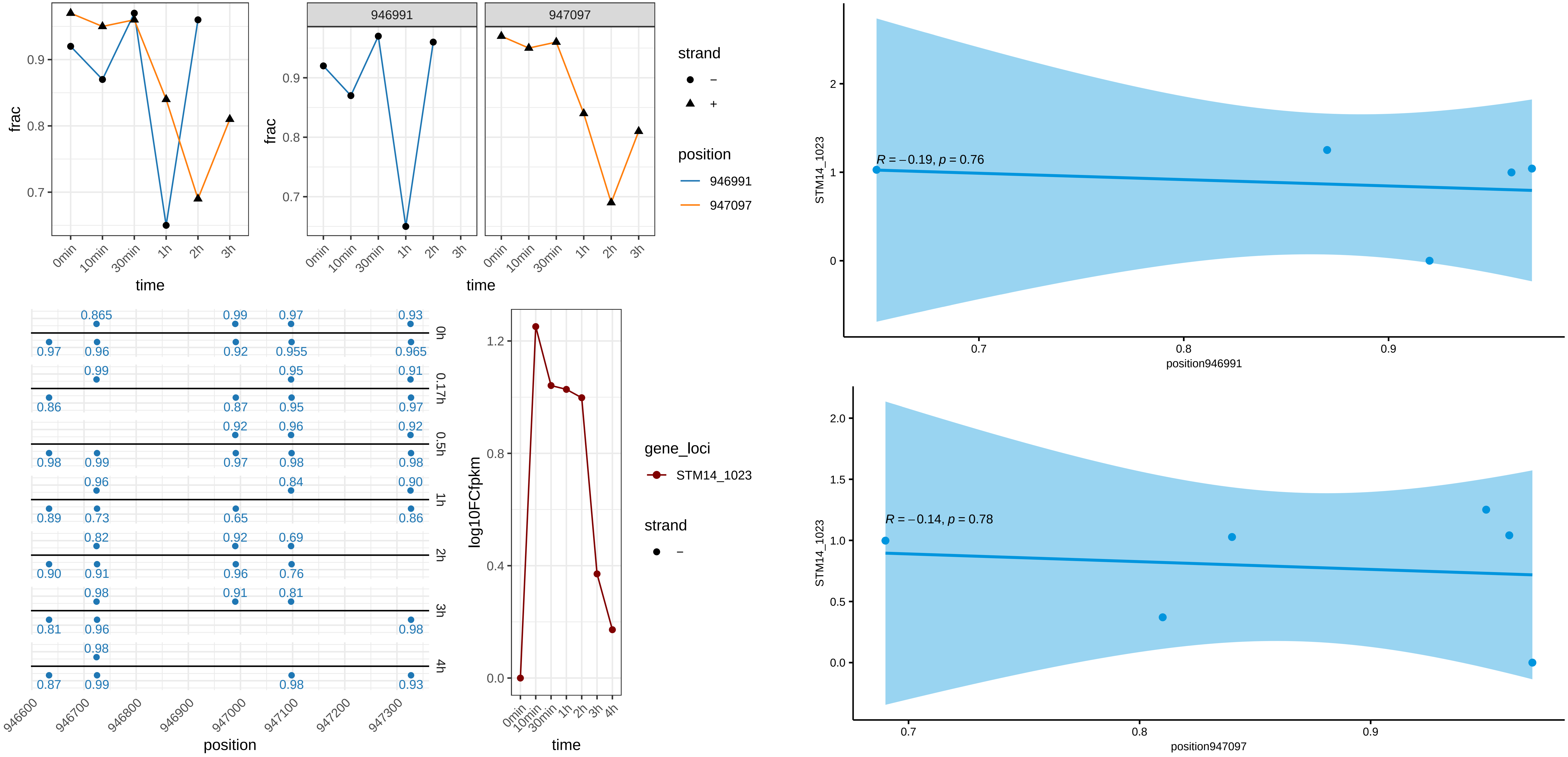

frac
time
0min
10min
30min
1h
2h
3h
946991
947097
strand
–
+
position
946991
947097
0h
0.17h
0.5h
1h
2h
3h
4h
position
946600
946700
946800
946900
947000
947100
947200
947300
log10FCfpkm
gene_loci
STM14_1023
R = –0.19, p = 0.76
position946991
R = –0.14, p = 0.78
position947097
STM14_1023

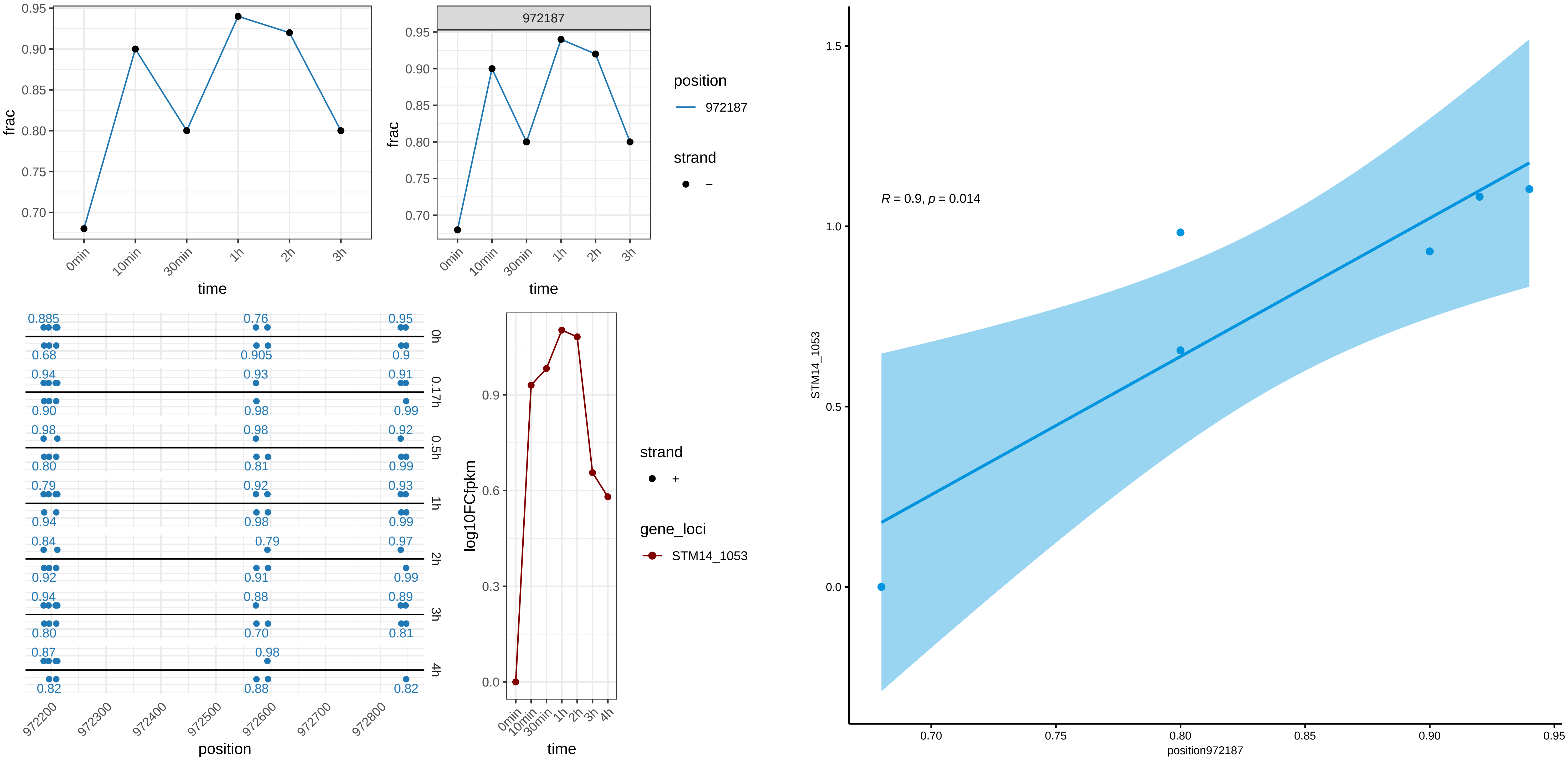

frac
time
0min
10min
30min
1h
2h
3h
972187
position
972187
strand
–
0.885
0.76
0.95
0.68
0.905
0.9
0h
0.94
0.93
0.91
0.90
0.98
0.99
0.17h
0.98
0.98
0.92
0.80
0.81
0.99
0.5h
0.79
0.92
0.93
0.94
0.98
0.99
1h
0.84
0.79
0.97
0.92
0.91
0.99
2h
0.94
0.88
0.89
0.80
0.70
0.81
3h
0.87
0.98
0.82
0.88
0.82
4h
position
log10FCfpkm
time
4h
strand
+
gene_loci
STM14_1053
R = 0.9, p = 0.014
STM14_1053
position972187

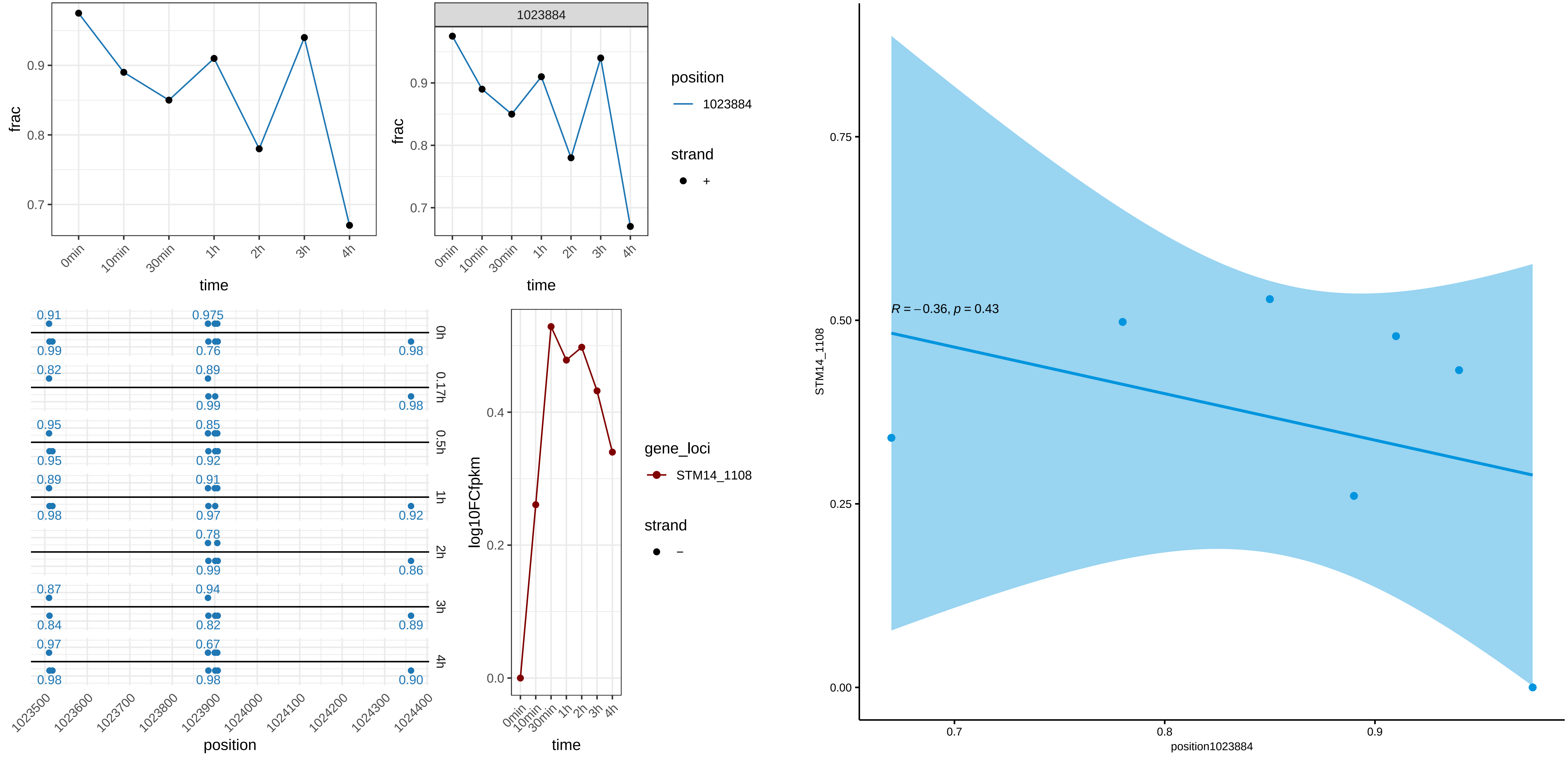

frac
time
0min
10min
30min
1h
2h
3h
4h
1023884
position
strand
+
0.91
0.975
0.99
0.76
0.98
0.82
0.89
0.95
0.85
0.92
0.97
0.78
0.86
0.87
0.94
0.84
0.82
0.67
0.90
0h
0.17h
0.5h
position
log10FCfpkm
gene_loci
STM14_1108
–
R = –0.36, p = 0.43
STM14_1108
position1023884

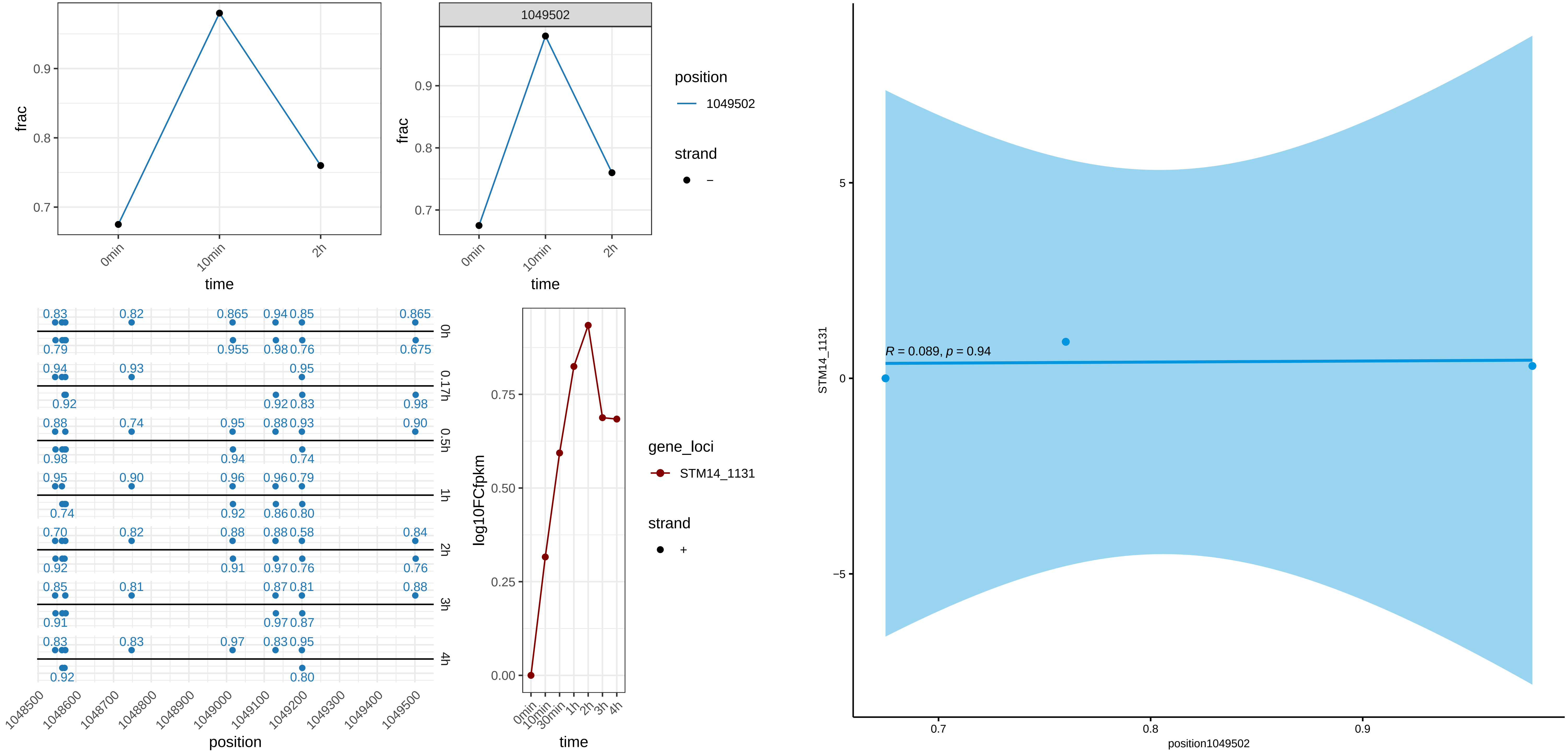

frac
time
0min
10min
2h
1049502
position
strand
−
+
0h
0.17h
0.5h
1h
2h
3h
4h
log10FCfpkm
30min
gene_loci
STM14_1131
R = 0.089, p = 0.94
position1049502

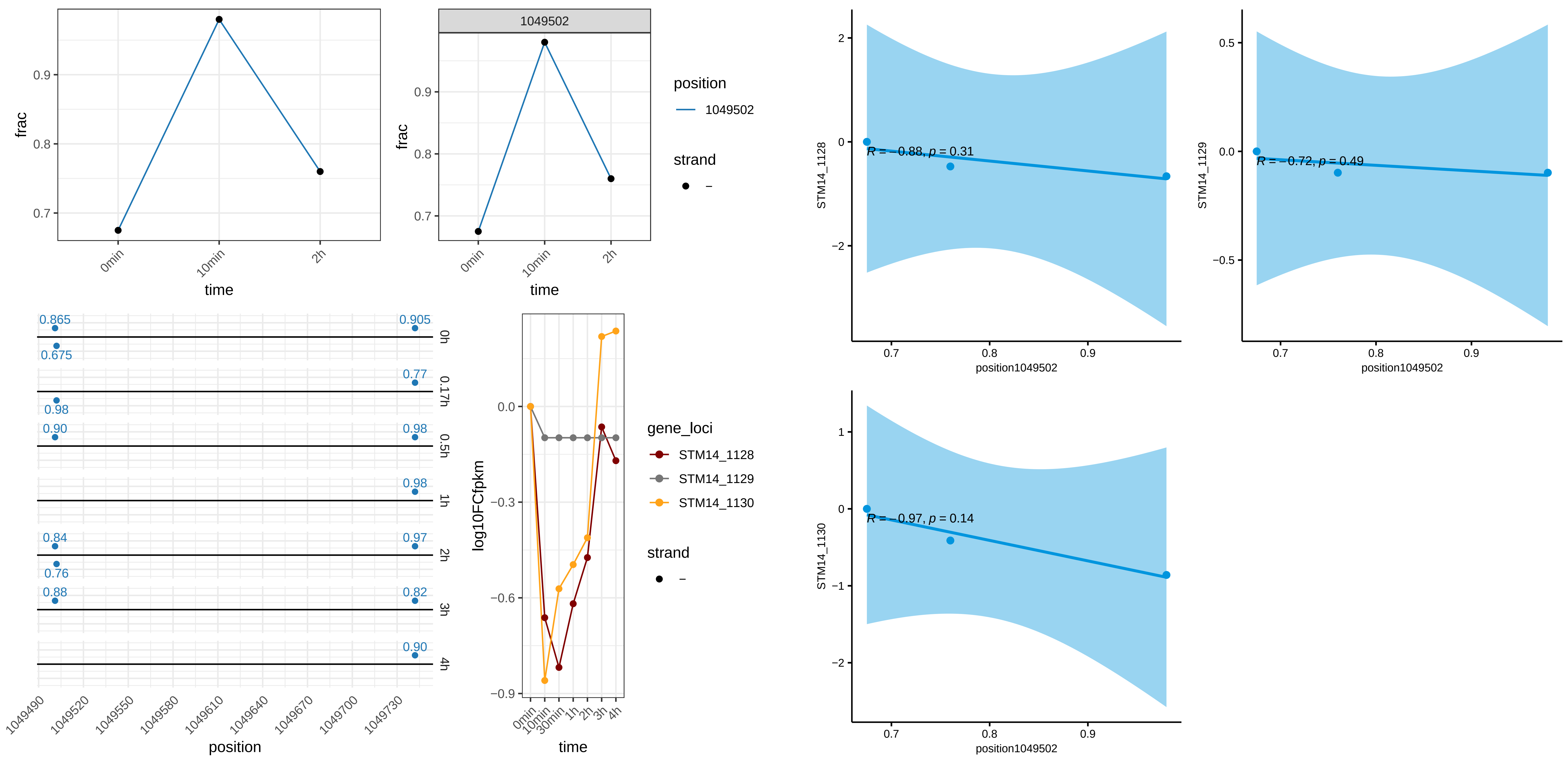

frac
time
0min
10min
2h
1049502
position
strand
gene_loci
STM14_1128
STM14_1129
STM14_1130
log10FCfpkm
0h
0.17h
0.5h
1h
2h
3h
4h
R = −0.88, p = 0.31
R = −0.72, p = 0.49
R = −0.97, p = 0.14
position1049502

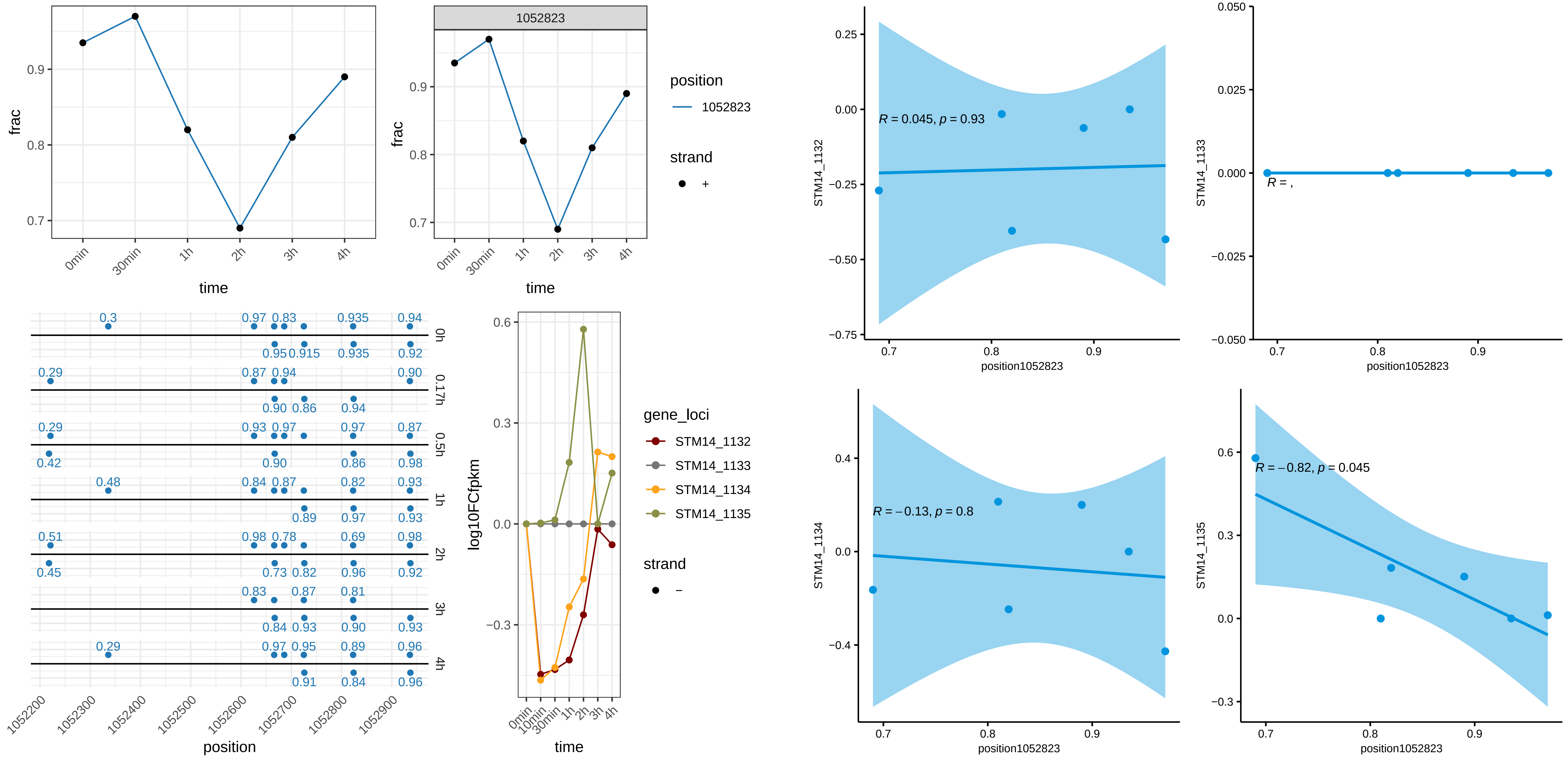

frac
time
0min
30min
1h
2h
3h
4h
1052823
position
1052823
strand
+
position
0h
0.17h
0.5h
1h
2h
3h
4h
log10FCfpkm
gene_loci
STM14_1132
STM14_1133
STM14_1134
STM14_1135
strand
–
0min
10min
30min
STM14_1132
position1052823
R = 0.045, p = 0.93
STM14_1133
R = ,
STM14_1134
R = –0.13, p = 0.8
STM14_1135
R = –0.82, p = 0.045

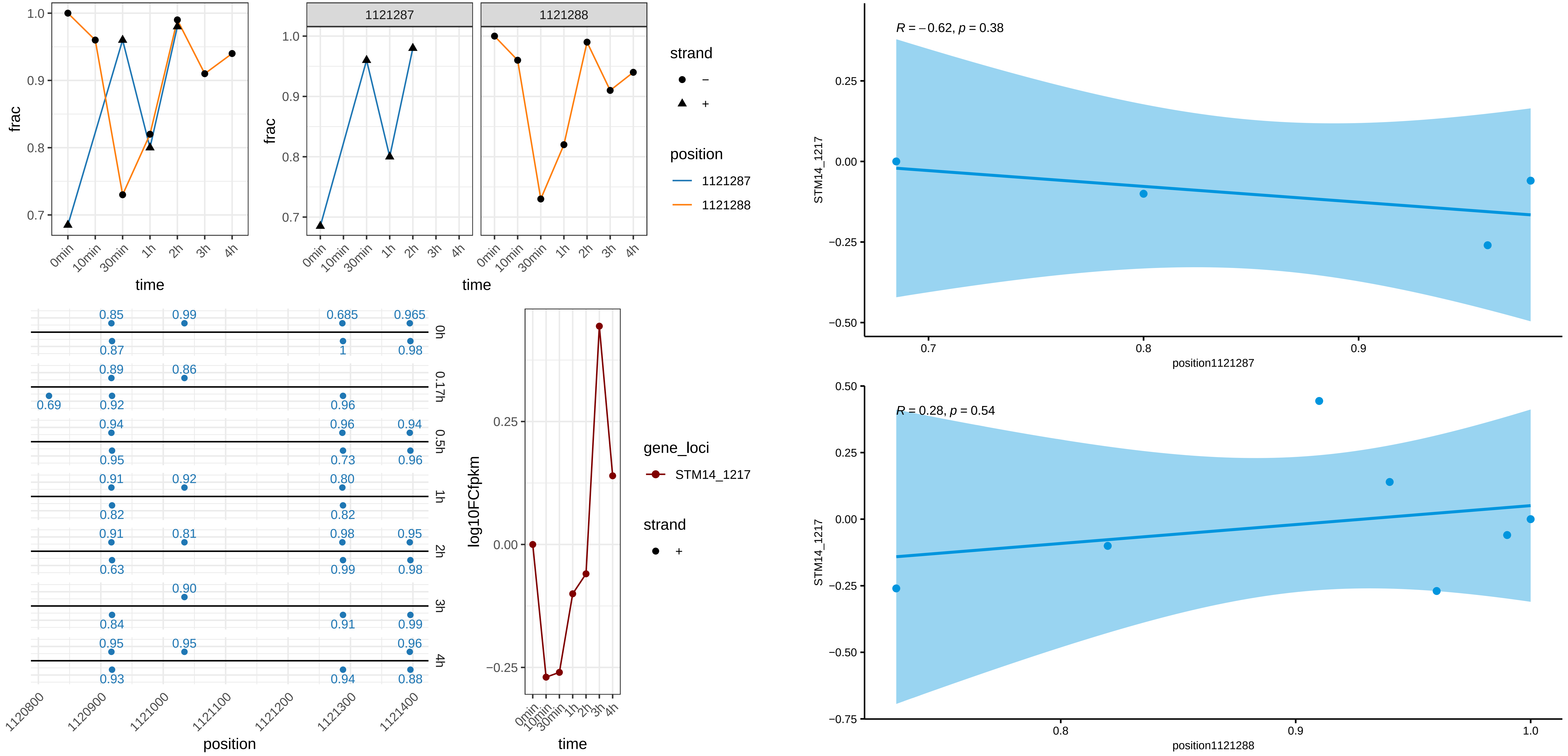

frac
time
0min
10min
30min
1h
2h
3h
4h
1121287
1121288
strand
−
+
position
1121287
1121288
0h
0.17h
0.5h
1h
2h
3h
4h
position
log10FCfpkm
gene_loci
STM14_1217
strand
+
R = −0.62, p = 0.38
STM14_1217
position1121287
R = 0.28, p = 0.54
STM14_1217
position1121288

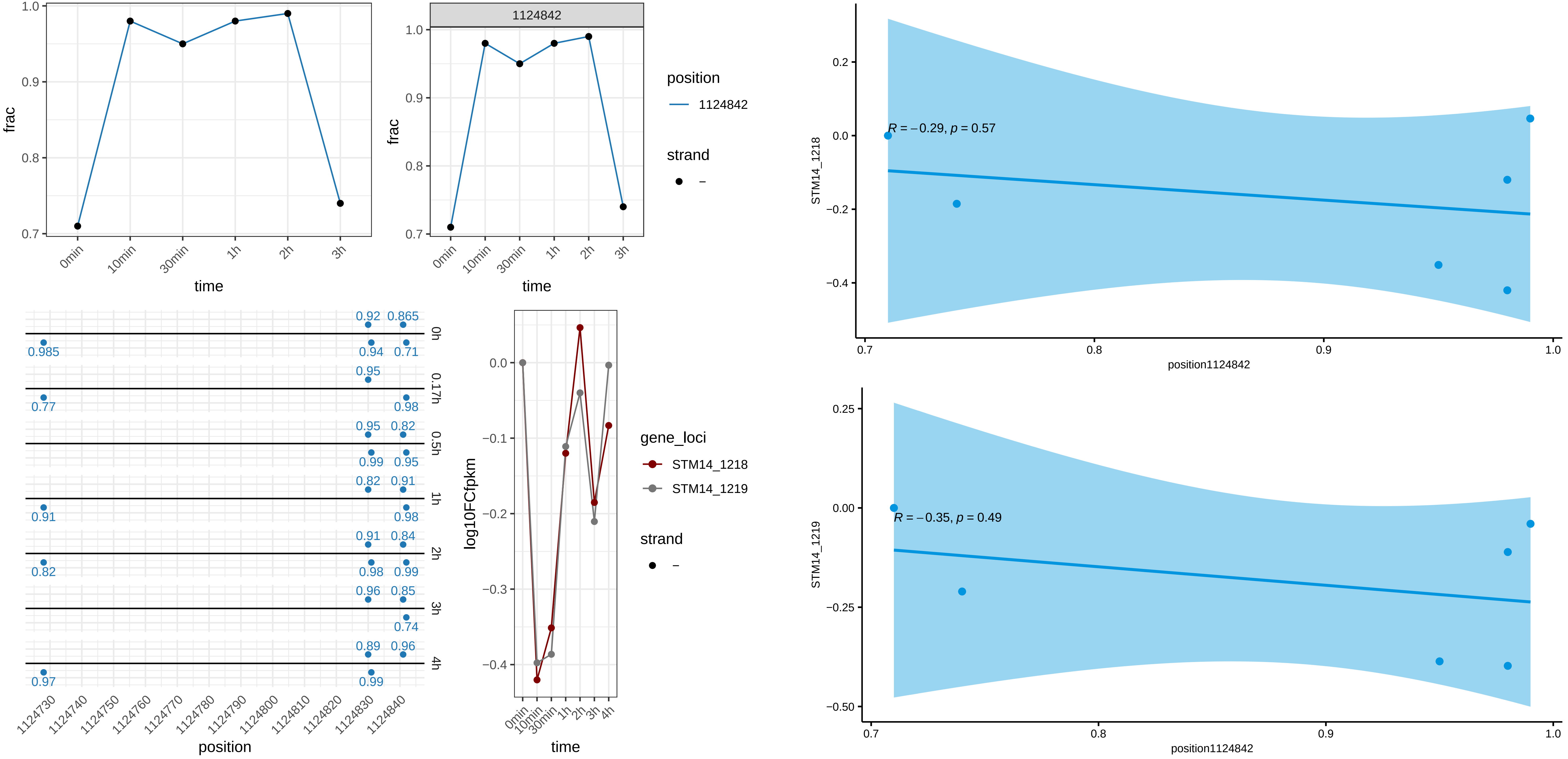

frac
time
0min
10min
30min
1h
2h
3h
1124842
position
1124842
strand
–
log10FCfpkm
4h
gene_loci
STM14_1218
STM14_1219
0h
0.17h
0.5h
position
R = −0.29, p = 0.57
STM14_1218
position1124842
R = −0.35, p = 0.49
STM14_1219
position1124842

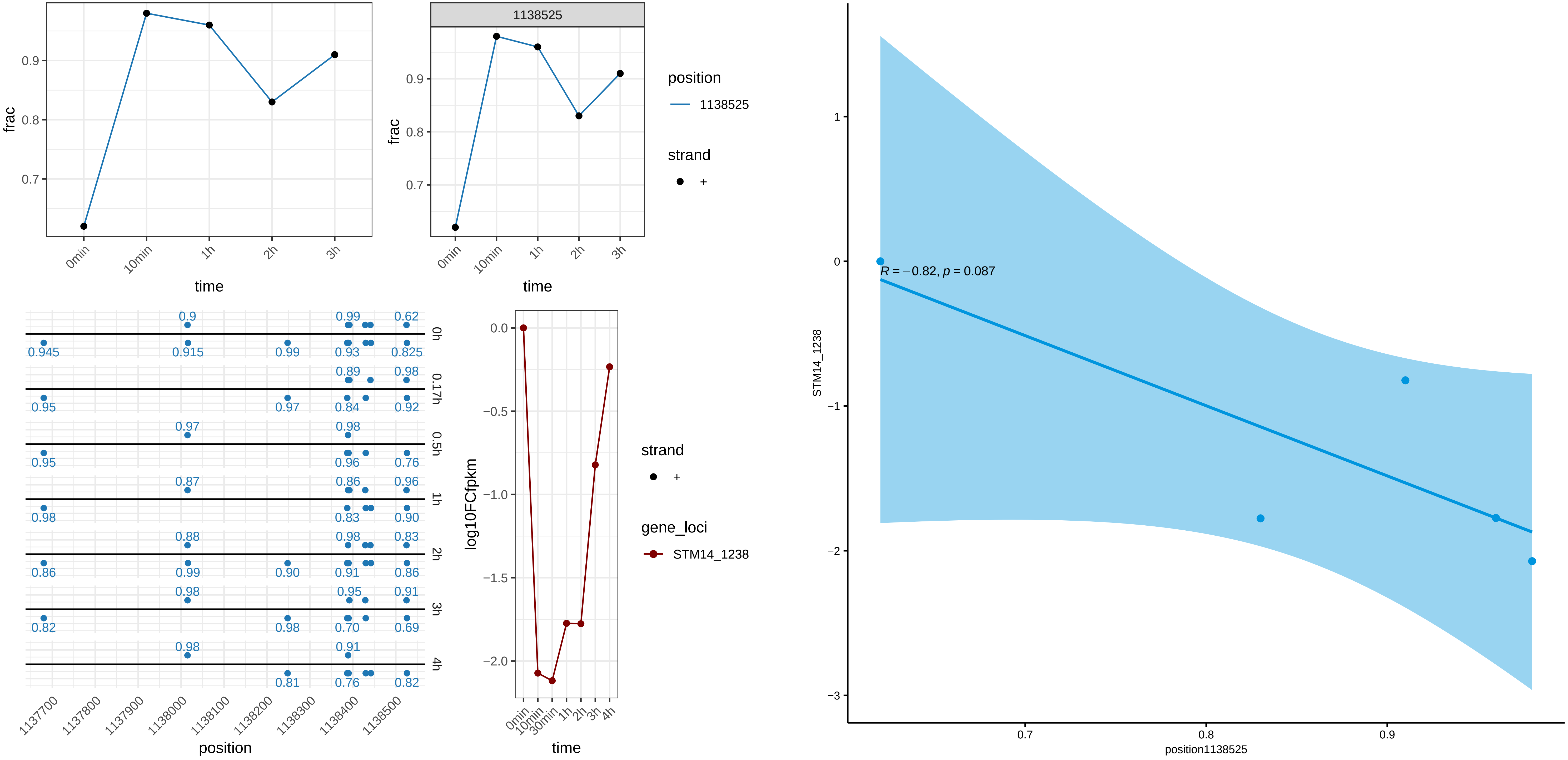

frac
time
0min
10min
1h
2h
3h
1138525
position
1138525
strand
+
position
log10FCfpkm
30min
4h
gene_loci
STM14_1238
0h
0.17h
0.5h
STM14_1238
position1138525
R = −0.82, p = 0.087

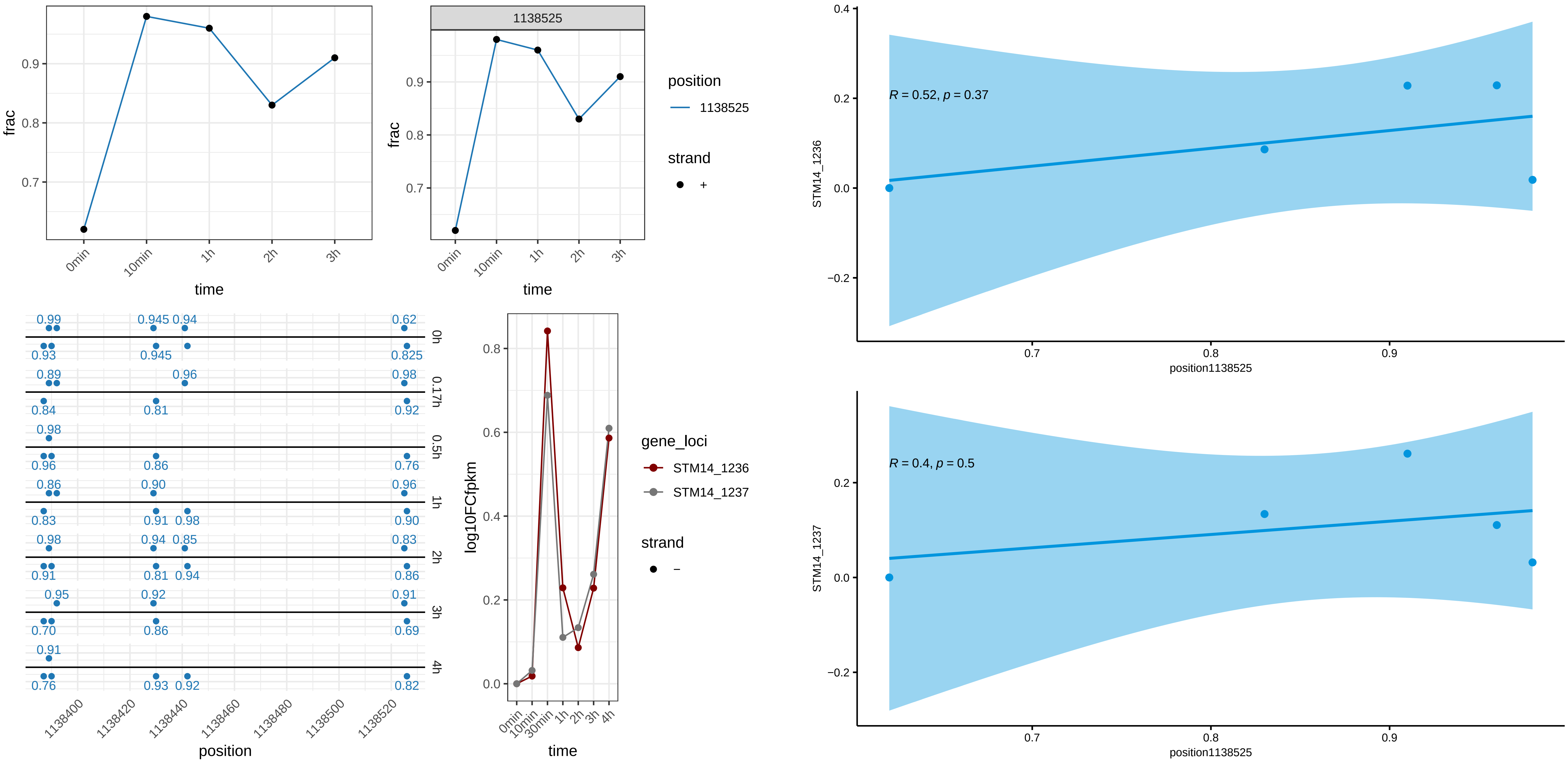

frac
time
0min
10min
1h
2h
3h
1138525
position
strand
+
0.9
0.8
0.7
0h
0.17h
0.5h
4h
0.99
0.945
0.94
0.62
0.93
0.825
0.89
0.96
0.98
0.84
0.81
0.92
0.86
0.76
0.90
0.83
0.91
0.85
0.95
0.70
0.69
0.82
1138400
1138420
1138440
1138460
1138480
1138500
1138520
log10FCfpkm
30min
gene_loci
STM14_1236
STM14_1237
–
R = 0.52, p = 0.37
R = 0.4, p = 0.5
position1138525
0.4
0.2
0.0
−0.2

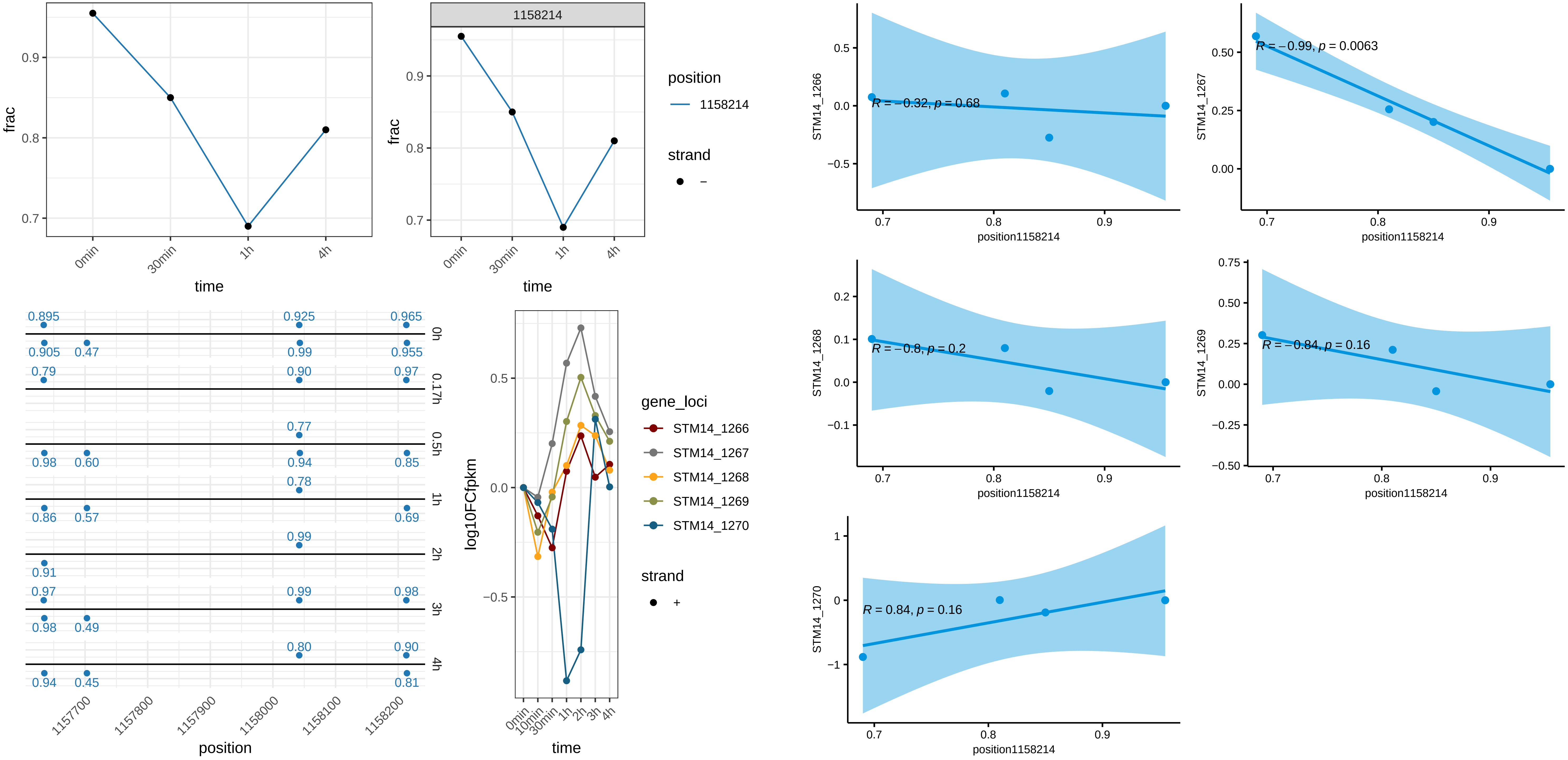

frac
0.9
0.8
0.7
0min
30min
1h
4h
time
1158214
position
1158214
strand
–
0.895
0.925
0.965
0.905
0.47
0.99
0.955
0h
0.79
0.90
0.97
0.17h
0.77
0.98
0.60
0.94
0.85
0.5h
0.78
0.86
0.57
0.69
1h
0.99
0.91
2h
0.97
0.99
0.98
0.98
0.49
3h
0.80
0.90
0.94
0.45
0.81
4h
1157700
1157800
1157900
1158000
1158100
1158200
position
log10FCfpkm
0.5
0.0
−0.5
0min
10min
30min
1h
2h
3h
4h
time
gene_loci
STM14_1266
STM14_1267
STM14_1268
STM14_1269
STM14_1270
strand
+
STM14_1266
R = −0.32, p = 0.68
position1158214
STM14_1267
R = −0.99, p = 0.0063
position1158214
STM14_1268
R = −0.8, p = 0.2
position1158214
STM14_1269
R = −0.84, p = 0.16
position1158214
STM14_1270
R = 0.84, p = 0.16
position1158214

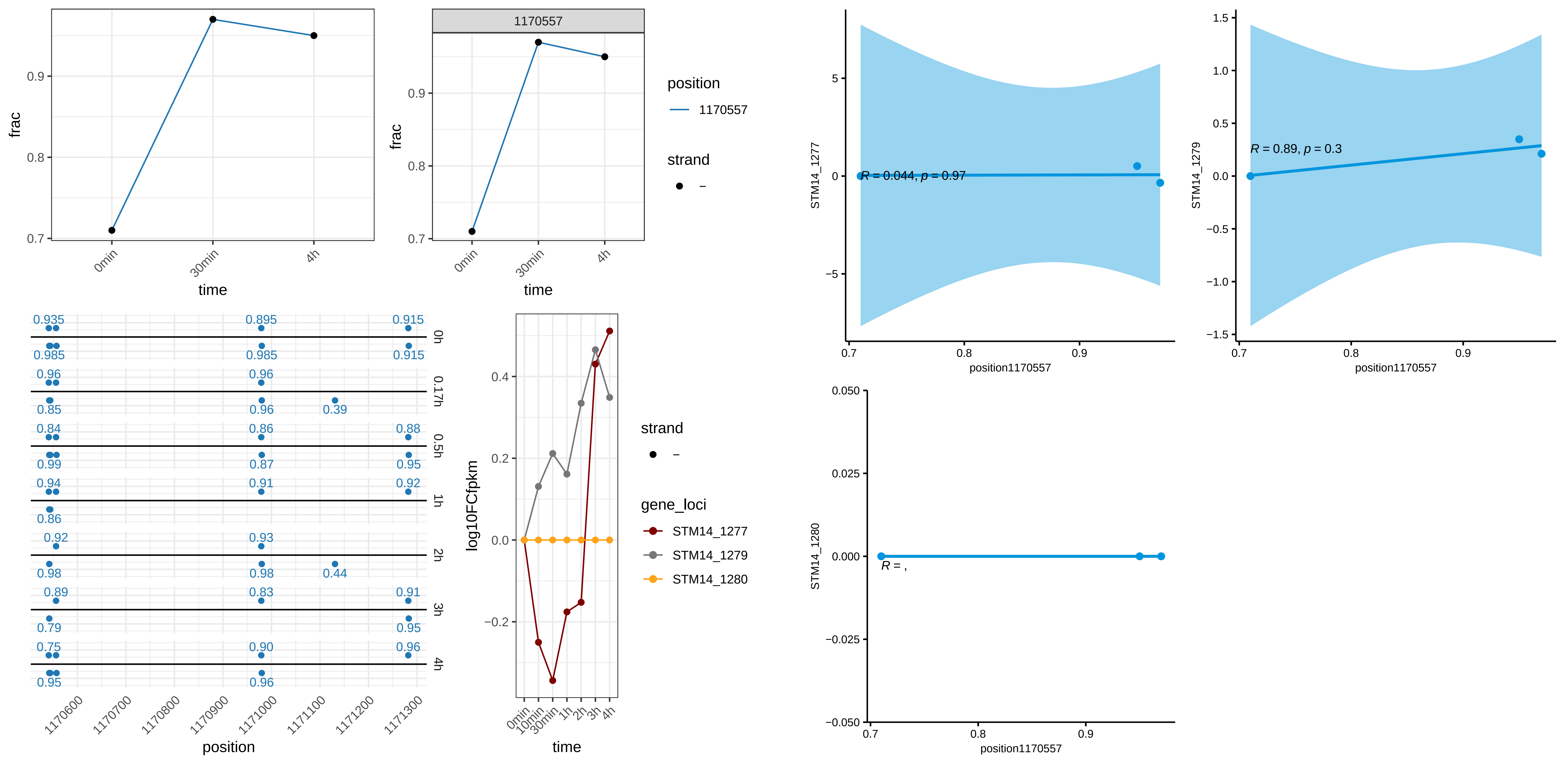

frac
time
0min
30min
4h
1170557
position
strand
–
log10FCfpkm
gene_loci
STM14_1277
STM14_1279
STM14_1280
0h
0.17h
0.5h
1h
2h
3h
4h
0min
10min
30min
STM14_1277
STM14_1279
STM14_1280
position1170557
R = 0.044, p = 0.97
R = 0.89, p = 0.3
R = ,

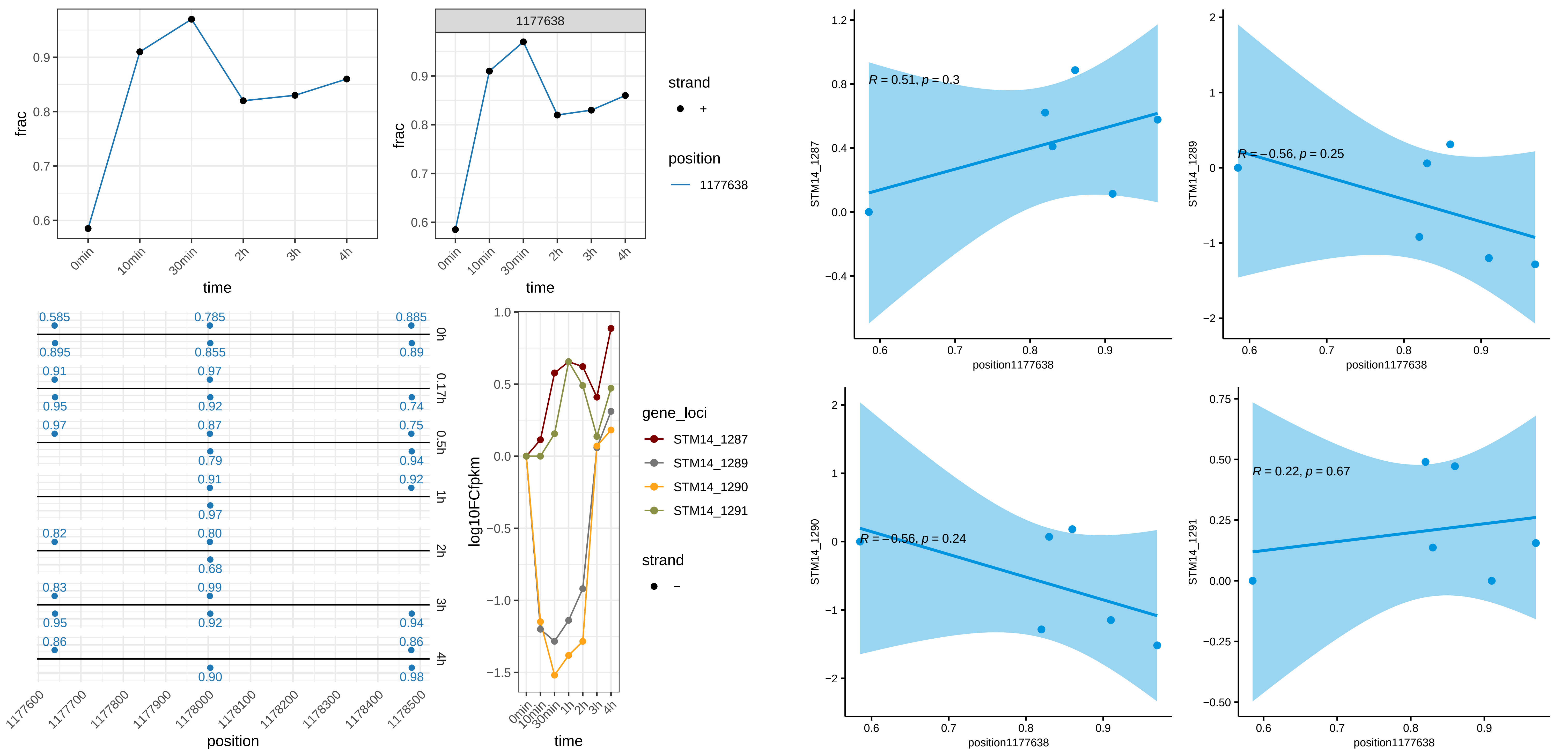

frac
time
1177638
strand
+
position
1177638
log10FCfpkm
gene_loci
STM14_1287
STM14_1289
STM14_1290
STM14_1291
strand
–
position
0h
0.17h
0.5h
1h
2h
3h
4h
STM14_1287
STM14_1289
STM14_1290
STM14_1291
position1177638
R = 0.51, p = 0.3
R = −0.56, p = 0.25
R = −0.56, p = 0.24
R = 0.22, p = 0.67

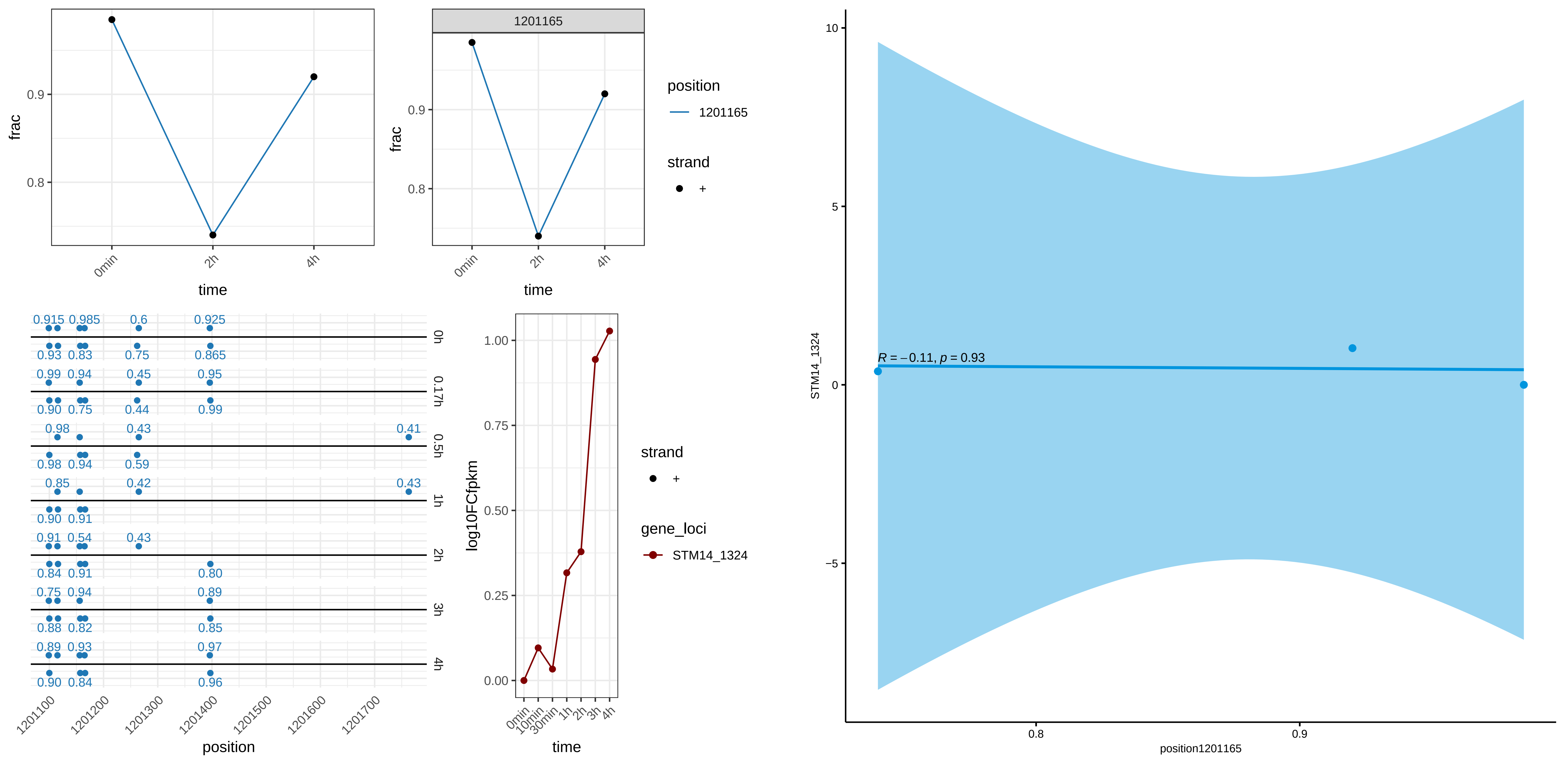

frac
time
0min
2h
4h
1201165
position
strand
+
0h
0.17h
0.5h
1h
2h
3h
4h
position
log10FCfpkm
gene_loci
STM14_1324
R = −0.11, p = 0.93
STM14_1324
position1201165

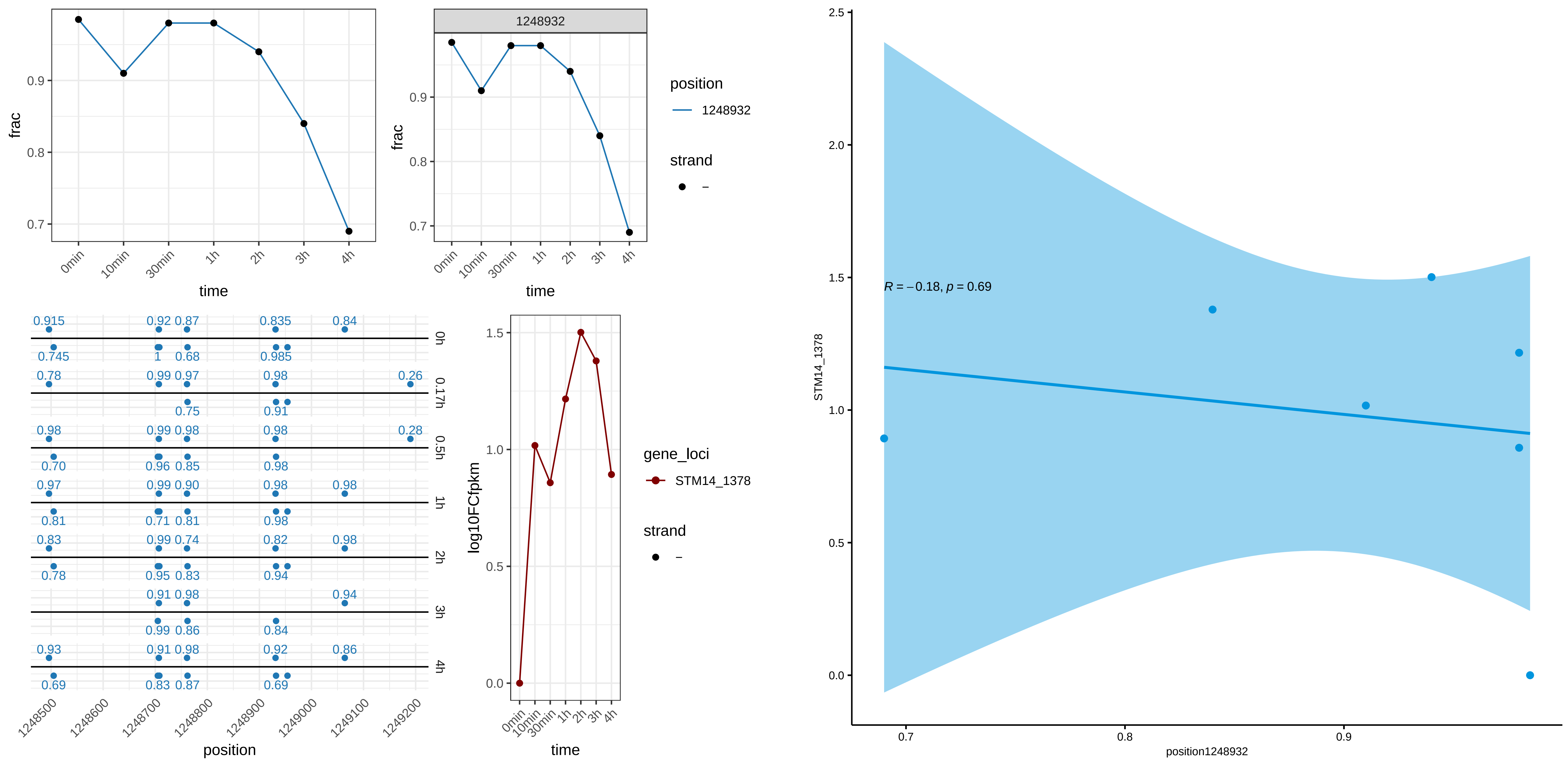

frac
0.9
0.8
0.7
0min
10min
30min
1h
2h
3h
4h
time
1248932
position
1248932
strand
–
0.915
0.92 0.87
0.835
0.84
0.745
1 0.68
0.985
0.78
0.99 0.97
0.98
0.26
0.75
0.91
0.98
0.99 0.98
0.98
0.28
0.70
0.96 0.85
0.98
0.97
0.99 0.90
0.98
0.98
0.81
0.71 0.81
0.98
0.83
0.99 0.74
0.82
0.98
0.78
0.95 0.83
0.94
0.91 0.98
0.94
0.99 0.86
0.84
0.93
0.91 0.98
0.92
0.86
0.69
0.83 0.87
0.69
0h
0.17h
0.5h
1h
2h
3h
4h
1248500
1248600
1248700
1248800
1248900
1249000
1249100
1249200
position
log10FCfpkm
1.5
1.0
0.5
0.0
gene_loci
STM14_1378
strand
–
R = −0.18, p = 0.69
STM14_1378
2.5
2.0
1.5
1.0
0.5
0.0
0.7
0.8
0.9
position1248932

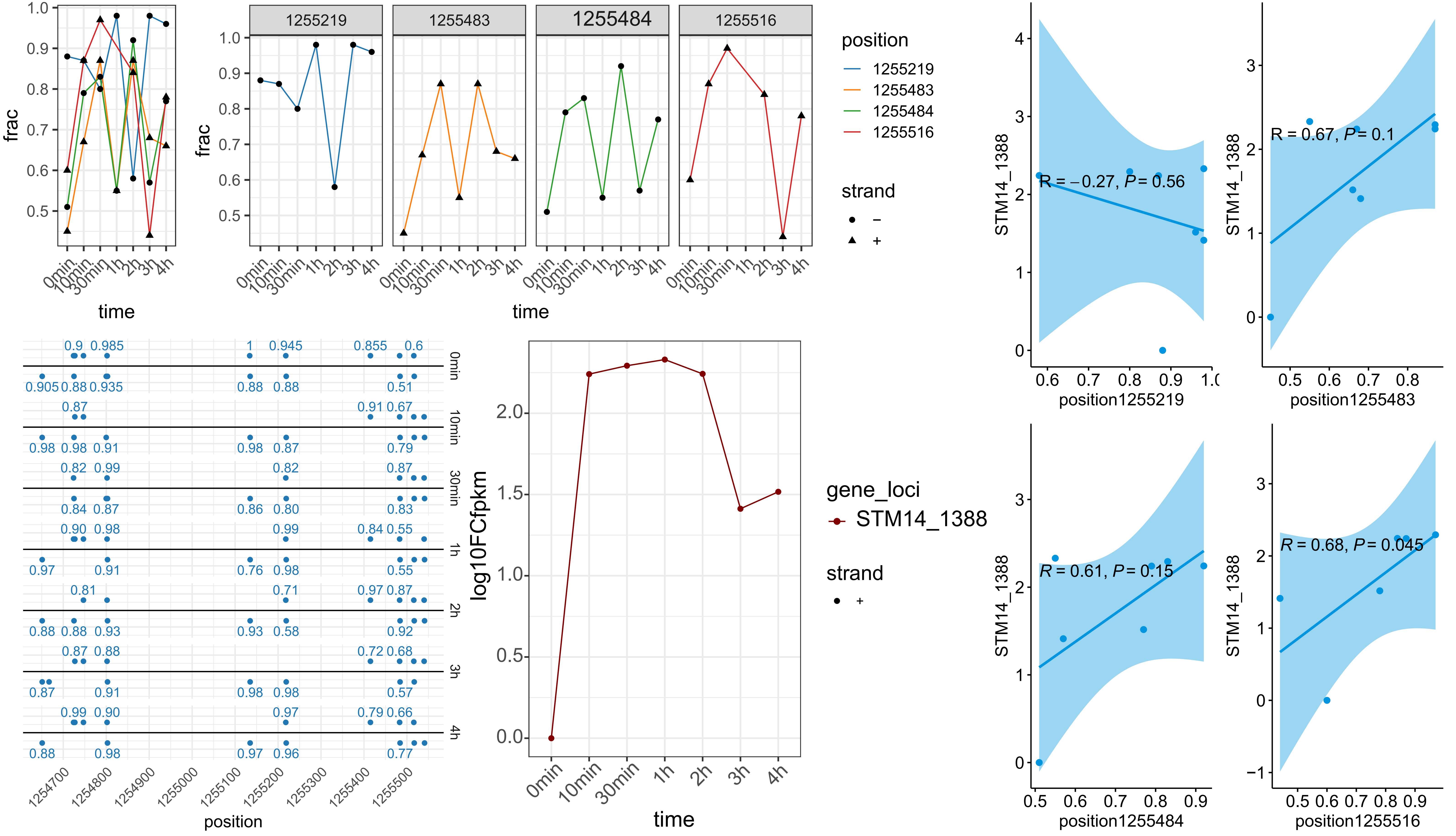

frac
time
0min 10min 30min 1h 2h 3h 4h
1255219
1255483
1255484
1255516
position
strand
–
+
R = –0.27, P = 0.56
R = 0.67, P = 0.1
R = 0.61, P = 0.15
R = 0.68, P = 0.045
STM14_1388
position1255219
position1255483
position1255484
position1255516
log10FCfpkm
gene_loci
STM14_1388
1254700 1254800 1254900 1255000 1255100 1255200 1255300 1255400 1255500

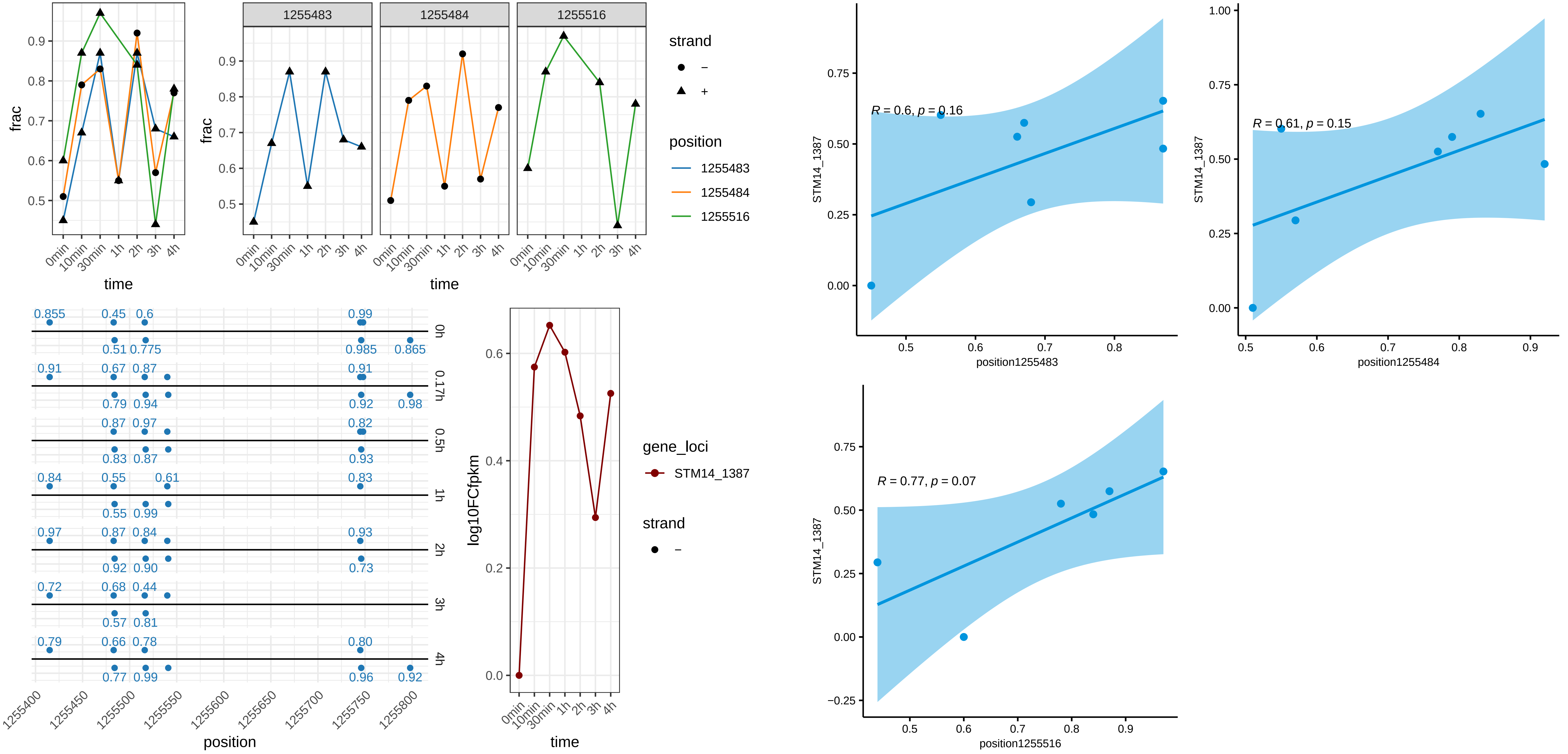

frac
time
0min
10min
30min
1h
2h
3h
4h
1255483
1255484
1255516
strand
–
+
position
1255483
1255484
1255516
0h
0.17h
0.5h
1h
2h
3h
4h
0.855 0.45 0.6 0.99
0.51 0.775 0.985 0.865
0.91 0.67 0.87 0.91
0.79 0.94 0.92 0.98
0.87 0.97 0.82
0.83 0.87 0.93
0.84 0.55 0.61 0.83
0.55 0.99
0.97 0.87 0.84 0.93
0.92 0.90 0.73
0.72 0.68 0.44
0.57 0.81
0.79 0.66 0.78 0.80
0.77 0.99 0.96 0.92
position
log10FCfpkm
gene_loci
STM14_1387
strand
–
STM14_1387
position1255483
R = 0.6, p = 0.16
position1255484
R = 0.61, p = 0.15
position1255516
R = 0.77, p = 0.07

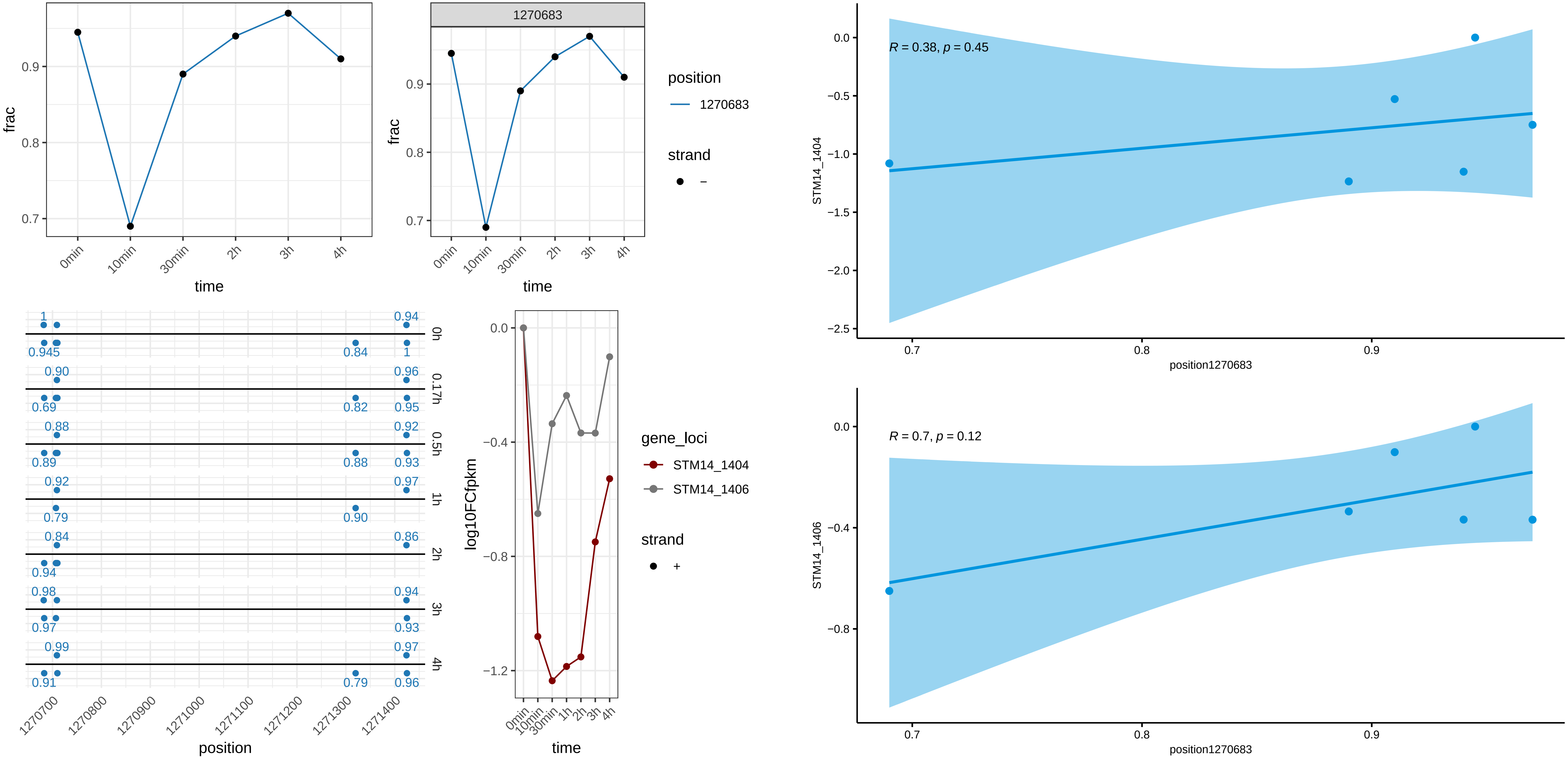

1270683
position
1270683
strand
frac
time
R = 0.38, p = 0.45
STM14_1404
position1270683
R = 0.7, p = 0.12
STM14_1406
position1270683
gene_loci
STM14_1404
STM14_1406
strand
+
log10FCfpkm
position

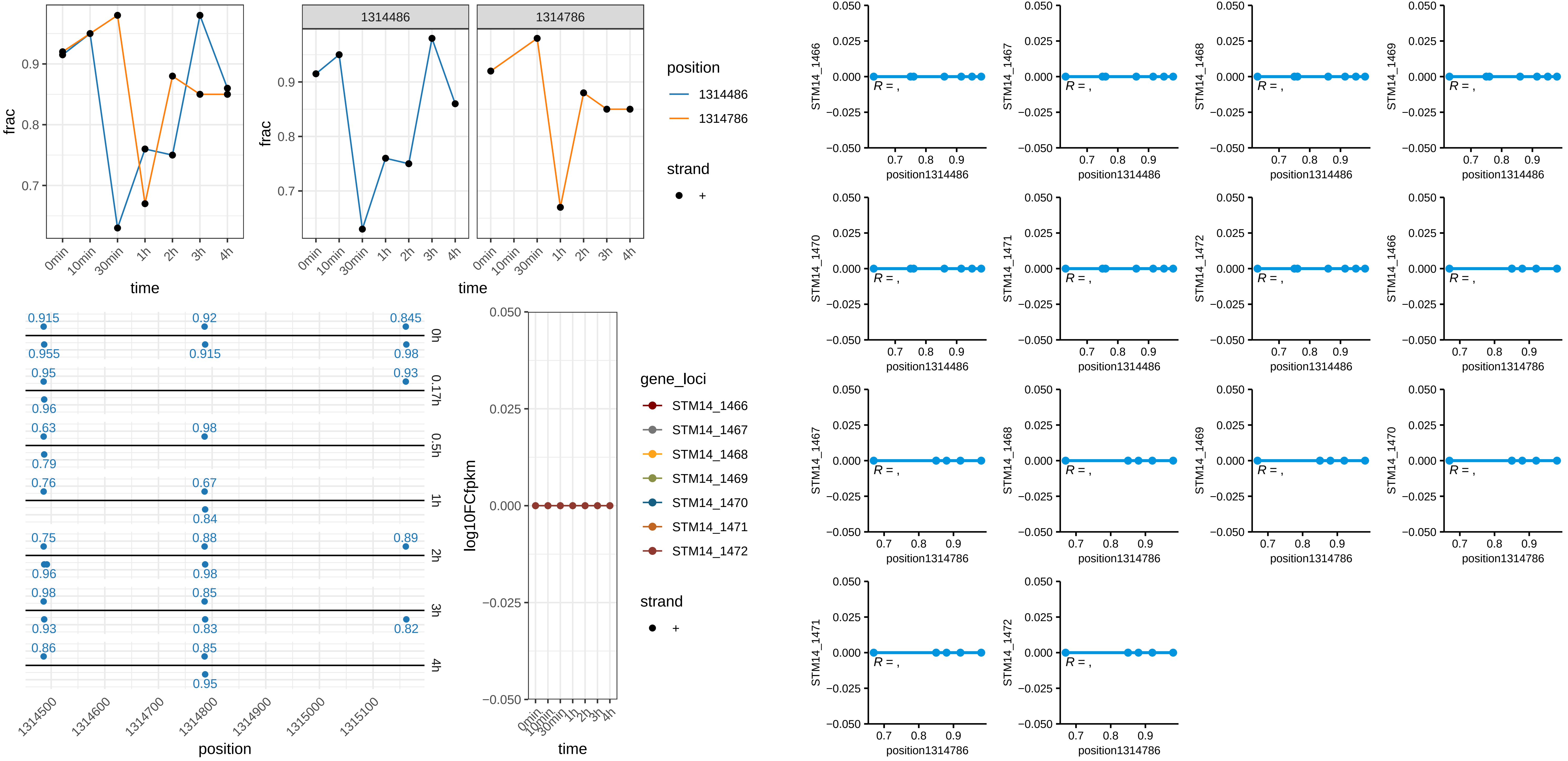

frac
time
0min
10min
30min
1h
2h
3h
4h
1314486
1314786
position
strand
+
0.915
0.92
0.845
0.955
0.915
0.98
0.95
0.93
0.96
0.63
0.98
0.79
0.76
0.67
0.84
0.75
0.88
0.89
0.96
0.98
0.98
0.85
0.93
0.83
0.82
0.86
0.85
0.95
0h
0.17h
0.5h
1h
2h
3h
4h
log10FCfpkm
gene_loci
STM14_1466
STM14_1467
STM14_1468
STM14_1469
STM14_1470
STM14_1471
STM14_1472
R = ,
position1314486
position1314786

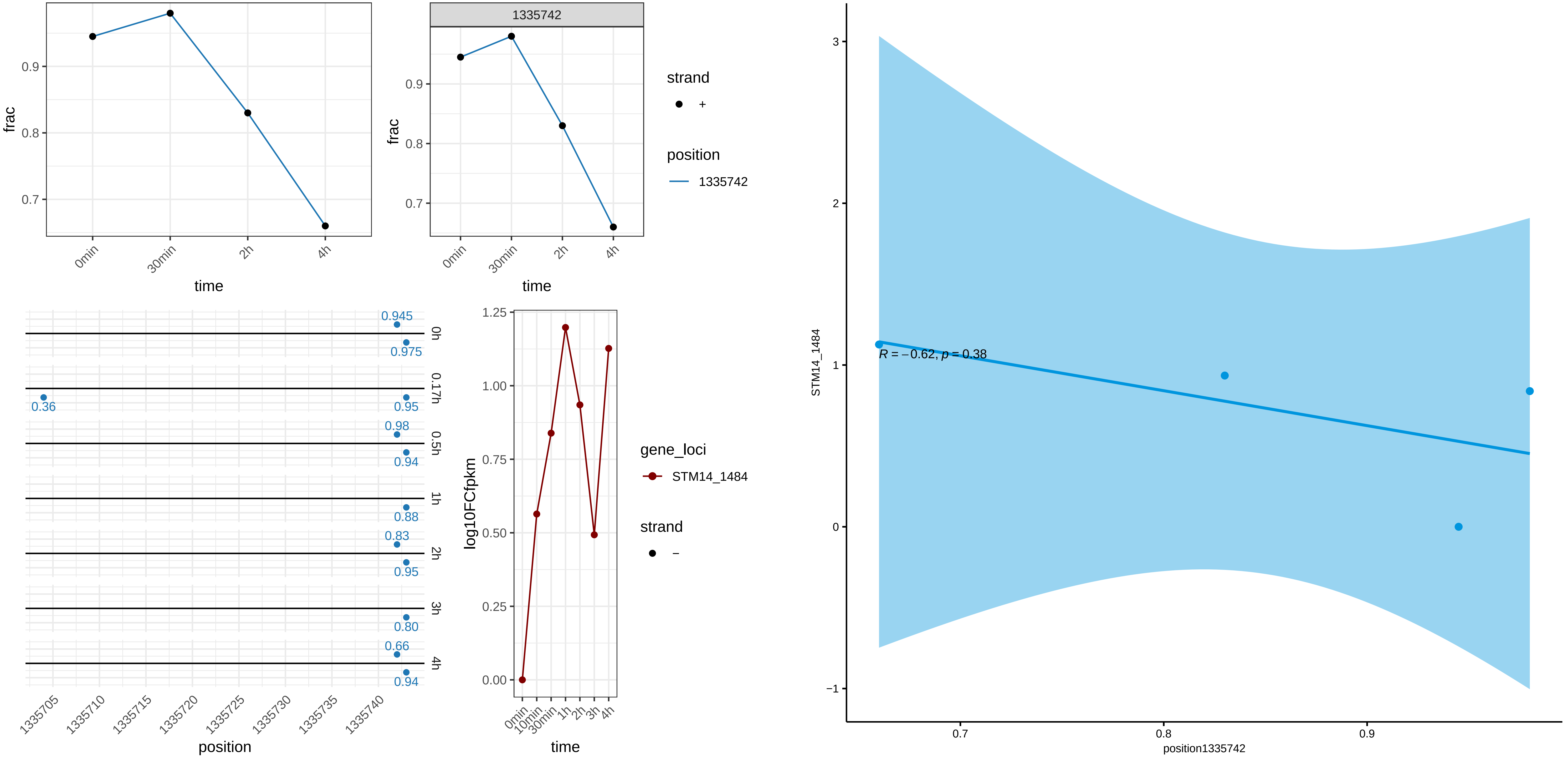

frac
time
0min
30min
2h
4h
1335742
strand
+
position
1335742
0.945
0.975
0.36
0.95
0.98
0.94
0.88
0.83
0.95
0.80
0.66
0.94
0h
0.17h
0.5h
1h
2h
3h
4h
position
log10FCfpkm
gene_loci
STM14_1484
strand
−
10min
1h
3h
STM14_1484
R = −0.62, p = 0.38
position1335742

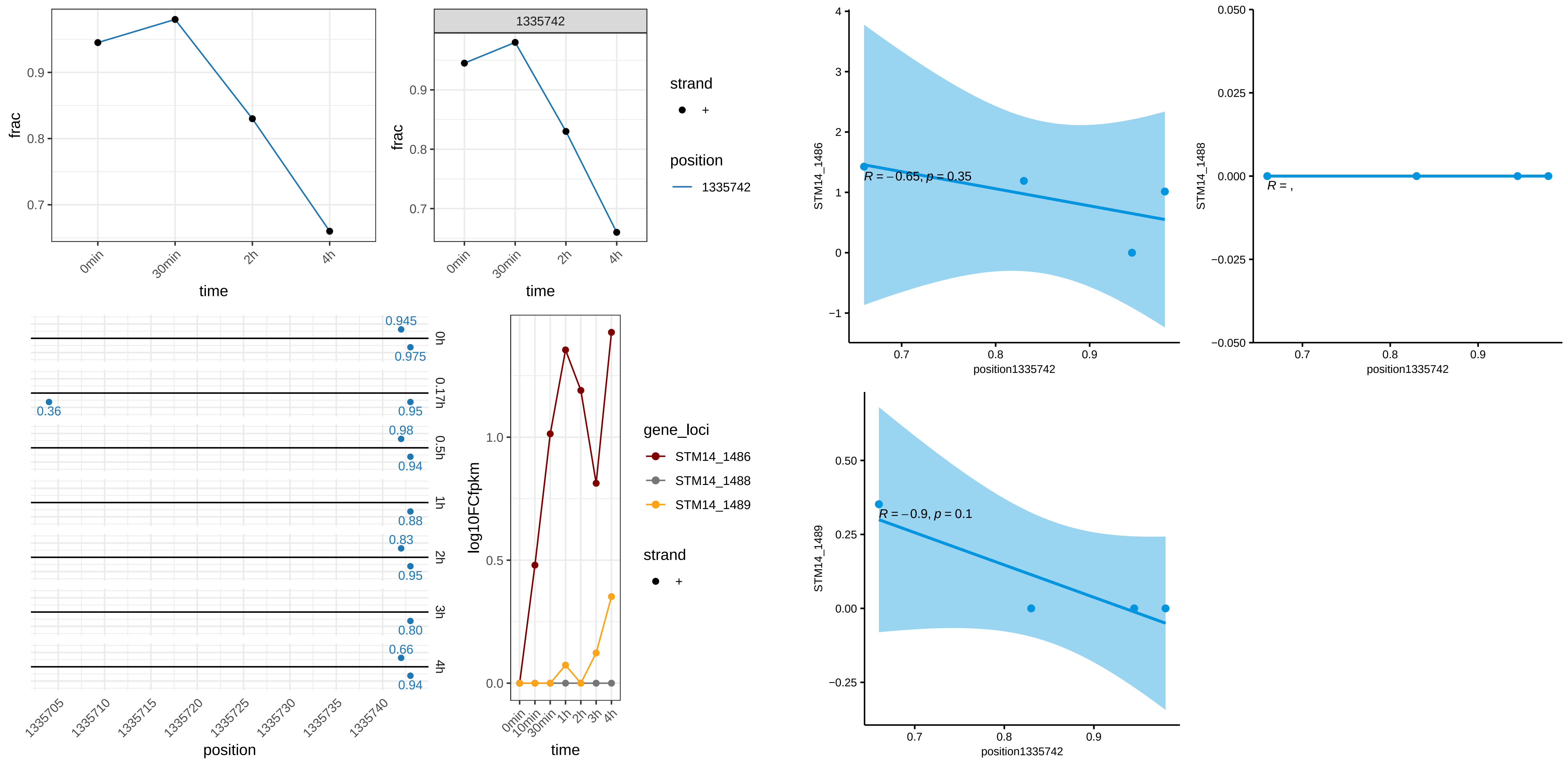

frac
time
0min
30min
2h
4h
1335742
strand
+
position
1335742
0.945
0.975
0.36
0.95
0.98
0.94
0.88
0.83
0.95
0.80
0.66
0.94
0h
0.17h
0.5h
1h
2h
3h
4h
position
log10FCfpkm
10min
gene_loci
STM14_1486
STM14_1488
STM14_1489
R = −0.65, p = 0.35
position1335742
R = ,
R = −0.9, p = 0.1

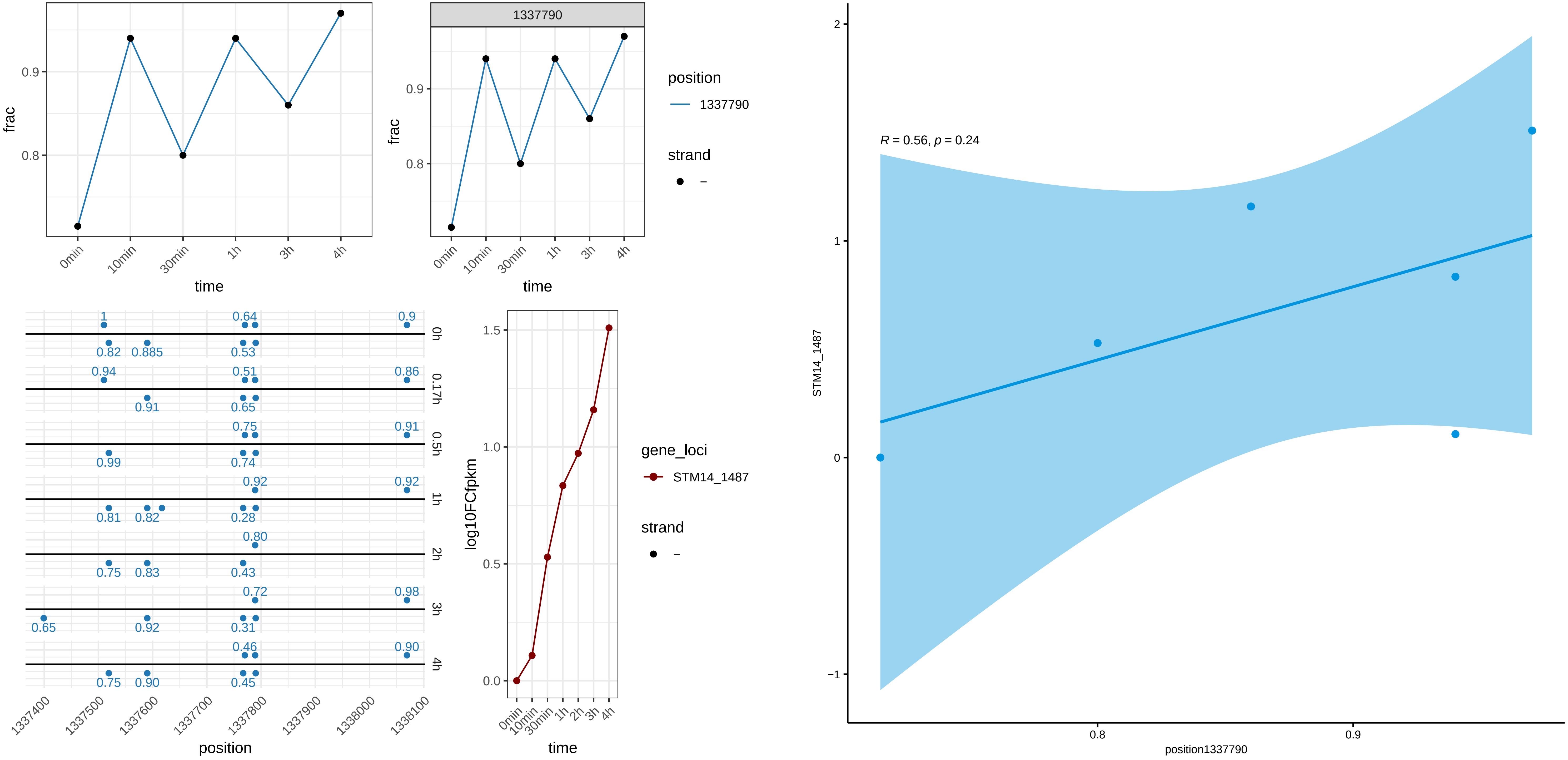
frac
0.9
0.8
0min
10min
30min
1h
3h
4h
time
1337790
position
1337790
strand
–
1
0.64
0.9
0.82
0.885
0.53
0.94
0.51
0.86
0.91
0.65
0.75
0.91
0.99
0.74
0.92
0.92
0.81
0.82
0.28
0.80
0.75
0.83
0.43
0.72
0.98
0.65
0.92
0.31
0.46
0.90
0.75
0.90
0.45
0h
0.17h
0.5h
1h
2h
3h
4h
1337400
1337500
1337600
1337700
1337800
1337900
1338000
1338100
position
log10FCfpkm
1.5
1.0
0.5
0.0
2h
gene_loci
STM14_1487
R = 0.56, p = 0.24
STM14_1487
position1337790
2
1
0
−1

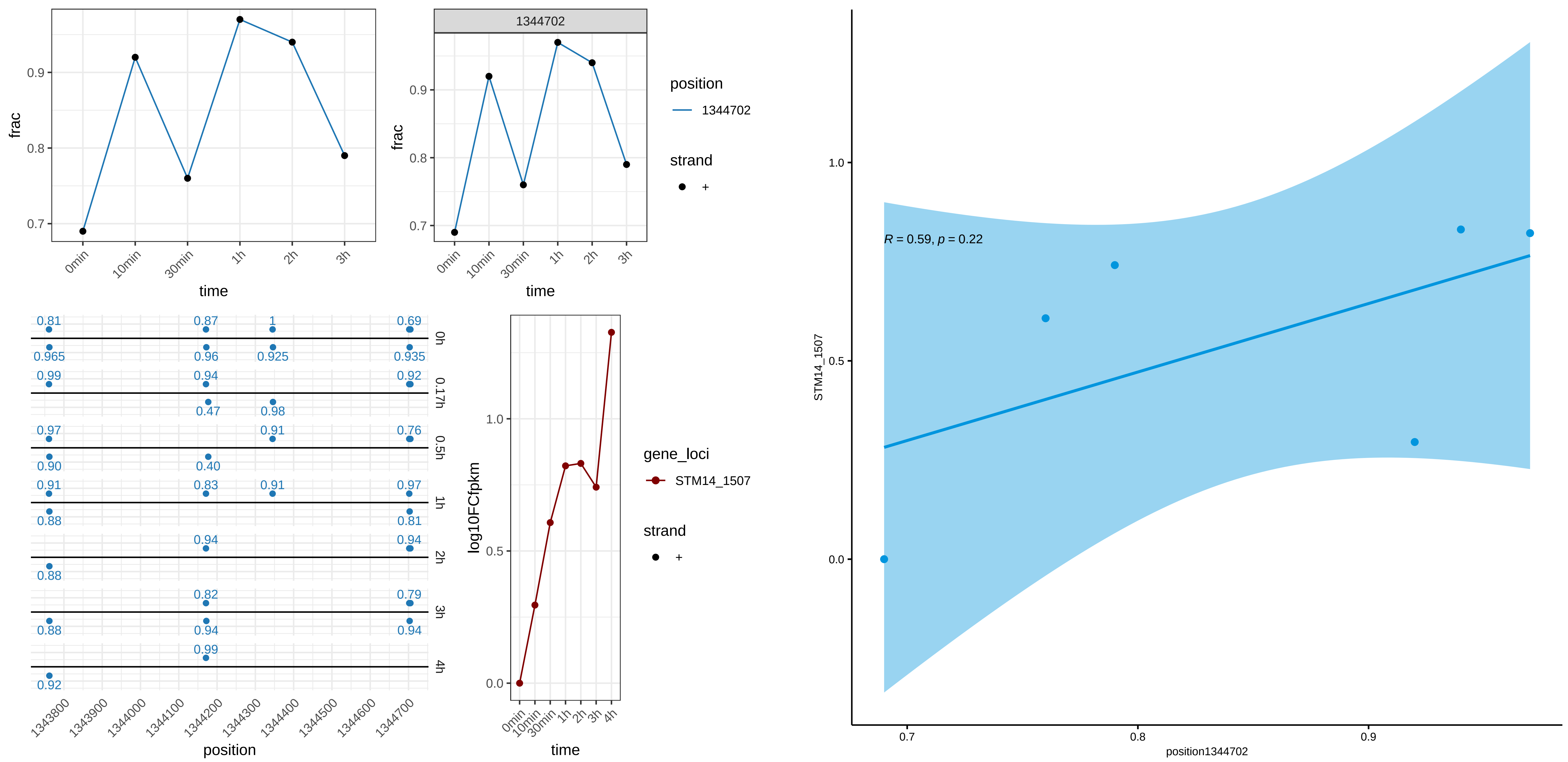

1344702
frac
time
position
1344702
strand
+
log10FCfpkm
gene_loci
STM14_1507
R = 0.59, p = 0.22
STM14_1507
position1344702

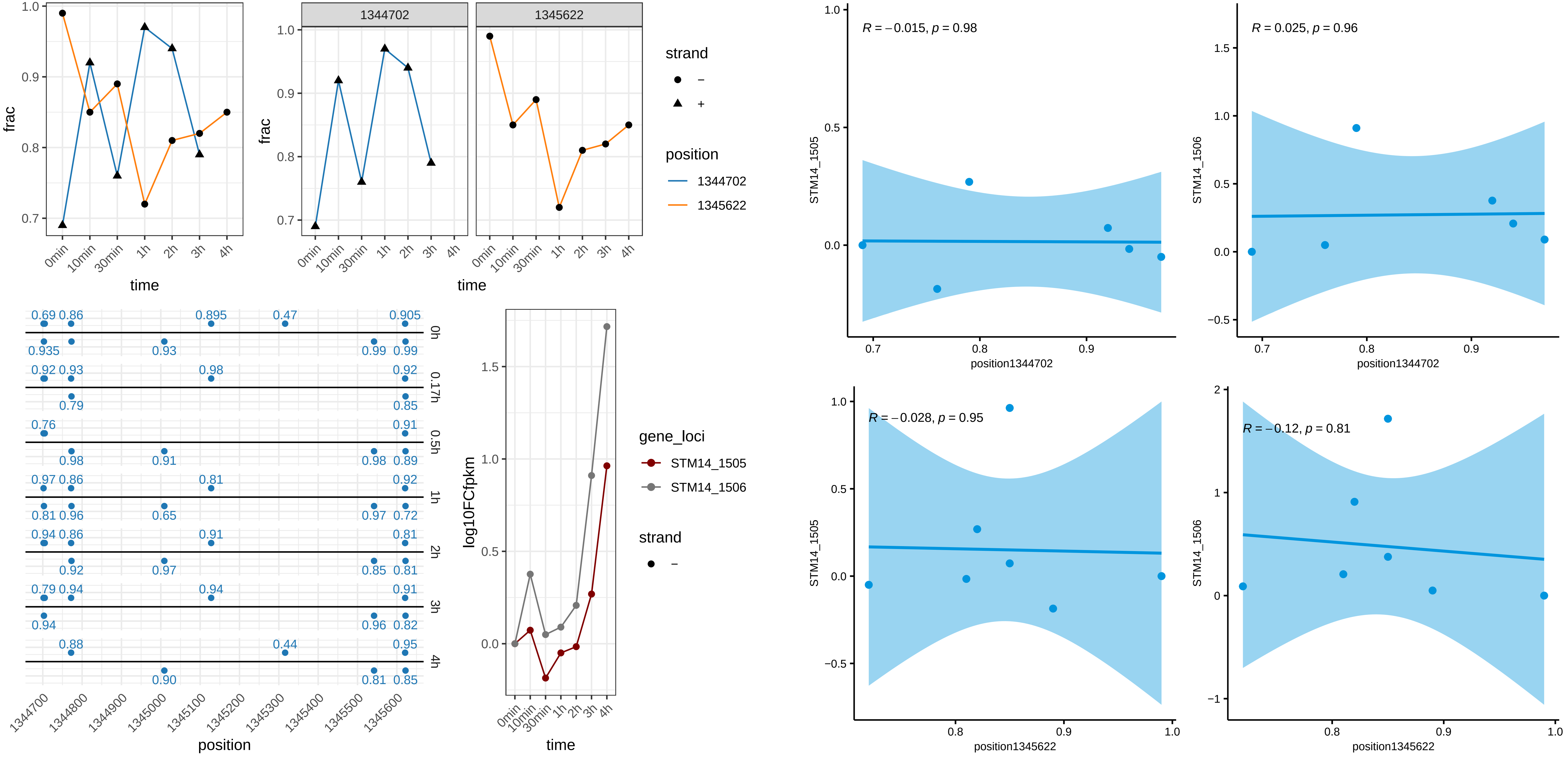

frac
time
0min 10min 30min 1h 2h 3h 4h
1344702
1345622
strand
–
+
position
1344702
1345622
R = –0.015, p = 0.98
STM14_1505
position1344702
R = 0.025, p = 0.96
STM14_1506
position1344702
R = –0.028, p = 0.95
STM14_1505
position1345622
R = –0.12, p = 0.81
STM14_1506
position1345622
0.69 0.86 0.895 0.47 0.905
0.935 0.93 0.99 0.99
0h
0.92 0.93 0.98 0.92
0.79 0.85
0.17h
0.76 0.91
0.98 0.91 0.98 0.89
0.5h
0.97 0.86 0.81 0.92
0.81 0.96 0.65 0.97 0.72
1h
0.94 0.86 0.91 0.81
0.92 0.97 0.85 0.81
2h
0.79 0.94 0.94 0.91
0.94 0.96 0.82
3h
0.88 0.44 0.95
0.90 0.81 0.85
4h
1344700 1344800 1344900 1345000 1345100 1345200 1345300 1345400 1345500 1345600
position
log10FCfpkm
time
gene_loci
STM14_1505
STM14_1506
strand
–

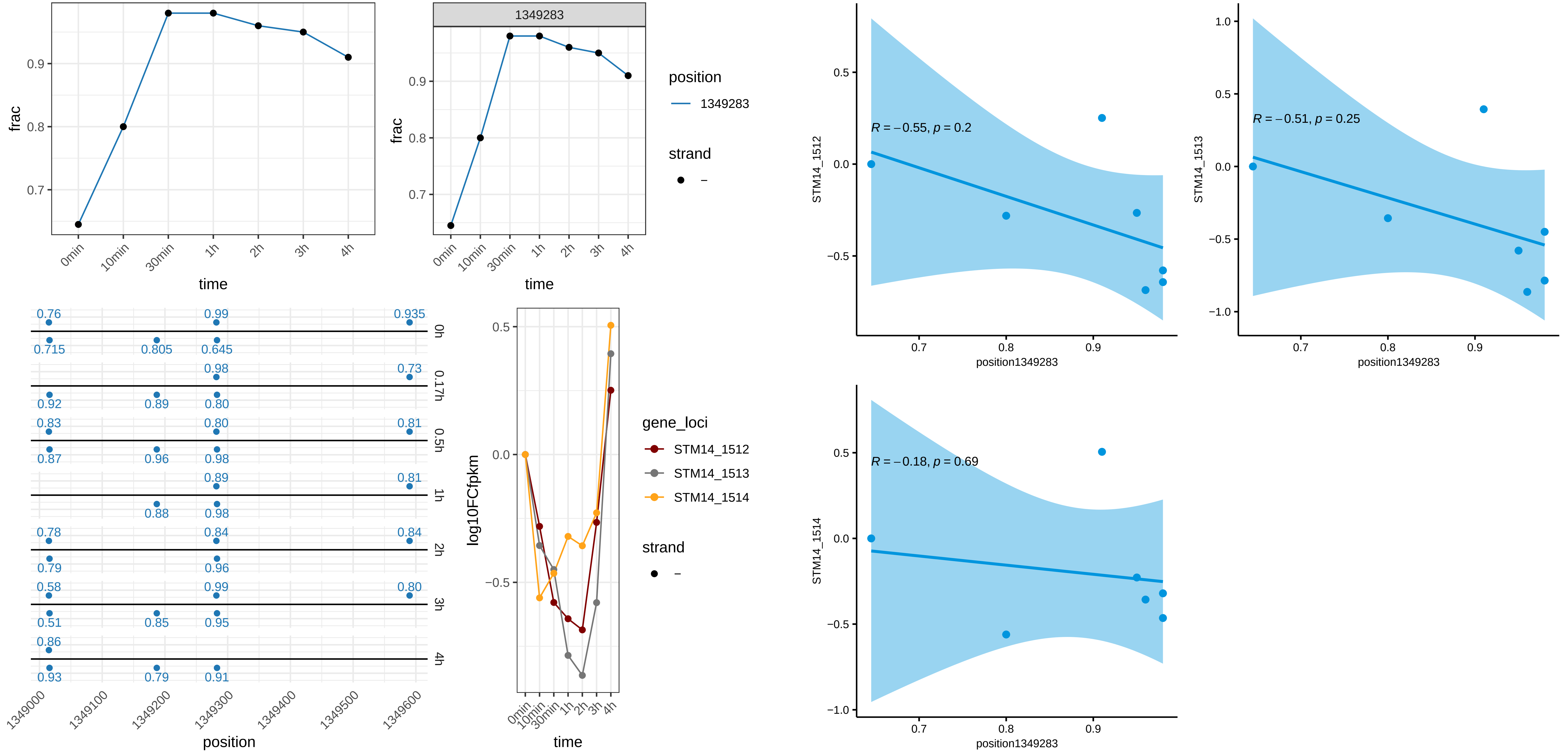

frac
time
0min
10min
30min
1h
2h
3h
4h
1349283
position
1349283
strand
–
0.76
0.99
0.935
0.715
0.805
0.645
0.98
0.73
0.92
0.89
0.80
0.83
0.80
0.81
0.87
0.96
0.98
0.89
0.81
0.88
0.98
0.78
0.84
0.84
0.79
0.96
0.58
0.99
0.80
0.51
0.85
0.95
0.86
0.93
0.79
0.91
0h
0.17h
0.5h
1h
2h
3h
4h
1349000
1349100
1349200
1349300
1349400
1349500
1349600
position
log10FCfpkm
gene_loci
STM14_1512
STM14_1513
STM14_1514
strand
–
R = –0.55, p = 0.2
STM14_1512
position1349283
R = –0.51, p = 0.25
STM14_1513
position1349283
R = –0.18, p = 0.69
STM14_1514
position1349283

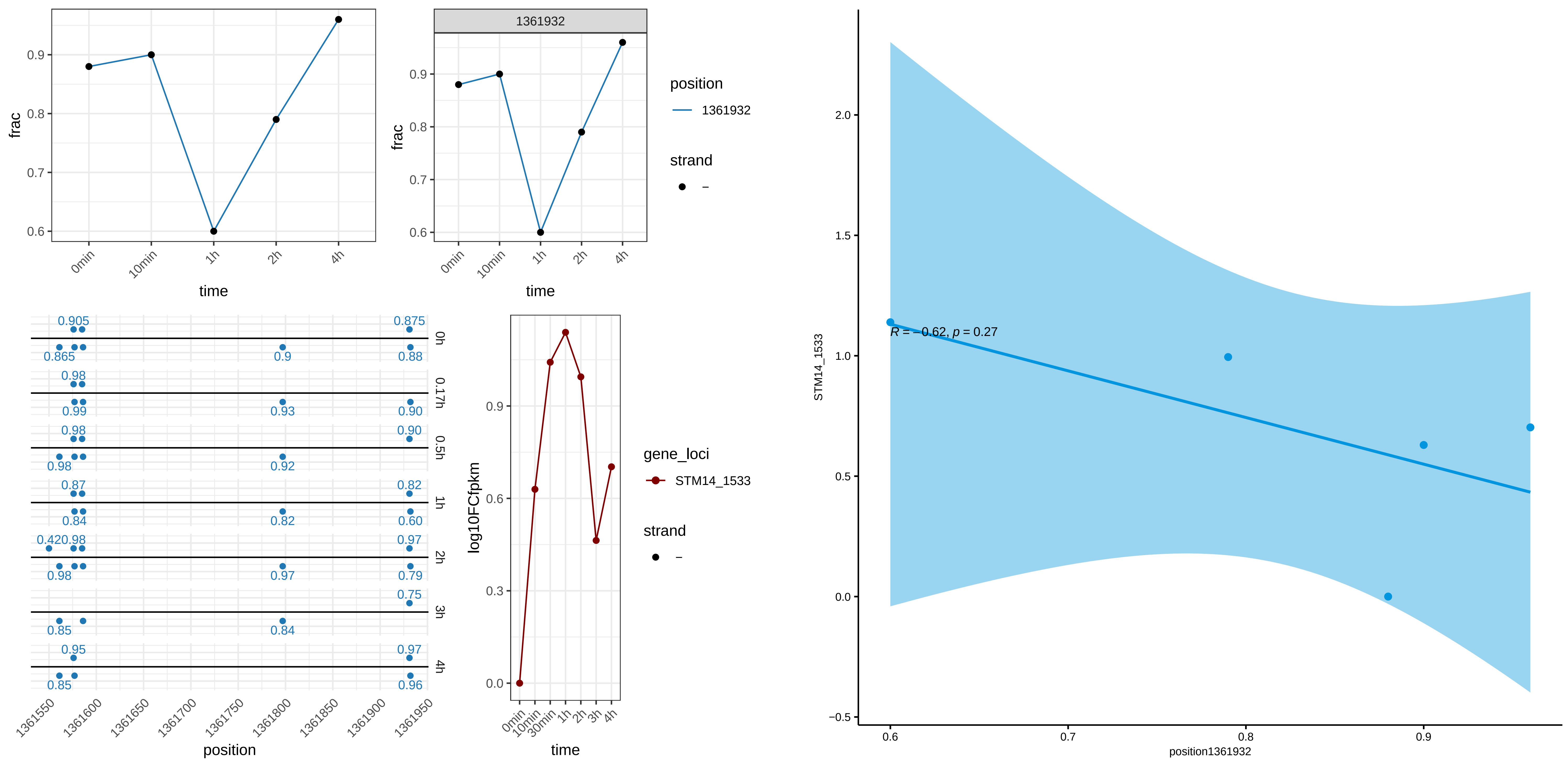

frac
time
0min
10min
1h
2h
4h
1361932
position
strand
log10FCfpkm
gene_loci
STM14_1533
R = −0.62, p = 0.27
STM14_1533
position1361932

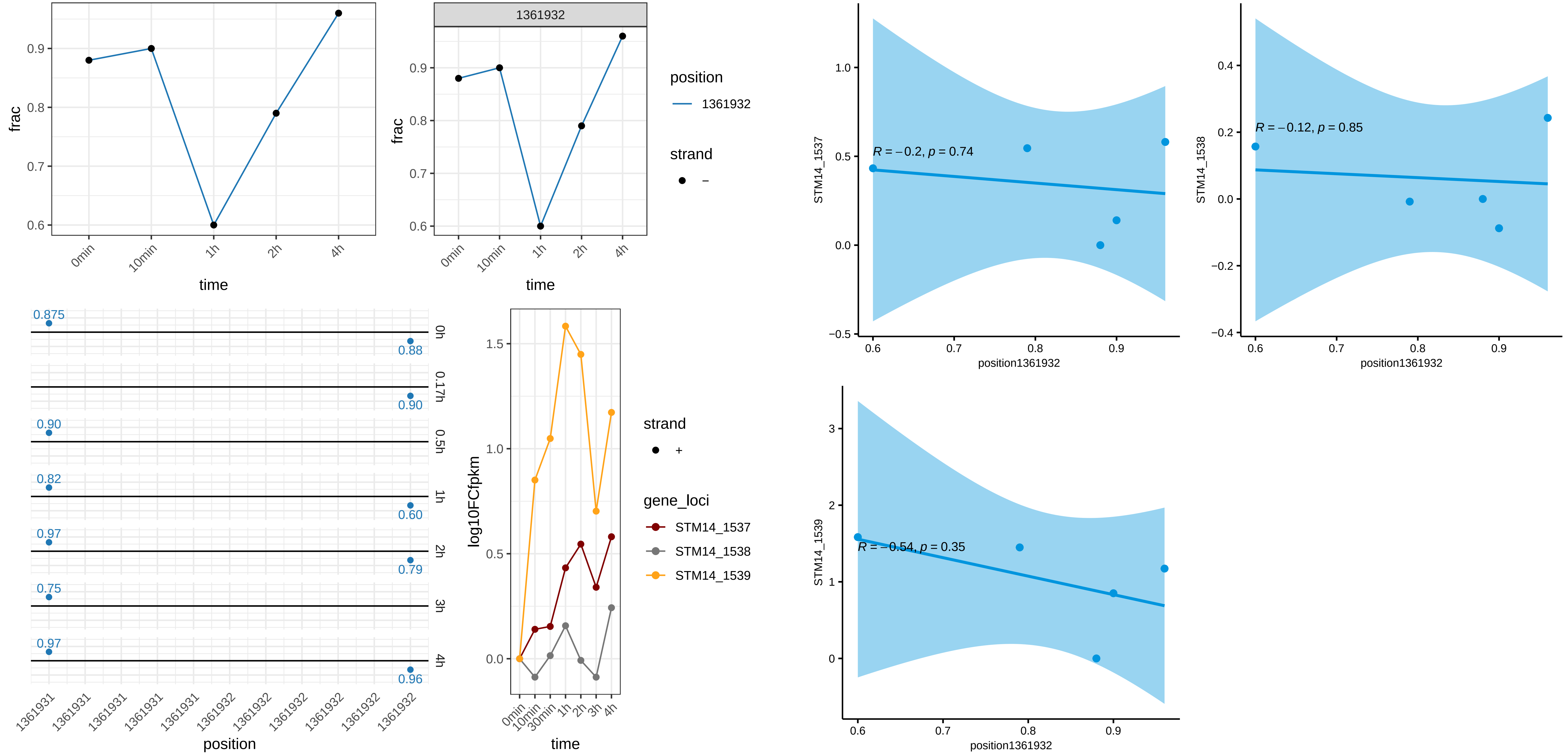

frac
time
0min
10min
1h
2h
4h
1361932
position
strand
–
+
0.875
0.88
0.90
0.82
0.60
0.97
0.79
0.75
0.96
0h
0.17h
0.5h
3h
log10FCfpkm
30min
gene_loci
STM14_1537
STM14_1538
STM14_1539
R = −0.2, p = 0.74
R = −0.12, p = 0.85
R = −0.54, p = 0.35
position1361932

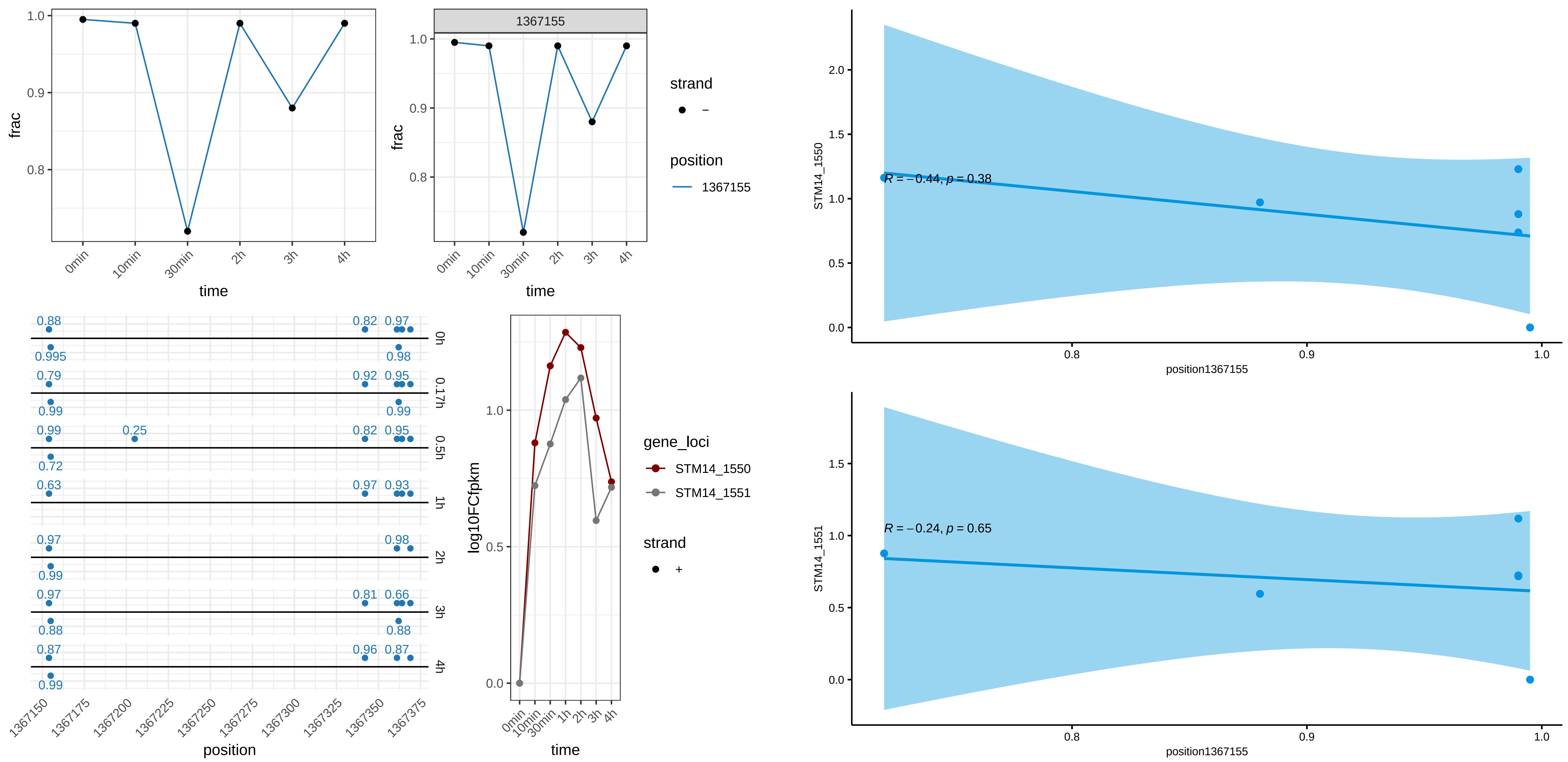

frac
time
0min
10min
30min
2h
3h
4h
1367155
strand
position
1367155
position
log10FCfpkm
gene_loci
STM14_1550
STM14_1551
0h
0.17h
0.5h
1h
2h
3h
4h
R = −0.44, p = 0.38
STM14_1550
position1367155
R = −0.24, p = 0.65
STM14_1551
position1367155

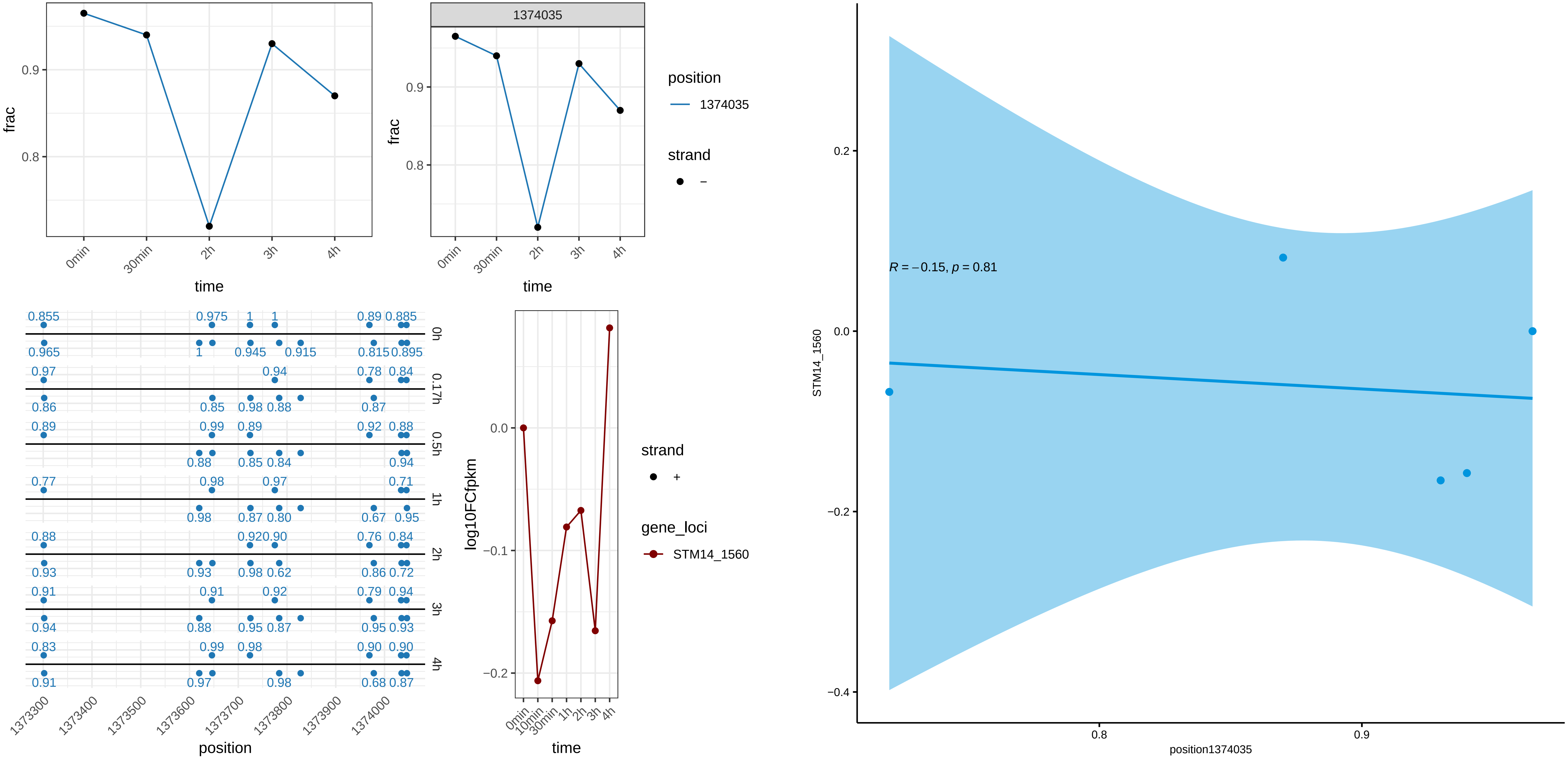
frac
time
1374035
position
strand
log10FCfpkm
gene_loci
STM14_1560
R = −0.15, p = 0.81
STM14_1560
position1374035

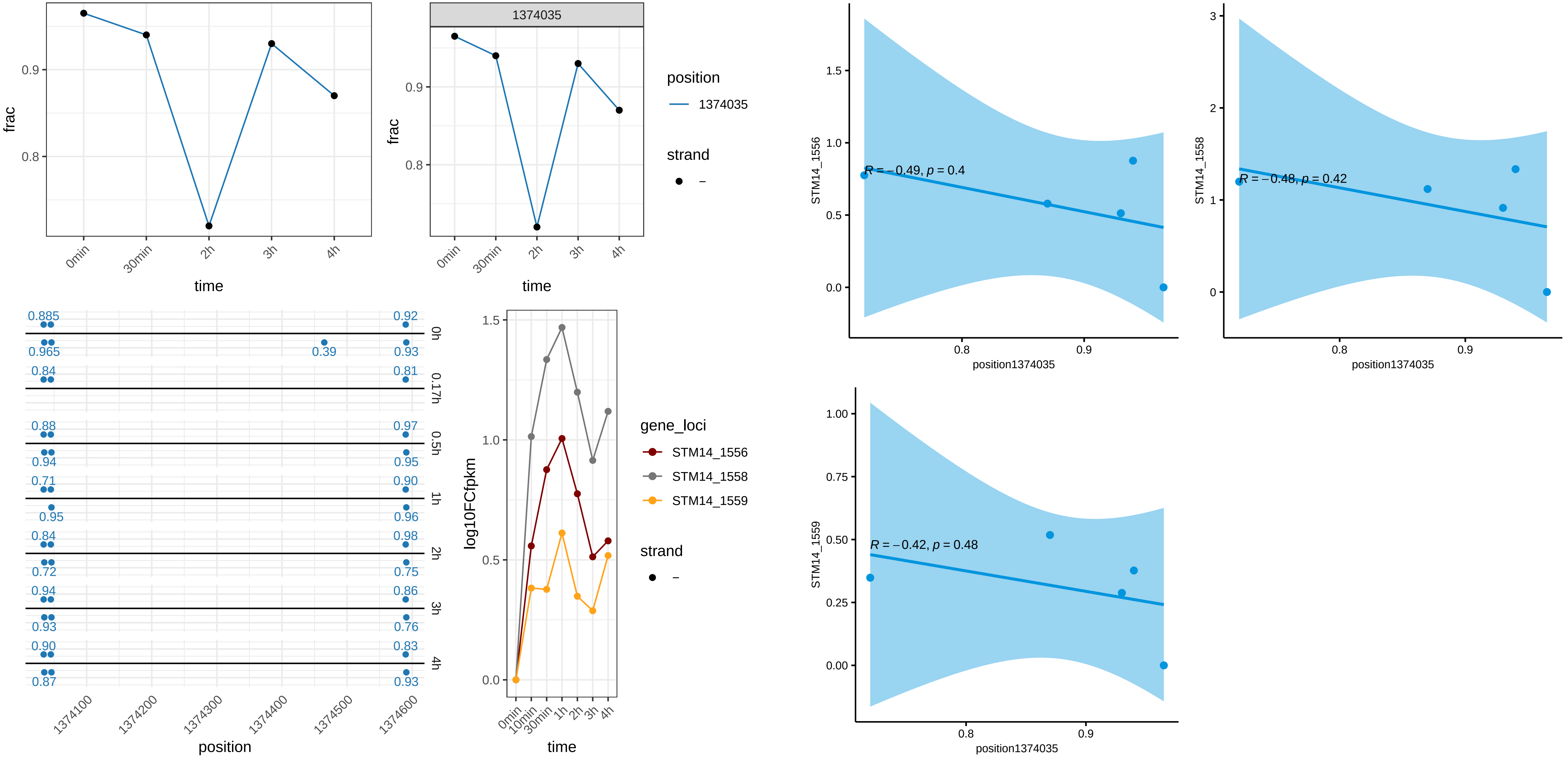

frac
time
0min
30min
2h
3h
4h
1374035
position
strand
–
0.885
0.92
0.965
0.39
0.93
0.84
0.81
0.88
0.97
0.94
0.95
0.71
0.90
0.96
0.98
0.72
0.75
0.86
0.76
0.83
0.87
0h
0.17h
0.5h
1h
1374100
1374200
1374300
1374400
1374500
1374600
log10FCfpkm
10min
gene_loci
STM14_1556
STM14_1558
STM14_1559
R = −0.49, p = 0.4
R = −0.48, p = 0.42
R = −0.42, p = 0.48
position1374035

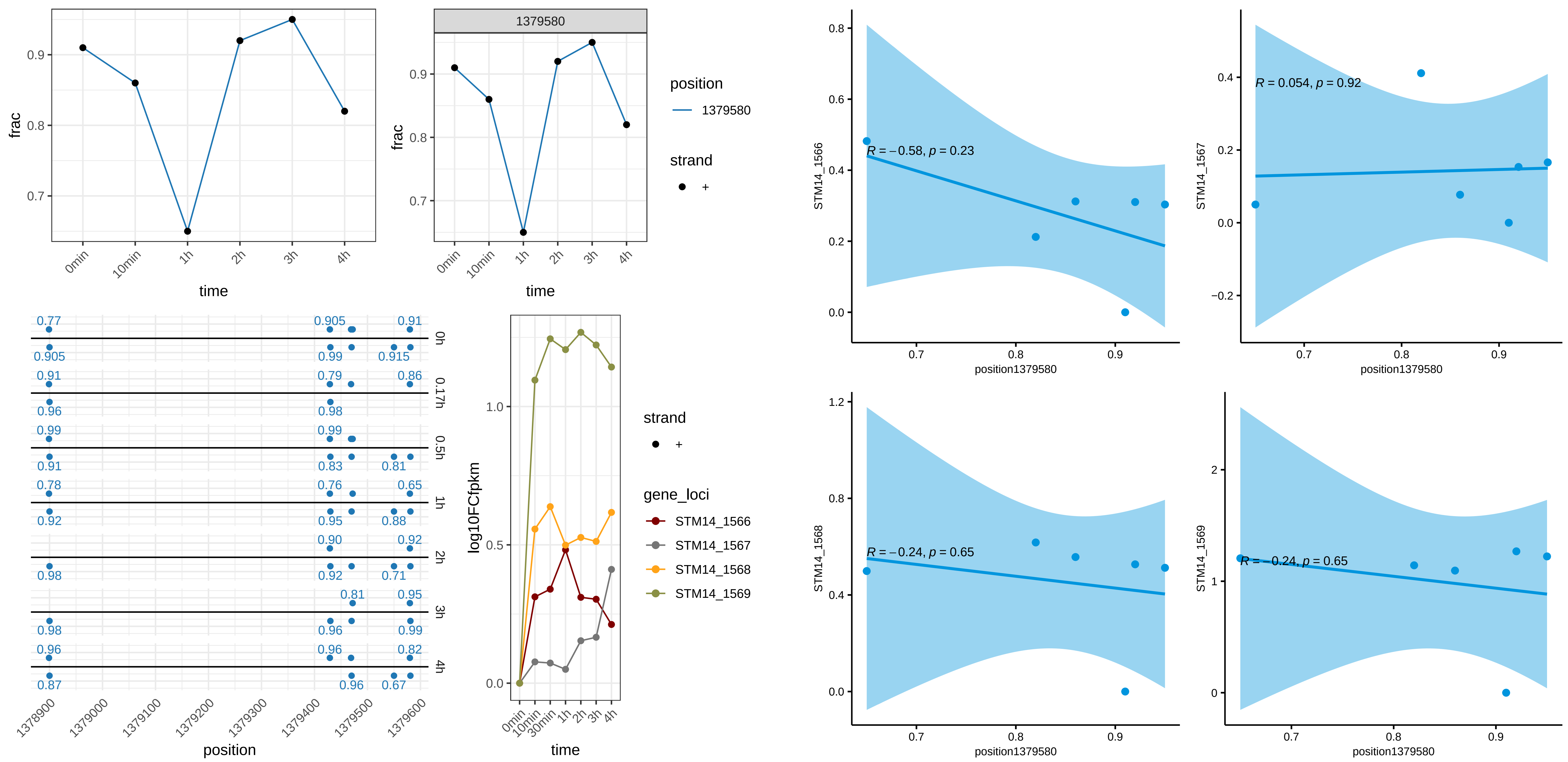

frac
time
0min
10min
1h
2h
3h
4h
1379580
position
strand
+
position
0h
0.17h
0.5h
1h
2h
3h
4h
log10FCfpkm
gene_loci
STM14_1566
STM14_1567
STM14_1568
STM14_1569
30min
STM14_1566
STM14_1567
STM14_1568
STM14_1569
position1379580
R = −0.58, p = 0.23
R = 0.054, p = 0.92
R = −0.24, p = 0.65
R = −0.24, p = 0.65

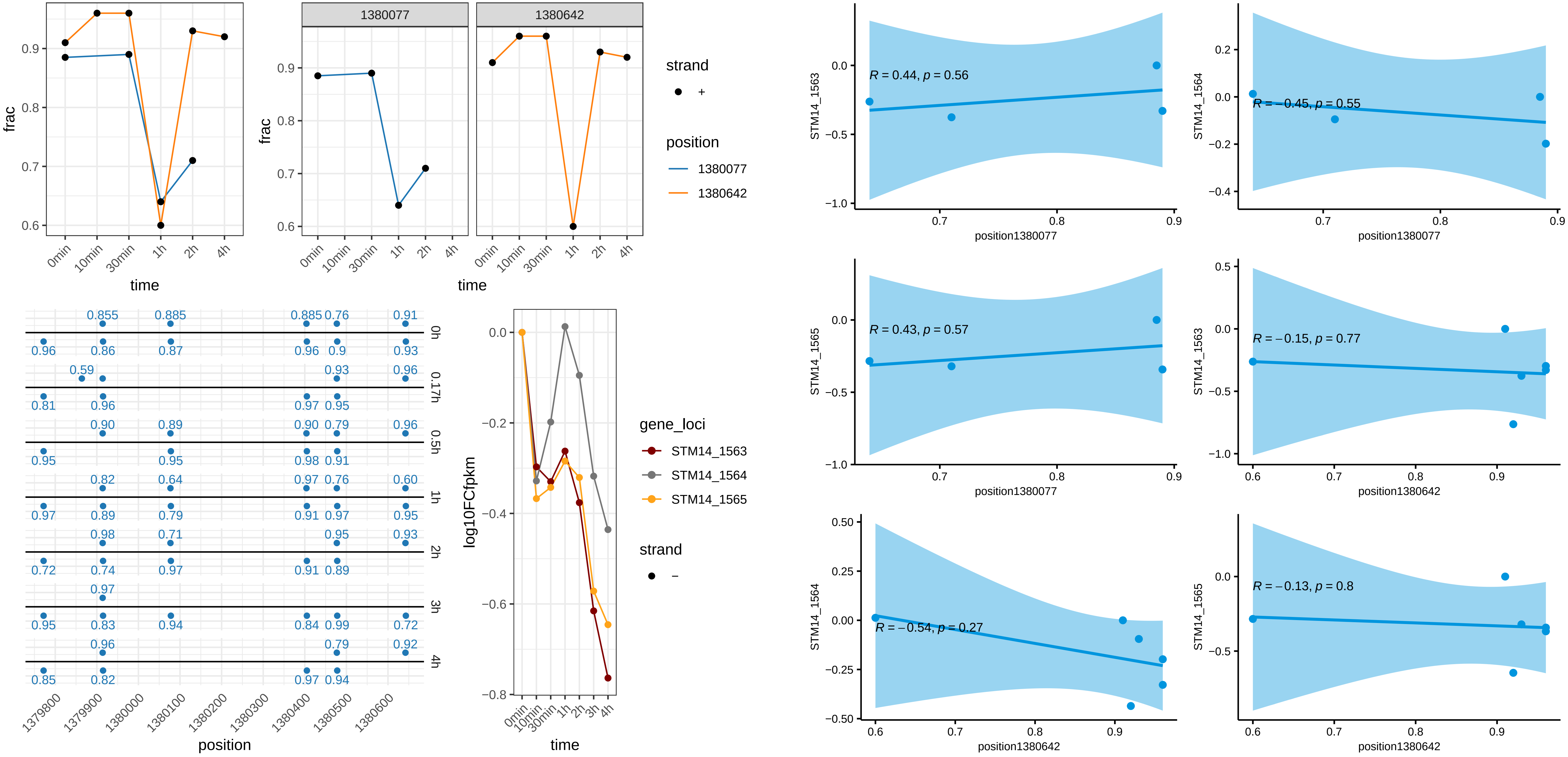

frac
time
1380077
1380642
strand
+
position
1380077
1380642
gene_loci
STM14_1563
STM14_1564
STM14_1565
strand
–
log10FCfpkm
position
R = 0.44, p = 0.56
R = −0.45, p = 0.55
R = 0.43, p = 0.57
R = −0.15, p = 0.77
R = −0.54, p = 0.27
R = −0.13, p = 0.8
STM14_1563
STM14_1564
STM14_1565
position1380077
position1380642

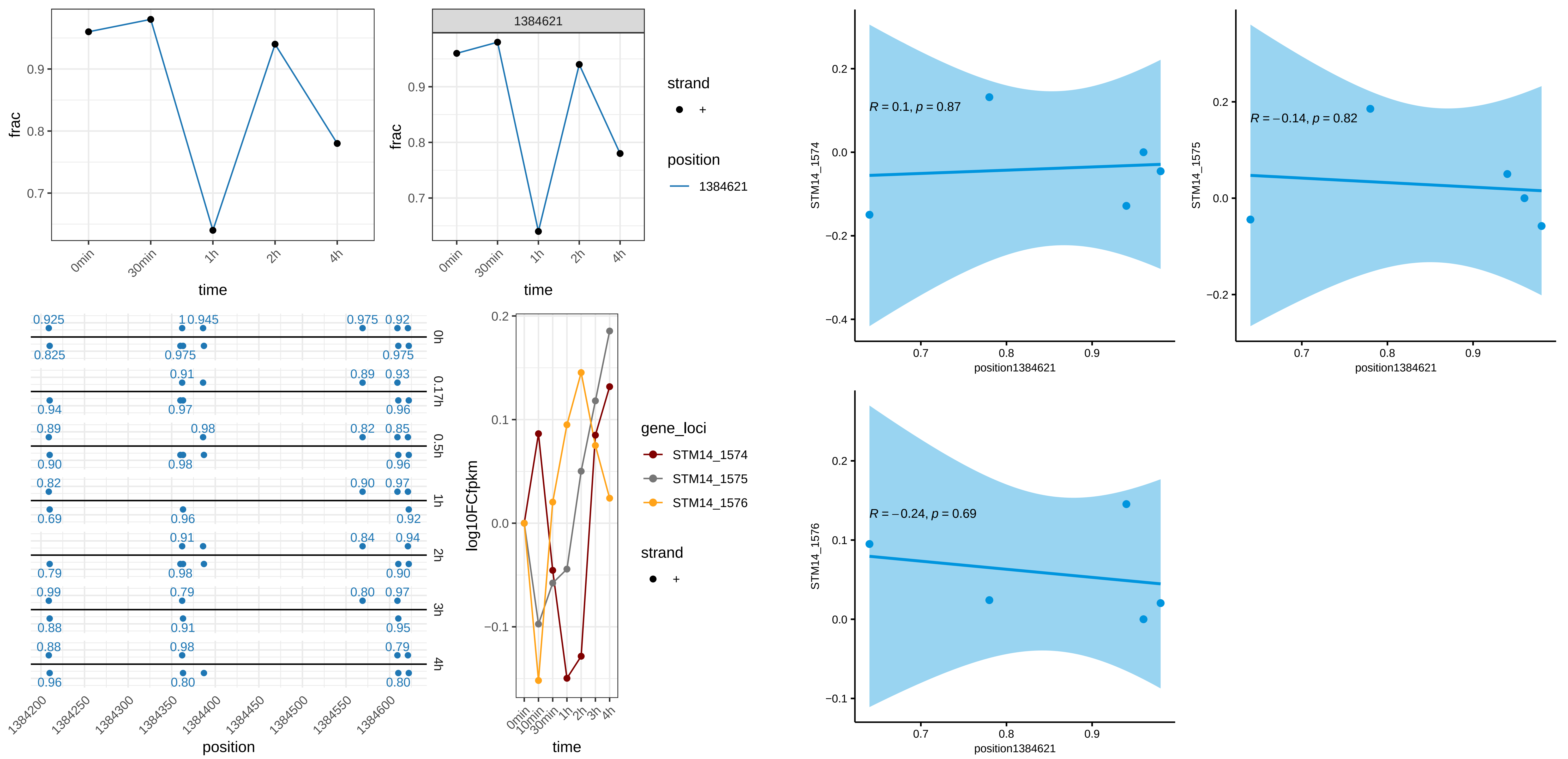

frac
time
0min
30min
1h
2h
4h
1384621
strand
+
position
1384621
R = 0.1, p = 0.87
STM14_1574
position1384621
R = −0.14, p = 0.82
STM14_1575
R = −0.24, p = 0.69
STM14_1576
gene_loci
STM14_1574
STM14_1575
STM14_1576
log10FCfpkm
10min
3h
position
0h
0.17h
0.5h

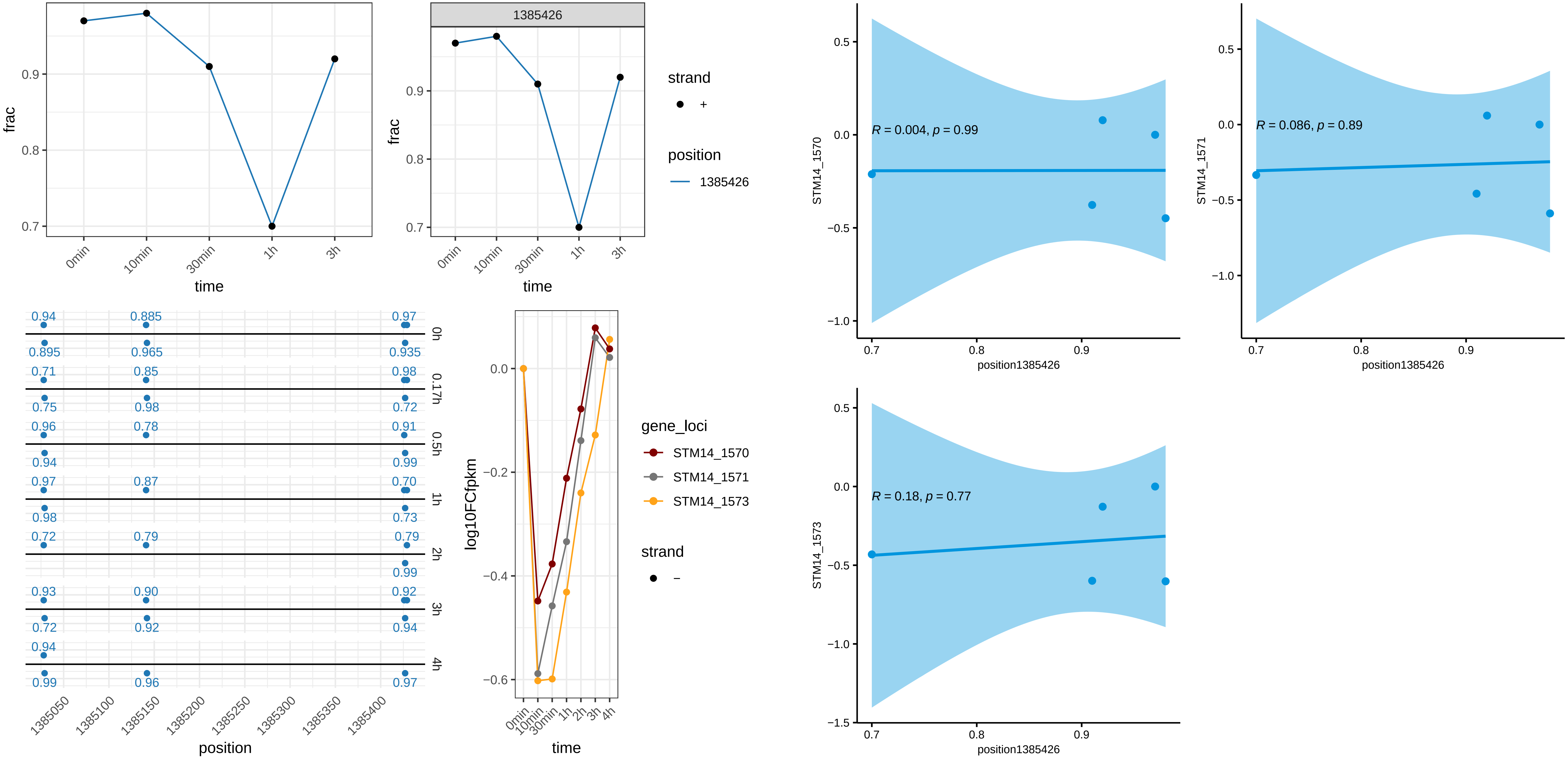

frac
time
0min
10min
30min
1h
3h
1385426
strand
+
position
1385426
0.94
0.885
0.97
0.895
0.965
0.935
0.71
0.85
0.98
0.75
0.98
0.72
0.96
0.78
0.91
0.94
0.99
0.97
0.87
0.70
0.98
0.73
0.72
0.79
0.79
0.99
0.93
0.90
0.92
0.72
0.92
0.94
0.94
0.99
0.96
0.97
0h
0.17h
0.5h
1h
2h
3h
4h
1385050
1385100
1385150
1385200
1385250
1385300
1385350
1385400
position
log10FCfpkm
2h
4h
gene_loci
STM14_1570
STM14_1571
STM14_1573
strand
–
R = 0.004, p = 0.99
STM14_1570
position1385426
R = 0.086, p = 0.89
STM14_1571
R = 0.18, p = 0.77
STM14_1573

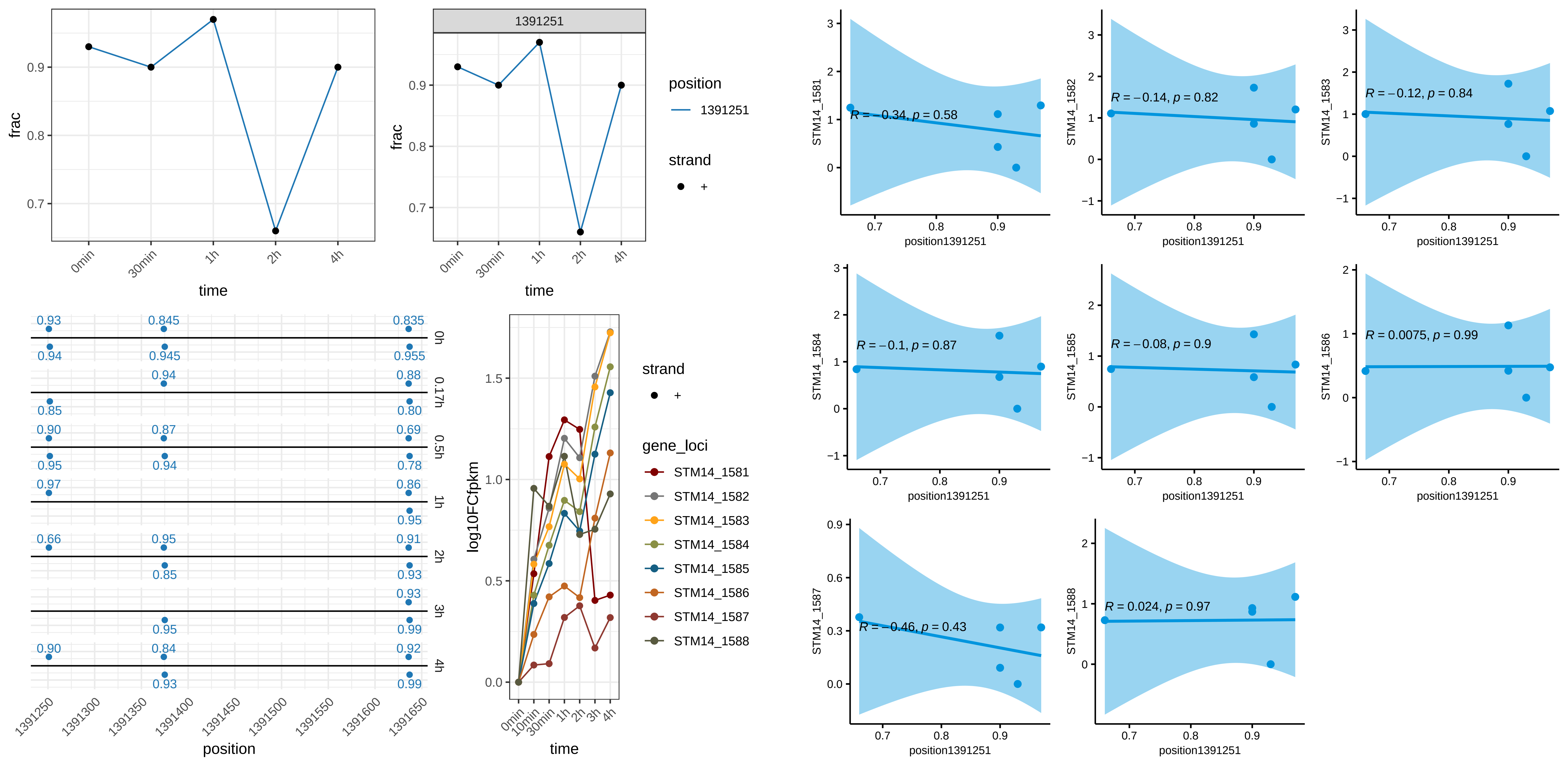

frac
time
0min
30min
1h
2h
4h
1391251
position
strand
+
position
log10FCfpkm
gene_loci
STM14_1581
STM14_1582
STM14_1583
STM14_1584
STM14_1585
STM14_1586
STM14_1587
STM14_1588
0h
0.17h
0.5h
3h
10min
position1391251
R = −0.34, p = 0.58
R = −0.14, p = 0.82
R = −0.12, p = 0.84
R = −0.1, p = 0.87
R = −0.08, p = 0.9
R = 0.0075, p = 0.99
R = −0.46, p = 0.43
R = 0.024, p = 0.97

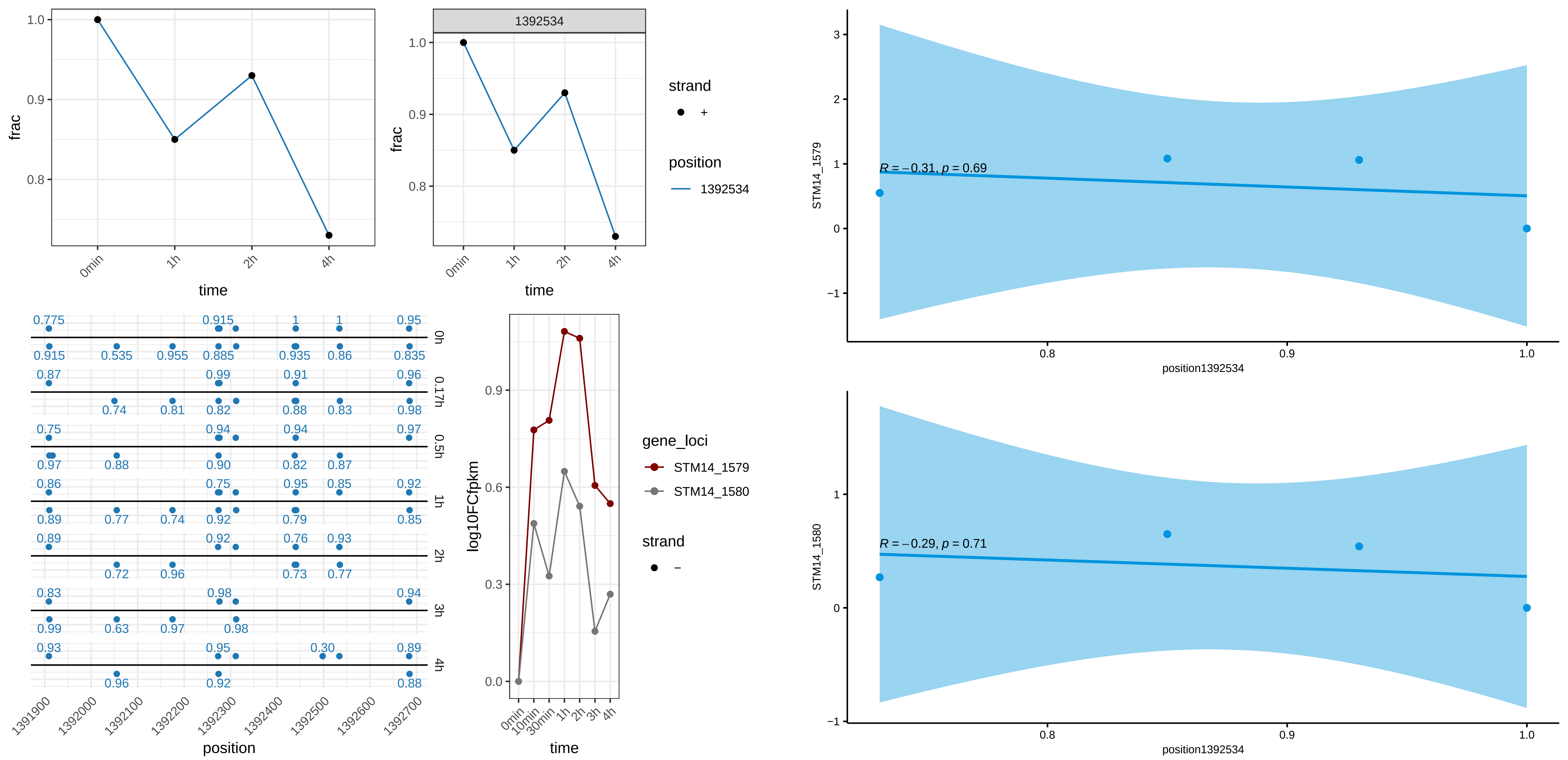

frac
time
0min
1h
2h
4h
1392534
strand
+
position
1392534
position
0h
0.17h
0.5h
1h
2h
3h
4h
log10FCfpkm
0min
10min
30min
3h
gene_loci
STM14_1579
STM14_1580
strand
–
STM14_1579
R = −0.31, p = 0.69
position1392534
STM14_1580
R = −0.29, p = 0.71
position1392534

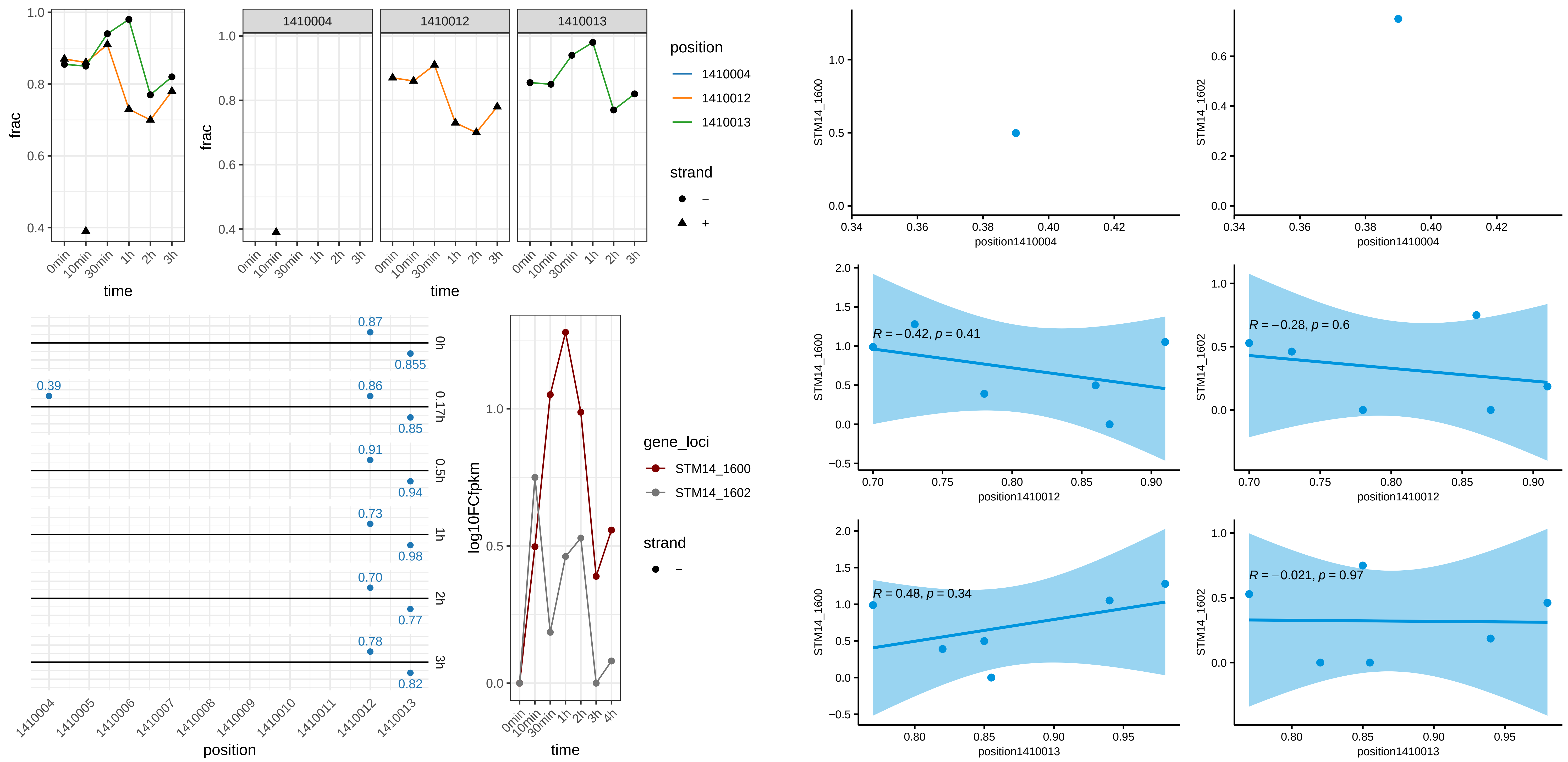
frac
time
1410004
1410012
1410013
position
strand
R = −0.42, p = 0.41
R = −0.28, p = 0.6
R = 0.48, p = 0.34
R = −0.021, p = 0.97
STM14_1600
STM14_1602
position1410004
position1410012
position1410013
log10FCfpkm
gene_loci
0h
0.17h
0.5h
1h
2h
3h

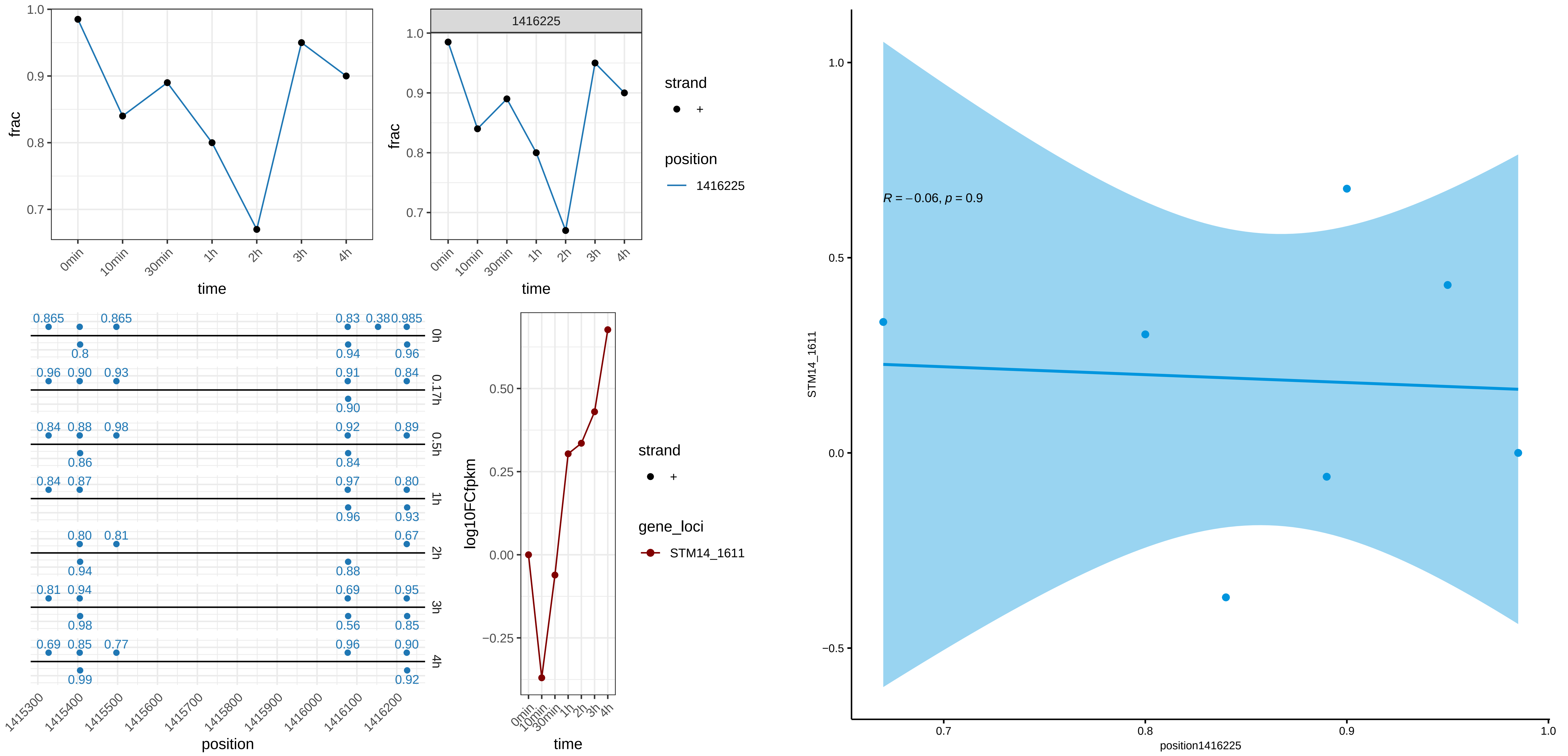
frac
time
0min
10min
30min
1h
2h
3h
4h
1416225
strand
+
position
1416225
0h
0.17h
0.5h
1h
2h
3h
4h
0.865
0.865
0.83
0.38
0.985
0.8
0.94
0.96
0.96
0.90
0.93
0.91
0.84
0.90
0.84
0.88
0.98
0.92
0.89
0.86
0.84
0.84
0.87
0.97
0.80
0.96
0.93
0.80
0.81
0.67
0.94
0.88
0.81
0.94
0.69
0.95
0.98
0.56
0.85
0.69
0.85
0.77
0.96
0.90
0.99
0.92
position
log10FCfpkm
gene_loci
STM14_1611
R = −0.06, p = 0.9
STM14_1611
position1416225

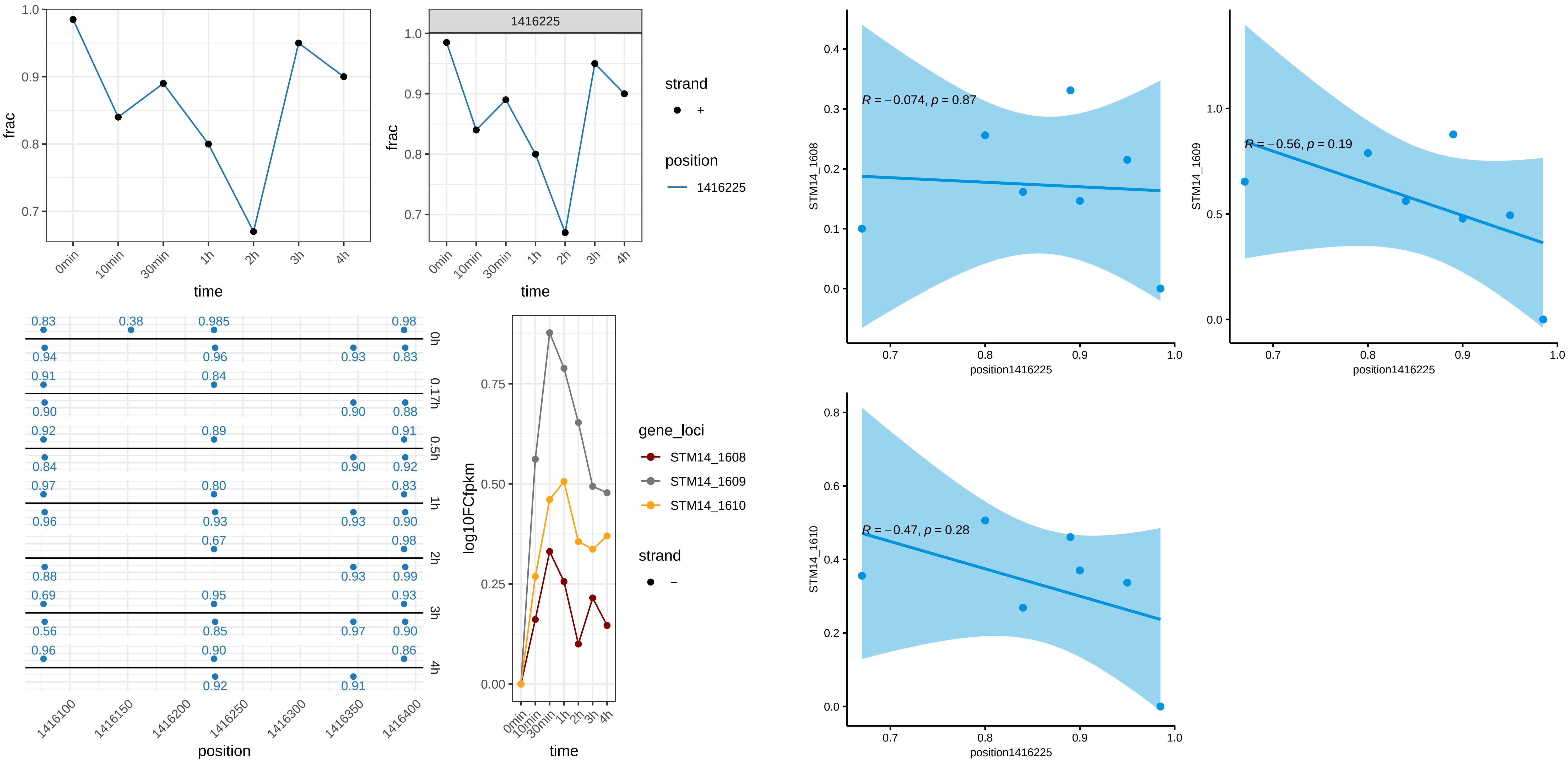
frac
time
0min
10min
30min
1h
2h
3h
4h
1416225
strand
+
position
1416225
0.83
0.38
0.985
0.98
0.94
0.96
0.93
0.83
0.91
0.84
0.90
0.90
0.88
0.92
0.89
0.91
0.84
0.90
0.92
0.97
0.80
0.83
0.96
0.93
0.93
0.90
0.67
0.98
0.88
0.93
0.99
0.69
0.95
0.93
0.56
0.85
0.97
0.90
0.96
0.90
0.86
0.92
0.91
0h
0.17h
0.5h
1h
2h
3h
4h
position
log10FCfpkm
gene_loci
STM14_1608
STM14_1609
STM14_1610
strand
–
R = –0.074, p = 0.87
STM14_1608
position1416225
R = –0.56, p = 0.19
STM14_1609
position1416225
R = –0.47, p = 0.28
STM14_1610
position1416225

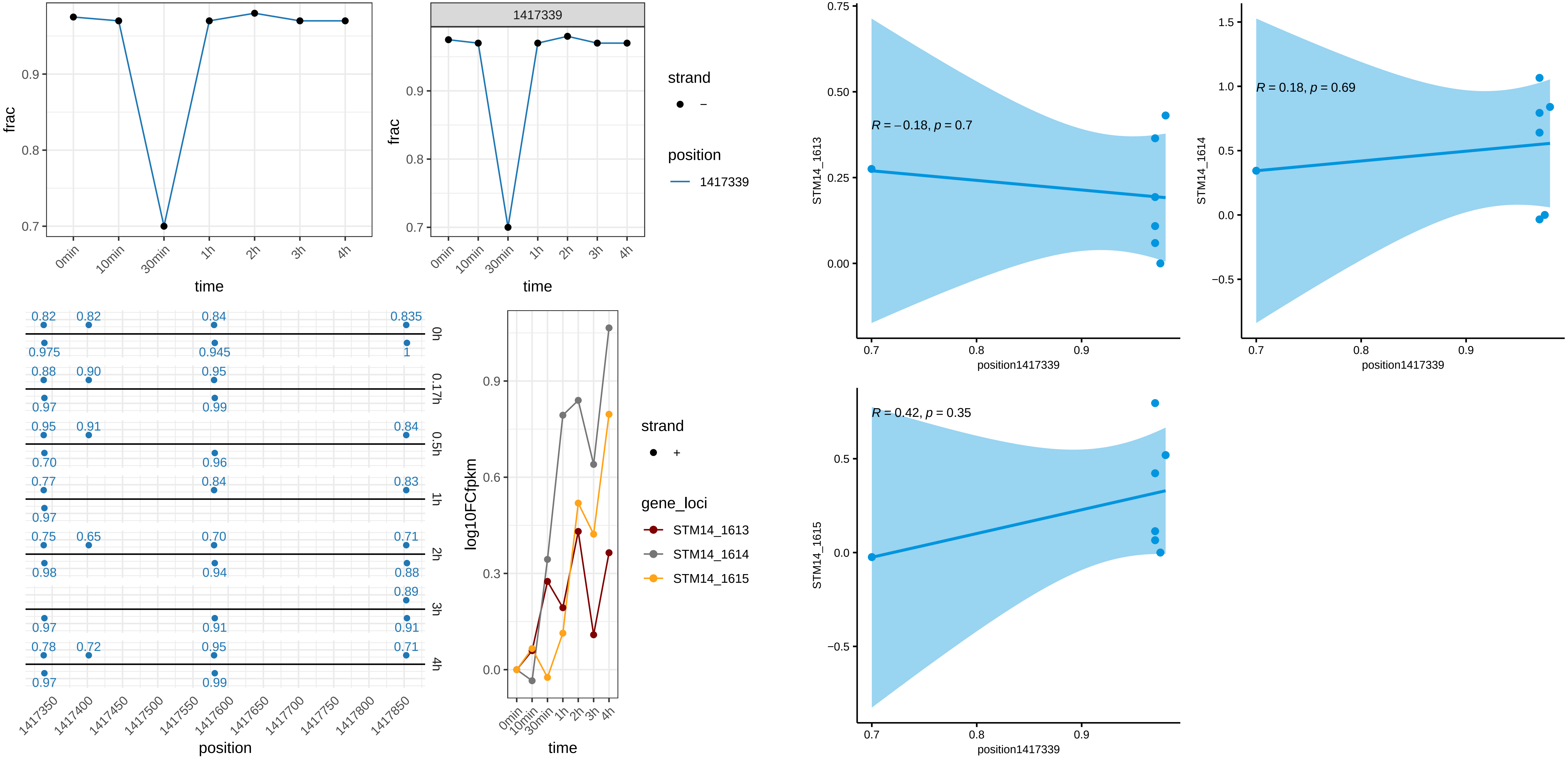

frac
time
0min
10min
30min
1h
2h
3h
4h
1417339
strand
–
position
1417339
0h
0.17h
0.5h
1h
2h
3h
4h
0.82
0.82
0.84
0.835
0.975
0.945
1
0.88
0.90
0.95
0.97
0.99
0.95
0.91
0.84
0.70
0.96
0.77
0.84
0.83
0.97
0.75
0.65
0.70
0.71
0.98
0.94
0.88
0.89
0.97
0.91
0.91
0.78
0.72
0.95
0.71
0.97
0.99
position
log10FCfpkm
strand
+
gene_loci
STM14_1613
STM14_1614
STM14_1615
R = −0.18, p = 0.7
STM14_1613
position1417339
R = 0.18, p = 0.69
STM14_1614
position1417339
R = 0.42, p = 0.35
STM14_1615
position1417339

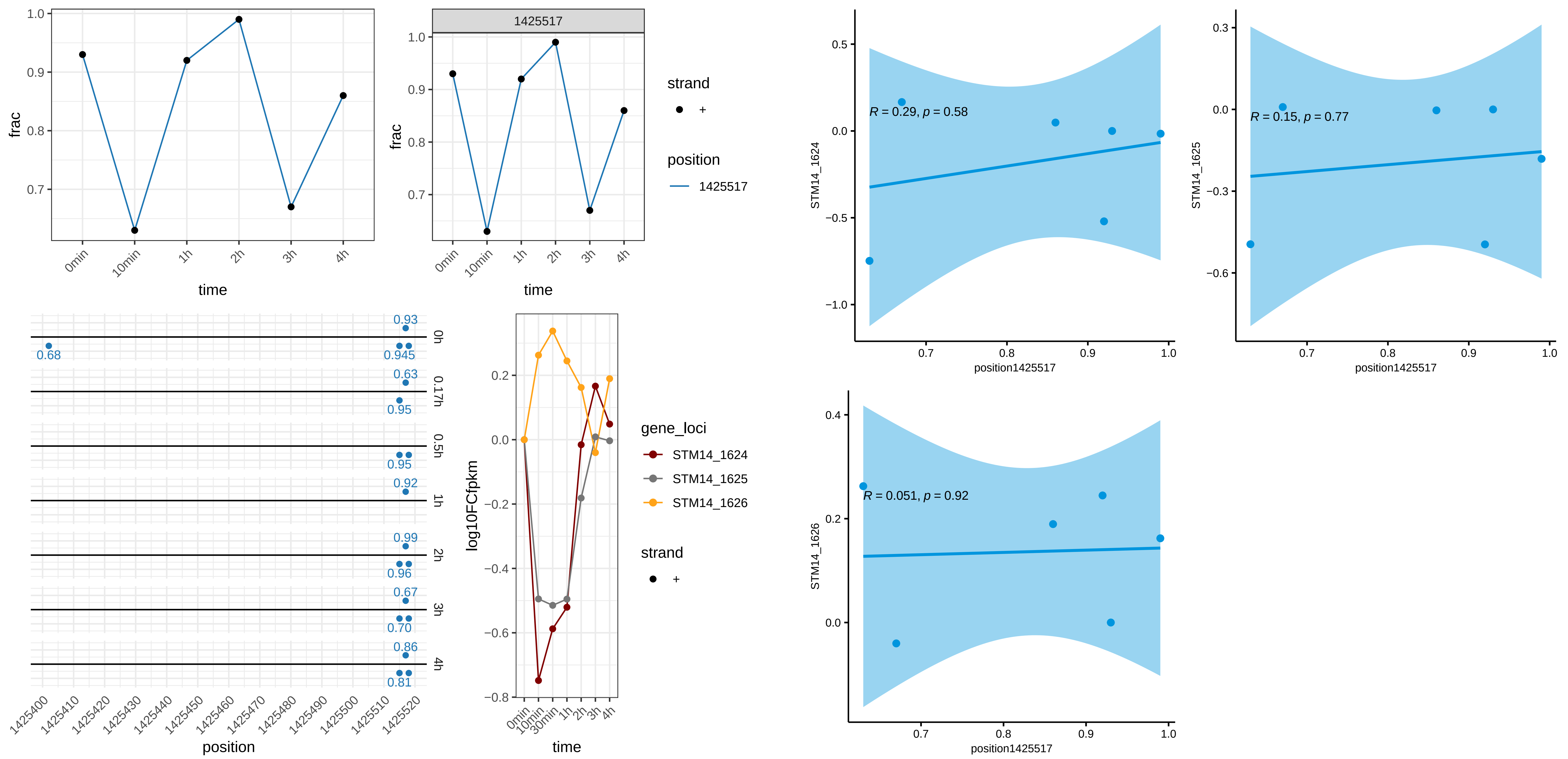

frac
time
0min
10min
1h
2h
3h
4h
1425517
strand
+
position
1425517
STM14_1624
position1425517
R = 0.29, p = 0.58
STM14_1625
R = 0.15, p = 0.77
STM14_1626
R = 0.051, p = 0.92
position
0h
0.17h
0.5h
0.93
0.68
0.945
0.63
0.95
0.92
0.99
0.96
0.67
0.70
0.86
0.81
log10FCfpkm
30min
gene_loci

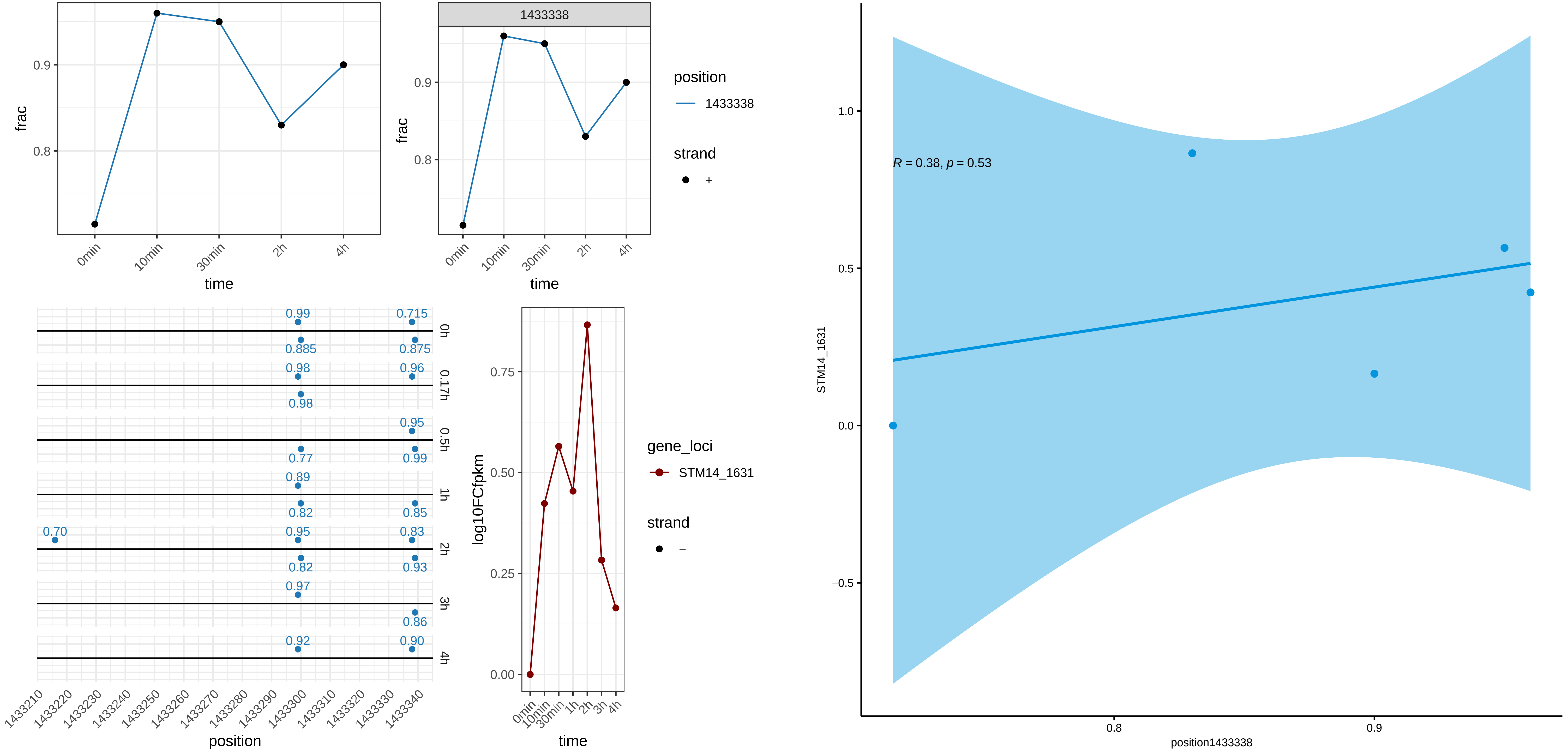
frac
0.9
0.8
0min
10min
30min
2h
4h
time
1433338
position
1433338
strand
+
0.99
0.715
0.885
0.875
0.98
0.96
0.98
0.95
0.77
0.99
0.89
0.82
0.85
0.70
0.95
0.83
0.82
0.93
0.97
0.86
0.92
0.90
0h
0.17h
0.5h
1h
2h
3h
4h
1433210
1433220
1433230
1433240
1433250
1433260
1433270
1433280
1433290
1433300
1433310
1433320
1433330
1433340
position
log10FCfpkm
0.75
0.50
0.25
0.00
0min
10min
30min
1h
2h
3h
4h
time
gene_loci
STM14_1631
strand
–
R = 0.38, p = 0.53
STM14_1631
1.0
0.5
0.0
−0.5
0.8
0.9
position1433338

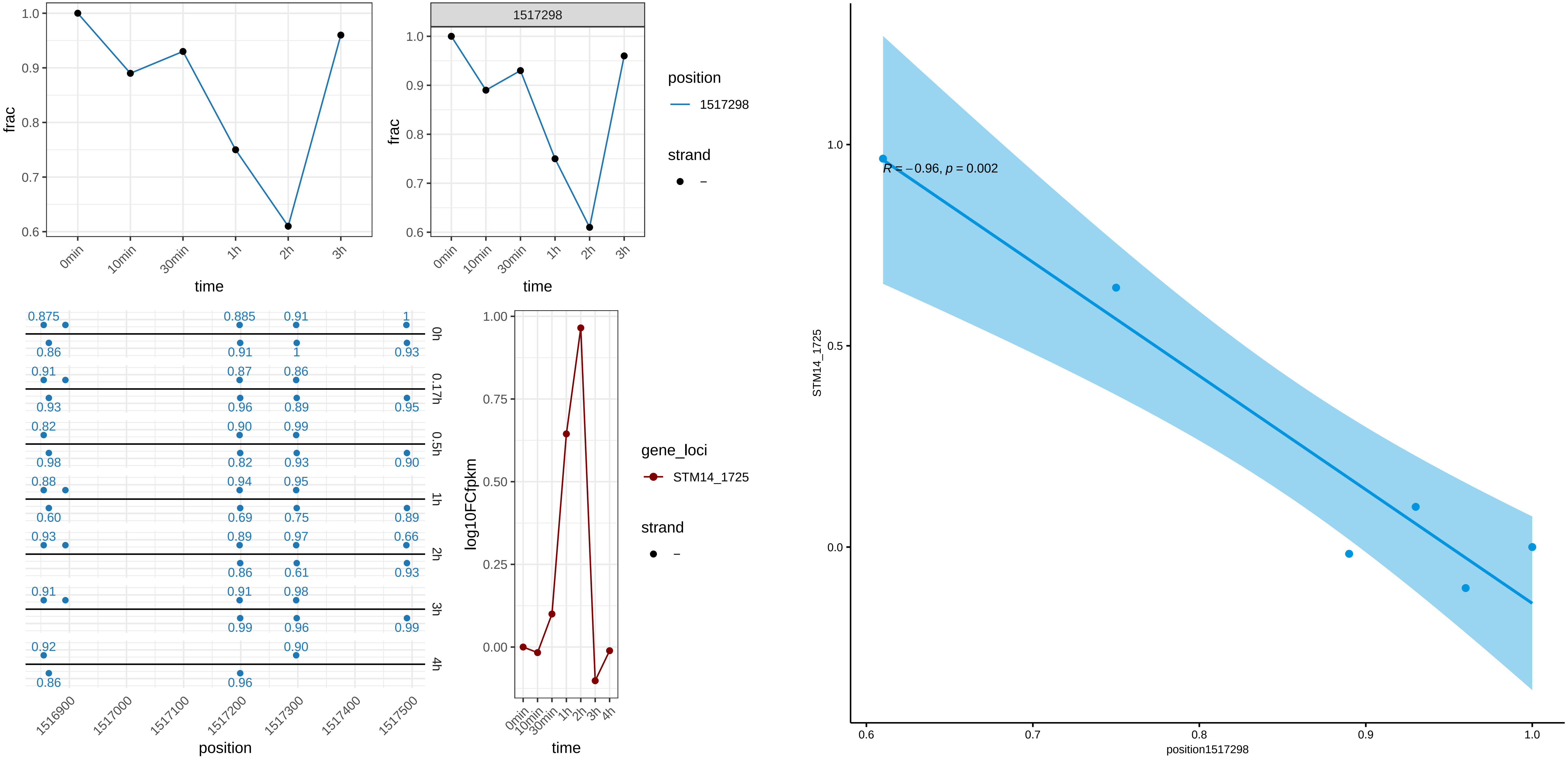

frac
time
0min
10min
30min
1h
2h
3h
1517298
position
strand
−
position
0h
0.17h
0.5h
1h
2h
3h
4h
1516900
1517000
1517100
1517200
1517300
1517400
1517500
log10FCfpkm
4h
gene_loci
STM14_1725
R = −0.96, p = 0.002
STM14_1725
position1517298

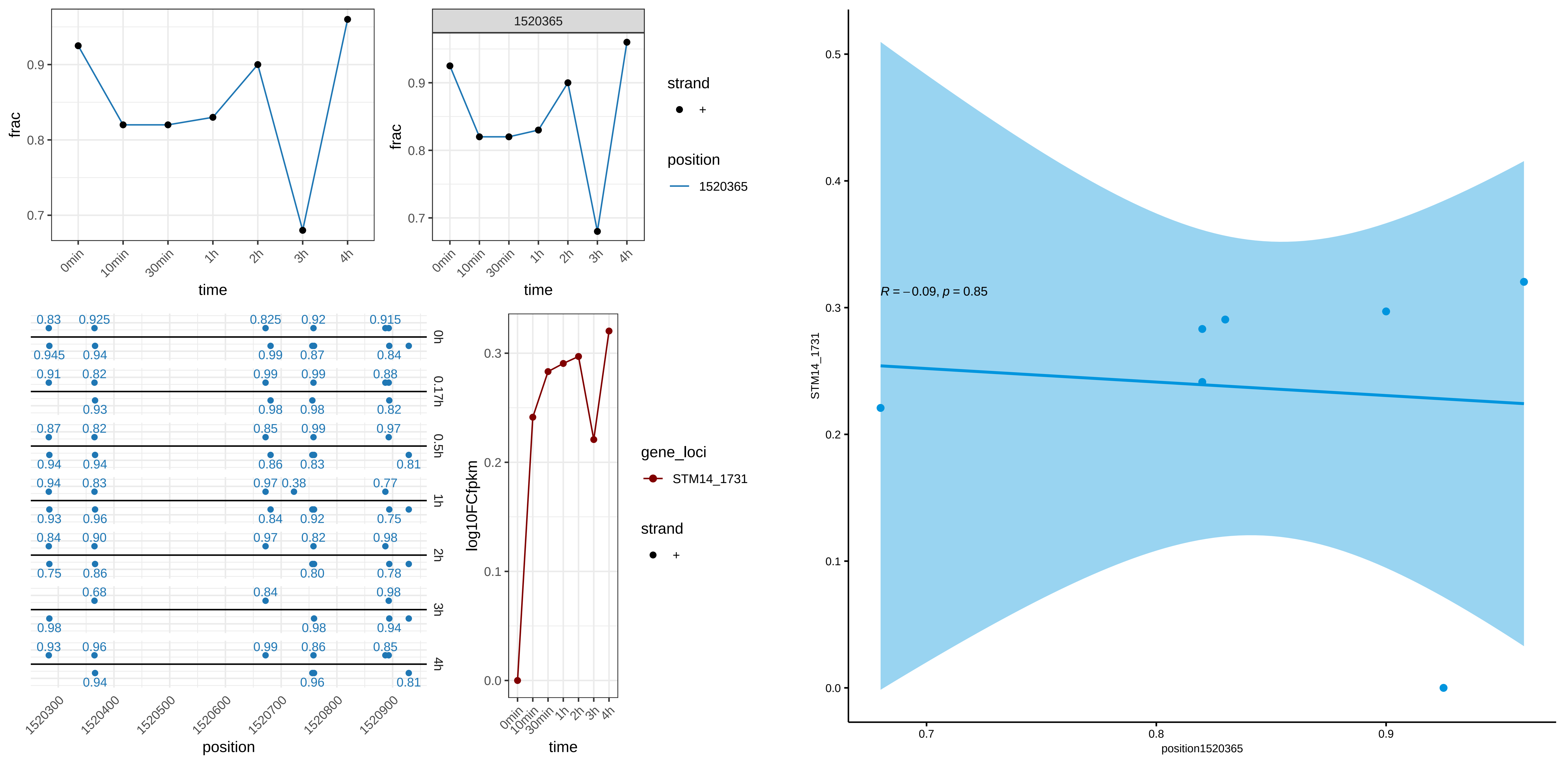

frac
time
0min
10min
30min
1h
2h
3h
4h
1520365
strand
+
position
1520365
log10FCfpkm
gene_loci
STM14_1731
position
0h
0.17h
0.5h
1h
2h
3h
4h
R = −0.09, p = 0.85
STM14_1731
position1520365

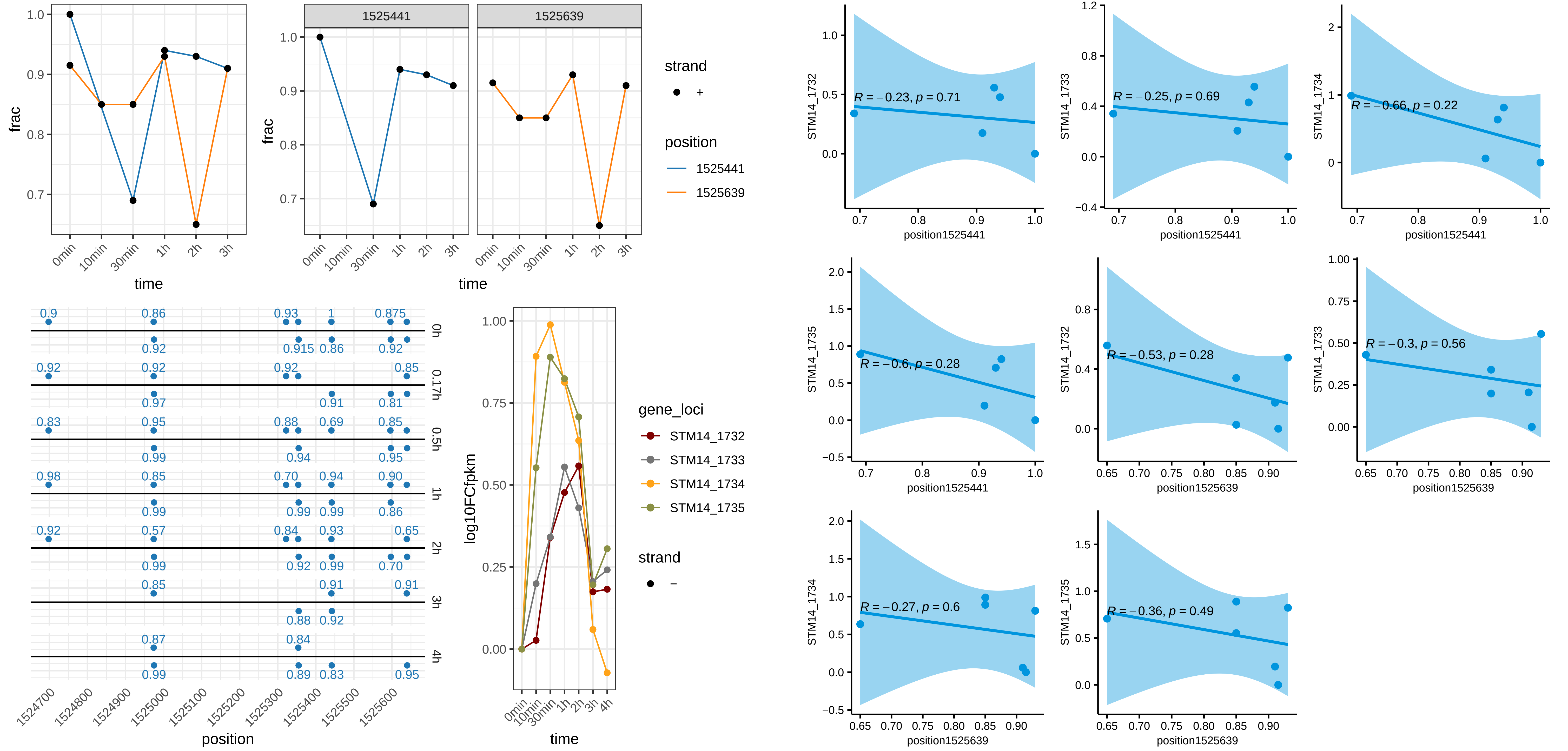

frac
time
0min
10min
30min
1h
2h
3h
1525441
1525639
strand
+
position
1525441
1525639
0.9
0.86
0.93
1
0.875
0.92
0.915
0.86
0.92
0.92
0.92
0.92
0.85
0.97
0.91
0.81
0.83
0.95
0.88
0.69
0.85
0.99
0.94
0.95
0.98
0.85
0.70
0.94
0.90
0.99
0.99
0.99
0.86
0.92
0.57
0.84
0.93
0.65
0.99
0.92
0.99
0.70
0.85
0.91
0.91
0.88
0.92
0.87
0.84
0.99
0.89
0.83
0.95
0h
0.17h
0.5h
1h
2h
3h
4h
position
log10FCfpkm
time
4h
gene_loci
STM14_1732
STM14_1733
STM14_1734
STM14_1735
strand
–
STM14_1732
R = −0.23, p = 0.71
position1525441
STM14_1733
R = −0.25, p = 0.69
position1525441
STM14_1734
R = −0.66, p = 0.22
position1525441
STM14_1735
R = −0.6, p = 0.28
position1525441
STM14_1732
R = −0.53, p = 0.28
position1525639
STM14_1733
R = −0.3, p = 0.56
position1525639
STM14_1734
R = −0.27, p = 0.6
position1525639
STM14_1735
R = −0.36, p = 0.49
position1525639

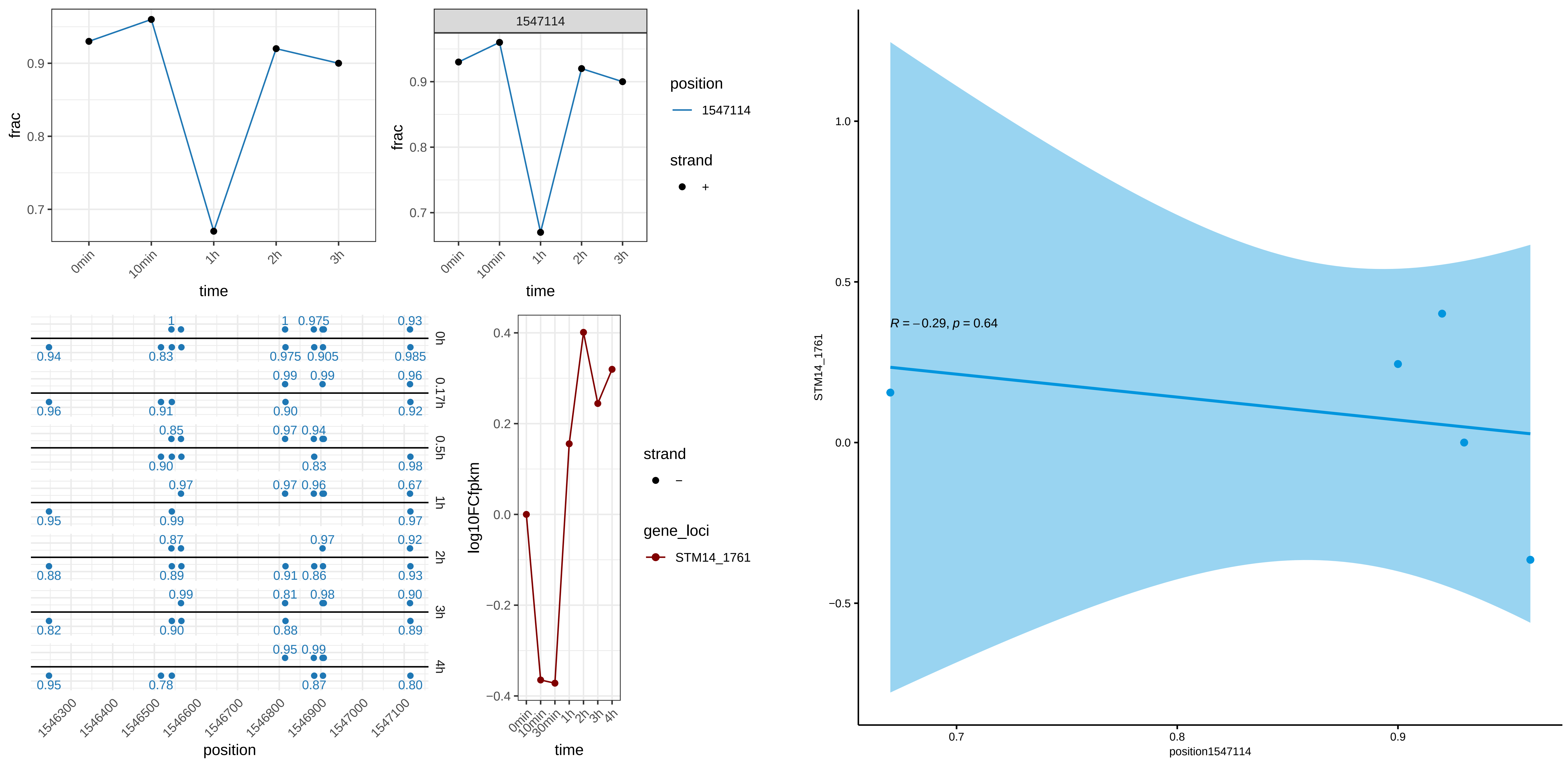

frac
time
0min
10min
1h
2h
3h
1547114
position
strand
+
position
0h
0.17h
0.5h
1h
2h
3h
4h
log10FCfpkm
0min
10min
30min
4h
–
gene_loci
STM14_1761
R = −0.29, p = 0.64
position1547114

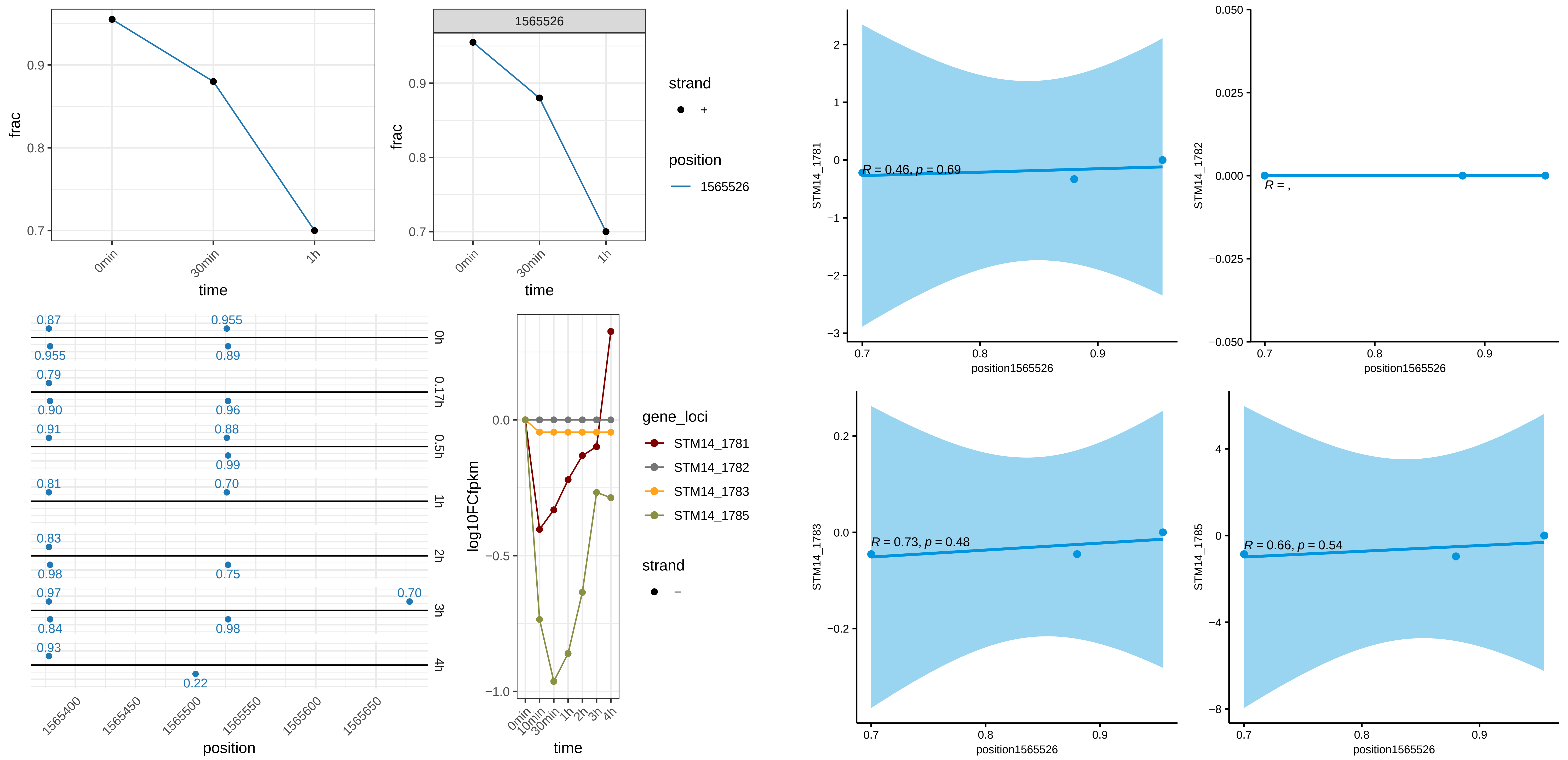

frac
time
0min
30min
1h
1565526
strand
+
position
1565526
0h
0.17h
0.5h
1h
2h
3h
4h
position
1565400
1565450
1565500
1565550
1565600
1565650
log10FCfpkm
10min
2h
3h
4h
gene_loci
STM14_1781
STM14_1782
STM14_1783
STM14_1785
strand
–
R = 0.46, p = 0.69
R = ,
R = 0.73, p = 0.48
R = 0.66, p = 0.54
position1565526

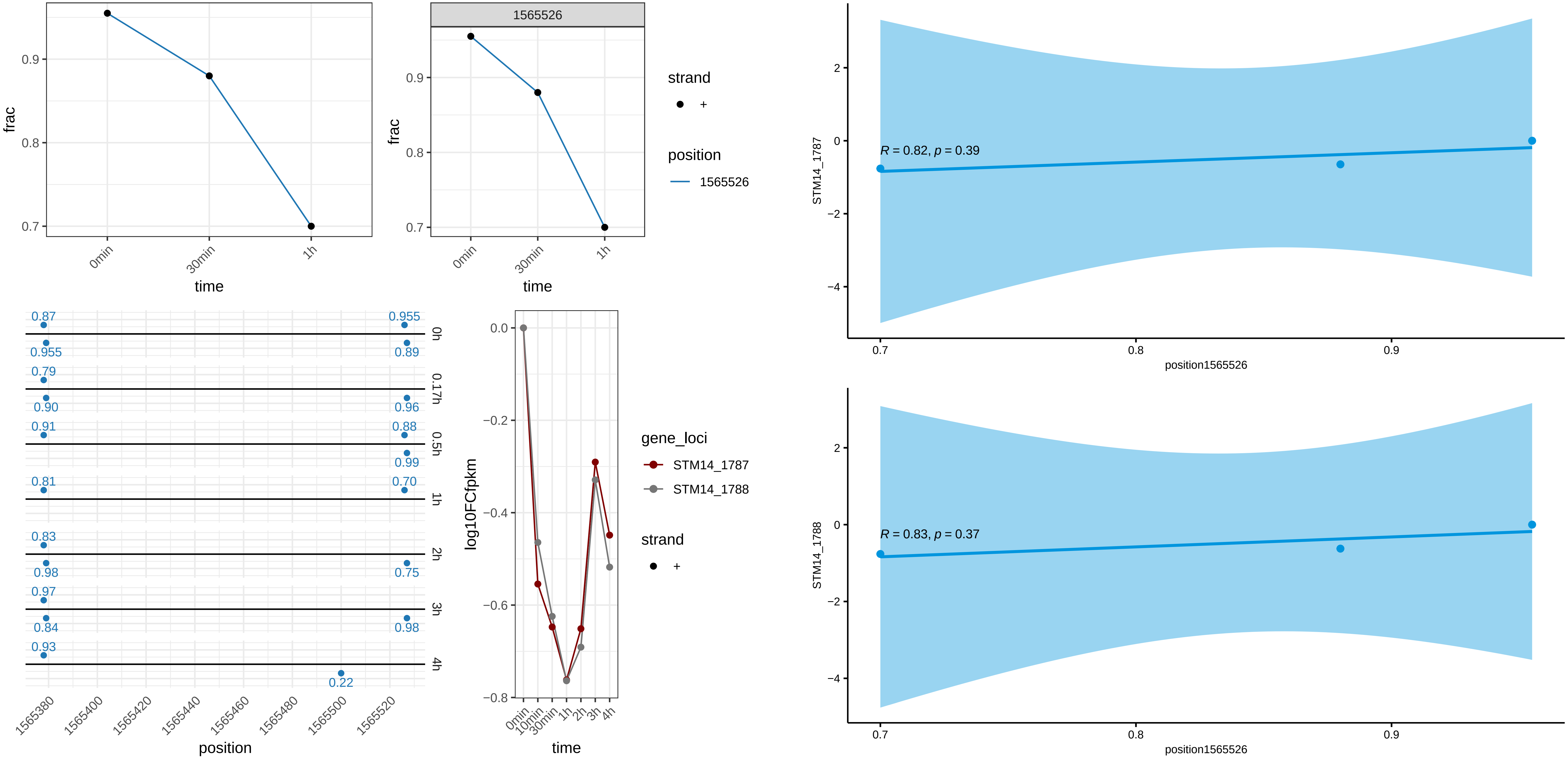

frac
time
0min
30min
1h
1565526
strand
+
position
1565526
0.87
0.955
0.955
0.89
0.79
0.96
0.90
0.91
0.88
0.99
0.81
0.70
0.83
0.98
0.75
0.97
0.84
0.98
0.93
0.22
0h
0.17h
0.5h
1h
2h
3h
4h
position
log10FCfpkm
10min
2h
3h
4h
gene_loci
STM14_1787
STM14_1788
STM14_1787
STM14_1788
R = 0.82, p = 0.39
R = 0.83, p = 0.37
position1565526
position1565526

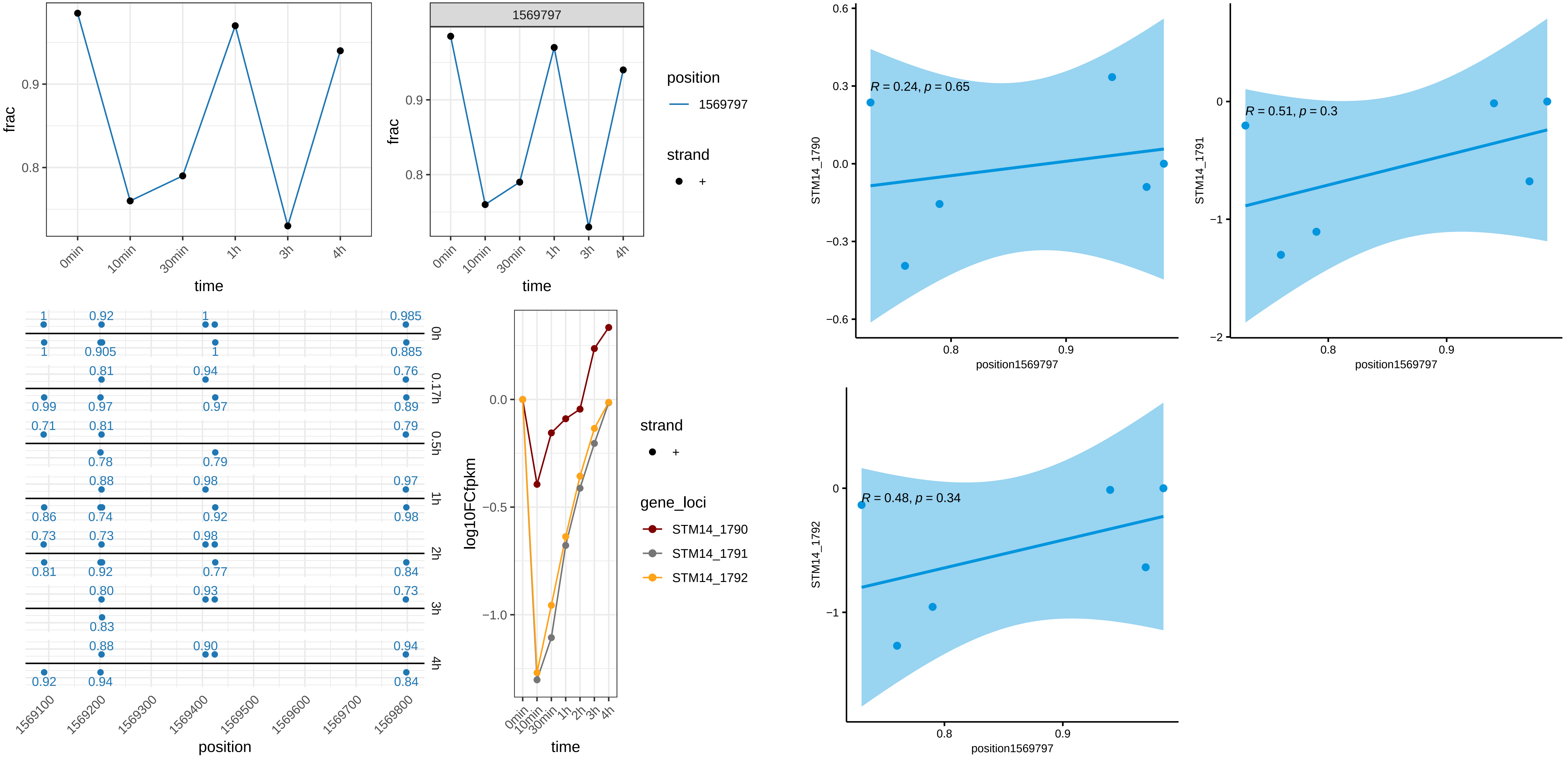

frac
time
0min
10min
30min
1h
3h
4h
0.9
0.8
1569797
position
strand
+
1
0.92
0.985
0.905
0.885
0.81
0.94
0.76
0.99
0.97
0.89
0.71
0.79
0.78
0.88
0.98
0.86
0.74
0.73
0.77
0.84
0.80
0.93
0.83
0.90
0.92
0h
0.17h
0.5h
2h
1569100
1569200
1569300
1569400
1569500
1569600
1569700
1569800
log10FCfpkm
gene_loci
STM14_1790
STM14_1791
STM14_1792
R = 0.24, p = 0.65
R = 0.51, p = 0.3
R = 0.48, p = 0.34
position1569797

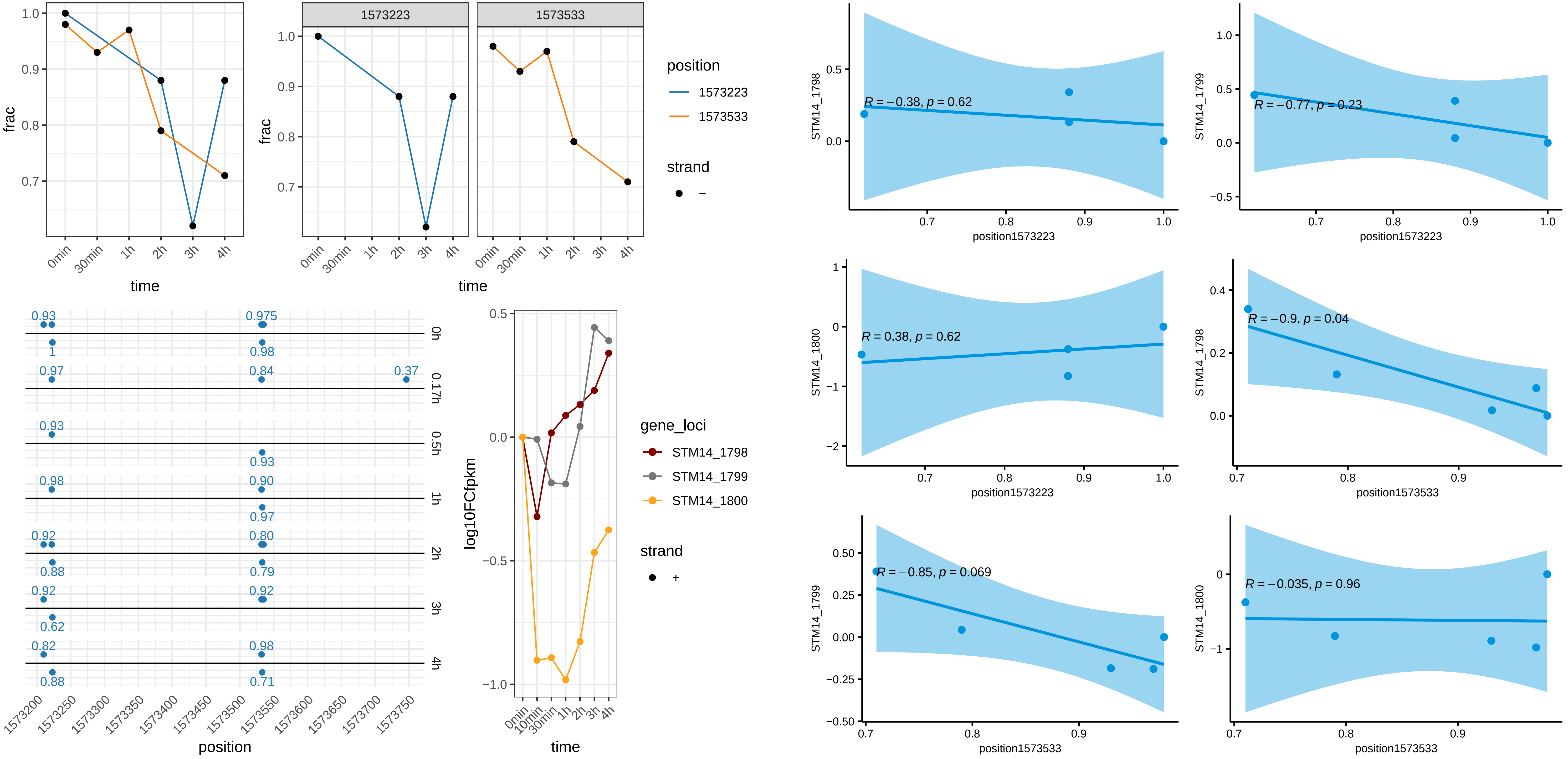

frac
time
0min
30min
1h
2h
3h
4h
1573223
1573533
position
strand
gene_loci
STM14_1798
STM14_1799
STM14_1800
log10FCfpkm
10min
0h
0.17h
0.5h
$R = -0.38, p = 0.62$
$R = -0.77, p = 0.23$
$R = 0.38, p = 0.62$
$R = -0.9, p = 0.04$
$R = -0.85, p = 0.069$
$R = -0.035, p = 0.96$
position1573223
position1573533

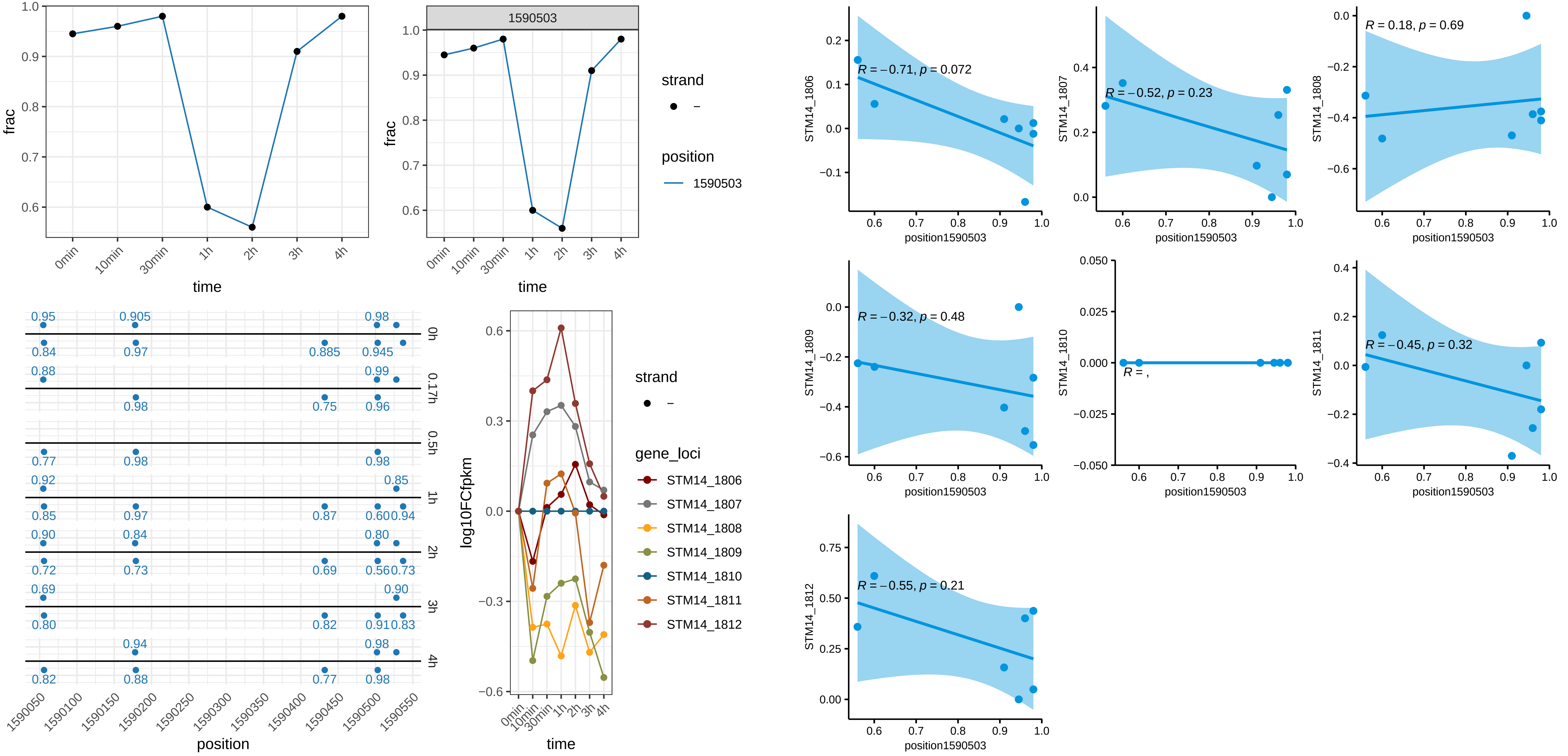

frac
time
0min
10min
30min
1h
2h
3h
4h
1590503
strand
–
position
1590503
position
1590050
1590100
1590150
1590200
1590250
1590300
1590350
1590400
1590450
1590500
1590550
0h
0.17h
0.5h
1h
2h
3h
4h
log10FCfpkm
gene_loci
STM14_1806
STM14_1807
STM14_1808
STM14_1809
STM14_1810
STM14_1811
STM14_1812
R = –0.71, p = 0.072
R = –0.52, p = 0.23
R = 0.18, p = 0.69
R = –0.32, p = 0.48
R = ,
R = –0.45, p = 0.32
R = –0.55, p = 0.21
position1590503

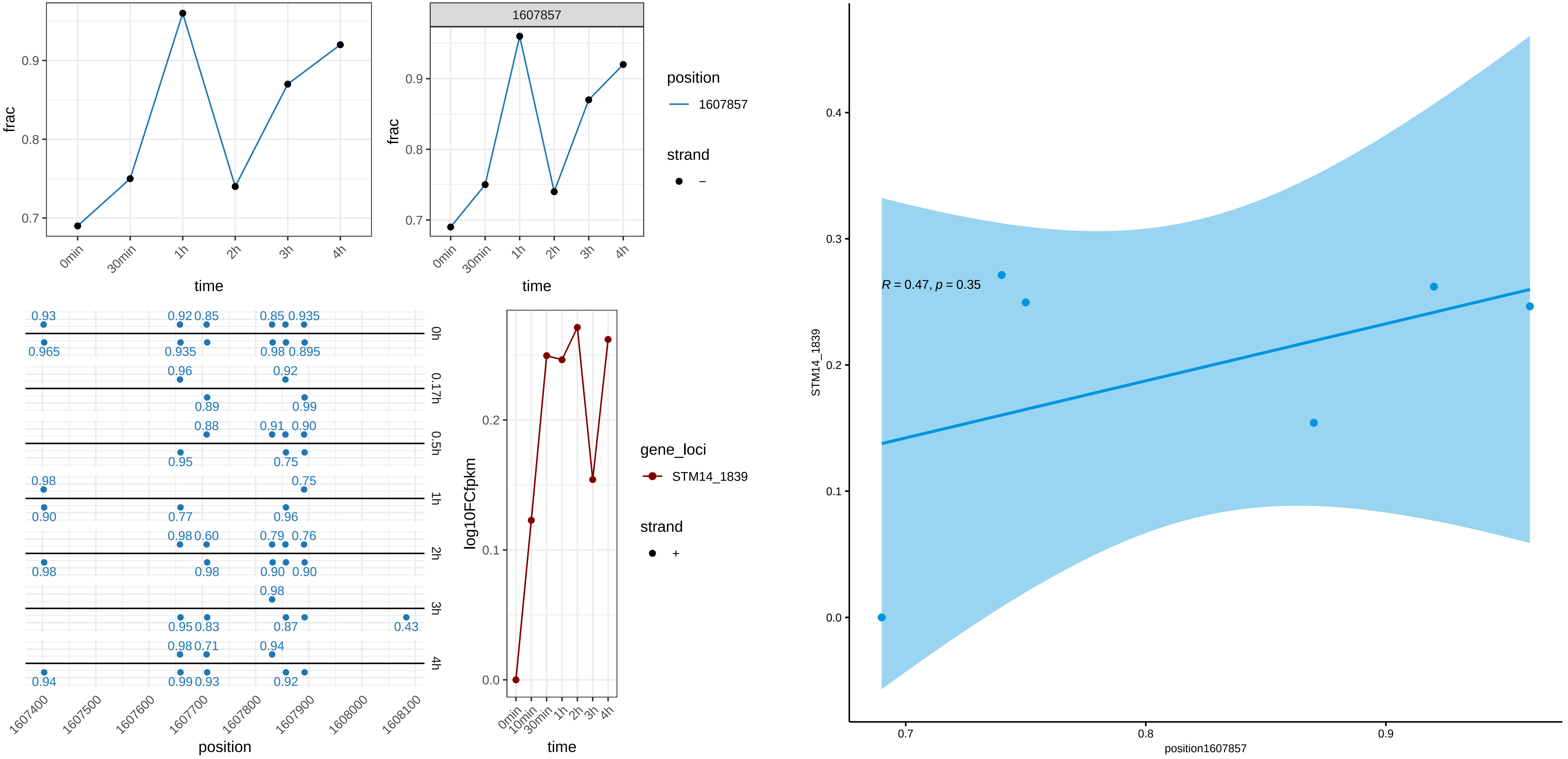

frac
time
0min
30min
1h
2h
3h
4h
1607857
position
strand
−
0h
0.17h
0.5h
1h
2h
3h
4h
1607400
1607500
1607600
1607700
1607800
1607900
1608000
1608100
log10FCfpkm
gene_loci
STM14_1839
+
R = 0.47, p = 0.35
STM14_1839
position1607857

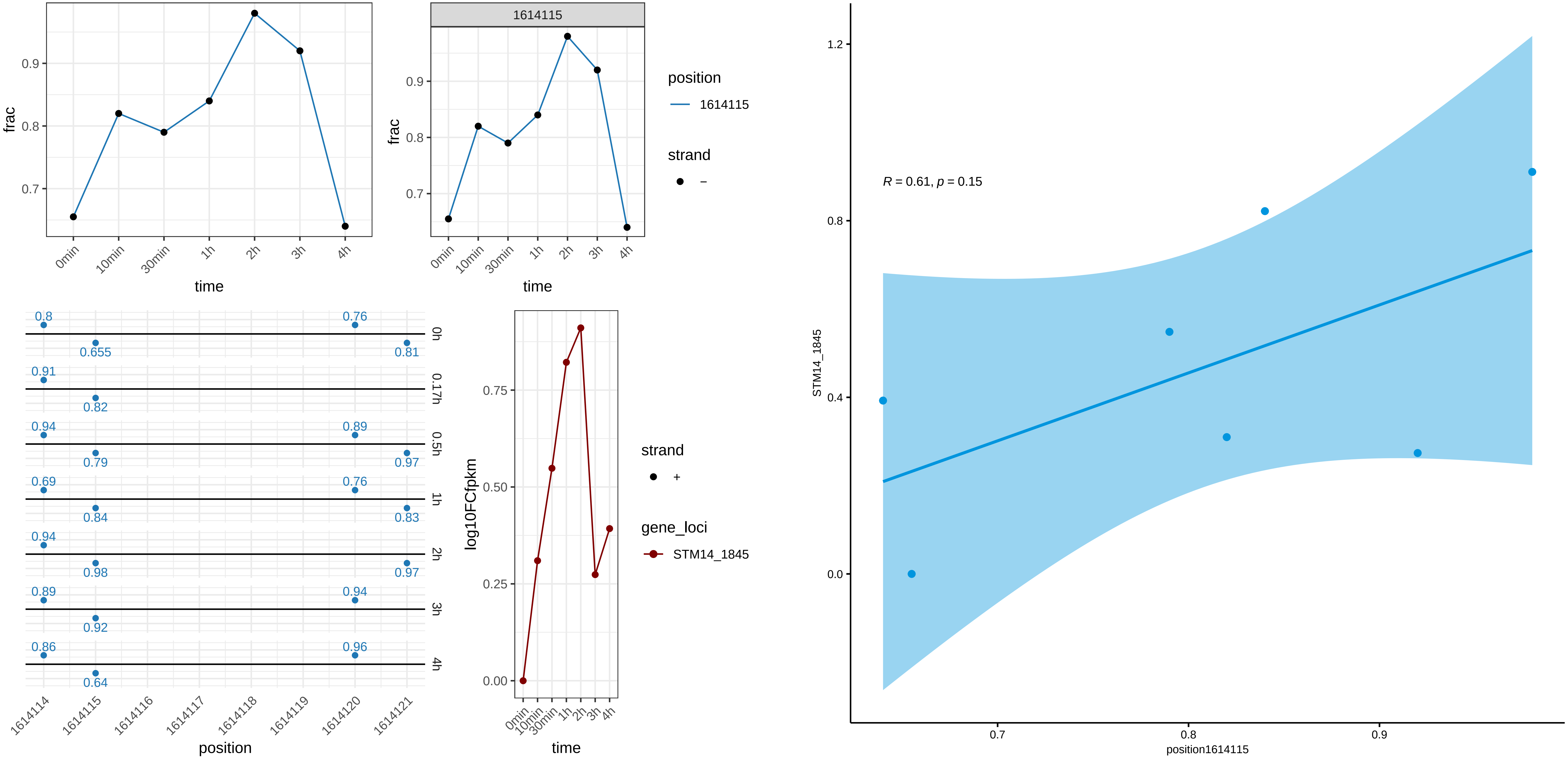
frac
time
1614115
position
strand
log10FCfpkm
gene_loci
STM14_1845
R = 0.61, p = 0.15
STM14_1845
position1614115

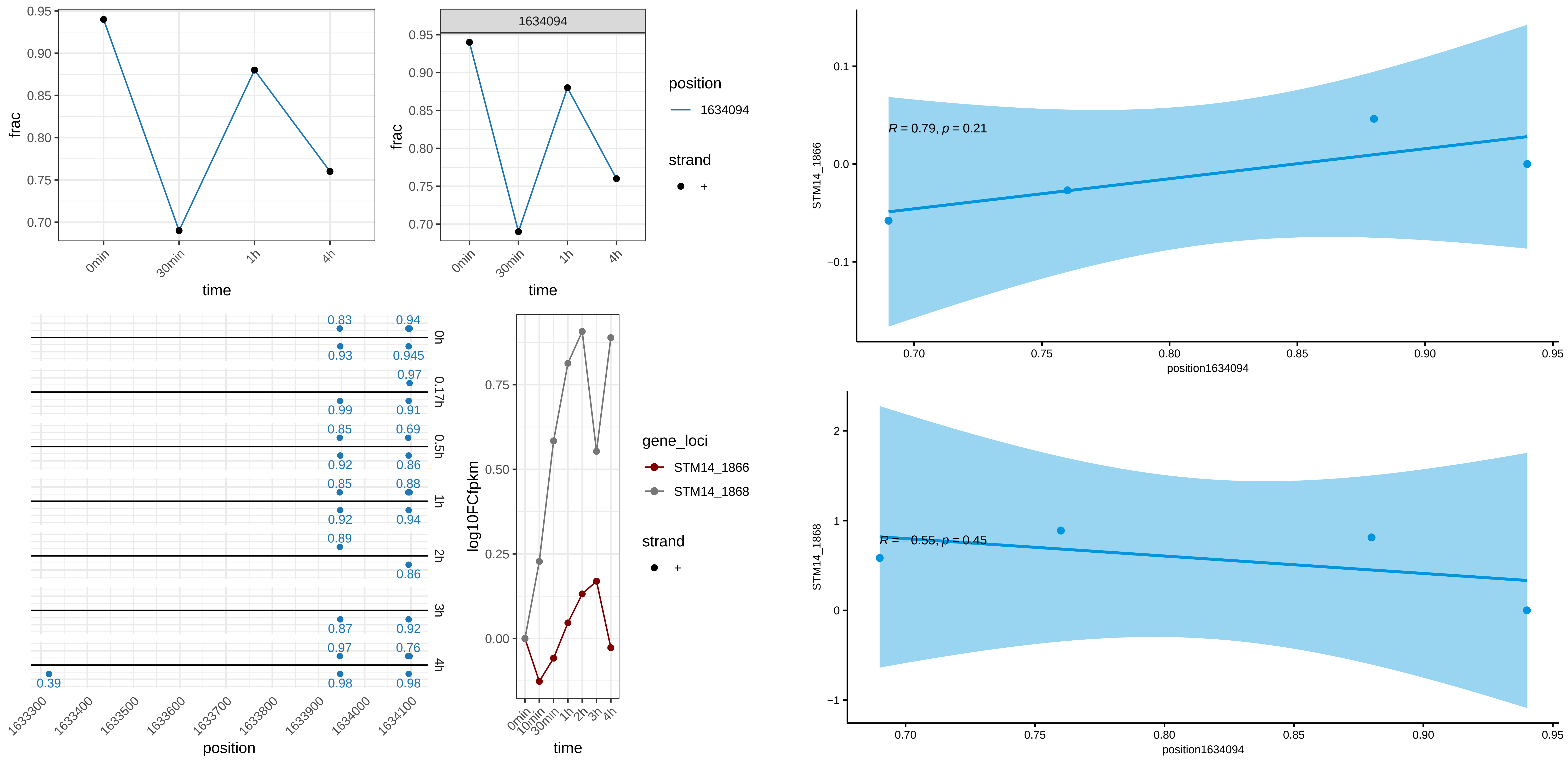

frac
time
0min
30min
1h
4h
1634094
position
strand
+
position
0h
0.17h
0.5h
1h
2h
3h
4h
log10FCfpkm
0min
10min
30min
2h
3h
gene_loci
STM14_1866
STM14_1868
R = 0.79, p = 0.21
STM14_1866
position1634094
R = −0.55, p = 0.45
STM14_1868

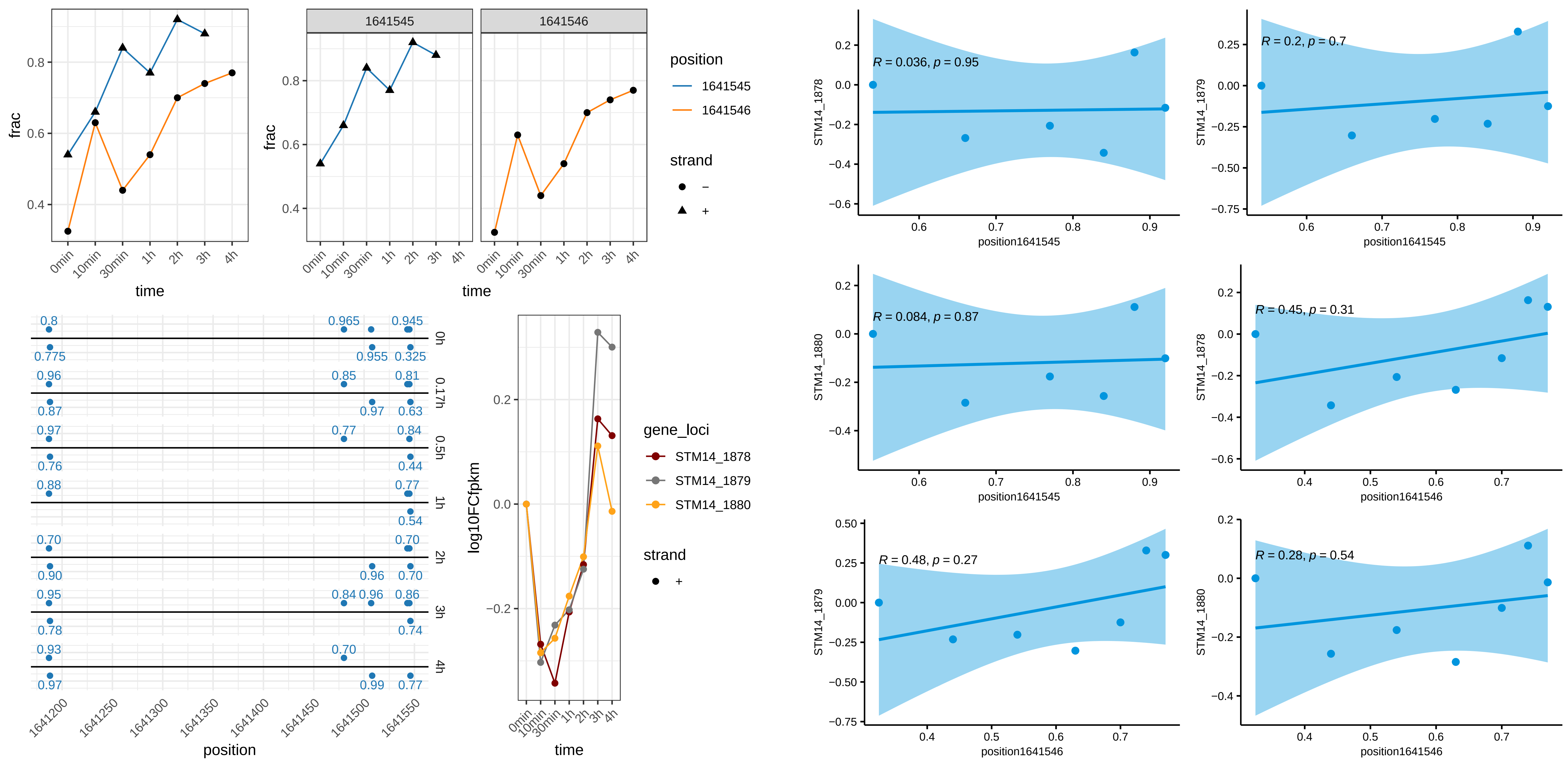

frac
time
position
1641545
1641546
strand
−
+
position
log10FCfpkm
gene_loci
STM14_1878
STM14_1879
STM14_1880
0h
0.17h
0.5h
1h
2h
3h
4h
R = 0.036, p = 0.95
R = 0.2, p = 0.7
R = 0.084, p = 0.87
R = 0.45, p = 0.31
R = 0.48, p = 0.27
R = 0.28, p = 0.54
position1641545
position1641546

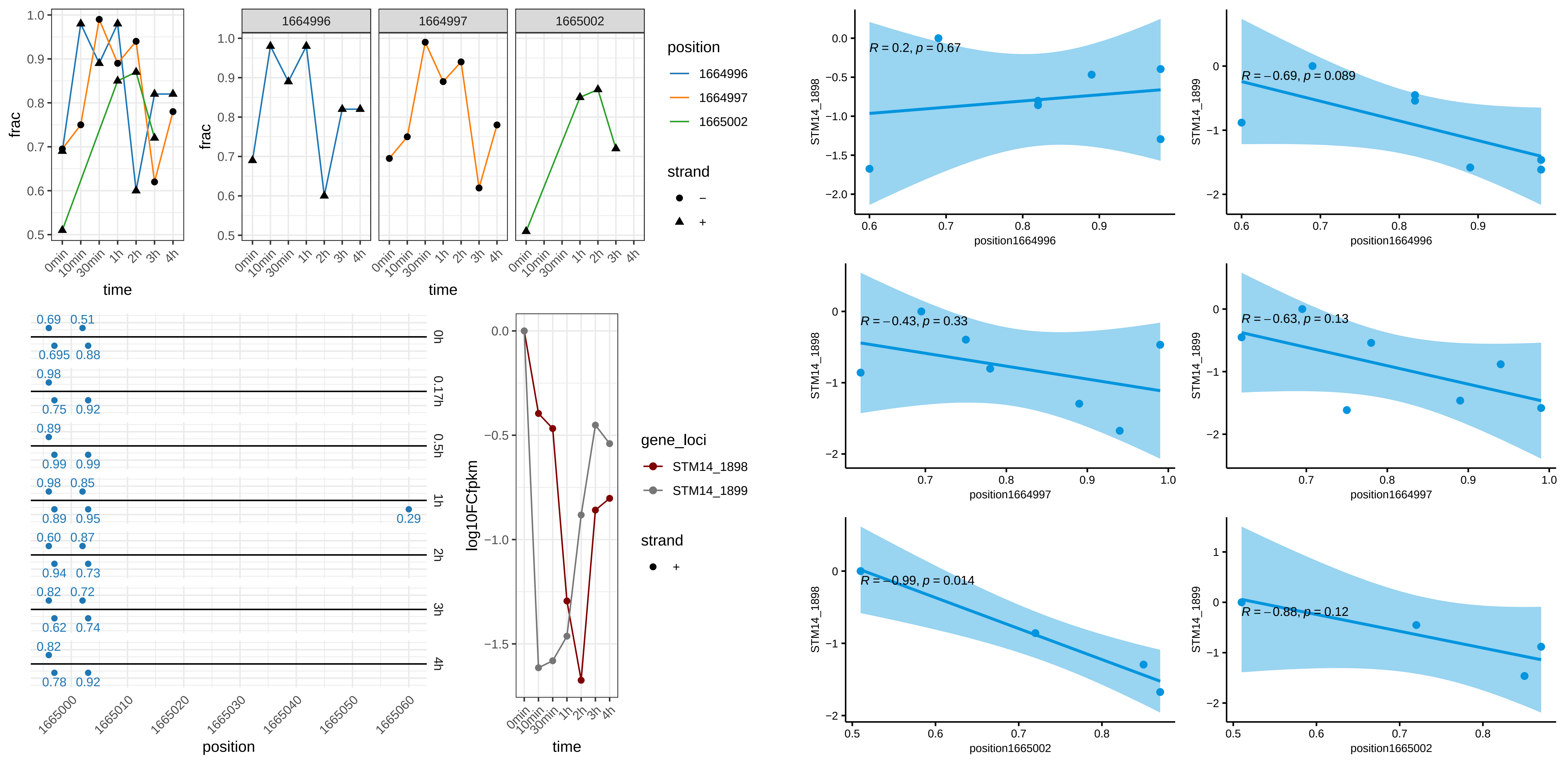

frac
time
1664996
1664997
1665002
position
strand
–
+
0min
10min
30min
1h
2h
3h
4h
0h
0.17h
0.5h
log10FCfpkm
gene_loci
STM14_1898
STM14_1899
R = 0.2, p = 0.67
R = −0.69, p = 0.089
R = −0.43, p = 0.33
R = −0.63, p = 0.13
R = −0.99, p = 0.014
R = −0.88, p = 0.12
position1664996
position1664997
position1665002

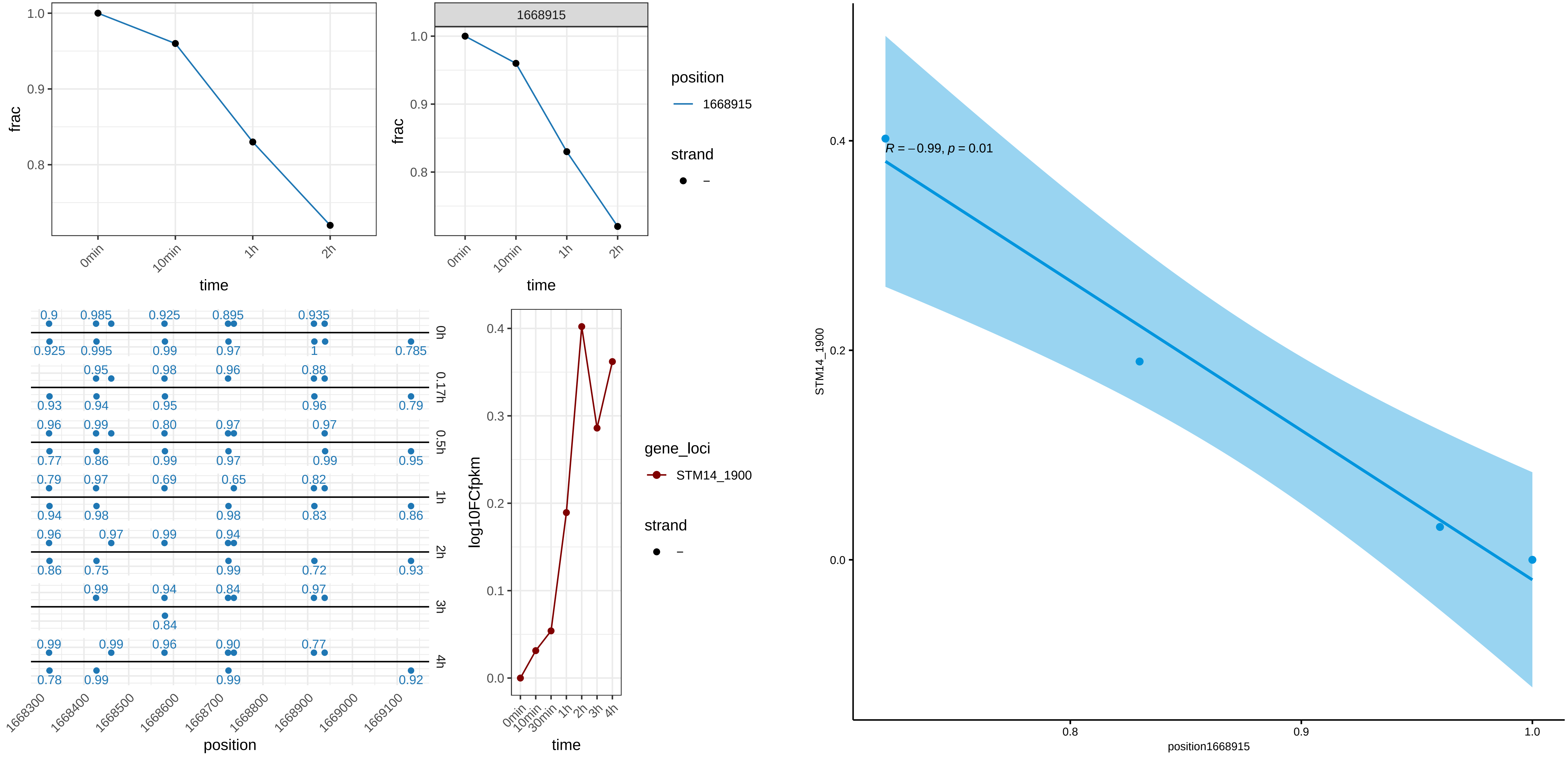
frac
time
0min
10min
1h
2h
1668915
position
strand
–
0h
0.17h
0.5h
1h
2h
3h
4h
position
1668300
1668400
1668500
1668600
1668700
1668800
1668900
1669000
1669100
log10FCfpkm
30min
3h
4h
gene_loci
STM14_1900
R = –0.99, p = 0.01
STM14_1900
position1668915

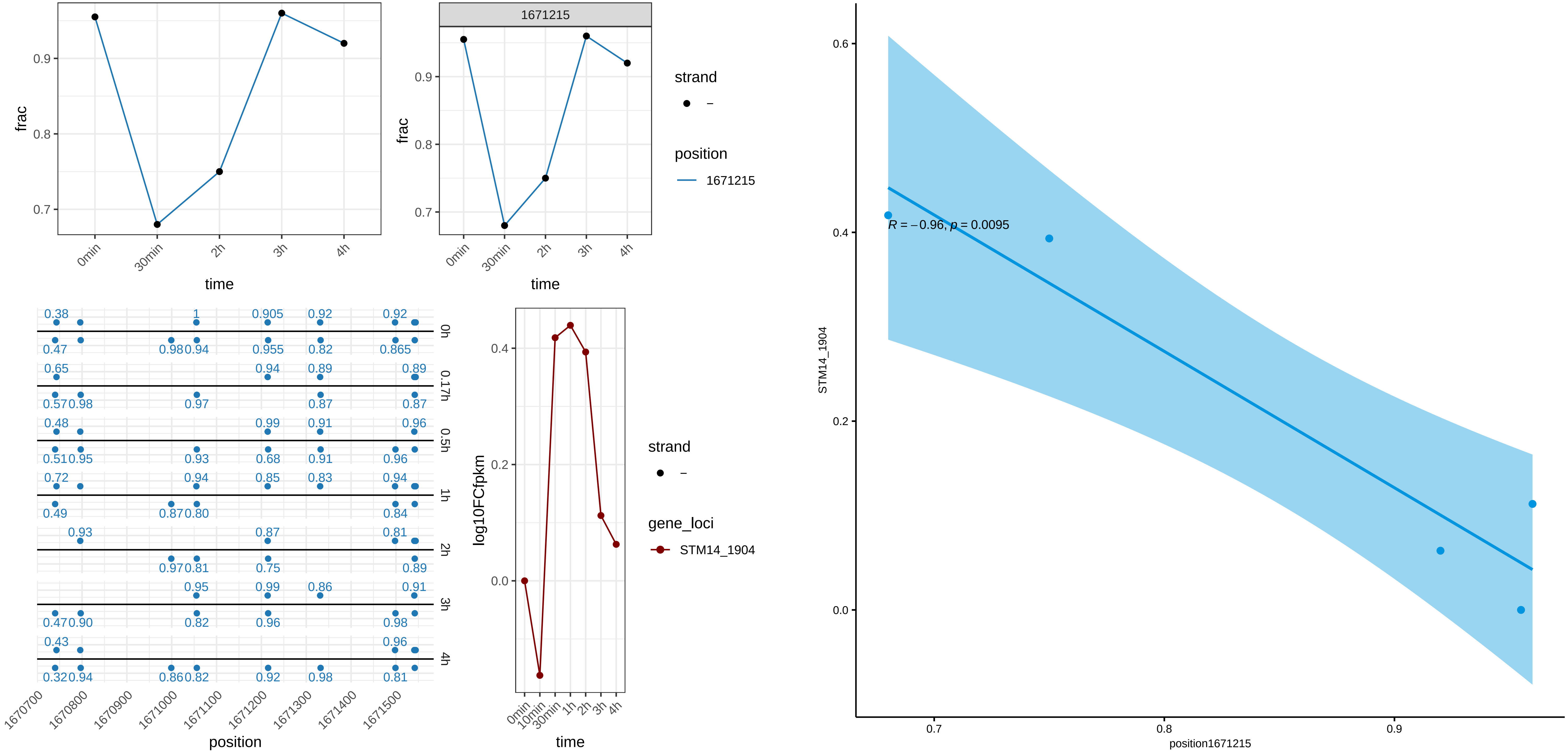

frac
time
0min
30min
2h
3h
4h
1671215
strand
position
1671215
position
0h
0.17h
0.5h
1h
2h
3h
4h
log10FCfpkm
10min
1h
gene_loci
STM14_1904
R = −0.96, p = 0.0095
STM14_1904
position1671215

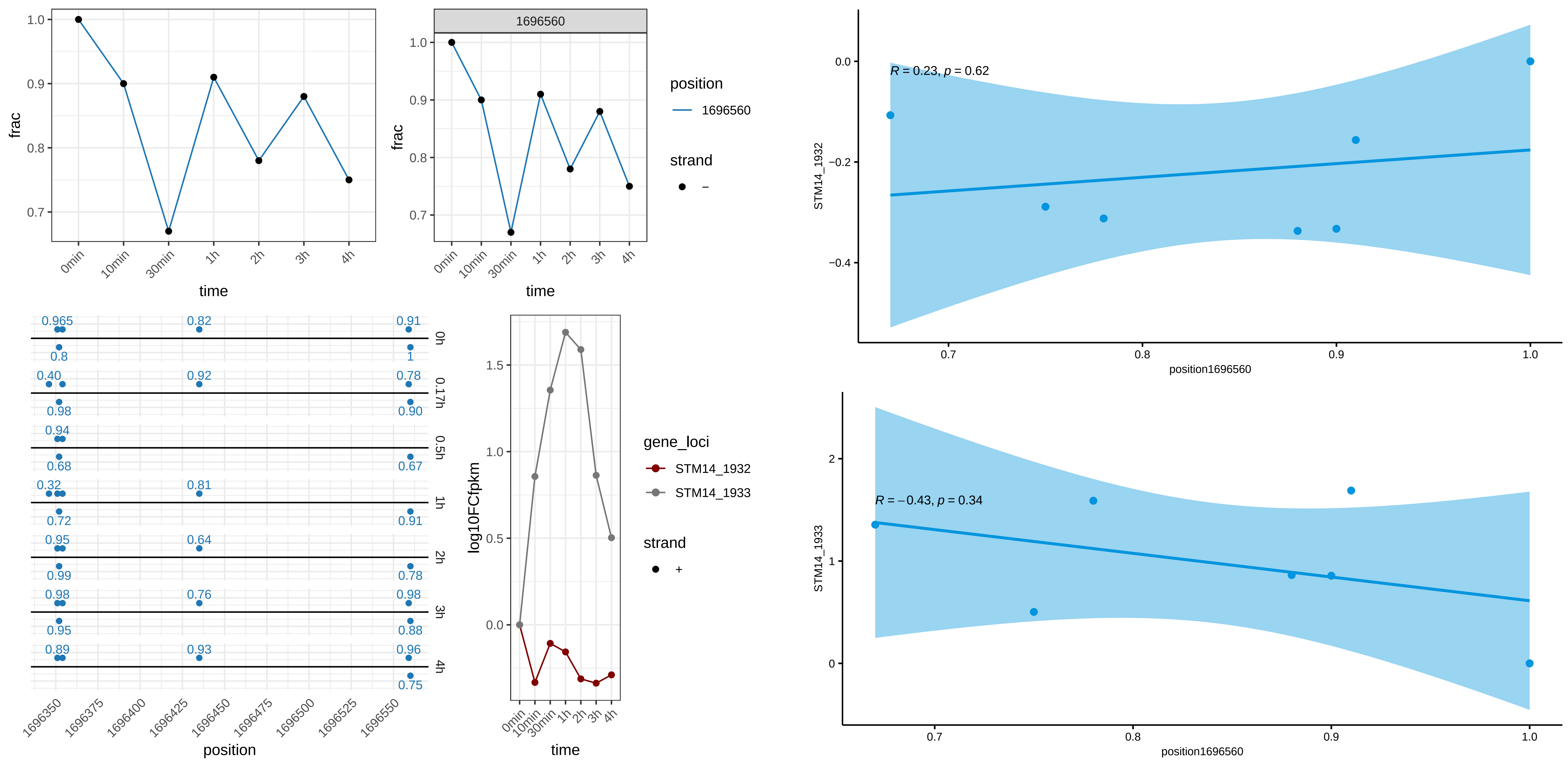

frac
time
1696560
position
strand
R = 0.23, p = 0.62
STM14_1932
position1696560
R = −0.43, p = 0.34
STM14_1933
log10FCfpkm
gene_loci

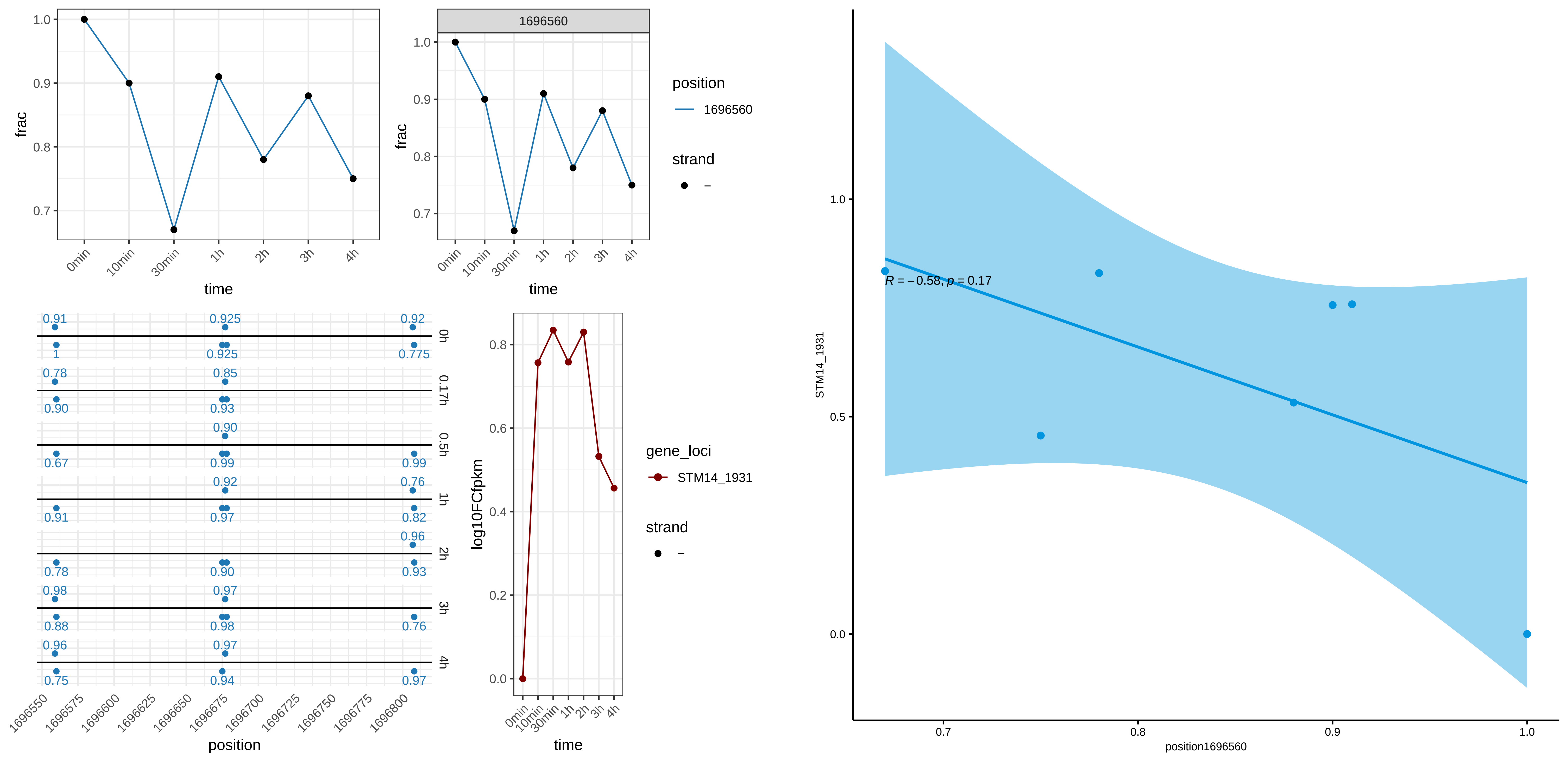

frac
time
0min
10min
30min
1h
2h
3h
4h
1696560
position
strand
position
log10FCfpkm
gene_loci
STM14_1931
0h
0.17h
0.5h
1h
2h
3h
4h
R = −0.58, p = 0.17
STM14_1931
position1696560

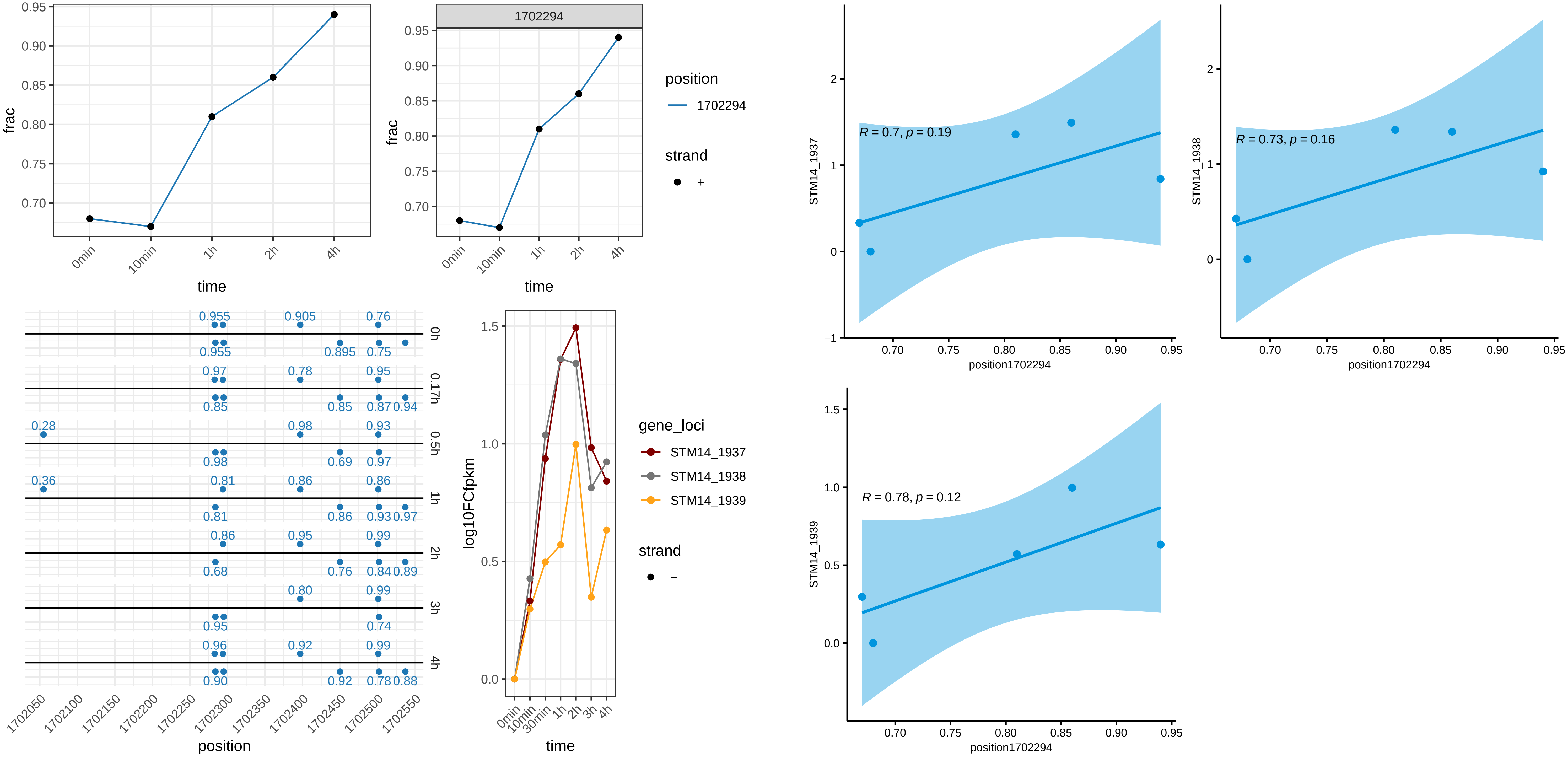

frac
time
0min
10min
1h
2h
4h
1702294
position
strand
+
0.955
0.905
0.76
0.895
0.75
0.97
0.78
0.95
0.85
0.87
0.94
0.28
0.98
0.93
0.69
0.36
0.81
0.86
0.97
0.99
0.68
0.76
0.84
0.89
0.80
0.74
0.96
0.92
0.90
0.88
0h
0.17h
0.5h
3h
1702050
1702100
1702150
1702200
1702250
1702300
1702350
1702400
1702450
1702500
1702550
log10FCfpkm
30min
gene_loci
STM14_1937
STM14_1938
STM14_1939
–
R = 0.7, p = 0.19
R = 0.73, p = 0.16
R = 0.78, p = 0.12
position1702294

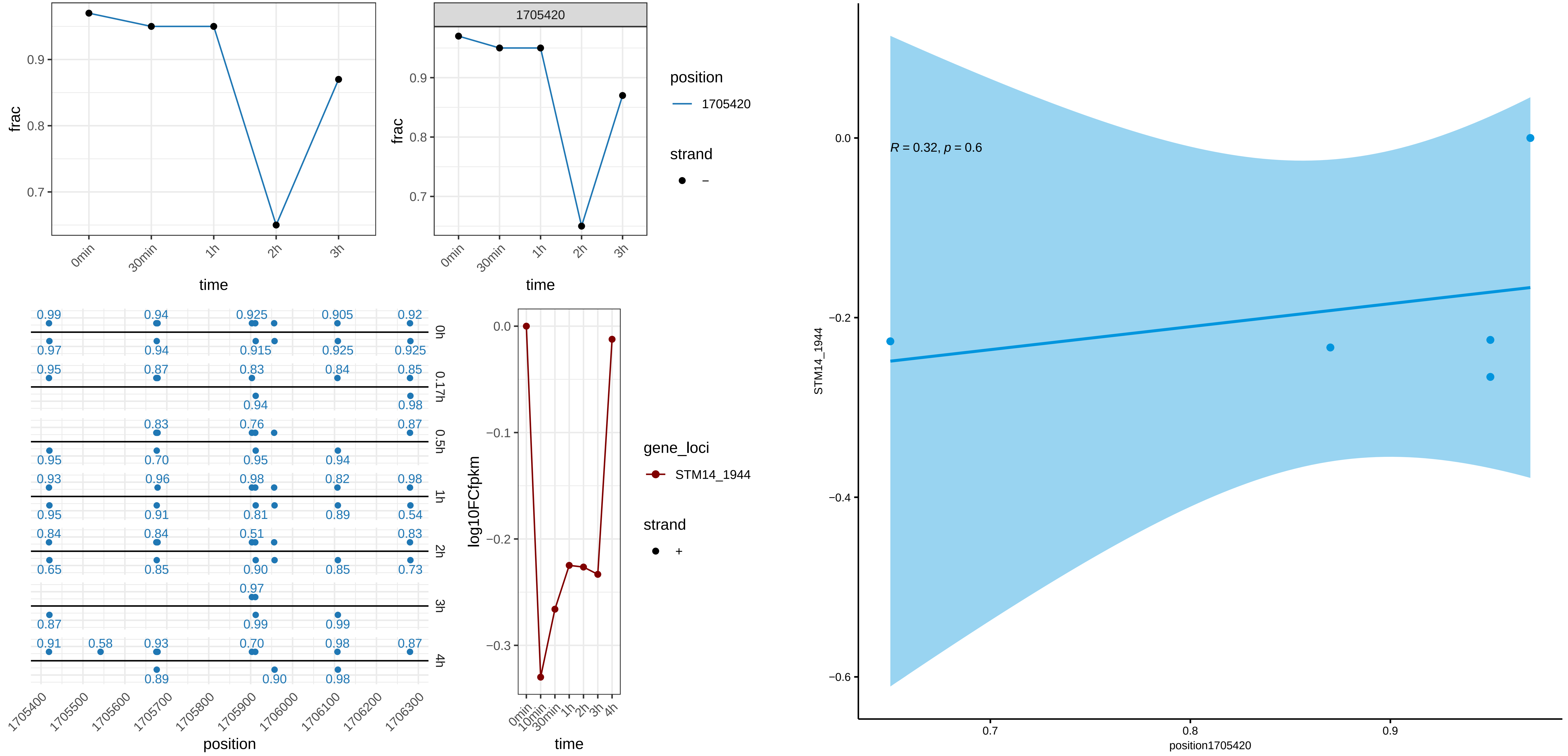

1705420
frac
time
0min
30min
1h
2h
3h
position
1705420
strand
–
log10FCfpkm
10min
4h
gene_loci
STM14_1944
+
0h
0.17h
0.5h
R = 0.32, p = 0.6
STM14_1944
position1705420

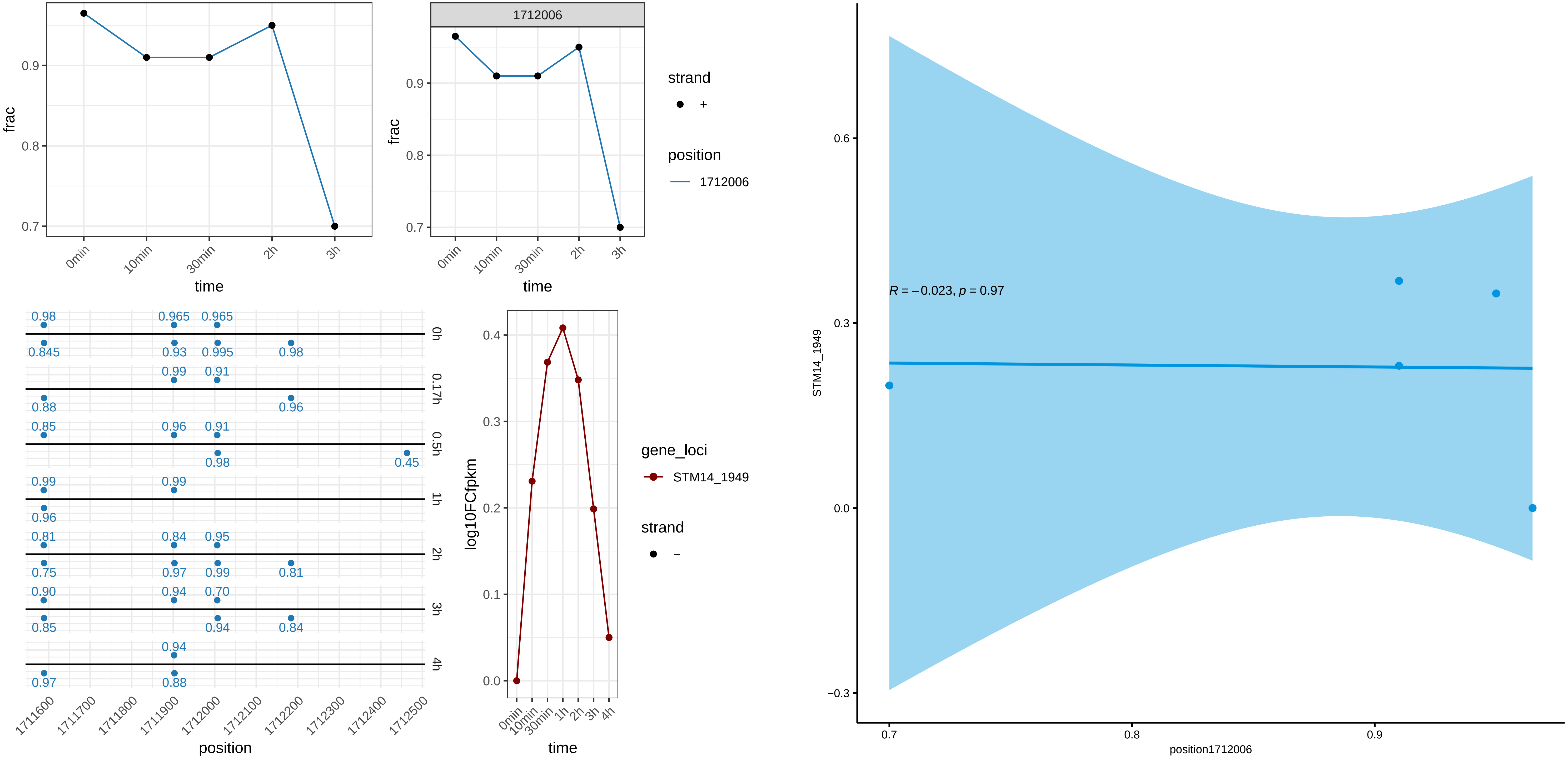
frac
time
1712006
strand
+
position
1712006
log10FCfpkm
gene_loci
STM14_1949
strand
–
position
R = −0.023, p = 0.97
STM14_1949
position1712006

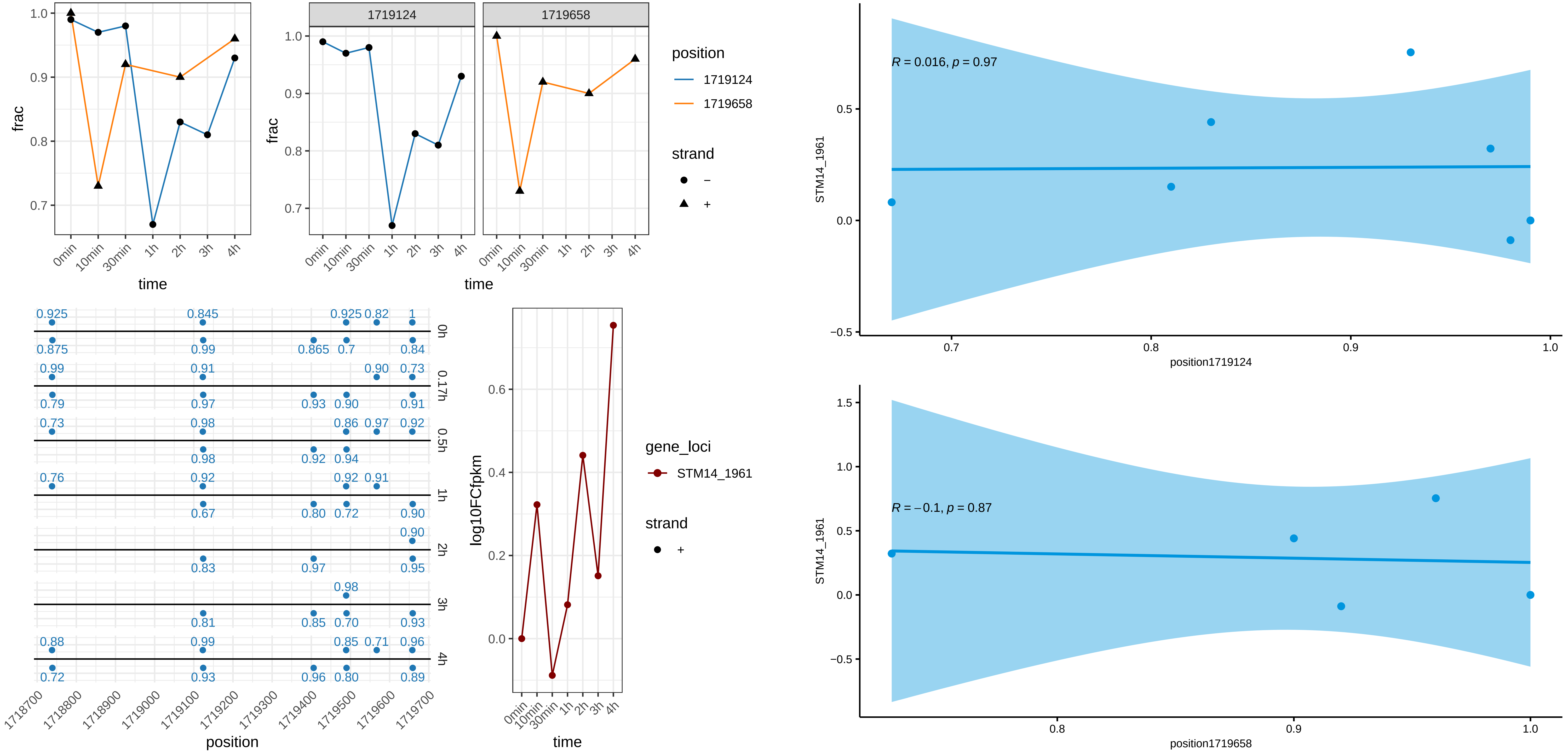

frac
time
0min
10min
30min
1h
2h
3h
4h
1719124
1719658
position
1719124
1719658
strand
–
+
R = 0.016, p = 0.97
STM14_1961
position1719124
R = –0.1, p = 0.87
STM14_1961
position1719658
0h
0.17h
0.5h
1h
2h
3h
4h
position
log10FCfpkm
gene_loci
STM14_1961
strand
+

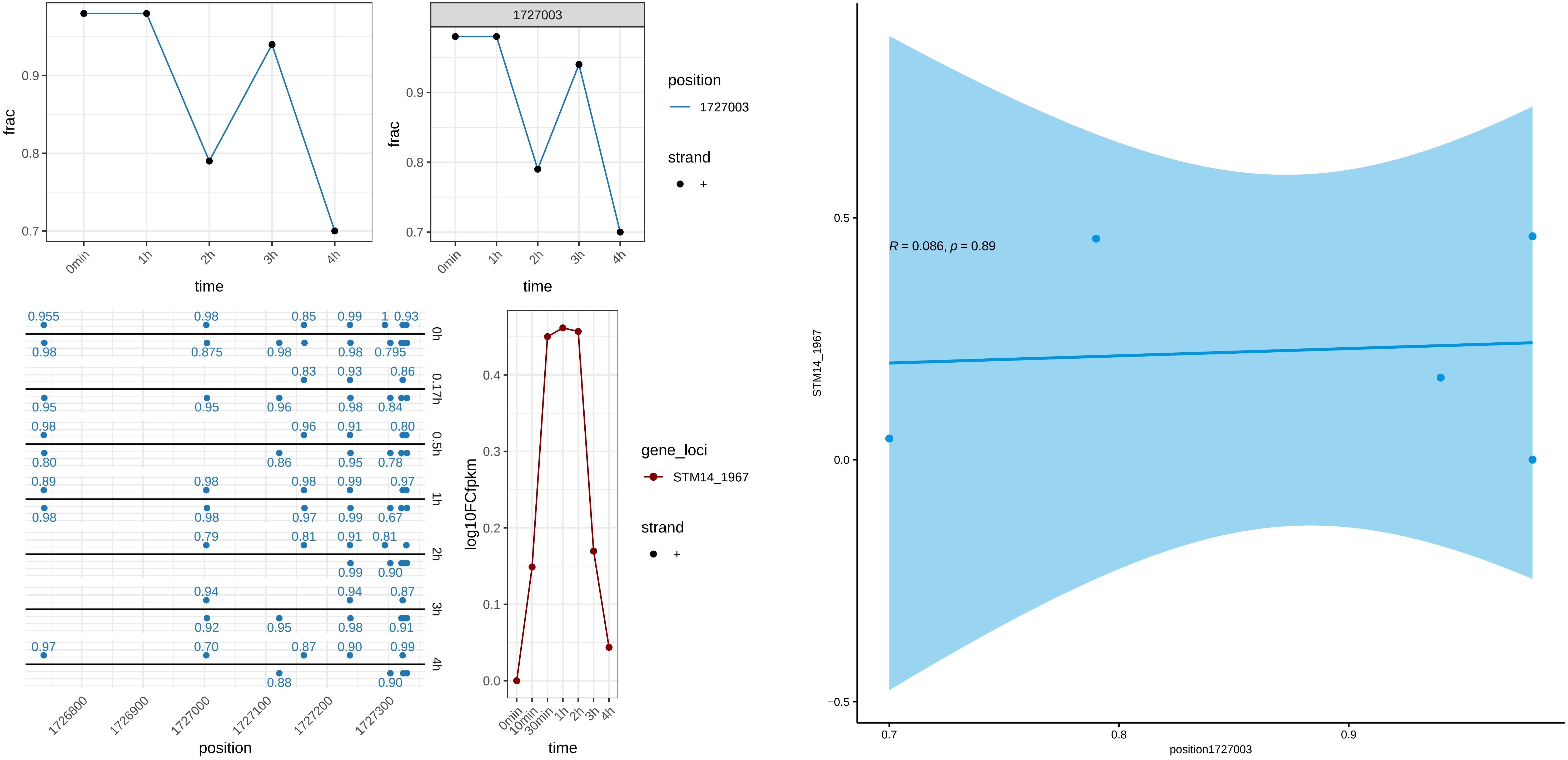

frac
time
0min
1h
2h
3h
4h
1727003
position
strand
+
position
1726800
1726900
1727000
1727100
1727200
1727300
0h
0.17h
0.5h
1h
2h
3h
4h
log10FCfpkm
10min
30min
gene_loci
STM14_1967
R = 0.086, p = 0.89
position1727003

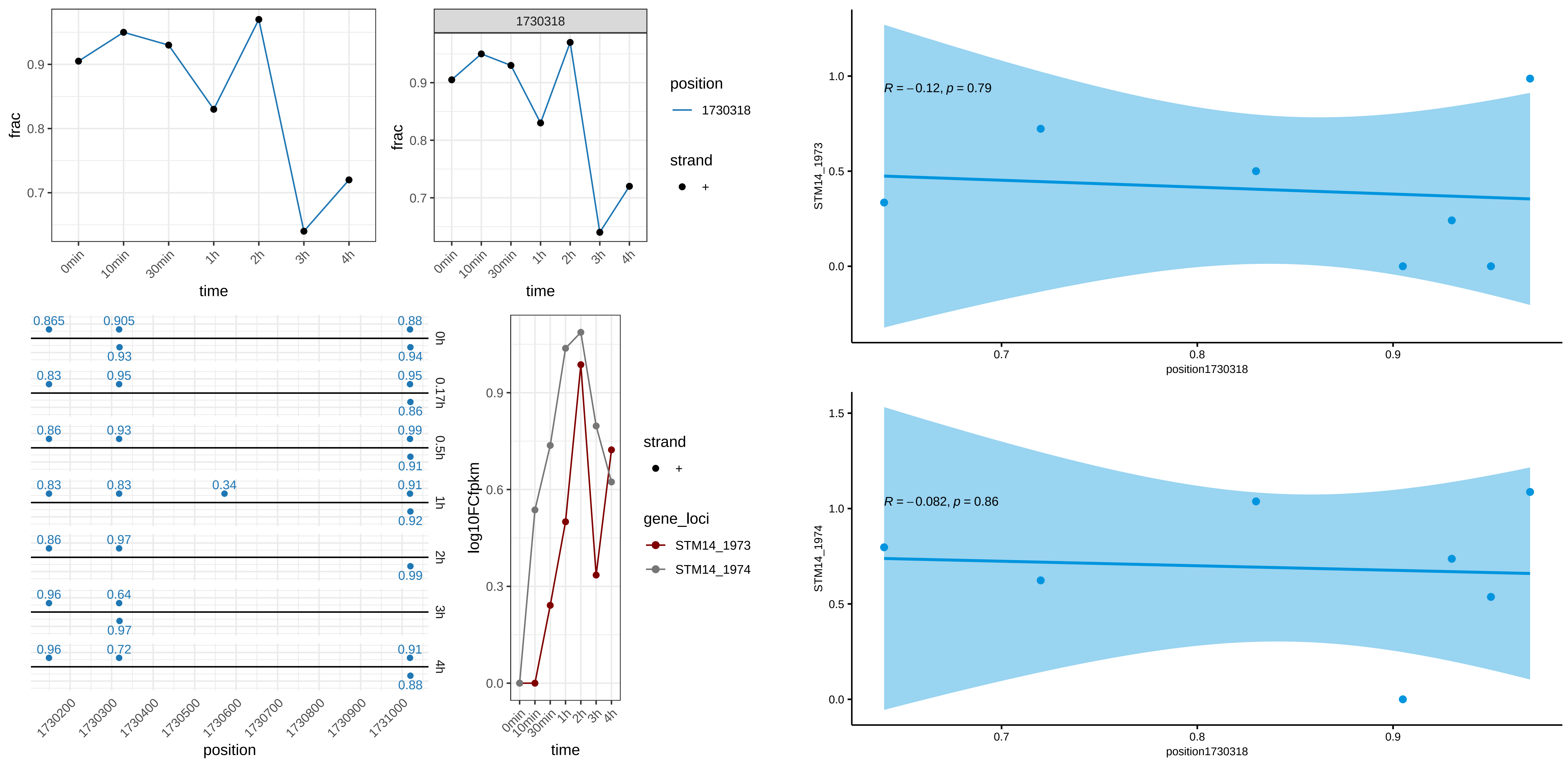

frac
time
1730318
position
strand
+
log10FCfpkm
gene_loci
STM14_1973
STM14_1974
R = −0.12, p = 0.79
R = −0.082, p = 0.86
position1730318

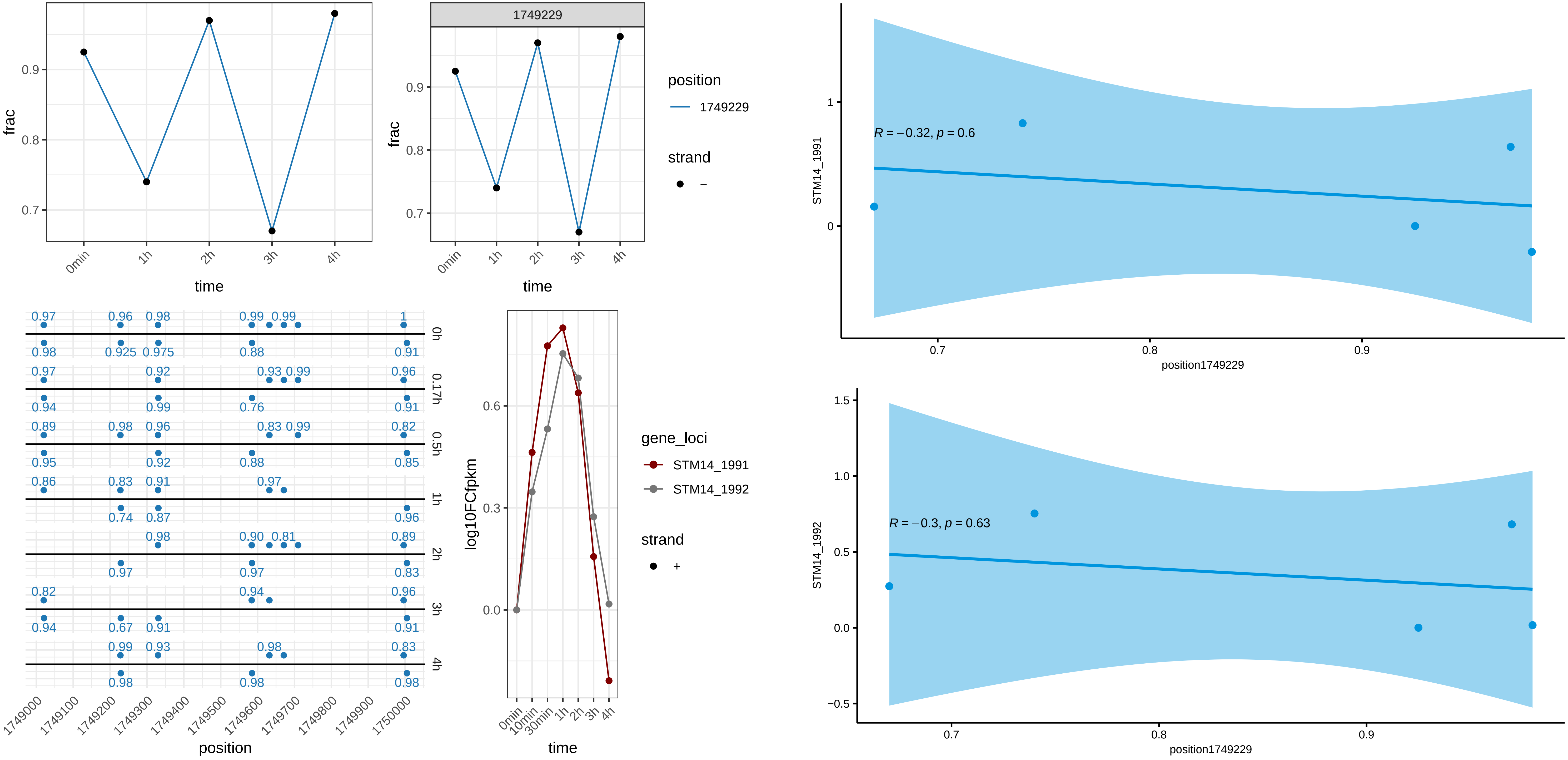

frac
time
0min
1h
2h
3h
4h
1749229
position
1749229
strand
–
0h
0.17h
0.5h
1h
2h
3h
4h
position
log10FCfpkm
0min
10min
30min
gene_loci
STM14_1991
STM14_1992
strand
+
R = −0.32, p = 0.6
STM14_1991
position1749229
R = −0.3, p = 0.63
STM14_1992
position1749229

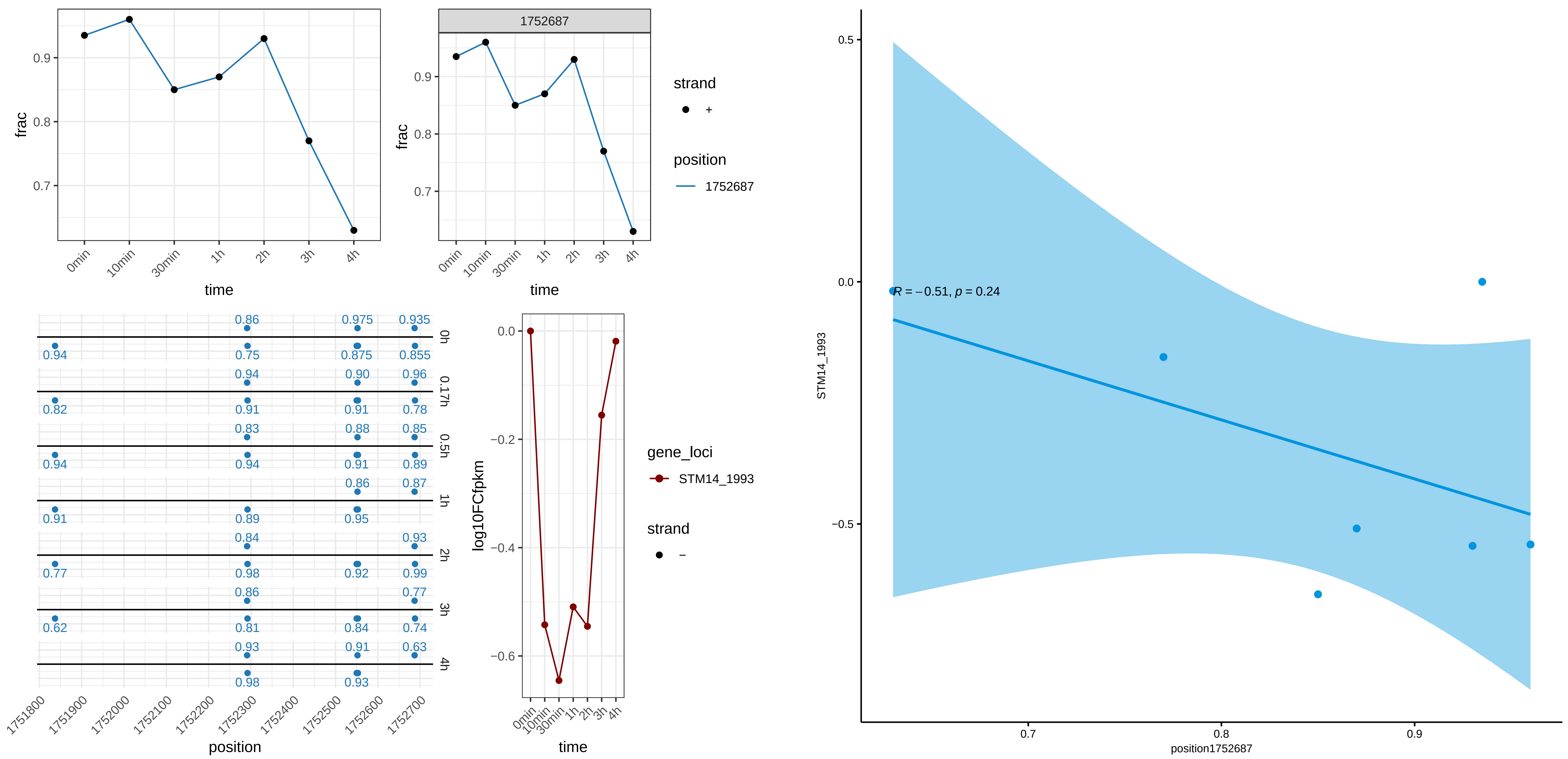

frac
time
1752687
strand
+
position
1752687
0.86
0.975
0.935
0.94
0.75
0.875
0.855
0.94
0.90
0.96
0.82
0.91
0.91
0.78
0.83
0.88
0.85
0.94
0.94
0.91
0.89
0.86
0.87
0.91
0.89
0.95
0.84
0.93
0.77
0.98
0.92
0.99
0.86
0.77
0.62
0.81
0.84
0.74
0.93
0.91
0.63
0.98
0.93
0h
0.17h
0.5h
1h
2h
3h
4h
position
log10FCfpkm
time
gene_loci
STM14_1993
strand
–
R = −0.51, p = 0.24
STM14_1993
position1752687

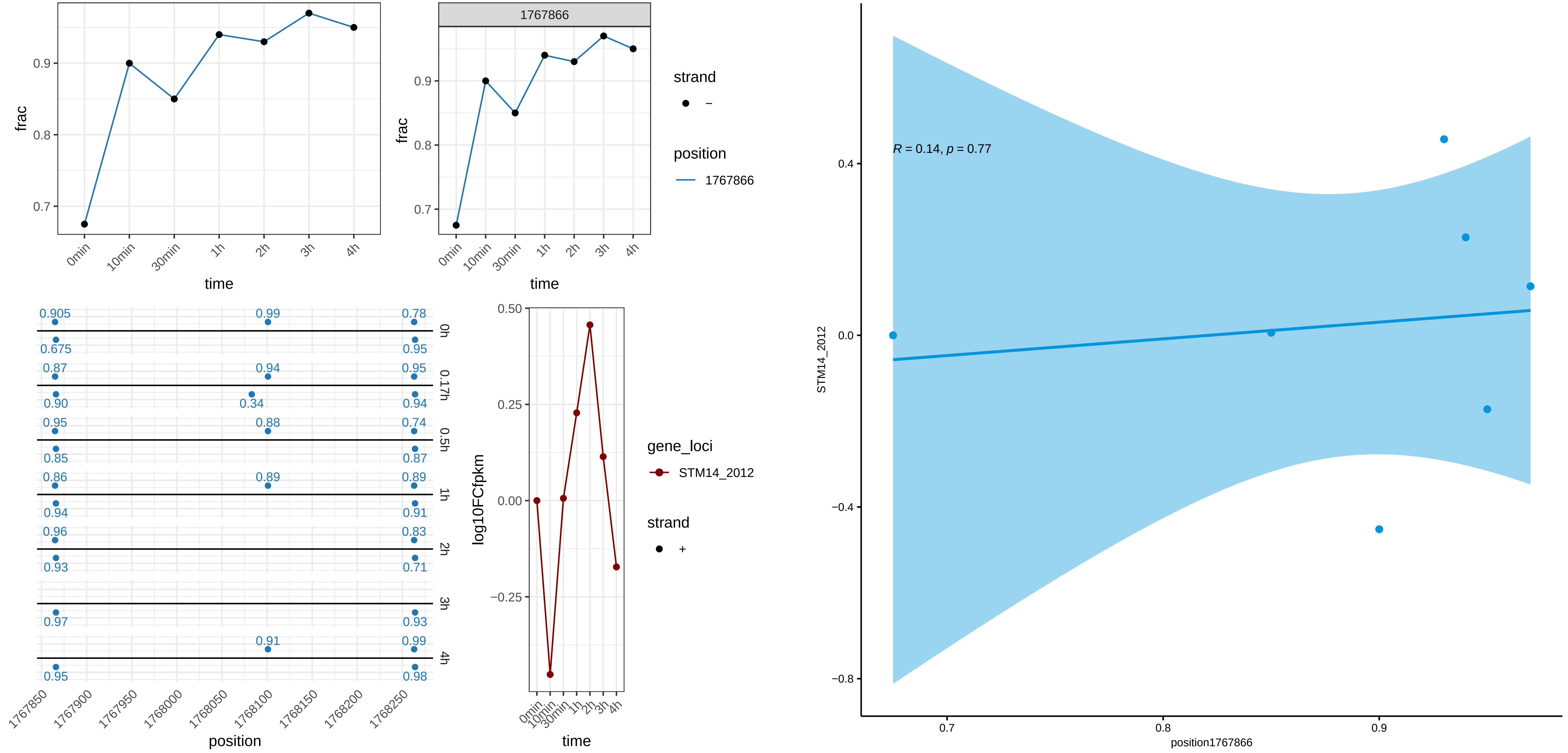

frac
0.7
0.8
0.9
0min
10min
30min
1h
2h
3h
4h
time
1767866
strand
–
position
1767866
0.905
0.99
0.78
0.675
0.95
0.87
0.94
0.95
0.90
0.34
0.94
0.95
0.88
0.74
0.85
0.87
0.86
0.89
0.89
0.94
0.91
0.96
0.83
0.93
0.71
0.97
0.93
0.91
0.99
0.95
0.98
0h
0.17h
0.5h
1h
2h
3h
4h
1767850
1767900
1767950
1768000
1768050
1768100
1768150
1768200
1768250
position
log10FCfpkm
0.50
0.25
0.00
−0.25
gene_loci
STM14_2012
strand
+
R = 0.14, p = 0.77
STM14_2012
0.4
0.0
−0.4
−0.8
0.7
0.8
0.9
position1767866

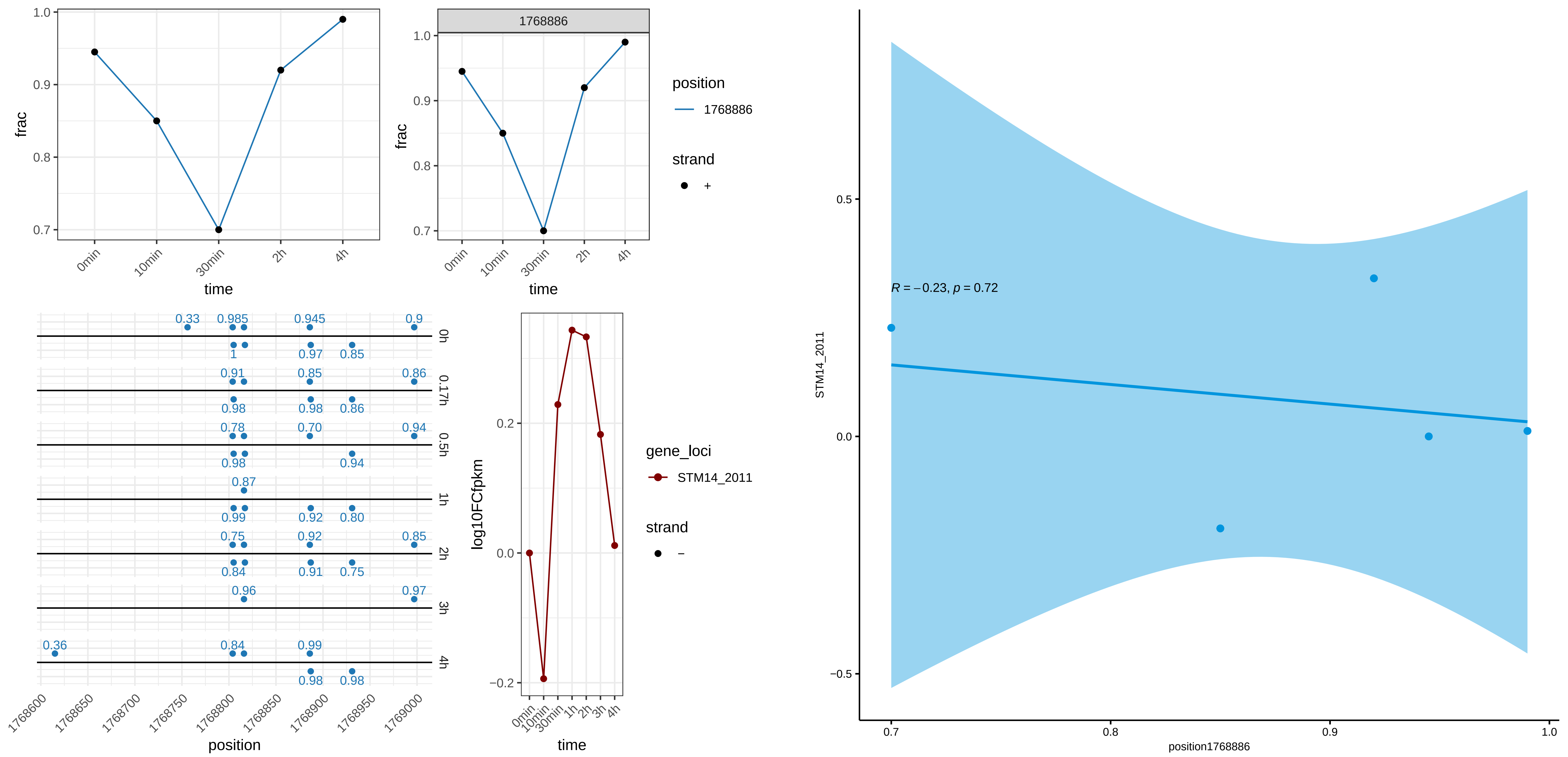

frac
1.0
0.9
0.8
0.7
0min
10min
30min
2h
4h
time
1768886
position
strand
+
0.33
0.985
0.945
0.9
1
0.97
0.85
0h
0.91
0.85
0.86
0.98
0.98
0.86
0.17h
0.78
0.70
0.94
0.98
0.94
0.5h
0.87
0.99
0.92
0.80
1h
0.75
0.92
0.85
0.84
0.91
0.75
2h
0.96
0.97
3h
0.36
0.84
0.99
0.98
0.98
4h
1768600
1768650
1768700
1768750
1768800
1768850
1768900
1768950
1769000
log10FCfpkm
0.2
0.0
−0.2
1h
3h
gene_loci
STM14_2011
–
STM14_2011
R = −0.23, p = 0.72
0.5
0.0
−0.5
0.7
0.8
0.9
1.0
position1768886

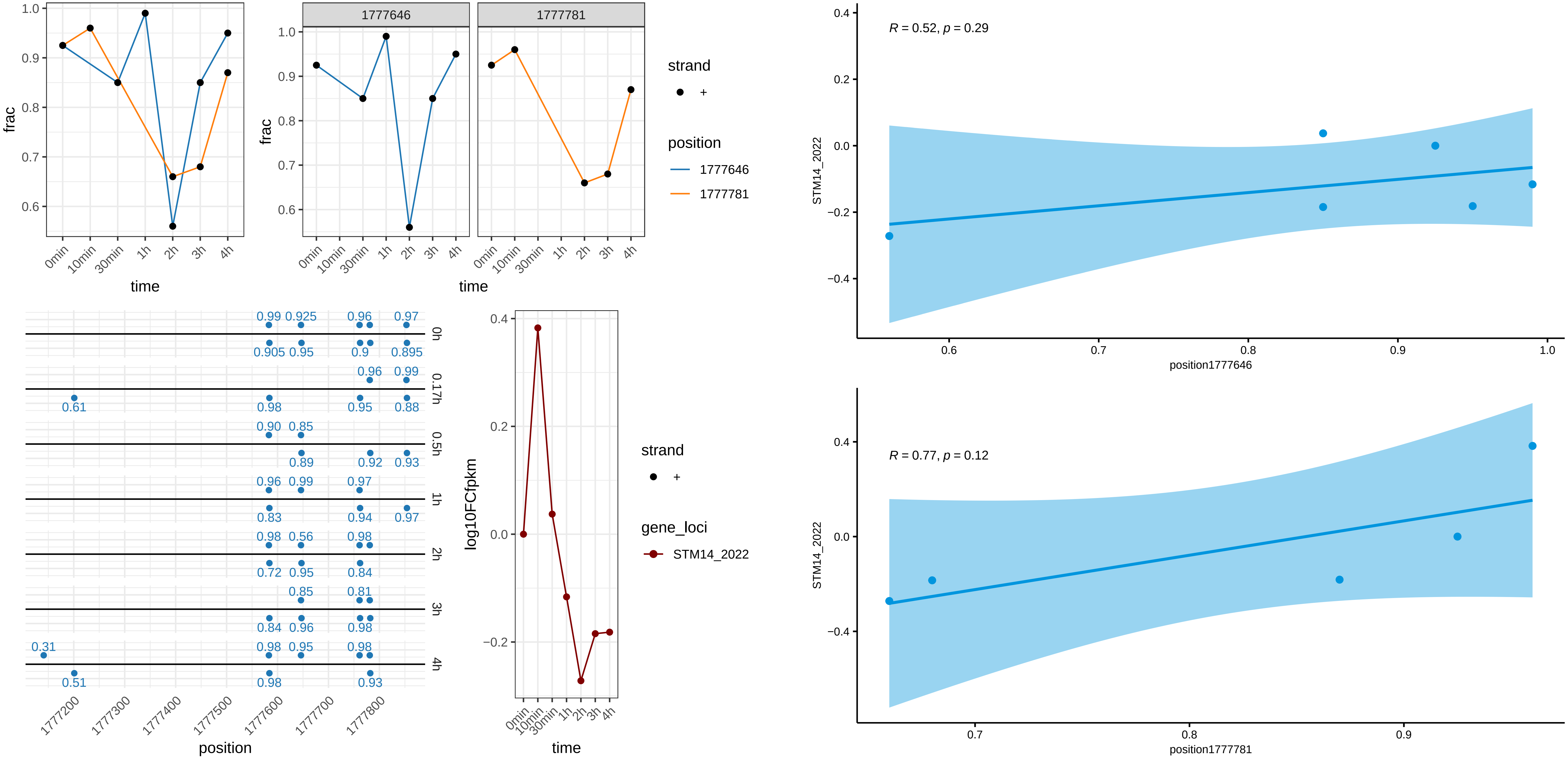
frac
time
0min
10min
30min
1h
2h
3h
4h
1777646
1777781
strand
+
position
1777646
1777781
R = 0.52, p = 0.29
STM14_2022
position1777646
R = 0.77, p = 0.12
position1777781
0.99 0.925 0.96 0.97
0.905 0.95 0.9 0.895
0h
0.96 0.99
0.61 0.98 0.95 0.88
0.17h
0.90 0.85
0.89 0.92 0.93
0.5h
0.96 0.99 0.97
0.83 0.94 0.97
1h
0.98 0.56 0.98
0.72 0.95 0.84
2h
0.85 0.81
0.84 0.96 0.98
3h
0.31 0.98 0.95 0.98
0.51 0.98 0.93
4h
1777200
1777300
1777400
1777500
1777600
1777700
1777800
position
log10FCfpkm
gene_loci
STM14_2022

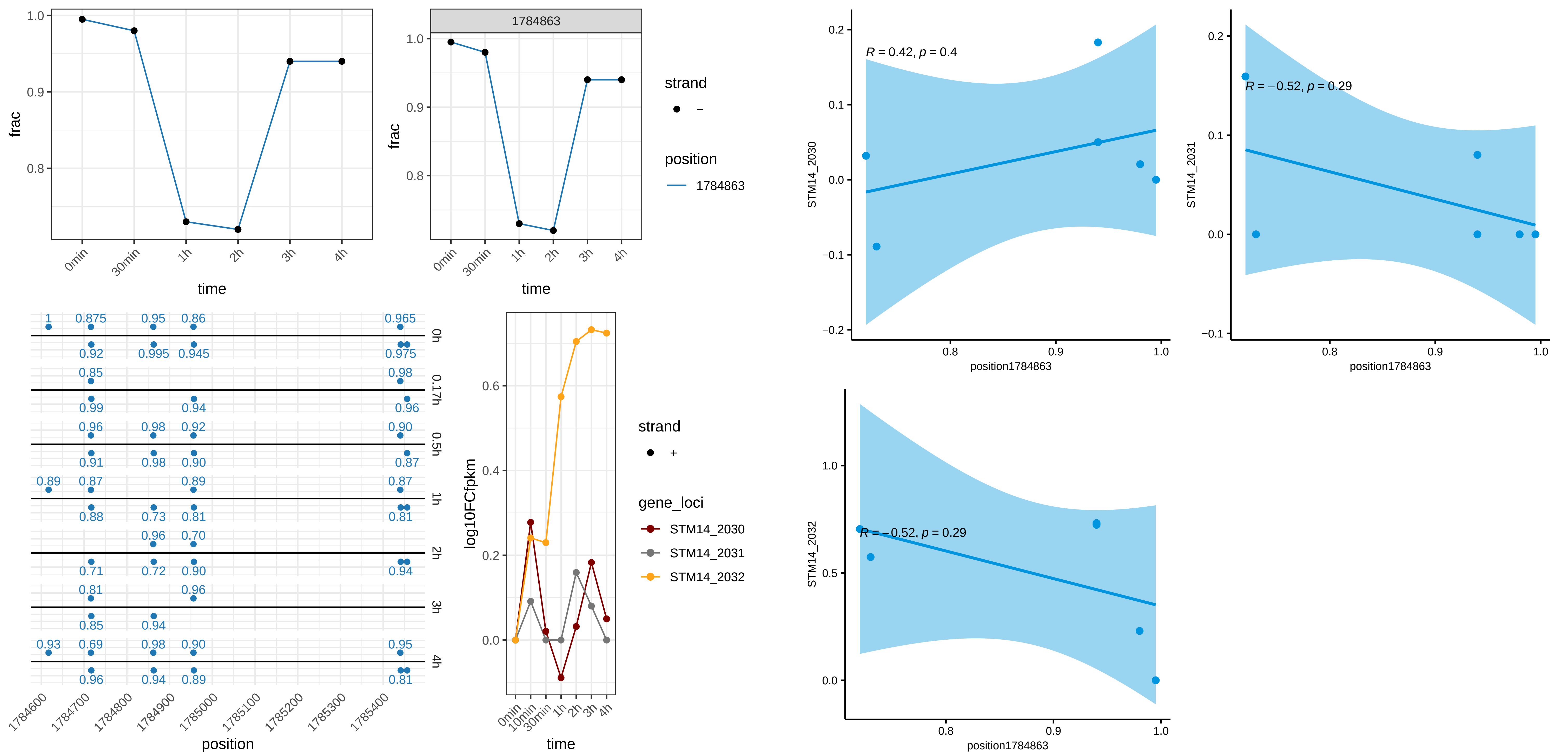

frac
time
1784863
strand
position
log10FCfpkm
gene_loci
STM14_2030
STM14_2031
STM14_2032
R = 0.42, p = 0.4
R = −0.52, p = 0.29
R = −0.52, p = 0.29
position1784863

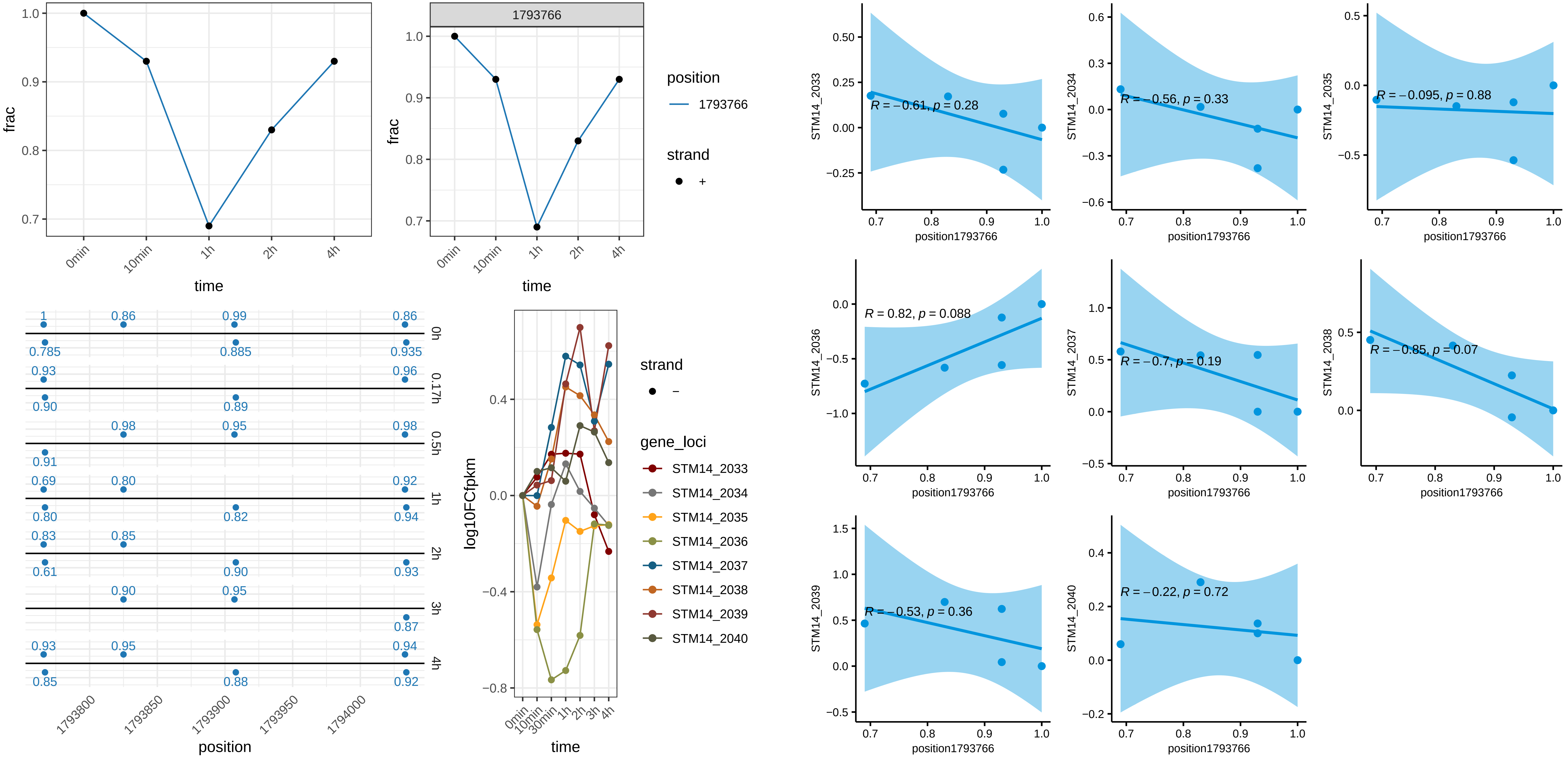

frac
time
0min
10min
1h
2h
4h
1793766
position
1793766
strand
+
log10FCfpkm
30min
3h
strand
–
gene_loci
STM14_2033
STM14_2034
STM14_2035
STM14_2036
STM14_2037
STM14_2038
STM14_2039
STM14_2040
position
1793800
1793850
1793900
1793950
1794000
0h
0.17h
0.5h
1h
2h
3h
4h
1
0.86
0.99
0.86
0.785
0.885
0.935
0.93
0.96
0.90
0.89
0.98
0.95
0.98
0.91
0.69
0.80
0.92
0.80
0.82
0.94
0.83
0.85
0.61
0.90
0.93
0.90
0.95
0.87
0.93
0.95
0.94
0.85
0.88
0.92
position1793766
STM14_2033
R = −0.61, p = 0.28
STM14_2034
R = −0.56, p = 0.33
STM14_2035
R = −0.095, p = 0.88
STM14_2036
R = 0.82, p = 0.088
STM14_2037
R = −0.7, p = 0.19
STM14_2038
R = −0.85, p = 0.07
STM14_2039
R = −0.53, p = 0.36
STM14_2040
R = −0.22, p = 0.72

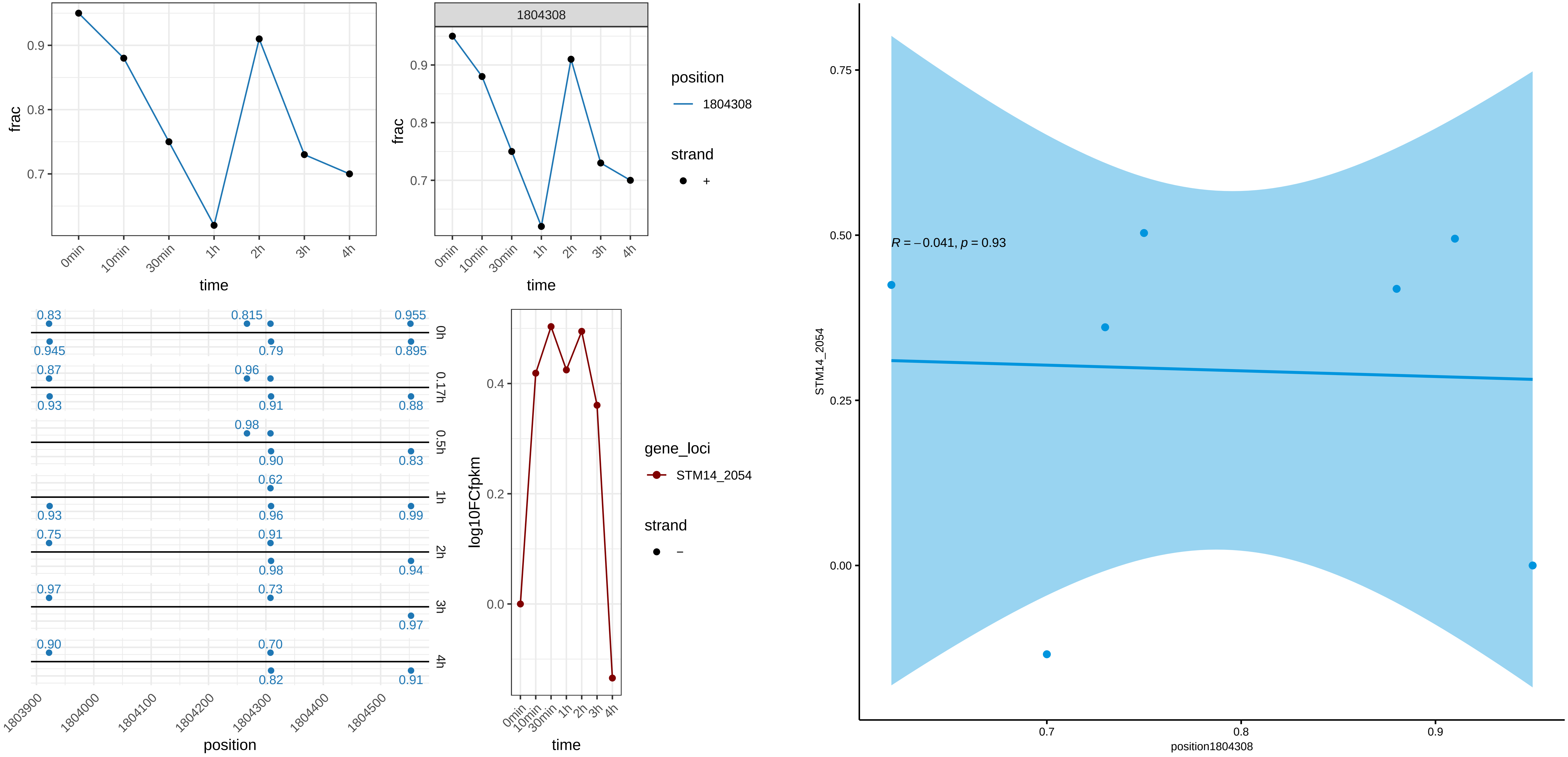

frac
time
0min
10min
30min
1h
2h
3h
4h
1804308
position
strand
+
log10FCfpkm
gene_loci
STM14_2054
−
0h
0.17h
0.5h
1h
2h
3h
4h
1803900
1804000
1804100
1804200
1804300
1804400
1804500
R = −0.041, p = 0.93
STM14_2054
position1804308

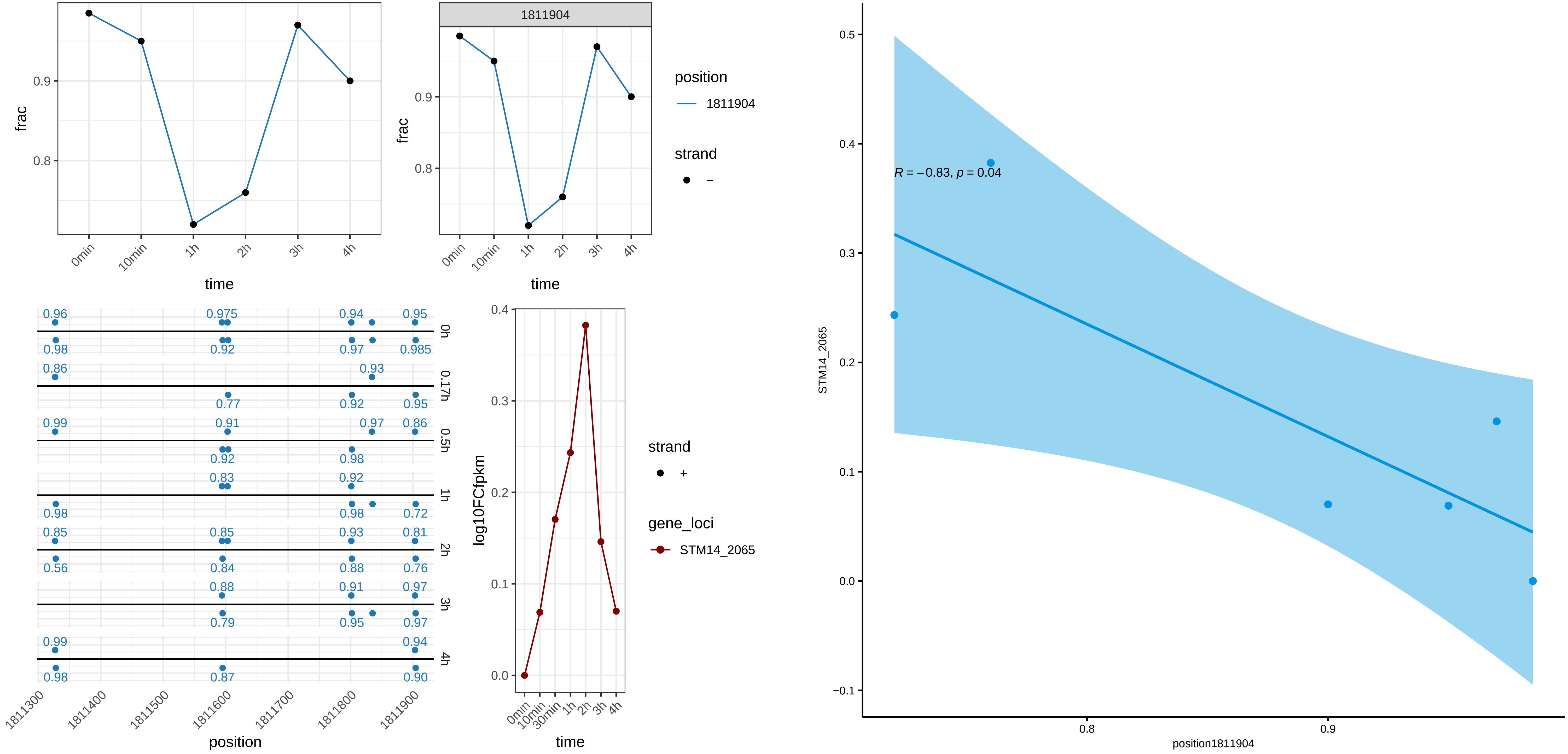
frac
0.9
0.8
0min
10min
1h
2h
3h
4h
time
1811904
frac
0.9
0.8
0min
10min
1h
2h
3h
4h
time
position
1811904
strand
–
0.96
0.975
0.94
0.95
0.98
0.92
0.97
0.985
0h
0.86
0.93
0.77
0.92
0.95
0.17h
0.99
0.91
0.97
0.86
0.92
0.98
0.5h
0.83
0.92
0.98
0.98
0.72
1h
0.85
0.85
0.93
0.81
0.56
0.84
0.88
0.76
2h
0.88
0.91
0.97
0.79
0.95
0.97
3h
0.99
0.94
0.98
0.87
0.90
4h
1811300
1811400
1811500
1811600
1811700
1811800
1811900
position
log10FCfpkm
0.4
0.3
0.2
0.1
0.0
0min
10min
30min
1h
2h
3h
4h
time
strand
+
gene_loci
STM14_2065
R = −0.83, p = 0.04
STM14_2065
0.5
0.4
0.3
0.2
0.1
0.0
−0.1
0.8
0.9
position1811904

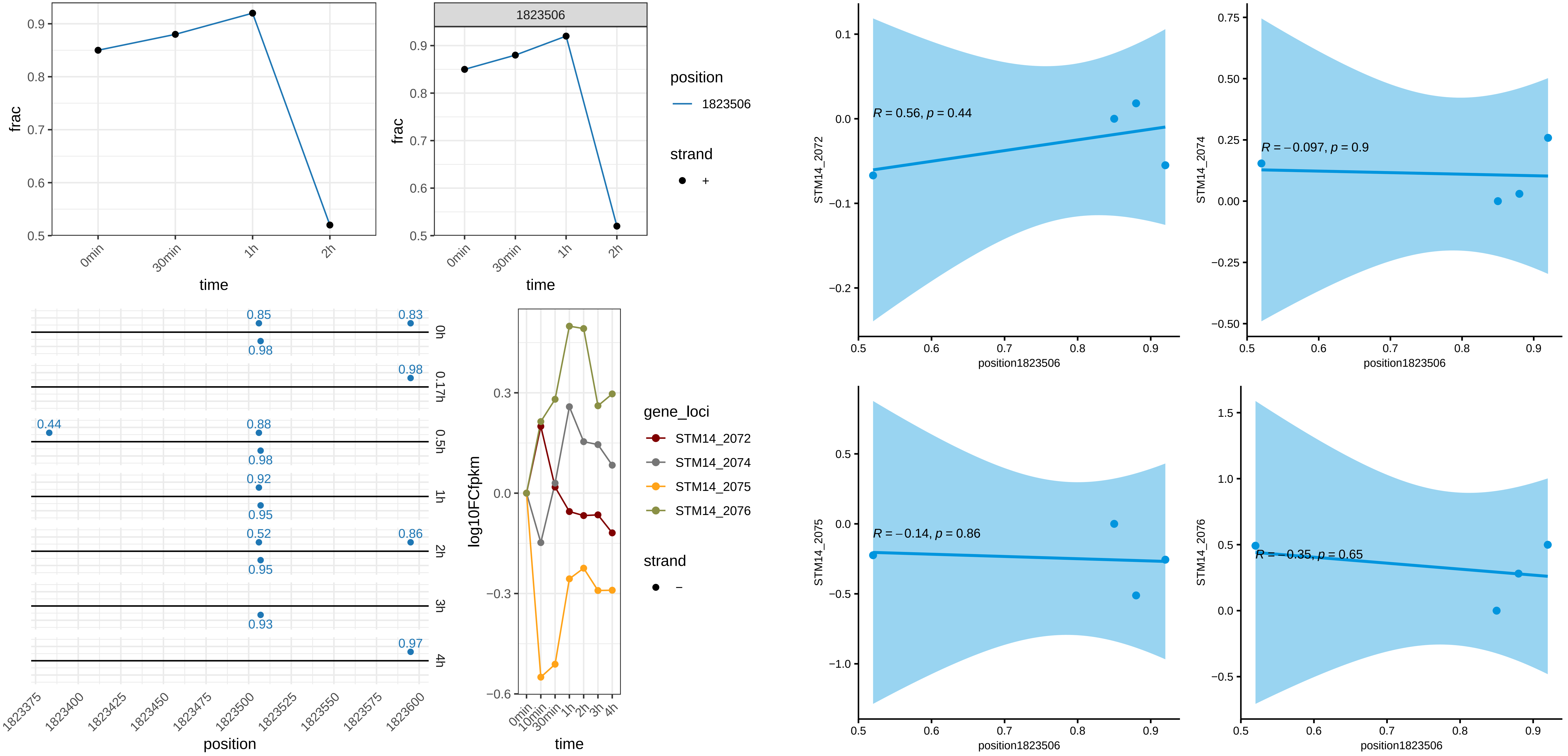

frac
time
0min
30min
1h
2h
1823506
position
1823506
strand
+
0.85
0.83
0.98
0.98
0.44
0.88
0.98
0.92
0.95
0.52
0.86
0.95
0.93
0.97
0h
0.17h
0.5h
1h
2h
3h
4h
position
log10FCfpkm
0min
10min
30min
1h
2h
3h
4h
time
gene_loci
STM14_2072
STM14_2074
STM14_2075
STM14_2076
strand
–
STM14_2072
position1823506
R = 0.56, p = 0.44
STM14_2074
position1823506
R = –0.097, p = 0.9
STM14_2075
position1823506
R = –0.14, p = 0.86
STM14_2076
position1823506
R = –0.35, p = 0.65

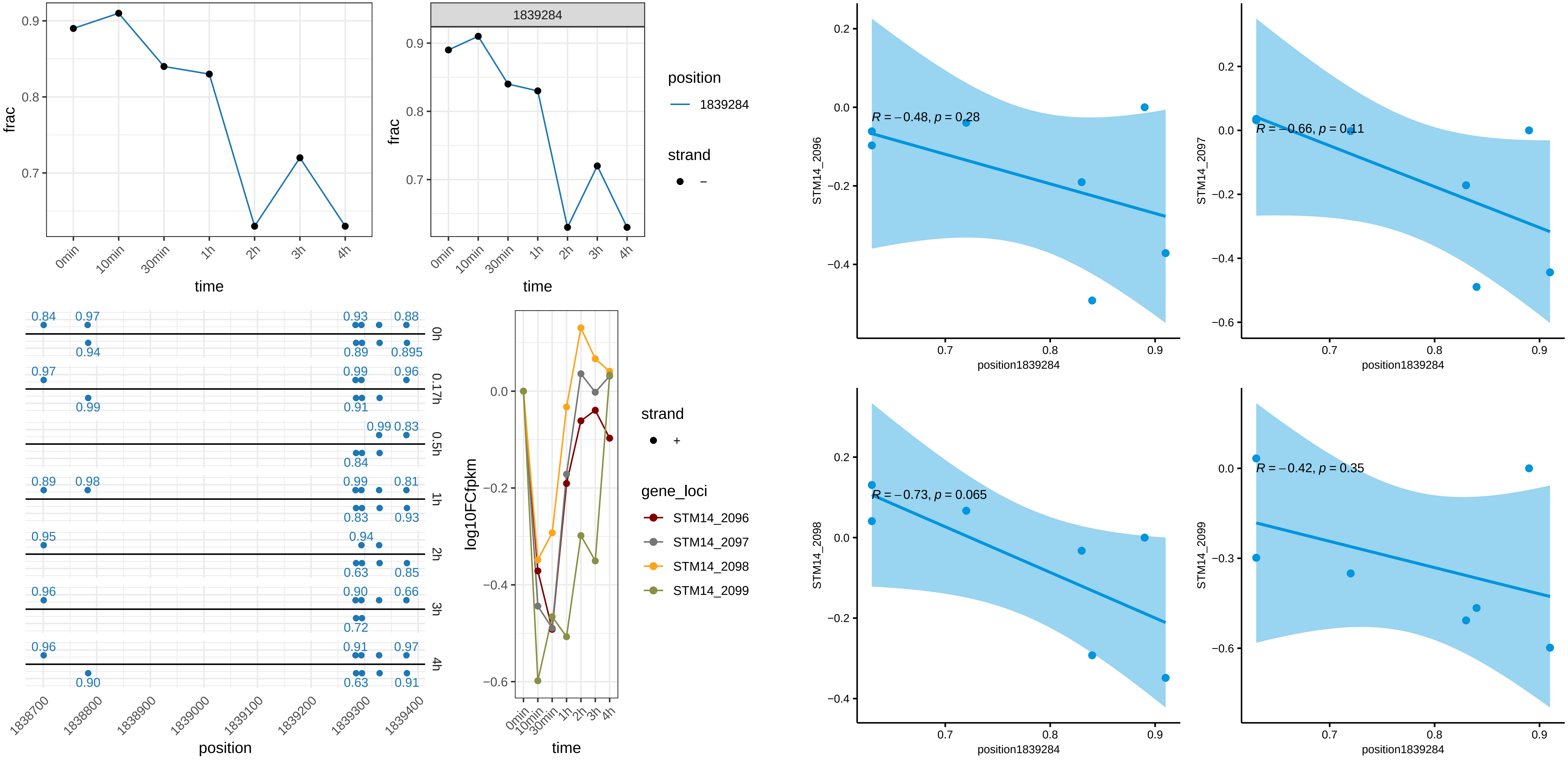

frac
time
0min
10min
30min
1h
2h
3h
4h
1839284
position
strand
–
+
position
log10FCfpkm
gene_loci
STM14_2096
STM14_2097
STM14_2098
STM14_2099
0h
0.17h
0.5h
1h
2h
3h
4h
R = −0.48, p = 0.28
R = −0.66, p = 0.11
R = −0.73, p = 0.065
R = −0.42, p = 0.35
position1839284

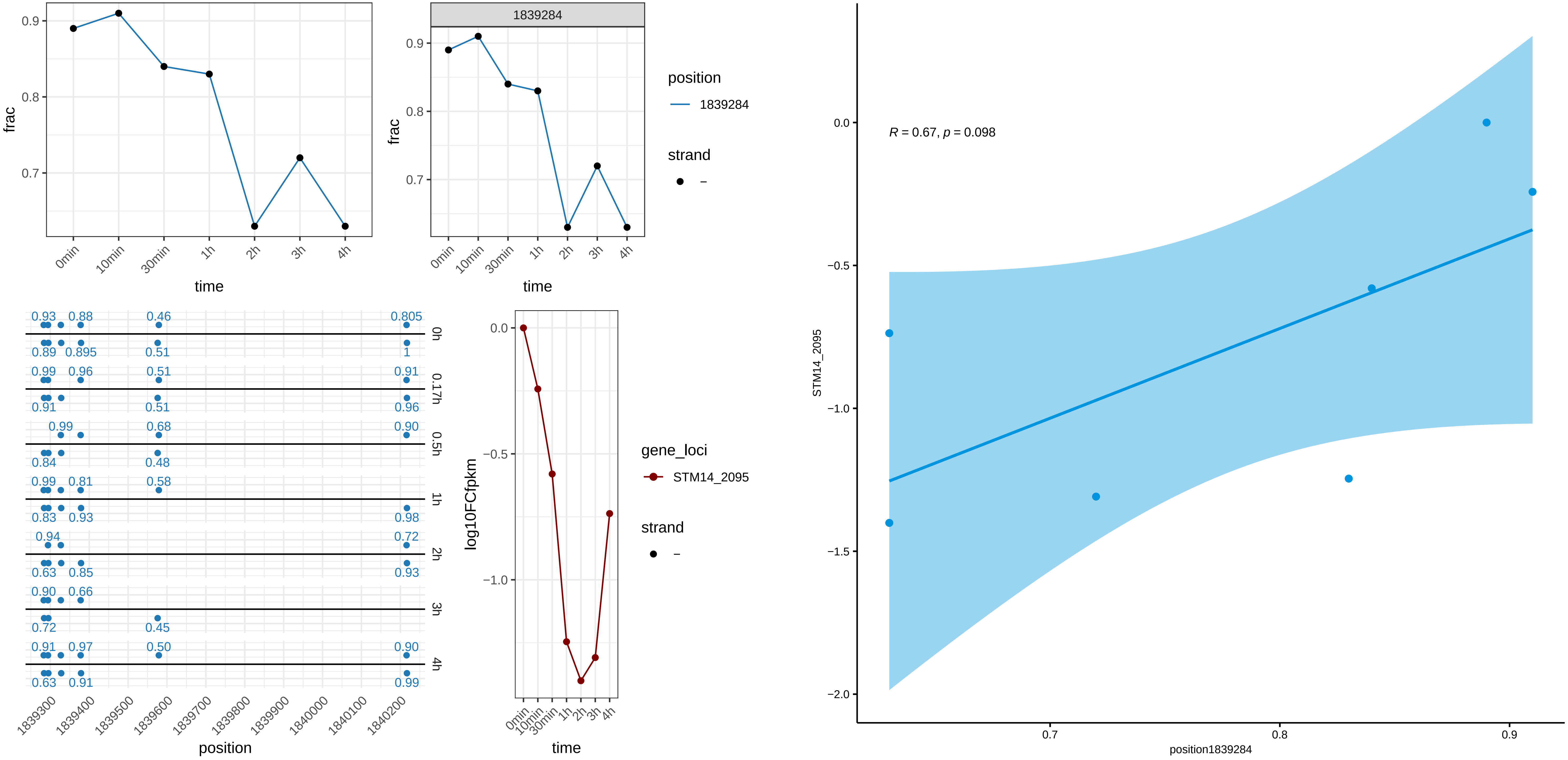

frac
time
1839284
position
1839284
strand
log10FCfpkm
gene_loci
STM14_2095
R = 0.67, p = 0.098
STM14_2095
position1839284

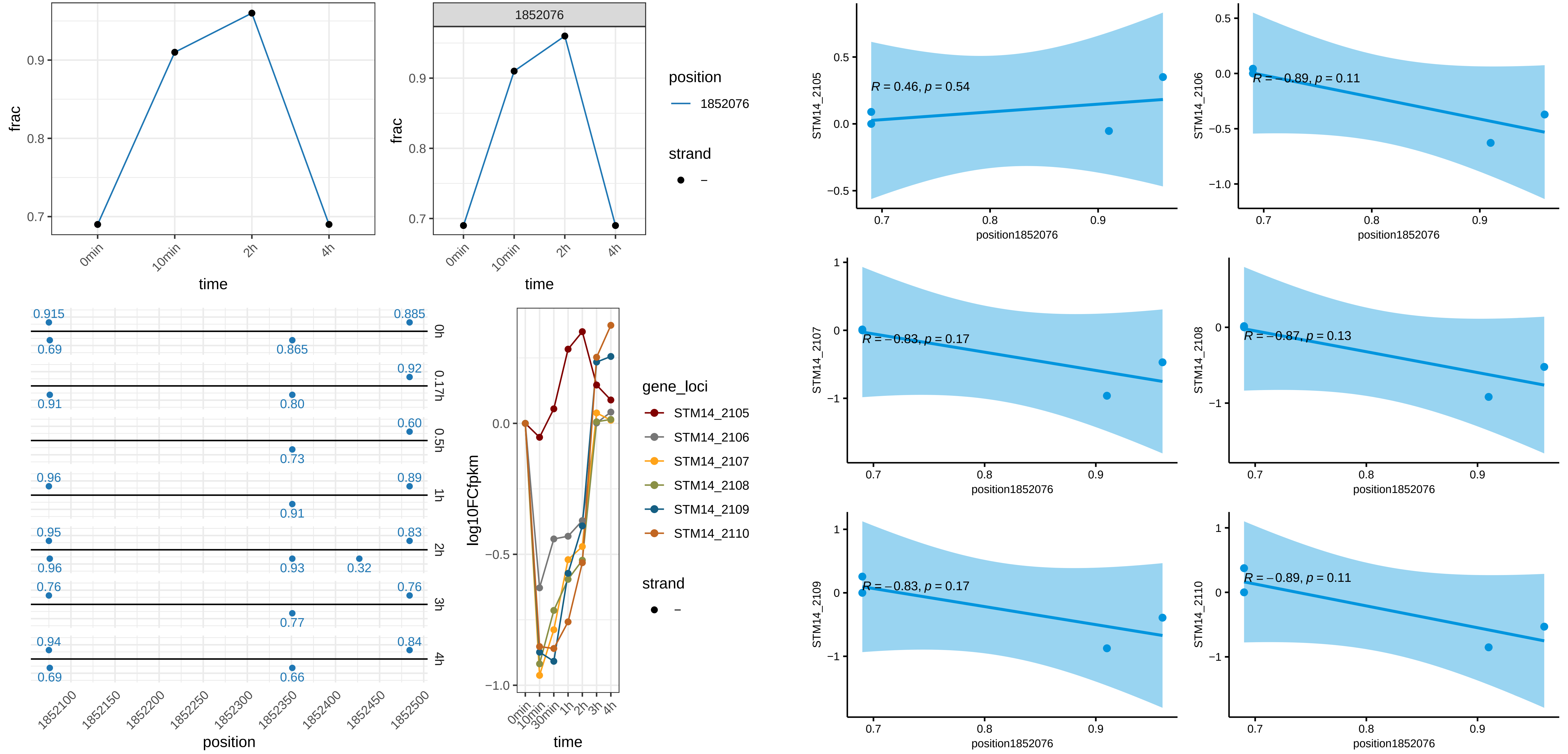

frac
0.7
0.8
0.9
0min
10min
2h
4h
time
1852076
position
1852076
strand
–
0.915
0.885
0.69
0.865
0h
0.92
0.91
0.80
0.17h
0.60
0.73
0.5h
0.96
0.89
0.91
1h
0.95
0.83
0.96
0.93
0.32
2h
0.76
0.76
0.77
3h
0.94
0.84
0.69
0.66
4h
1852100
1852150
1852200
1852250
1852300
1852350
1852400
1852450
1852500
position
log10FCfpkm
0.0
−0.5
−1.0
0min
10min
30min
1h
2h
3h
4h
gene_loci
STM14_2105
STM14_2106
STM14_2107
STM14_2108
STM14_2109
STM14_2110
R = 0.46, p = 0.54
STM14_2105
R = −0.89, p = 0.11
STM14_2106
R = −0.83, p = 0.17
STM14_2107
R = −0.87, p = 0.13
STM14_2108
R = −0.83, p = 0.17
STM14_2109
R = −0.89, p = 0.11
STM14_2110
position1852076
0.7
0.8
0.9

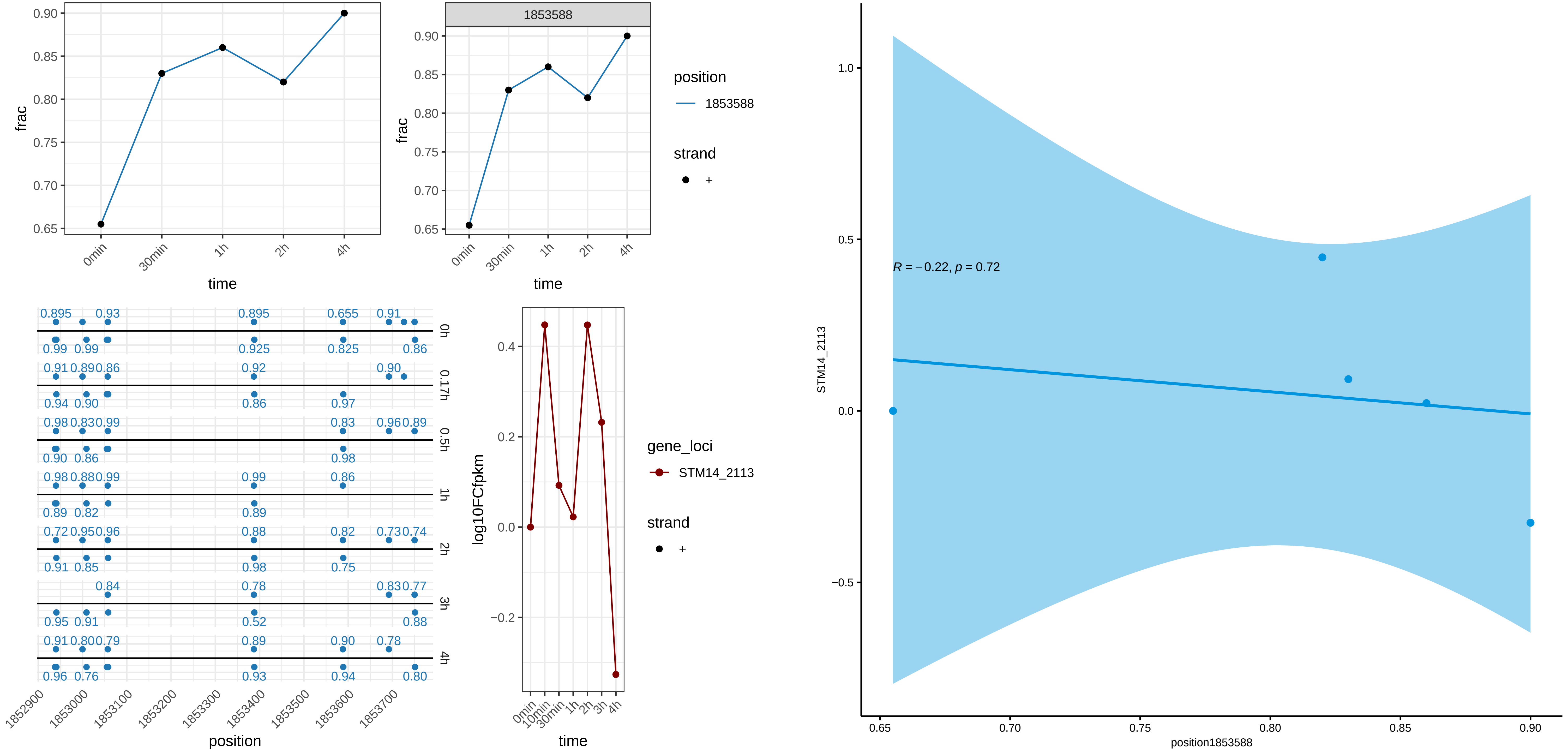

frac
time
0min
30min
1h
2h
4h
1853588
position
1853588
strand
+
position
log10FCfpkm
gene_loci
STM14_2113
0min
10min
30min
1h
2h
3h
4h
R = −0.22, p = 0.72
STM14_2113
position1853588

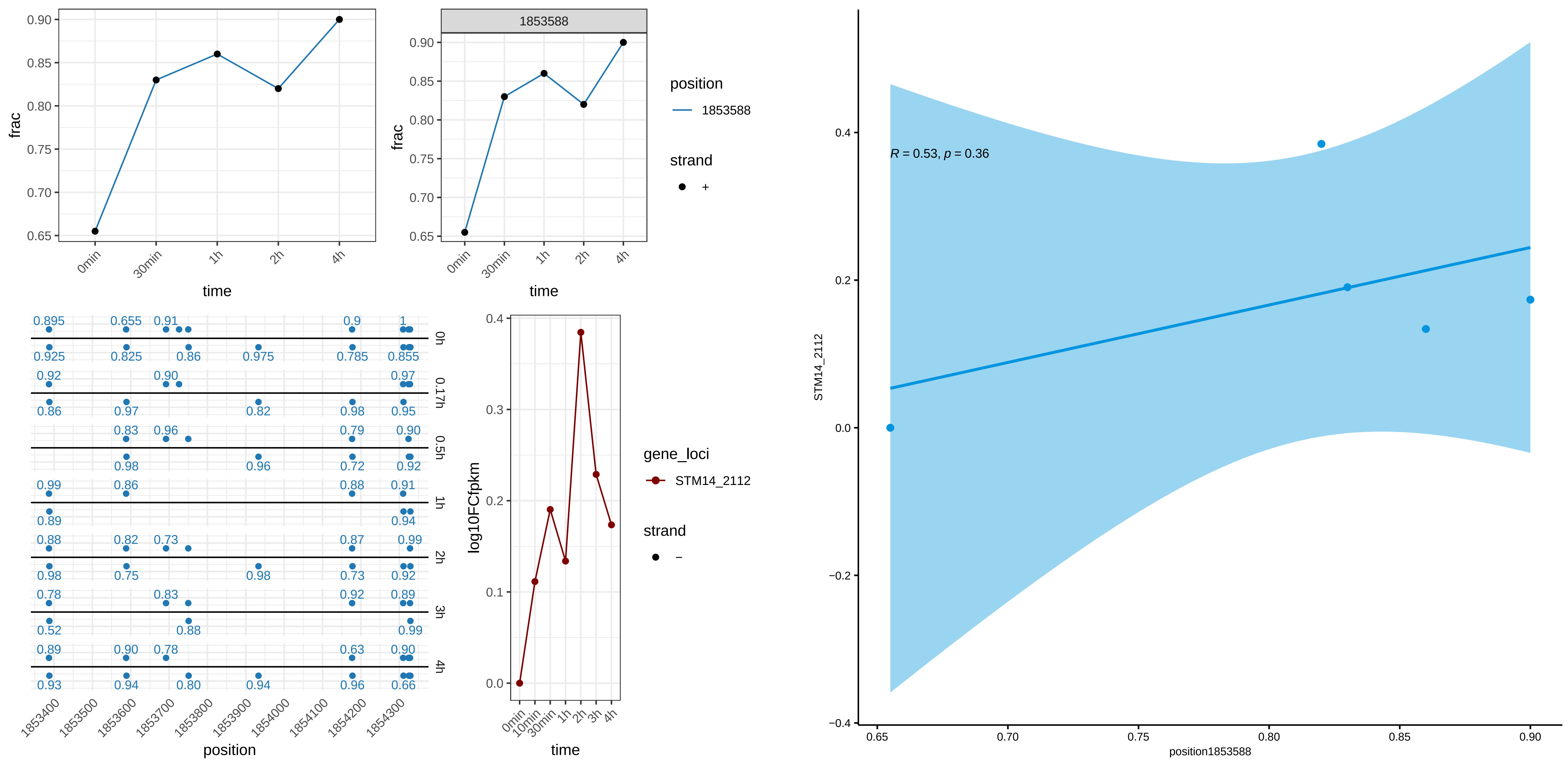

frac
time
0min
30min
1h
2h
4h
1853588
position
1853588
strand
+
position
0h
0.17h
0.5h
1h
2h
3h
4h
log10FCfpkm
10min
3h
gene_loci
STM14_2112
strand
–
R = 0.53, p = 0.36
STM14_2112
position1853588

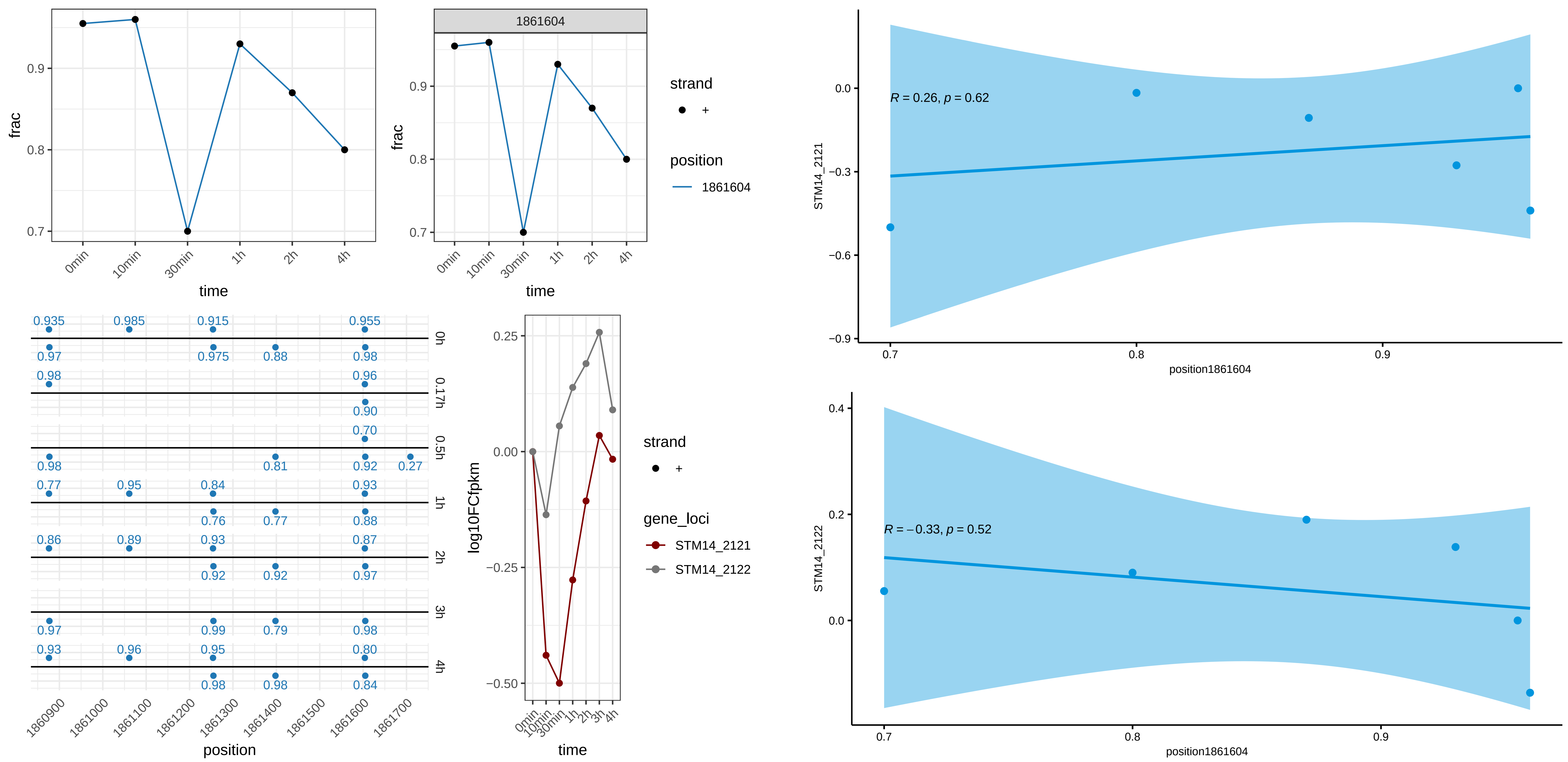

frac
time
0min
10min
30min
1h
2h
4h
1861604
strand
+
position
1861604
R = 0.26, p = 0.62
STM14_2121
position1861604
R = −0.33, p = 0.52
STM14_2122
position
log10FCfpkm
gene_loci
STM14_2121
STM14_2122
0h
0.17h
0.5h
3h

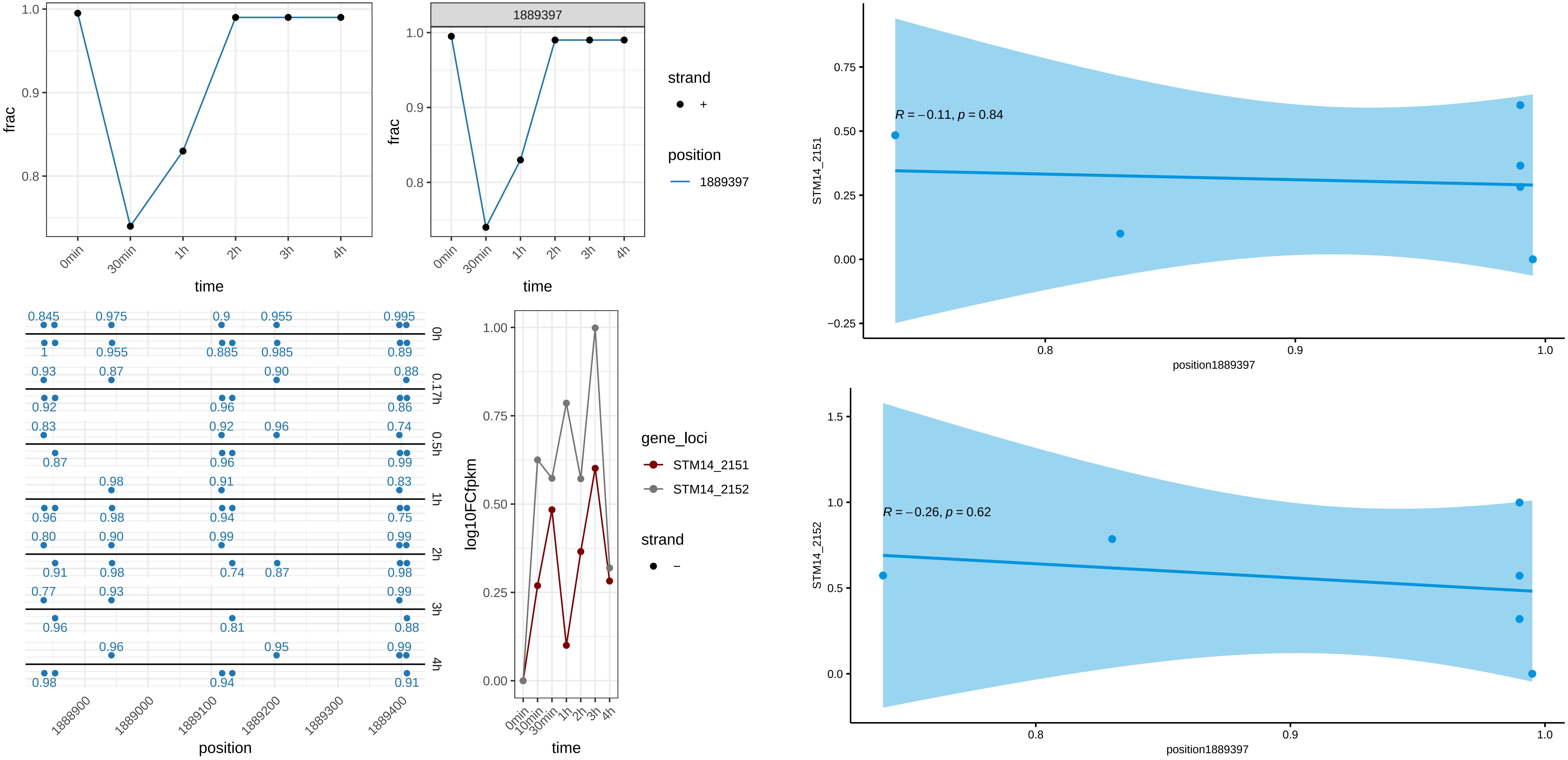

frac
time
0min
30min
1h
2h
3h
4h
1889397
strand
+
position
1889397
position
0h
0.17h
0.5h
1h
2h
3h
4h
log10FCfpkm
gene_loci
STM14_2151
STM14_2152
strand
–
R = –0.11, p = 0.84
STM14_2151
position1889397
R = –0.26, p = 0.62
STM14_2152
position1889397

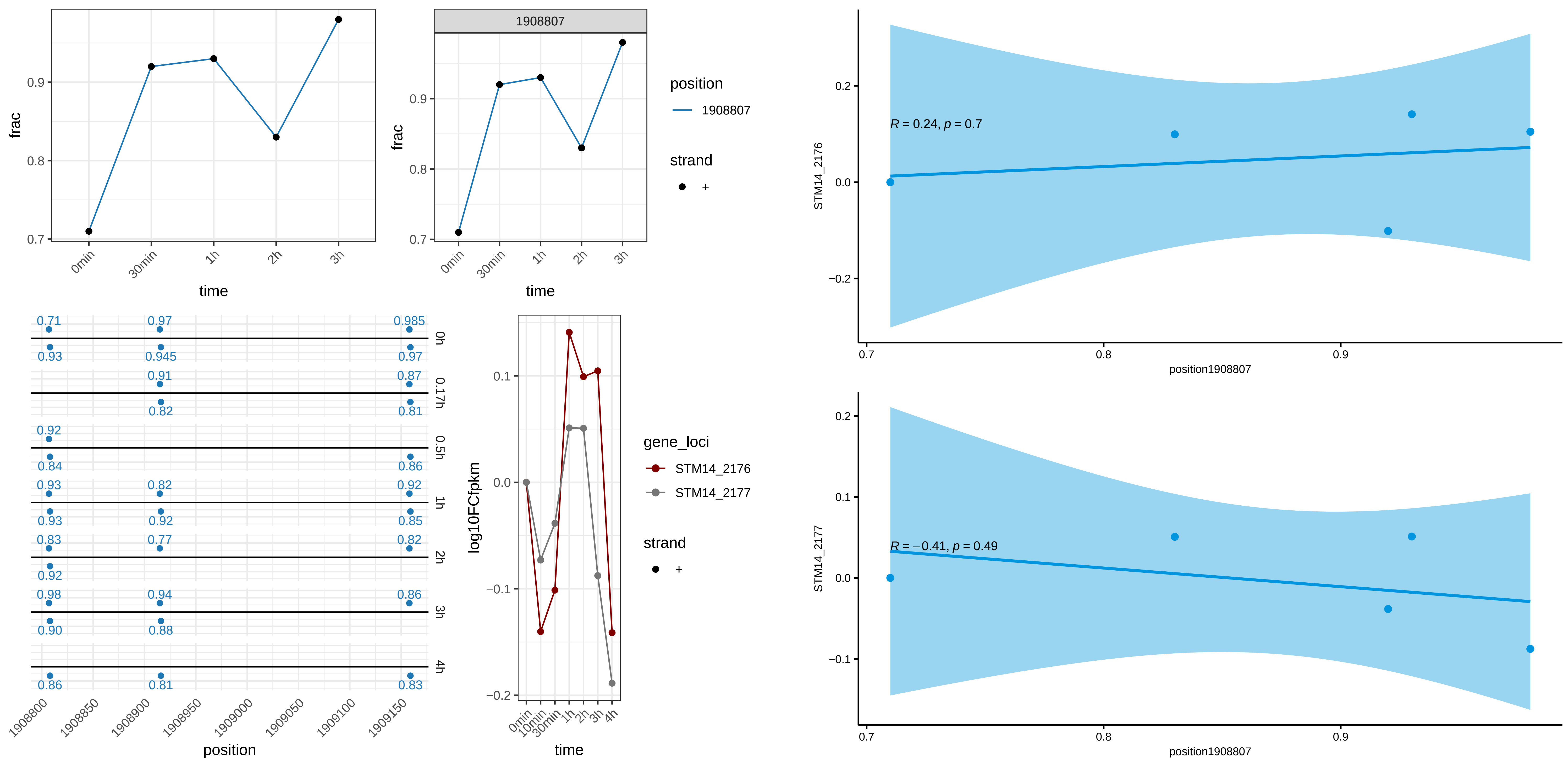

frac
time
0min
30min
1h
2h
3h
1908807
position
strand
+
position
0h
0.17h
0.5h
1h
2h
3h
4h
log10FCfpkm
gene_loci
STM14_2176
STM14_2177
10min
4h
R = 0.24, p = 0.7
STM14_2176
position1908807
R = −0.41, p = 0.49
STM14_2177

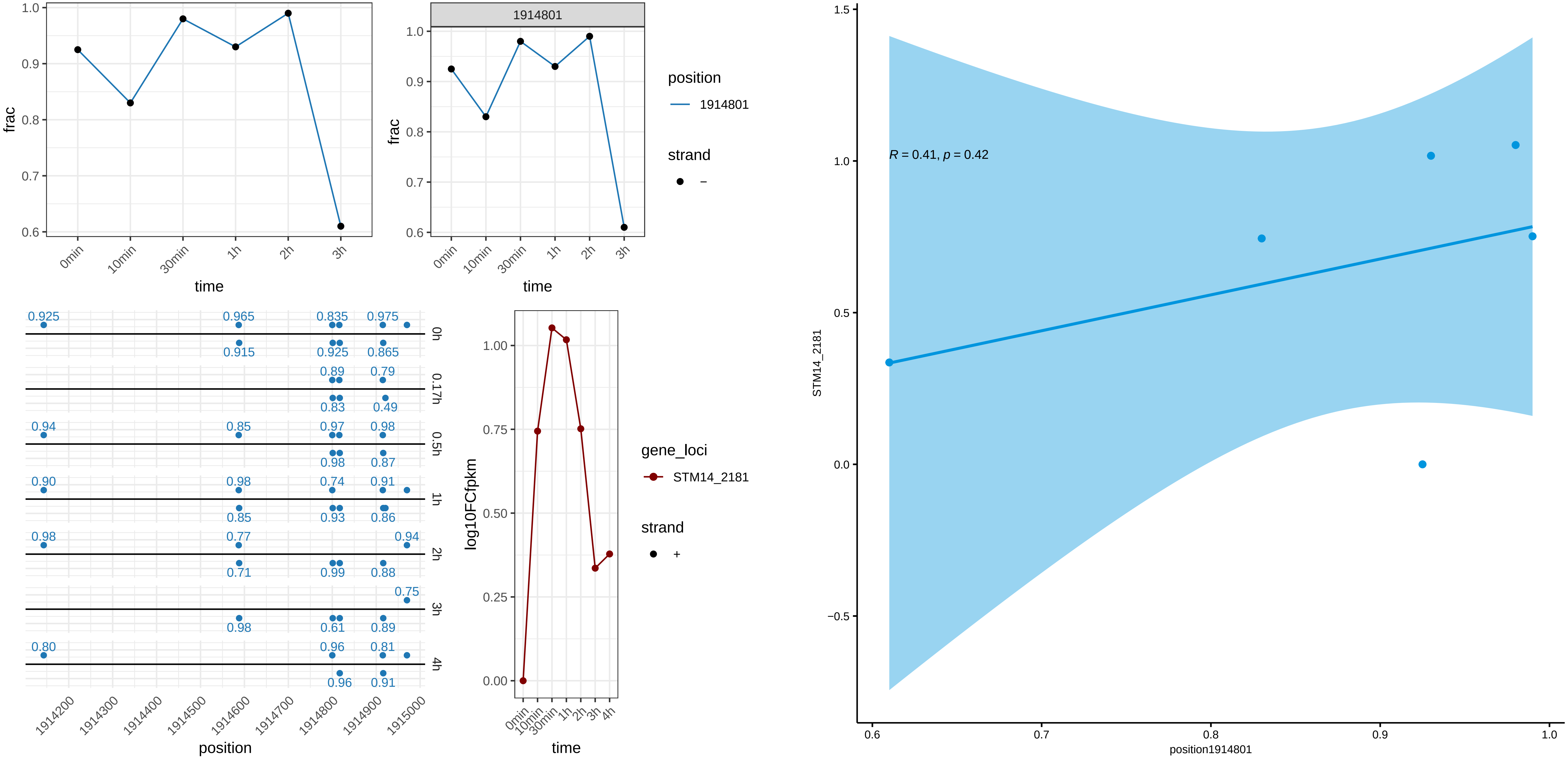

frac
time
0min
10min
30min
1h
2h
3h
1914801
position
1914801
strand
–
0.925
0.965
0.835
0.975
0.915
0.925
0.865
0.89
0.79
0.83
0.49
0.94
0.85
0.97
0.98
0.98
0.87
0.90
0.98
0.74
0.91
0.85
0.93
0.86
0.98
0.77
0.94
0.71
0.99
0.88
0.75
0.98
0.61
0.89
0.80
0.96
0.81
0.96
0.91
0h
0.17h
0.5h
1h
2h
3h
4h
position
log10FCfpkm
4h
gene_loci
STM14_2181
strand
+
R = 0.41, p = 0.42
STM14_2181
position1914801

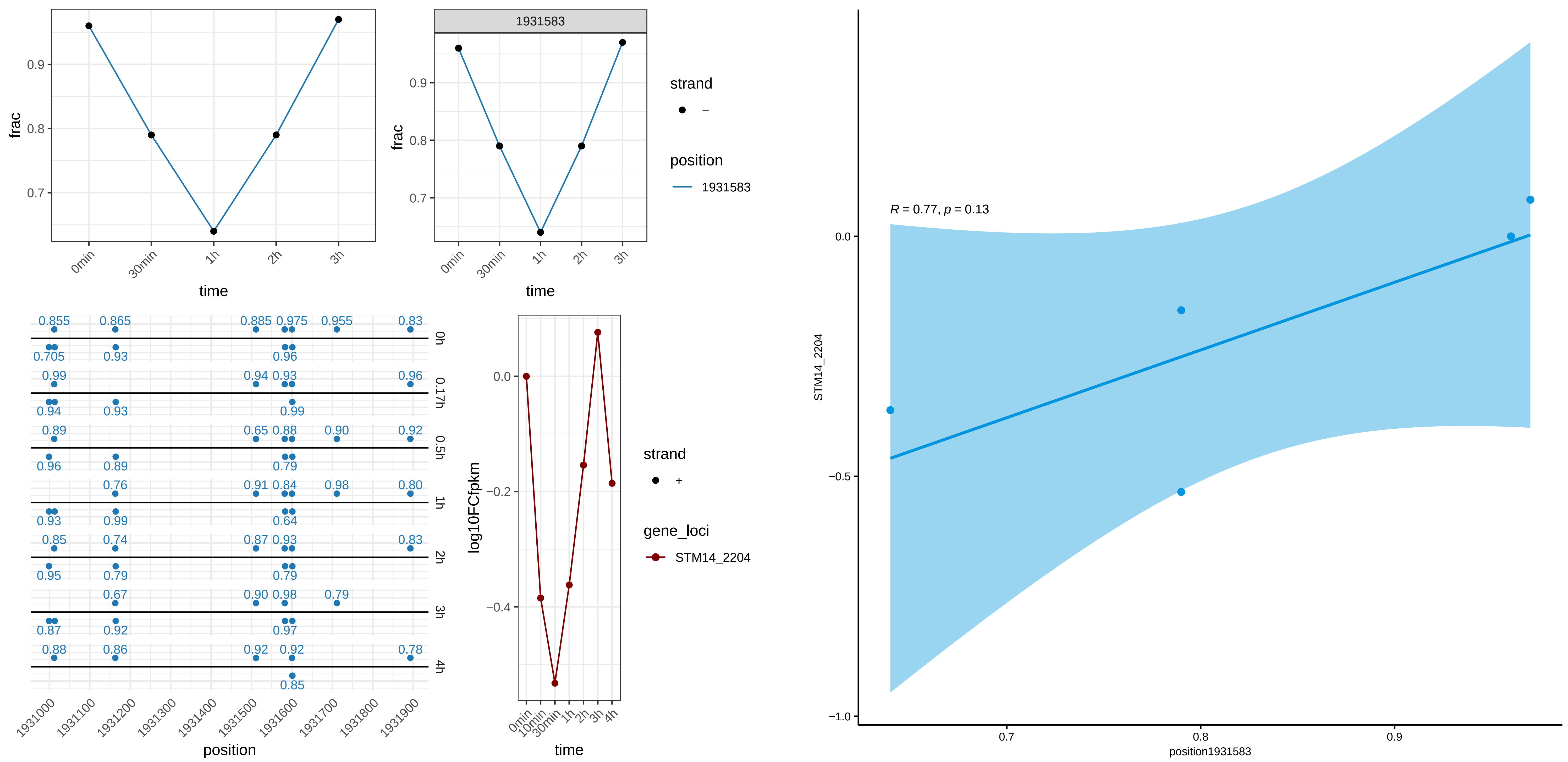

frac
time
0min
30min
1h
2h
3h
1931583
strand
–
position
1931583
0.855 0.865 0.885 0.975 0.955 0.83
0.705 0.93 0.96
0h
0.99 0.94 0.93 0.96
0.94 0.93 0.99
0.17h
0.89 0.65 0.88 0.90 0.92
0.96 0.89 0.79
0.5h
0.76 0.91 0.84 0.98 0.80
0.93 0.99 0.64
1h
0.85 0.74 0.87 0.93 0.83
0.95 0.79 0.79
2h
0.67 0.90 0.98 0.79
0.87 0.92 0.97
3h
0.88 0.86 0.92 0.92 0.78
0.85
4h
position
log10FCfpkm
0min
10min
30min
1h
2h
3h
4h
time
strand
+
gene_loci
STM14_2204
R = 0.77, p = 0.13
STM14_2204
position1931583

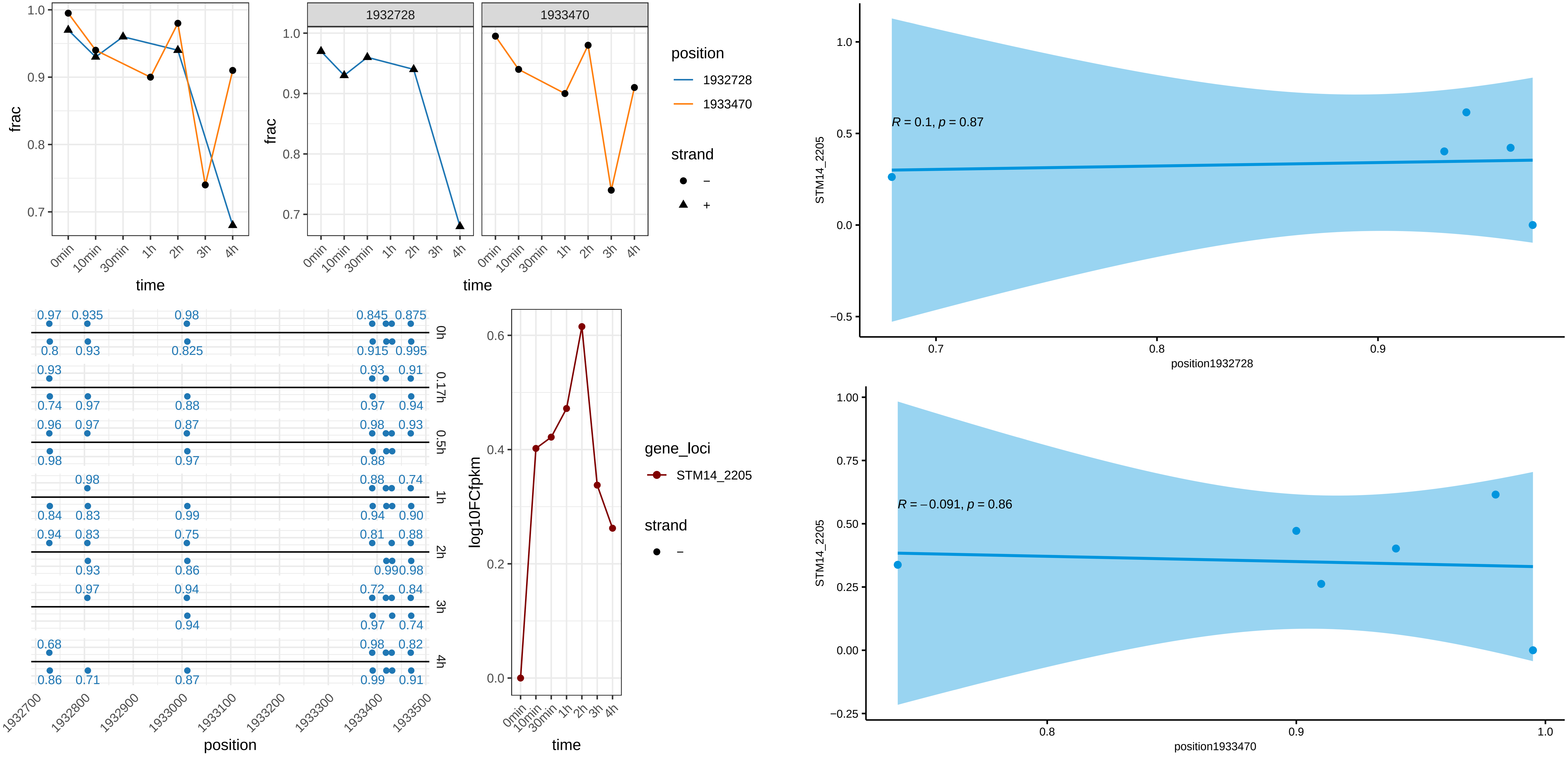

frac
time
0min
10min
30min
1h
2h
3h
4h
1932728
1933470
position
1932728
1933470
strand
–
+
position
0h
0.17h
0.5h
1h
2h
3h
4h
1932700
1932800
1932900
1933000
1933100
1933200
1933300
1933400
1933500
log10FCfpkm
gene_loci
STM14_2205
strand
–
R = 0.1, p = 0.87
STM14_2205
position1932728
R = –0.091, p = 0.86
STM14_2205
position1933470

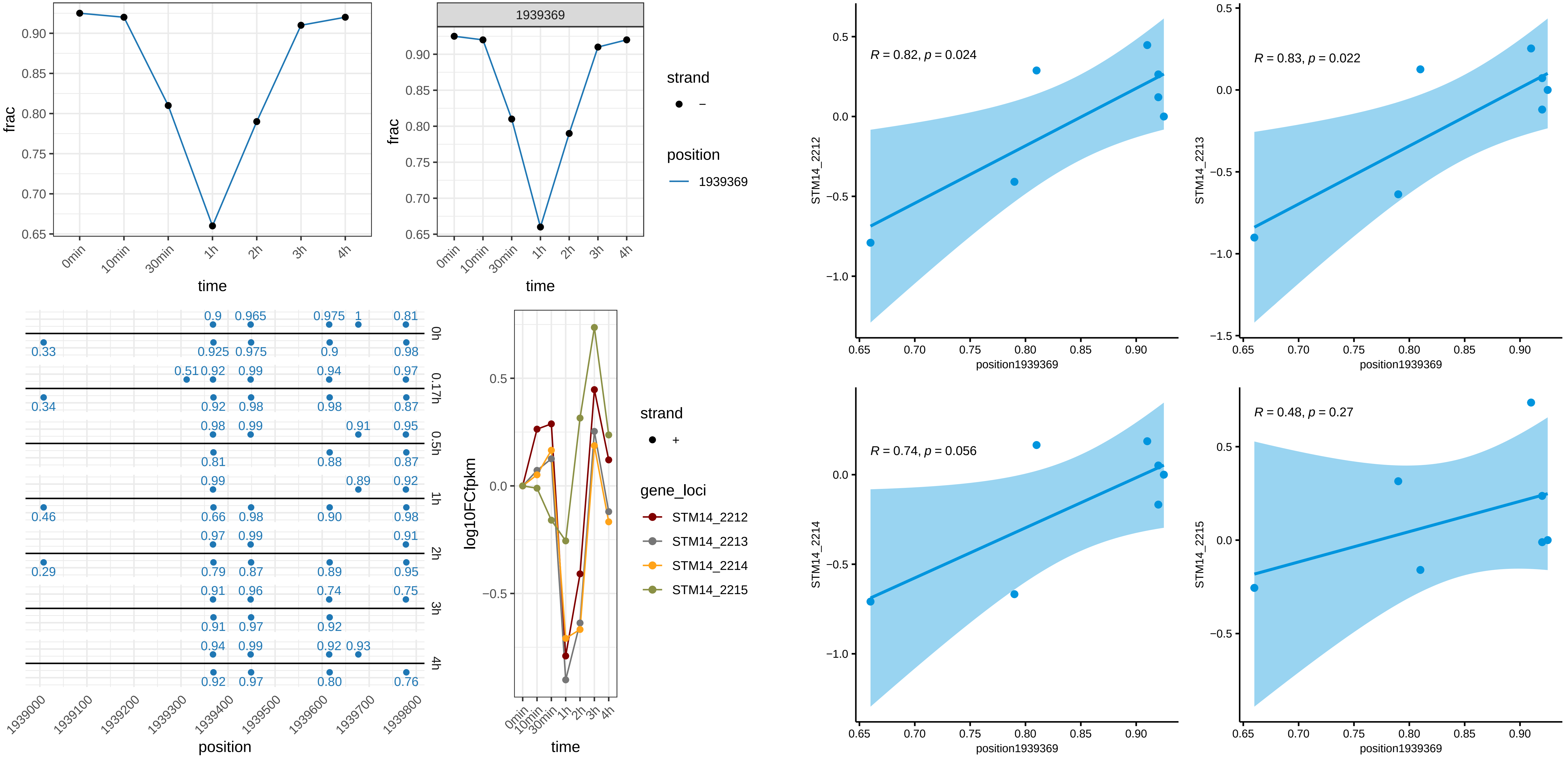

frac
time
0min
10min
30min
1h
2h
3h
4h
1939369
strand
–
position
1939369
R = 0.82, p = 0.024
R = 0.83, p = 0.022
R = 0.74, p = 0.056
R = 0.48, p = 0.27
STM14_2212
STM14_2213
STM14_2214
STM14_2215
position1939369
0h
0.17h
0.5h
1h
2h
3h
4h
position
log10FCfpkm
strand
+
gene_loci
STM14_2212
STM14_2213
STM14_2214
STM14_2215

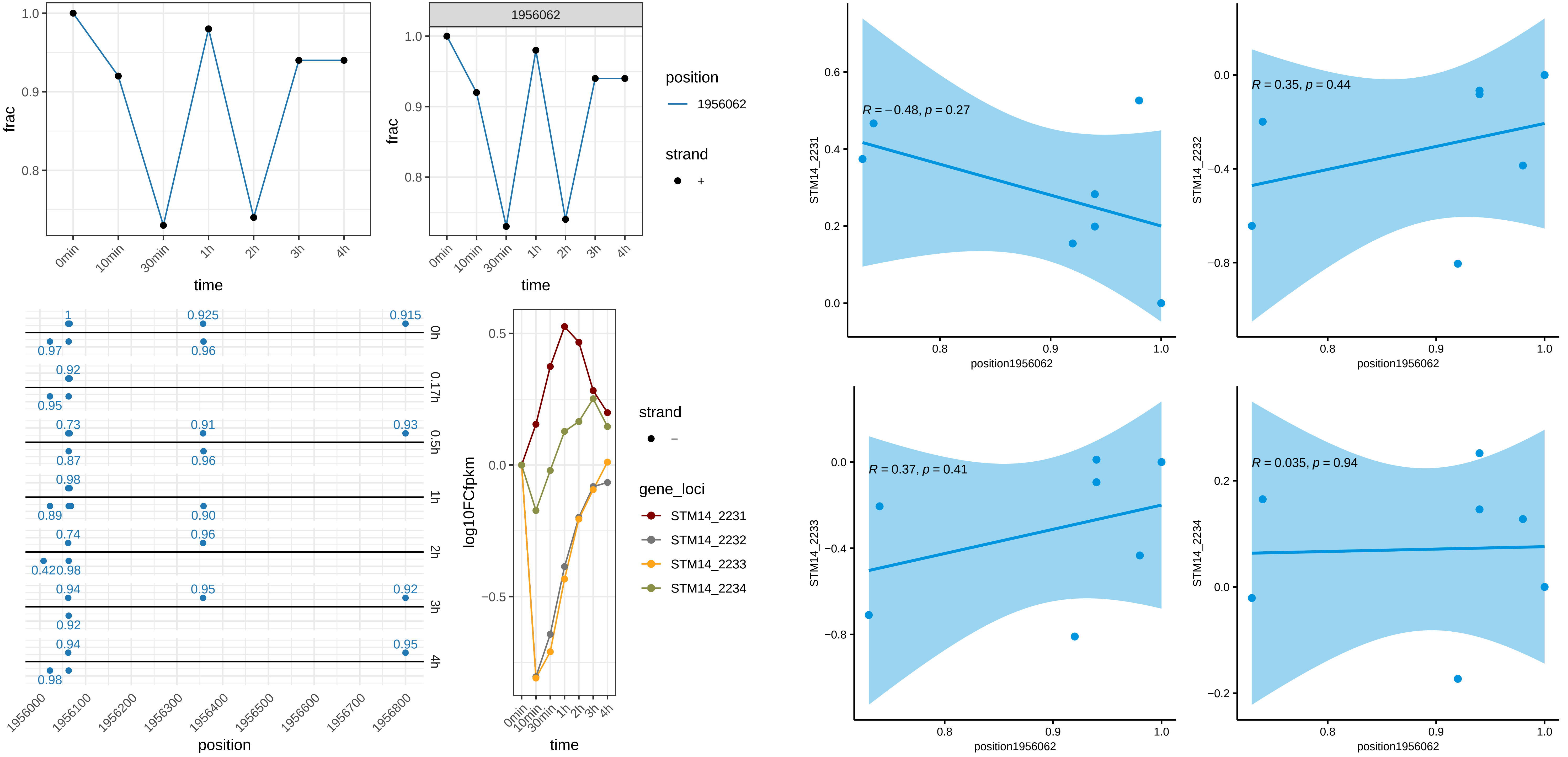

frac
time
0min
10min
30min
1h
2h
3h
4h
1956062
position
strand
+
−
gene_loci
STM14_2231
STM14_2232
STM14_2233
STM14_2234
log10FCfpkm
position
0h
0.17h
0.5h
1h
2h
3h
4h
R = −0.48, p = 0.27
R = 0.35, p = 0.44
R = 0.37, p = 0.41
R = 0.035, p = 0.94
position1956062

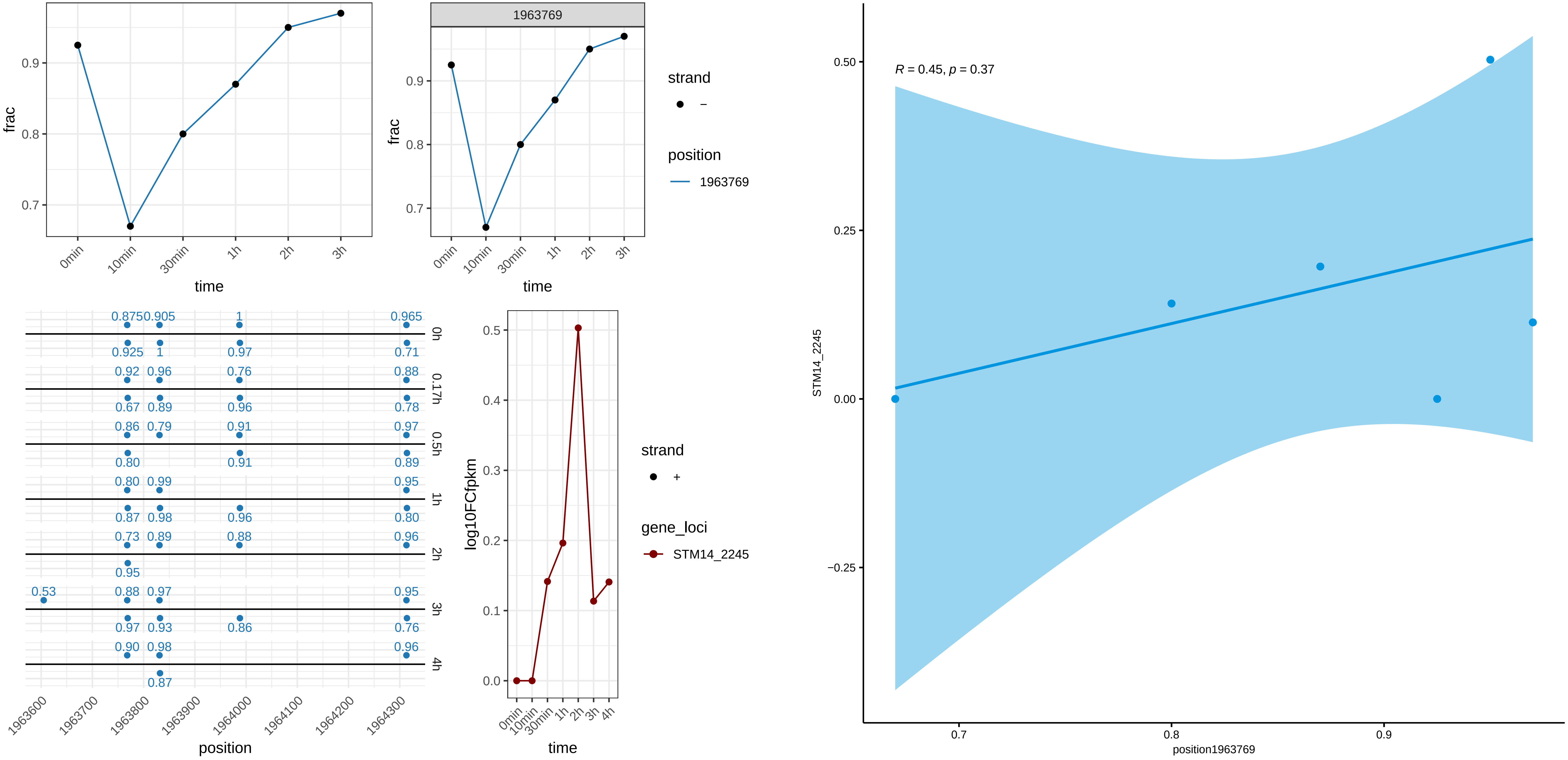

frac
time
0min
10min
30min
1h
2h
3h
1963769
strand
–
position
1963769
0h
0.17h
0.5h
4h
position
log10FCfpkm
strand
+
gene_loci
STM14_2245
R = 0.45, p = 0.37
STM14_2245
position1963769

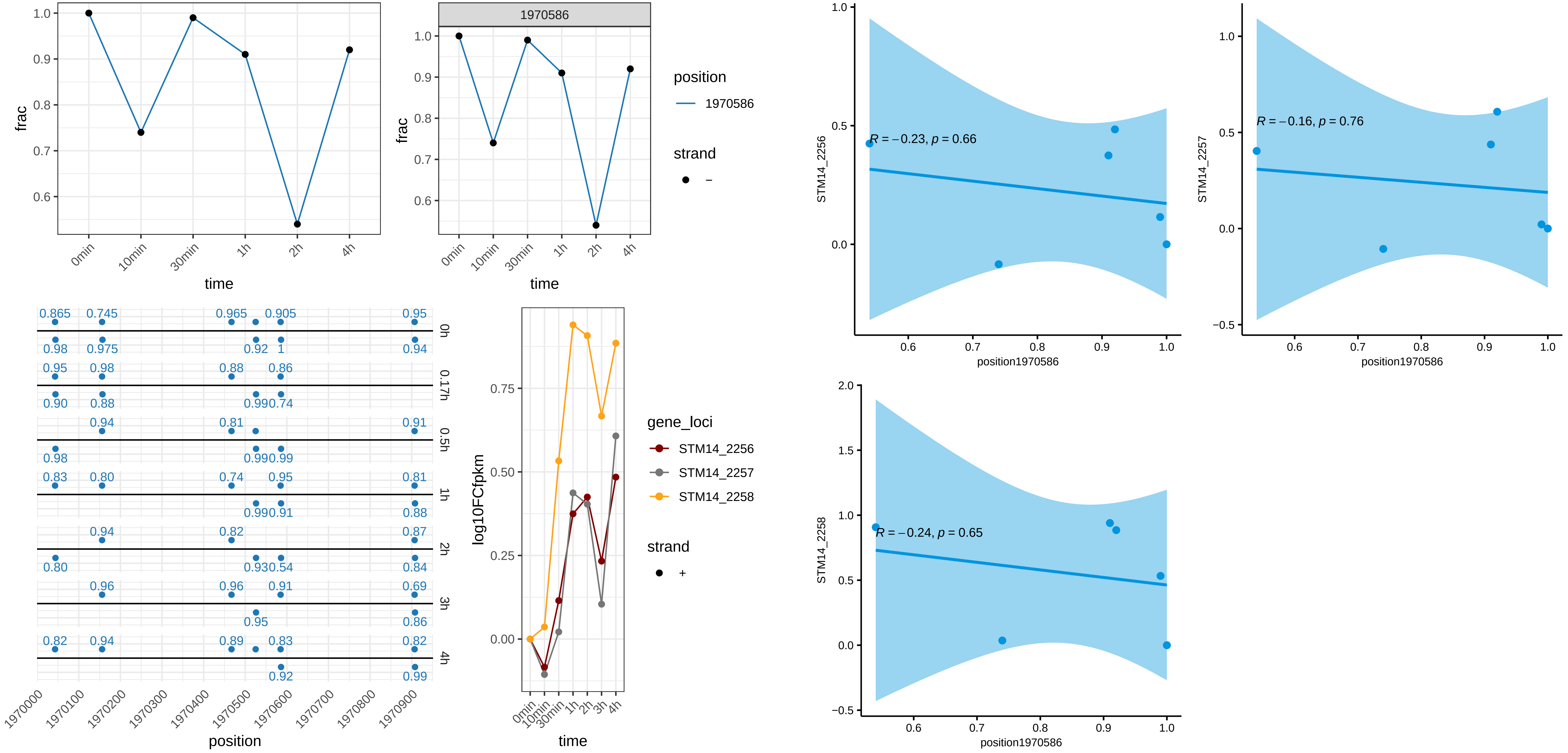

frac
time
0min
10min
30min
1h
2h
4h
1970586
position
1970586
strand
−
0.865
0.745
0.965
0.905
0.95
0.98
0.975
0.92
1
0.94
0h
0.17h
0.5h
1h
2h
3h
4h
position
log10FCfpkm
gene_loci
STM14_2256
STM14_2257
STM14_2258
strand
+
R = −0.23, p = 0.66
STM14_2256
position1970586
R = −0.16, p = 0.76
STM14_2257
position1970586
R = −0.24, p = 0.65
STM14_2258
position1970586

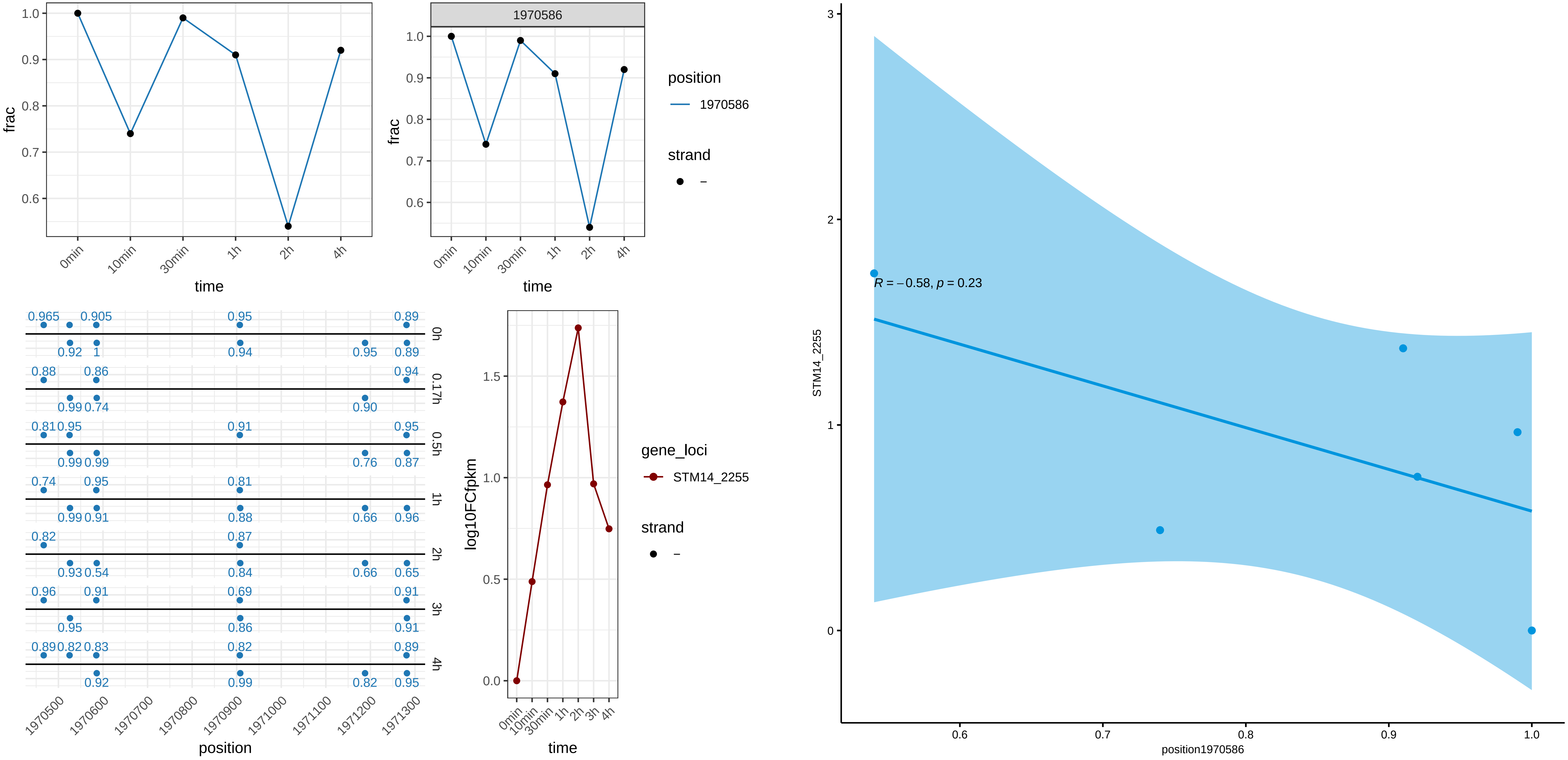

frac
time
0min
10min
30min
1h
2h
4h
1970586
position
strand
–
log10FCfpkm
gene_loci
STM14_2255
position
0h
0.17h
0.5h
1h
2h
3h
4h
R = −0.58, p = 0.23
STM14_2255
position1970586

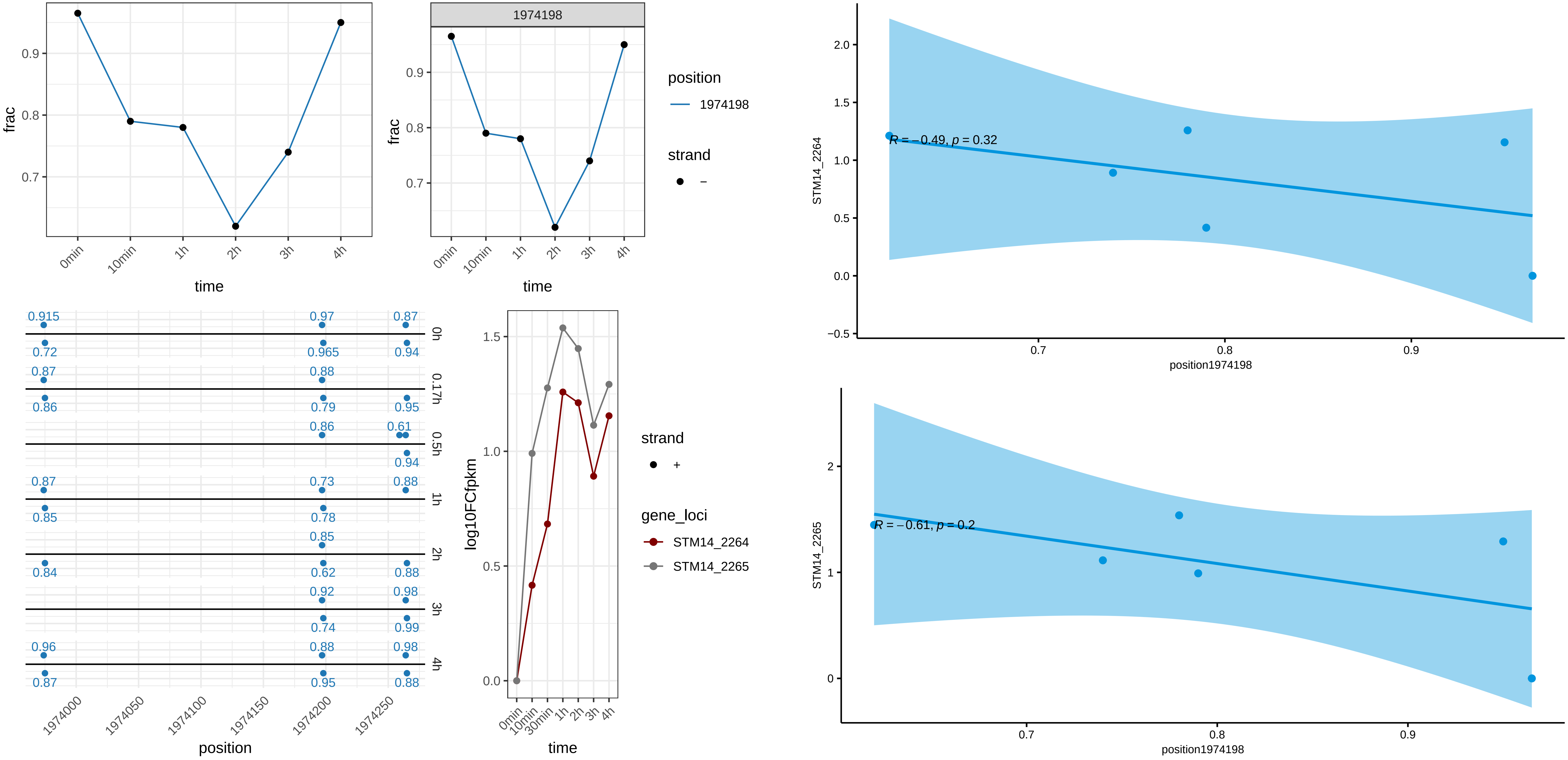
frac
0.9
0.8
0.7
0min
10min
1h
2h
3h
4h
time
1974198
position
1974198
strand
−
0.915
0.97
0.87
0.72
0.965
0.94
0h
0.87
0.88
0.86
0.79
0.95
0.17h
0.86
0.61
0.94
0.5h
0.87
0.73
0.88
0.85
0.78
1h
0.85
0.84
0.62
0.88
2h
0.92
0.98
0.74
0.99
3h
0.96
0.88
0.98
0.87
0.95
0.88
4h
1974000
1974050
1974100
1974150
1974200
1974250
position
log10FCfpkm
1.5
1.0
0.5
0.0
0min
10min
30min
1h
2h
3h
4h
time
strand
+
gene_loci
STM14_2264
STM14_2265
STM14_2264
R = −0.49, p = 0.32
2.0
1.5
1.0
0.5
0.0
−0.5
0.7
0.8
0.9
position1974198
STM14_2265
R = −0.61, p = 0.2
2
1
0
0.7
0.8
0.9
position1974198

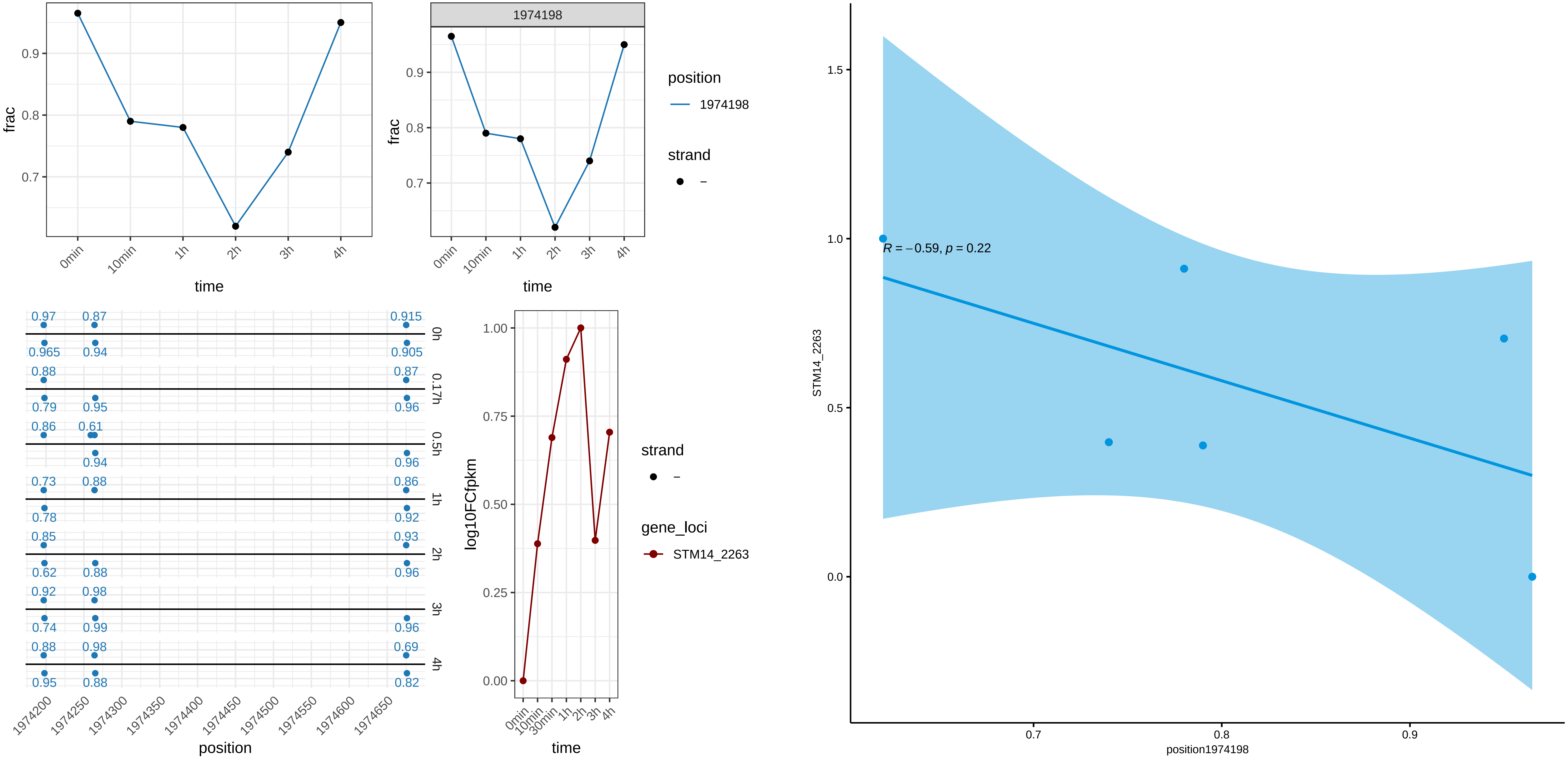

frac
time
0min
10min
1h
2h
3h
4h
1974198
position
1974198
strand
–
0.97
0.87
0.915
0.965
0.94
0.905
0h
0.88
0.87
0.79
0.95
0.96
0.17h
0.86
0.61
0.94
0.96
0.5h
0.73
0.88
0.86
0.78
0.92
1h
0.85
0.93
0.62
0.88
0.96
2h
0.92
0.98
0.74
0.99
0.96
3h
0.88
0.98
0.69
0.95
0.88
0.82
4h
1974200
1974250
1974300
1974350
1974400
1974450
1974500
1974550
1974600
1974650
position
log10FCfpkm
30min
gene_loci
STM14_2263
R = −0.59, p = 0.22
STM14_2263
position1974198

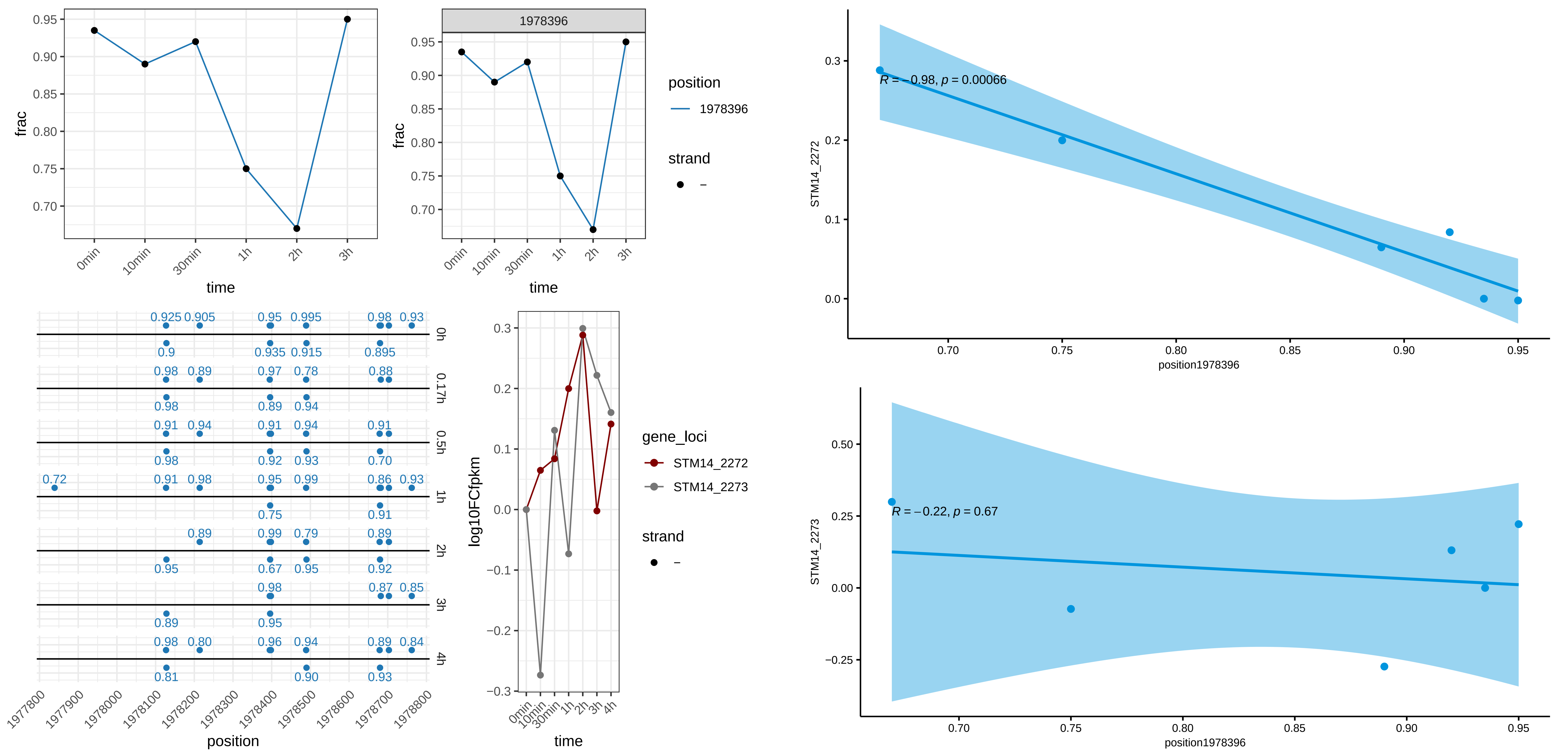

frac
time
0min
10min
30min
1h
2h
3h
1978396
position
1978396
strand
–
0h
0.17h
0.5h
1h
2h
3h
4h
0.925
0.905
0.95
0.995
0.98
0.93
0.9
0.935
0.915
0.895
0.98
0.89
0.97
0.78
0.88
0.98
0.89
0.94
0.91
0.94
0.91
0.94
0.91
0.98
0.92
0.93
0.70
0.72
0.91
0.98
0.95
0.99
0.86
0.93
0.75
0.91
0.89
0.99
0.79
0.89
0.95
0.67
0.95
0.92
0.98
0.87
0.85
0.89
0.95
0.98
0.80
0.96
0.94
0.89
0.84
0.81
0.90
0.93
position
log10FCfpkm
time
4h
gene_loci
STM14_2272
STM14_2273
strand
–
STM14_2272
R = −0.98, p = 0.00066
position1978396
STM14_2273
R = −0.22, p = 0.67
position1978396

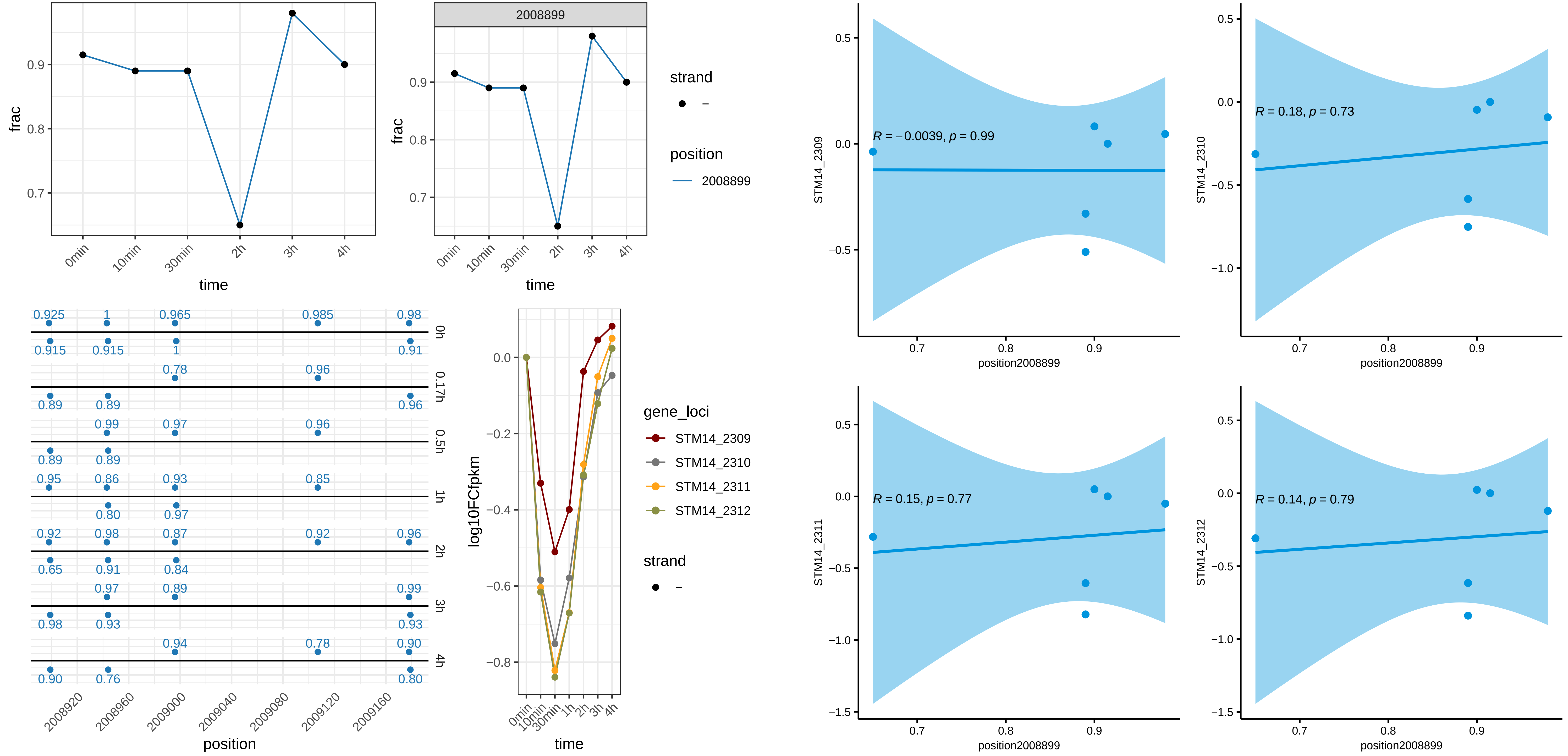

frac
time
0min
10min
30min
2h
3h
4h
2008899
strand
–
position
2008899
0h
0.17h
0.5h
1h
2h
3h
4h
position
log10FCfpkm
gene_loci
STM14_2309
STM14_2310
STM14_2311
STM14_2312
R = −0.0039, p = 0.99
R = 0.18, p = 0.73
R = 0.15, p = 0.77
R = 0.14, p = 0.79
STM14_2309
STM14_2310
STM14_2311
STM14_2312
position2008899

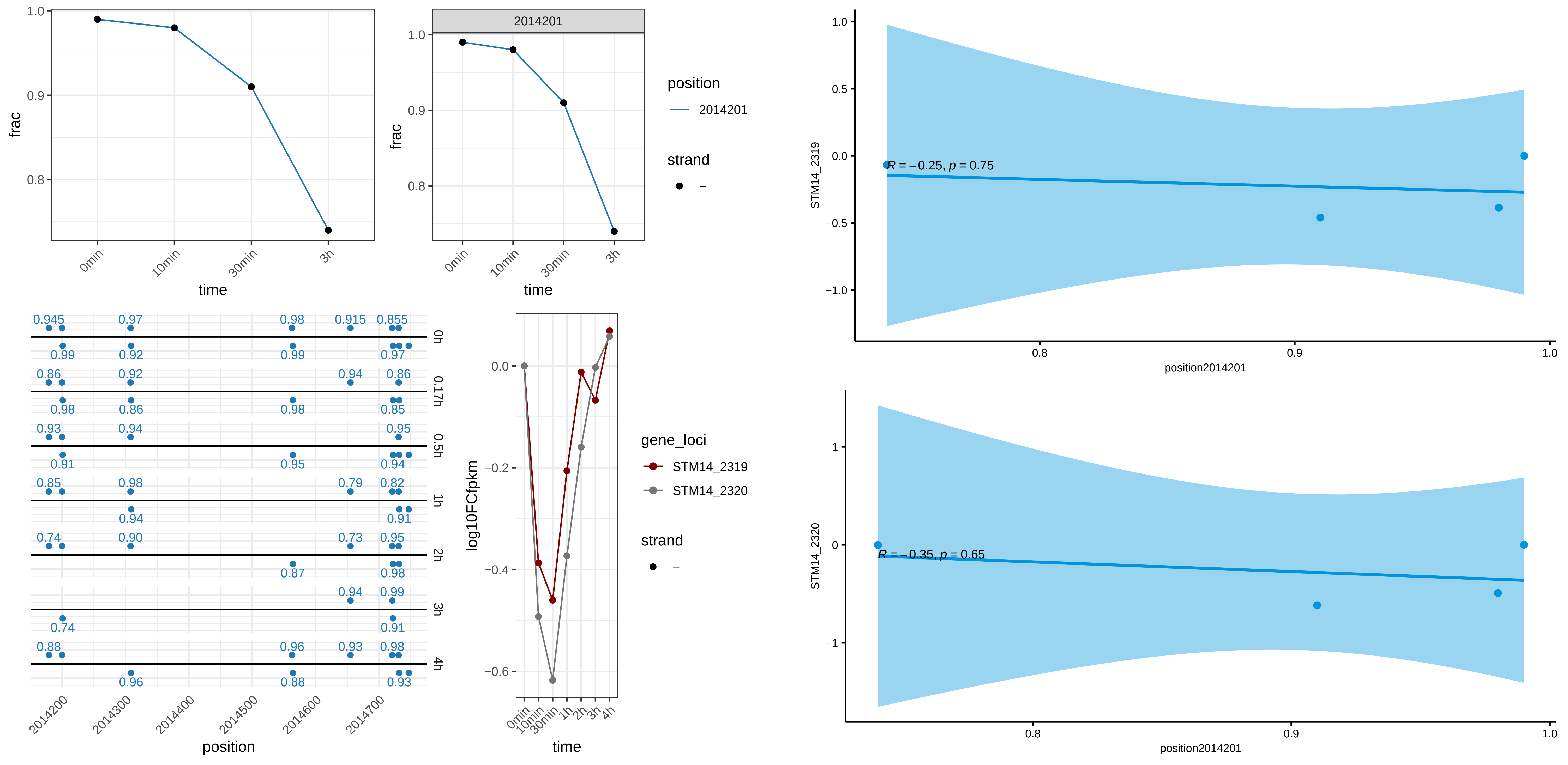
frac
time
0min
10min
30min
3h
2014201
position
strand
–
position
0h
0.17h
0.5h
1h
2h
3h
4h
2014200
2014300
2014400
2014500
2014600
2014700
0.945
0.97
0.98
0.915
0.855
0.99
0.92
0.99
0.97
0.86
0.92
0.94
0.86
0.98
0.86
0.98
0.85
0.93
0.94
0.95
0.91
0.95
0.94
0.85
0.98
0.79
0.82
0.94
0.91
0.74
0.90
0.73
0.95
0.87
0.98
0.94
0.99
0.74
0.91
0.88
0.96
0.93
0.98
0.96
0.88
0.93
log10FCfpkm
1h
2h
4h
gene_loci
STM14_2319
STM14_2320
STM14_2319
R = −0.25, p = 0.75
position2014201
STM14_2320
R = −0.35, p = 0.65
position2014201

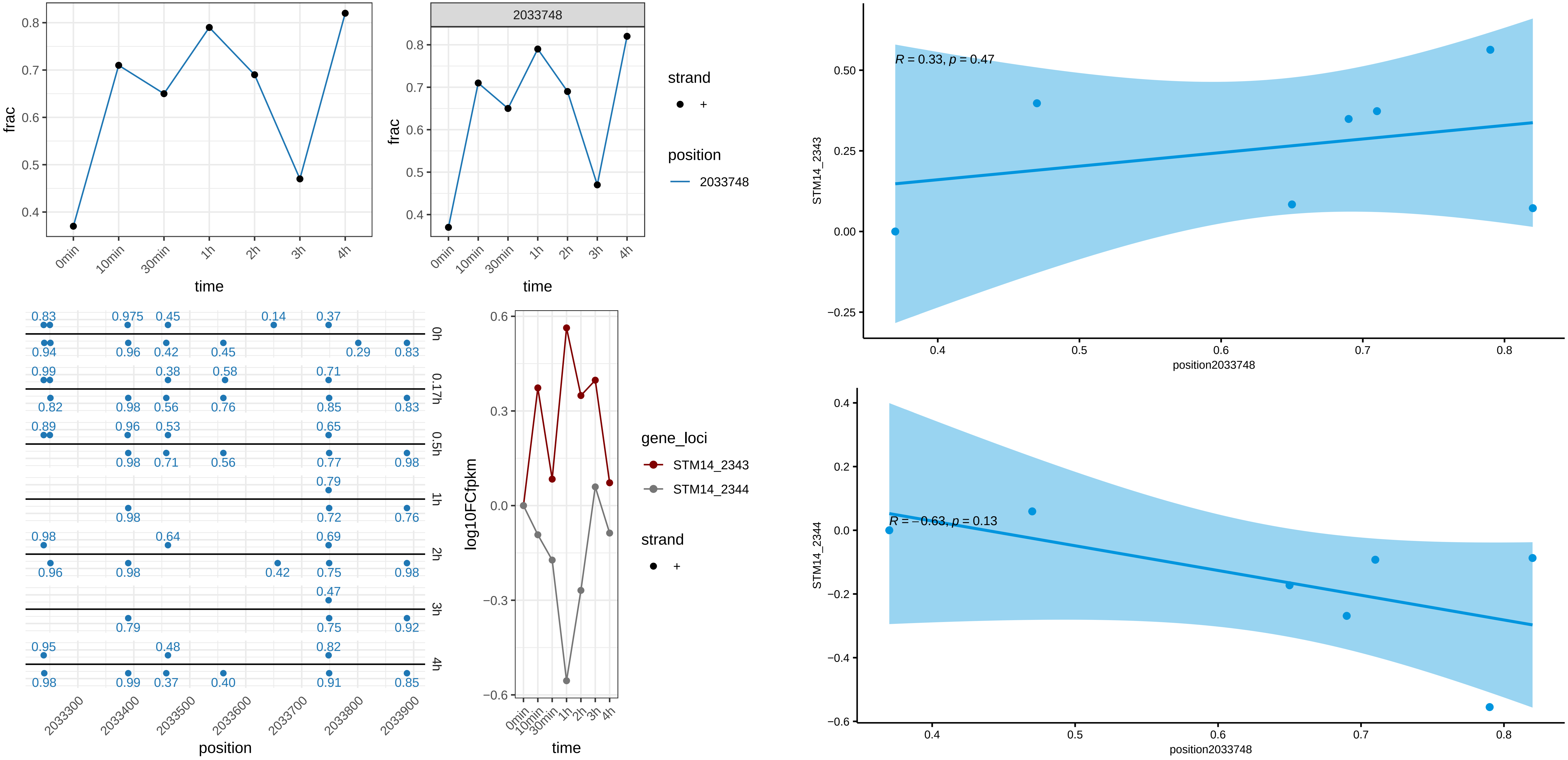
frac
time
0min
10min
30min
1h
2h
3h
4h
2033748
strand
+
position
2033748
R = 0.33, p = 0.47
STM14_2343
position2033748
0.83
0.975
0.45
0.14
0.37
0.94
0.96
0.42
0.45
0.29
0.83
0.99
0.38
0.58
0.71
0.82
0.98
0.56
0.76
0.85
0.83
0.89
0.96
0.53
0.65
0.98
0.71
0.56
0.77
0.98
0.79
0.98
0.72
0.76
0.98
0.64
0.69
0.96
0.98
0.42
0.75
0.98
0.47
0.79
0.75
0.92
0.95
0.48
0.82
0.98
0.99
0.37
0.40
0.91
0.85
0h
0.17h
0.5h
1h
2h
3h
4h
2033300
2033400
2033500
2033600
2033700
2033800
2033900
position
log10FCfpkm
gene_loci
STM14_2343
STM14_2344
R = −0.63, p = 0.13
STM14_2344

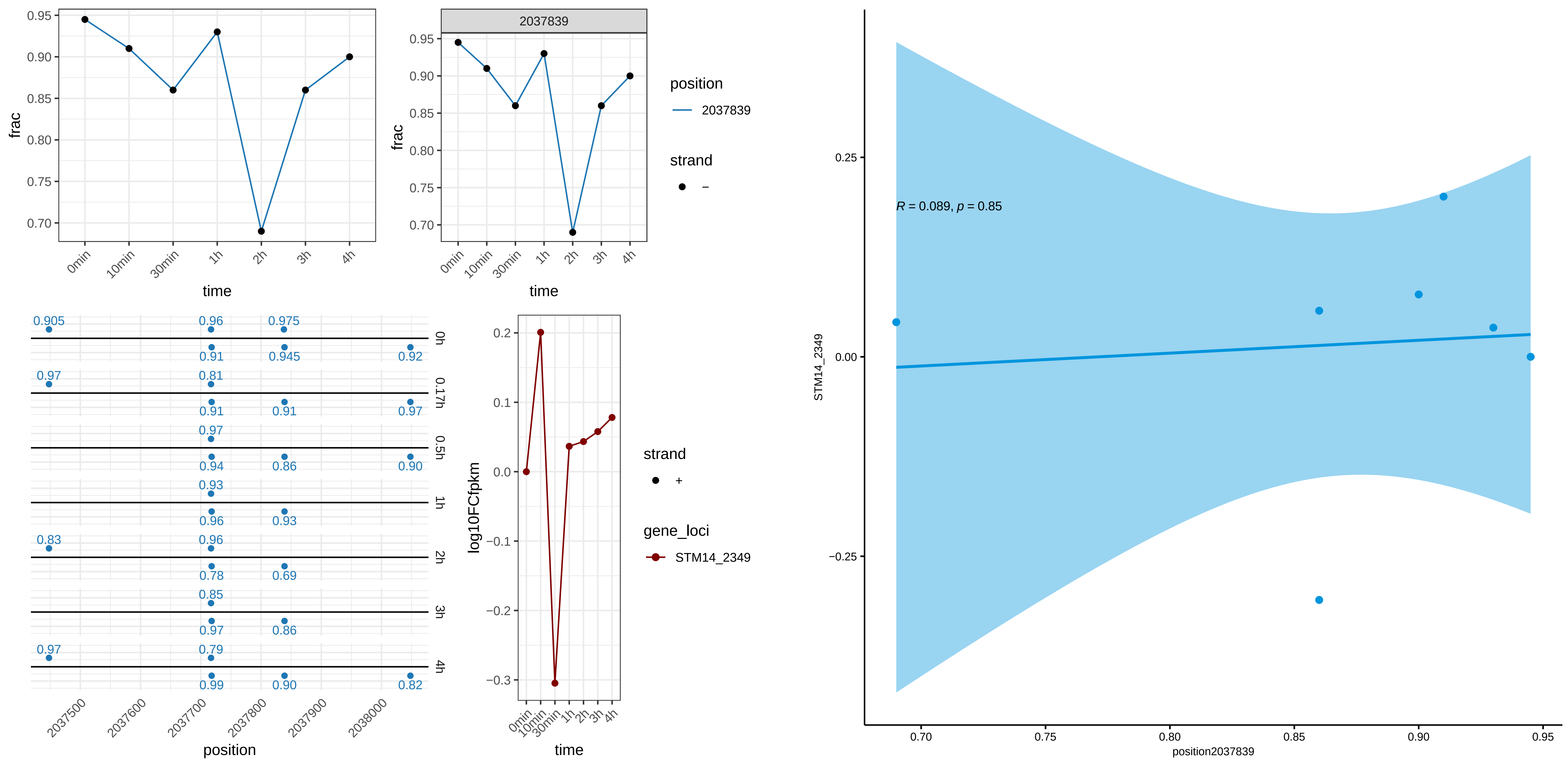

frac
time
2037839
position
strand
−
0.905
0.96
0.975
0.91
0.945
0.92
0.97
0.81
0.91
0.91
0.97
0.97
0.94
0.86
0.90
0.93
0.96
0.93
0.83
0.96
0.78
0.69
0.85
0.97
0.86
0.97
0.79
0.99
0.90
0.82
0h
0.17h
0.5h
1h
2h
3h
4h
log10FCfpkm
+
gene_loci
STM14_2349
R = 0.089, p = 0.85
STM14_2349
position2037839

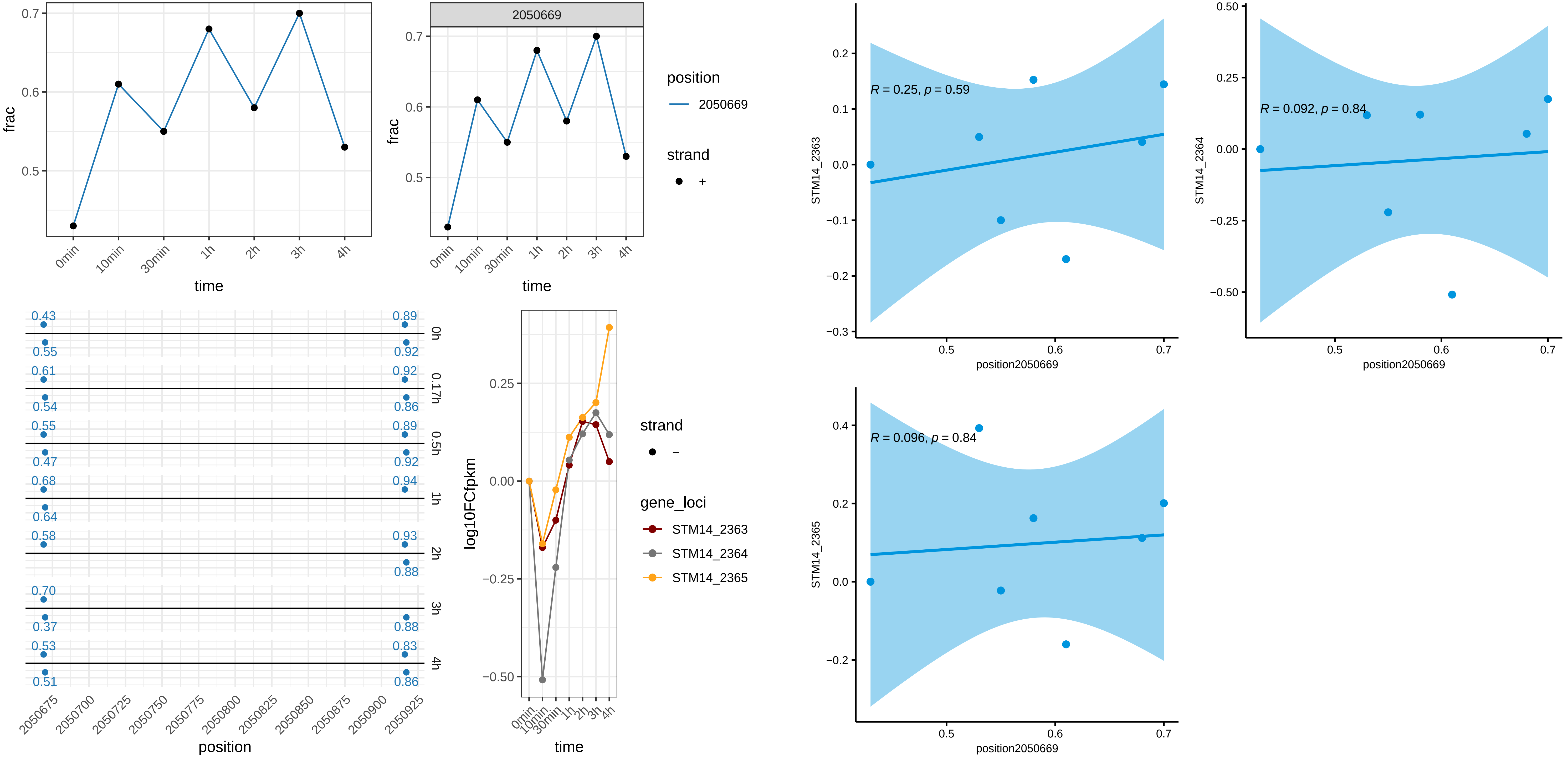

frac
time
0min
10min
30min
1h
2h
3h
4h
2050669
position
strand
+
position
log10FCfpkm
–
gene_loci
STM14_2363
STM14_2364
STM14_2365
0h
0.17h
0.5h
R = 0.25, p = 0.59
R = 0.092, p = 0.84
R = 0.096, p = 0.84
position2050669

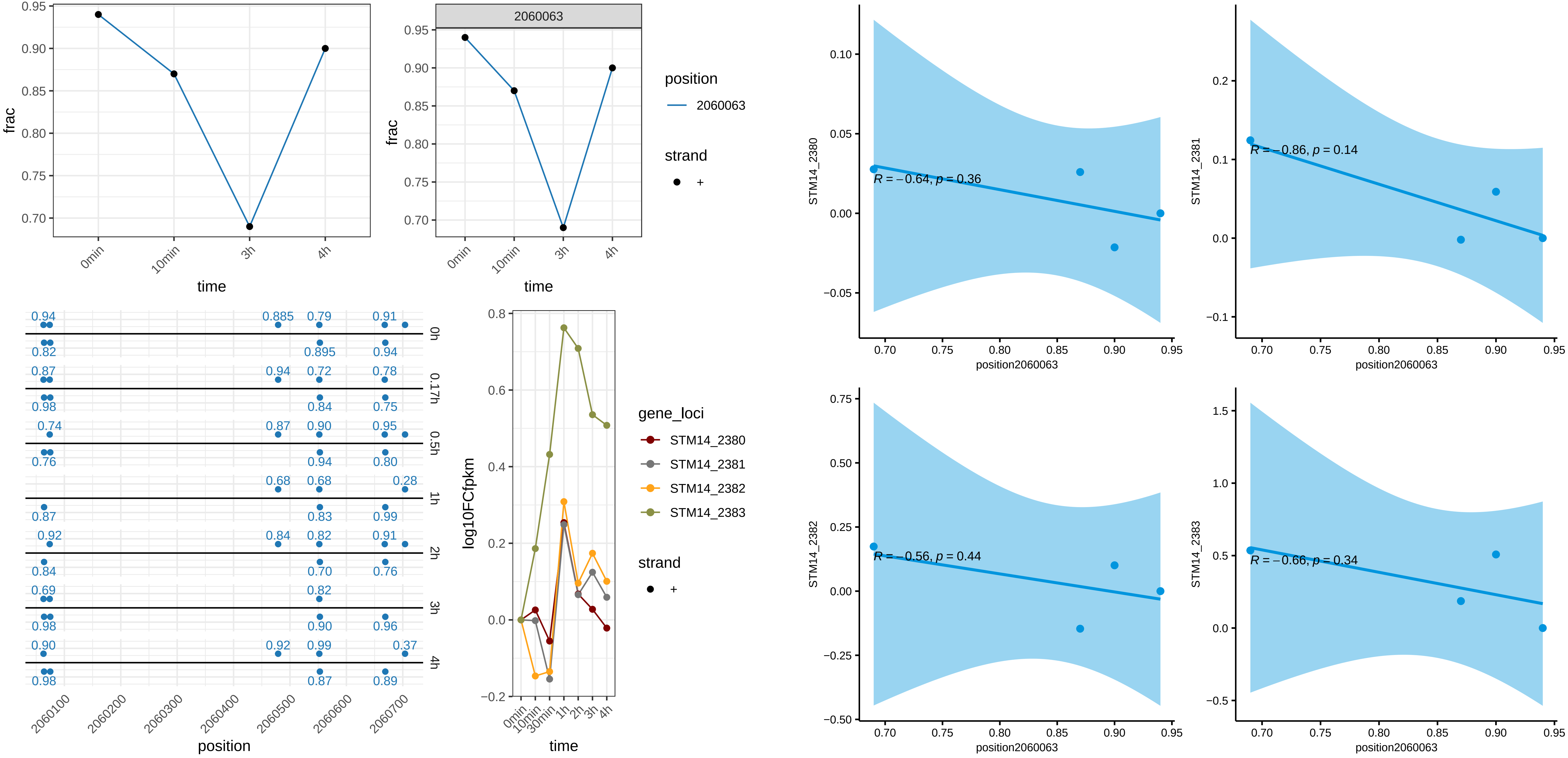

frac
time
0min
10min
3h
4h
2060063
position
2060063
strand
+
0h
0.17h
0.5h
1h
2h
3h
4h
position
log10FCfpkm
30min
gene_loci
STM14_2380
STM14_2381
STM14_2382
STM14_2383
STM14_2380
STM14_2381
STM14_2382
STM14_2383
position2060063
R = −0.64, p = 0.36
R = −0.86, p = 0.14
R = −0.56, p = 0.44
R = −0.66, p = 0.34

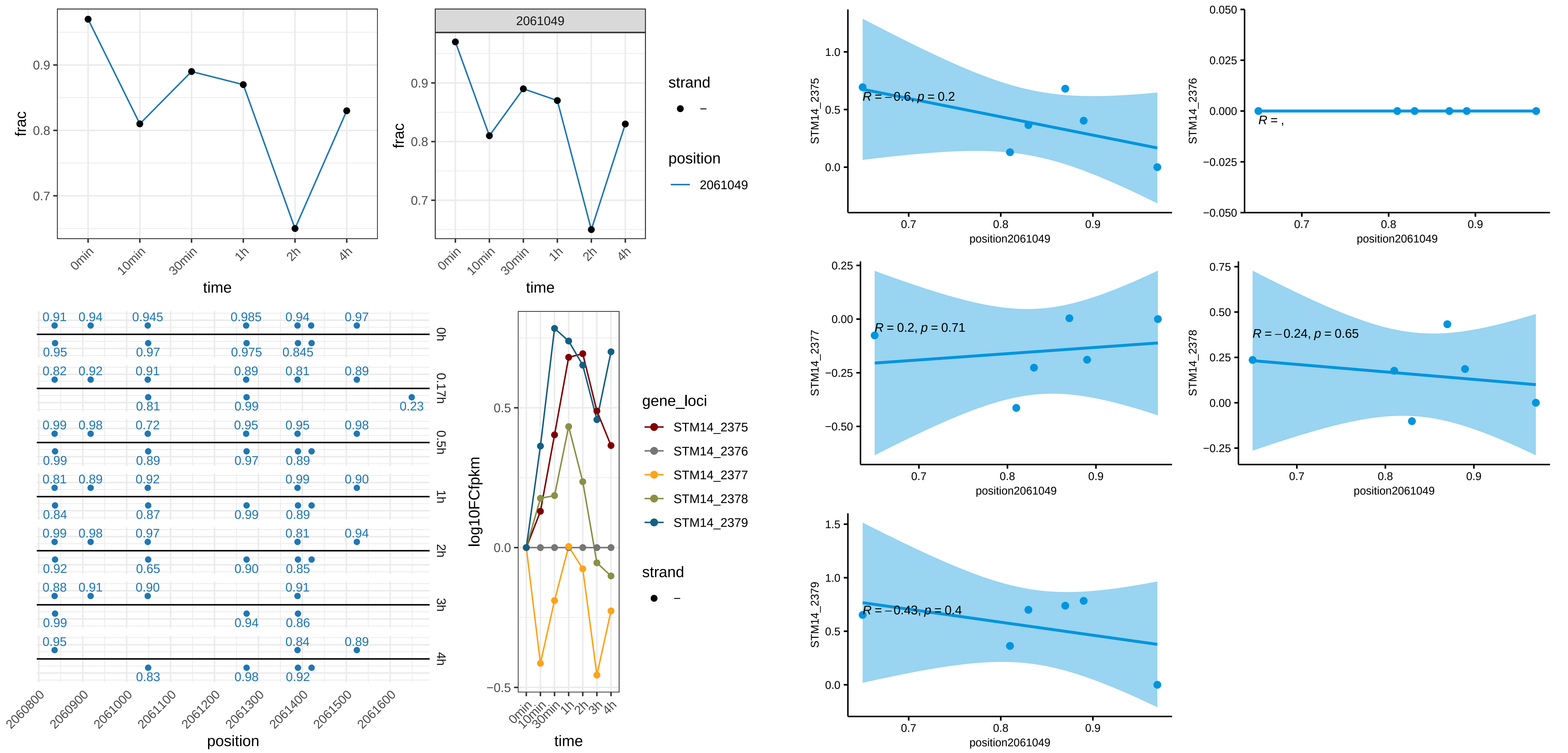

frac
time
2061049
strand
position
gene_loci
STM14_2375
STM14_2376
STM14_2377
STM14_2378
STM14_2379
log10FCfpkm
position2061049
R = −0.6, p = 0.2
R = ,
R = 0.2, p = 0.71
R = −0.24, p = 0.65
R = −0.43, p = 0.4

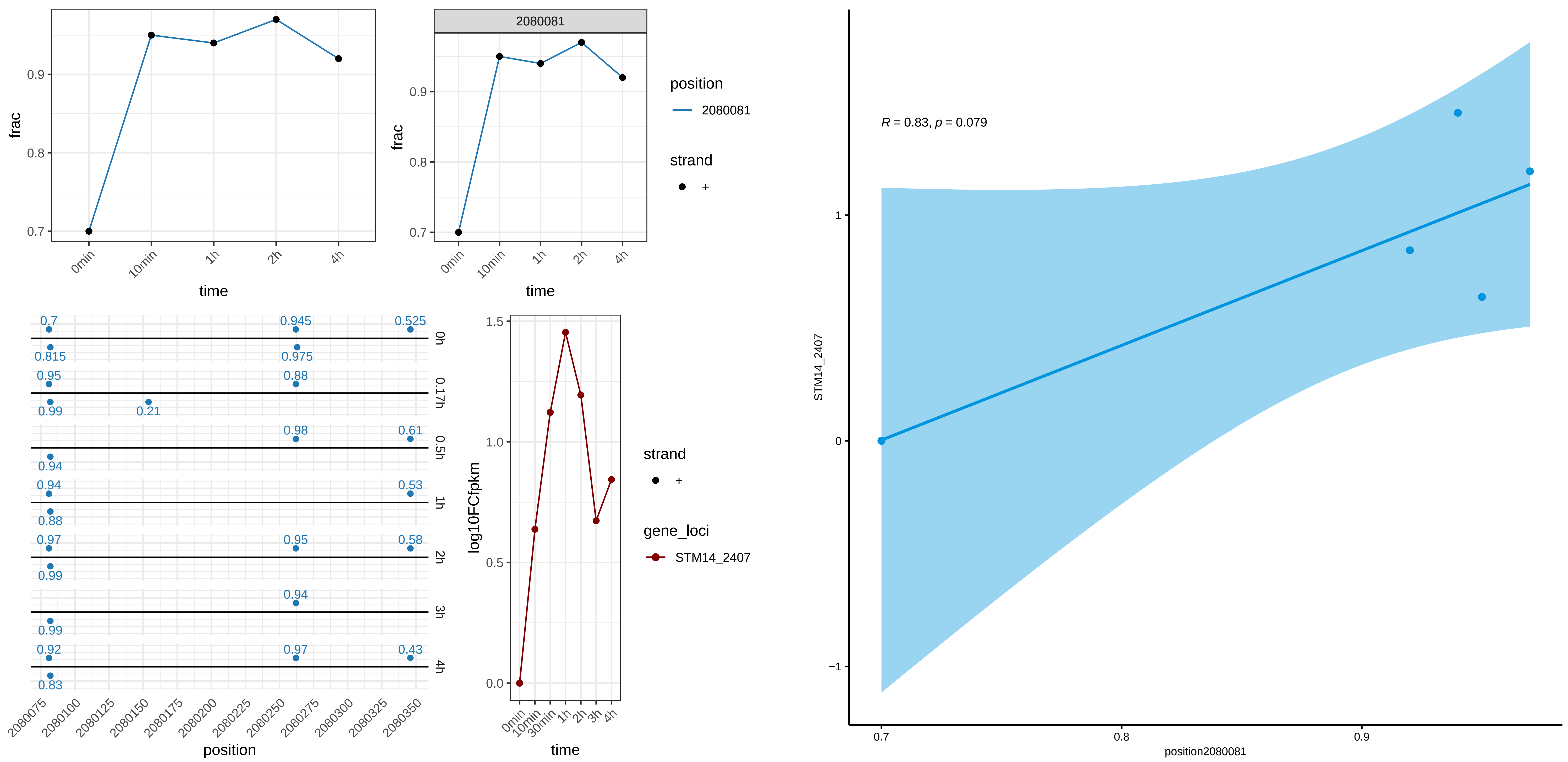
frac
time
0min
10min
1h
2h
4h
2080081
position
strand
+
log10FCfpkm
gene_loci
STM14_2407
0h
0.17h
0.5h
3h
position
R = 0.83, p = 0.079
STM14_2407
position2080081

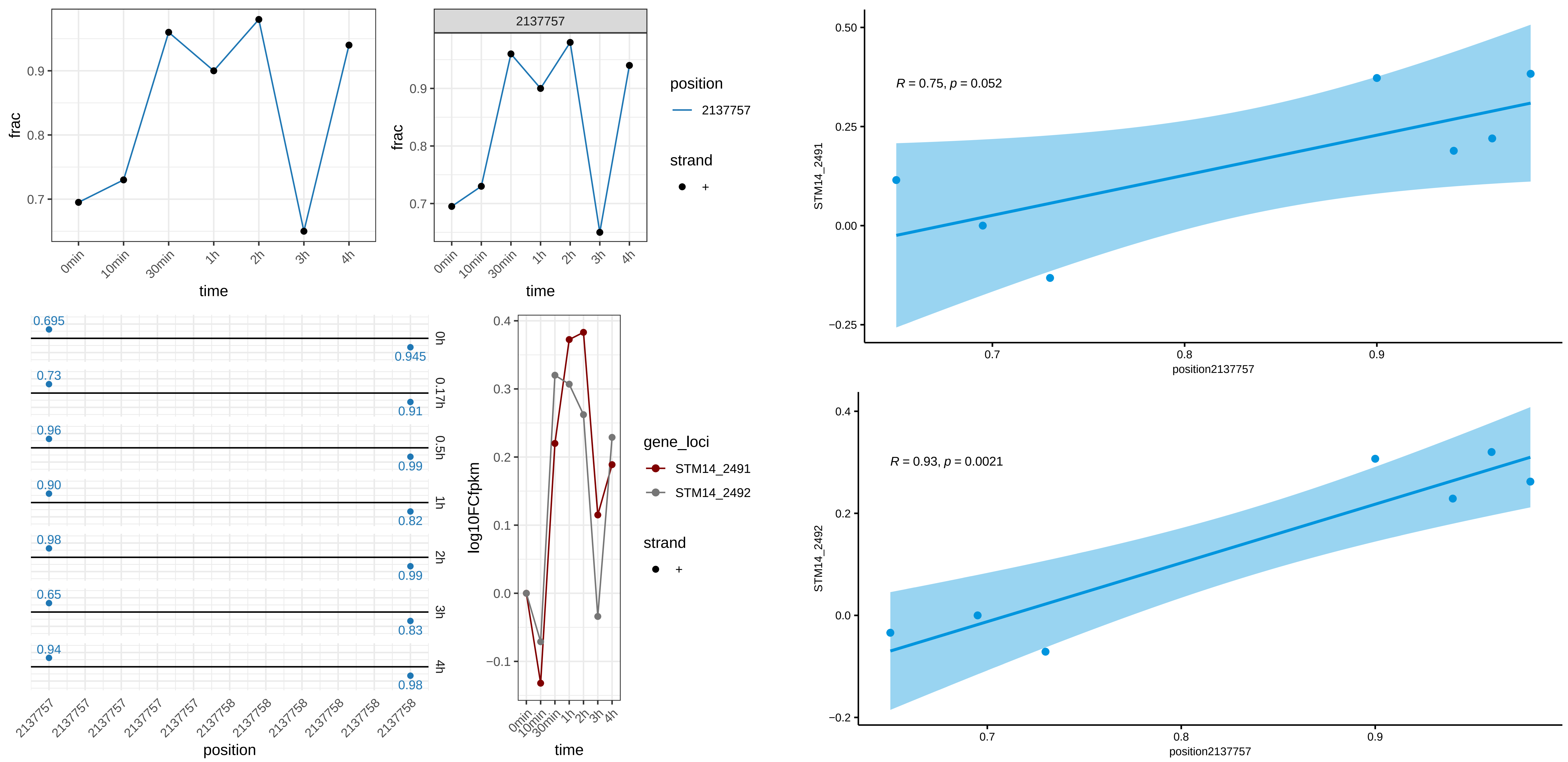

frac
time
0min
10min
30min
1h
2h
3h
4h
2137757
position
strand
+
0.695
0.945
0.73
0.91
0.96
0.99
0.90
0.82
0.98
0.99
0.65
0.83
0.94
0.98
0h
0.17h
0.5h
position
log10FCfpkm
gene_loci
STM14_2491
STM14_2492
R = 0.75, p = 0.052
STM14_2491
position2137757
R = 0.93, p = 0.0021
STM14_2492
position2137757

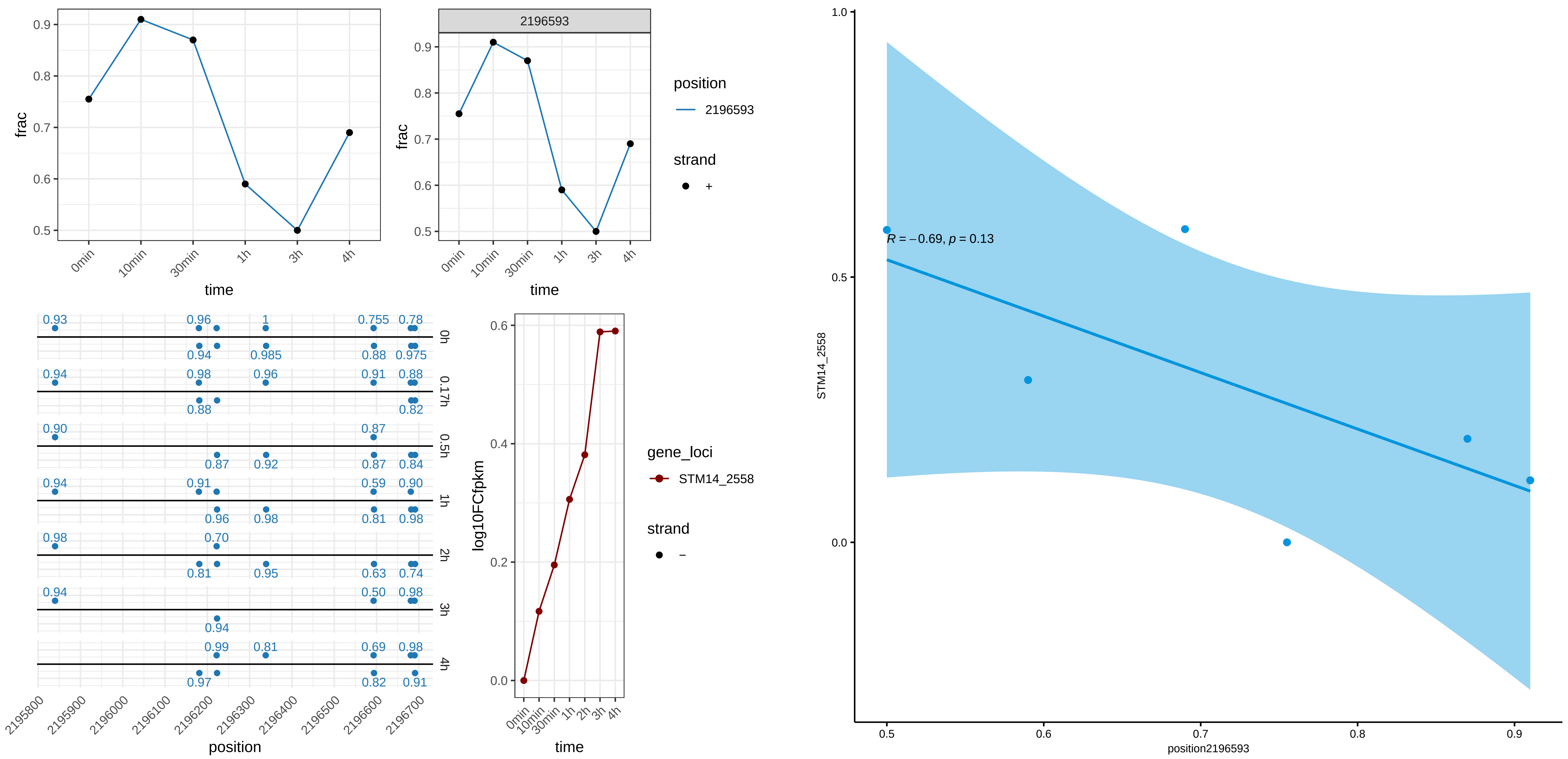

frac
time
0min
10min
30min
1h
3h
4h
2196593
position
2196593
strand
+
position
0h
0.17h
0.5h
1h
2h
3h
4h
log10FCfpkm
gene_loci
STM14_2558
strand
–
R = –0.69, p = 0.13
STM14_2558
position2196593

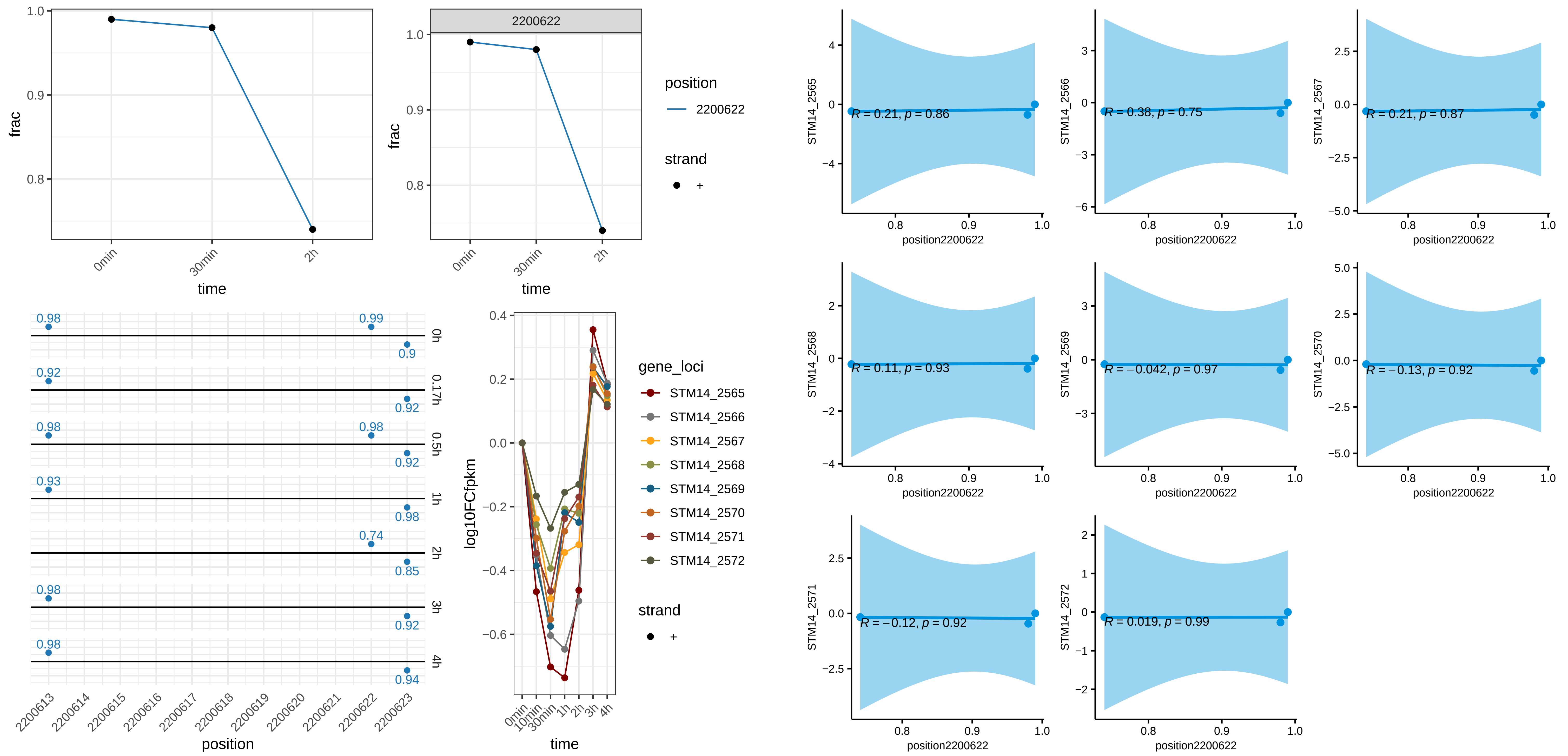

frac
time
0min
30min
2h
2200622
position
2200622
strand
+
0.98
0.99
0.9
0.92
0.92
0.98
0.98
0.92
0.93
0.98
0.74
0.85
0.98
0.92
0.98
0.94
0h
0.17h
0.5h
1h
2h
3h
4h
position
log10FCfpkm
gene_loci
STM14_2565
STM14_2566
STM14_2567
STM14_2568
STM14_2569
STM14_2570
STM14_2571
STM14_2572
R = 0.21, p = 0.86
R = 0.38, p = 0.75
R = 0.21, p = 0.87
R = 0.11, p = 0.93
R = −0.042, p = 0.97
R = −0.13, p = 0.92
R = −0.12, p = 0.92
R = 0.019, p = 0.99
position2200622

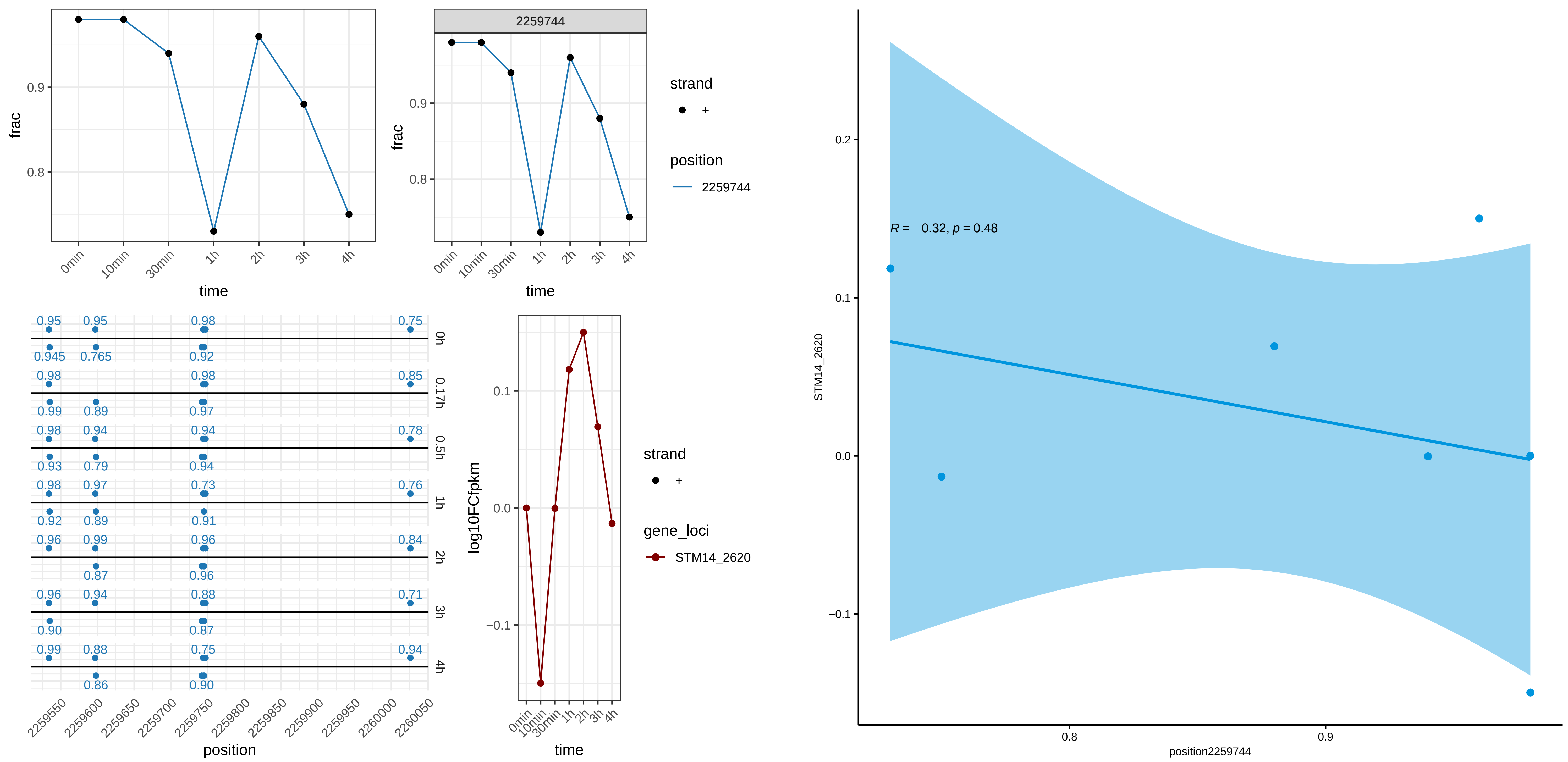

frac
time
0min
10min
30min
1h
2h
3h
4h
2259744
strand
+
position
2259744
log10FCfpkm
gene_loci
STM14_2620
position
0h
0.17h
0.5h
1h
2h
3h
4h
R = −0.32, p = 0.48
STM14_2620
position2259744

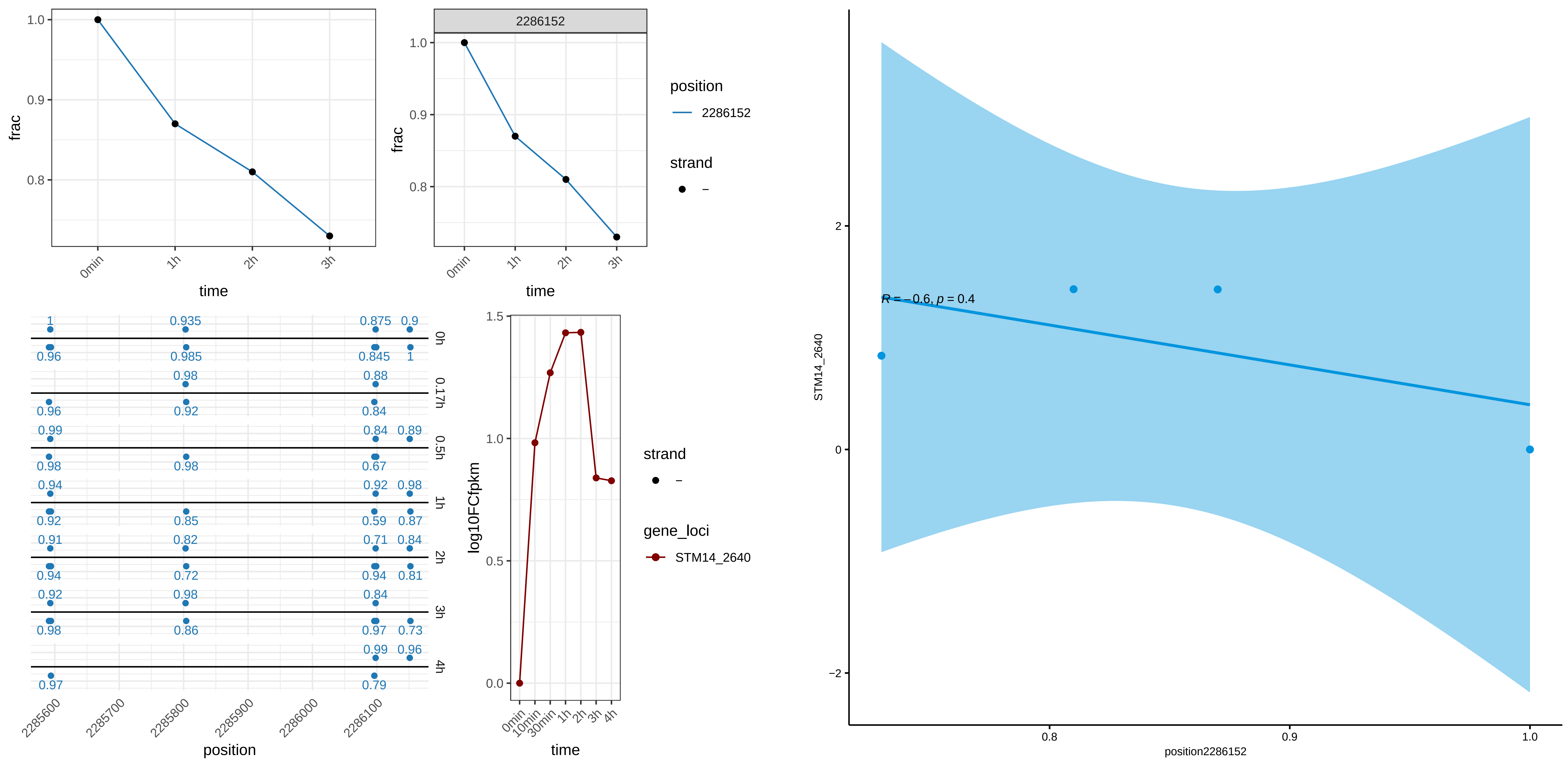

frac
time
0min
1h
2h
3h
2286152
position
2286152
strand
–
0.96
0.985
0.845
1
0.935
0.875
0.9
0h
0.17h
0.5h
1h
2h
3h
4h
position
log10FCfpkm
gene_loci
STM14_2640
R = −0.6, p = 0.4
STM14_2640
position2286152

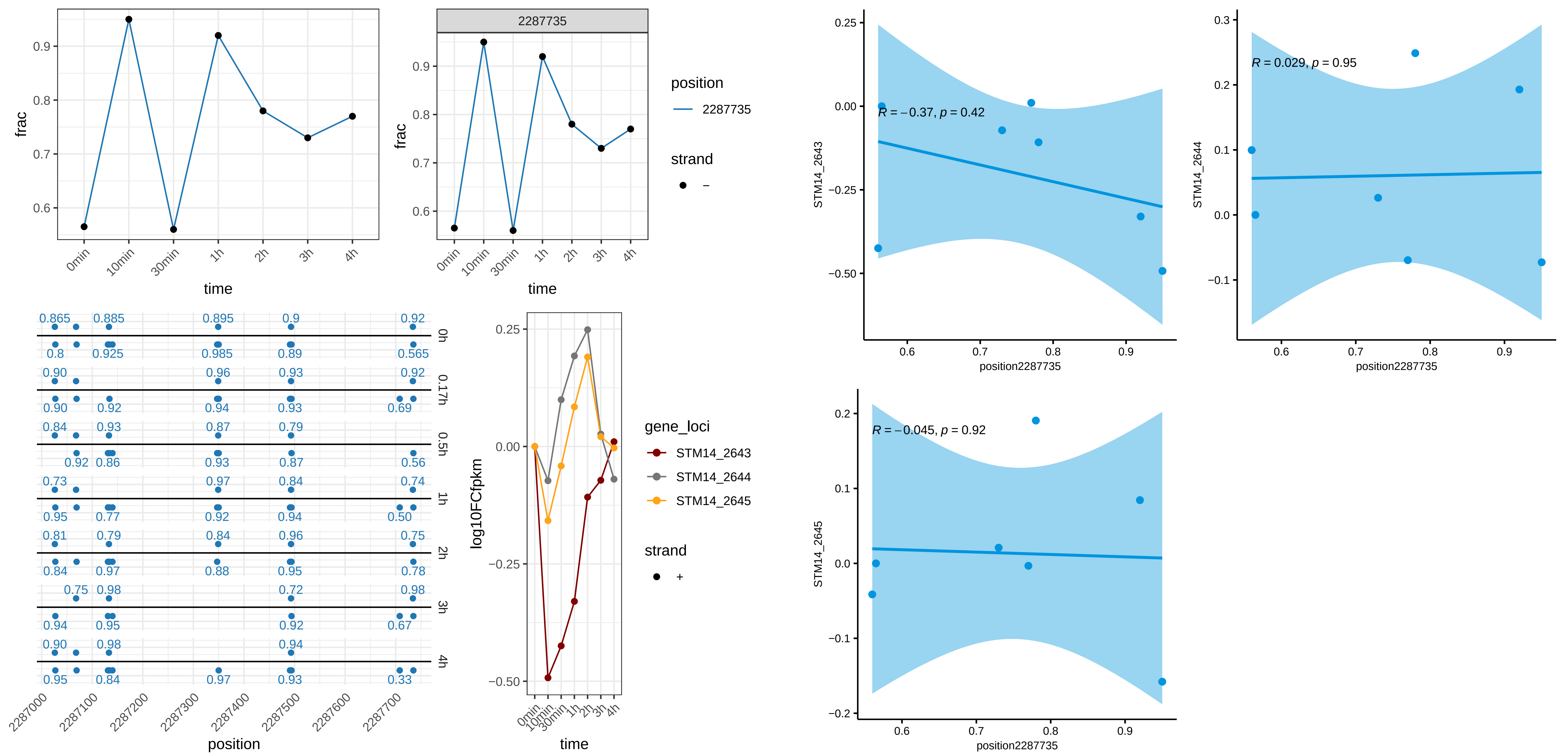

frac
time
2287735
position
2287735
strand
–
position
log10FCfpkm
gene_loci
STM14_2643
STM14_2644
STM14_2645
+
R = −0.37, p = 0.42
STM14_2643
position2287735
R = 0.029, p = 0.95
STM14_2644
position2287735
R = −0.045, p = 0.92
STM14_2645
position2287735

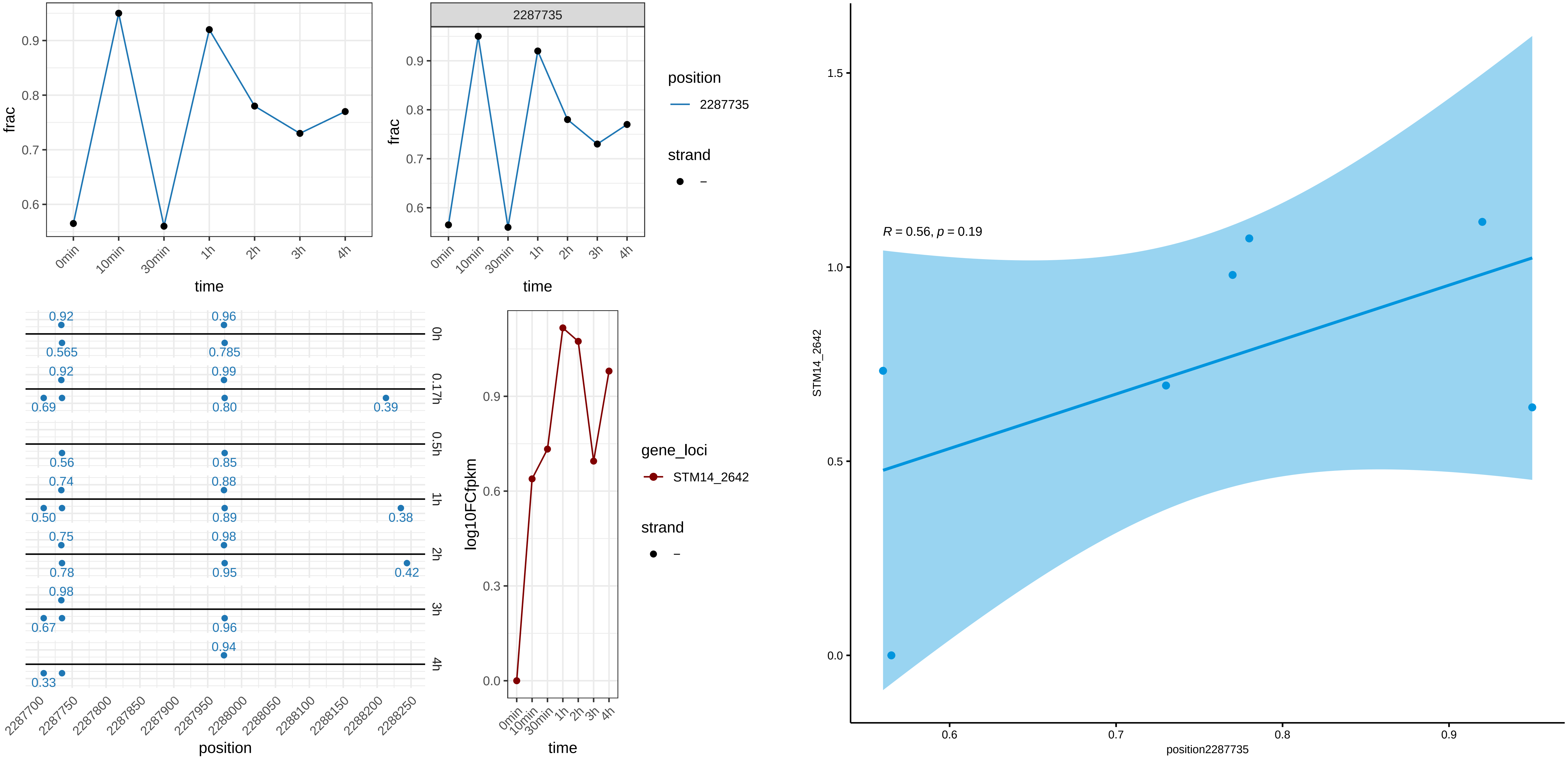

frac
time
2287735
position
2287735
strand
–
0.92
0.96
0.565
0.785
0.99
0.69
0.80
0.39
0.56
0.85
0.74
0.88
0.50
0.89
0.38
0.75
0.98
0.78
0.95
0.42
0.67
0.94
0.33
0h
0.17h
0.5h
1h
2h
3h
4h
position
log10FCfpkm
gene_loci
STM14_2642
R = 0.56, p = 0.19
STM14_2642
position2287735

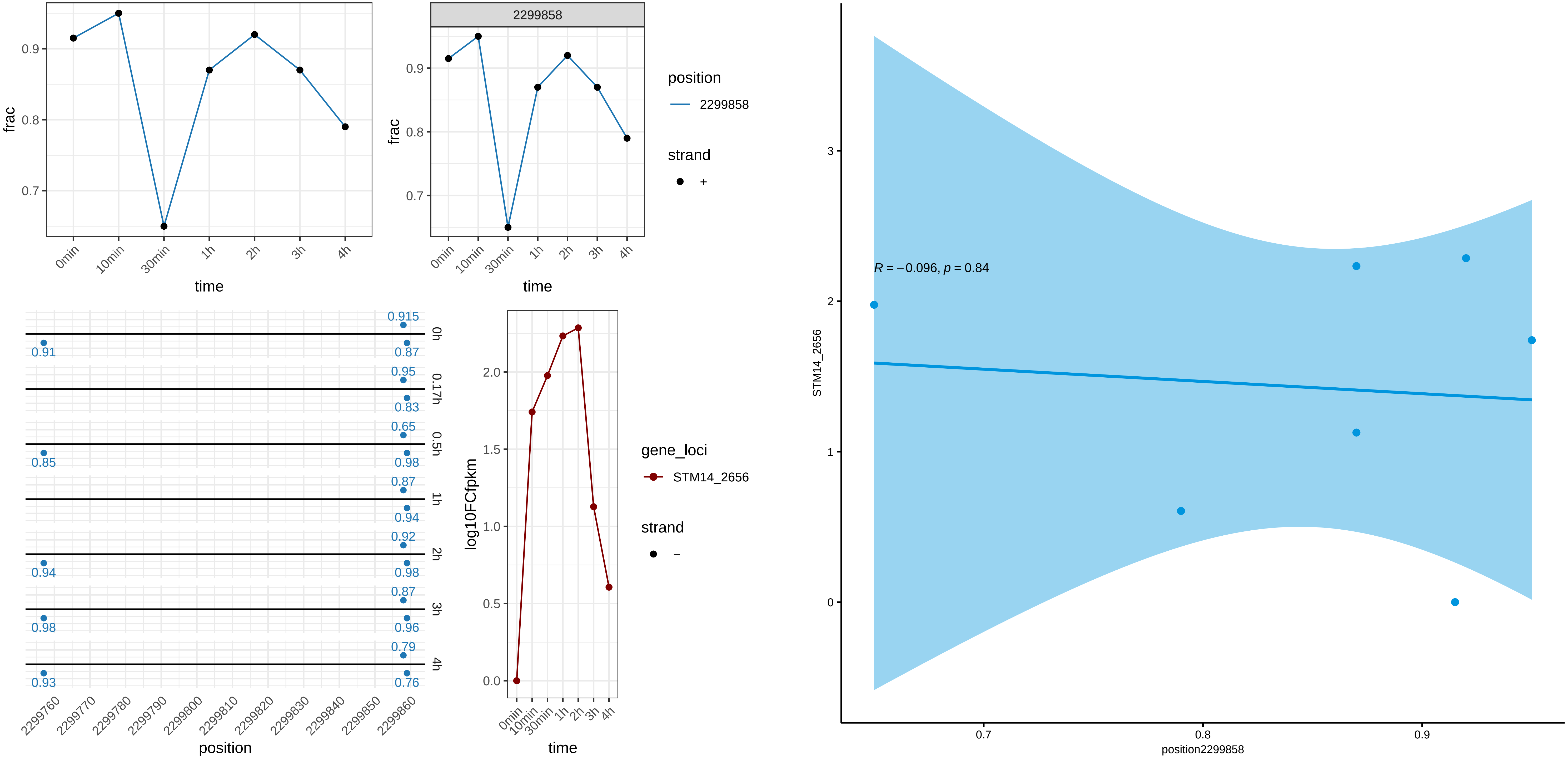

frac
0.9
0.8
0.7
0min
10min
30min
1h
2h
3h
4h
time
2299858
position
2299858
strand
+
0.915
0.91
0.87
0.95
0.83
0.65
0.85
0.98
0.87
0.94
0.92
0.94
0.98
0.87
0.98
0.96
0.79
0.93
0.76
0h
0.17h
0.5h
1h
2h
3h
4h
2299760
2299770
2299780
2299790
2299800
2299810
2299820
2299830
2299840
2299850
2299860
position
log10FCfpkm
2.0
1.5
1.0
0.5
0.0
gene_loci
STM14_2656
strand
–
R = –0.096, p = 0.84
STM14_2656
3
2
1
0
0.7
0.8
0.9
position2299858

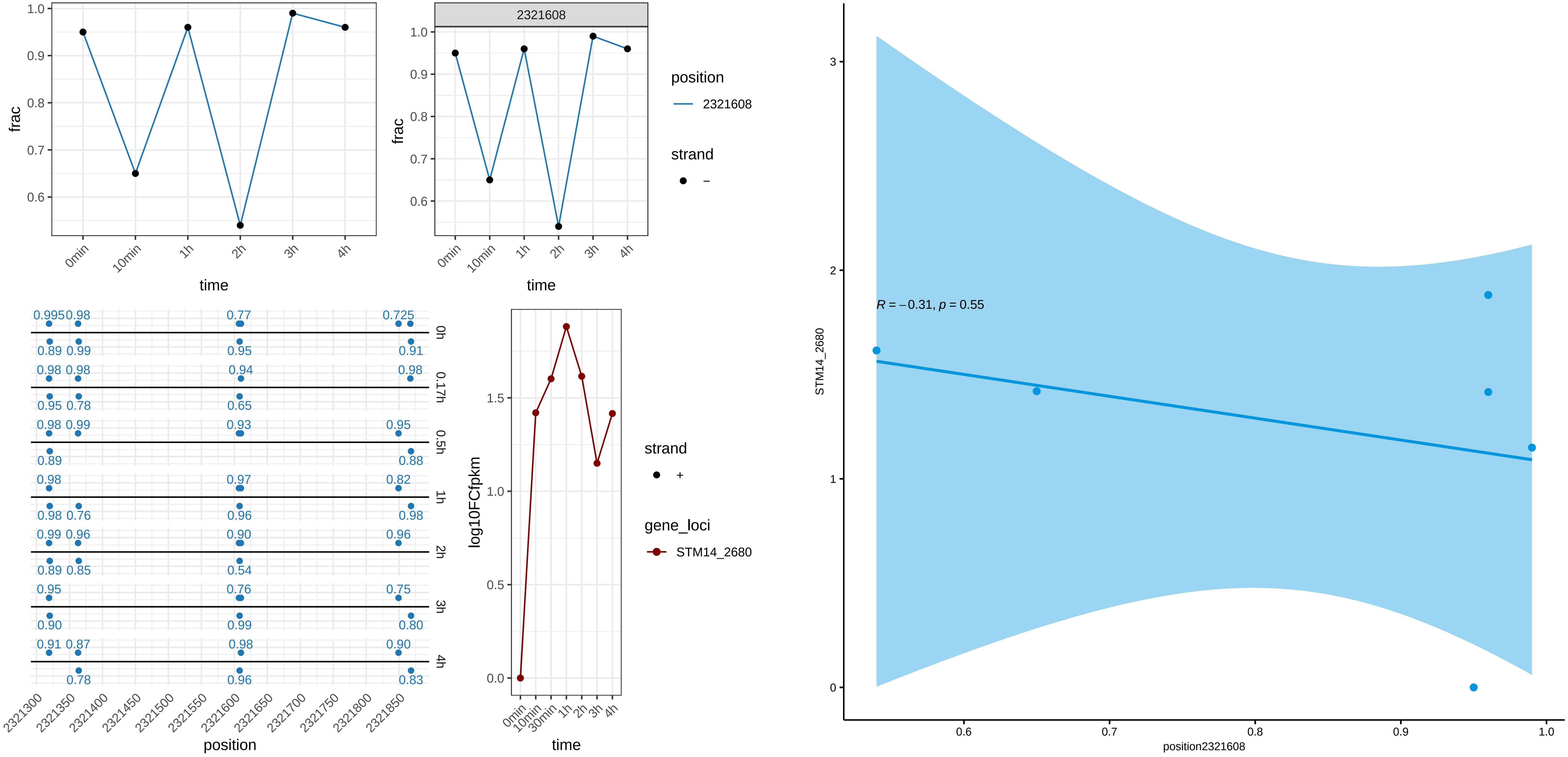

frac
time
0min
10min
1h
2h
3h
4h
2321608
position
2321608
strand
–
0.995 0.98
0.77
0.725
0h
0.89 0.99
0.95
0.91
0.98 0.98
0.94
0.98
0.17h
0.95 0.78
0.65
0.98 0.99
0.93
0.95
0.5h
0.89
0.88
0.98
0.97
0.82
1h
0.98 0.76
0.96
0.98
0.99 0.96
0.90
0.96
2h
0.89 0.85
0.54
0.95
0.76
0.75
3h
0.90
0.99
0.80
0.91 0.87
0.98
0.90
4h
0.78
0.96
0.83
position
log10FCfpkm
30min
strand
+
gene_loci
STM14_2680
R = −0.31, p = 0.55
STM14_2680
position2321608

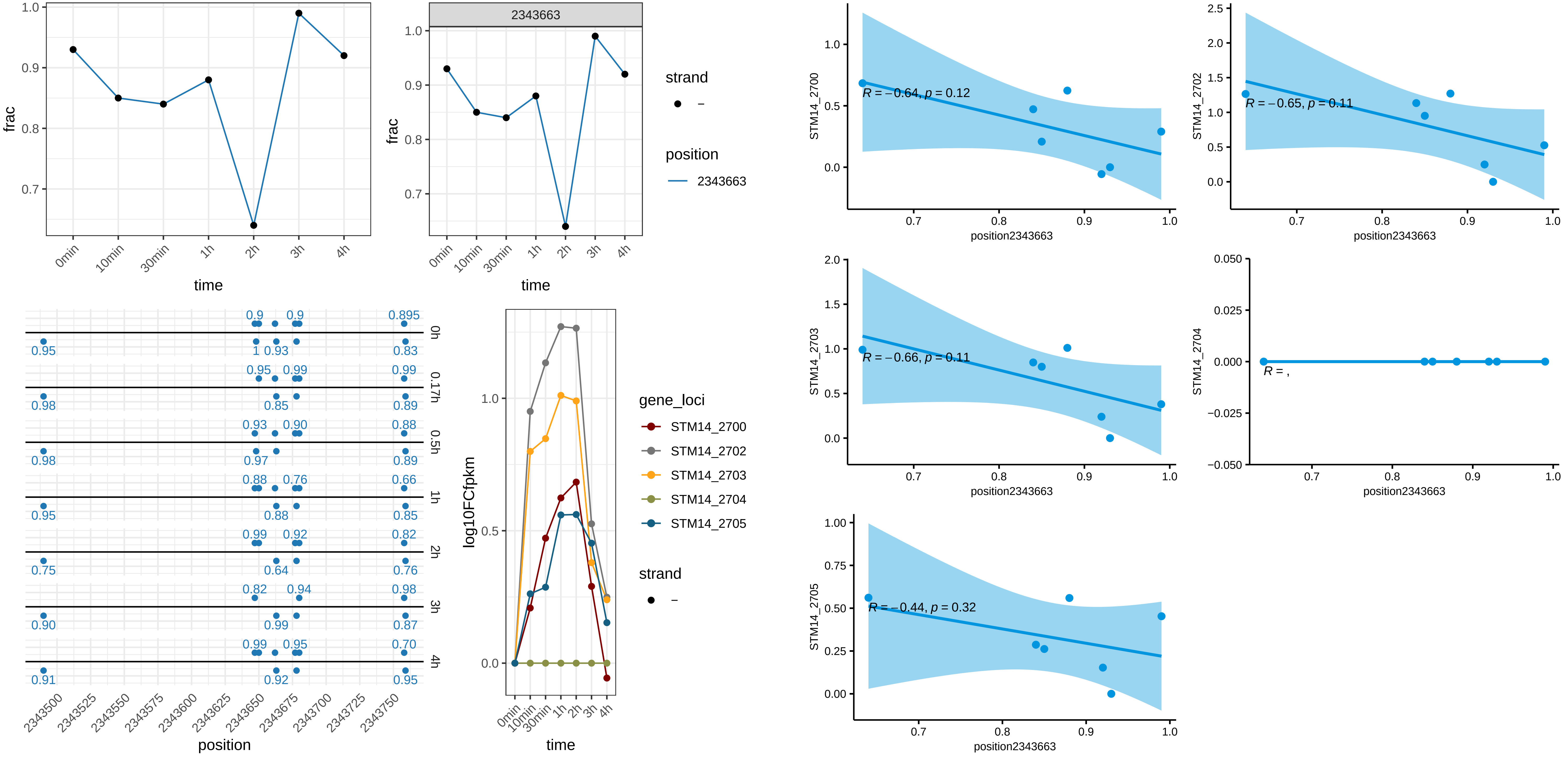

frac
time
0min
10min
30min
1h
2h
3h
4h
2343663
strand
–
position
2343663
log10FCfpkm
gene_loci
STM14_2700
STM14_2702
STM14_2703
STM14_2704
STM14_2705
position
0h
0.17h
0.5h
1h
2h
3h
4h
STM14_2700
R = −0.64, p = 0.12
position2343663
STM14_2702
R = −0.65, p = 0.11
position2343663
STM14_2703
R = −0.66, p = 0.11
position2343663
STM14_2704
R = ,
position2343663
STM14_2705
R = −0.44, p = 0.32
position2343663

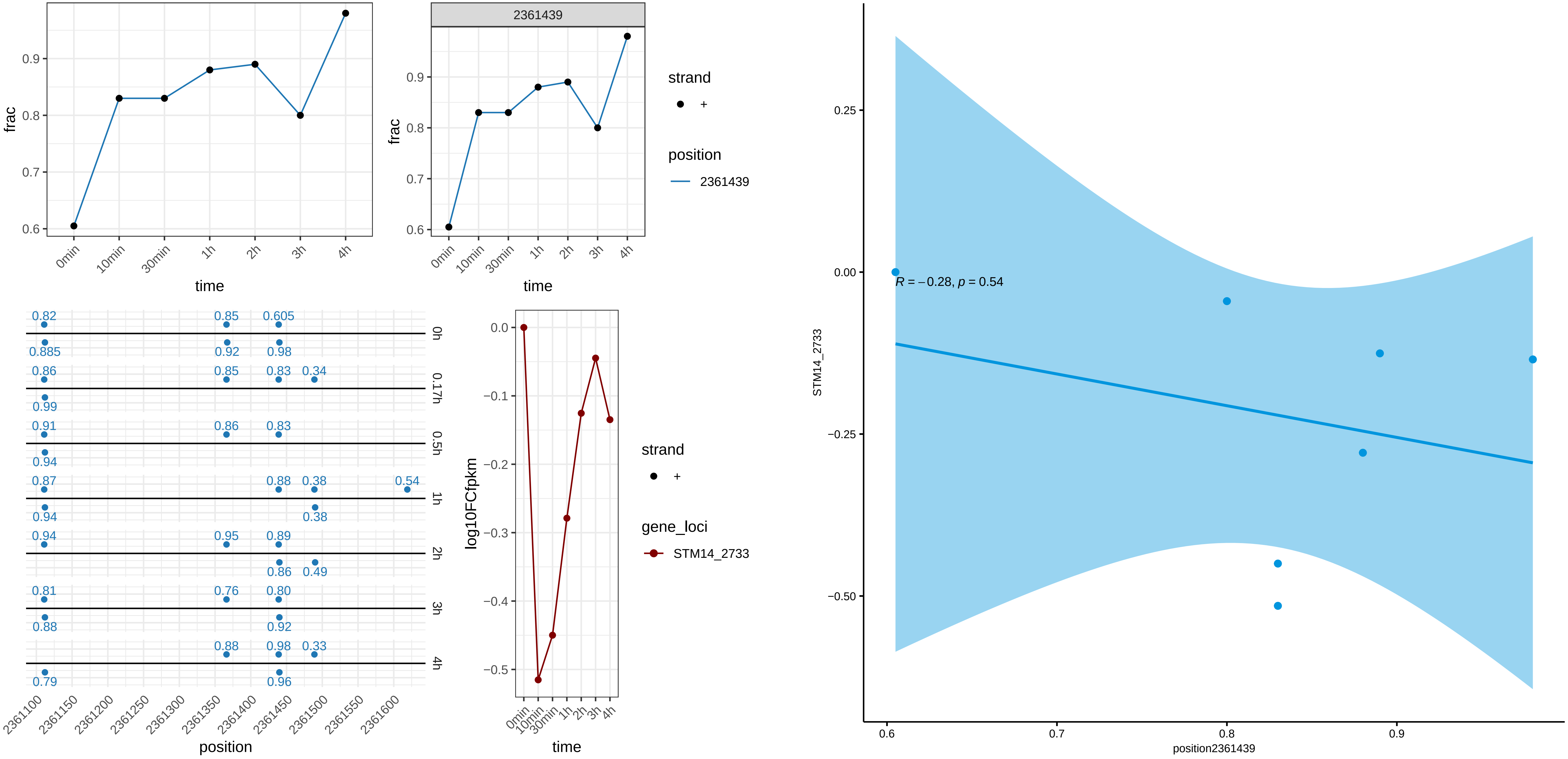
frac
time
0min
10min
30min
1h
2h
3h
4h
2361439
strand
+
position
2361439
0.82
0.85
0.605
0.885
0.92
0.98
0.86
0.85
0.83
0.34
0.99
0.91
0.86
0.83
0.94
0.87
0.88
0.38
0.54
0.94
0.38
0.94
0.95
0.89
0.86
0.49
0.81
0.76
0.80
0.88
0.92
0.88
0.98
0.33
0.79
0.96
0h
0.17h
0.5h
1h
2h
3h
4h
position
log10FCfpkm
gene_loci
STM14_2733
R = −0.28, p = 0.54
STM14_2733
position2361439

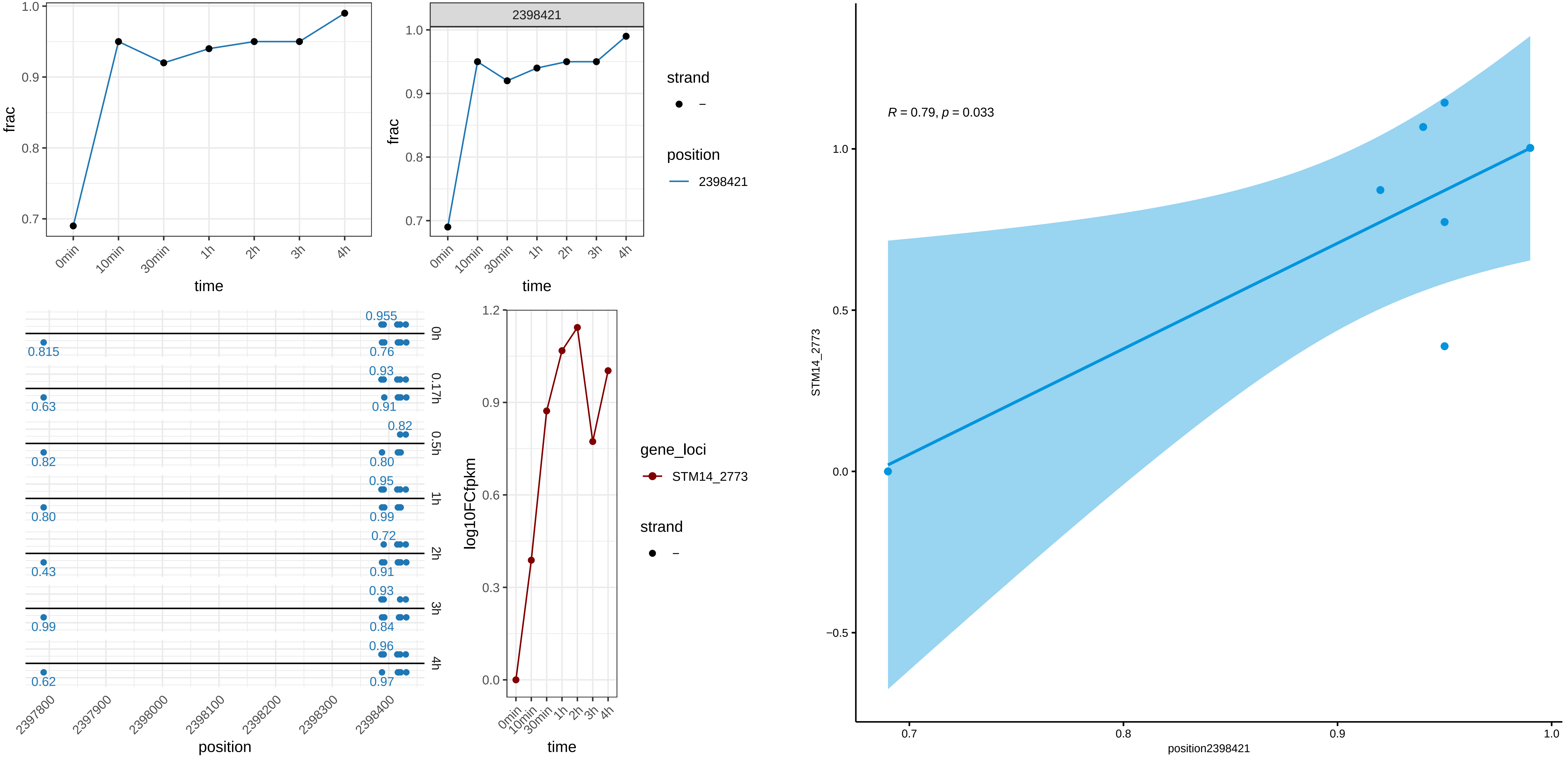

frac
time
0min
10min
30min
1h
2h
3h
4h
2398421
strand
–
position
2398421
R = 0.79, p = 0.033
STM14_2773
position2398421
0.955
0.815
0.76
0.93
0.63
0.91
0.82
0.82
0.80
0.95
0.80
0.99
0.72
0.43
0.91
0.93
0.99
0.84
0.96
0.62
0.97
0h
0.17h
0.5h
1h
2h
3h
4h
position
log10FCfpkm
gene_loci
STM14_2773
strand
–

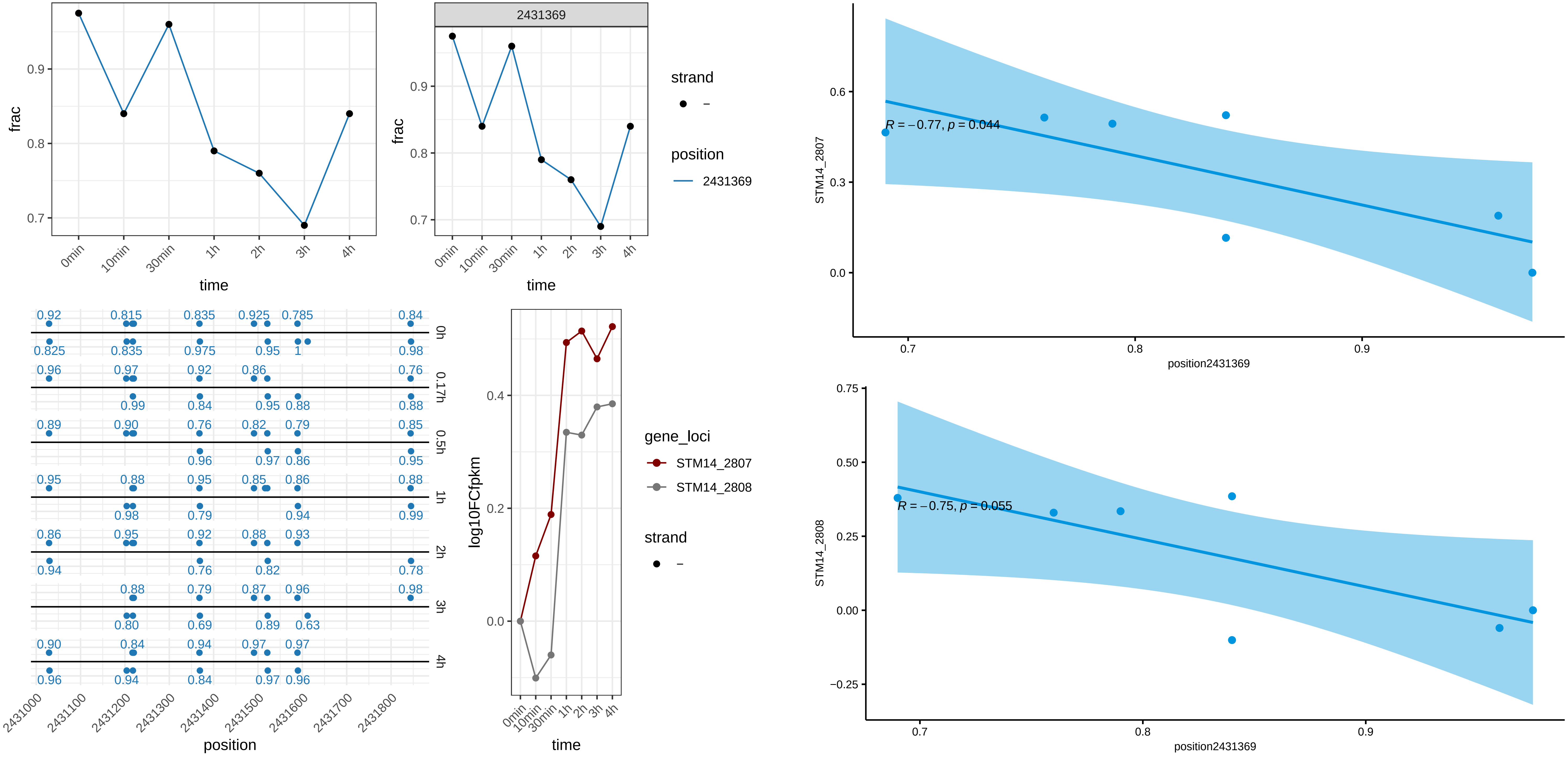

frac
time
0min
10min
30min
1h
2h
3h
4h
2431369
strand
–
position
2431369
gene_loci
STM14_2807
STM14_2808
log10FCfpkm
position
0h
0.17h
0.5h
1h
2h
3h
4h
$R = -0.77$, $p = 0.044$
STM14_2807
position2431369
$R = -0.75$, $p = 0.055$
STM14_2808
position2431369

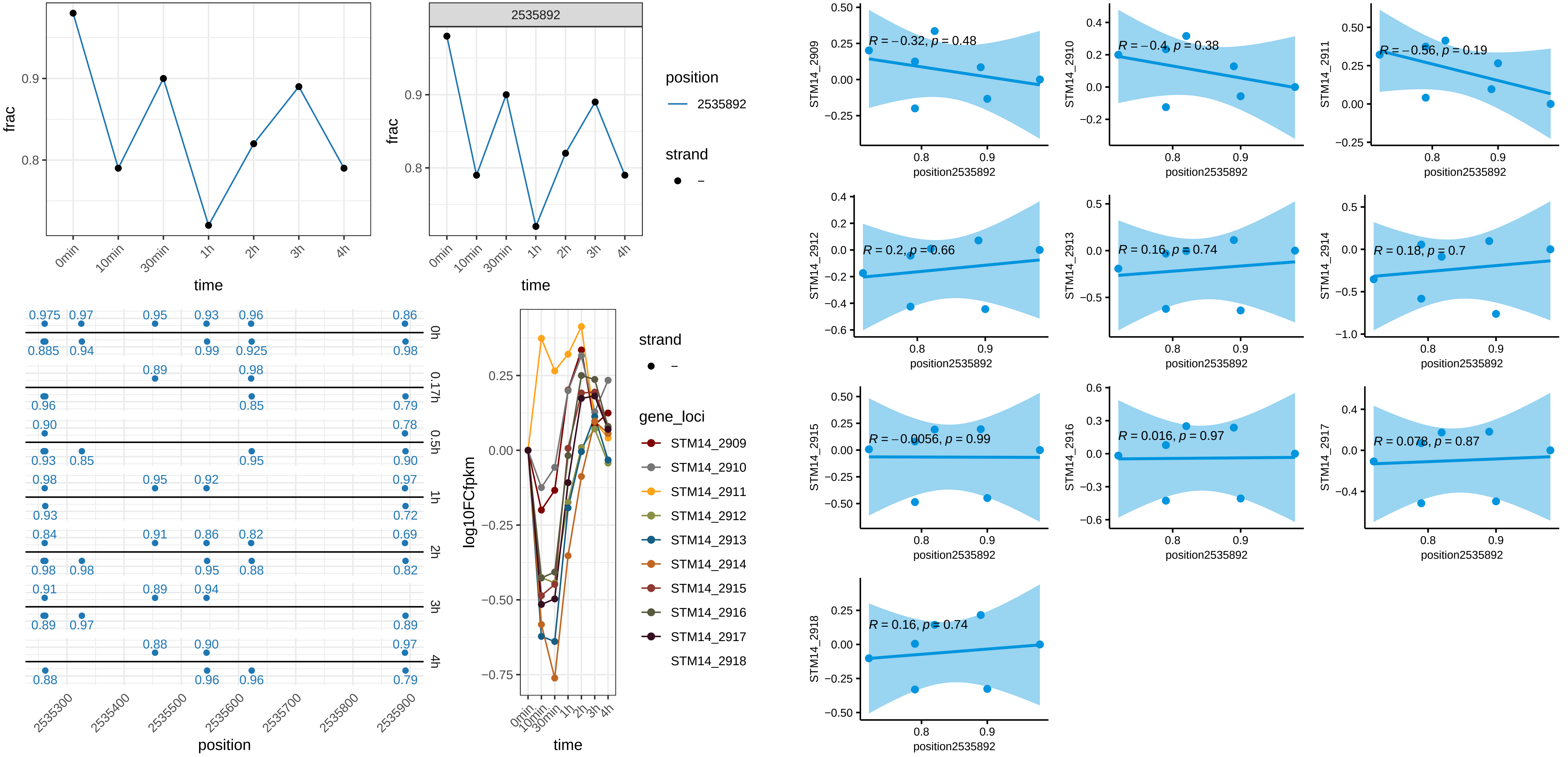

frac
time
0min 10min 30min 1h 2h 3h 4h
2535892
position
2535892
strand
–
0.975 0.97 0.95 0.93 0.96 0.86
0.885 0.94 0.99 0.925 0.98
0.89 0.98
0.96 0.85 0.79
0.90 0.78
0.93 0.85 0.95 0.90
0.98 0.95 0.92 0.97
0.93 0.72
0.84 0.91 0.86 0.82 0.69
0.98 0.98 0.95 0.88 0.82
0.91 0.89 0.94
0.89 0.97 0.89
0.88 0.90 0.97
0.88 0.96 0.96 0.79
0h 0.17h 0.5h 1h 2h 3h 4h
2535300 2535400 2535500 2535600 2535700 2535800 2535900
position
log10FCfpkm
gene_loci
STM14_2909
STM14_2910
STM14_2911
STM14_2912
STM14_2913
STM14_2914
STM14_2915
STM14_2916
STM14_2917
STM14_2918
position2535892
R = −0.32, p = 0.48
R = −0.4, p = 0.38
R = −0.56, p = 0.19
R = 0.2, p = 0.66
R = 0.16, p = 0.74
R = 0.18, p = 0.7
R = −0.0056, p = 0.99
R = 0.016, p = 0.97
R = 0.078, p = 0.87
R = 0.16, p = 0.74

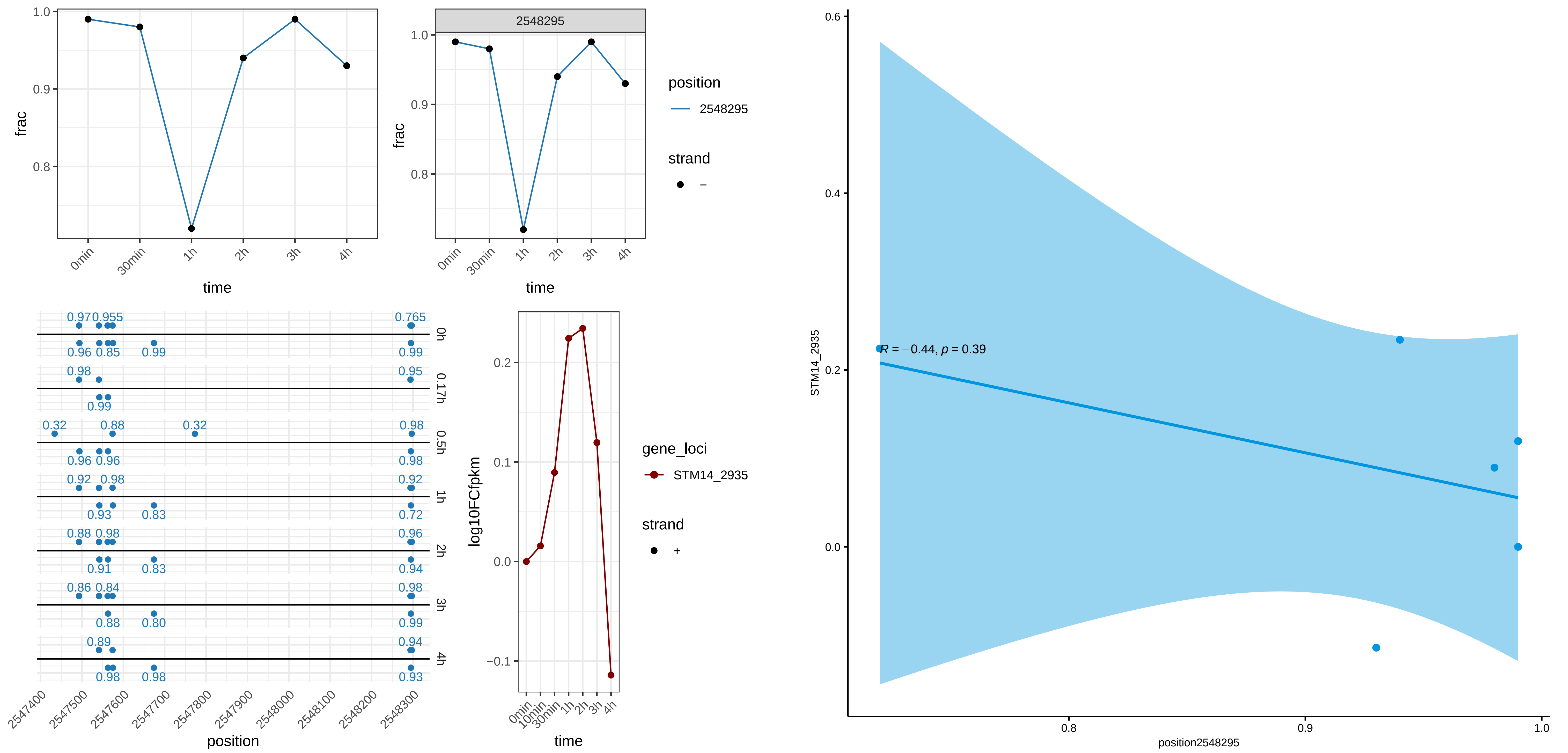
frac
time
0min
30min
1h
2h
3h
4h
2548295
position
strand
–
position
log10FCfpkm
gene_loci
STM14_2935
+
R = −0.44, p = 0.39
STM14_2935
position2548295

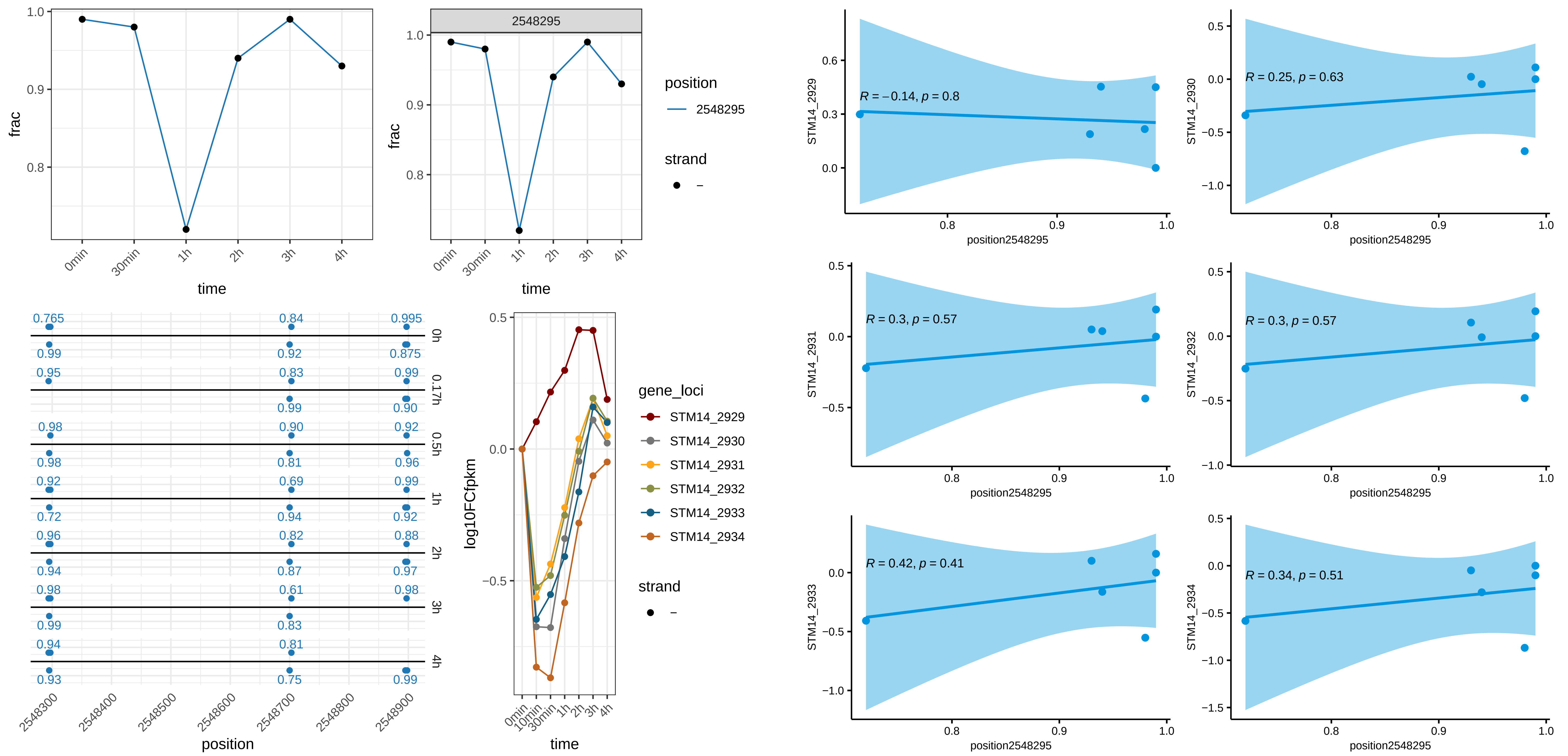

frac
time
2548295
position
strand
gene_loci
STM14_2929
STM14_2930
STM14_2931
STM14_2932
STM14_2933
STM14_2934
log10FCfpkm
R = −0.14, p = 0.8
R = 0.25, p = 0.63
R = 0.3, p = 0.57
R = 0.3, p = 0.57
R = 0.42, p = 0.41
R = 0.34, p = 0.51
position2548295

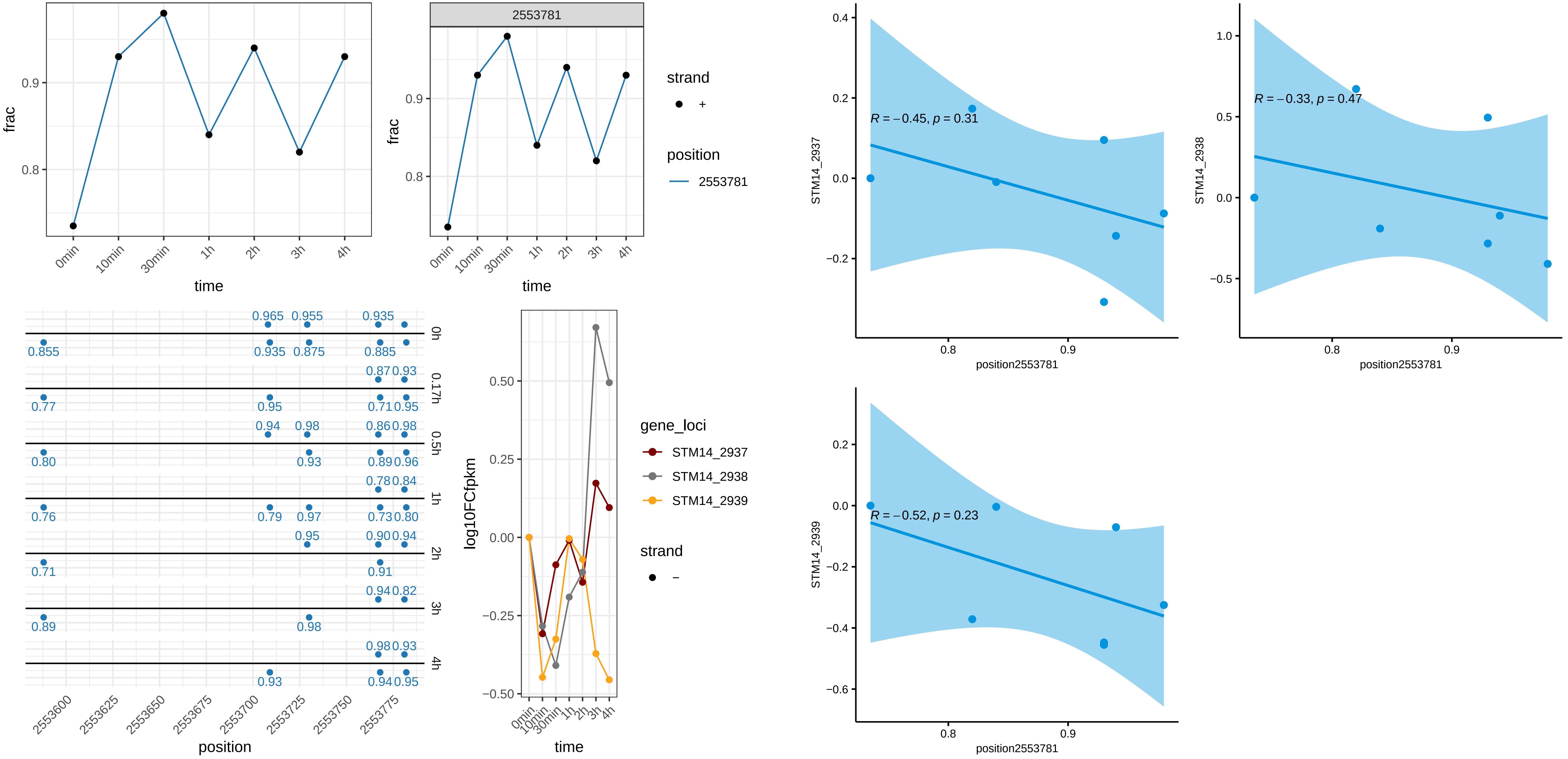

frac
time
0min
10min
30min
1h
2h
3h
4h
2553781
strand
+
position
2553781
R = −0.45, p = 0.31
STM14_2937
position2553781
R = −0.33, p = 0.47
STM14_2938
position2553781
R = −0.52, p = 0.23
STM14_2939
position2553781
0h
0.17h
0.5h
1h
2h
3h
4h
position
2553600
2553625
2553650
2553675
2553700
2553725
2553750
2553775
log10FCfpkm
gene_loci
STM14_2937
STM14_2938
STM14_2939
strand
−

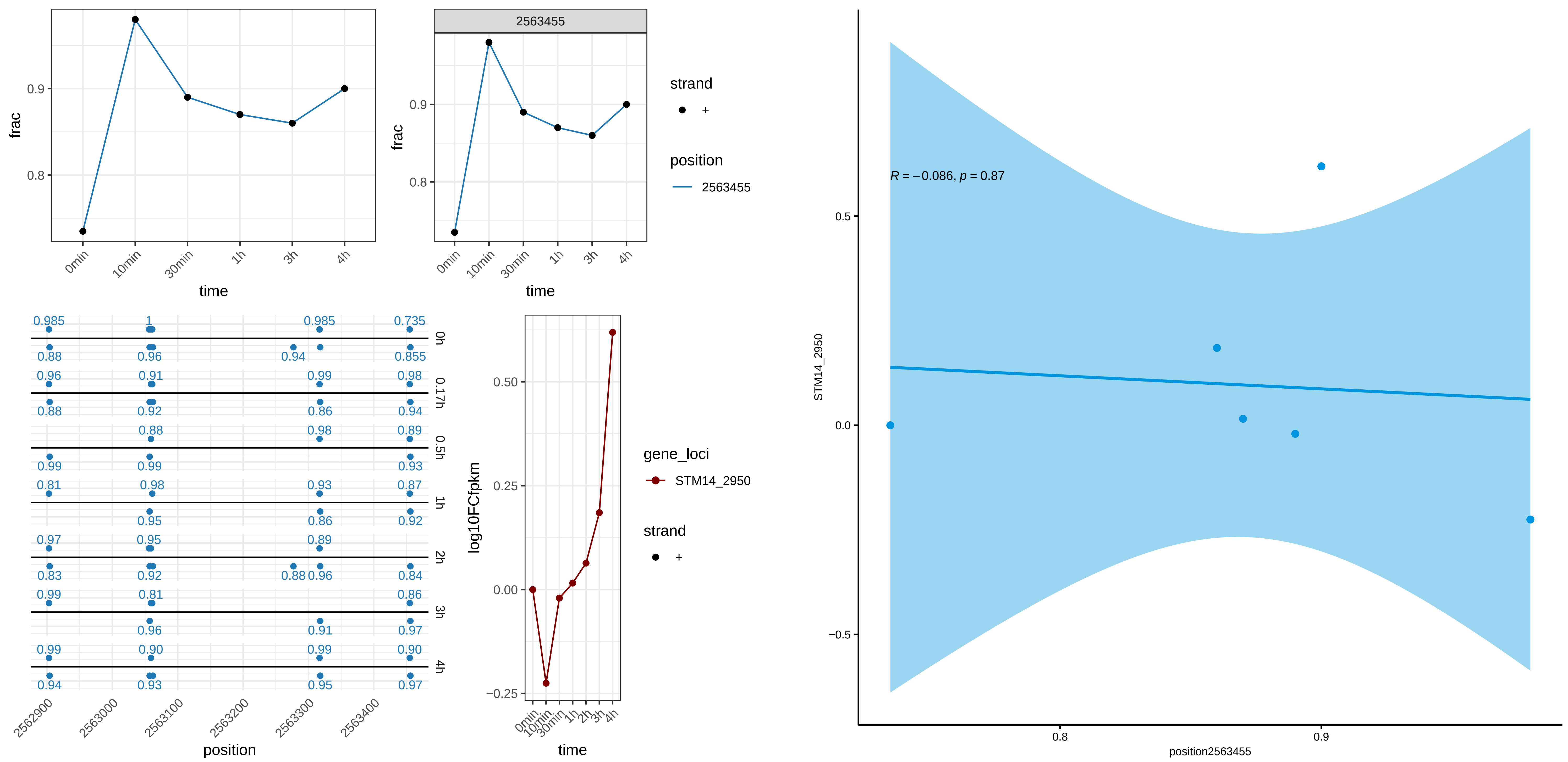

frac
time
0min
10min
30min
1h
3h
4h
2563455
strand
+
position
2563455
position
log10FCfpkm
gene_loci
STM14_2950
STM14_2950
position2563455
R = −0.086, p = 0.87

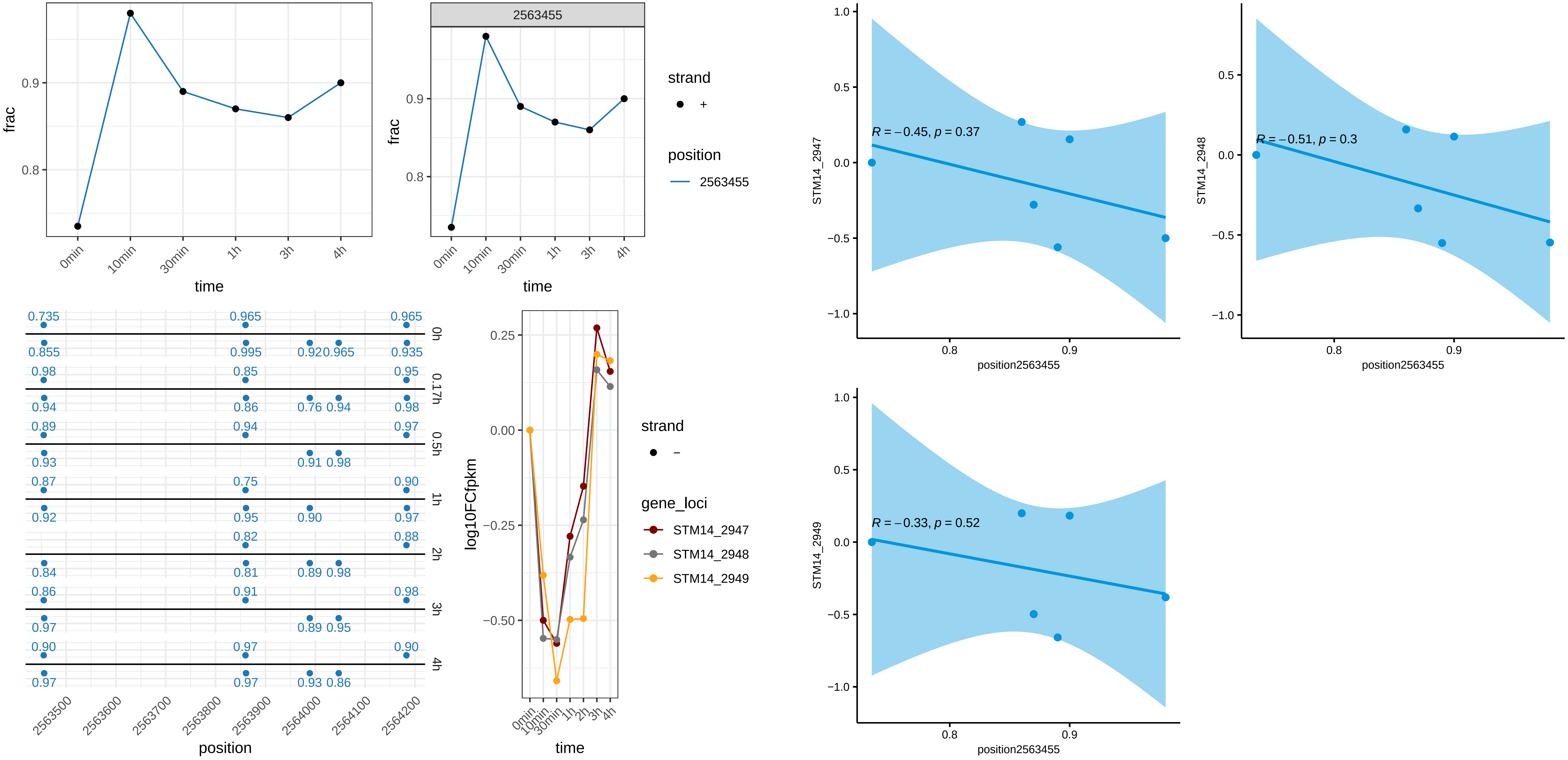

frac
time
0min
10min
30min
1h
3h
4h
2563455
strand
+
position
2563455
0.735
0.965
0.965
0h
0.855
0.995
0.920.965
0.935
0.98
0.85
0.95
0.17h
0.94
0.86
0.76 0.94
0.98
0.89
0.94
0.97
0.5h
0.93
0.91 0.98
0.87
0.75
0.90
1h
0.92
0.95
0.90
0.97
0.82
0.88
2h
0.84
0.81
0.89 0.98
0.86
0.91
0.98
3h
0.97
0.89 0.95
0.90
0.97
0.90
4h
0.97
0.97
0.93 0.86
2563500
2563600
2563700
2563800
2563900
2564000
2564100
2564200
position
log10FCfpkm
time
strand
–
gene_loci
STM14_2947
STM14_2948
STM14_2949
R = −0.45, p = 0.37
STM14_2947
position2563455
R = −0.51, p = 0.3
STM14_2948
position2563455
R = −0.33, p = 0.52
STM14_2949
position2563455

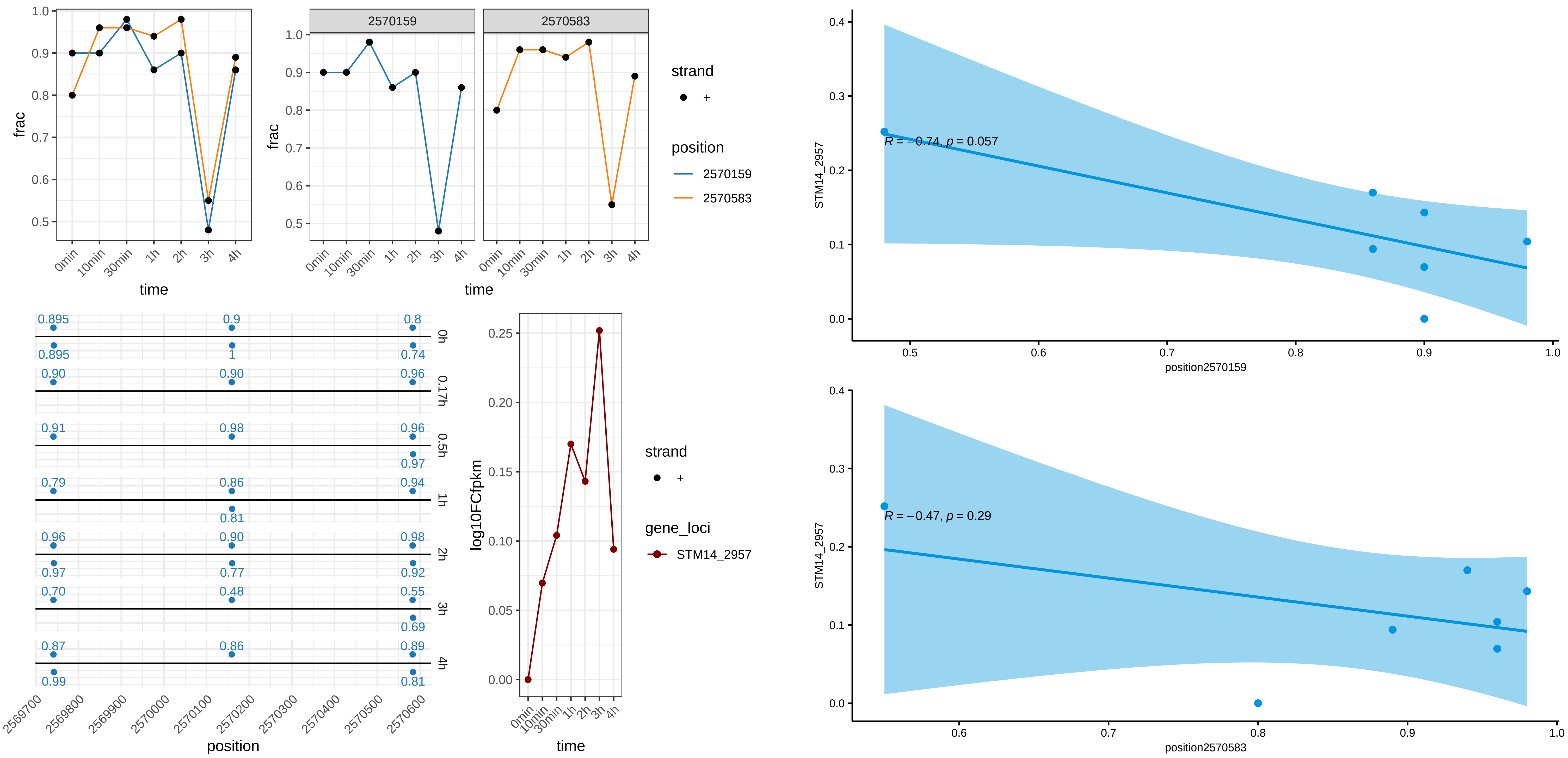

frac
time
0min
10min
30min
1h
2h
3h
4h
2570159
2570583
strand
+
position
2570159
2570583
0h
0.17h
0.5h
1h
2h
3h
4h
position
log10FCfpkm
gene_loci
STM14_2957
STM14_2957
position2570159
R = −0.74, p = 0.057
position2570583
R = −0.47, p = 0.29

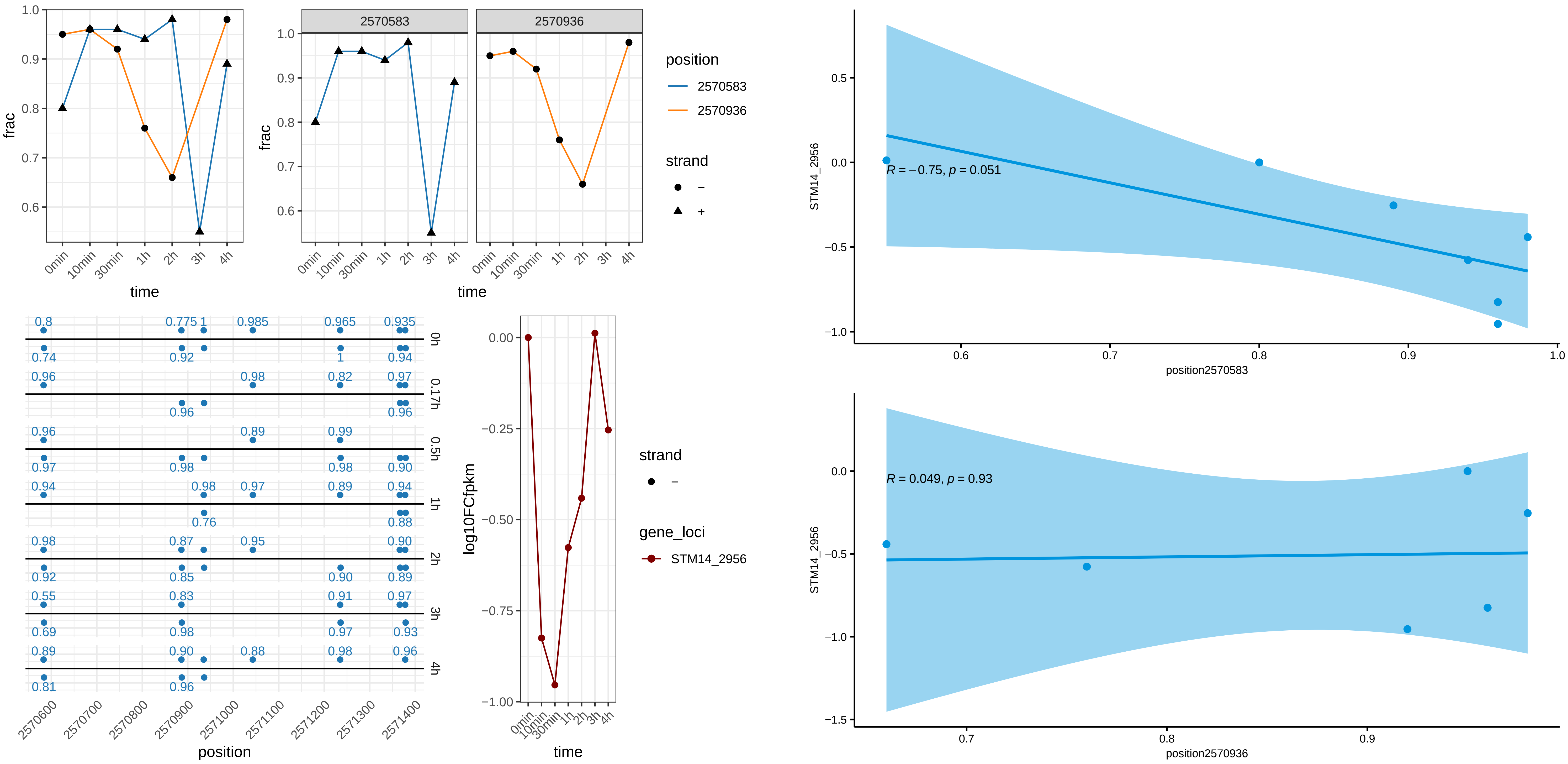

frac
time
2570583
2570936
position
2570583
2570936
strand
–
+
position
0h
0.17h
0.5h
1h
2h
3h
4h
log10FCfpkm
time
strand
–
gene_loci
STM14_2956
STM14_2956
R = −0.75, p = 0.051
position2570583
STM14_2956
R = 0.049, p = 0.93
position2570936

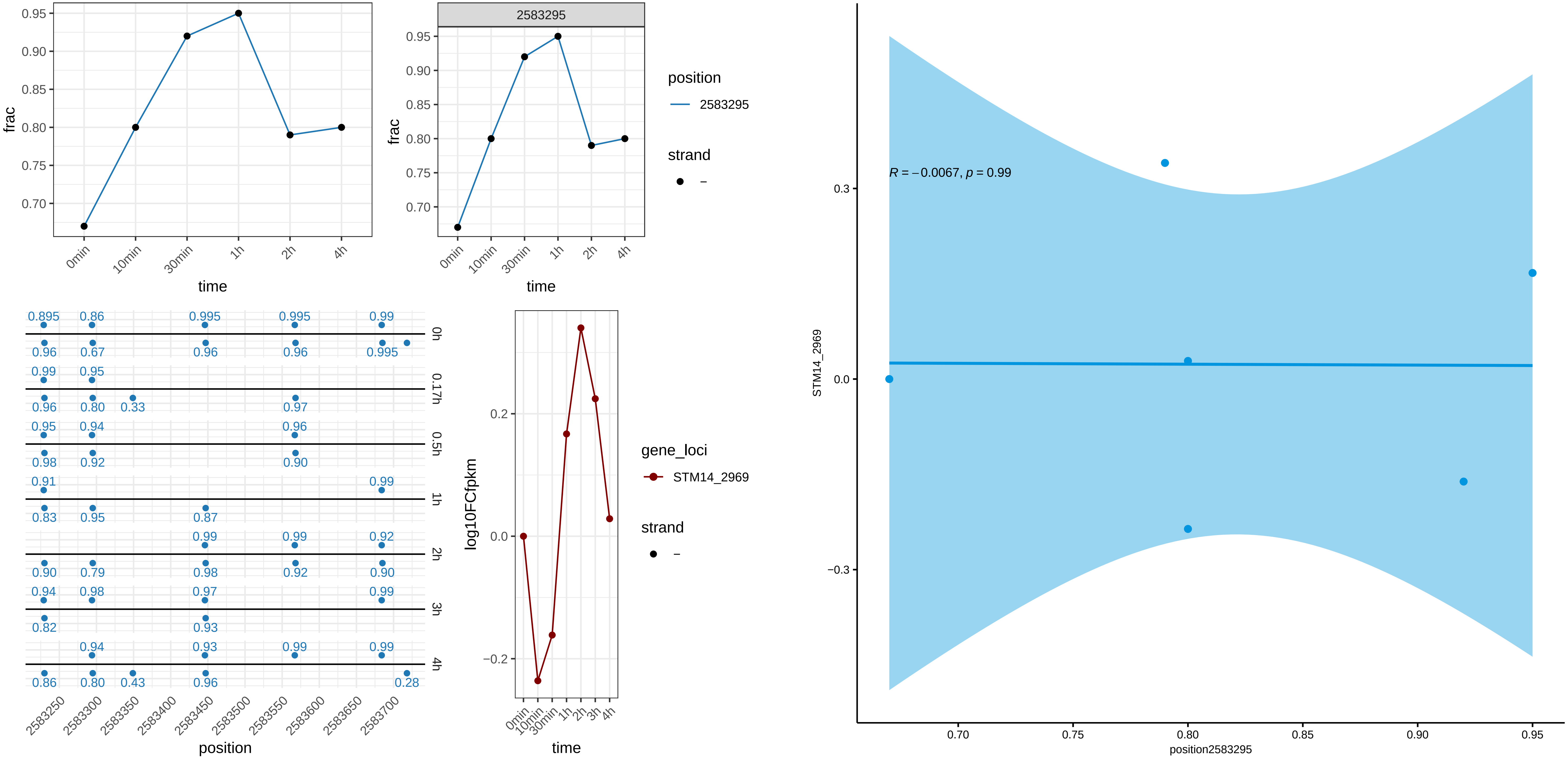
frac
time
0min
10min
30min
1h
2h
4h
2583295
position
2583295
strand
–
position
0h
0.17h
0.5h
1h
2h
3h
4h
log10FCfpkm
gene_loci
STM14_2969
strand
–
3h
R = −0.0067, p = 0.99
STM14_2969
position2583295

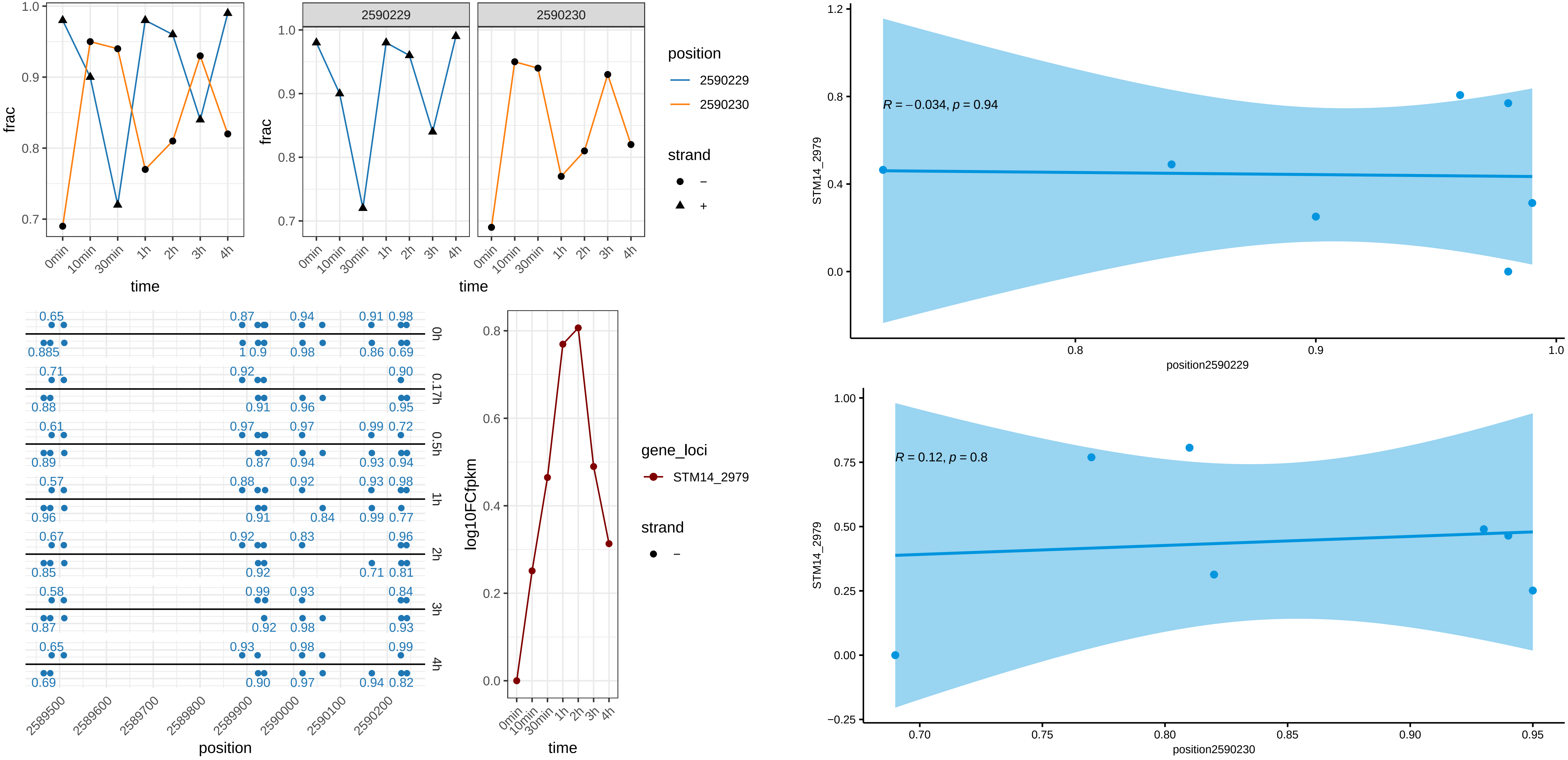

frac
time
0min
10min
30min
1h
2h
3h
4h
2590229
2590230
position
2590229
2590230
strand
–
+
position
0h
0.17h
0.5h
1h
2h
3h
4h
2589500
2589600
2589700
2589800
2589900
2590000
2590100
2590200
log10FCfpkm
gene_loci
STM14_2979
strand
–
R = –0.034, p = 0.94
STM14_2979
position2590229
R = 0.12, p = 0.8
STM14_2979
position2590230

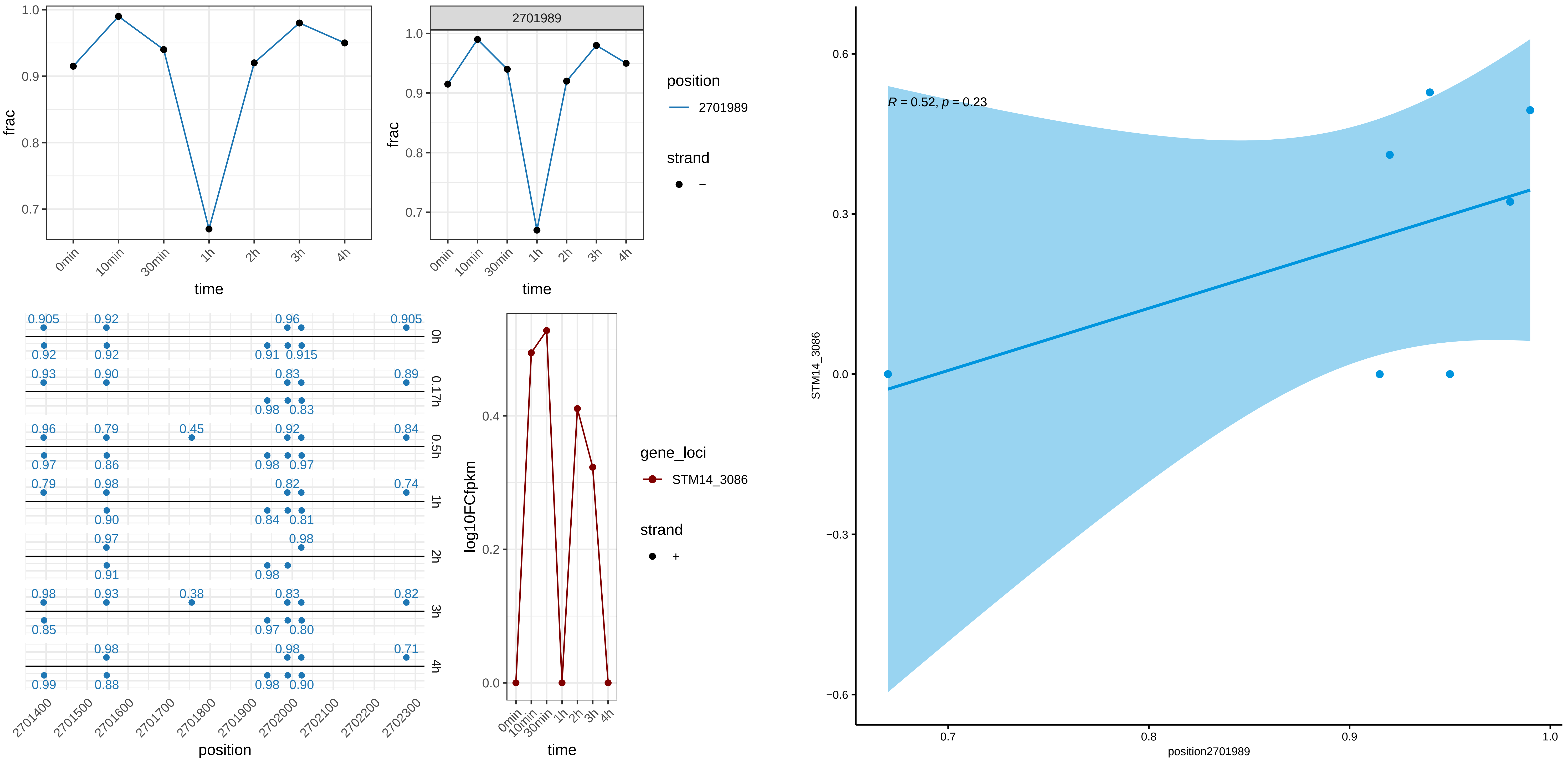

frac
time
0min
10min
30min
1h
2h
3h
4h
2701989
position
2701989
strand
−
0.905
0.92
0.96
0.905
0.92
0.92
0.91
0.915
0.93
0.90
0.83
0.89
0.98
0.83
0.96
0.79
0.45
0.92
0.84
0.97
0.86
0.98
0.97
0.79
0.98
0.82
0.74
0.90
0.84
0.81
0.97
0.98
0.91
0.98
0.98
0.93
0.38
0.83
0.82
0.85
0.97
0.80
0.98
0.98
0.71
0.99
0.88
0.98
0.90
0h
0.17h
0.5h
1h
2h
3h
4h
position
log10FCfpkm
gene_loci
STM14_3086
strand
+
R = 0.52, p = 0.23
STM14_3086
position2701989

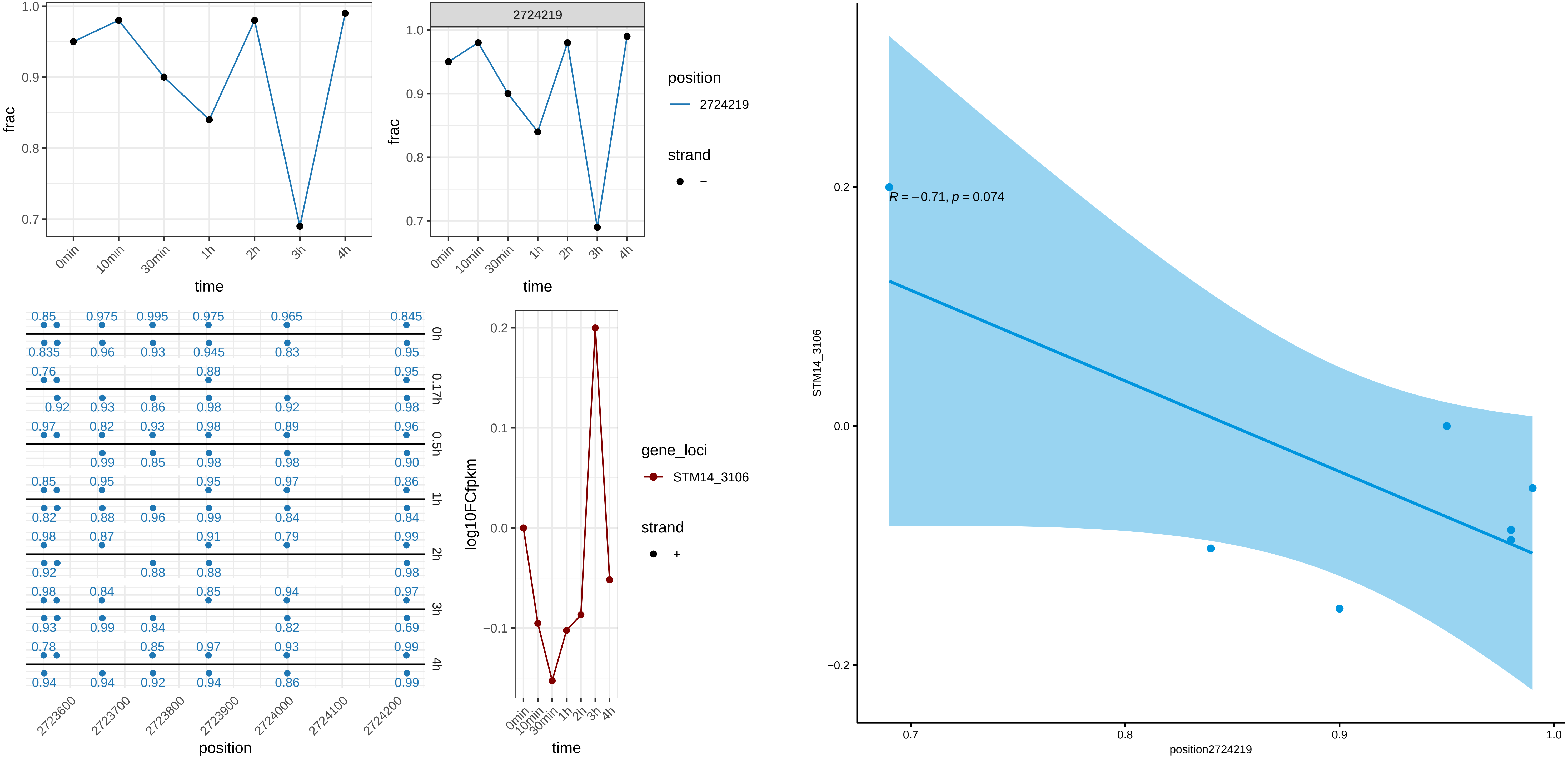

frac
time
0min
10min
30min
1h
2h
3h
4h
2724219
position
2724219
strand
−
0h
0.17h
0.5h
1h
2h
3h
4h
position
2723600
2723700
2723800
2723900
2724000
2724100
2724200
log10FCfpkm
gene_loci
STM14_3106
strand
+
R = −0.71, p = 0.074
STM14_3106
position2724219

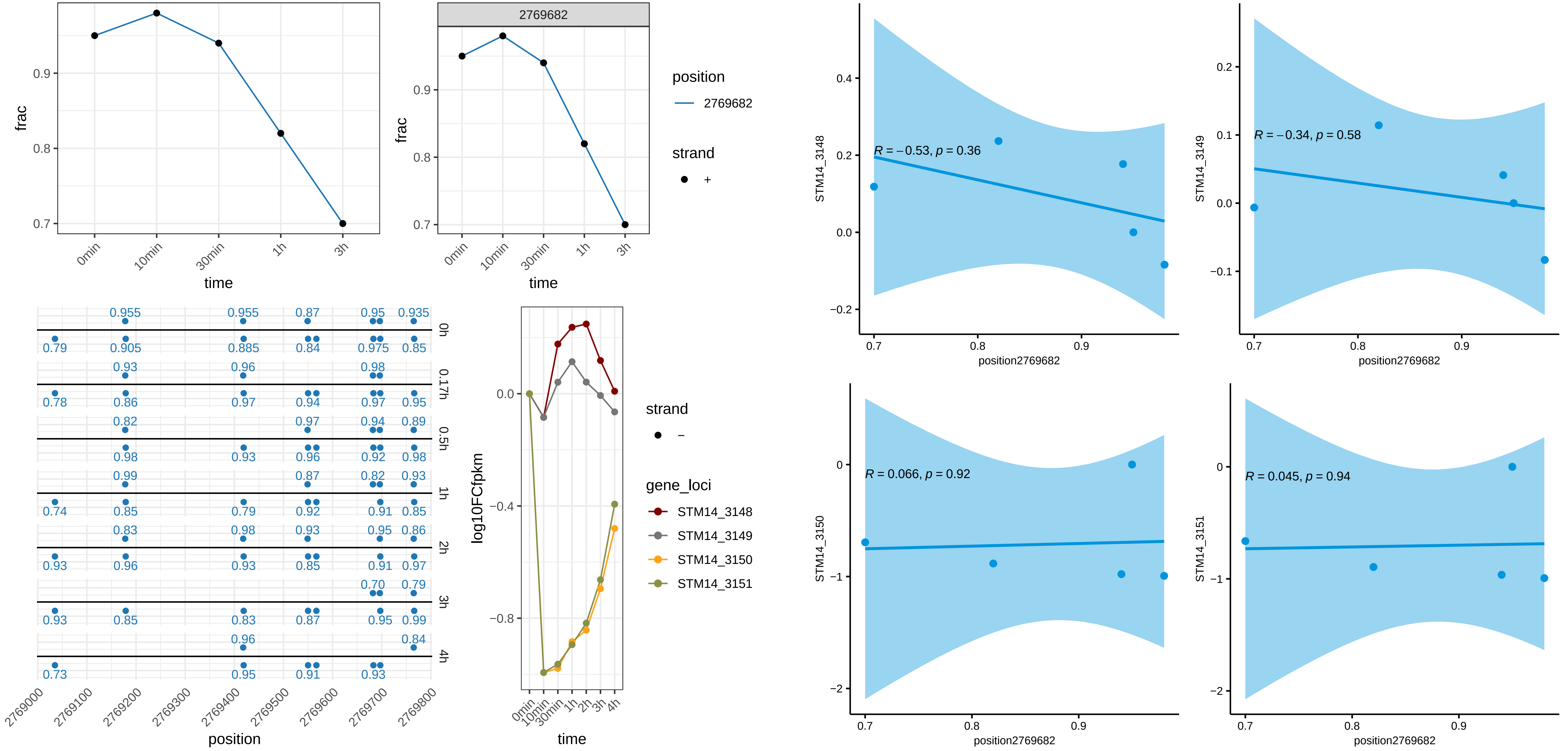

frac
time
0min
10min
30min
1h
3h
2769682
position
2769682
strand
+
0.955
0.905
0.87
0.84
0.95
0.975
0.935
0.85
0h
0.17h
0.5h
1h
2h
3h
4h
position
log10FCfpkm
time
strand
–
gene_loci
STM14_3148
STM14_3149
STM14_3150
STM14_3151
R = −0.53, p = 0.36
R = −0.34, p = 0.58
R = 0.066, p = 0.92
R = 0.045, p = 0.94
position2769682

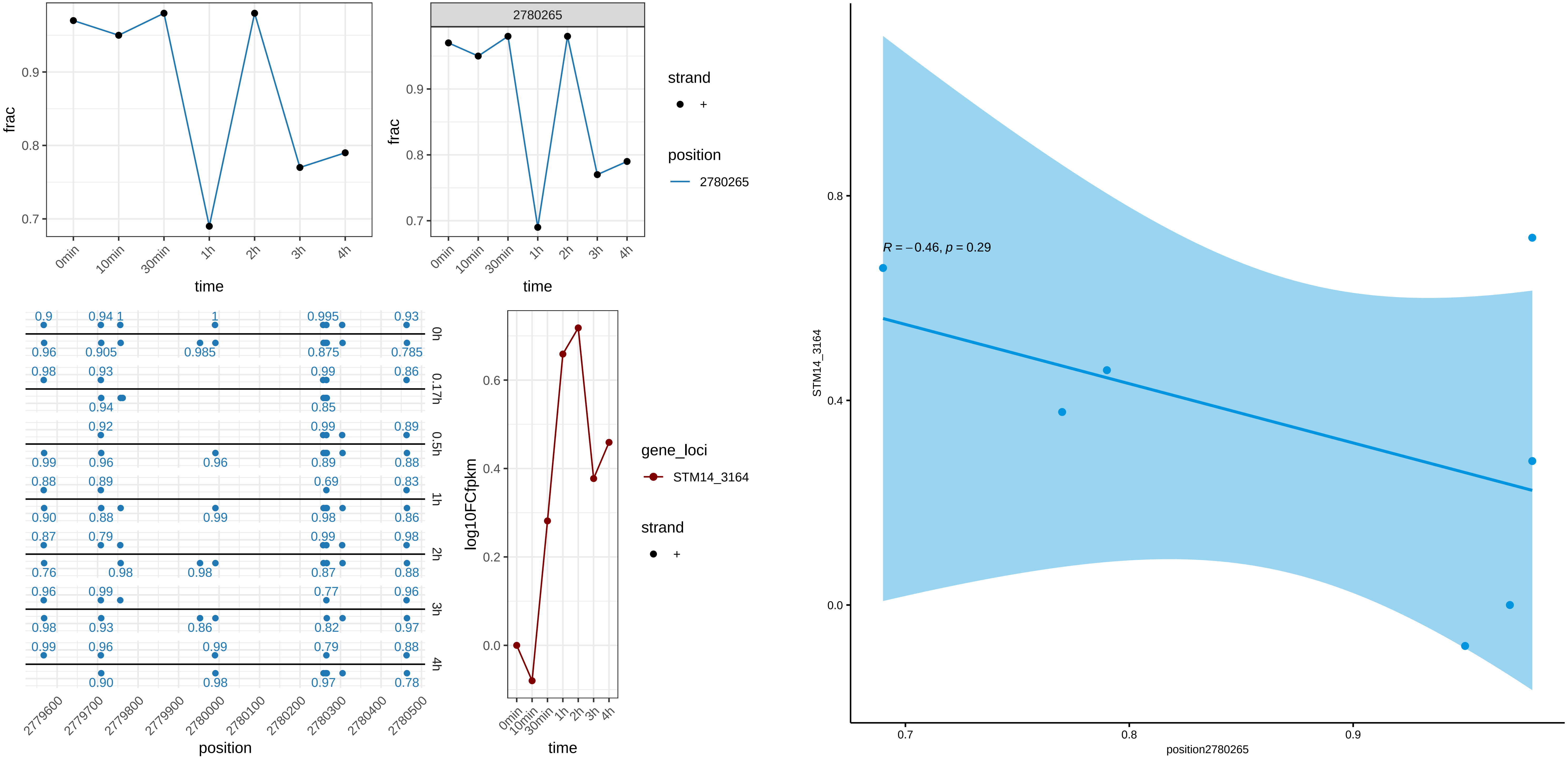

frac
time
0min
10min
30min
1h
2h
3h
4h
2780265
strand
+
position
2780265
position
0h
0.17h
0.5h
1h
2h
3h
4h
log10FCfpkm
gene_loci
STM14_3164
R = −0.46, p = 0.29
STM14_3164
position2780265

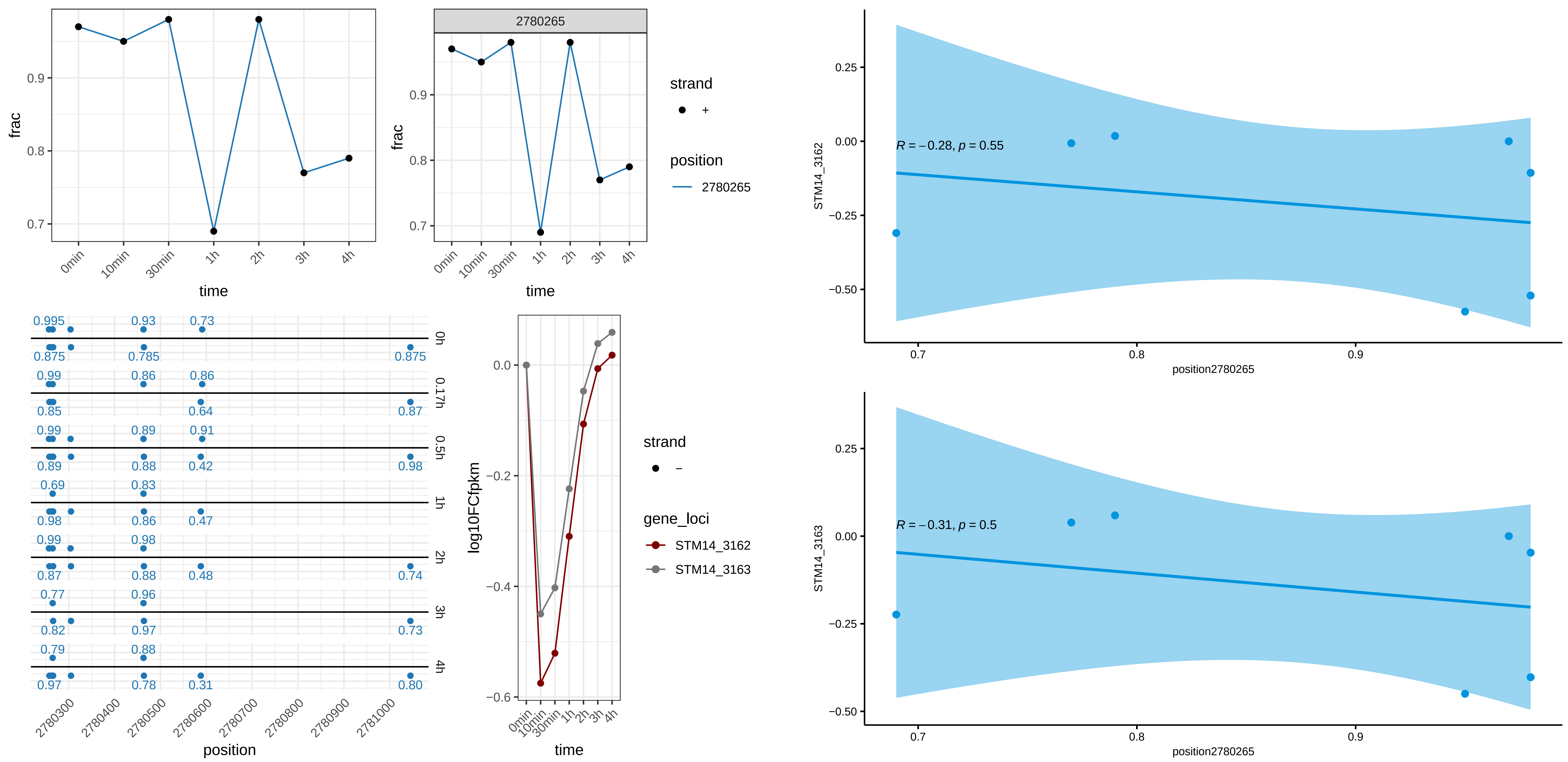
frac
time
0min
10min
30min
1h
2h
3h
4h
2780265
strand
+
position
2780265
R = −0.28, p = 0.55
STM14_3162
position2780265
R = −0.31, p = 0.5
STM14_3163
position
log10FCfpkm
strand
–
gene_loci
STM14_3162
STM14_3163
0h
0.17h
0.5h
1h
2h
3h
4h
0.995
0.93
0.73
0.875
0.785
0.875
0.99
0.86
0.86
0.85
0.64
0.87
0.99
0.89
0.91
0.89
0.88
0.42
0.98
0.69
0.83
0.98
0.86
0.47
0.99
0.98
0.87
0.88
0.48
0.74
0.77
0.96
0.82
0.97
0.73
0.79
0.88
0.97
0.78
0.31
0.80
2780300
2780400
2780500
2780600
2780700
2780800
2780900
2781000

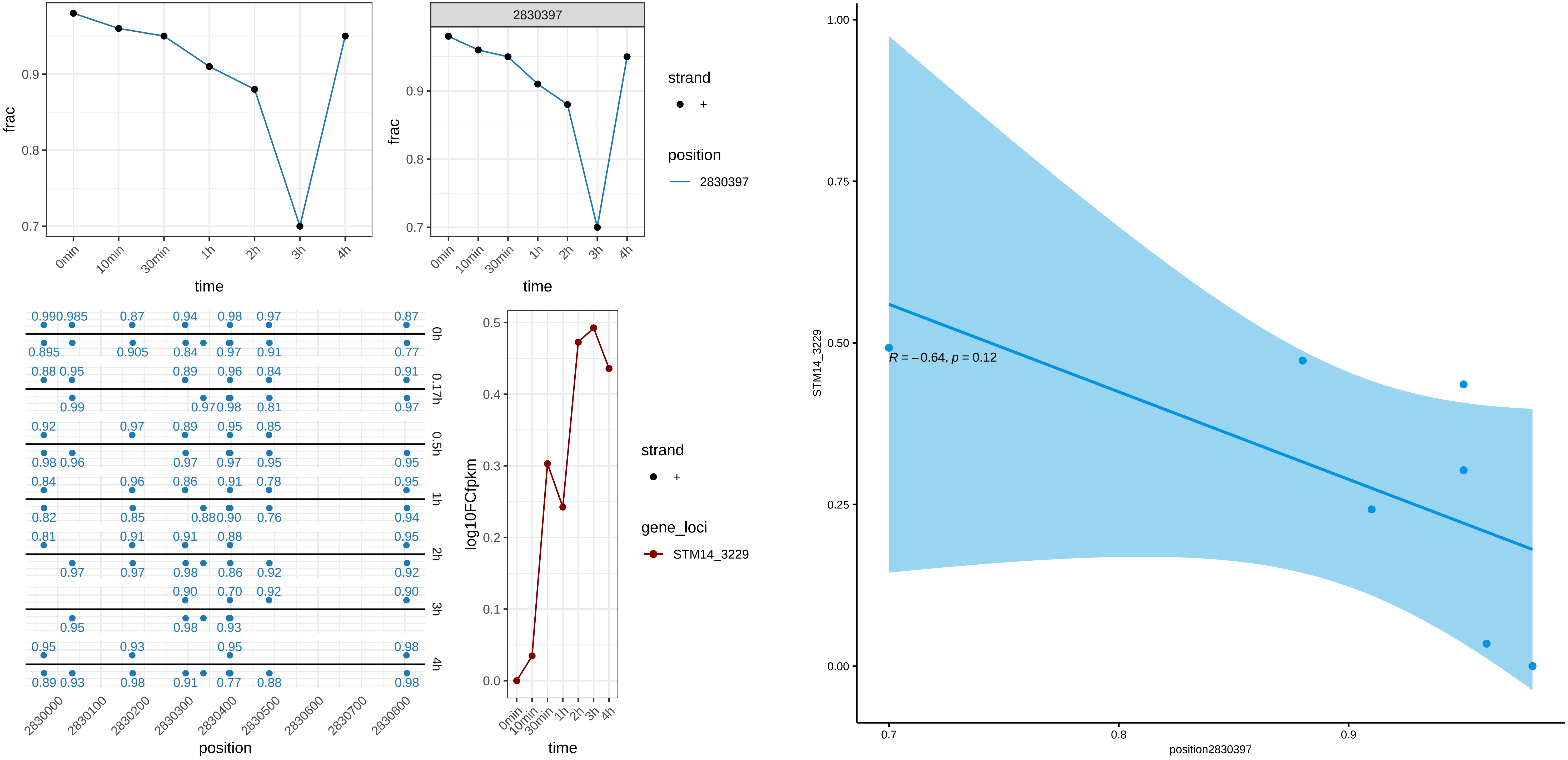

frac
time
0min
10min
30min
1h
2h
3h
4h
2830397
strand
+
position
2830397
0h
0.17h
0.5h
1h
2h
3h
4h
log10FCfpkm
gene_loci
STM14_3229
STM14_3229
R = −0.64, p = 0.12
position2830397

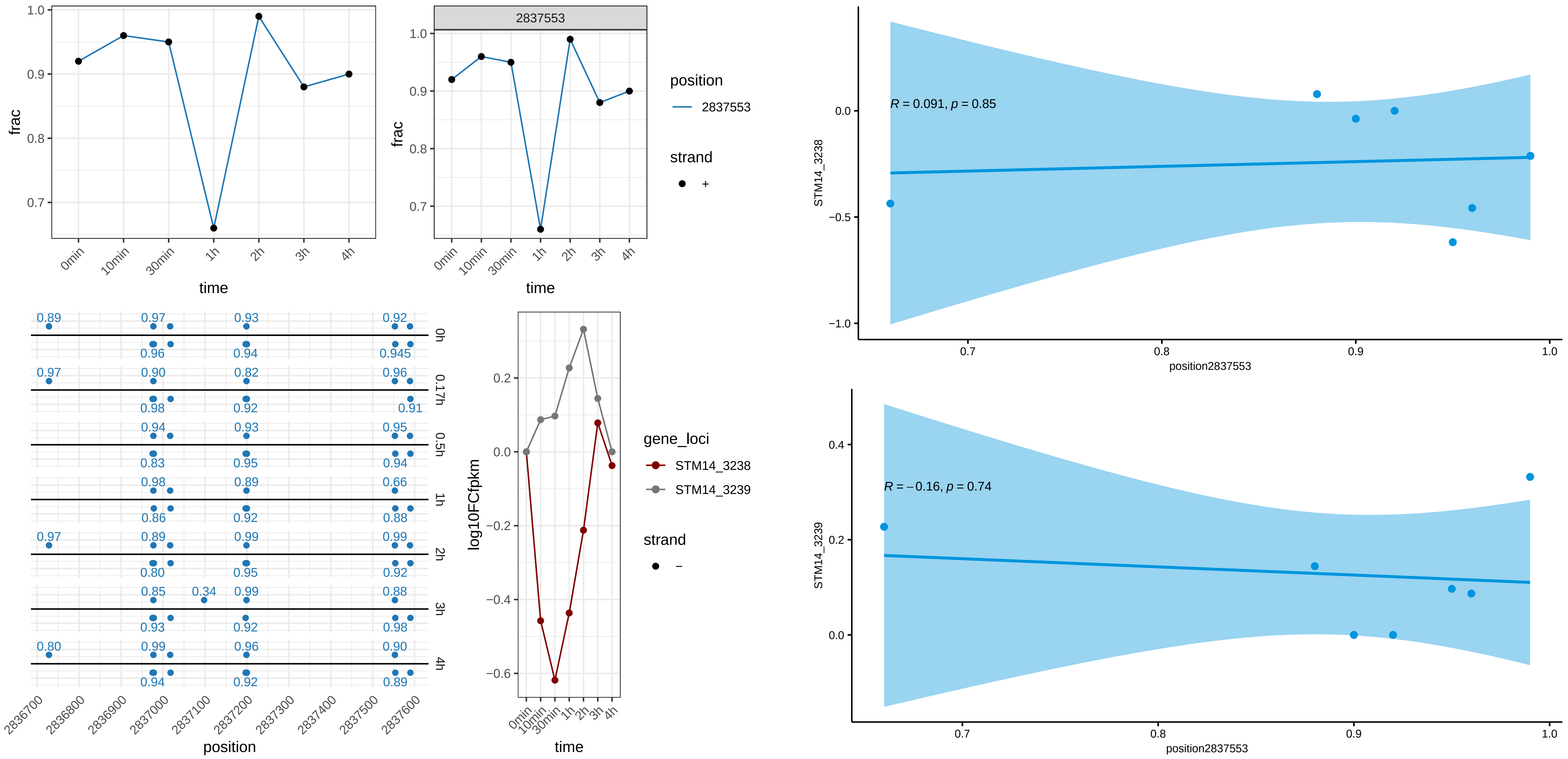

frac
time
2837553
position
2837553
strand
+
0min
10min
30min
1h
2h
3h
4h
0h
0.17h
0.5h
position
log10FCfpkm
gene_loci
STM14_3238
STM14_3239
strand
–
R = 0.091, p = 0.85
STM14_3238
position2837553
R = –0.16, p = 0.74
STM14_3239
position2837553

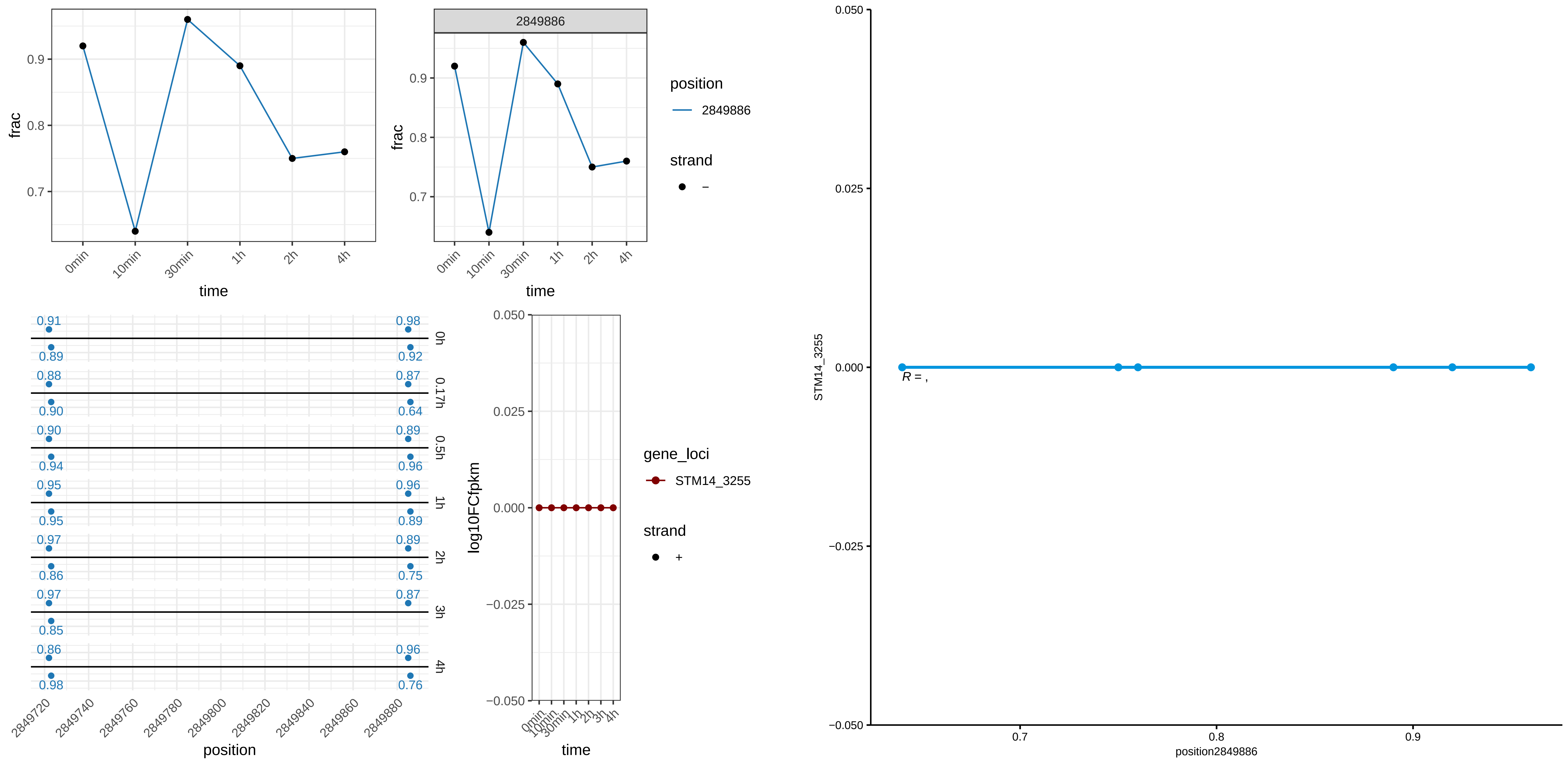

frac
time
0min
10min
30min
1h
2h
4h
2849886
position
2849886
strand
–
0h
0.17h
0.5h
1h
2h
3h
4h
position
log10FCfpkm
gene_loci
STM14_3255
strand
+
STM14_3255
R = ,
position2849886

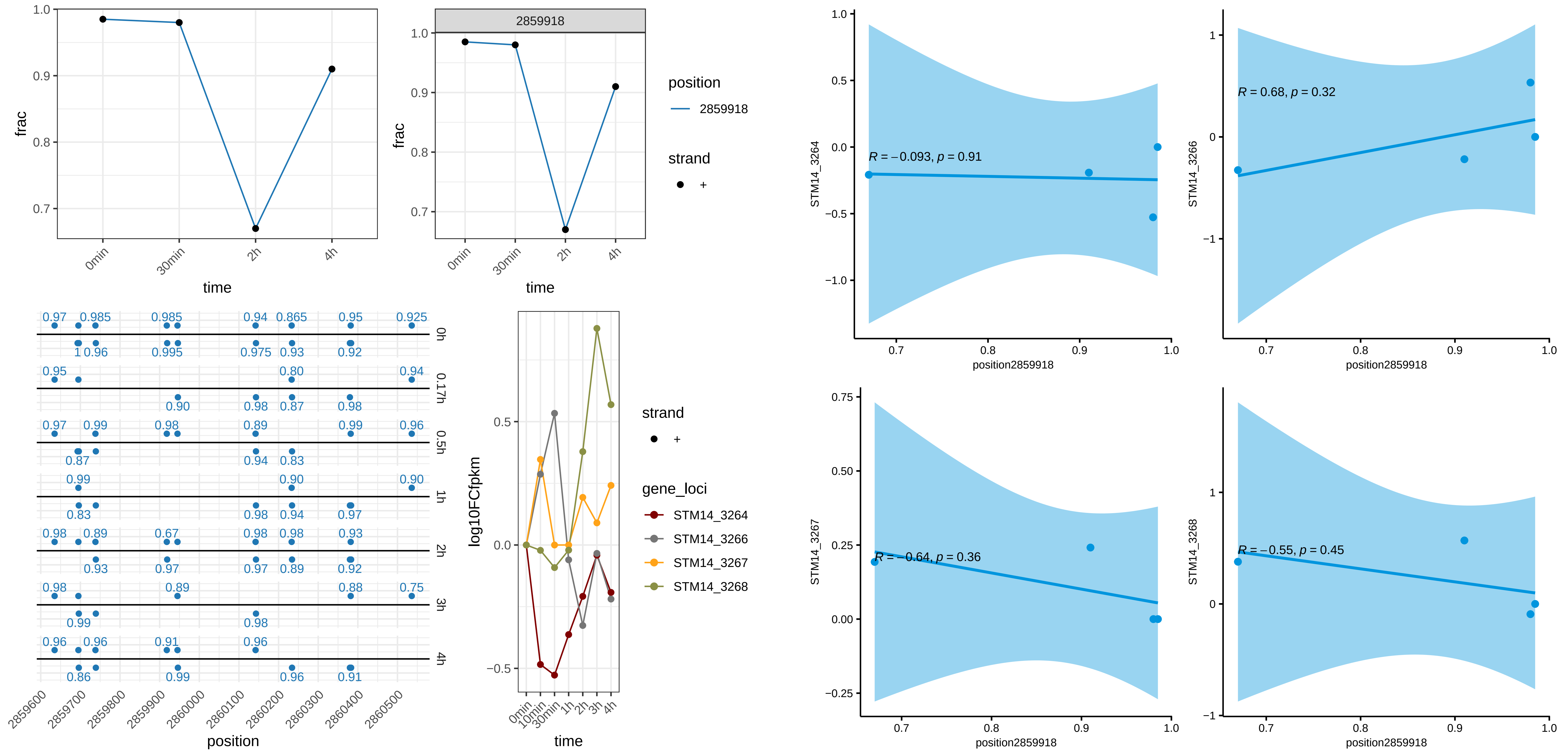
frac
time
0min
30min
2h
4h
2859918
position
2859918
strand
+
log10FCfpkm
gene_loci
STM14_3264
STM14_3266
STM14_3267
STM14_3268
0h
0.17h
0.5h
1h
2h
3h
4h
position
STM14_3264
R = −0.093, p = 0.91
position2859918
STM14_3266
R = 0.68, p = 0.32
STM14_3267
R = −0.64, p = 0.36
STM14_3268
R = −0.55, p = 0.45

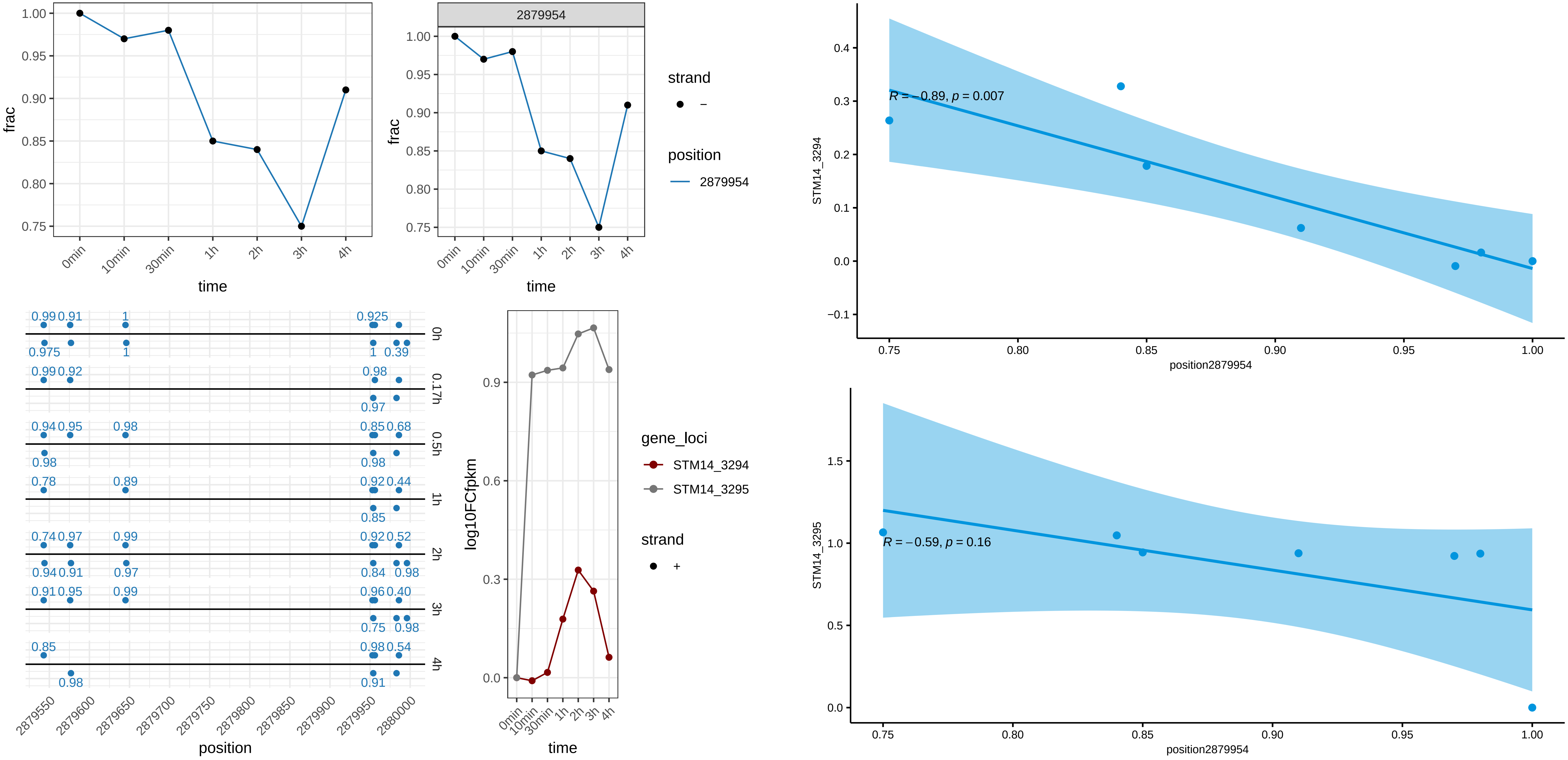

frac
time
0min
10min
30min
1h
2h
3h
4h
2879954
strand
–
position
2879954
R = −0.89, p = 0.007
STM14_3294
position2879954
0.75
0.80
0.85
0.90
0.95
1.00
0h
0.17h
0.5h
1h
2h
3h
4h
position
log10FCfpkm
gene_loci
STM14_3294
STM14_3295
strand
+
R = −0.59, p = 0.16
STM14_3295
position2879954

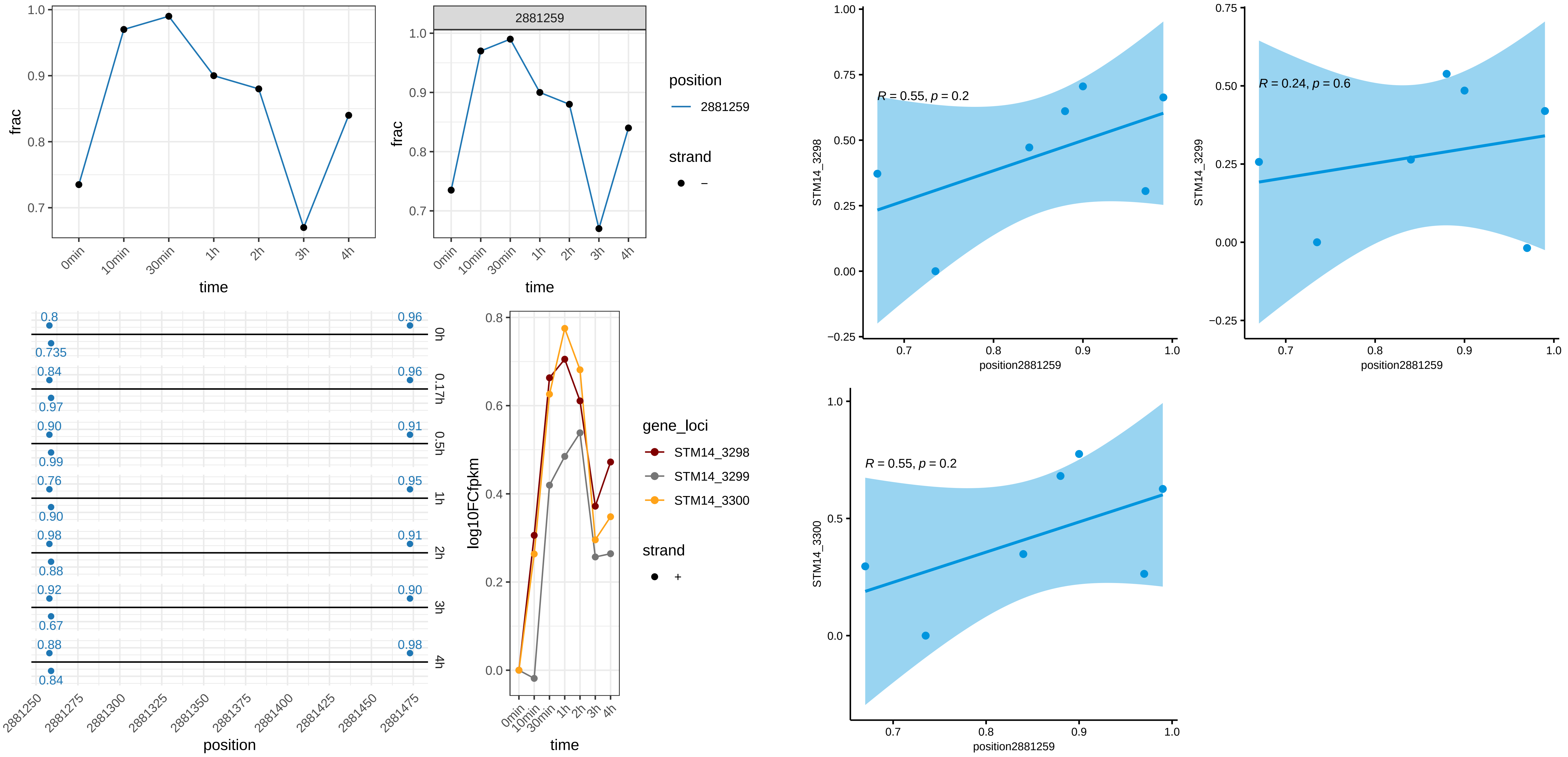

frac
time
2881259
position
strand
log10FCfpkm
gene_loci
STM14_3298
STM14_3299
STM14_3300
0min
10min
30min
1h
2h
3h
4h
0h
0.17h
0.5h
R = 0.55, p = 0.2
R = 0.24, p = 0.6
R = 0.55, p = 0.2
position2881259

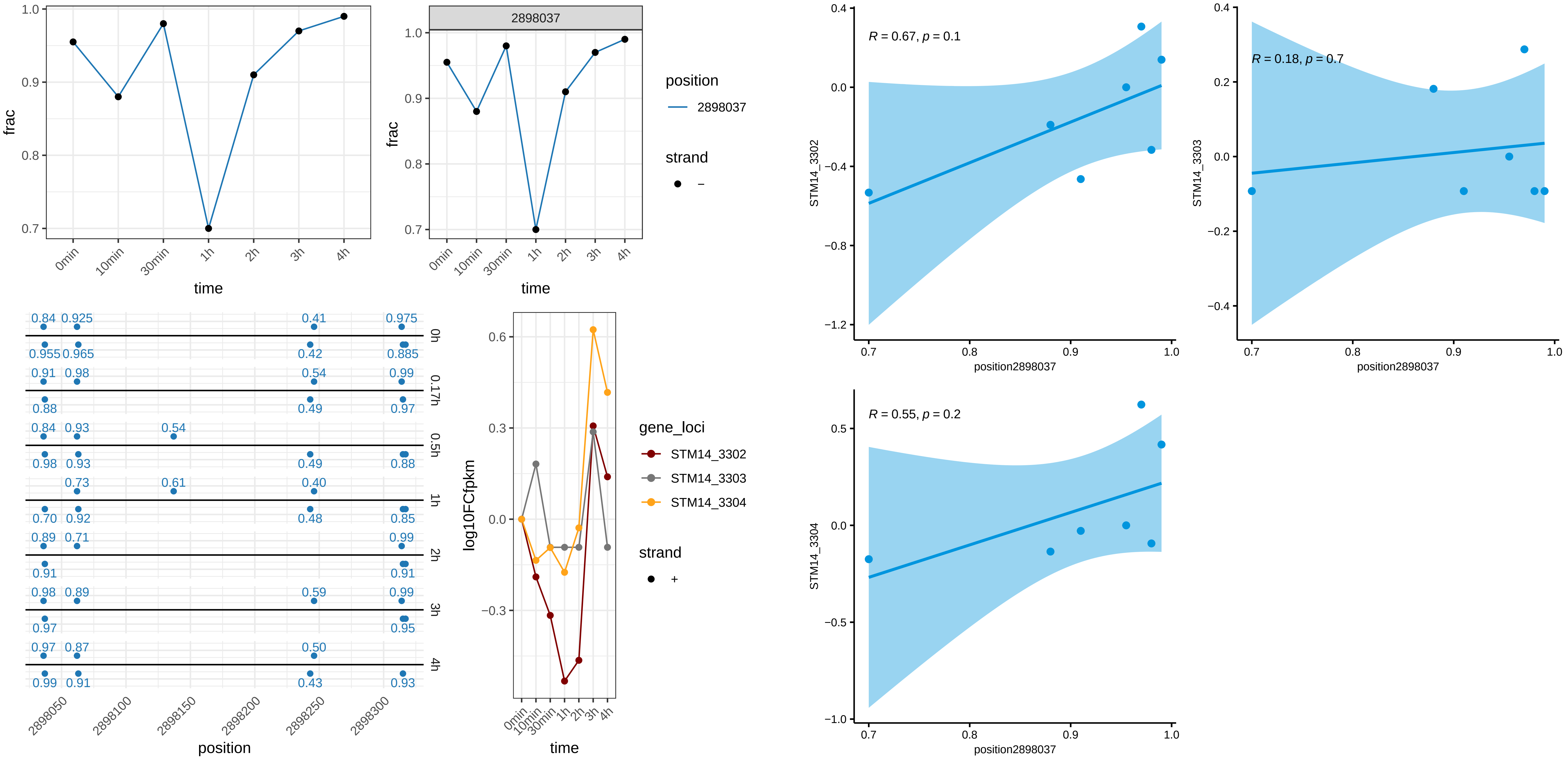

frac
time
0min
10min
30min
1h
2h
3h
4h
2898037
position
2898037
strand
–
R = 0.67, p = 0.1
STM14_3302
position2898037
R = 0.18, p = 0.7
STM14_3303
position2898037
0h
0.17h
0.5h
1h
2h
3h
4h
position
log10FCfpkm
gene_loci
STM14_3302
STM14_3303
STM14_3304
strand
+
R = 0.55, p = 0.2
STM14_3304
position2898037

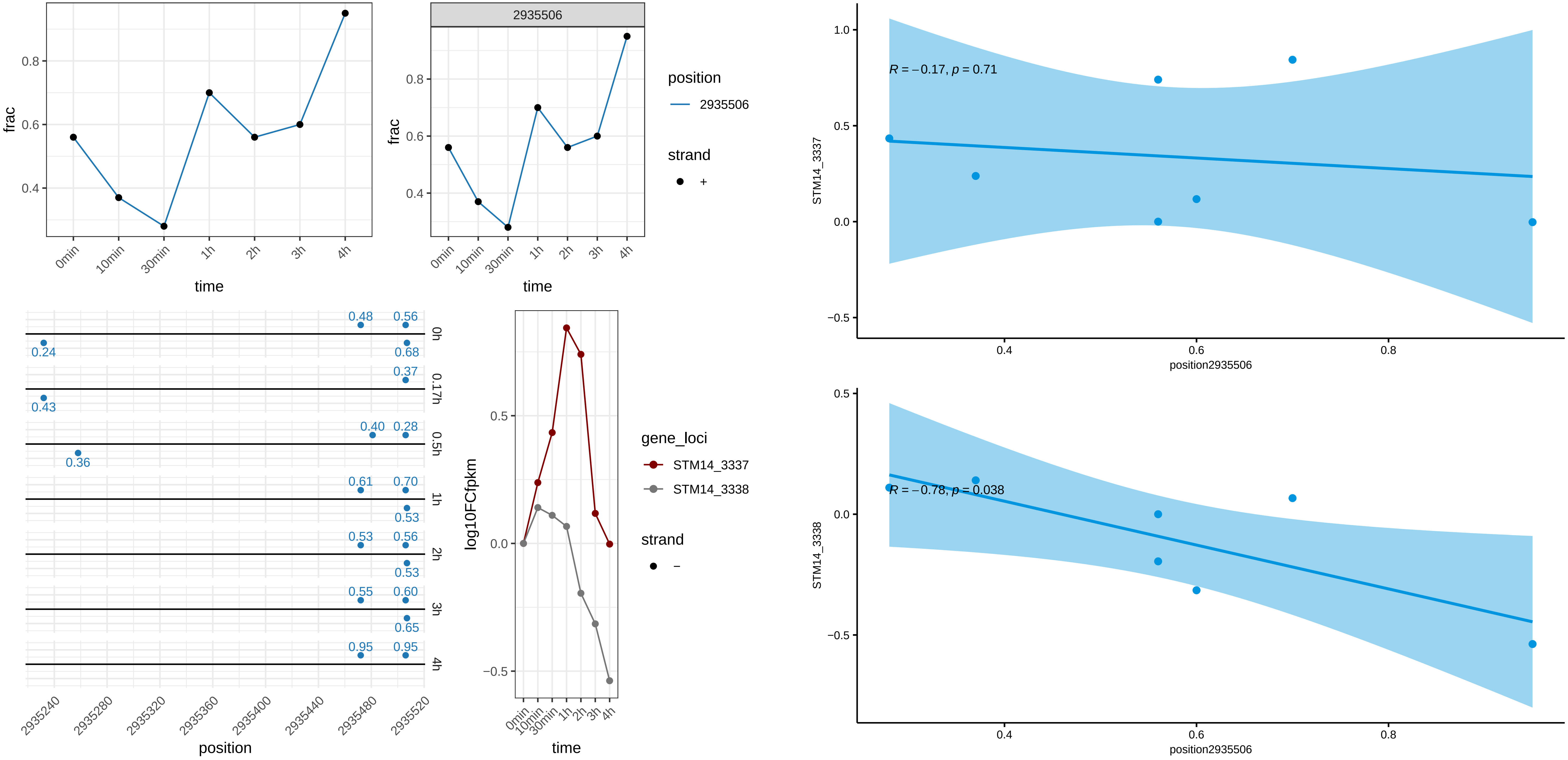

frac
0.4
0.6
0.8
0min
10min
30min
1h
2h
3h
4h
time
2935506
position
2935506
strand
+
0.48
0.56
0.24
0.68
0h
0.37
0.43
0.17h
0.40
0.28
0.36
0.5h
0.61
0.70
0.53
1h
0.53
0.56
0.53
2h
0.55
0.60
0.65
3h
0.95
0.95
4h
2935240
2935280
2935320
2935360
2935400
2935440
2935480
2935520
position
log10FCfpkm
0.5
0.0
−0.5
gene_loci
STM14_3337
STM14_3338
strand
–
STM14_3337
1.0
0.5
0.0
−0.5
$R = -0.17, p = 0.71$
0.4
0.6
0.8
position2935506
STM14_3338
$R = -0.78, p = 0.038$
position2935506

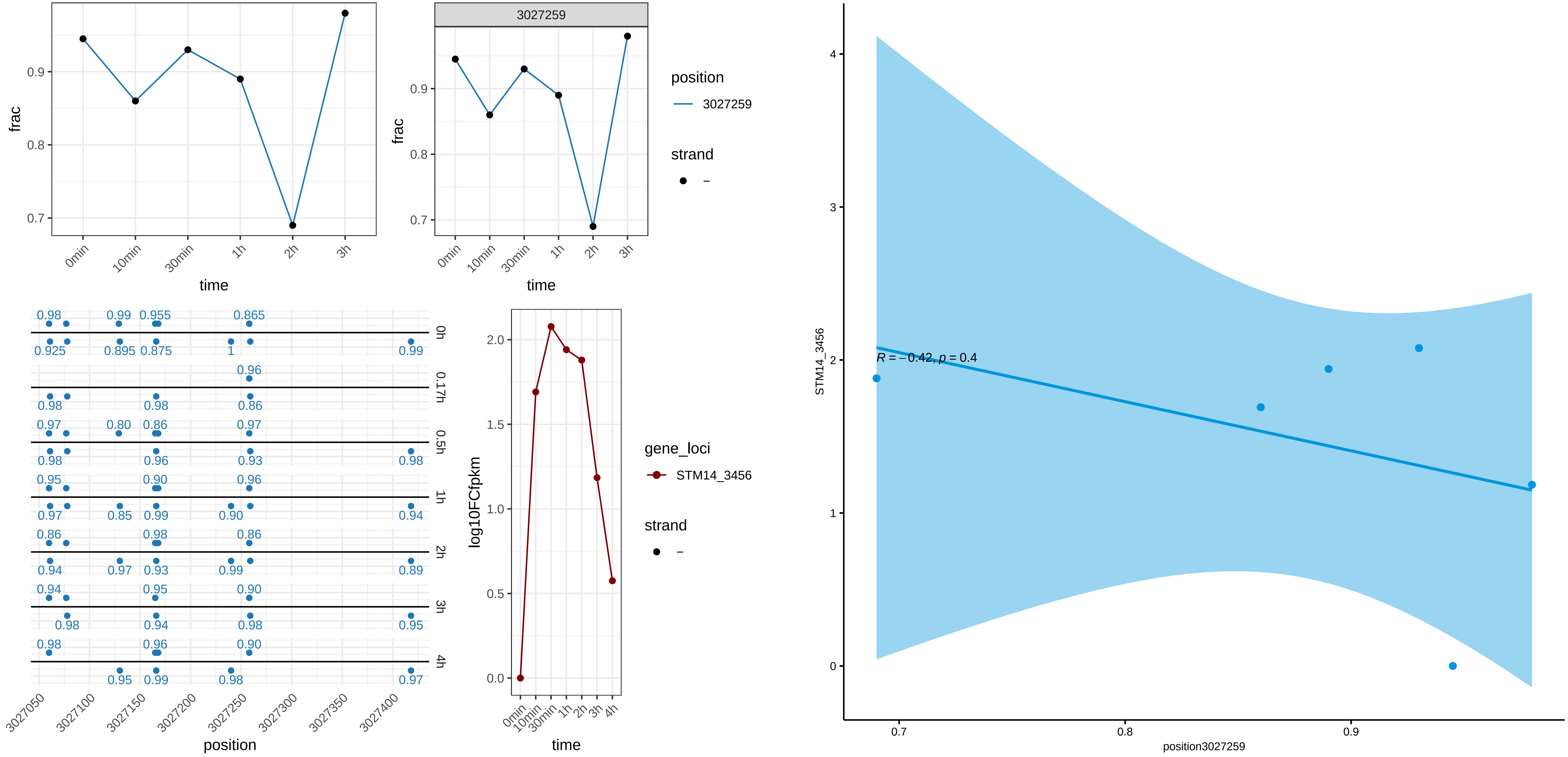

frac
time
0min
10min
30min
1h
2h
3h
3027259
position
3027259
strand
–
log10FCfpkm
gene_loci
STM14_3456
strand
–
0h
0.17h
0.5h
1h
2h
3h
4h
position
3027050
3027100
3027150
3027200
3027250
3027300
3027350
3027400
STM14_3456
R = −0.42, p = 0.4
position3027259

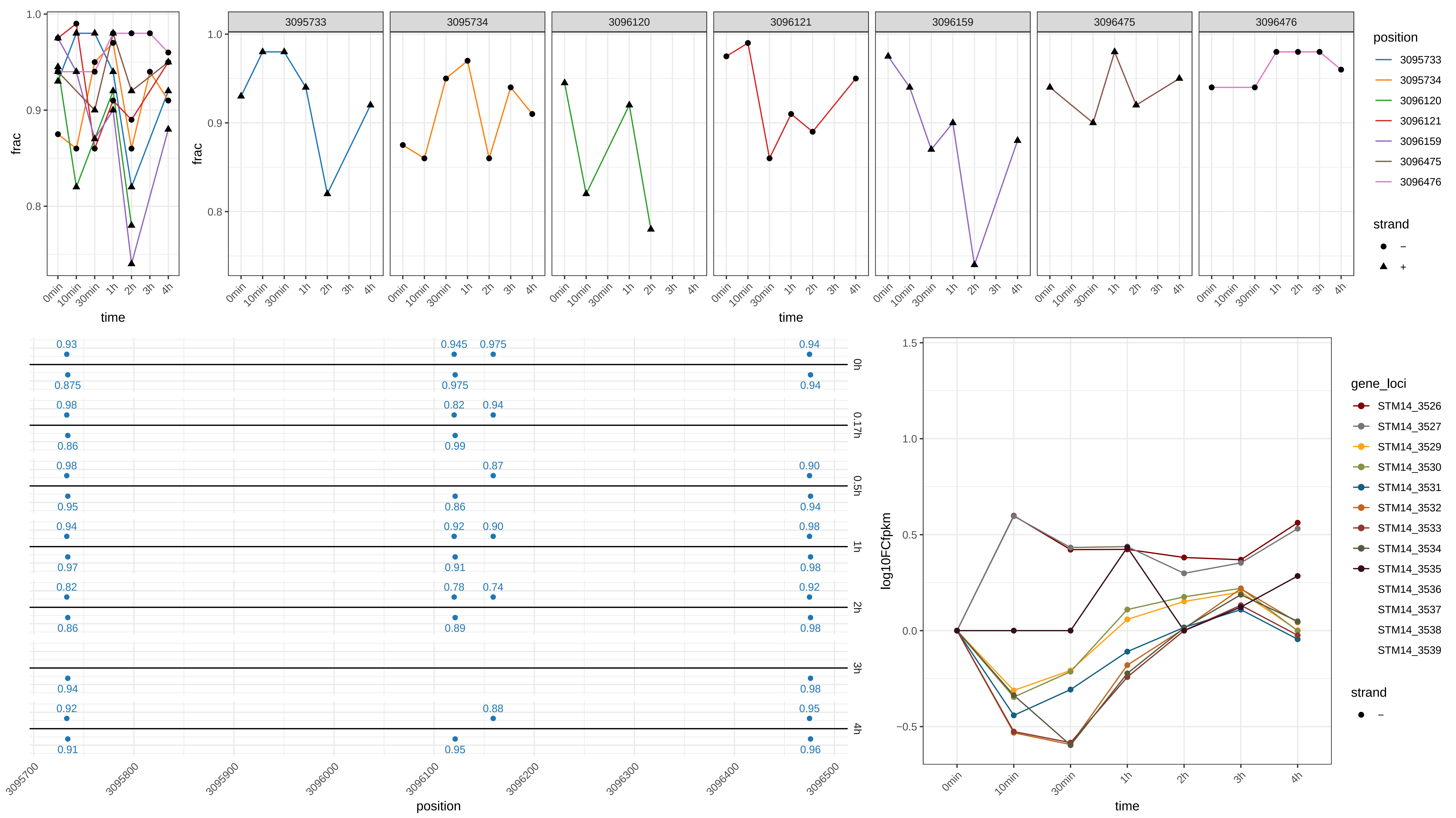

frac
time
3095733
3095734
3096120
3096121
3096159
3096475
3096476
position
strand
position
log10FCfpkm
time
gene_loci
STM14_3526
STM14_3527
STM14_3529
STM14_3530
STM14_3531
STM14_3532
STM14_3533
STM14_3534
STM14_3535
STM14_3536
STM14_3537
STM14_3538
STM14_3539

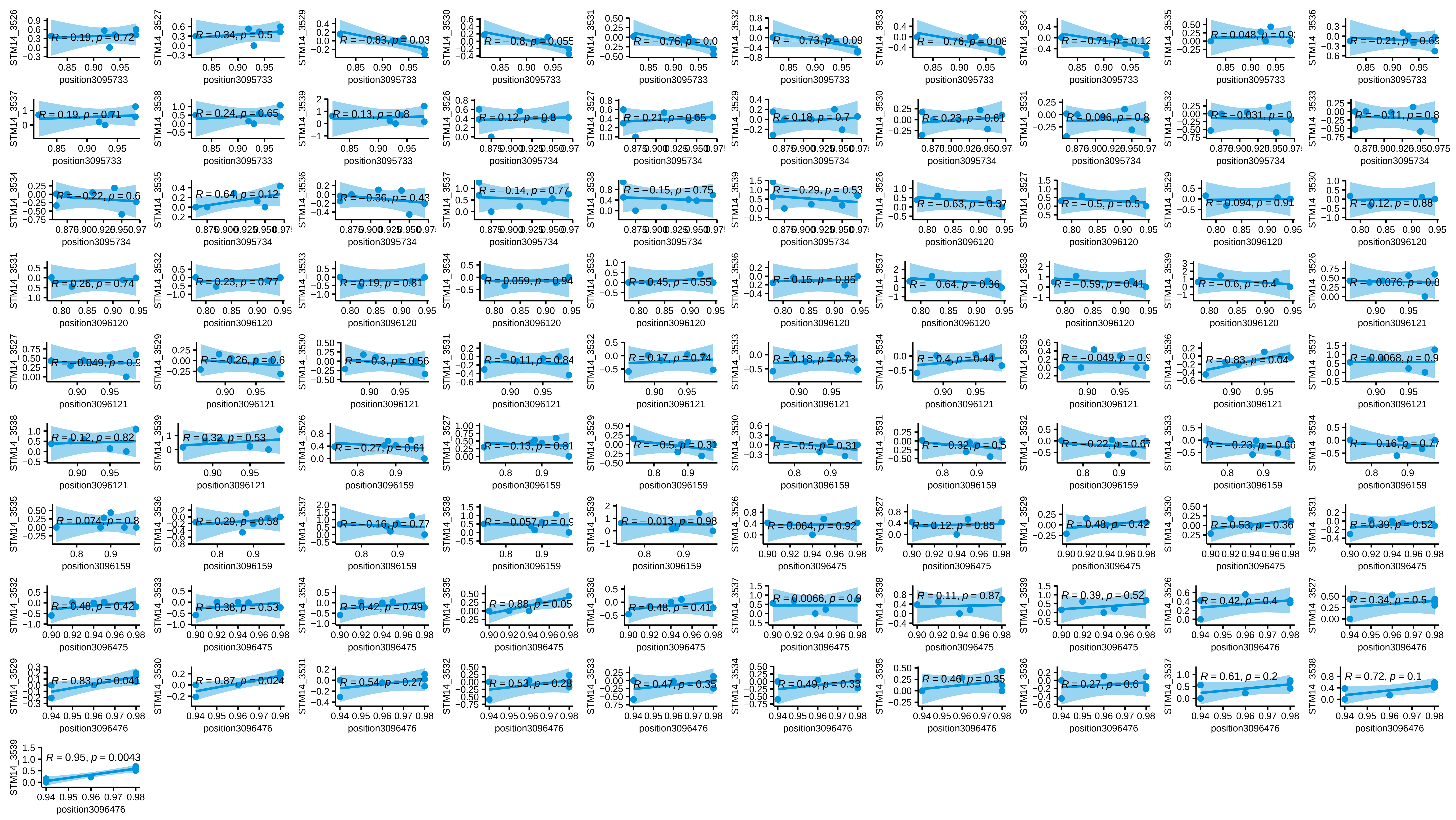

position3095733
position3095734
position3096120
position3096121
position3096159
position3096475
position3096476

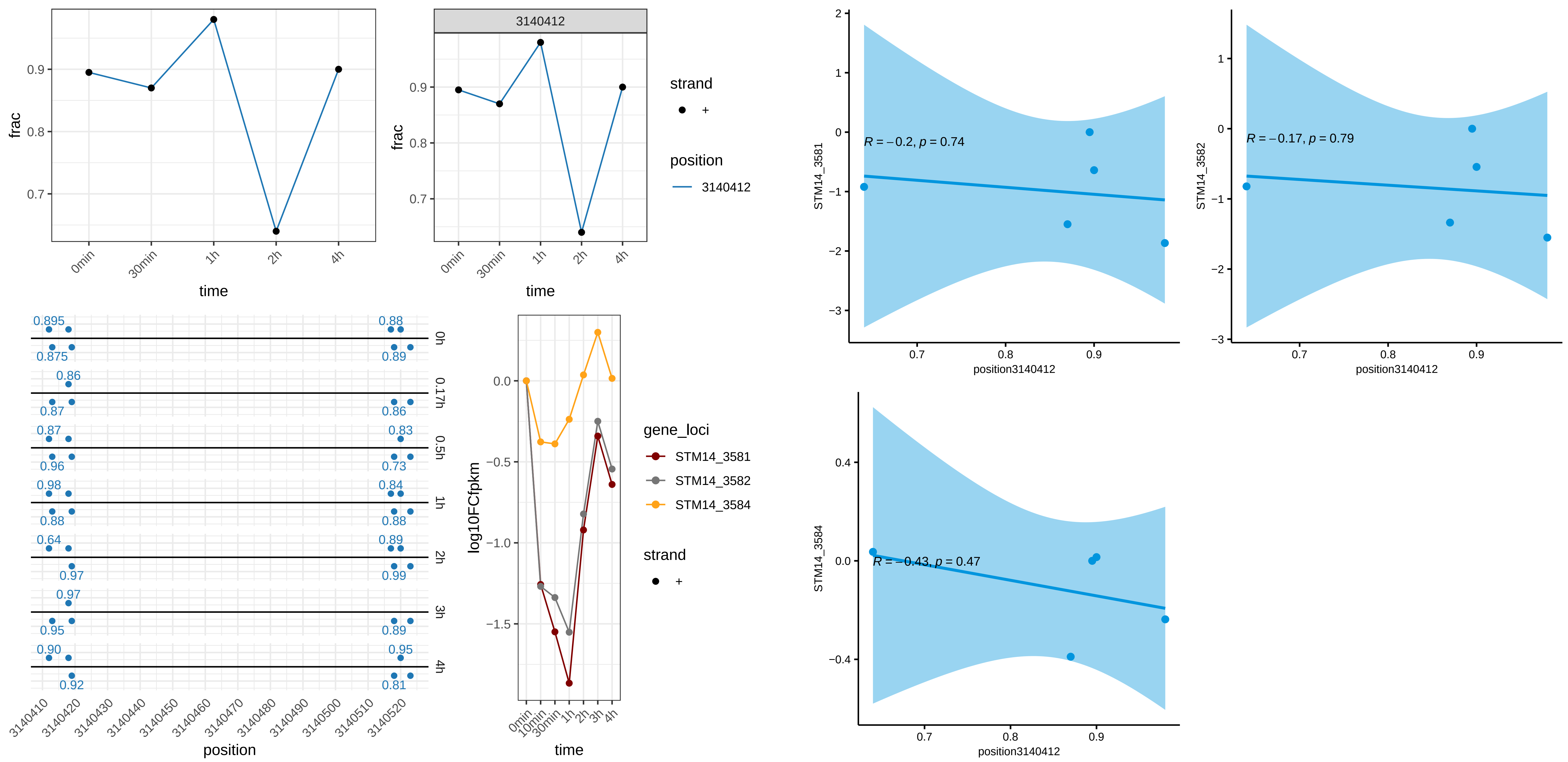

frac
time
0min
30min
1h
2h
4h
3140412
strand
+
position
3140412
position
log10FCfpkm
gene_loci
STM14_3581
STM14_3582
STM14_3584
R = −0.2, p = 0.74
R = −0.17, p = 0.79
R = −0.43, p = 0.47
STM14_3581
STM14_3582
STM14_3584
position3140412

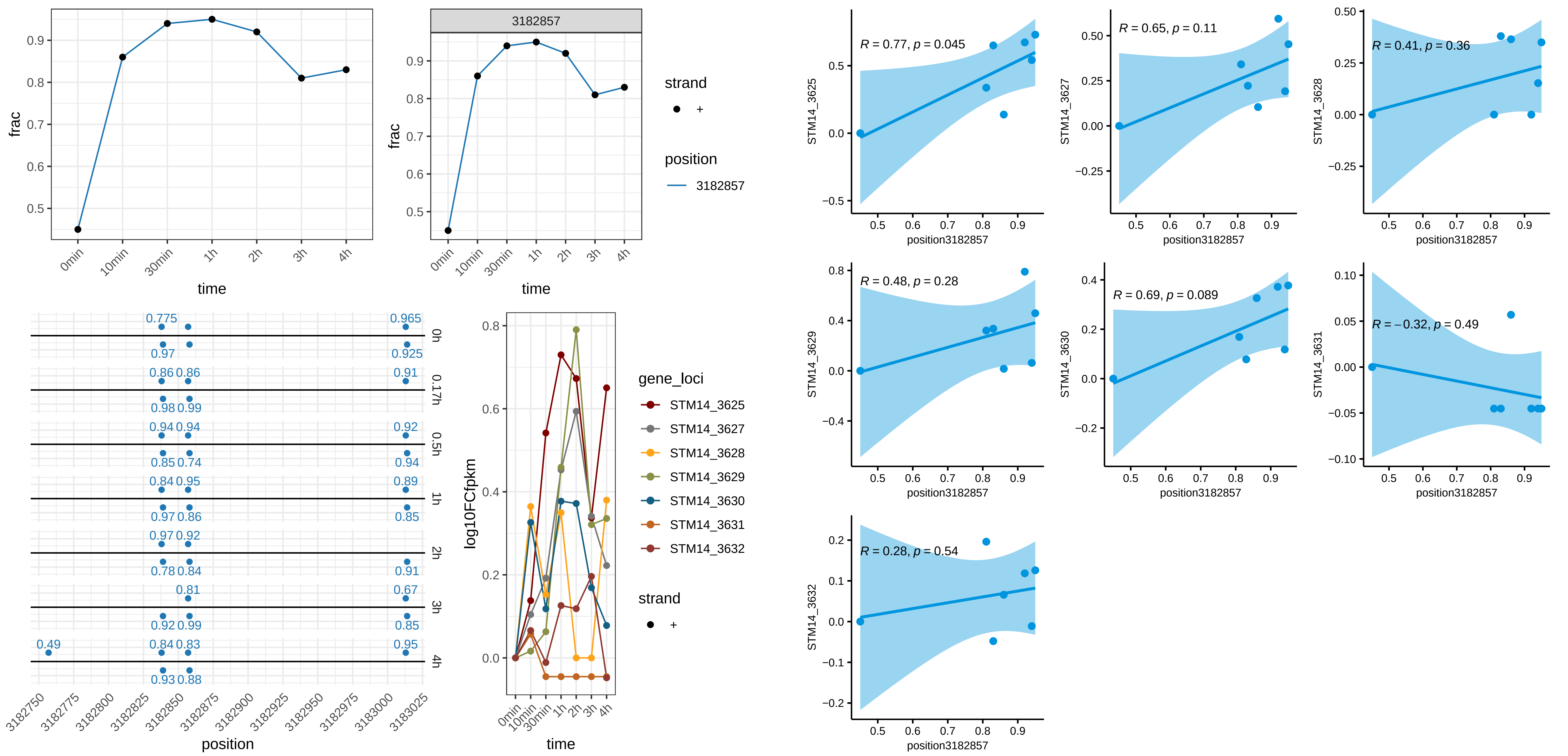

frac
time
3182857
strand
+
position
3182857
0.775
0.965
0.97
0.925
0.86 0.86
0.91
0.98 0.99
0.94 0.94
0.92
0.85 0.74
0.94
0.84 0.95
0.89
0.97 0.86
0.85
0.97 0.92
0.78 0.84
0.91
0.81
0.67
0.92 0.99
0.85
0.49
0.84 0.83
0.95
0.93 0.88
0h
0.17h
0.5h
1h
2h
3h
4h
position
log10FCfpkm
gene_loci
STM14_3625
STM14_3627
STM14_3628
STM14_3629
STM14_3630
STM14_3631
STM14_3632
strand
+
R = 0.77, p = 0.045
R = 0.65, p = 0.11
R = 0.41, p = 0.36
R = 0.48, p = 0.28
R = 0.69, p = 0.089
R = −0.32, p = 0.49
R = 0.28, p = 0.54
position3182857

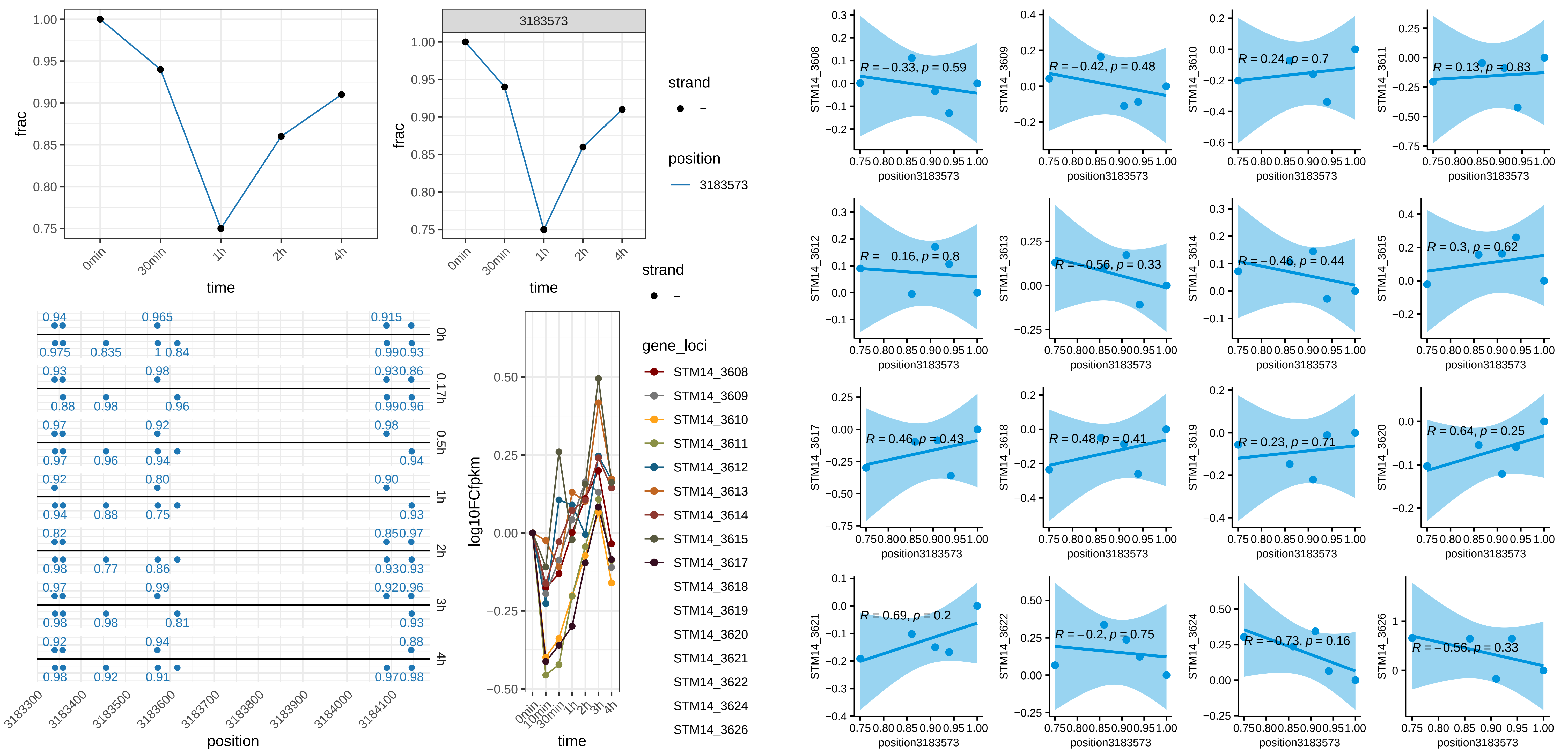

frac
time
3183573
strand
position
3183573
log10FCfpkm
gene_loci
STM14_3608
STM14_3609
STM14_3610
STM14_3611
STM14_3612
STM14_3613
STM14_3614
STM14_3615
STM14_3617
STM14_3618
STM14_3619
STM14_3620
STM14_3621
STM14_3622
STM14_3624
STM14_3626
position3183573
R = −0.33, p = 0.59
R = −0.42, p = 0.48
R = 0.24, p = 0.7
R = 0.13, p = 0.83
R = −0.16, p = 0.8
R = −0.56, p = 0.33
R = −0.46, p = 0.44
R = 0.3, p = 0.62
R = 0.46, p = 0.43
R = 0.48, p = 0.41
R = 0.23, p = 0.71
R = 0.64, p = 0.25
R = 0.69, p = 0.2
R = −0.2, p = 0.75
R = −0.73, p = 0.16
R = −0.56, p = 0.33

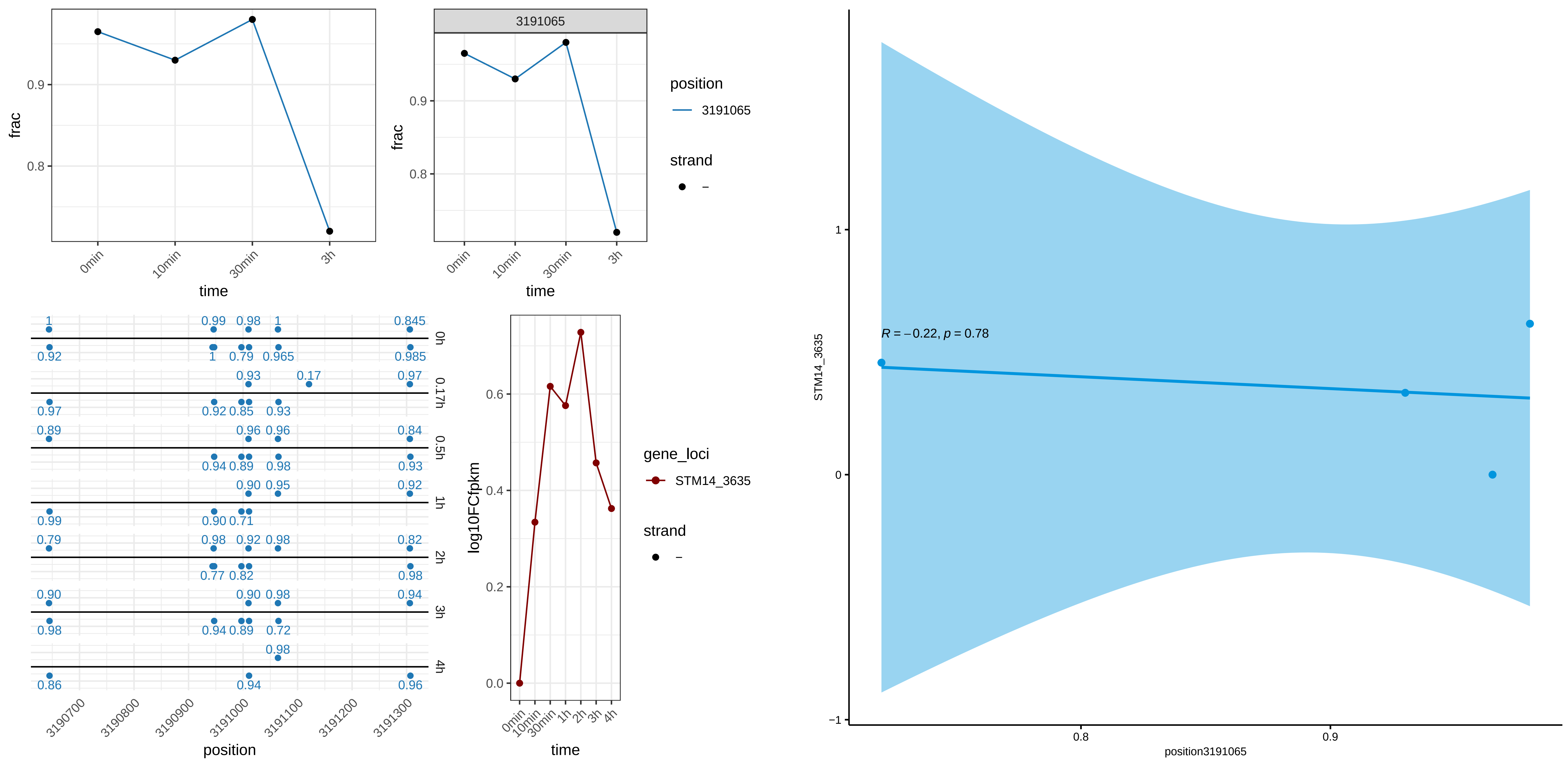

frac
time
0min
10min
30min
3h
3191065
position
3191065
strand
–
log10FCfpkm
gene_loci
STM14_3635
position
0h
0.17h
0.5h
1h
2h
3h
4h
R = –0.22, p = 0.78
STM14_3635
position3191065

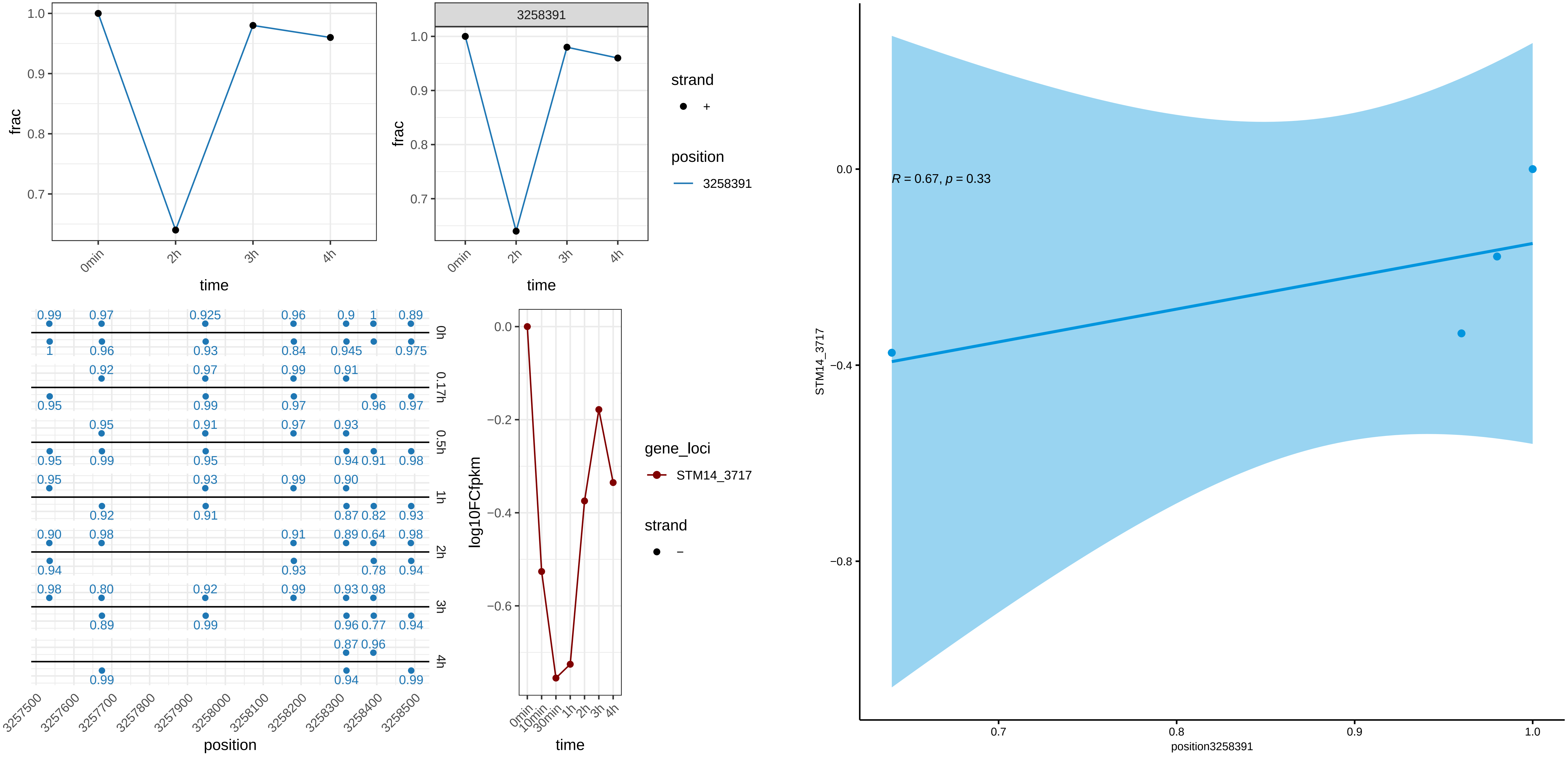
frac
time
0min
2h
3h
4h
3258391
strand
+
position
3258391
position
0h
0.17h
0.5h
1h
2h
3h
4h
log10FCfpkm
10min
30min
1h
gene_loci
STM14_3717
strand
–
R = 0.67, p = 0.33
STM14_3717
position3258391

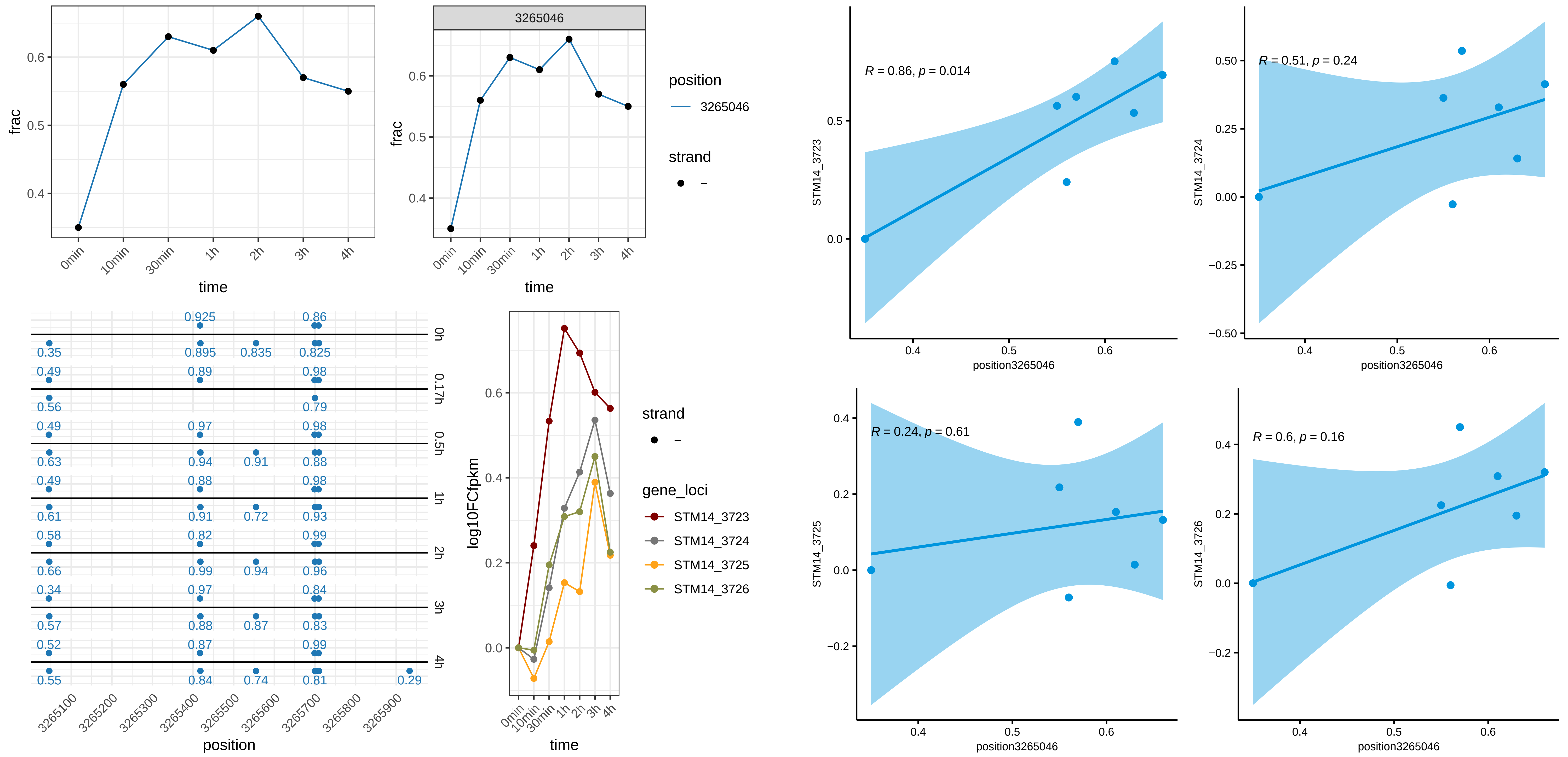

frac
time
0min
10min
30min
1h
2h
3h
4h
3265046
position
strand
–
R = 0.86, p = 0.014
R = 0.51, p = 0.24
R = 0.24, p = 0.61
R = 0.6, p = 0.16
STM14_3723
STM14_3724
STM14_3725
STM14_3726
position3265046
log10FCfpkm
gene_loci
0h
0.17h
0.5h
1h
2h
3h
4h
3265100
3265200
3265300
3265400
3265500
3265600
3265700
3265800
3265900

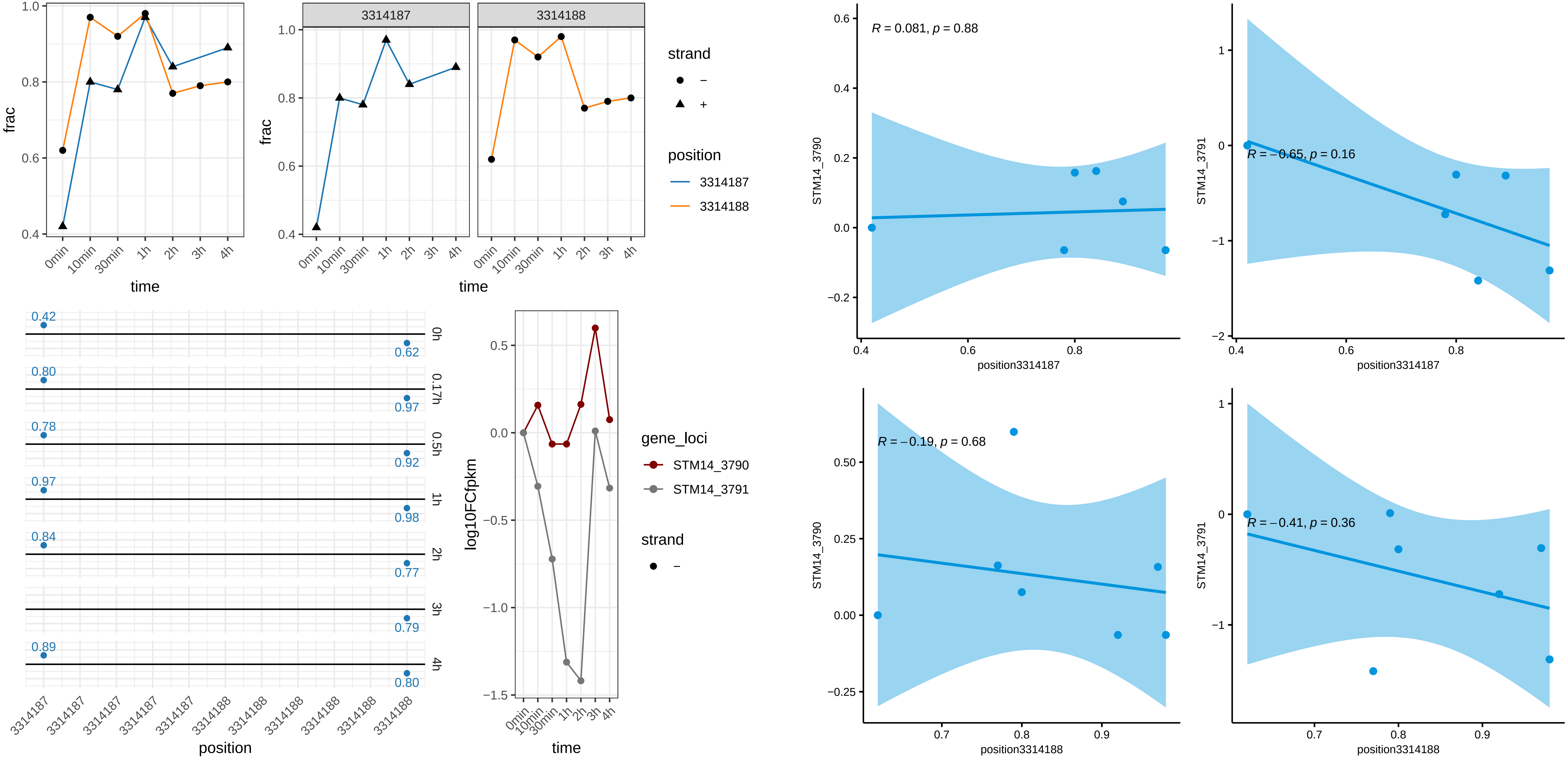
frac
time
0min
10min
30min
1h
2h
3h
4h
3314187
3314188
strand
–
+
position
3314187
3314188
0h
0.17h
0.5h
1h
2h
3h
4h
0.42
0.62
0.80
0.97
0.78
0.92
0.97
0.98
0.84
0.77
0.79
0.89
0.80
log10FCfpkm
gene_loci
STM14_3790
STM14_3791
R = 0.081, p = 0.88
R = −0.65, p = 0.16
R = −0.19, p = 0.68
R = −0.41, p = 0.36
STM14_3790
STM14_3791
position3314187
position3314188

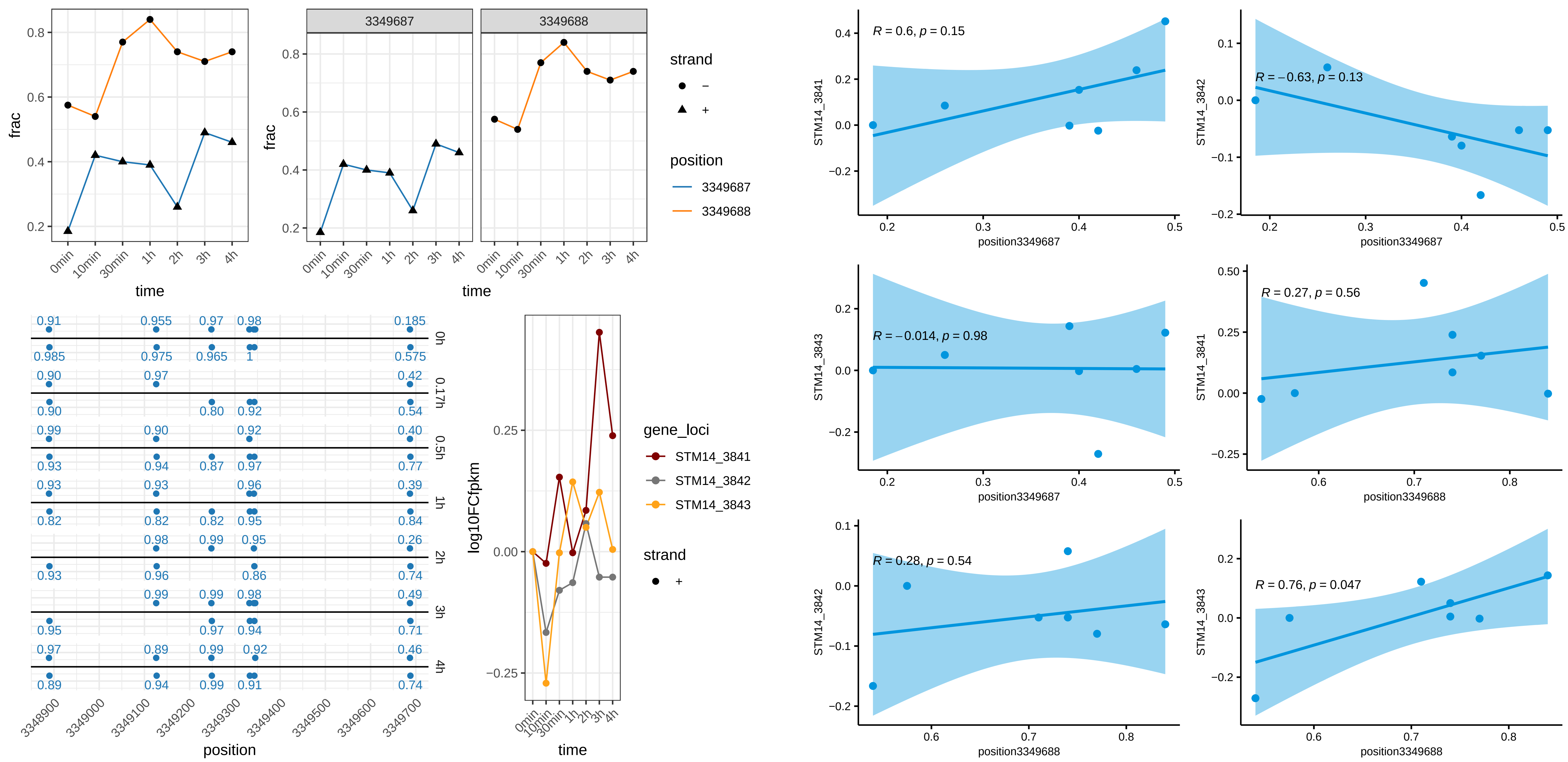

frac
time
3349687
3349688
strand
position
3349687
3349688
position
log10FCfpkm
gene_loci
STM14_3841
STM14_3842
STM14_3843
R = 0.6, p = 0.15
R = −0.63, p = 0.13
R = −0.014, p = 0.98
R = 0.27, p = 0.56
R = 0.28, p = 0.54
R = 0.76, p = 0.047
position3349687
position3349688

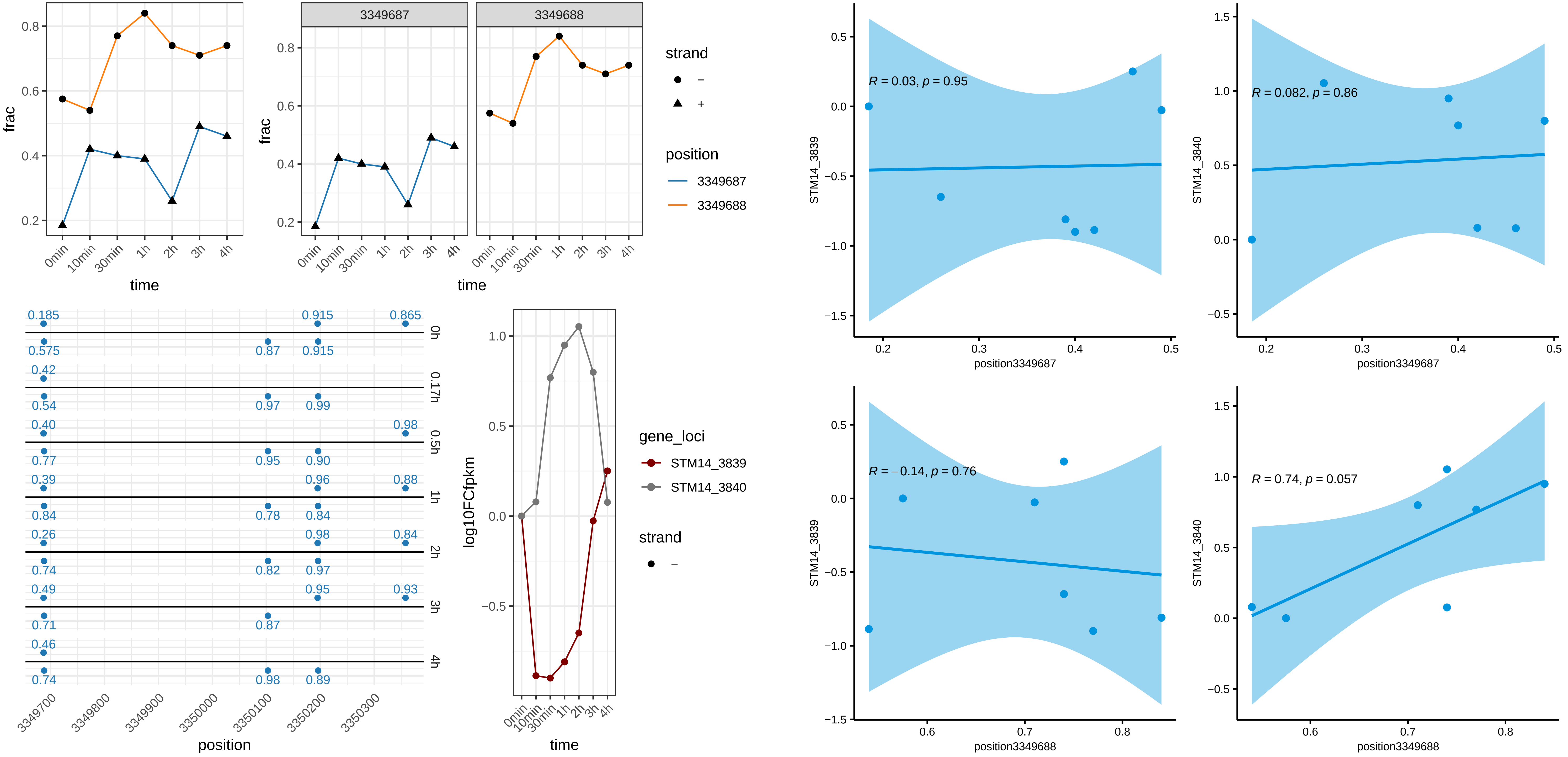

frac
time
0min
10min
30min
1h
2h
3h
4h
3349687
3349688
strand
–
+
position
3349687
3349688
position
0h
0.17h
0.5h
1h
2h
3h
4h
log10FCfpkm
gene_loci
STM14_3839
STM14_3840
strand
–
R = 0.03, p = 0.95
STM14_3839
position3349687
R = 0.082, p = 0.86
STM14_3840
position3349687
R = −0.14, p = 0.76
STM14_3839
position3349688
R = 0.74, p = 0.057
STM14_3840
position3349688

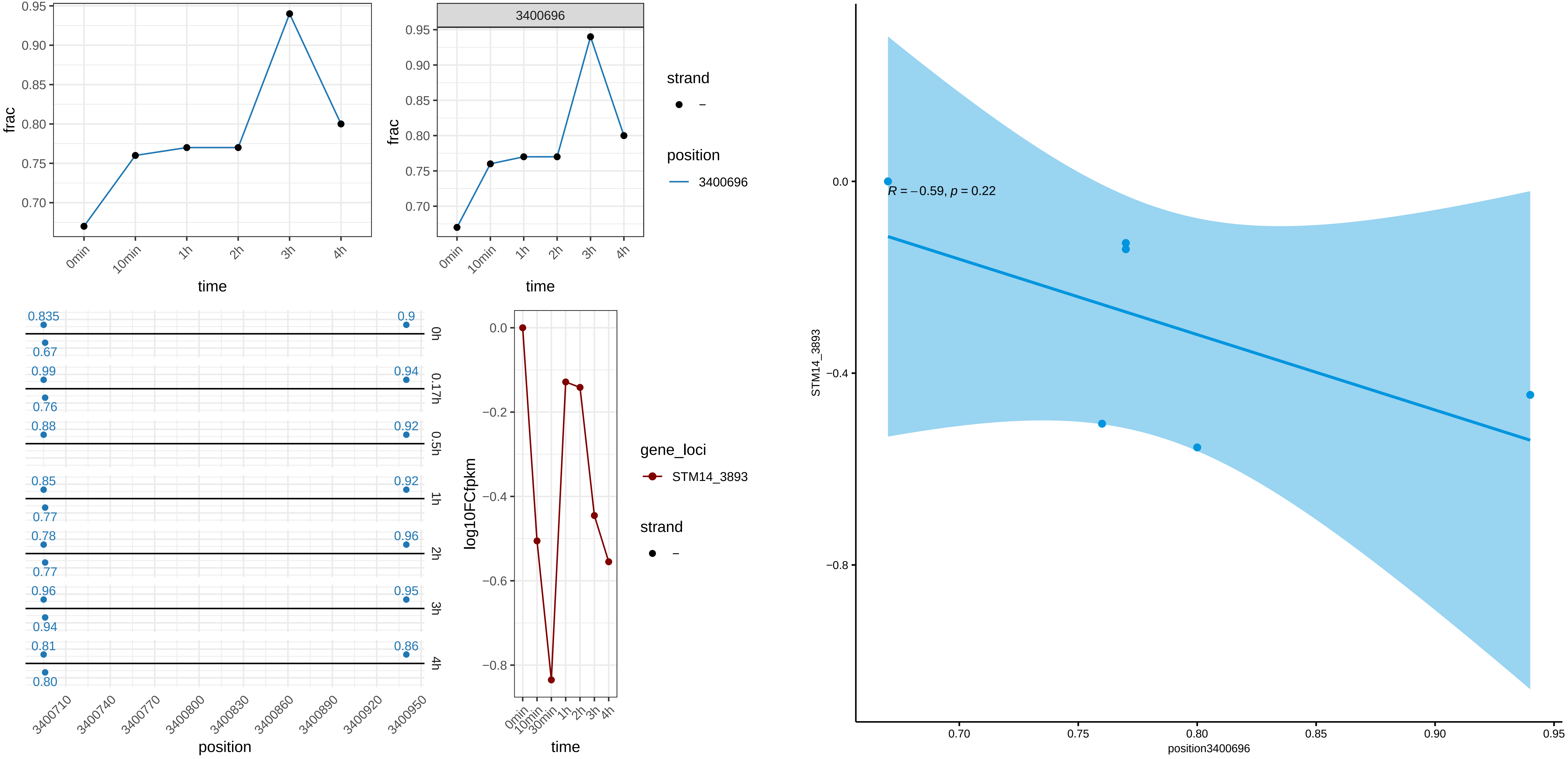

frac
time
0min
10min
1h
2h
3h
4h
3400696
strand
–
position
3400696
0h
0.17h
0.5h
1h
2h
3h
4h
position
log10FCfpkm
gene_loci
STM14_3893
R = −0.59, p = 0.22
STM14_3893
position3400696

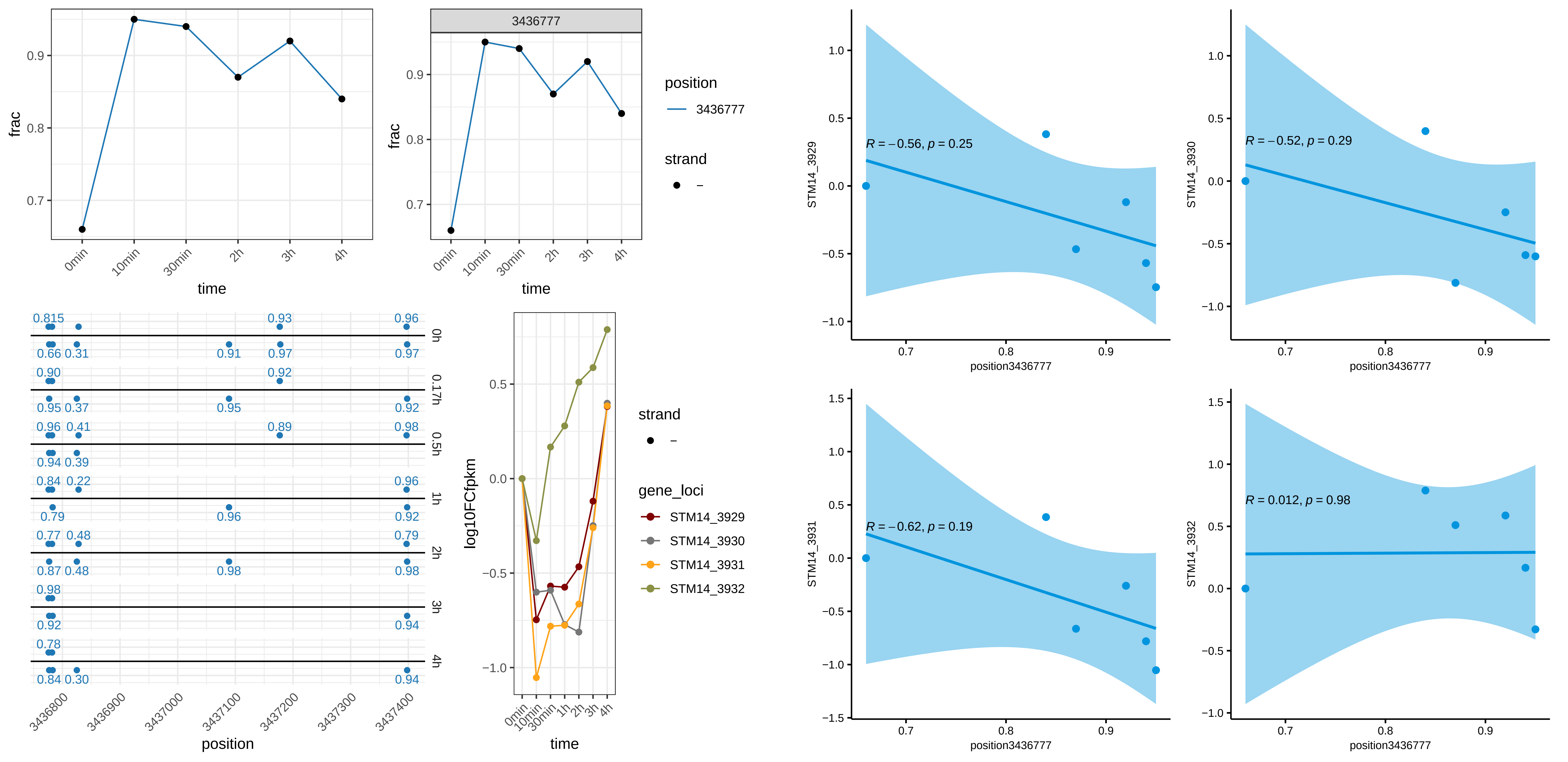

frac
time
0min
10min
30min
2h
3h
4h
3436777
position
strand
–
position
0h
0.17h
0.5h
1h
2h
3h
4h
log10FCfpkm
gene_loci
STM14_3929
STM14_3930
STM14_3931
STM14_3932
STM14_3929
STM14_3930
STM14_3931
STM14_3932
position3436777
R = −0.56, p = 0.25
R = −0.52, p = 0.29
R = −0.62, p = 0.19
R = 0.012, p = 0.98

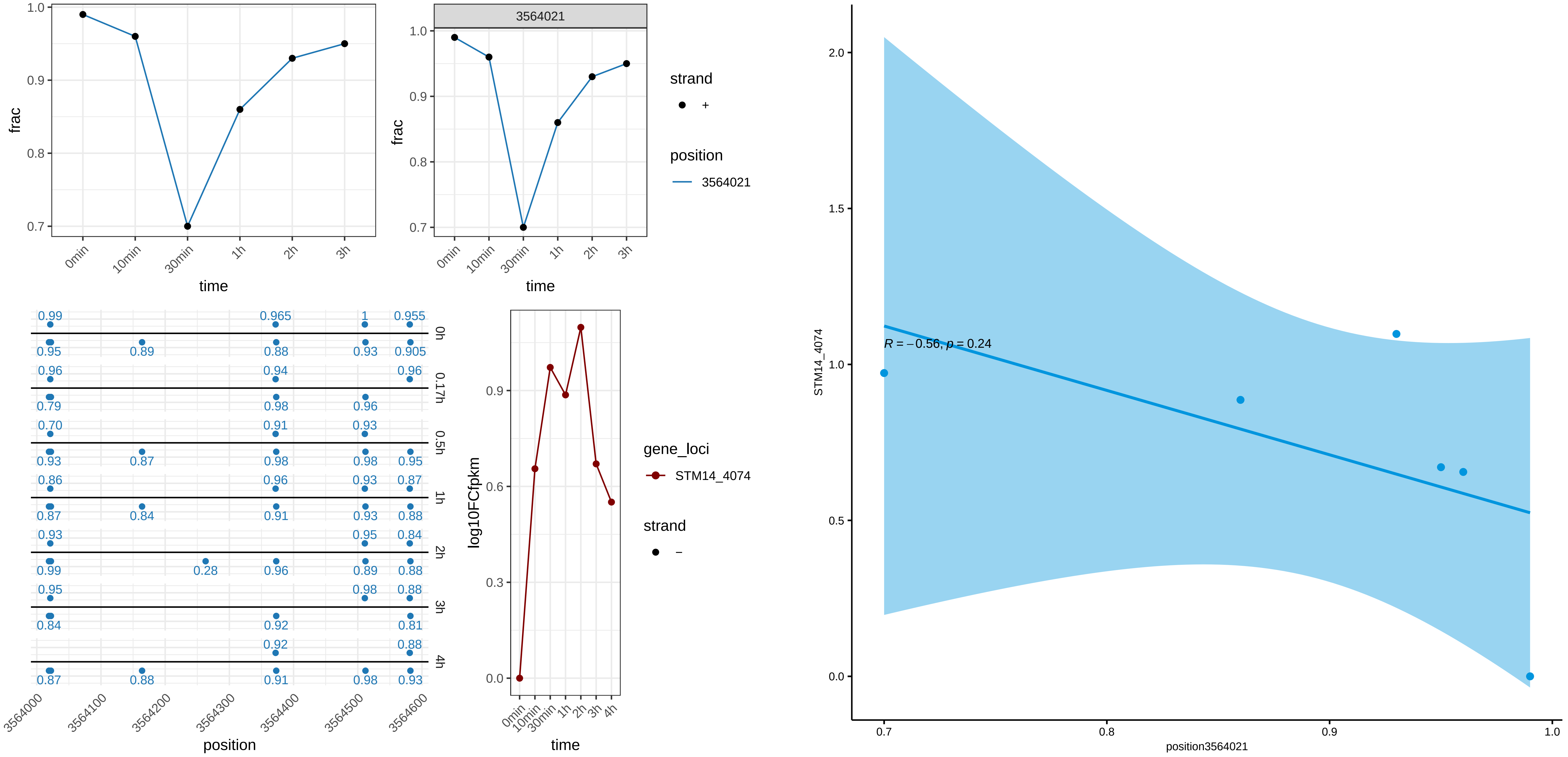

frac
time
0min
10min
30min
1h
2h
3h
3564021
strand
+
position
3564021
log10FCfpkm
4h
gene_loci
STM14_4074
–
0h
0.17h
0.5h
position
STM14_4074
R = −0.56, p = 0.24
position3564021

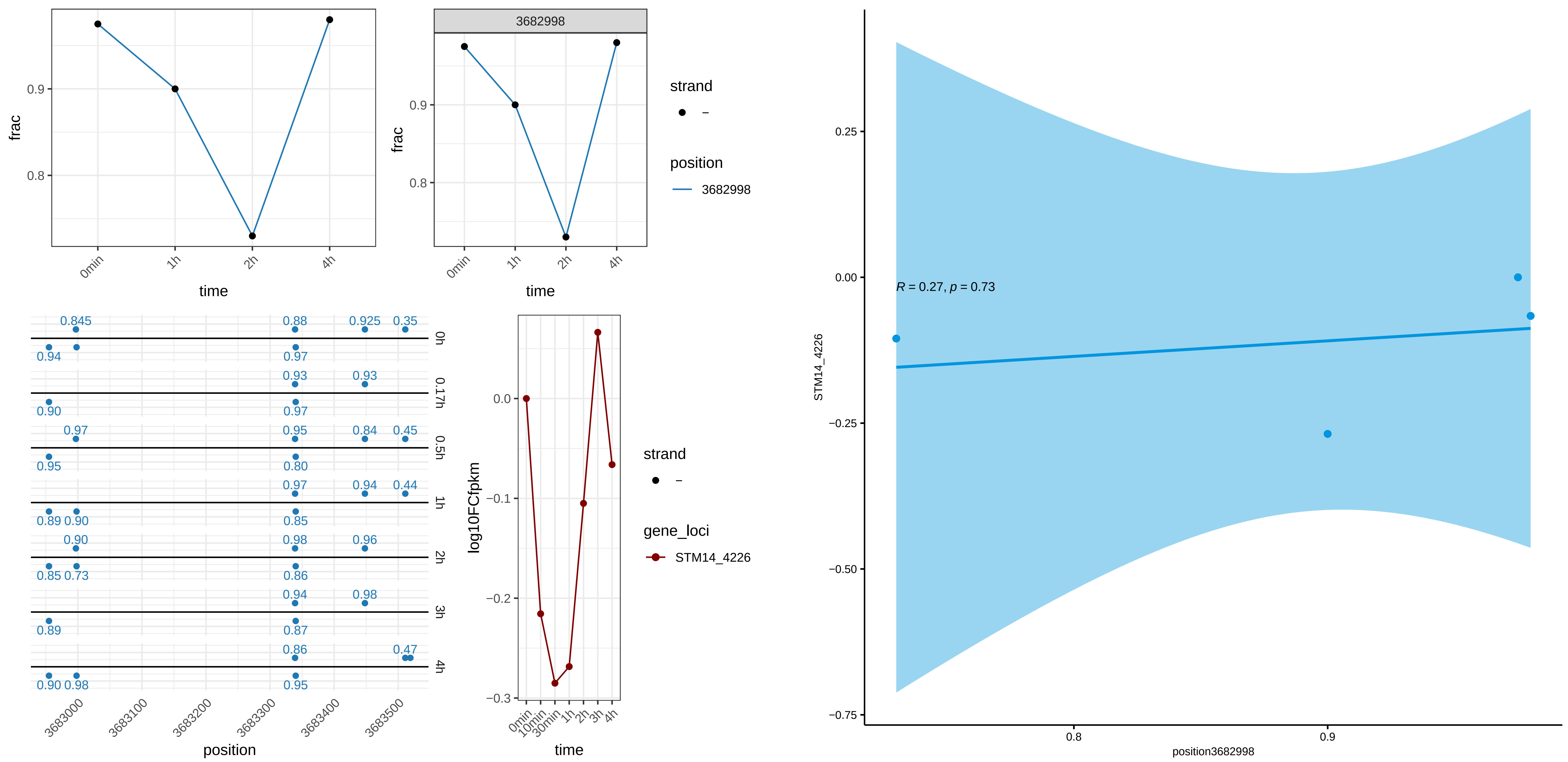

frac
time
0min
1h
2h
4h
3682998
strand
–
position
3682998
0.845
0.88
0.925
0.35
0.94
0.97
0h
0.93
0.93
0.90
0.97
0.17h
0.97
0.95
0.84
0.45
0.95
0.80
0.5h
0.97
0.94
0.44
0.89 0.90
0.85
1h
0.90
0.98
0.96
0.85 0.73
0.86
2h
0.94
0.98
0.89
0.87
3h
0.86
0.47
0.90 0.98
0.95
4h
position
log10FCfpkm
0min
10min
30min
1h
2h
3h
4h
time
strand
–
gene_loci
STM14_4226
R = 0.27, p = 0.73
STM14_4226
position3682998

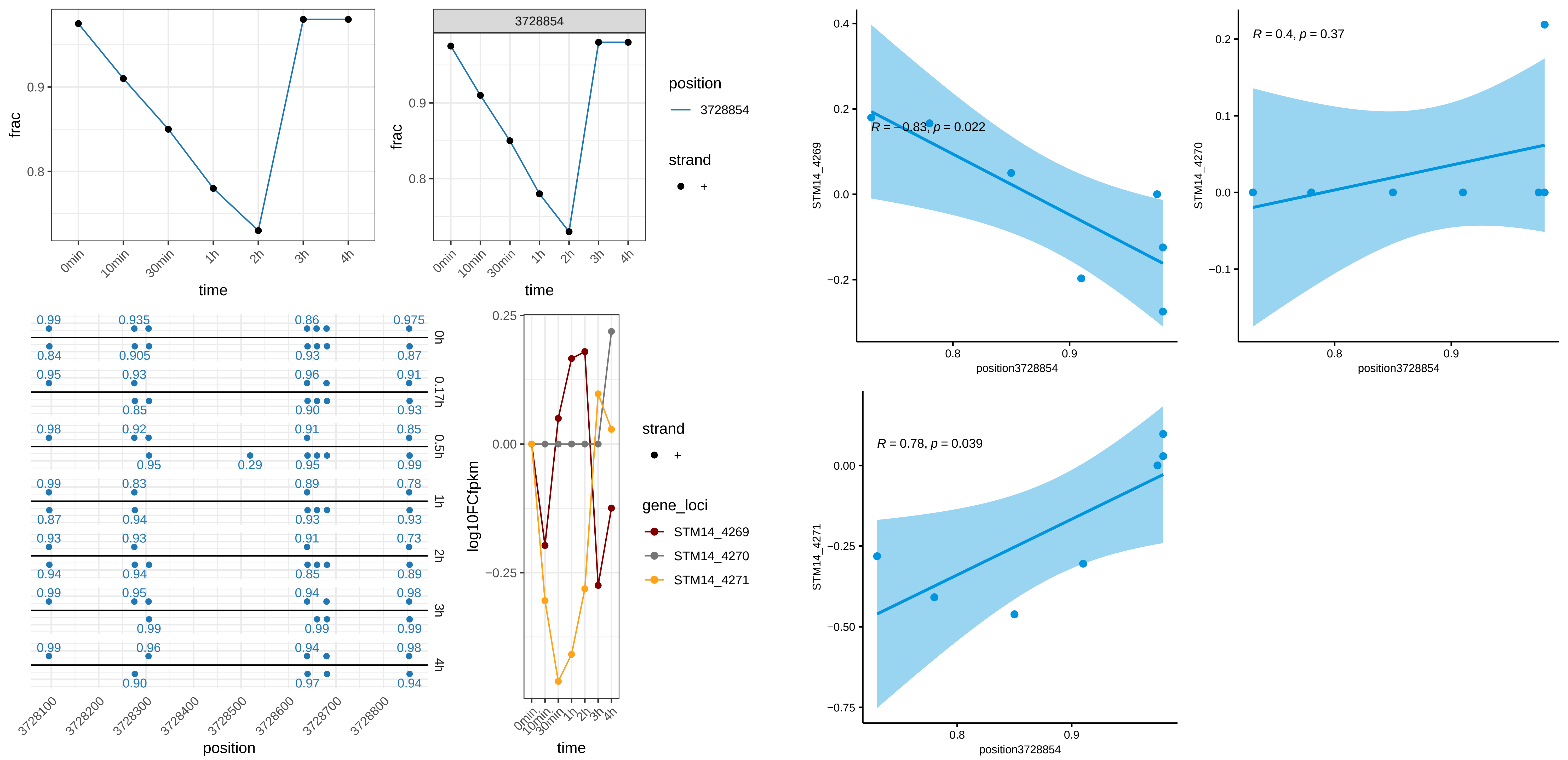

frac
time
0min
10min
30min
1h
2h
3h
4h
3728854
position
strand
+
log10FCfpkm
gene_loci
STM14_4269
STM14_4270
STM14_4271
0h
0.17h
0.5h
position
STM14_4269
STM14_4270
STM14_4271
position3728854
R = −0.83, p = 0.022
R = 0.4, p = 0.37
R = 0.78, p = 0.039

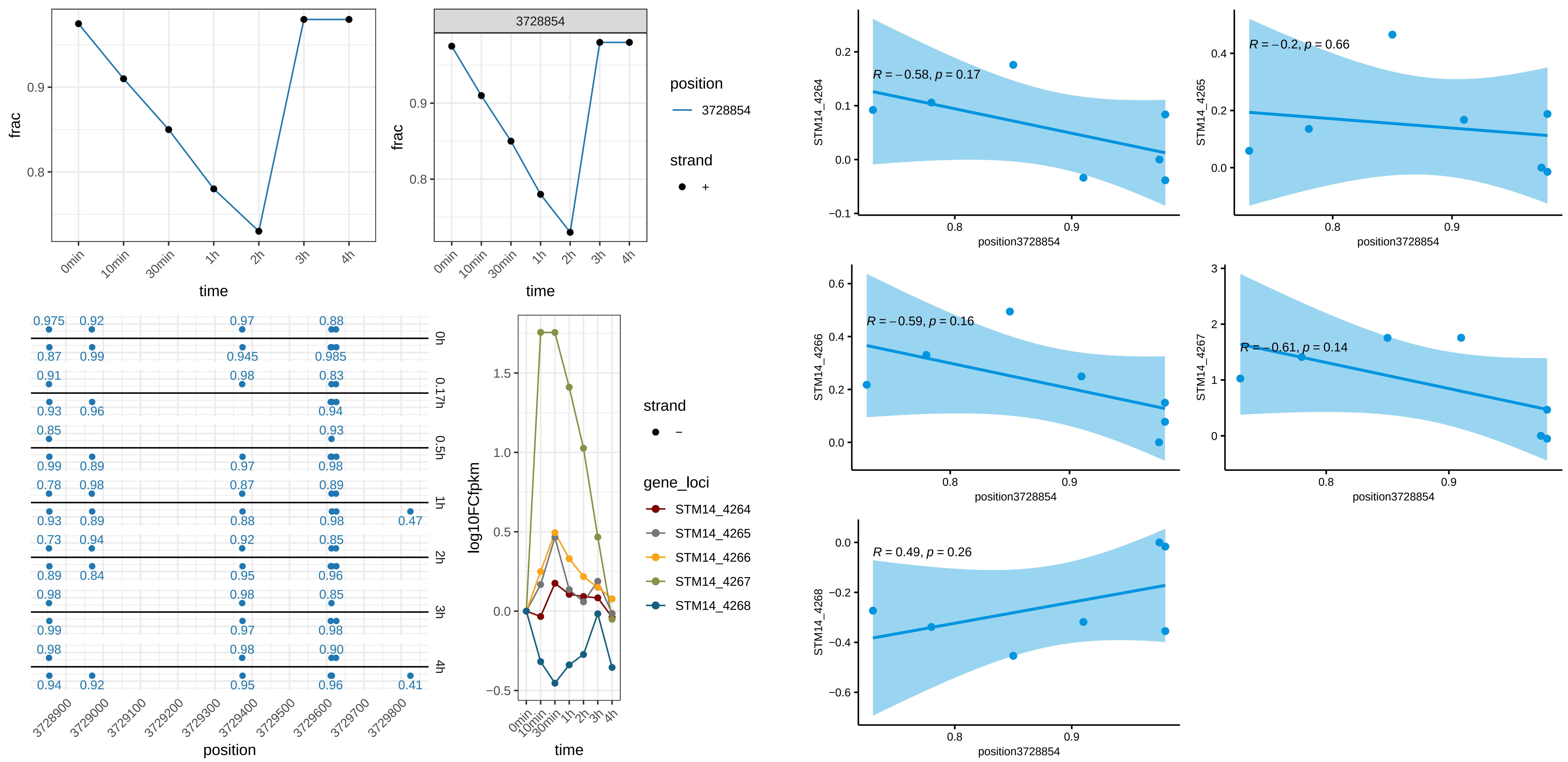

frac
time
3728854
position
strand
gene_loci
STM14_4264
STM14_4265
STM14_4266
STM14_4267
STM14_4268
log10FCfpkm
position3728854
R = −0.58, p = 0.17
R = −0.2, p = 0.66
R = −0.59, p = 0.16
R = −0.61, p = 0.14
R = 0.49, p = 0.26

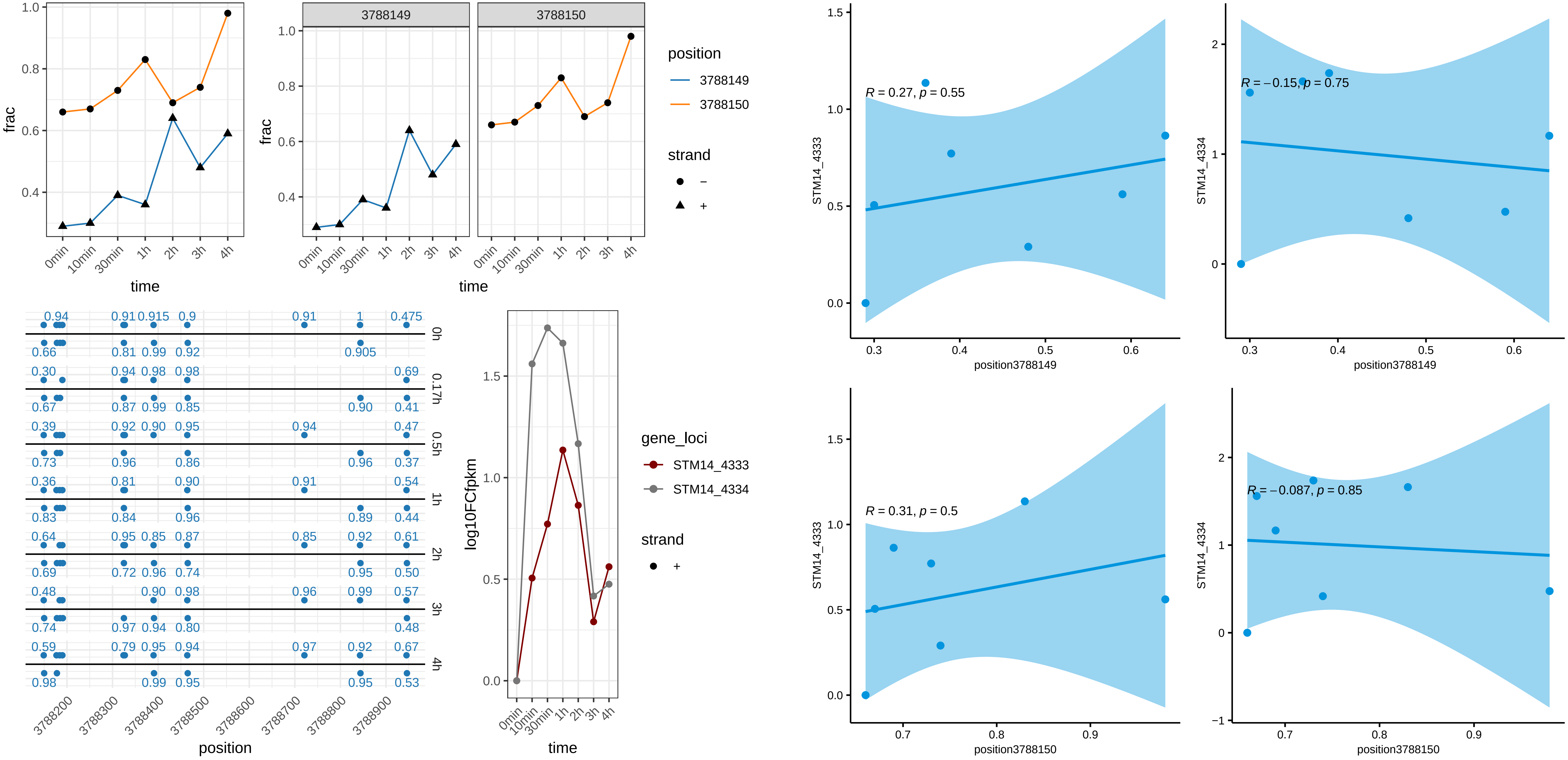

frac
time
0min
10min
30min
1h
2h
3h
4h
3788149
3788150
position
strand
−
+
0.94 0.91 0.915 0.9 0.91 1 0.475
0.66 0.81 0.99 0.92 0.905
0.30 0.94 0.98 0.98 0.69
0.67 0.87 0.99 0.85 0.90 0.41
0.39 0.92 0.90 0.95 0.94 0.47
0.73 0.96 0.86 0.96 0.37
0.36 0.81 0.90 0.91 0.54
0.83 0.84 0.96 0.89 0.44
0.64 0.95 0.85 0.87 0.85 0.92 0.61
0.69 0.72 0.96 0.74 0.95 0.50
0.48 0.90 0.98 0.96 0.99 0.57
0.74 0.97 0.94 0.80 0.48
0.59 0.79 0.95 0.94 0.97 0.92 0.67
0.98 0.99 0.95 0.95 0.53
0h
0.17h
0.5h
1h
2h
3h
4h
3788200
3788300
3788400
3788500
3788600
3788700
3788800
3788900
log10FCfpkm
gene_loci
STM14_4333
STM14_4334
R = 0.27, p = 0.55
STM14_4333
position3788149
R = −0.15, p = 0.75
STM14_4334
position3788149
R = 0.31, p = 0.5
STM14_4333
position3788150
R = −0.087, p = 0.85
STM14_4334
position3788150

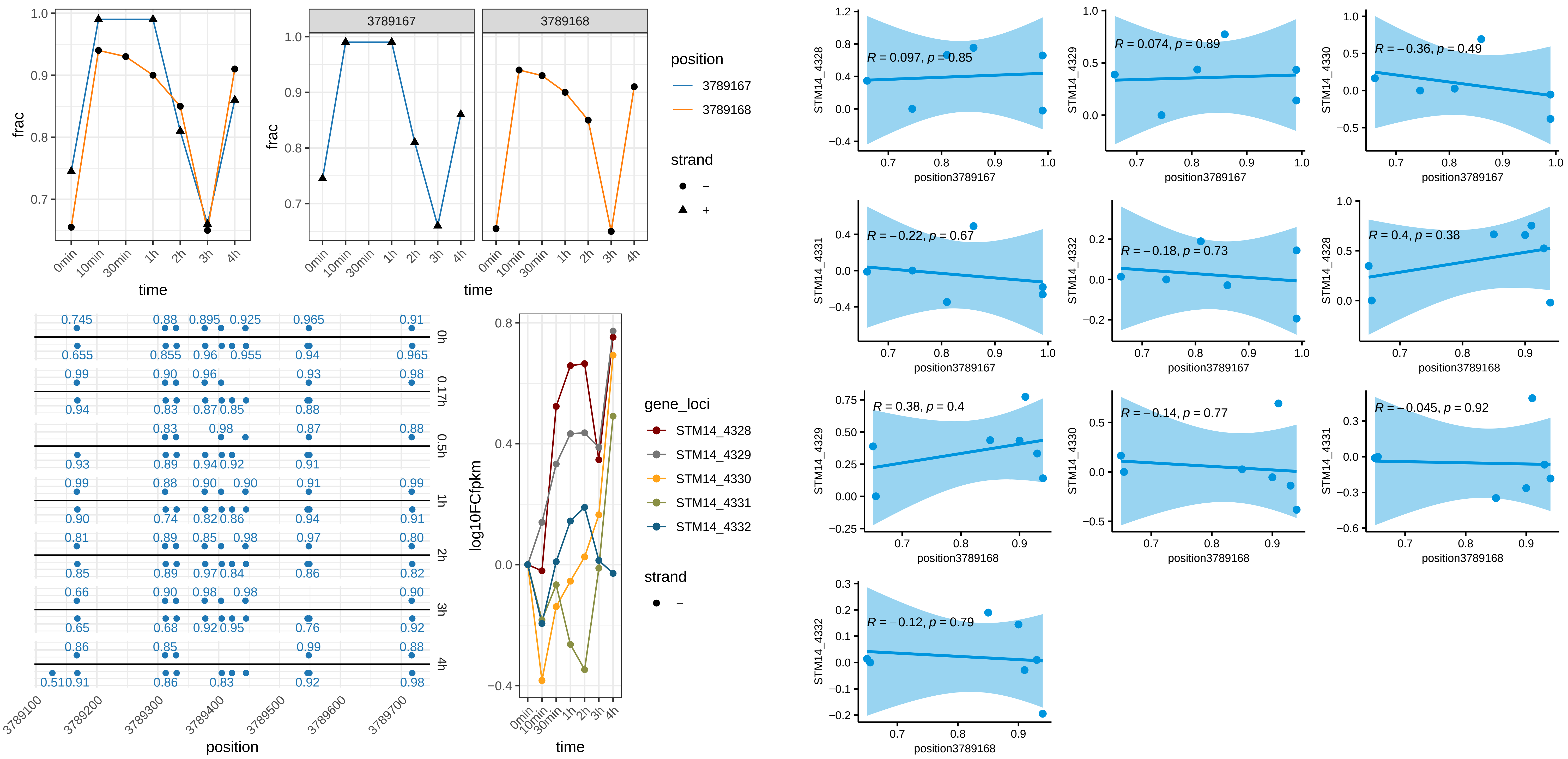

frac
time
0min
10min
30min
1h
2h
3h
4h
3789167
3789168
position
strand
−
+
gene_loci
STM14_4328
STM14_4329
STM14_4330
STM14_4331
STM14_4332
log10FCfpkm
0h
0.17h
0.5h
1h
2h
3h
4h
3789100
3789200
3789300
3789400
3789500
3789600
3789700
R = 0.097, p = 0.85
R = 0.074, p = 0.89
R = −0.36, p = 0.49
R = −0.22, p = 0.67
R = −0.18, p = 0.73
R = 0.4, p = 0.38
R = 0.38, p = 0.4
R = −0.14, p = 0.77
R = −0.045, p = 0.92
R = −0.12, p = 0.79
position3789167
position3789168

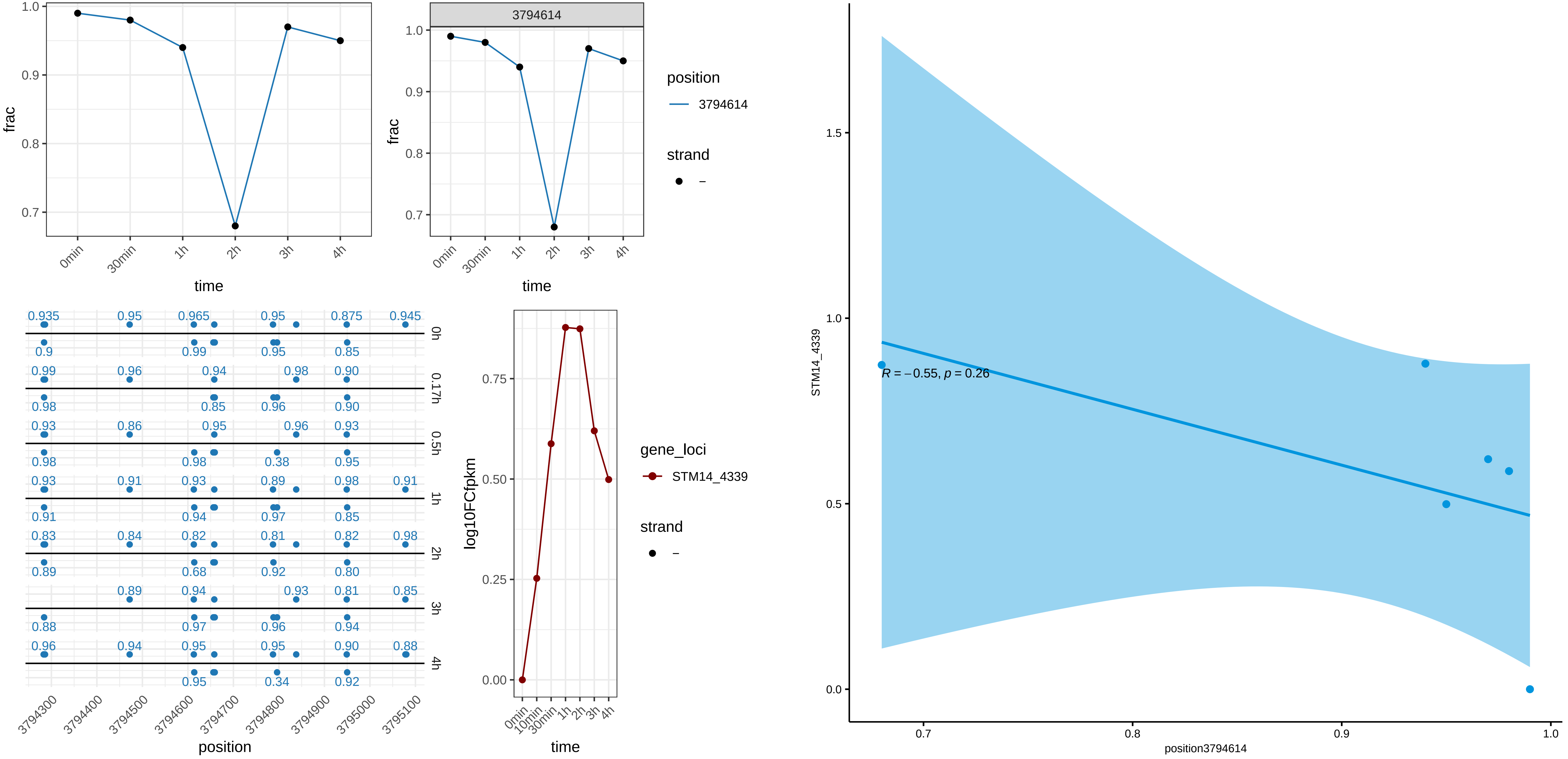
frac
time
3794614
position
strand
–
position
log10FCfpkm
gene_loci
STM14_4339
STM14_4339
R = −0.55, p = 0.26
position3794614

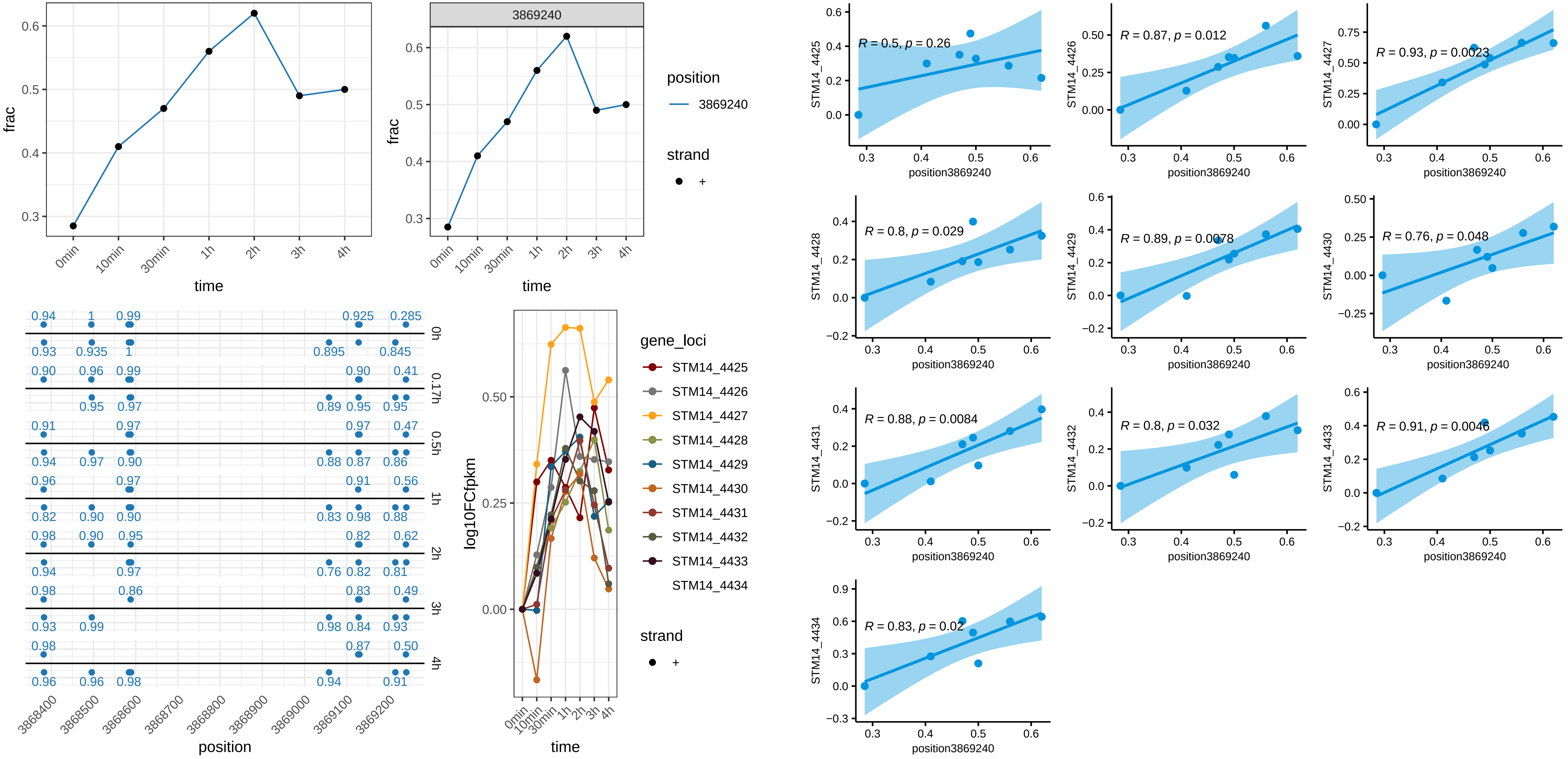

frac
time
0min 10min 30min 1h 2h 3h 4h
3869240
position
3869240
strand
+
position
0h 0.17h 0.5h 1h 2h 3h 4h
3868400 3868500 3868600 3868700 3868800 3868900 3869000 3869100 3869200
log10FCfpkm
gene_loci
STM14_4425
STM14_4426
STM14_4427
STM14_4428
STM14_4429
STM14_4430
STM14_4431
STM14_4432
STM14_4433
STM14_4434
strand
+
position3869240
R = 0.5, p = 0.26
R = 0.87, p = 0.012
R = 0.93, p = 0.0023
R = 0.8, p = 0.029
R = 0.89, p = 0.0078
R = 0.76, p = 0.048
R = 0.88, p = 0.0084
R = 0.8, p = 0.032
R = 0.91, p = 0.0046
R = 0.83, p = 0.02

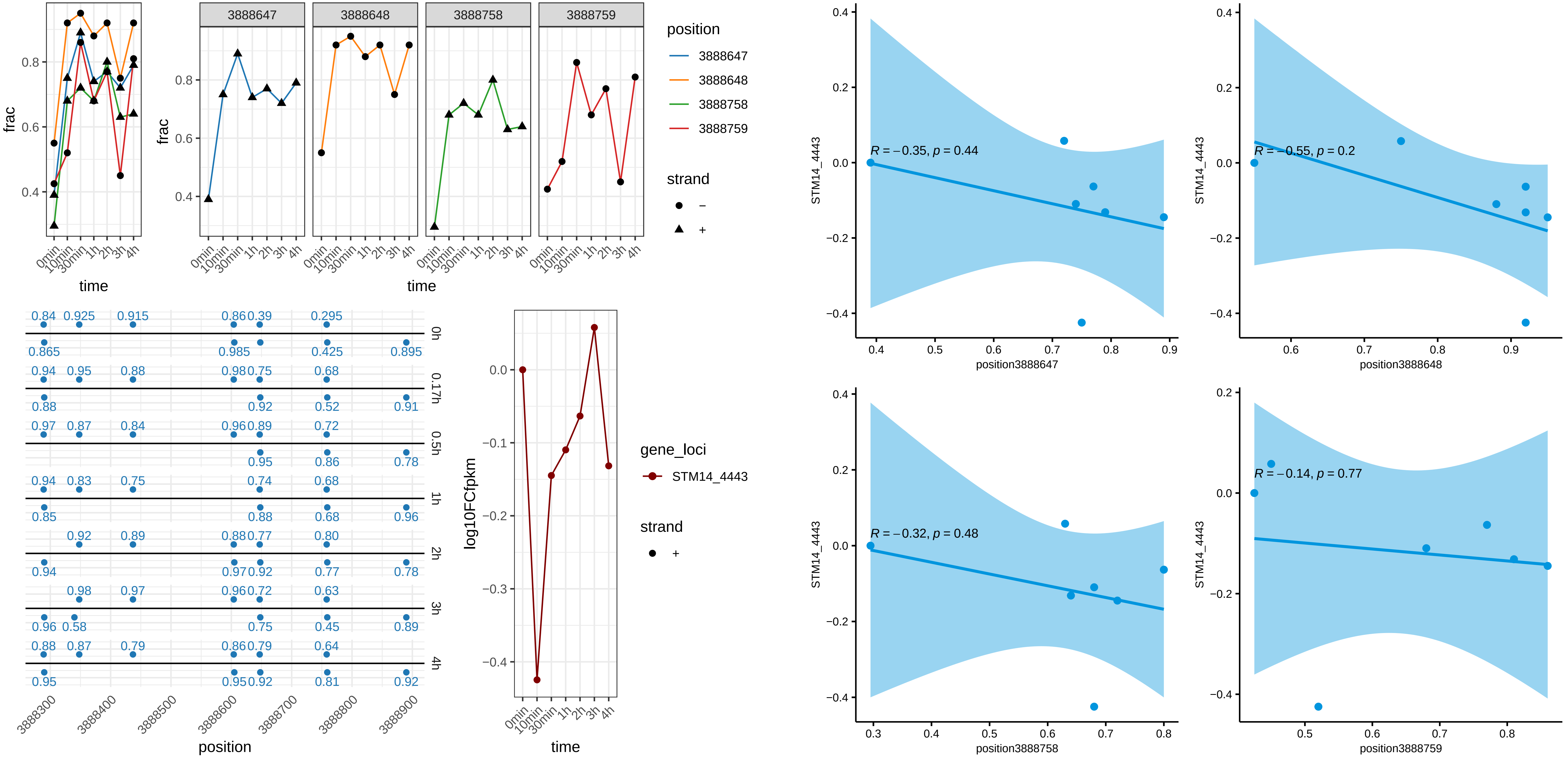
frac
time
0min
10min
30min
1h
2h
3h
4h
3888647
3888648
3888758
3888759
position
strand
–
+
position
log10FCfpkm
gene_loci
STM14_4443
0h
0.17h
0.5h
1h
2h
3h
4h
R = −0.35, p = 0.44
position3888647
STM14_4443
R = −0.55, p = 0.2
position3888648
R = −0.32, p = 0.48
position3888758
R = −0.14, p = 0.77
position3888759

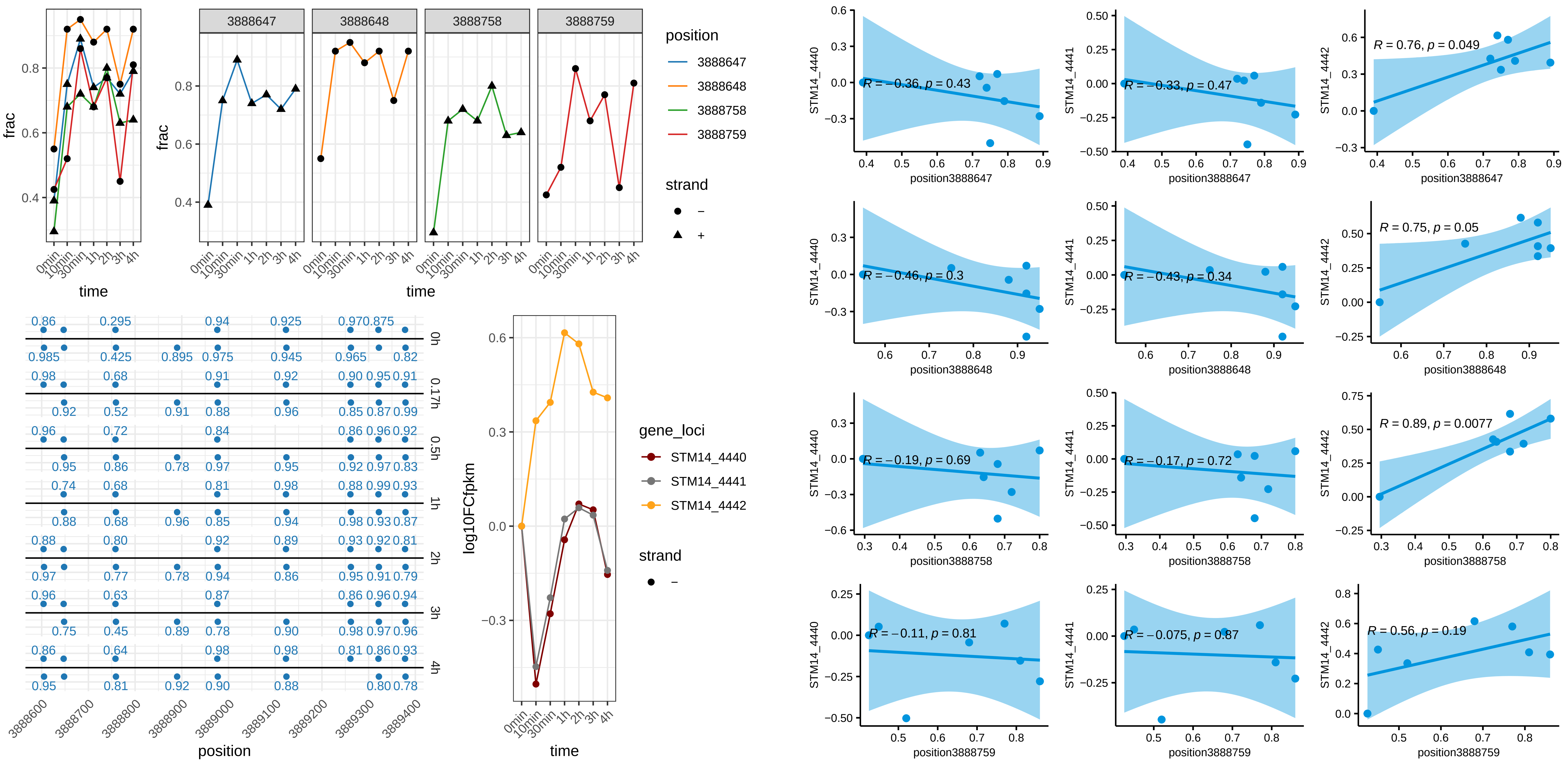

frac
time
3888647
3888648
3888758
3888759
position
strand
log10FCfpkm
gene_loci
STM14_4440
STM14_4441
STM14_4442
0min
10min
30min
1h
2h
3h
4h
0h
0.17h
0.5h
R = −0.36, p = 0.43
R = −0.33, p = 0.47
R = 0.76, p = 0.049
R = −0.46, p = 0.3
R = −0.43, p = 0.34
R = 0.75, p = 0.05
R = −0.19, p = 0.69
R = −0.17, p = 0.72
R = 0.89, p = 0.0077
R = −0.11, p = 0.81
R = −0.075, p = 0.87
R = 0.56, p = 0.19
position3888647
position3888648
position3888758
position3888759

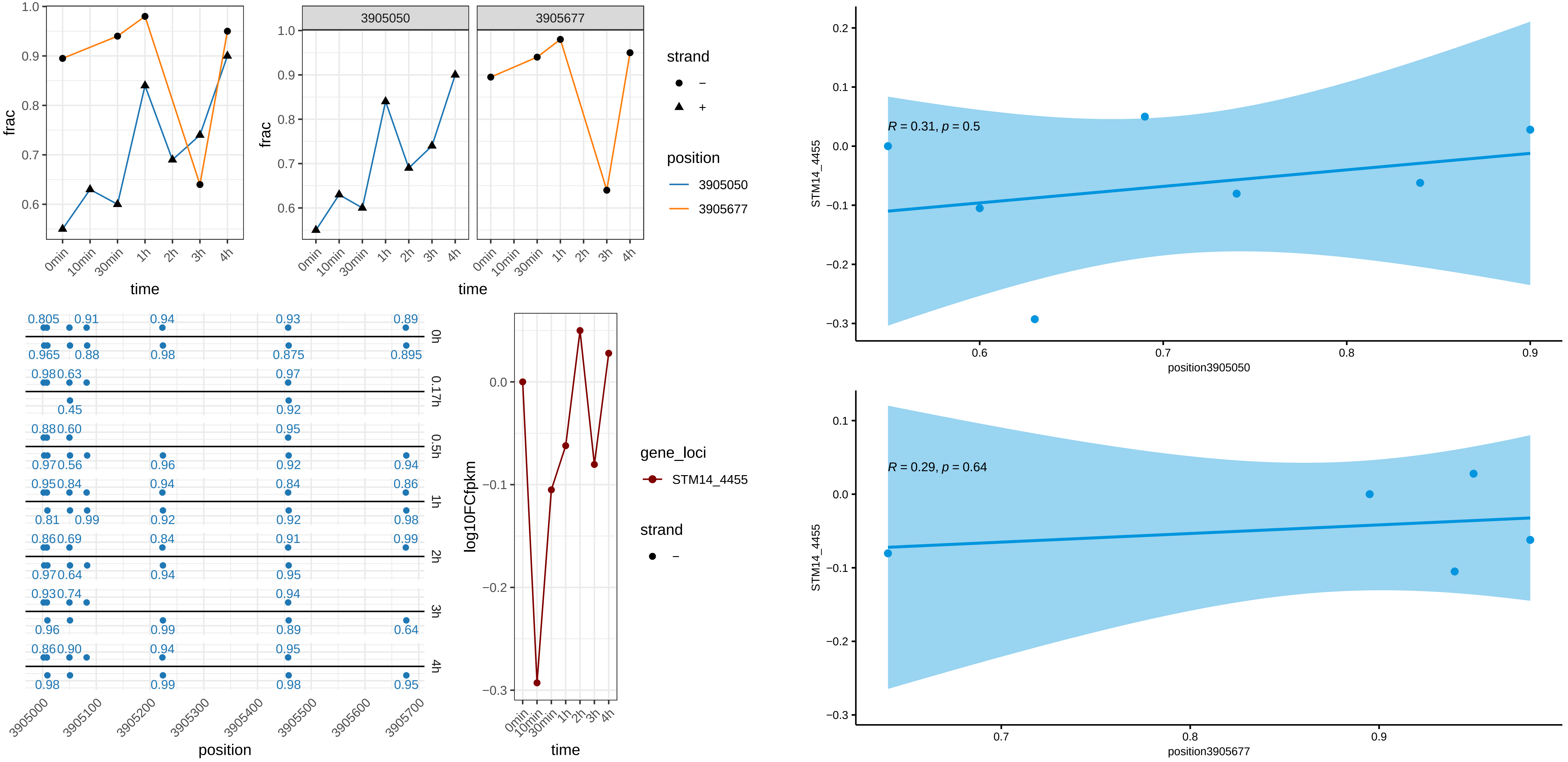

frac
time
0min
10min
30min
1h
2h
3h
4h
3905050
3905677
strand
–
+
position
3905050
3905677
0h
0.17h
0.5h
1h
2h
3h
4h
position
log10FCfpkm
gene_loci
STM14_4455
strand
–
R = 0.31, p = 0.5
STM14_4455
position3905050
R = 0.29, p = 0.64
STM14_4455
position3905677

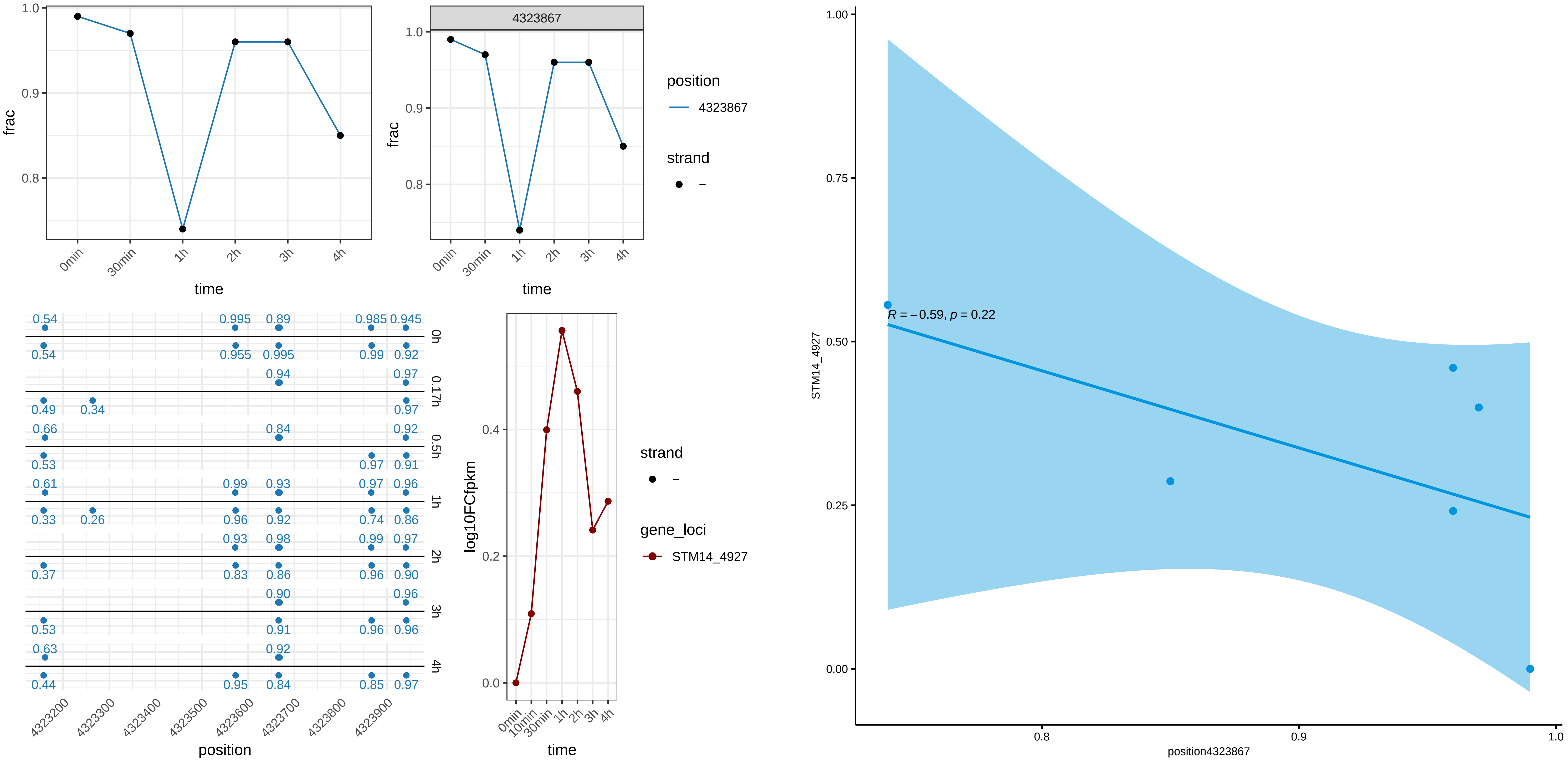

frac
1.0
0.9
0.8
0min
30min
1h
2h
3h
4h
time
4323867
position
4323867
strand
–
0.54 0.995 0.89 0.985 0.945
0.54 0.955 0.995 0.99 0.92
0h
0.94 0.97
0.49 0.34 0.97
0.17h
0.66 0.84 0.92
0.53 0.97 0.91
0.5h
0.61 0.99 0.93 0.97 0.96
0.33 0.26 0.96 0.92 0.74 0.86
1h
0.93 0.98 0.99 0.97
0.37 0.83 0.86 0.96 0.90
2h
0.90 0.96
0.53 0.91 0.96 0.96
3h
0.63 0.92
0.44 0.95 0.84 0.85 0.97
4h
4323200
4323300
4323400
4323500
4323600
4323700
4323800
4323900
position
log10FCfpkm
0.4
0.2
0.0
0min
10min
30min
1h
2h
3h
4h
time
strand
–
gene_loci
STM14_4927
STM14_4927
1.00
0.75
0.50
0.25
0.00
R = –0.59, p = 0.22
0.8
0.9
1.0
position4323867

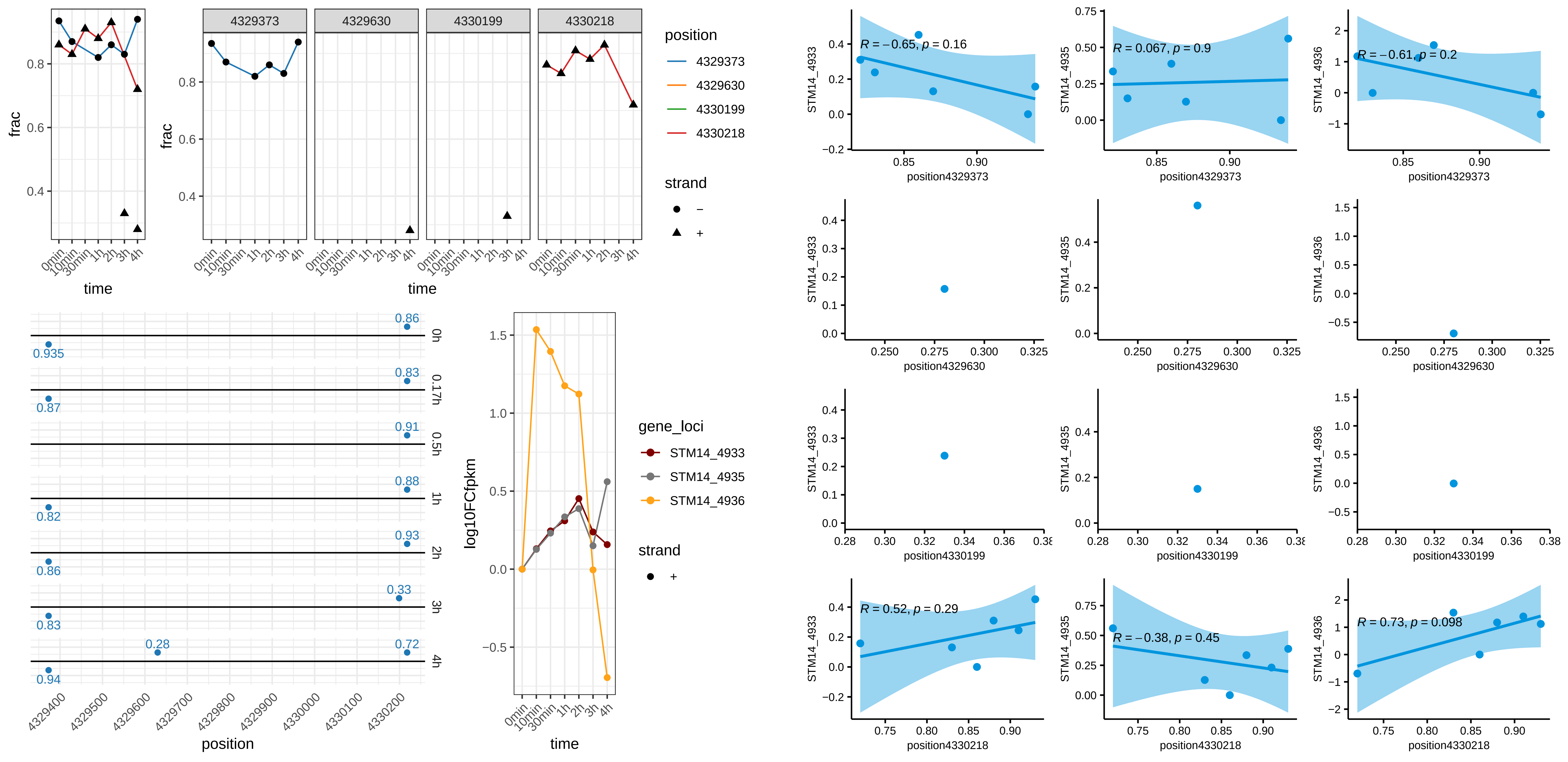

frac
time
4329373
4329630
4330199
4330218
position
strand
0min 10min 30min 1h 2h 3h 4h
0.86
0.935
0.83
0.87
0.91
0.88
0.82
0.93
0.86
0.33
0.83
0.28
0.72
0.94
0h 0.17h 0.5h 1h 2h 3h 4h
position
log10FCfpkm
gene_loci
STM14_4933
STM14_4935
STM14_4936
R = −0.65, p = 0.16
R = 0.067, p = 0.9
R = −0.61, p = 0.2
R = 0.52, p = 0.29
R = −0.38, p = 0.45
R = 0.73, p = 0.098
position4329373
position4329630
position4330199
position4330218

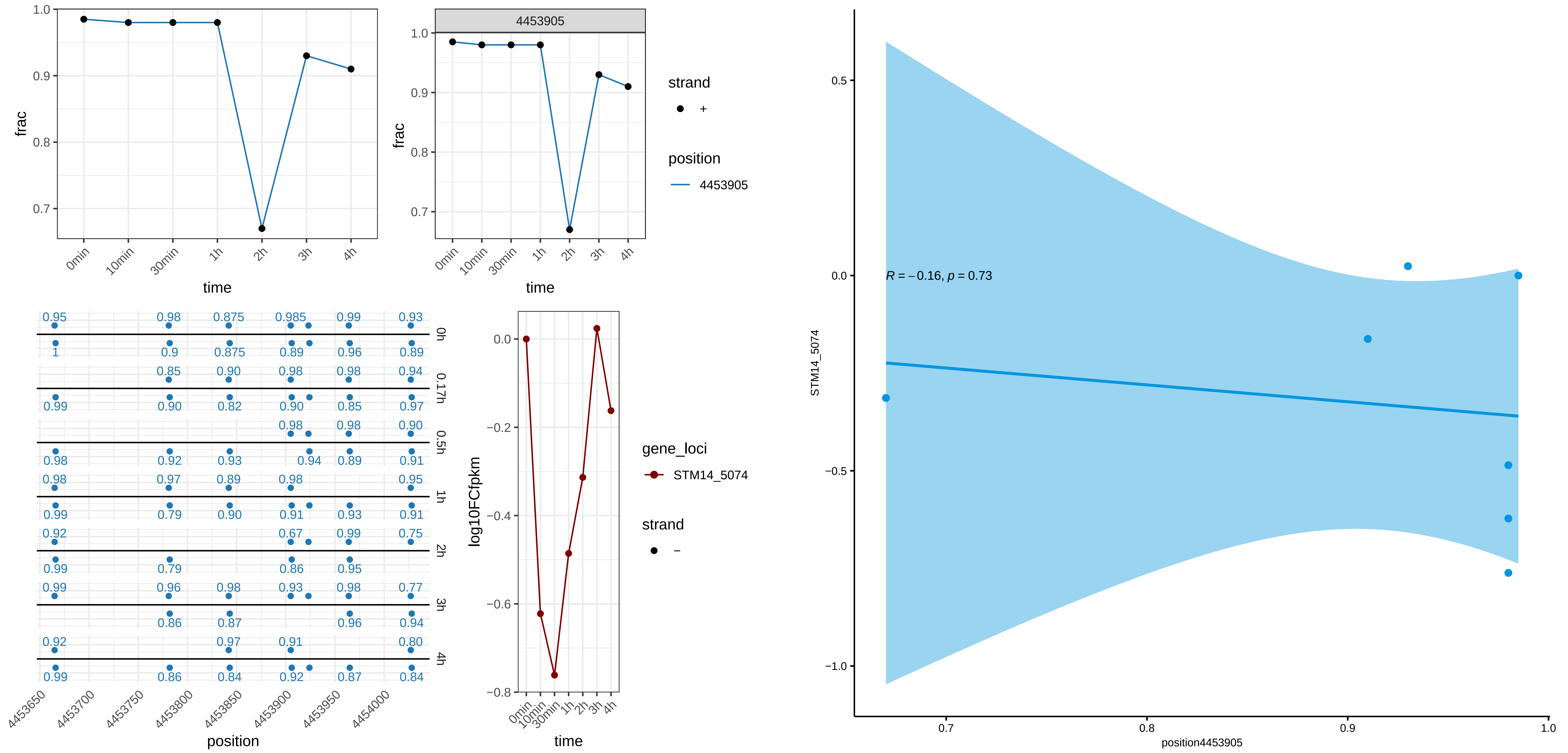

frac
time
0min
10min
30min
1h
2h
3h
4h
4453905
strand
+
position
4453905
0h
0.17h
0.5h
1h
2h
3h
4h
4453650
4453700
4453750
4453800
4453850
4453900
4453950
4454000
position
log10FCfpkm
gene_loci
STM14_5074
strand
–
R = –0.16, p = 0.73
STM14_5074
position4453905

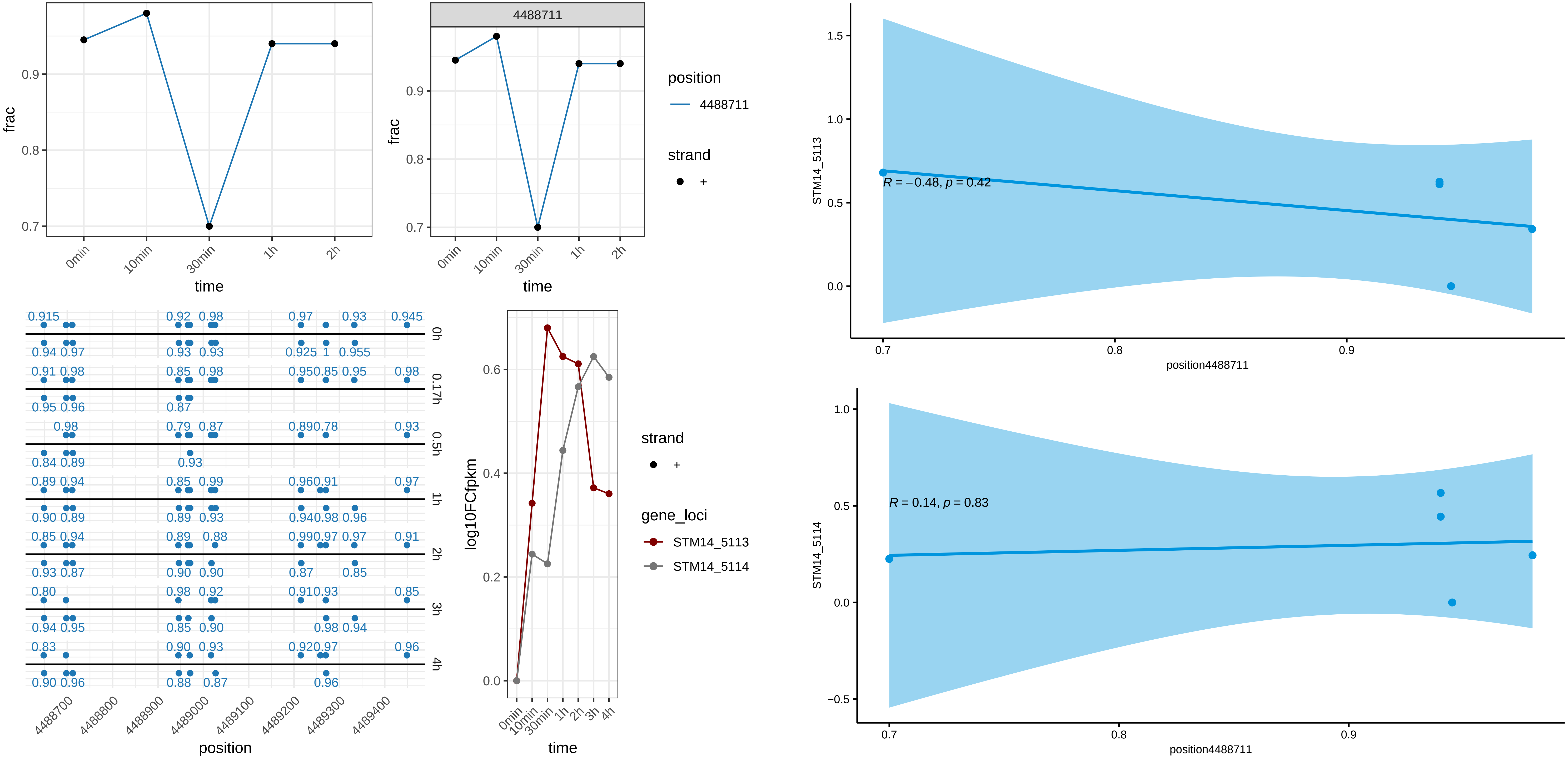

frac
time
0min
10min
30min
1h
2h
4488711
position
strand
+
position
log10FCfpkm
gene_loci
STM14_5113
STM14_5114
0h
0.17h
0.5h
1h
2h
3h
4h
STM14_5113
position4488711
R = −0.48, p = 0.42
STM14_5114
R = 0.14, p = 0.83

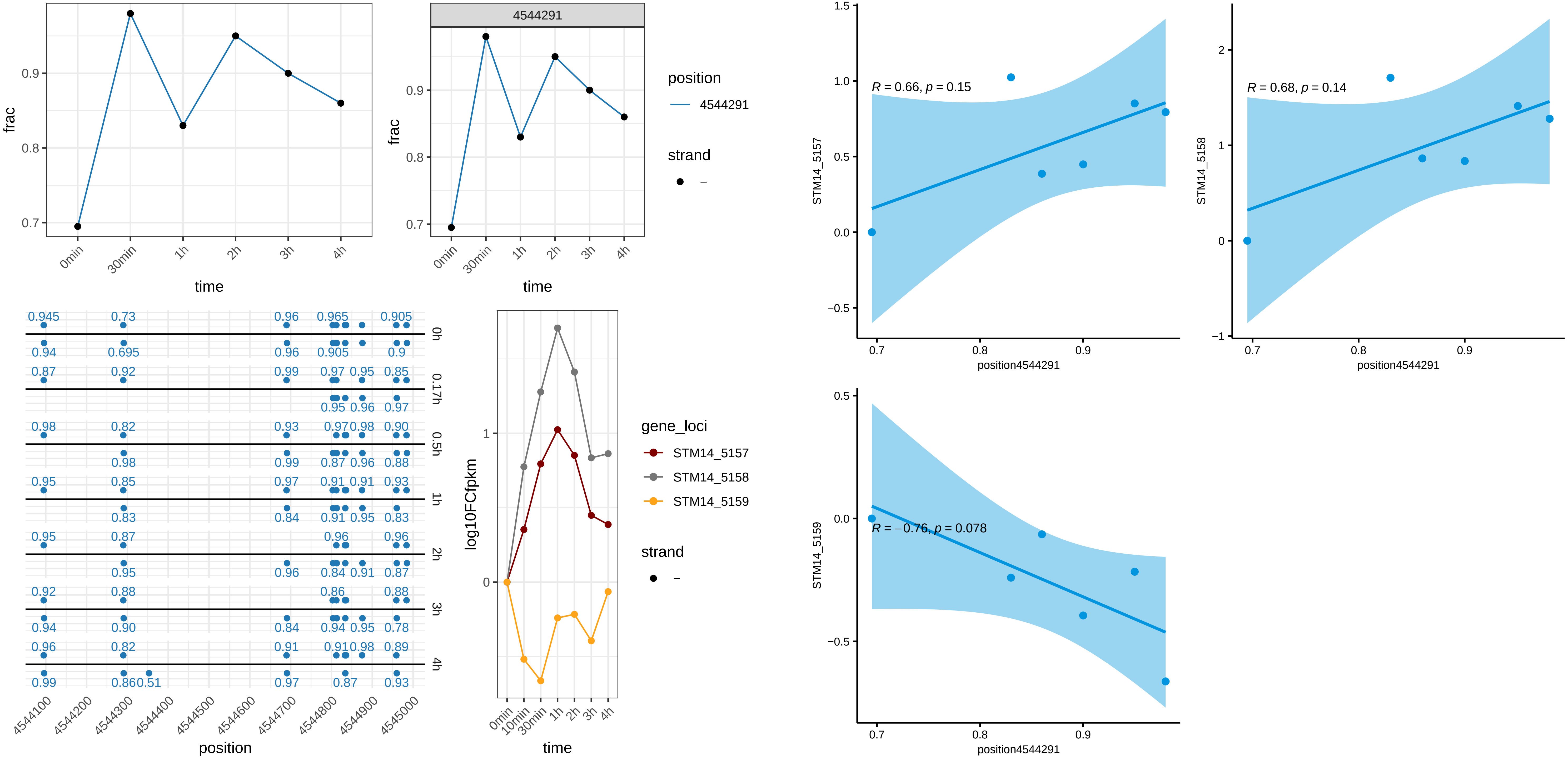
frac
time
0min
30min
1h
2h
3h
4h
4544291
position
strand
position
4544100
4544200
4544300
4544400
4544500
4544600
4544700
4544800
4544900
4545000
0h
0.17h
0.5h
1h
2h
3h
4h
log10FCfpkm
gene_loci
STM14_5157
STM14_5158
STM14_5159
10min
R = 0.66, p = 0.15
R = 0.68, p = 0.14
R = −0.76, p = 0.078
position4544291

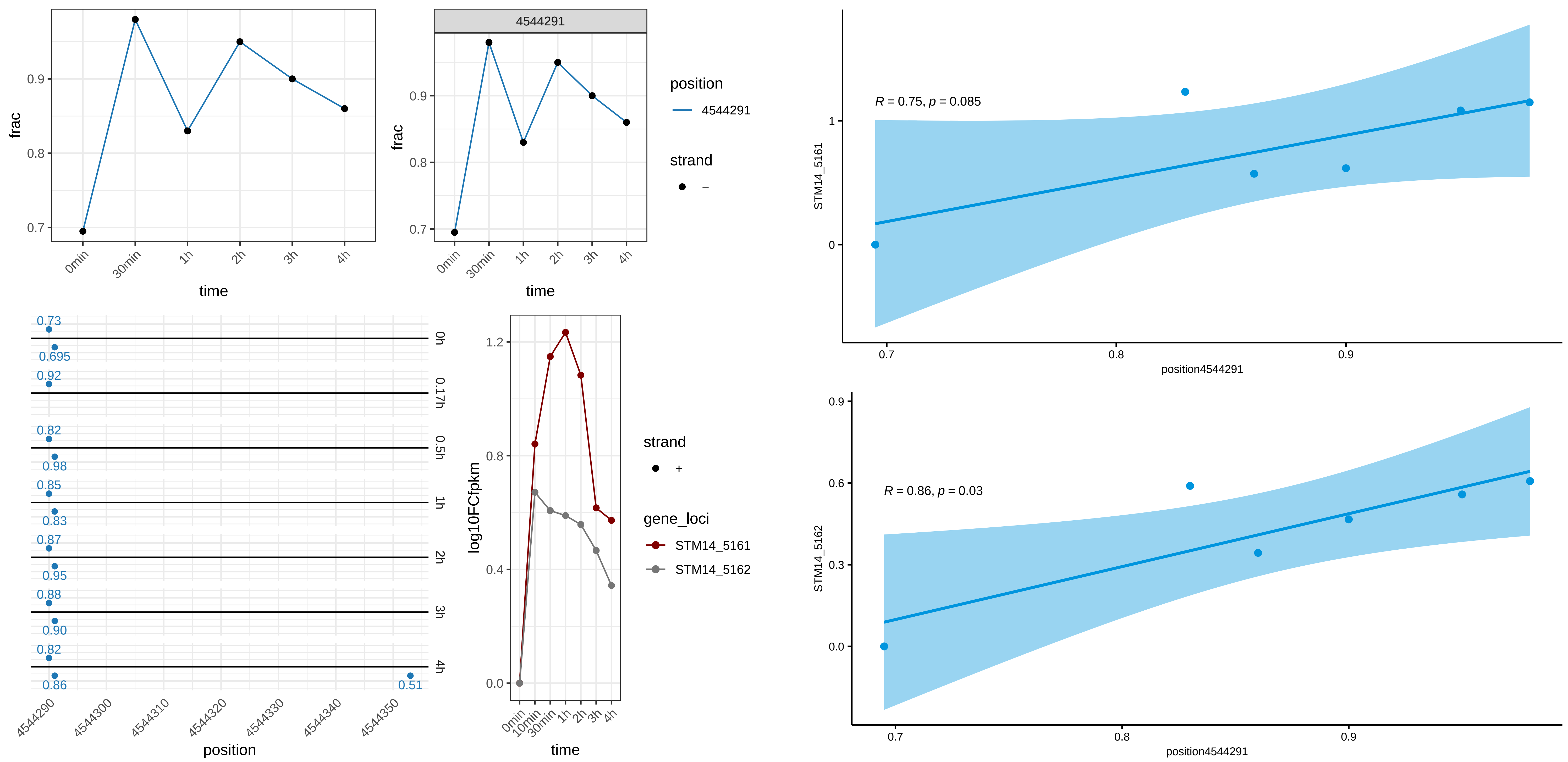

frac
time
4544291
position
4544291
strand
−
R = 0.75, p = 0.085
STM14_5161
position4544291
0.73
0.695
0.92
0.82
0.98
0.85
0.83
0.87
0.95
0.88
0.90
0.82
0.86
0.51
0h
0.17h
0.5h
1h
2h
3h
4h
position
log10FCfpkm
strand
+
gene_loci
STM14_5161
STM14_5162
R = 0.86, p = 0.03
STM14_5162
position4544291

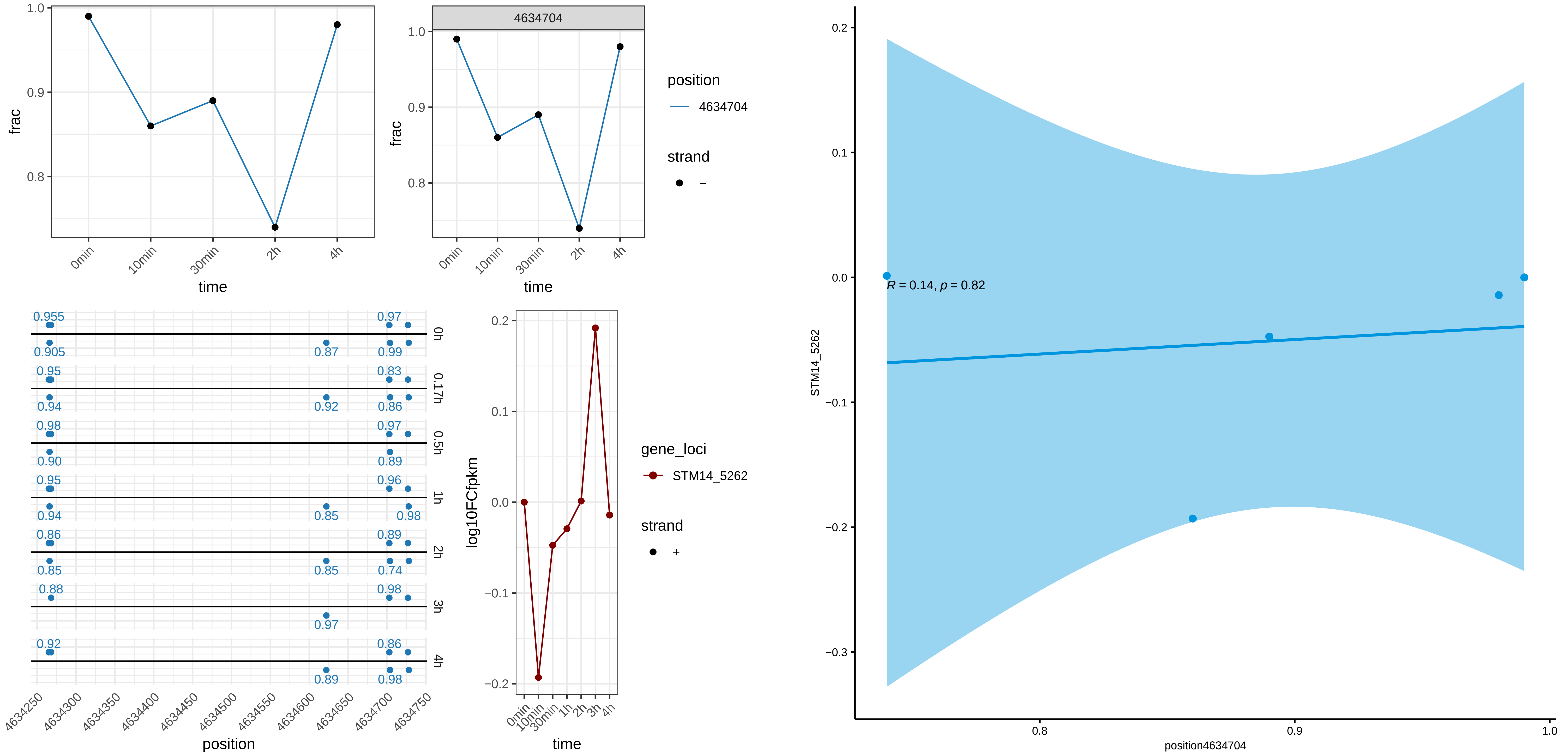

frac
time
0min
10min
30min
2h
4h
4634704
position
strand
gene_loci
STM14_5262
log10FCfpkm
0.17h
0.5h
1h
3h
R = 0.14, p = 0.82
STM14_5262
position4634704

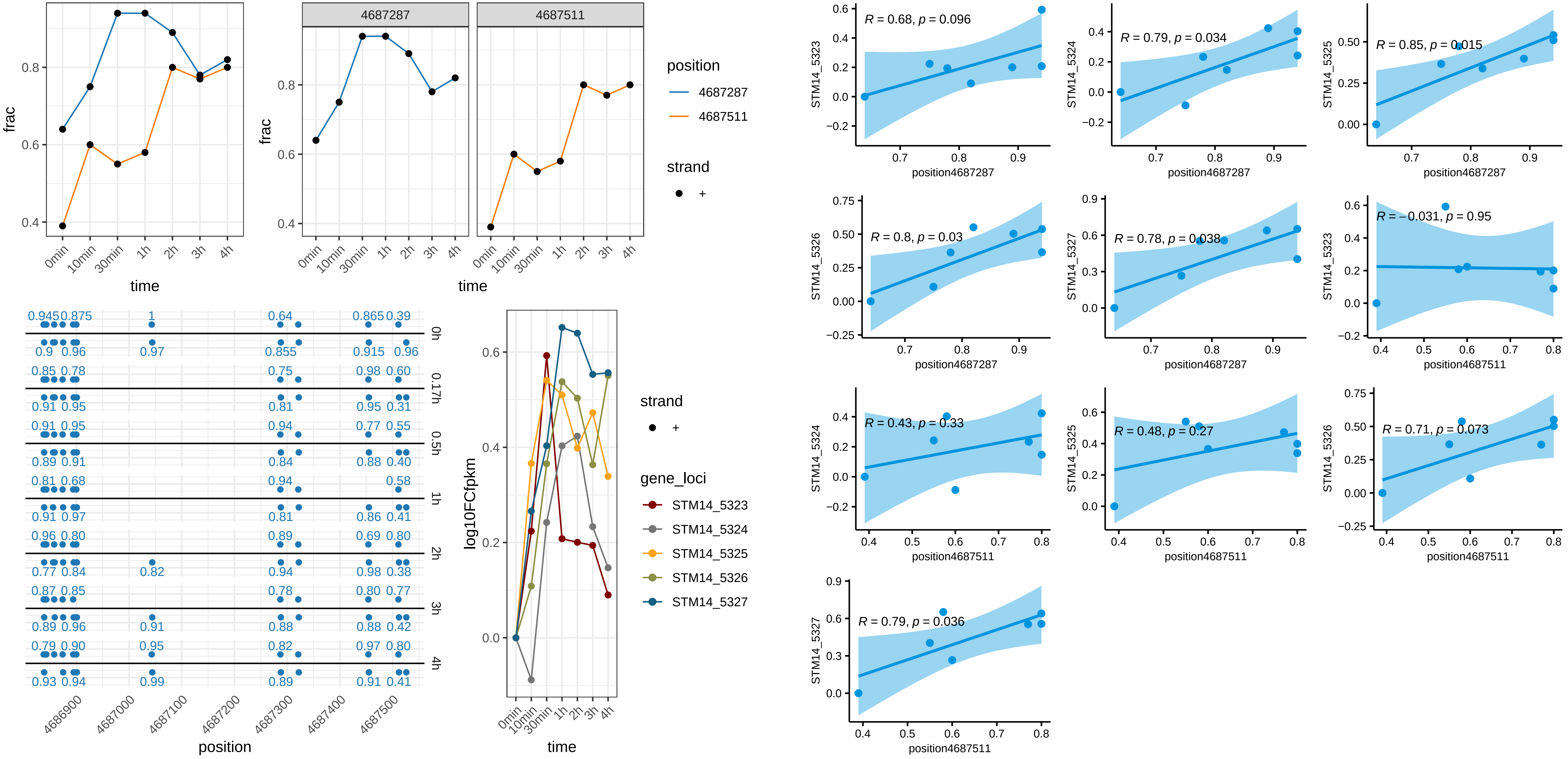

frac
time
4687287
4687511
position
strand
+
gene_loci
STM14_5323
STM14_5324
STM14_5325
STM14_5326
STM14_5327
log10FCfpkm
R = 0.68, p = 0.096
R = 0.79, p = 0.034
R = 0.85, p = 0.015
R = 0.8, p = 0.03
R = 0.78, p = 0.038
R = −0.031, p = 0.95
R = 0.43, p = 0.33
R = 0.48, p = 0.27
R = 0.71, p = 0.073
R = 0.79, p = 0.036
position4687287
position4687511

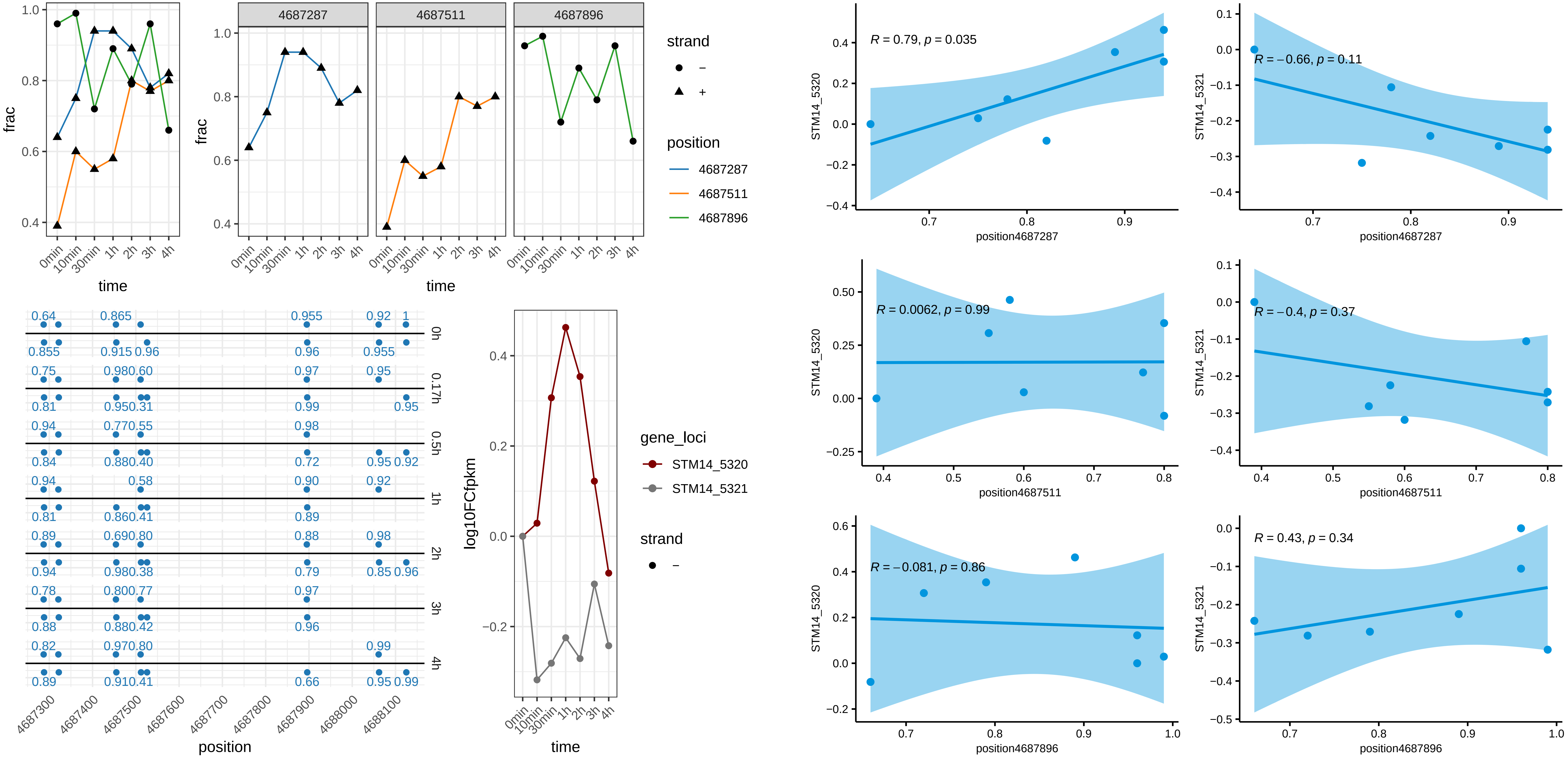

frac
time
4687287
4687511
4687896
strand
–
+
position
4687287
4687511
4687896
gene_loci
STM14_5320
STM14_5321
log10FCfpkm
position
R = 0.79, p = 0.035
R = −0.66, p = 0.11
R = 0.0062, p = 0.99
R = −0.4, p = 0.37
R = −0.081, p = 0.86
R = 0.43, p = 0.34
STM14_5320
STM14_5321
position4687287
position4687511
position4687896

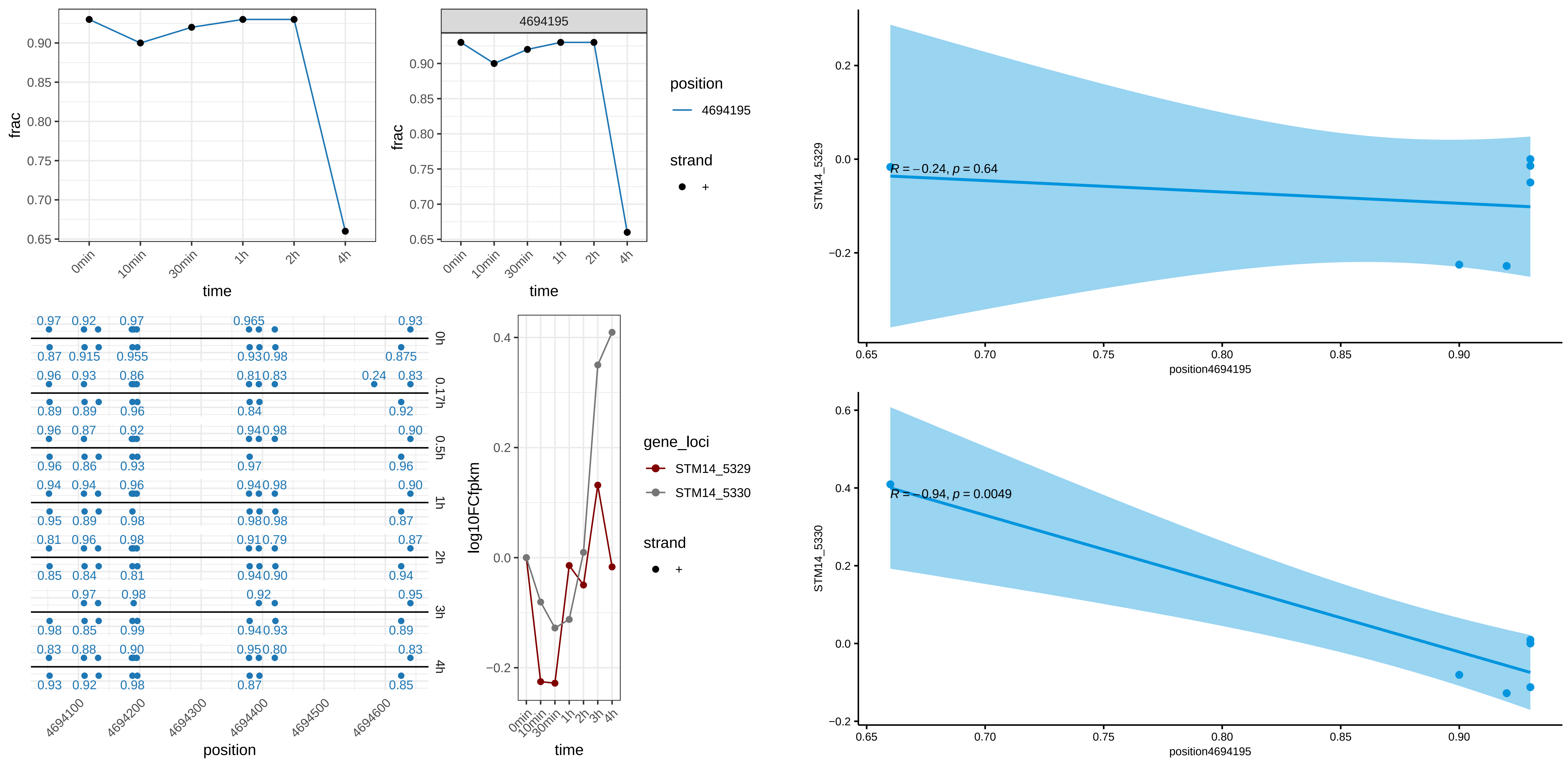

frac
0.90
0.85
0.80
0.75
0.70
0.65
0min
10min
30min
1h
2h
4h
time
4694195
position
4694195
strand
+
0.97 0.92 0.97 0.965 0.93
0.87 0.915 0.955 0.93 0.98 0.875
0h
0.96 0.93 0.86 0.81 0.83 0.24 0.83
0.89 0.89 0.96 0.84 0.92
0.17h
0.96 0.87 0.92 0.94 0.98 0.90
0.96 0.86 0.93 0.97 0.96
0.5h
0.94 0.94 0.96 0.94 0.98 0.90
0.95 0.89 0.98 0.98 0.98 0.87
1h
0.81 0.96 0.98 0.91 0.79 0.87
0.85 0.84 0.81 0.94 0.90 0.94
2h
0.97 0.98 0.92 0.95
0.98 0.85 0.99 0.94 0.93 0.89
3h
0.83 0.88 0.90 0.95 0.80 0.83
0.93 0.92 0.98 0.87 0.85
4h
4694100
4694200
4694300
4694400
4694500
4694600
position
log10FCfpkm
0.4
0.2
0.0
−0.2
0min
10min
30min
1h
2h
3h
4h
time
gene_loci
STM14_5329
STM14_5330
strand
+
STM14_5329
0.2
0.0
−0.2
R = −0.24, p = 0.64
0.65
0.70
0.75
0.80
0.85
0.90
position4694195
STM14_5330
0.6
0.4
0.2
0.0
−0.2
R = −0.94, p = 0.0049
0.65
0.70
0.75
0.80
0.85
0.90
position4694195

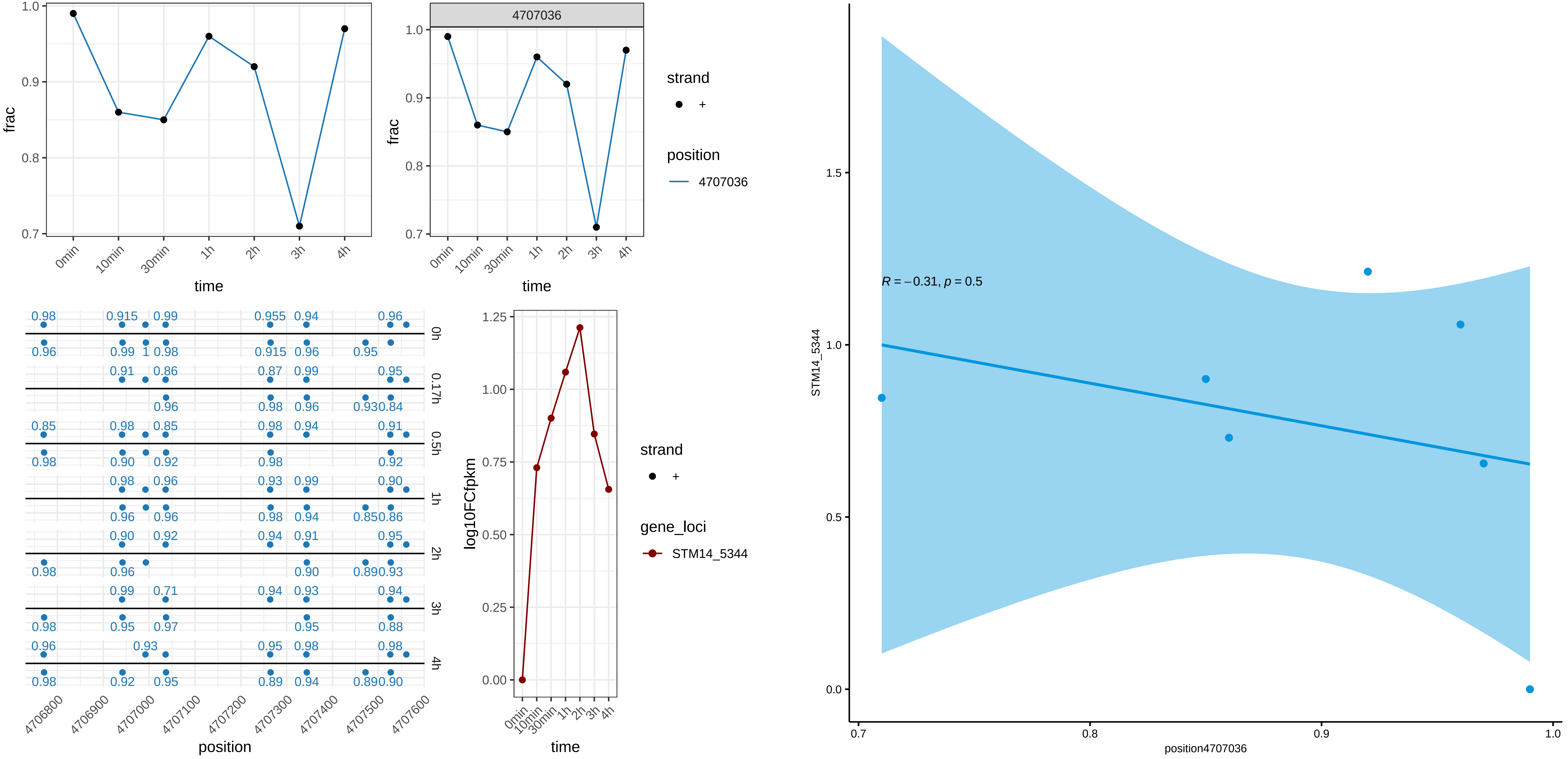
frac
time
0min
10min
30min
1h
2h
3h
4h
4707036
strand
+
position
4707036
position
log10FCfpkm
gene_loci
STM14_5344
0h
0.17h
0.5h
1h
2h
3h
4h
4706800
4706900
4707000
4707100
4707200
4707300
4707400
4707500
4707600
R = −0.31, p = 0.5
STM14_5344
position4707036

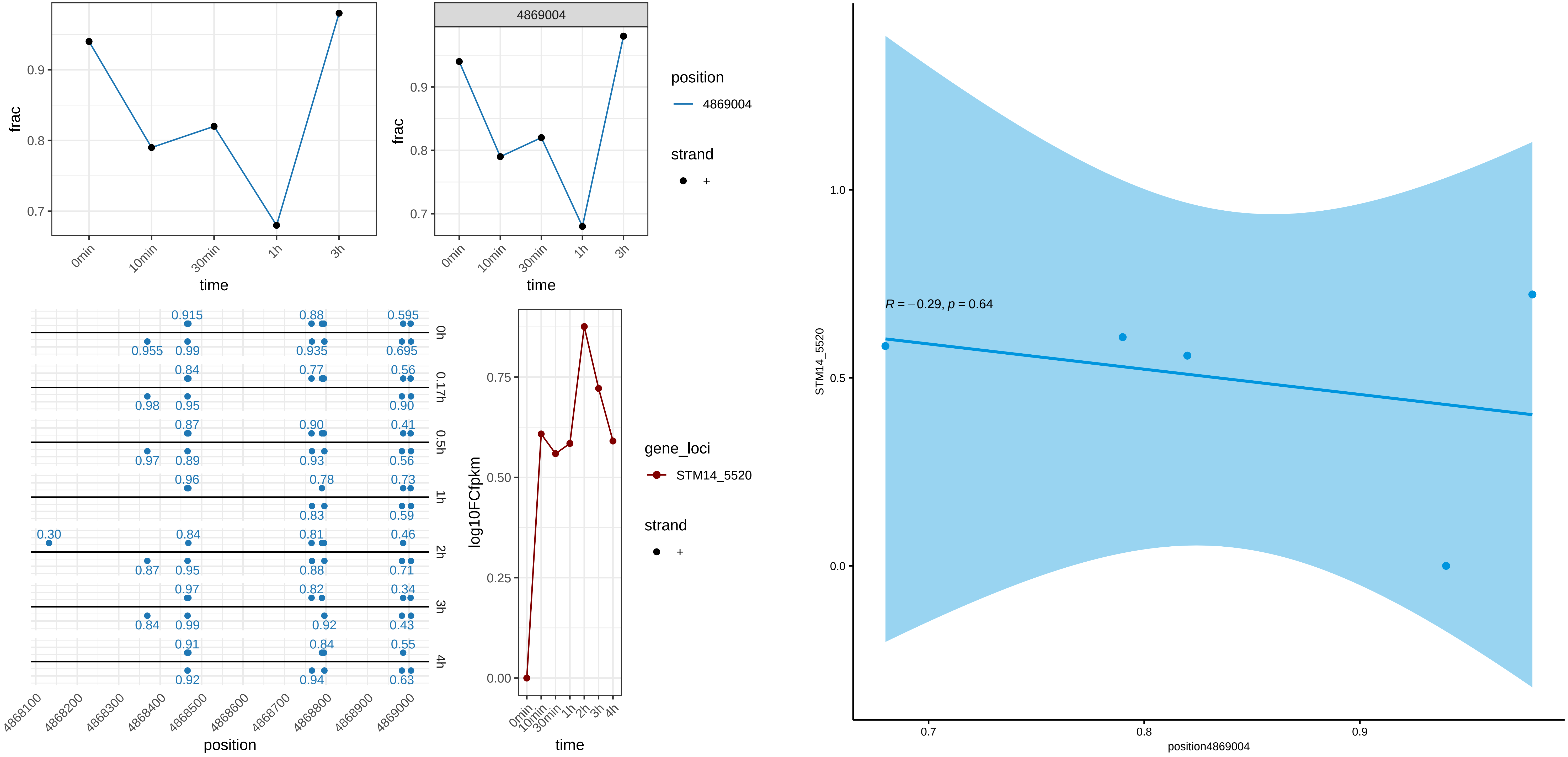

frac
time
0min
10min
30min
1h
3h
4869004
position
strand
+
0.915
0.88
0.595
0.955
0.99
0.935
0.695
0h
0.17h
0.5h
1h
2h
3h
4h
position
log10FCfpkm
gene_loci
STM14_5520
R = −0.29, p = 0.64
STM14_5520
position4869004
