# Supplementary material for: Integrative DNA methylome and transcriptome analysis reveals DNA adenine methylation is involved in Salmonella enterica Typhimurium response to oxidative stress
Source: Microbiol Spectr. 2023 Oct 26;11(6):e02479-23. doi: 10.1128/spectrum.02479-23 (PMC10715015; doi:10.1128/spectrum.02479-23)
Supplement: Table S1 — Bacterial strains and plasmids used in this study [file spectrum.02479-23-s0003.pdf]

Table S1. Bacterial strains and plasmids used in this study

Strain or plasmid	Genetic background	Reference/Source
**Strain**		
S. Typhimurium 14028S	Wild-type	Laboratory stock[1]
Δ*dam*	*dam* mutant (Cm^R) from 14028S	This study
Δ*mod*	*mod* mutant (Cm^R) from 14028S	This study
Δ*dcm*	*dcm* mutant (Cm^R) from 14028S	This study
Δ*STM14_1435*	*STM14_1435* mutant (Cm^R) from 14028S	This study
Δ*hsdM*	*hsdM* mutant (Cm^R) from 14028S	This study
E. coli DH5α	F- 80*lacZ* M15 (lacZYA–argF)U169 *eoR* *recA1endA1 hsdR17 phoA supE*44-thi-1 *gyrA96 relA1*	Laboratory stock[1]
**Plasmid**		
pKD46	Helper plasmid expressing Red recombinase system including γ,β,exo	Laboratory stock[1]
pKD3	The Cm^R template plasmid for generating a liner knockout fragment	Laboratory stock[1]
pCP20	FLP helper plasmid for eliminating antibiotic resistance genes	Laboratory stock[1]
pCDSS	The Spe^R vector with Ara-inducing promoter	Laboratory stock[1]
pCDSS-*dam*	pCDSS reconstructed with *dam* at the *Xho* I and *Spe* I restriction sites	This study

1 Ren, J., Sang, Y., Qin, R., Su, Y., Cui, Z., Mang, Z., Li, H., Lu, S., Zhang, J., Cheng, S. J. E. M. & Infections. Metabolic intermediate acetyl phosphate modulates bacterial virulence via acetylation. **8**, 55-69 (2019).
