# Supplementary material for: Integrative DNA methylome and transcriptome analysis reveals DNA adenine methylation is involved in Salmonella enterica Typhimurium response to oxidative stress
Source: Microbiol Spectr. 2023 Oct 26;11(6):e02479-23. doi: 10.1128/spectrum.02479-23 (PMC10715015; doi:10.1128/spectrum.02479-23)
Supplement: Table S2 — DNA oligonucleotides used in this study [file spectrum.02479-23-s0004.pdf]

Table S2. DNA oligonucleotides used in this study

Primer Name	Primer Sequence
dam-KO-F	GCTGTCGGAGCTTTCTCCACAGCCGGAGAAGGTGTAATT
	AGTTAGTCAGCATGGTGTAGGCTGGAGCTGCTTC
dam-KO-R	ATACCACTCGCGTGTGAGCGCCGTATCATGGTTGGAAAT
	GAGCACCGGTACATATGAATATCCTCCTTAC
dcm-KO-F	TTAGTCTGTCGGCAATTATTTTTGGCCGGTTAAATGTATG
	GTGTAGGCTGGAGCTGCTTC
dcm-KO-R	TAGGCCTGCGTATCCGAGACAGGTATCCTGAACTGGTAC
	GCATATGAATATCCTCCTTAC
mod-KO-F	TGCTGCACCTTAAAATTATCAACGGAGCGAAGATATCATG
	GTGTAGGCTGGAGCTGCTTC
mod-KO-R	TGTGGTTGAGAAGATCGCGGGTCTGCTCTGTTCCCCATC
	GCATATGAATATCCTCCTTAC
STM14_1435-KO-F	GTCTGCATATAGCACACGCACTGACATTGGTGTGAAGGT
	GGAATGAGATGGTGTAGGCTGGAGCTGCTTC
STM14_1435-KO-R	TTTGAAAATGAGTACACCGTGTGGCCGCAATACACGGAA
	CGCCTCTTTGACATATGAATATCCTCCTTAC
hsdM-KO-F	CCCACAATGGTGGTCGCCATGTCAAATAGGATCTTCAAT
	GGTGTAGGCTGGAGCTGCTTC
hsdM-KO-R	CTGCCGCCAGCACGTCCGGCTCCGGCAGACTGTCGGCG
	TCCATATGAATATCCTCCTTAC
dam-check-F	CATCAGGTTCAGGCCGATCT
dam-check-R	CAGTTCGTCCACCTTTTTAC
dcm-check-F	AGTAACCCCTGTCGCTGAAA
dcm-check-R	CTTAACAGGCCAGCAAGACG
mod- check-F	CTTCCTCCCGTCGTTTTCAC
mod- check-R	GCCGATTTCCAGTTCACGAA
STM14_1435-check-F	CGCAATGCATGTCTGCATAT

STM14_1435-check-R	GTCGAGGCCAAACGGAATA
hsdM-check-F	CCAATGTTACAGCGCACCTT
hsdM-check-R	CAACTGACGCTGAGCATCC
pCDSS-*dam*-up-*Xho* I	CCGCTCGAGATGAAAAAAAATCGCGCTTTTTTGAAGT
pCDSS-*dam*-down-*Spe* I	GGACTAGTTTATTTTCTTGCAGGCGTTGCG
pCDSS-check-F	ATGCCATAGCATTTTTATCC
katG-qPCR-F	TAGCGAGATGGTTTCGGTTG
katG-qPCR-R	TTCTAATACCGGCAGAACGC
katE-qPCR-F	CCGGTCTTTGTTCGCTTTTC
katE-qPCR-R	TGCATTGATCAGACGGAAGG
ahpC-qPCR-F	CTTTGTTTGCCCGACTGAAC
ahpC-qPCR-R	TGTGCGTGAAGTGAGTATCG
ahpF-qPCR-F	GTTGCATTAATGCAGTCCCGCG
ahpF-qPCR-R	CAGCAGTTCCTTGATTTCCGCC
rpoS-qPCR-F	GATGAGAACGGAGTAGAGGCTT
rpoS-qPCR-R	CGCGACGCGCAAAATAGACT
xthA-qPCR-F	GATTGTCGAAAAACACCAGCCC
xthA-qPCR-R	CGTGGCTTTTGTTAGCAGCG
dam-qPCR-F	TCCTCGCCGATATCAACAGC
dam-qPCR-R	TCCTGGCAGGTGTTGAACTC
dcm-qPCR-F	TTTCAGTAACACACGCCCGG
dcm-qPCR-R	ATTGTCGCTCAAGCGATGCC
mod-qPCR-F	GAGTCTGTTGCCCCGAATAG
mod-qPCR-R	ACGGATTTTTCACCCGCCTG
hsdM-qPCR-F	GTGGAAACTGTGCGACAACC
hsdM-qPCR-R	GCCTGCACCAGCTTTTTCTC
STM14_1435-qPCR-F	GCGCTACCGTTTGCTGATTC
STM14_1435-qPCR-R	CCGGAATCTGCGTTTCGTTC

16S rRNA-qPCR-F	CAGCCACACTGGAACTGAGA
16S rRNA-qPCR-R	GTGCTTCTTCTGCGGGTAAC
